# Identification and Characterization of an Ageing-Associated 13-lncRNA Signature That Predicts Prognosis and Immunotherapy in Hepatocellular Carcinoma

**DOI:** 10.1155/2023/4615297

**Published:** 2023-02-17

**Authors:** Fulei Li, Xiaofei Xue

**Affiliations:** ^1^Department of Infectious Disease, The First Affiliated Hospital of Zhengzhou University, Zhengzhou 450000, China; ^2^Department of Cardiology, The First Affiliated Hospital of Zhengzhou University, Zhengzhou 450000, China

## Abstract

**Background:**

In cancer pathology, cell senescence not only alters cell function but also reshapes the immune microenvironments in tumours. However, the association between cell senescence, tumour microenvironment, and disease progression of hepatocellular carcinoma (HCC) is yet to be fully understood. Therefore, the role of cell senescence-related genes and long noncoding RNAs (lncRNAs) in evaluating the clinical prognosis and immune cell infiltration (ICI) of HCC patients requires further investigation.

**Methods:**

The *limma* R package was utilised to investigate differentially expressed genes according to the multiomics data. The *CIBERSORT* R package was utilised to assess ICI, and unsupervised cluster analysis was conducted using the R software's *ConsensusClusterPlus* package. A polygenic prognostic model of lncRNAs was constructed by conducting univariate and least absolute shrinkage and selection operator (Lasso) cox proportional-hazards regression analyses. The time-dependent receiver operating characteristic (ROC) curves were used for validation. We utilised the survminer R package to evaluate the tumour mutational burden (TMB). Moreover, the gene set enrichment analysis (GSEA) helped in pathway enrichment analysis, and the immune infiltration level of the model was evaluated using the IMvigor210 cohort.

**Results:**

The identification of 36 prognosis-related genes was achieved based on their differential expression between healthy and liver cancer tissues. Liver cancer individuals were categorised into 3 independent senescence subtypes using the gene list, revealing considerable survival differences (variations). We observed that the prognosis of patients in the ARG-ST2 subtype was substantially better as compared to that in the ARG-ST3 subtype. Differences were observed in gene expression profiles among the three subtypes, with the differentially expressed genes predominantly associated with cell cycle control. The enrichment of upregulated genes in the ARG-ST3 subtype was observed in pathways related to biological processes, for instance, organelle fission, nuclear division, and chromosome recombination. ICI in the ARG-ST1 and ARG-ST2 subtypes, with relatively better prognosis, was substantially higher as compared to the ARG-ST3 subtype. Furthermore, a risk-score model, which can be employed as a reliable prognostic factor in an independent manner for individuals suffering from liver cancer, was constructed based on 13 cell senescence-related lncRNAs (MIR99AHG, LINC01224, LINC01138, SLC25A30AS1, AC006369.2, SOCS2AS1, LINC01063, AC006037.2, USP2AS1, FGF14AS2, LINC01116, KIF25AS1, and AC002511.2). The individuals with higher risk scores had noticeably poor prognoses in contrast with those having low-risk scores. Moreover, increased levels of TMB and ICI were observed in individuals with low-risk scores and gaining more benefit from immune checkpoint therapy.

**Conclusion:**

Cell senescence is an essential factor in HCC onset and progression. We identified 13 senescence-related lncRNAs as HCC prognostic markers, which can help understand their function in the onset and progression of HCC and guide clinical diagnosis and treatment.

## 1. Introduction

Liver cancer is a prevalent disease and a global health problem; by 2025, over 1 million people will be affected by it each year [1–3]. Studies have identified that hepatocellular carcinoma (HCC) is the most widely occurring lung cancer type, which accounts for around 90% of all cases. Upon initial diagnosis of HCC, most individuals are in the advanced stages. Thus, they lose the benefits of early surgical treatment. Therefore, the treatment of choice for advanced HCC is targeted therapy. Presently, the only drug approved by the FDA for targeted therapy in advanced HCC is sorafenib. However, most patients develop primary or secondary drug resistance over time [4]. Although immunotherapy offers a promising new approach to treatment, not all HCC patients can be effectively treated with this strategy [5]. Therefore, the development of new personalised treatment options and intervention strategies is essential for HCC.

Cell senescence is an inherent process in all cells and performs a two-way function in tumour development. Cellular senescence can hinder the development of early tumours, as the senescent cells undergo cell cycle arrest and are subsequently cleared by the immune system [6], which ensures tissue homoeostasis and prevents tumourigenesis [7–9]. On the other hand, if senescent cells are not recognised and cleared by the immune system in time, the accumulated cells release senescence-associated secretory proteins (SASPs), such as cytokines, growth factors, extracellular matrix (ECM) components, and ECM-degrading enzymes, to promote tumour development [10–13]. Furthermore, long noncoding RNAs (lncRNAs) related to other metabolic pathways similar to senescence, such as iron death [14] and autophagy [15], have been used to establish prognostic models of HCC, indicating that metabolic abnormalities perform an essential function in the onset and progression of HCC. However, the correlation between senescence and cancer, especially liver cancer, is highly complicated. According to our knowledge, only a few studies have been conducted to investigate this relationship and explore its clinical applications.

In our research, the assessment of the differentially expressed genes in healthy and tumour tissues was carried out based on the TCGA-LIHC dataset. In total, 36 genes related to disease prognosis were identified. Based on these genes, 3 independent ageing subtypes were identified. These subtypes significantly differ in survival rate, gene expression profiles, pathways, and immune cell infiltration. Furthermore, a combined analysis of overall and cell ageing-related gene (ARG) mutations revealed TP53 and CTNNB1 as the primary mutated genes in liver cancer, and mutations in these genes significantly altered the ARG expression. In order to further enhance the clinical utility of this research, we looked at the differential expression profiles of lncRNAs associated with ageing among the various subtypes. Thirteen cell ageing-related lncRNAs were then verified as independent prognostic markers for patients suffering from liver cancer, and a risk-score model was developed on the basis of these markers. Patients exhibiting various risk scores had different clinical features concerning immunotherapy response, immune cell infiltration (ICI), and tumour mutational burden (TMB). Patients displaying low-risk scores had a good prognosis and responded well to immunotherapy. Overall, the research conducted by our group provides a further comprehension of the regulatory mechanisms of ageing in liver cancer cells and their utility in the clinical assessment of disease prognosis and personalised immunotherapy response.

## 2. Methodology

### 2.1. Clinical Information and Expression Profile Data Acquisition

The TCGA (https://portal.gdc.cancer.gov/) was utilised to download the clinical follow-up and expression data of patients suffering from LIHC. In the TCGA-LIHC dataset, we found preprocessed RNA-seq data of 344 tumour samples, which were processed by following these steps: (1) samples having no clinical follow-up data were eliminated, (2) samples having no specified survival time were eliminated, i.e., <30 days, or absolutely no survival record, (3) conversion of probes to gene symbols, (4) probes that revealed correspondence with multiple genes were eliminated, and (5) evaluation of levels of gene expression in accordance with the median value. The demographic and clinical data are presented in Table 1.

### 2.2. Tumour Immunophenoscore Database

The Cancer Immune Database (TCIA) (https://tcia.at/patients) was employed to acquire tumour immunophenoscore (IPS), which was described according to the characteristics of the ICI in tumours. It is a bridge between ICI and genetic subtypes [16].

### 2.3. Evaluation of Tumour Immune Cell Infiltration

We employed the CIBERSORT R package for infiltration level quantification of a total of 22 cells in the immune system in LIHC in accordance with the LM22 signature at 1000 permutations. The fractions of the following cells were distinguished from each other: resting and activated memory CD4^+^ T cells, naïve B cells, memory B cells, plasma cells, CD8^+^ T cells, naïve CD4^+^ T cells, follicular helper T cells, regulatory T cells (Tregs), gamma delta T cells, activated and resting NK cells, activated and resting dendritic cells, monocytes, M0, M1, and M2 macrophages, activated and resting mast cells, neutrophils, and eosinophils.

### 2.4. Consistent Clustering of Tumour Ageing-Related Gene Expression Profiles

The Human Ageing Genomic Resources (HAGR) helped us identify the central genes related to ageing through the comprehensive analysis of the biology and genetics of human ageing, and ARGs were considered the centre of the network. The R Consensus Cluster Plus package was utilised, and Ward's linkage was employed for unsupervised clustering using the Pam method depending on the Euclidean distance. The repetition was conducted 1000 times in order to confirm the classification stability.

### 2.5. Identification of the Differentially Expressed Genes among Tumour Ageing Subtypes (ARG_DEGs)

Based on the ARG expression in tumours and the outcomes of consistent clustering, the 3 subtypes of tumour samples were established: ARG-ST1, ARG-ST2, and ARG-ST3 groups. Furthermore, the limma R package was utilised to study the differentially expressed genes in the iron death subtypes of TCGA-LIHC tumour samples. Moreover, *P* < 0.05 and |log2 (fold change)| > 1 were set as the screening threshold for elucidating differential expression of genes, and their lncRNAs were obtained utilising the annotation file (^*∗*^.GTF) of genome assembly.

### 2.6. Dimensionality Reduction of Gene Features and Development of a lncRNA Risk-Score Model Related to Ageing

The risk-score model construction for LIHC was carried out in accordance with the ageing-related lncRNAs. Moreover, we employed the univariate Cox regression analysis to decrease noise, eliminate redundant genes, and reduce the lncRNA gene set associated with the ICI subtypes in its size. Followed by the size reduction, the variables were screened via the Lasso [17] method to narrow the gene number in the risk model. Subsequently, we employed multivariate Cox regression analysis in order to carry out the construction of the tumour ICI risk-score model. Finally, the evaluation of the risk score was conducted with the help of the formula given as follows:

(1) $$\begin{array}{c} {\text{Risk}}_{scores}=\sum Coef\left(i\right)*\mathrm{Exp}\left(i\right)\text{.} \end{array}$$

### 2.7. Gene Set Enrichment Analysis

For the interpretation of genome-wide expression profiles, a knowledge-based approach was established in 2005 known as Gene set enrichment analysis (GSEA). In our investigation of gene expression data, the analysis's purpose was determined by choosing one or multiple functional gene sets in MSigDB (gene matrix transposition file format [^*∗*^.GMT]). Subsequently, all the data were classified in accordance with the link between the expression data of genes and phenotypes (interpretable as the change in the expression of genes). Finally, the genes in all the gene sets were checked for enrichment in the lower or upper part of the list after phenotypic association ranking for the examination of the influence of the synergistic change in genes on phenotypic change.

### 2.8. Acquisition of Tumour Somatic Mutation Data

Additionally, the TCGA database (https://www.cancer.gov/tcga/) was employed to download the mutation data of patients available in the TCGA-LIHC dataset. For the assessment of the LIHC's somatic mutation burden, the calculation of the overall nonsynonymous mutations was performed as a quantitative index. The survminer R package was utilised for the evaluation of the optimal density gradient threshold linked to survival and TMB, and the classification of samples was carried out into a couple of TMB groups: the low-TMB group and the high-TMB group. Moreover, the Maftools R package was utilised to perform the comparison of the mutation status of the 30 driver genes between the low- and high-risk score groups.

### 2.9. Acquisition of Immunotherapy Datasets

For the examination of the correlation between immunotherapy and ICI scores and the effectiveness of ICI scores in the prediction of the therapy response of patients, the samples were classified into a couple of groups: the high-rating group and the low-rating group, in accordance with the ICI scoring model by utilising the clinical information and expression data of the IMvigor210 cohort (https://research-pub.gene.com/IMvigor210CoreBiologies/).

### 2.10. Statistical Analysis and Hypothesis Testing

We employed R software (version 3.6) to carry out all the statistical analyses.

## 3. Results

### 3.1. Molecular Parameters of Ageing-Related Genes in Liver Cancer

**Figure 1:**
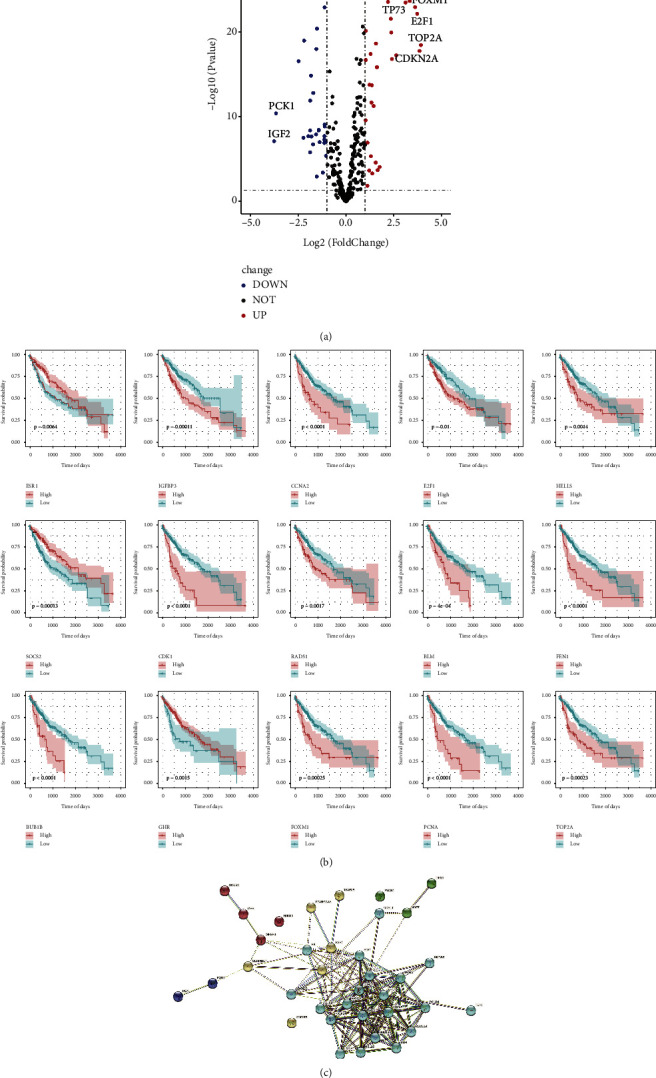
Characteristics of ageing-related genes in TCGA-LIHC dataset: (a) the expression of DEGs illustrated by volcano plot, between LIHC and normal tissue samples, (b) survival curves in accordance with individual gene expression of the leading 15 ageing-related genes and overall survival in the TCGA-LIHC dataset, and (c) string network diagram showing molecular interactions.

**Figure 2:**
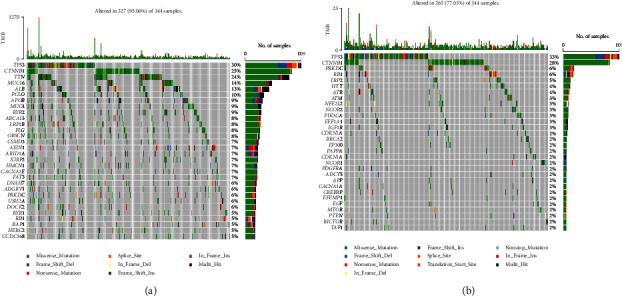
Waterfall plot of gene mutations in TCGA-LIHC dataset: (a) mutation profiles of all gene sets and (b) mutation spectrum of ageing-related genes.

Our analyses of the differential expression of 307 ARGs in healthy and tumour tissues using the TCGA-LIHC dataset revealed 77 differentially expressed genes among both groups (Table S1). In total, 45 genes were upregulated, and the downregulation of 32 was observed in LIHC tumour tissues (Figure 1(a)). Moreover, we employed the univariate Cox analysis for the evaluation of the link between the prognosis and differentially expressed genes of LIHC. Furthermore, with the filtering threshold set at *P* < 0.05 and |beta| > 0.1, 36 genes were identified to exhibit a correlation with disease prognosis (Table S2). Visualising the interaction network of the 36 prognostic genes using the STRING database showed strong interactions between the molecules (Figure 1(c)). The survival curves based on the top 15 of the 36 genes with prognostic significance are illustrated in Figure 2(b). Additionally, the optimal density algorithm was utilised for the classification of the genes into two groups: the low- and high-expression groups. It was observed that high levels of expression of ESR1, SOCS2, and GHR, and low levels of expression of BUB1B, IGFBP3, CDK1, CCNA2, RAD51, FOXM1, E2F1, BLM, PCNA, HELLS, FEN1, and TOP2A were substantially associated with better overall survival (OS) (Figure 1(b)).

Our analyses of gene mutation frequencies using the TCGA-LIHC dataset showed that 95.06% of tumour samples had gene mutations. Among all genes identified, TP53, CTNNB1, TTN, MUC16, and ALB had the highest mutation rates of 30%, 25%, 24%, 14%, and 13%, respectively (Figure 2(a)). The mutation spectrum of ARGs was further assessed (Figure 2(b)), and the top five mutated genes were found to be TP53 (33%), CTNNB1 (28%), PRKDC (6%), RB1 (6%), and LRP2 (5%).

A hypothesis test was conducted to examine whether TP53, CTNNB1, and TTN mutations affected the expression of ARGs. It was observed that the TP53 gene mutations were substantially associated with high levels of expression of BLM, CCNA2, PCNA, BUB1B, RAD51, HELLS, FOXM1, FEN1, CDK1, E2F1, and TOP2A, and low expression levels of ESR1, GHR, and SOCS2 (Supplementary Figure 1). Meanwhile, mutations in the CTNNB1 gene were considerably linked to low levels of expression of GHR, BLM, SOCS2, and IGFBP3 (Supplementary Figure 2). Furthermore, the TTN gene mutations were notably associated with overexpression of ESR1, GHR, SOCS2, and IGFBP3, and low levels of expression of CCNA2, PCNA, BUB1B, HELLS, FOXM1, FEN1, CDK1, E2F1, and TOP2A (Supplementary Figure 3).

### 3.2. Screening of Ageing Subtypes and Differentially Expressed Genes in Hepatocellular Carcinoma

**Figure 3:**
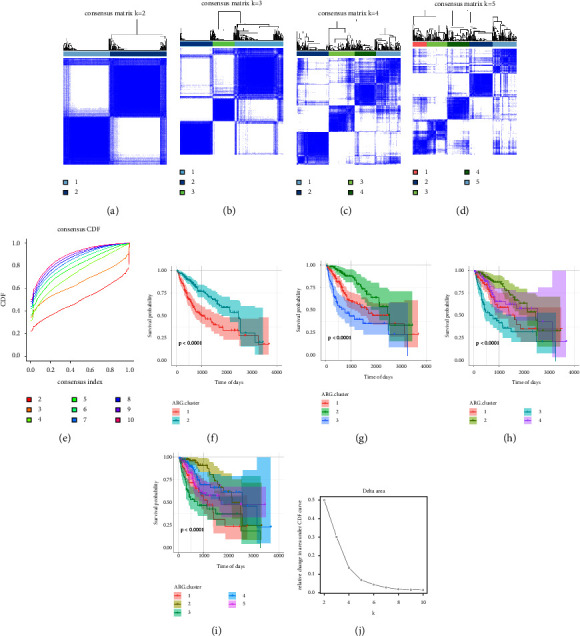
Consistent clustering of the expression profiles of tumour ageing-related genes. Clustering analysis results when the classification number is set at (a) *k* = 2, (b) *k* = 3, (c) *k* = 4, and (d) *K* = 5. (e) CDF curve of consistent clustering. Corresponding survival curves when the classification number is set at (f) *k* = 2, (g) *k* = 3, (h) *k* = 4, and (i) *K* = 5. (j) The area distribution under the CDF curve of consistent clustering.

The consistent cluster analysis was carried out in accordance with the expression of the 36 ARGs in our list and classified 3 independent ageing subtypes (ARG-ST) with evident survival variations. Among the 3 subtypes, disease prognosis in the ARG-ST2 subtype was substantially better compared to the ARG-ST3 subtype, with 2488 days median survival time. Meanwhile, the ARG-ST3 subtype was observed to have a poor prognosis, with 747 days median survival time (Figures 3(a)–3(j)).

**Figure 4:**
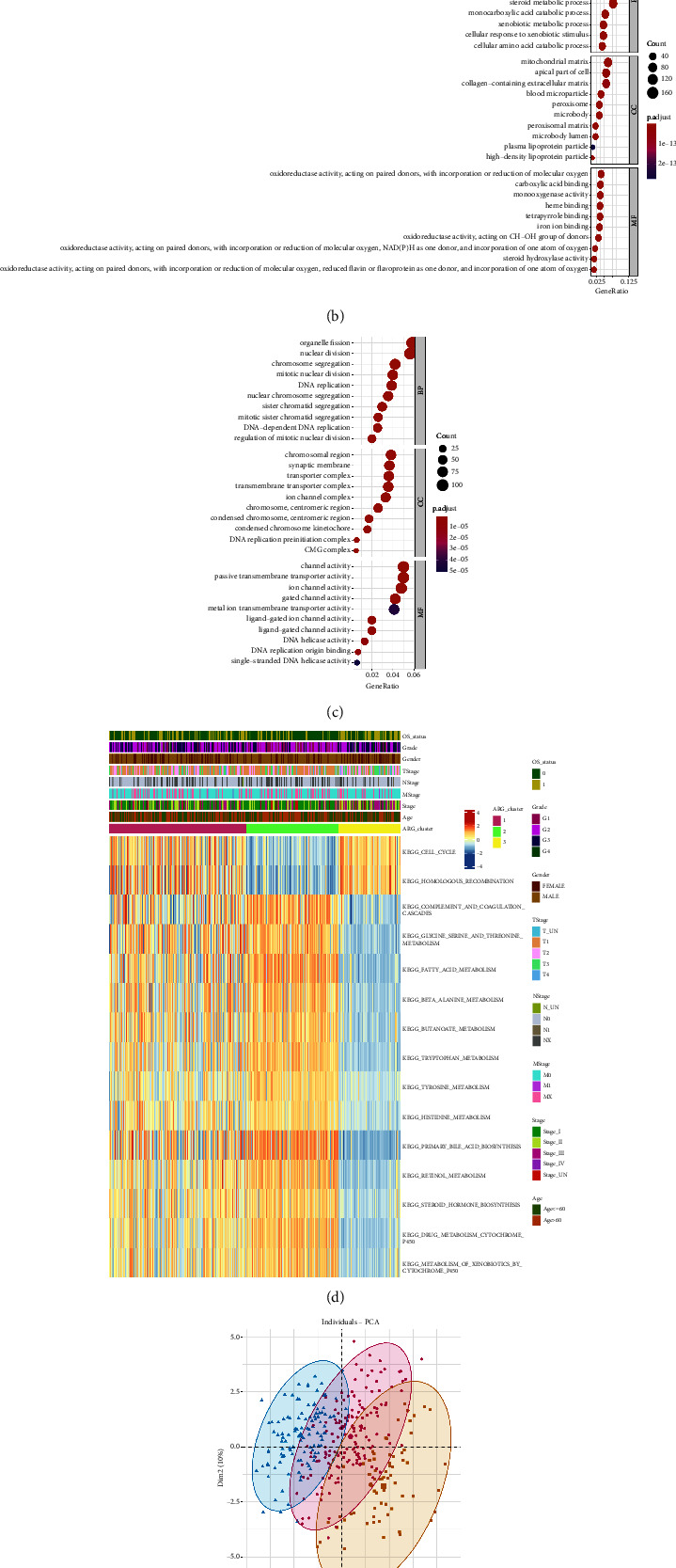
Functional analysis and identification of the differentially expressed genes between ageing subtypes: (a) the differentially expressed genes are illustrated in the volcano plot; (b)-(c) bubble diagram of GO enrichment analysis illustrating the genes that were subjected to downregulation and upregulation; (d) KEGG enrichment analysis; (e) PCA analysis of gene expression profiles; (f) evaluation of immune cell infiltration.

In order to characterise the different ageing states, differential gene expression among the subtypes in the TCGA-LIHC dataset was investigated by employing the limma R package. The identification of the expression of a total of 5363 DEGs was carried out in accordance with the screening threshold of adjusted *P* < 0.05 and |log2 (fold change) | > 1 (Table S3). Out of the total genes, 2867 and 2496 were found to be highly expressed in the ARG-ST3 and ARG-ST2 subtypes, respectively (Figure 4(a)). Subsequently, gene ontology (GO) functional enrichment analysis was carried out on the upregulated genes in various ageing subtypes, and the bubble diagram (Figures 4(b) and 4(c)) was utilised to illustrate the enrichment of 10 pathways in the 3 functional classifications (CC, BP, and MF). Genes with low levels of expression in the ARG-ST3 subtype were enriched in pathways correlated with the biological processes, i.e., small molecule metabolism, cell stress response, and lipid metabolism. Whereas genes with high levels of expression in the ARG-ST3 subtype were enriched in pathways associated with the biological processes, i.e., chromosome recombination, nuclear division, and organelle fission.

Furthermore, GSEA indicated that the first 15 KEGG pathways correlated with the differentially expressed genes were related to cell cycle; homologous recombination; complement and coagulation cascades; metabolism of glycine, serine, threonine, beta-alanine, fatty acid, butanoate, tryptophan, tyrosine, histidine, retinol; primary bile acid and steroid hormone biosynthesis; drug and xenobiotic metabolism via cytochrome p450 (Figure 4(d)).

Moreover, we employed principal component analysis (PCA) in our study to observe the expression profiles of ARGs and investigate the link of the immune cells with tumour ageing subtypes. The samples exhibited good aggregation in the first and second dimensions (Figure 4(e)), demonstrating the reliability of the classification method of ageing subtypes.

In addition, variations in ICI among different ageing subtypes were also compared (Figure 4(f)). The results revealed considerably high infiltration levels of natural B cells (naïve B cells), natural CD4^+^ T cells (naïve CD4^+^ T cells), memory CD4^+^ T cells, resting mast cells, resting NK cells, monocytes, and M2 macrophages in the ARG-ST1 and ARG-ST2 subtypes. Meanwhile, the ARG-ST3 subtype had significantly high infiltration levels of memory B cells, M0 macrophages, Tregs, and follicular helper T cells.

### 3.3. Construction of a Risk-Score Model for Liver Cancer Based on Ageing-Related lncRNAs

**Figure 5:**
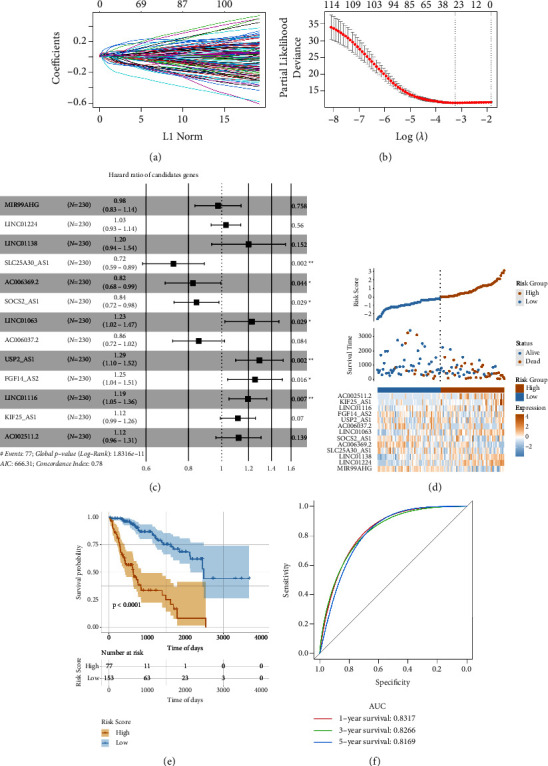
Risk model construction and lncRNAs screening: (a) changes in all the independent variables. The graph's horizontal axis represents the log value of the independent variable lambda, whereas the vertical axis represents the coefficient of the independent variable, (b) the confidence interval of all the variables, (c) multivariate cox regression analysis, (d) risk scores' distribution map, (e) Kaplan–Meier survival curve, and (f) the ROC curves with the AUC values.

This study examined the function of ageing-related lncRNAs in predicting overall survival (OS). A total of 346 differentially expressed lncRNAs among the different ageing subtypes were identified (Table S4). Furthermore, analyses revealed that 180 were upregulated and 166 were downregulated in the ARG-ST3 subtype. The construction of a risk-score model for ICI was carried out in accordance with the differential expression of the lncRNAs. Samples from the TCGA-LIHC dataset (*n* = 344) were grouped into the training (*n* = 230) and test (*n* = 114) sets in an approximate ratio of 2 : 1. From a total of 346 candidate lncRNAs identified in the training set by utilising univariate Cox analysis, 116 were retained on the basis of the threshold of *P* value <0.05 (Table S5). Moreover, for the investigation of clinical utility, Lasso was used for screening the variables, further highlighting 13 lncRNAs from the initial list (Figures 5(a) and 5(b)), and based on these lncRNAs, construction of a risk-score model related to ICI was carried out via multivariate Cox regression (Figure 5(c)).

The formula for the computation of the risk score on the basis of 13 lncRNAs gene signature is as follows:

Risk Score = (−0.025)*∗*MIR99AHG+ (0.030)*∗*LINC01224 + (0.182)*∗*LINC01138 + (−0.326)*∗*SLC25A30AS1 + (−0.194)*∗*AC006369.2 + (−0.170)*∗*SOCS2AS1 + (0.204)*∗*LINC01063 + (−0.155)*∗*AC006037.2 + (0.256)*∗*USP2AS1 + (0.226)*∗*FGF14AS2 + (0.177)*∗*LINC01116 + (0.109)*∗*KIF25AS1 + (0.115)*∗*AC002511.2.

The link between the OS and risk score was investigated using the constructed model. The samples were classified into two groups: the low- and high-risk groups by utilising the R software package ggrisk and the optimal density gradient algorithm (Figure 5(d)). A higher death proportion was observed in samples of the high-risk group. Moreover, Kaplan–Meier analysis revealed that the patients' OS in the high-risk group were considerably lower in comparison with the low-risk group (Figure 5(e)). Additionally, our risk-score model reliably predicted the patients' OS in the TCGA-LIHC dataset, as shown by the respective AUC values, predicting 1-, 3-, and 5-year OS at 0.8317, 0.8266, and 0.8169 (Figure 5(f)).

**Figure 6:**
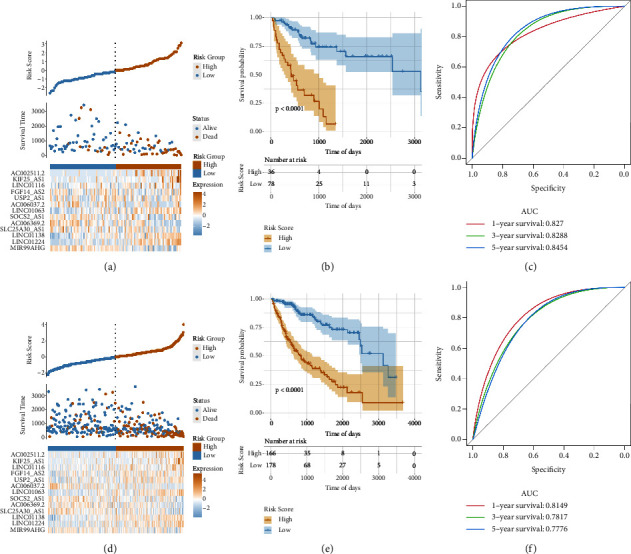
Validation of the risk model in the test and TCGA-LIHC sets: (a) distribution map of the risk scores of the test set, (b) the test set-related survival curve, (c) the test set-related ROC curve predicting 1-, 3-, and 5-year OS, (d) the overall risk score-related distribution map, (e) survival curve representing the TCGA-LIHC set, and (f) ROC curves representing the TCGA-LIHC set for predicting 1-, 3-, and 5-year OS.

**Figure 7:**
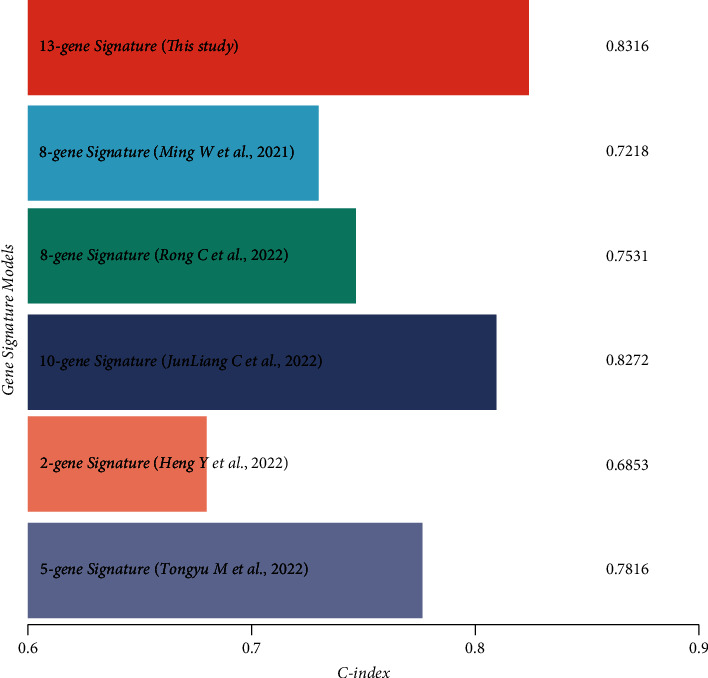
C-indexes comparisons of the 6 prognostic risk signatures.

We employed a similar strategy in evaluating the risk score model's effectiveness in estimating the OS of the test group and entire TCGA-LIHC datasets. As previously described, the samples were classified into the high- and low-risk groups using the R software package ggrisk and the optimal density gradient algorithm (Figures 6(a) and 6(d)). Consistent with our results in the training group, the samples in the high-risk group displayed a higher proportion of death events. Similar to previous results, Kaplan–Meier analysis revealed that the OS of the individuals in the high-risk group was considerably lower in comparison to the individuals in the low-risk group (Figures 6(b) and 6(e)). Furthermore, our risk-score model had a good ability to calculate the OS of patients in the test set as shown by the respective AUC values, predicting 1-, 3-, and 5-year OS at 0.8270, 0.8288, and 0.8454 (Figure 7). Similarly, in the overall TCGA-LIHC dataset, the risk-score model displayed a good ability to predict the OS of individuals, revealed by the respective AUC values, predicting 1-, 3-, and 5-year OS at 0.8149, 0.7817, and 0.7776 (Figure 6(f)).

For further evaluation of the effectiveness of our risk-score model, an assessment of the OS of patients included in the GSE14520 dataset from the GEO database was conducted. Samples classification was performed to establish two groups: the low- and high-risk groups, as previously described (Supplementary Figure 4(a)). Consistent with previous findings, individuals in the high-risk group displayed a high percentage of death and a considerably lower OS than individuals in the low-risk group (Supplementary Figure 4(b)). Furthermore, high AUC values were also noted during the prediction of 1-, 3-, and 5-year OS.

### 3.4. Comparison of Different Signatures

Based on previous publications, 5 prognostic risk models were selected, including 8-gene [18], 8-gene [19], 10-gene [20], 2-gene [21], and 5-gene signatures [22], and compared these signatures with our model. The *rms* package in R was employed to calculate the concordance index (C-index) of the various signatures and to compare the prediction performance of these models in HCC samples. Our results demonstrated that our signature's average C-index was substantially higher than the other signatures (Figure 7), suggesting that the overall performance of the 13-gene signature outweighed that of the other 5 signatures.

### 3.5. Relationship between the Clinical Characteristics and Risk Score

**Figure 8:**
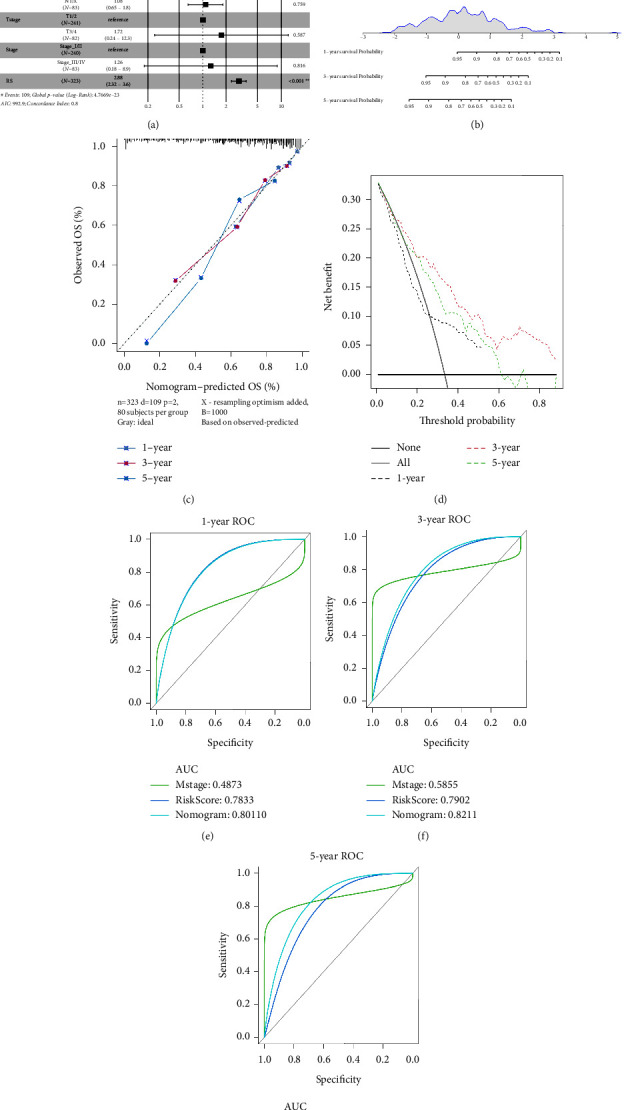
Correlation between the clinical characteristics and risk score: (a) multivariate cox analysis of risk score and clinical features; (b) nomogram of risk score and clinical features; (c) calibration diagram of the nomogram for predicting 1-, 3-, and 5-year OS; (d) DCA of the nomogram for prediction of 1-, 3-, and 5-year OS; (e)–(g) ROC curves for prediction of 1-, 3-, and 5-year OS, respectively.

It is necessary to describe the correlation between clinical characteristics (age, tumour grade, etc.) and patients' risk scores. Therefore, the multivariate Cox analysis was utilised to examine the effectiveness of the risk score as a prognostic factor irrespective of age, sex, tumour stage, and M-, N-, and T-stages (Figure 8(a)). The M-stage and risk score were recognised as independent prognostic factors and were utilised for the construction of a nomogram for assessing clinical utility (Figure 8(b)). Moreover, the calibration curves of the nomogram for predicting 1-, 3-, and 5-year OS indicated good stability of the system (Figure 8(c)). For further assessment of the clinical effectiveness of the nomogram, we conducted DCA and found that the net ability of the nomogram in predicting 3-year OS was considerably higher than its ability in predicting 1- and 5 -years OS (Figure 8(d)). Additionally, the ROC curve revealed that the prognostic ability of the nomogram in OS prediction of 1, 3, and 5 years (AUC >0.80 for all) was higher than that of other indicators (Figures 8(e)–8(g)).

### 3.6. Correlation between the Tumour Mutational Burden and Risk Score

**Figure 9:**
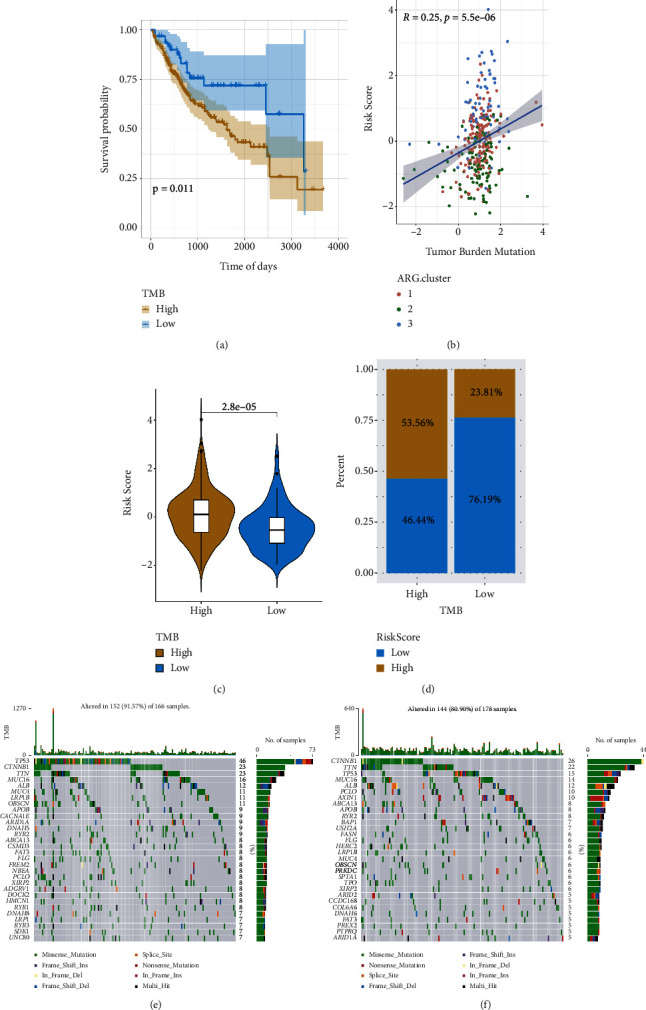
Association between the tumour mutational burden and risk score: (a) KM survival curve based on the TMB scores, (b) correlation linear regression analysis, (c) violin plot, (d) proportional distribution bar chart, (e) waterfall plot to evaluate gene mutations observed in the patients in the high-risk subgroup, and (f) waterfall plot to evaluate gene mutations observed in the individuals in the low-risk subgroup.

Several studies have suggested that TMB determines an individual's response to cancer immunotherapy. The correlational analysis of the risk scores and TMB was conducted to evaluate the genetic characteristics of all the ageing subgroups. The optimal density gradient threshold was evaluated by employing the survminer R package linked to survival and TMB. The classification of the tumour samples in the TCGA-LIHC dataset was completed by creating two groups: the low-TMB score group and the high-TMB score group. The subsequent study suggested that the low TMB score (Figure 9(a)) had a higher survival rate. Linear regression analyses revealed a highly positive association between the risk score and TMB (Figure 9(b)). In addition, the TMB of patients was considerably higher in the high-risk subgroup compared to the individuals in the low-risk subgroup (Figures 9(c) and 9(d)).

Subsequently, for the characterization of the mutational burden, the somatic variation distribution in LIHC driver genes across the high- and low-risk subgroups was evaluated, and a comparison of the 30 essential driver genes with the highest variation frequencies was performed (Figures 9(e) and 9(f)). Analyses of the mutation annotation file of the TCGA-LIHC cohort displayed substantial variations in the mutation spectrum between the patients in the low- and high-risk subgroups. Our findings may serve as bases for future research on the impacts of ageing on gene mutations in immune checkpoints during cancer development.

### 3.7. Correlation between the Risk Score and Immune Cell Infiltration

**Figure 10:**
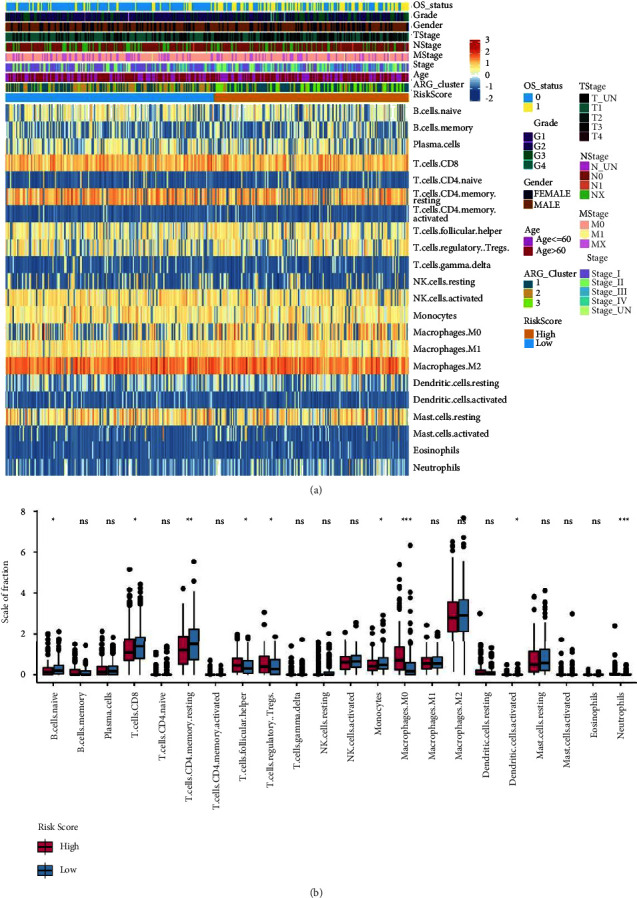
Association between the risk score and immune cell infiltration: (a) the proportion of immune cell infiltration is illustrated by a distribution heat map and (b) the variations in immune cell infiltration between individuals in low- and high-risk groups are illustrated via box plot.

The link between the tumour immune microenvironment and risk score was studied using the CIBERSORT tool and determining the infiltration status of 22 immune cells in the TCGA-LIHC dataset. High levels of infiltrating immune cells, including resting memory CD4^+^ T cells, CD8^+^ T cells, follicular helper T cells, Tregs, activated NK cells, monocytes, resting mast cells, and M1/M2 macrophages, were observed to be common in LIHC. Meanwhile, low infiltration levels of natural CD4^+^ T cells (naïve CD4 T cells) and activated memory CD4^+^ T cells were observed (Figure 10(a)).

A hypothesis test was carried out to examine the variations in ICI among patients in the low-risk and high-risk groups. The individuals in the high-risk group revealed considerably higher infiltration levels of Tregs, M0 macrophages, and follicular helper T cells and substantially lower infiltration levels of CD8^+^ T cells, natural B cells (naïve B cells), resting memory CD4^+^ T cells, and monocytes as compared to the individuals in the low-risk group (Figure 10(b)).

### 3.8. Assessing the Ability of the Risk Model in Predicting Immunotherapy Response

**Figure 11:**
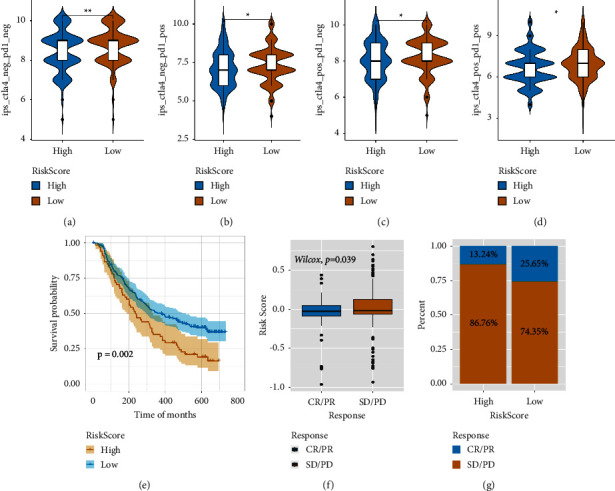
The function of risk scores in the prediction of immunotherapeutic benefits. (a)–(d) Variations in the four types of IPS between individuals in low- and high-risk groups. (e) Variations in risk scores among various treatment response groups in the IMvigor210 cohort. (f) Survival curve of the low- and high-risk scores in the IMvigor210 cohort. (g) Variations in therapy responses between the low- and high-risk groups in the IMvigor210 cohort.

The efficiency of the risk score to predict the immunotherapy response was assessed in accordance with the IPS of TCGA-LIHC samples available in the IMvigor210 dataset (https://researchpub.gene.com/IMvigor210CoreBiologies) and TCIA database. IPS can determine tumour immunogenicity and help in the prediction of the response of different tumour types to immunotherapy (Figures 11(a)–11(d)). The scores of four types (ipsctla4negpd1neg, ipsctla4pospd1neg, ipsctla4negpd1pos, and ipsctla4pospd1pos) of IPS were considerably higher in the patients in the low-risk group in comparison to individuals in the high-risk group, indicating that the immunotherapy will be more beneficial to individuals in the low-risk group. Furthermore, patients in the IMvigor210 cohort received anti-PD-L1 immunotherapy, and a low or high-risk score was assigned to them. We observed that individuals in the low-risk group lived a long life as compared to the patients in the high-risk group (Figure 11(f)). Moreover, individuals in the low-risk group revealed a higher objective response rate to anti-PD-L1 therapy (CR/PR) than the high-risk group (Figures 11(e) and 11(g)). Overall, there is an indication in our data that risk scores have a substantial correlation with immunotherapy response.

## 4. Discussion

Cell ageing is a complex biological process that performs a vital function in remodelling the cellular microenvironment [10, 23]. Physiological ageing plays a vital role in inhibiting tumour progression by eliminating senescent cells through the immune response initiated by SASPs [24]. However, as cell ageing progresses, the accumulation of SASP increases, promoting tumour development through immunosuppression [11, 12, 25, 26]. Although it is an essential cellular process, the underlying mechanism of how cell ageing regulates tumour progression remains unclear. Therefore, our research analysed the differential expression of ARGs in HCC and healthy tissues and determined their potential role in evaluating disease prognosis. We found that these genes do not exist independently but instead have a close relationship with each other. Furthermore, we assessed the possible causes of the aberrant expression of these genes. We first focussed on gene mutations and found that the mutation frequency in the overall genome of tumour samples and ARGs was abnormally increased to 95.06% and 77.03%, respectively. Consistent with previous studies, we observed that TP53 and CTNNB1 genes had high mutation rates in the two groups.

TP53 is a transcriptional gene of the tumour suppressor protein p53 and has the highest mutation rate in human tumours. Approximately 50% of patients with tumours harbour TP53 gene mutations, and about 80% of patients have TP53 dysfunction [27]. It performs a critical function in maintaining genomic stability and cell homoeostasis [28, 29]. When cells experience extreme stimuli, activated p53 protein can eliminate damaged and potential precancerous cells by inducing cell cycle arrest, apoptosis, and ageing [30]. Additionally, p53 as a transcription factor can inhibit or activate the transcription of target genes, such as genes regulating cell cycle and apoptosis, by directly binding to specific DNA sequences [31–34]. In this study, we found that TP53 mutation can lead to an aberrant expression of ARGs. These genes include DNA damage repair genes, i.e., FEN1 and RAD51, and cell cycle-related genes, i.e., CDK1 and E2F1.

CTNNB1 is a transcriptional gene of the classical oncogenic protein *β*-catenin, and mutation in this gene abnormally activates the Wnt pathway in osteosarcoma, thus promoting tumour progression [35]. In this study, CTNNB1 mutation led to abnormally low expression of some genes, suggesting that ARGs are likely to be regulated by mutations in major tumour suppressors or tumour-promoting genes, and hence, may promote tumour progression. However, our study could not elucidate the association between our prognostic risk model and the mutational burden on these specific genes.

Furthermore, we genotyped HCC samples in accordance with ARG expression and classified 3 independent ageing subtypes with considerable survival variations. Among the 3 subtypes, the ARG-ST2 subtype prognosis was notably better as compared to the ARG-ST3 subtype, and significant differences were observed in gene expression among them. Genes upregulated in the ARG-ST3 subtype were enriched in pathways linked to biological processes, i.e., organelle fission, nuclear division, and chromosome recombination. Overall, the cell cycle was the most important pathway associated with these DEGs [36, 37]. Based on the gene enrichment in these cellular processes, our findings further support the ongoing idea that abnormal activation of cell division leads to tumour progression and an overall poor prognosis.

ARGs can not only affect individual cells but can also reshape the tumour microenvironment [10, 11] and promote the progression of tumours as well. In line with this, we determined the difference in ICI among different ageing subtypes. In the ARG-ST1 and ARG-ST2 subtypes with good prognosis, tumour suppressor immune cell infiltration, for instance, B cells [38] and CD4^+^ T cells [39], was observed to be considerably increased. Moreover, the infiltration of cancer-promoting immune cells, for example, M2 macrophages, was also increased simultaneously [40, 41]. This finding is not entirely consistent with previous studies. Whether this phenomenon is related to the early or late accumulation of ageing cells remains unclear. Moreover, we did not construct a model that can simulate the dynamic development process of ageing cells at different time points.

Furthermore, we established prognostic gene signatures based on ageing-related differentially expressed lncRNA genes to simplify the clinical application of ageing-related genes. lncRNAs are RNA transcripts that cannot encode polypeptides, generally consisting of more than 200 nucleotides [42]. Research has found that they perform a significant function in the onset and progression of many diseases, including tumours. After conducting lasso regression analysis, 13 lncRNAs (MIR99AHG, LINC01224, LINC01138, SLC25A30AS1, AC006369.2, SOCS2AS1, LINC01063, AC006037.2, USP2AS1, FGF14AS2, LINC01116, KIF25AS1, and AC002511.2) with the maximum frequency were obtained. Some studies on these lncRNAs have revealed their role in tumours. For example, MIR99AHG, as a tumour progression-inhibiting factor, can delay lung cancer progression by synergistically promoting autophagy in lung cancer [43]. However, in gastric cancer, it can promote EMT and the progression of gastric cancer [44]. LINC01224 performs a consistent role in tumours, and current studies have revealed that it promotes progression in different types of tumours, i.e., gastric cancer [45], colorectal cancer [46], and lung cancer [47]. Studies on LINC01138 in tumours are limited, but the current results show the progress of LINC01138 in liver cancer [48] and kidney cancer [49]. SLC25A30AS1 and LINC01063 are only shown to be constituent factors in the tumour prognosis model, but their specific role in tumours has not been studied [50, 51]. SOCS2AS1 promotes tumour progression in prostate cancer [52] but inhibits tumour progression in epithelial, ovarian, and colorectal cancer [53–55]. USP2AS1 is a cancer-promoting factor that promotes tumour progression by different mechanisms in various types of tumours, including ovarian and colorectal cancer [56–58]. FGF14AS2 inhibits tumour progression in many ways, such as inhibiting metastasis by regulating the miR-370-3p/FGF14 axis [59]. Its overexpression inhibits cell proliferation through RERG/Ras/ERK signalling [60] but promotes glioma progression via glioma microRNA-1288-3p [61]. LINC01116 is a poor prognostic factor for different types of tumours and plays a role in tumour progression [62]. For AC006369.2, AC006037.2, KIF25AS1, and AC002511.2, there is no research to explore their role in tumours. The diversity of these lncRNAs also reflects the complex mechanism of cell ageing in tumours, but we lack effective *in vivo* verification in this part.

Moreover, patients suffering from HCC with high-risk scores showed a considerably worse prognosis than those with low-risk scores. Additionally, it was revealed that HCC patients with high prognostic risk scores were at an advanced tumour stage, i.e., M stage and T stage, a potential reason for the poor prognosis. However, the risk score was identified as an independent and stable prognostic factor, as confirmed by our ROC curve analysis. Furthermore, we also compared the characteristics of TMB and the immune microenvironment in patients with low- and high-risk scores. We found that risk scores have a positive association with TMB, which is defined as the total number of somatic coding mutations, which often leads to the emergence of new antigens that trigger antitumour immunity [63]. Studies have revealed that higher mutation rates in certain tumours can result in the emergence of novel antigens, which play a vital role in the activation of immune cells [64]. Currently, TMB has been identified as an emerging biomarker for evaluating tumour sensitivity to immune checkpoint inhibitors [65]. In this research, the risk score was observed to have a positive correlation with TMB, which can mean that an increase in risk score leads to increased effectiveness of immunotherapy. However, the actual results do not seem to be consistent with it, and whether the difference in effective new antigens caused by the difference in mutant gene subgroups leads to the fact that the overall level of TMB cannot represent the level of effective immune activation because we found that the type of mutation changed in parallel with the risk score and if this change is caused by cell ageing remains to be determined.

From another perspective, the characteristics of the infiltrating immune cell population are also important. Significant variations in the distribution of immune cells between the scoring groups were also observed; for example, the infiltration levels of the tumour suppressor CD8^+^ T cells were substantially higher in the individuals of the low-risk group, suggesting that it may be one of the contributors to a good prognosis. Similarly, the low-risk group displayed a higher objective response rate to anti-PD-L1 therapy as compared to the high-risk group. Studies have shown that potential markers, including PDL1 expression, TMB, and MSI levels, can be used as immunosuppressants in tumours. In accordance with this, patients with high TMB generally have high response rates to PDL1 [66]. However, we found several contradictions in our study. Our research findings indicate that a single index in the complex regulatory network of the tumour microenvironment may not be efficient for predicting the effectiveness of immune checkpoint inhibitors. Therefore, we suggest that the risk score can be used as a more intuitive index for evaluating the efficacy of immune checkpoint inhibitors.

Additionally, our study has a few limitations that should be considered. First, the TCGA dataset's population composition is limited mainly to Caucasians and African–Americans. Hence, extrapolation of the results to other racial groups would need to be confirmed. Second, a robust multigene signature should be externally validated using different cohorts; therefore, our model needs further validation in multicentre clinical trials and prospective studies. Finally, the genes included in the signature need to be validated at the biological level to lay the foundation for clinical studies.

In conclusion, we systematically analysed ARGs in HCC, identified 36 genes related to prognosis, and classified HCC based on these genes. Three independent ageing subtypes with substantial survival variations were obtained, and the three subtypes had significantly different gene expression profiles, cell pathway enrichment, and ICI. Additionally, CTNNB1 and TP53 mutations were identified as the main mutations of ARGs in HCC, which can significantly alter the expression of ARGs. Furthermore, in accordance with the expression profiles of ageing-related lncRNAs among different subtypes, a risk-score model incorporating 13 lncRNAs was constructed and validated as an independent prognostic factor for individuals suffering from HCC. Moreover, patients exhibiting different risk scores demonstrated different clinical characteristics, TMB, ICI, and response to immunotherapy. Patients with low-risk scores exhibited good prognoses and responded well to immunotherapy. Altogether, we found that the risk score was an independent prognostic factor for patients with HCC and can serve as a predictive biomarker of immune checkpoint inhibitor response when combined with TMB assessment.

## Figures and Tables

**Table 1:** Clinical and demographic data of samples included from the TCGA-LIHC dataset.

	Number of samples
Survival	
OS	
Status 0	221
Status 1	123

Grade	
G1	53
G2	162
G3	112
G4	12
G unknown	5

Age	
Age >60 years	179
Age ≤60 years	165
Sex	
Female	109
Male	235

Stage	
Stage I	162
Stage II	78
Stage III	80
Stage IV	3
Stage V	21

M stage	
M0	245
M1	3
MX	96
Mun	0

N stage	
N0	240
N1	3
N2	1
NX	100
Nun	0

T stage	
T1	169
T2	85
T3	74
T4	13
Tun	3

Pharmaceutical therapy (PT)	
Yes	29
No	110
Un	205

Radiation therapy (RT)	
Yes	9
No	129
Un	206

## Data Availability

The datasets used for this study are included in the article.

## Supplementary Materials

Supplementary Materials Supplementary Figure 1: impacts of TP53 gene mutations on the expression of 15 ageing-related genes in the TCGA-LIHC dataset. Supplementary Figure 2: impacts of CTNNB1 gene mutations on the expression of 15 ageing-related genes in the TCGA-LIHC dataset. Supplementary Figure 3: impacts of TTN gene mutations on the expression of 15 ageing-related genes in the TCGA-LIHC dataset. Supplementary Figure 4: validation of the risk model in the external dataset GSE14520: (a) distribution map of risk scores, (b) KM survival curve, and (c) ROC curves for predicting 1-, 3-, and 5-year OS. Table S1: 77 differentially expressed genes identified among both groups. Table S2: 36 genes were identified to exhibit a correlation with disease prognosis. Table S3: the identification of the expression of 5363 DEGs. Table S4: a total of 346 differentially expressed lncRNAs among the different ageing subtypes. Table S5: a total of 116 candidate lncRNAs identified in the training set by utilising univariate Cox analysis.
