# Supplementary material for: Identification and Characterization of an Ageing-Associated 13-lncRNA Signature That Predicts Prognosis and Immunotherapy in Hepatocellular Carcinoma
Source: J Oncol. 2023 Feb 17;2023:4615297. doi: 10.1155/2023/4615297 (PMC9957638; doi:10.1155/2023/4615297)
Supplement: Supplementary Materials — Supplementary Figure 1: impacts of TP53 gene mutations on the expression of 15 ageing-related genes in the TCGA-LIHC dataset. Supplementary Figure 2: impacts of CTNNB1 gene mutations on the expression of 15 ageing-related genes in the TCGA-LIHC dataset. Supplementary Figure 3: impacts of TTN gene mutations on the expression of 15 ageing-related genes in the TCGA-LIHC dataset. Supplementary Figure 4: validation of the risk model in the external dataset GSE14520: (a) distribution map of risk scores, (b) KM survival curve, and (c) ROC curves for predicting 1-, 3-, and 5-year OS. Table S1: 77 differentially expressed genes identified among both groups. Table S2: 36 genes were identified to exhibit a correlation with disease prognosis. Table S3: the identification of the expression of 5363 DEGs. Table S4: a total of 346 differentially expressed lncRNAs among the different ageing subtypes. Table S5: a total of 116 candidate lncRNAs identified in the training set by utilising univariate Cox analysis. [file 4615297.f1.pdf]

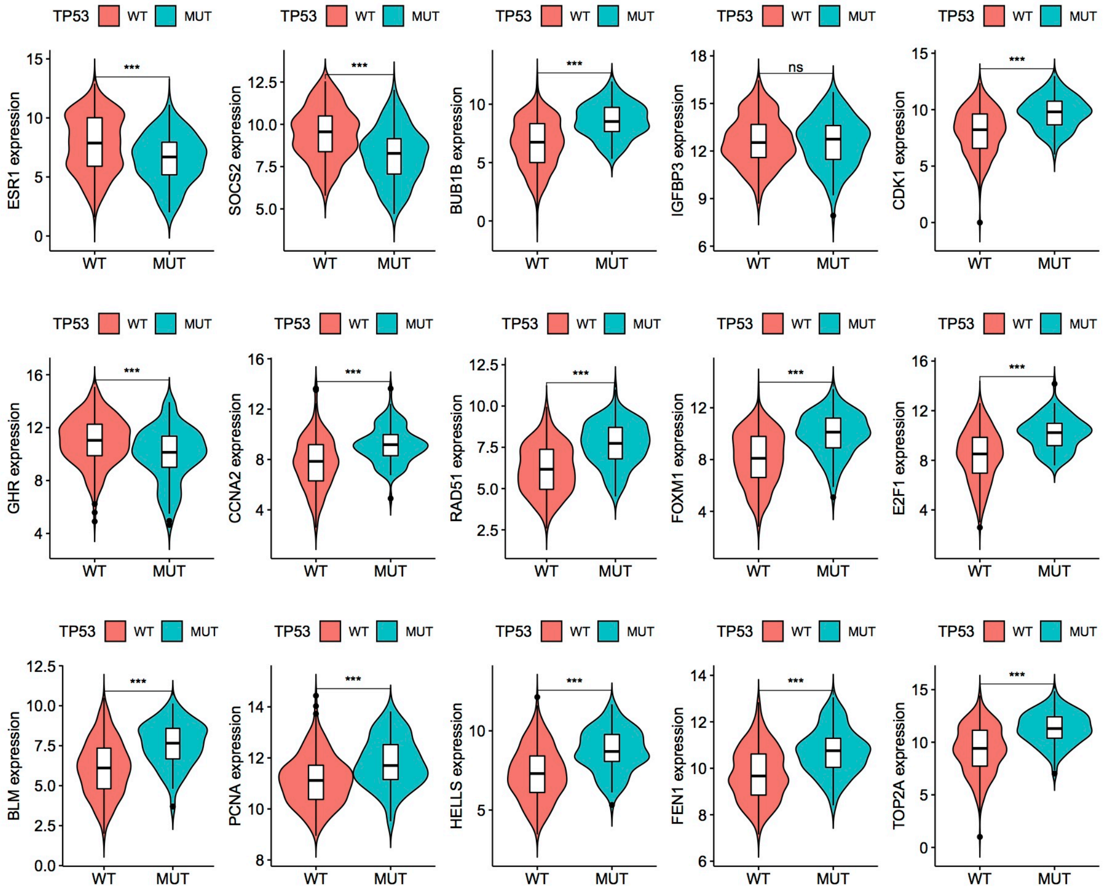

Supplementary Figure 1. Effects of TP53 gene mutations on the expression of 15 ageing-related genes in TCGA-LIHC dataset.

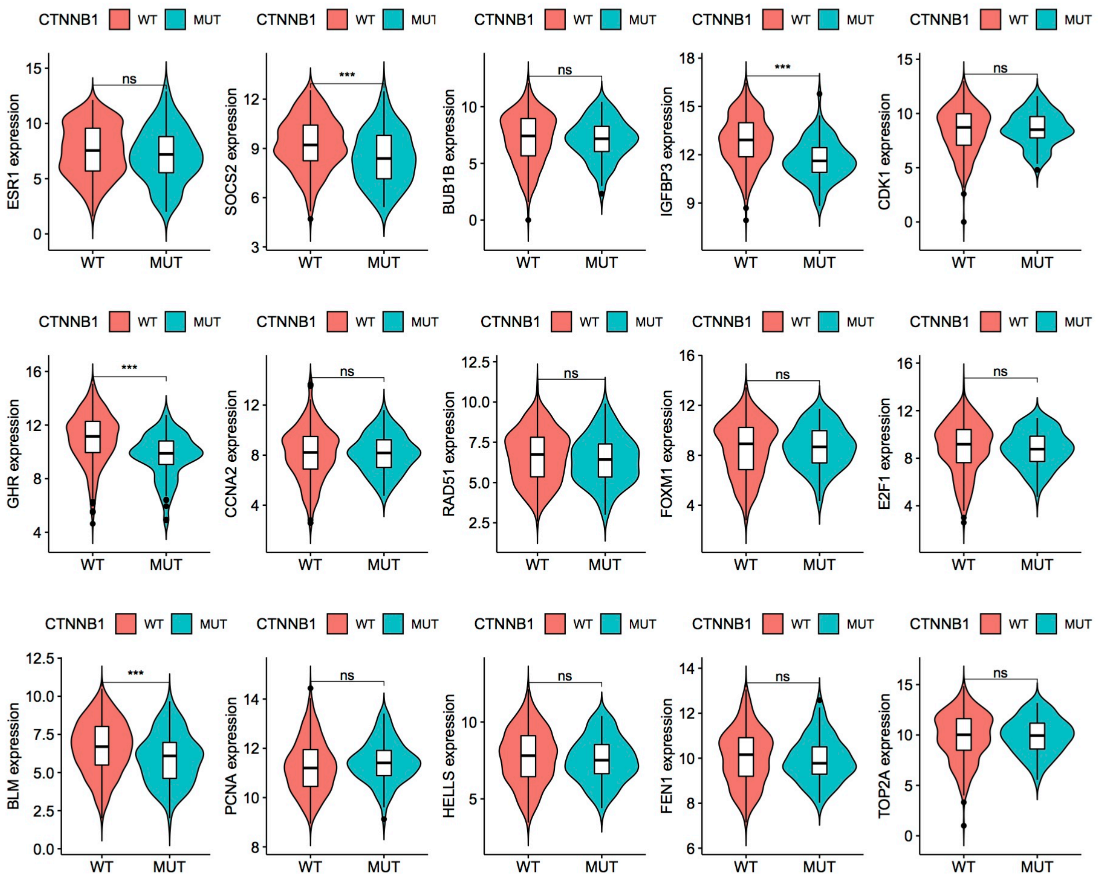

Supplementary Figure 2. Effects of CTNNB1 gene mutations on the expression of 15 ageing-related genes in TCGA-LIHC dataset.

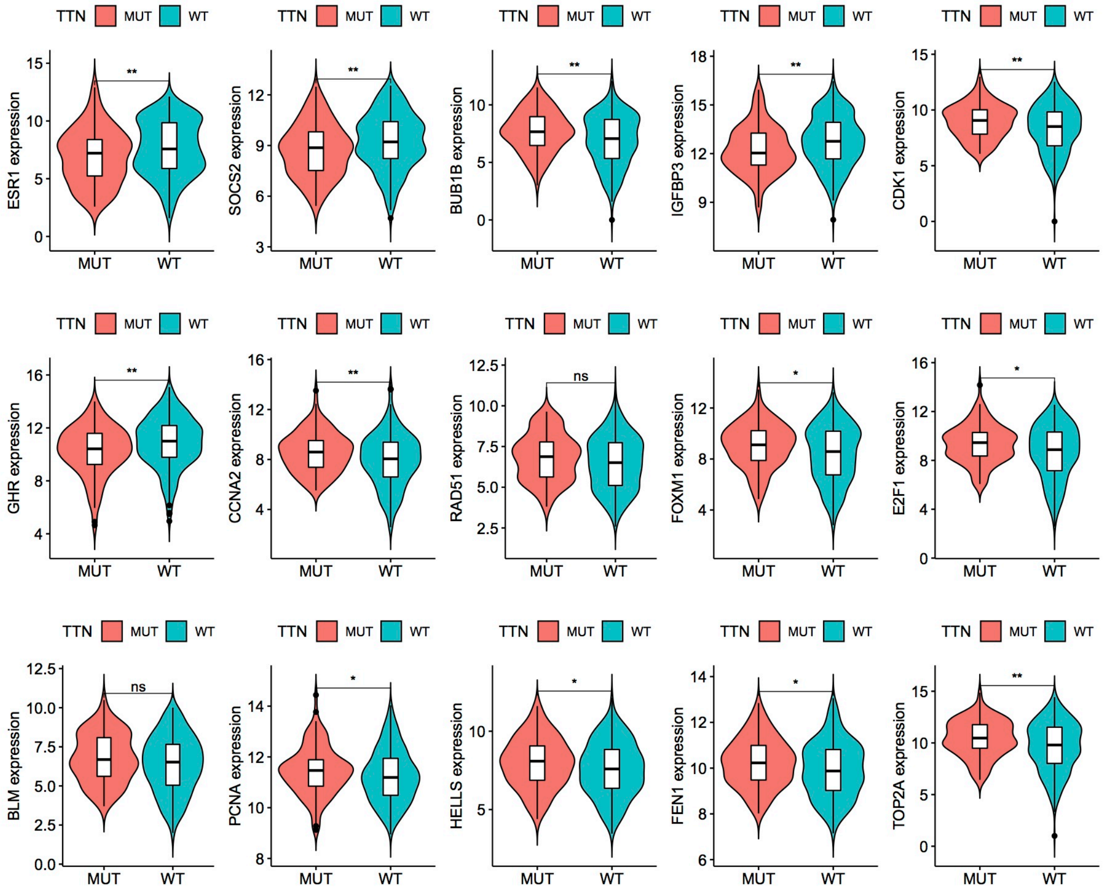

Supplementary Figure 3. Effects of TTN gene mutations on the expression of 15 ageing-related genes in TCGA-LIHC dataset.

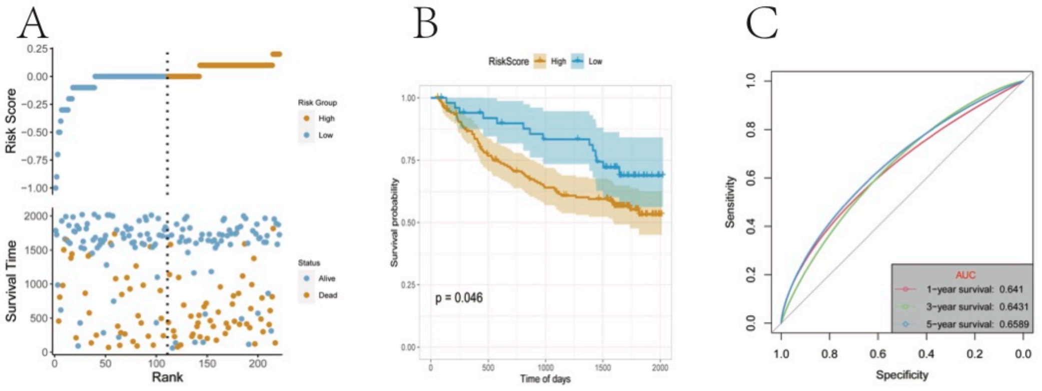

Supplementary Figure 4. Validation of the risk model in the external dataset GSE14520. (A) Distribution map of risk scores. (B) KM survival curve. (C) ROC curves for predicting 1-, 3-, and 5-year OS.

Supplementary table 1.

PID	logFC	AveExpr	t	P.Value	adj.P.Val	B	change
CETP	-3.19248292	6.52351021	-22.42135825	5.07E-74	1.55E-71	158.0044835	DOWN
ESR1	-3.896059982	6.279935668	-19.4586992	9.48E-61	1.45E-58	127.5596008	DOWN
NGFR	-3.108468442	6.881757351	-16.03745452	1.37E-45	1.39E-43	92.78359451	DOWN
SOCS2	-2.56827591	7.983300037	-14.36525281	2.04E-38	1.56E-36	76.30164628	DOWN
FGF23	-3.827654029	-0.898554927	-14.3280153	2.93E-38	1.79E-36	76.13558987	DOWN
PTGS2	-3.093288381	3.505841973	-13.52110606	6.81E-35	3.47E-33	68.49340805	DOWN
BUB1B	3.626630822	5.970255819	13.25425968	8.52E-34	3.72E-32	65.99016282	UP
FOS	-3.519035231	9.814305539	-12.86586555	3.24E-32	1.24E-30	62.13307807	DOWN
PDGFRA	-3.197355769	6.449715026	-12.82150752	4.90E-32	1.63E-30	61.76213106	DOWN
FOXO1	-1.778622514	8.985524292	-12.81220198	5.34E-32	1.63E-30	61.58601025	DOWN
RET	-3.512796475	3.27530017	-11.98868033	1.01E-28	2.80E-27	54.4357476	DOWN
TERT	5.088269358	3.182970727	11.94133812	1.54E-28	3.94E-27	54.03742719	UP
NRG1	-3.798164992	4.713625432	-11.92536766	1.78E-28	4.19E-27	53.76687197	DOWN
IGFBP3	-2.466638053	11.47685388	-11.79829728	5.57E-28	1.22E-26	52.47959601	DOWN
EGR1	-2.94395686	10.61715785	-11.70772107	1.25E-27	2.55E-26	51.65200927	DOWN
MSRA	-1.602893205	9.019211561	-11.50012181	7.89E-27	1.51E-25		

49.75103912 DOWN

MT1E -4.939356041 9.386458742 -11.42381213 1.55E-26 2.78E-25

49.1791628 DOWN

CDK1 3.578817149 7.34119085711.211299259.95E-26 1.69E-24

47.65477744 UP

GHR -2.975164102 9.588707848 -11.06519651 3.54E-25 5.70E-24

46.01807039 DOWN

CCNA2 3.345840719 6.968555951 10.85406638 2.18E-24 3.34E-23

44.61295044 UP

RAD51 2.201468103 5.415123777 10.82639246 2.77E-24 4.03E-23

44.38447871 UP

TP73 3.127046565 4.76959801 10.80487528 3.32E-24 4.62E-23

44.19802127 UP

FOXM1 3.624285281 7.433280262 10.65877645 1.15E-23 1.53E-22

42.96754502 UP

NCOR1 -1.121905236 10.09957181 -10.64853297 1.26E-23 1.60E-22

42.45168908 DOWN

E2F1 3.730226997 7.690019578 10.44229965 7.15E-23 8.76E-22

41.16614665 UP

BLM 2.350126947 5.328815363 10.27914132 2.79E-22 3.29E-21

39.83859682 UP

SHC1 0.879086935 11.4058958310.02304272 2.31E-21 2.61E-20

37.27530252 NOT

JUN -1.521821901 11.34577974-9.952465023 4.10E-21 4.48E-20

36.75512548 DOWN

PCNA 1.045045474 10.17313051 9.875426535 7.68E-21 8.10E-20

36.10292849 UP

HELLS 2.374132954 6.535749589 9.823669629 1.17E-20 1.19E-19

36.15054958 UP

LMNA 0.940734904 12.8401453 9.7918112291.51E-20 1.49E-19

35.45657853 NOT

IGF1 -2.191364585 6.463706943 -9.548917042 1.06E-19 1.01E-18

33.69370376 DOWN

FEN1 1.572747901 8.878907803 9.448498725 2.34E-19 2.17E-18 32.97944881 UP

TOP2A 3.925823688 8.689867195 9.396961713 3.51E-19 3.16E-18 32.76406138 UP

CAT -1.555490519 12.67631082 -9.258711168 1.04E-18 9.05E-18 31.35431531 DOWN

CDKN2A 3.852444407 7.096152736 9.1921659 1.74E-18 1.48E-17 31.23763931 UP

POLD1 1.295109963 8.5101119369.084194508 4.00E-18 3.31E-17 30.19182815 UP

MAPT 2.634255334 6.281060698 9.037150454 5.74E-18 4.62E-17 30.06598985 UP

BUB3 0.555464314 10.103769 8.909424277 1.52E-17 1.19E-16 28.55950821 NOT

RECQL4 2.409929321 8.040122856 8.905637951 1.57E-17 1.20E-16 28.99236065 UP

TCF3 1.037467097 9.245730744 8.869770181 2.06E-17 1.53E-16 28.43003644 UP

MAPK3 0.776677265 9.320537313 8.863767329 2.15E-17 1.57E-16 28.33463278 NOT

SERPINE1 -2.481451628 10.76738541 -8.828473443 2.81E-17 2.00E-16 28.02473273 DOWN

PARP1 0.716664397 11.341096568.728286712 5.97E-17 4.15E-16 27.22275245 NOT

NUDT1 1.623725623 7.258576041 8.606902965 1.48E-16 1.00E-15 26.79761139UP

NFE2L2 -0.856513921 10.6512088 -8.450661722 4.68E-16 3.11E-15 25.20871973 NOT

AGTR1 -1.827862717 9.198869799 -8.294885621 1.45E-15 9.47E-15 24.07146454 DOWN

CSNK1E 0.725677019 9.792532242 8.038103304 9.15E-15 5.83E-14 22.28676335 NOT

HMGB2 1.234814678 9.482834508 7.948631492 1.72E-14 1.07E-

13 21.78711947UP

BRCA2 1.3611287255.585425474 7.933747798 1.91E-14 1.16E-13 22.10408802 UP

BAX 0.91087313 9.875065192 7.932417301 1.93E-14 1.16E-13 21.56101539 NOT

CTF1 -1.713655256 3.509677096 -7.625488799 1.61E-13 9.47E-13 20.00239296 DOWN

STAT3 -0.704583134 11.69294788-7.472059372 4.55E-13 2.62E-12 18.4535059 NOT

PRKDC 0.766292122 10.77057481 7.460764704 4.90E-13 2.76E-12 18.31425949 NOT

PTK2 0.681032041 10.41904538 7.4591811474.96E-13 2.76E-12 18.30386647 NOT

NCOR2 0.586956588 10.58785689 7.433993601 5.87E-13 3.21E-12 18.13672101 NOT

SIRT7 0.972645203 8.532814802 7.334860182 1.14E-12 6.10E-12 17.79034709 NOT

KCNA3 -1.869046341 1.600342057 -7.315193876 1.29E-12 6.82E-12 17.99693068 DOWN

H2AFX 1.343065556 8.72074023 7.237018772 2.16E-12 1.12E-11 17.17194638 UP

GRN 0.598156435 12.63167701 7.215623619 2.49E-12 1.27E-11 16.78174924 NOT

SIRT1 -0.726492206 8.14140176 -7.199176955 2.77E-12 1.39E-11 16.73379815 NOT

HSF1 0.76443265 10.68850608 7.124862263 4.50E-12 2.22E-11 16.13520172 NOT

CDKN2B 1.452867142 6.870829666 7.092623193 5.54E-12 2.69E-11 16.48268498 UP

RAE1 0.587766427 8.824974573 6.970001876 1.22E-11 5.82E-11 15.34985045 NOT

NFKB2 0.737257522 10.18109202 6.82034644 3.14E-11 1.48E-10 14.2431669 NOT

GSK3A	0.455128328	9.80402716	6.796651097	3.64E-11	1.69E-10	14.10523838	NOT
PCK1	-3.665032261	12.59991987	-6.772166666	4.24E-11	1.94E-10	14.2110288	DOWN
APEX1	0.448359107	11.03314719	6.71662168	5.99E-11	2.70E-10	13.61315737	NOT
POLA1	0.827105348	7.308715447	6.700146992	6.64E-11	2.94E-10	13.96644785	NOT
ERCC3	0.405058052	9.106122998	6.649734271	9.06E-11	3.96E-10	13.30342758	NOT
PDGFB	1.038280803	7.982401634	6.473300526	2.65E-10	1.14E-09	12.54863228	UP
UBE2I	0.448974005	10.04153833	6.388447425	4.41E-10	1.87E-09	11.64611336	NOT
AKT1	-0.532829369	10.73644041	-6.369042239	4.95E-10	2.08E-09	11.56465176	NOT
CLU	-1.118996662	16.16139	-6.281338258	8.32E-10	3.44E-09	11.40031757	DOWN
AGPAT2	-1.116007628	11.16337654	-6.193478677	1.39E-09	5.68E-09	10.57939195	DOWN
JUND	-0.871696494	11.74615807	-6.162340791	1.67E-09	6.71E-09	10.41429399	NOT
XRCC6	0.407029949	12.00815717	6.153585132	1.75E-09	6.97E-09	10.34543983	NOT
UCP3	0.814260934	3.230127556	6.095907991	2.45E-09	9.60E-09	10.63880349	NOT
SPRTN	0.502089077	7.198745461	6.082813264	2.64E-09	1.02E-08	10.3635674	NOT
MXI1	-0.657905614	9.556260881	-6.081641657	2.66E-09	1.02E-08	9.88412221	NOT
MYC	-1.428414984	9.554014872	-6.024030576	3.69E-09	1.39E-08	9.587802023	DOWN
RGN	-1.430355155	11.19233873	-6.002040487	4.18E-09	1.54E-08		

9.513469683 DOWN

IL6 -1.87652653 2.260507955 -6.001724302 4.19E-09 1.54E-08

10.13220951 DOWN

PRKCA 0.759214482 9.458557325 5.987852487 4.53E-09 1.65E-08

9.470410938 NOT

FAS -0.972425729 7.709342092 -5.897898294 7.53E-09 2.71E-08

9.058060271 NOT

PDGFRB 0.976046967 9.98827479 5.885967728 8.04E-09 2.86E-08

8.864423349 NOT

HRAS 0.854728739 8.728153541 5.859300894 9.34E-09 3.28E-08

8.925549649 NOT

YWHAZ 0.601221578 12.46554996 5.815813737 1.19E-08 4.13E-

08 8.488997575 NOT

A2M -1.56870848 15.15245585 -5.810797736 1.22E-08 4.20E-08

8.747023993 DOWN

BAK1 0.927845489 8.008936808 5.800838836 1.29E-08 4.39E-08

8.741967836 NOT

HTT 0.462913401 10.36519792 5.751928811 1.69E-08 5.68E-08

8.08178643 NOT

MAP3K5 -1.128198598 7.732497363 -5.721661011 2.00E-08 6.64E-

08 8.070199616 DOWN

AR -1.967649395 9.977134844 -5.717578595 2.04E-08 6.71E-08

7.948092277 DOWN

PPARGC1A -1.803825469 9.29220883 -5.705810303 2.18E-08 7.08E-08

7.863890802 DOWN

SIRT6 0.6343801 8.193078972 5.662577787 2.75E-08 8.86E-08

7.940929771 NOT

PON1 -2.228708216 11.81502605 -5.640422303 3.10E-08 9.89E-08

7.63164095 DOWN

SUN1 0.490333074 10.29857935 5.5839504 4.20E-08 1.33E-07

7.196791919 NOT

CDK7 0.540082551 8.099525697 5.551533045 5.00E-08 1.56E-07

7.364545453 NOT

C1QA	-1.137383746	10.73928079	-5.542816757	5.24E-08	1.62E-07	7.035826329	DOWN
PCMT1	-0.428750395	9.708379609	-5.464887238	7.91E-08	2.40E-07	6.579729522	NOT
IGF2	-3.766483027	11.48997386	-5.464378925	7.93E-08	2.40E-07	6.79243606	DOWN
ERCC2	0.602668778	9.080127148	5.42898375	9.55E-08	2.87E-07	6.553618072	NOT
IL7R	-1.384701579	6.429991999	-5.41495699	1.03E-07	3.05E-07	6.709048284	DOWN
NGF	-1.062933258	3.51008306	-5.409134579	1.06E-07	3.12E-07	6.994491378	DOWN
LMNB1	1.130560054	8.435686492	5.385513446	1.20E-07	3.49E-07	6.527829256	UP
BRCA1	0.931421235	6.986185357	5.375696067	1.26E-07	3.64E-07	6.674317801	NOT
ADCY5	-1.135559331	6.155746807	-5.369613287	1.30E-07	3.72E-07	6.544874628	DOWN
PLCG2	-0.796280744	9.038176636	-5.355304517	1.40E-07	3.97E-07	6.034821976	NOT
PTK2B	-0.602328836	8.29822811	-5.326352244	1.63E-07	4.57E-07	6.003363795	NOT
PML	0.474113846	9.496350298	5.312880857	1.75E-07	4.81E-07	5.879715315	NOT
ATR	0.463470124	8.2221222	5.312832863	1.75E-07	4.81E-07	6.114888238	NOT
NFKBIA	-0.584004767	11.19456857	-5.306809062	1.80E-07	4.92E-07	5.838826999	NOT
LEPR	-1.722744728	10.69266951	-5.29662099	1.90E-07	5.14E-07	5.797419475	DOWN
SQSTM1	0.782596663	13.39447554	5.294748632	1.92E-07	5.14E-07	5.817408062	NOT
PLAU	0.999714163	6.967257428	5.260944651	2.28E-07	6.06E-07		

						6.114682753	NOT
HSP90AA1	0.439949373	13.7585993	5.241336115	2.52E-07	6.64E-07	5.58763464	NOT
GRB2	0.281303771	11.25340735	5.191840624	3.24E-07	8.46E-07	5.252720787	NOT
NFKB1	-0.435002397	9.29555112	-5.12527486	4.52E-07	1.17E-06	4.899699179	NOT
HDAC1	0.400781628	10.50664309	5.114020498	4.78E-07	1.23E-06	4.841367279	NOT
CHEK2	0.943776737	7.345169928	5.107955458	4.93E-07	1.26E-06	5.325499372	NOT
SIRT3	-0.436171714	9.087382392	-5.095663598	5.24E-07	1.32E-06	4.774134441	NOT
PTEN	-0.493958549	10.49045151	-5.094380543	5.27E-07	1.32E-06	4.774472877	NOT
PIK3R1	-0.786439734	10.63966664	-5.07732321	5.74E-07	1.43E-06	4.705890491	NOT
NR3C1	-0.472118159	10.21444883	-5.042529462	6.82E-07	1.68E-06	4.513266436	NOT
PRKCD	0.714030148	8.42298598	4.97622049	9.44E-07	2.31E-06	4.485695525	NOT
SOD1	-0.744280371	13.45762144	-4.938012081	1.14E-06	2.76E-06	4.180242466	NOT
ABL1	0.457348964	9.545571051	4.934071271	1.16E-06	2.79E-06	4.041667996	NOT
GCLC	-0.667622838	10.86858516	-4.920973424	1.23E-06	2.95E-06	3.97171923	NOT
FGFR1	-0.922653138	7.518724288	-4.894912034	1.40E-06	3.32E-06	4.02576429	NOT
APOC3	-1.877764316	15.53633273	-4.860297828	1.65E-06	3.89E-06	4.037485178	DOWN
EMD	0.430112093	9.966933812	4.839353255	1.83E-06	4.27E-06	3.556646976	NOT

MAPK8	-0.329140702	8.844358364	-4.781518698	2.40E-06	5.57E-06	3.345972026	NOT
PPP1CA	0.357719546	11.43182205	4.762014157	2.63E-06	6.02E-06	3.234801525	NOT
EEF1E1	0.643295992	7.043602885	4.76195394	2.64E-06	6.02E-06	3.722787157	NOT
TXN	0.65819407	12.28970151	4.729709257	3.07E-06	6.95E-06	3.108656504	NOT
ERCC8	0.365085125	6.426493664	4.693869713	3.62E-06	8.15E-06	3.464962093	NOT
NOG	-1.050209501	-0.756152629	-4.658486236	4.27E-06	9.54E-06	3.429012079	DOWN
DDIT3	0.767407547	9.015064295	4.654499705	4.35E-06	9.64E-06	2.915134427	NOT
DBN1	1.308272153	8.281742784	4.633753857	4.78E-06	1.05E-05	3.035685215	UP
RAD52	0.486492421	6.441405055	4.633187296	4.80E-06	1.05E-05	3.203527759	NOT
EGFR	-0.779826183	10.82704555	-4.556087257	6.82E-06	1.48E-05	2.329798186	NOT
MAPK9	0.335014873	9.289747745	4.466831793	1.02E-05	2.20E-05	1.973594328	NOT
UBB	-0.435478698	13.66612459	-4.459025324	1.06E-05	2.26E-05	2.037581076	NOT
TOP3B	0.820578172	2.30132899	4.293937066	2.18E-05	4.63E-05	1.886327633	NOT
ARHGAP1	0.290601808	10.448795	4.276828464	2.34E-05	4.95E-05	1.103130653	NOT
SNCG	1.559726053	6.791747394	4.253620272	2.59E-05	5.43E-05	1.639213232	UP
VCP	0.249249277	12.48997778	4.170689495	3.68E-05	7.67E-05	0.74443471	NOT
HTRA2	0.317983661	8.486372312	4.144358532	4.12E-05	8.51E-05		

0.795323286	NOT

HSPA1A	0.680888176	11.091794834.1421995114.15E-05	8.53E-05

0.571546448	NOT

TPP2	-0.343029737	9.299812126	-4.123702937	4.49E-05	9.15E-05

0.493478184	NOT

TNF	-0.969019768	2.138557386	-4.107628733	4.80E-05	9.72E-05

1.15450877	NOT

BMI1	0.351416784	9.559537138	4.094615047	5.06E-05

0.000101848	0.409911472NOT

GSTA4	0.8711305298.683145414	4.093278394	5.09E-05	0.000101848

0.646915681	NOT

ZMPSTE24	0.286430897	10.20683213	4.07549993	5.48E-05	0.00010892

0.291817408	NOT

HIC1	-0.592084775	6.417883791	-4.0672589	5.67E-05	0.000111965

0.751671071	NOT

IRS2	-0.731147992	10.5660212	-4.021739798	6.84E-05	0.000134127

0.122767896	NOT

EGF	1.769087861	1.17615209	3.964131201	8.64E-05	0.000168443

0.572520045	UP

PIN1	0.3565741	9.388166762	3.948898219	9.19E-05	0.000177986

-0.131784926	NOT

PDPK1	0.457650642	8.609885188	3.860600658	0.000130723

0.00025158	-0.303234227	NOT

SOD2	-0.656357708	13.71393428	-3.784916327	0.000175887

0.000336384	-0.615383718	NOT

GHRHR	1.663757199	2.05606002	3.7488998	0.000202219

0.000384343	-0.218811833	UP

TFAP2A	1.218279291	3.429218945	3.717101772	0.000228516

0.000431641	-0.322165921	UP

PTPN1	0.24420132	9.476145394	3.661802929	0.00028207	0.00052953	-
1.211950271NOT

UCP2	-0.585050534	8.789913429	-3.634307763	0.000312896

0.000583817	-1.303798466	NOT

CDKN1A	-0.681005051	11.23387646	-3.625461942	0.000323468	0.000599886	-1.32412341	NOT
PIK3CA	-0.29667613	8.058353443	-3.622444067	0.000327151	0.000603061	-1.173521792	NOT
TERF1	0.330769588	8.570897209	3.620177275	0.000329943	0.000604566	-1.1818927	NOT
MIF	0.680250342	9.618004585	3.600233389	0.000355493	0.000647504	-1.404888454	NOT
HOXB7	0.872605042	3.823109105	3.571975995	0.000394886	0.000715	-0.820958796	NOT
IGFBP2	-1.211752888	12.27753923	-3.56505185	0.000405145	0.00072926	-1.476200594	DOWN
JAK2	-0.431856801	6.70921225	-3.517808696	0.000482093	0.000858696	-1.291120196	NOT
CEBPB	-0.48780665	11.08378291	-3.517484656	0.000482666	0.000858696	-1.706766076	NOT
UCHL1	1.384071044	4.293512937	3.508977941	0.000497915	0.000880706	-1.038825287	UP
CEBPA	0.640280894	11.42403653	3.506745189	0.000501992	0.000882813	-1.762960496	NOT
PPARA	-0.502134373	10.61535402	-3.498837947	0.000516682	0.000903455	-1.785747351	NOT
APP	-0.55597478	12.76339624	-3.475370801	0.00056268	0.000975698	-1.787449422	NOT
TBP	0.274833373	7.377041434	3.474541157	0.000564375	0.000975698	-1.459622342	NOT
CTGF	-0.694841422	10.08491984	-3.447807598	0.000621589	0.001068574	-1.974366106	NOT
GSTP1	-0.660155702	8.767977413	-3.393553418	0.000754725	0.0012902	-2.132989403	NOT
STK11	0.264630534	9.99916054	3.345035747	0.000895841	0.001522929	-2.332469904	NOT
MLH1	0.215313993	8.935284822	3.312812201	0.001002734			

0.00169523 -2.313538251 NOT

DLL3 0.872325008 -0.692789161 3.296348277 0.001061814 0.001785247 -1.779815714 NOT

LRP2 -1.532686151 2.696118081-3.270025459 0.0011630220.001944726 -1.81397892 DOWN

HBP1 -0.383816849 9.260653798 -3.244265101 0.001270667 0.002113174-2.64757466 NOT

GSK3B 0.2016061189.584174189 3.224184768 0.0013609 0.002251002 -2.696316662 NOT

ELN -0.888610232 7.988940348 -3.207358468 0.00144104 0.002370743 -2.63490856 NOT

FOXO3 -0.302707295 9.364135411-3.057837645 0.002370439 0.003878901 -3.224119186 NOT

TP53BP1 0.477989743 7.4781111913.042164432 0.002494597 0.004060355 -2.833143076 NOT

FLT1 0.402320609 9.258869196 2.962297716 0.003225129 0.005221637 -3.416007443 NOT

HOXC4 0.792816121 1.544502389 2.94213319 0.003438197 0.005537307 -2.83574485 NOT

GCLM -0.409497719 10.06493215 -2.856746077 0.004490412 0.00719405 -3.808215276 NOT

ERCC4 -0.300609675 6.646049399 -2.85505627 0.004513913 0.00719405 -3.322937361 NOT

IL7 -0.379473173 5.930453868 -2.779566092 0.005685064 0.009013625 -3.435067312 NOT

HSPA1B 0.603868623 9.813400824 2.772217662 0.005812632 0.009168378 -4.010238859 NOT

CACNA1A 0.514993317 2.39552683 2.749924604 0.006215638 0.009753771 -3.364103936 NOT

HDAC2 0.238155609 9.538957016 2.743657464 0.006333393 0.009887848 -4.092639417 NOT

TRAP1 -0.305079907 10.542834 -2.715203505 0.006893759 0.010708072 -4.177468853 NOT

AIFM1	-0.30077173	11.19185544	-2.706259978	0.007078906	0.010940128	-4.176823693	NOT
EEF1A1	-0.255643098	15.57670007	-2.688118555	0.00746829	0.011483903	-3.951921925	NOT
SSTR3	0.87190369	0.272854945	2.637845607	0.008650026	0.01323454	-3.674603287	NOT
RB1	-0.350505873	8.438240029	-2.615554595	0.009225721	0.014045127	-4.329022052	NOT
RPA1	0.188715593	9.717580474	2.599516441	0.00966091	0.014634844	-4.493197543	NOT
PAPPA	-0.644774673	3.008082886	-2.575673602	0.010341848	0.01558919	-3.787787423	NOT
GSS	0.212477315	10.13339735	2.570046358	0.010508683	0.015763025	-4.58208255	NOT
IGF1R	-0.581505175	6.071832231	-2.554089686	0.010994903	0.016411904	-4.067502515	NOT
MED1	0.212288743	8.641751213	2.545365558	0.011269134	0.016739588	-4.458590064	NOT
PTPN11	-0.251655881	10.68876119	-2.536827977	0.011543386	0.017064135	-4.634028638	NOT
IFNB1	-0.286722392	-1.883063534	-2.46819131	0.013972999	0.020556431	-4.113473211	NOT
PYCR1	1.124461031	7.001274851	2.454118381	0.014523787	0.021264492	-4.271762038	UP
XPA	-0.201881453	7.869566107	-2.436298774	0.015248722	0.022219567	-4.624723483	NOT
ERCC1	0.234167587	9.563492655	2.311732178	0.021270446	0.030847188	-5.172587695	NOT
PMCH	0.388876169	-1.148267637	2.209700564	0.027659044	0.03992296	-4.705607731	NOT
PIK3CB	0.171037166	8.995714774	2.179061476	0.029876374	0.042920988	-5.387935722	NOT
HESX1	-0.308746114	3.560541434	-2.174310477	0.030233585			

0.043231201 -4.723275188 NOT

PPARG 0.389573348 8.3039110612.167977674 0.030715448 0.04371594

-5.232837021 NOT

EPS8 -0.231415557 9.630122001 -2.16348373 0.031061394

0.044003642 -5.532236492 NOT

HDAC3 0.145630443 9.6611466312.144557428 0.032555422

0.045907646 -5.558050398 NOT

MDM2 -0.216520757 10.52373949 -2.136333983 0.033223581

0.046634935 -5.567017363 NOT

CNR1 0.768670045 2.090196016 2.09797509 0.036498012

0.050997222 -4.918730697 NOT

HIF1A -0.277390983 10.08827332 -2.044572574 0.041513427

0.057741402 -5.775110953 NOT

EIF5A2 0.615255815 6.0751185222.039705731 0.041998328

0.058151531 -5.129329804 NOT

MTOR 0.21172725 10.1672167 2.026681201 0.043319729 0.059710978

-5.816425445 NOT

SP1 0.11825564910.16630646 2.013038594 0.044741471 0.061394126

-5.843638707 NOT

RICTOR -0.16750349 8.645817908 -2.004344035 0.045668012

0.062385767 -5.735670078 NOT

NBN 0.172935408 9.46118047 1.942292371 0.052762833 0.07172268

-5.943926409 NOT

S100B -0.503306662 2.992966008 -1.940575023 0.052971652

0.07172268 -5.199552306 NOT

EPOR -0.228628578 6.899709194 -1.906309686 0.057285135

0.077221371 -5.576389635 NOT

ERCC6 -0.15106977 6.761944507 -1.847946429 0.065305594

0.087646982 -5.654345197 NOT

PRDX1 0.197555135 12.65138747 1.8455258 0.065657372

0.087734304 -6.080689533 NOT

ATF2 0.139683388 9.452054822 1.840127244 0.066447564

0.088404151 -6.138999837 NOT

BCL2	-0.280651719	6.45821759	-1.790696839	0.074054454	0.098098108	-5.718894672	NOT
INS	-0.321202998	-1.789411216	-1.734155547	0.083616544	0.110287338	-5.632039582	NOT
INSR	-0.180237021	11.52902041	-1.722645716	0.085681012	0.112525278	-6.323323633	NOT
RELA	0.087305762	10.49868114	1.651105731	0.099456633	0.130058673	-6.491327899	NOT
TERF2	0.114703601	8.871192756	1.62357669	0.105208579	0.136995001	-6.420764591	NOT
HSPD1	0.135518224	13.30559104	1.619531228	0.106075737	0.137538879	-6.435179417	NOT
TAF1	0.189185187	8.428861833	1.613648542	0.107346847	0.138599727	-6.339920227	NOT
GTF2H2	0.340293711	4.824202649	1.590981357	0.112358253	0.144460611	-5.84348456	NOT
ATM	-0.149930695	8.790266565	-1.549651558	0.121970441	0.156162992	-6.564248604	NOT
SLC13A1	-0.324773843	-1.438320928	-1.536943687	0.125052192	0.159441545	-5.946959556	NOT
IL2	-0.274276776	-1.25244535	-1.523535236	0.128369539	0.162992029	-5.965351135	NOT
FOXO4	0.169683709	8.161029532	1.512499269	0.131151085	0.165835669	-6.438386458	NOT
PROP1	-0.155816899	-1.959286638	-1.510252736	0.131723013	0.165873424	-5.994106914	NOT
GPX1	0.190702361	12.49582088	1.498732998	0.134686264	0.16853306	-6.66212184	NOT
DGAT1	0.189541628	10.55864045	1.497768849	0.134936601	0.16853306	-6.73218166	NOT
POU1F1	-0.293482547	-0.378606655	-1.473737329	0.141293562	0.175755406	-6.029673781	NOT
SUMO1	-0.097534575	10.97800964	-1.456395321	0.146022679			

0.18090259 -6.76396845 NOT

CREBBP -0.112041958 10.14741547 -1.446757093 0.148703049 0.183480375 -6.814087469 NOT

TOP1 -0.102442659 10.14995269 -1.441383992 0.150213576 0.184599817 -6.822019291 NOT

SIN3A -0.099003628 9.518191395 -1.428574445 0.153862034 0.188327129 -6.832952989 NOT

STAT5B-0.106028555 10.03673224 -1.416102399 0.157478938 0.191986275 -6.860572973 NOT

BDNF 0.341797079 1.3911358561.406583464 0.160282668 0.193878984 -6.1181381 NOT

HMGB1 -0.093693915 11.28975723-1.40652961 0.160298637 0.193878984 -6.824542723 NOT

STUB1 0.1289981189.4111255851.402414465 0.161522472 0.194590065 -6.843358791 NOT

ERBB2 -0.271182351 10.52140005 -1.395765178 0.163514901 0.196217881 -6.86400676 NOT

CLOCK-0.126925565 8.64193801 -1.374647942 0.169965897 0.203162361 -6.790204571 NOT

APTX 0.107617571 8.700571086 1.37212945 0.170747862 0.203302901 -6.758530389 NOT

EFEMP1 -0.525947129 7.463023013 -1.320204813 0.187478141 0.222357795 -6.678220894 NOT

WRN -0.161975632 6.563280689 -1.278024046 0.201939428 0.238584807 -6.510085808 NOT

POLG 0.108686766 9.297361461 1.270014648 0.204775199 0.241004657 -7.00582951 NOT

ARNTL-0.184184027 7.708801784 -1.262208582 0.207566835 0.24335422 -6.760011709 NOT

IKBKB 0.1124122788.7524279111.2110258240.22655936 0.264607497 -6.977785252 NOT

MXD1 -0.13403158 7.582217844 -1.114361087 0.265754909 0.309205332 -6.887799185 NOT

CREB1	0.07373676	8.410678236	1.089793522	0.276422429	0.320398724	-7.056820704	NOT
GH1	-0.182811373	-1.319873576	-1.064255464	0.287818054	0.332348395	-6.556584875	NOT
ERCC5	-0.103171182	8.844366862	-1.056971199	0.291125932	0.334904268	-7.200693952	NOT
VEGFA	0.118633964	11.37016075	1.046403403	0.295970347	0.33920197	-7.267789956	NOT
EP300	-0.107654755	9.852906326	-1.039361066	0.299228544	0.340788754	-7.323270759	NOT
TP63	0.276656158	2.726628996	1.038599613	0.29958227	0.340788754	-6.552344052	NOT
SDHC	0.086076741	11.42487219	1.02782105	0.304619376	0.345235293	-7.284317466	NOT
CTNNB1	0.08462958	12.03163186	0.997157619	0.319256135	0.360488477	-7.297894784	NOT
NFE2L1	0.098198371	12.54513843	0.990737497	0.322378224	0.362675502	-7.288896669	NOT
XRCC5	-0.054603985	11.94075268	-0.947851499	0.343744386	0.385295905	-7.345412417	NOT
IRS1	-0.134610547	10.28537787	-0.916134007	0.360116819	0.402174258	-7.433511329	NOT
FGF21	-0.43367161	7.679066831	-0.863376079	0.388418323	0.432203661	-7.181791536	NOT
GPX4	0.093937374	12.85476331	0.842362518	0.400059457	0.44354418	-7.411622158	NOT
CISD2	-0.070682277	9.768902133	-0.835219699	0.404063899	0.446366618	-7.512939802	NOT
COQ7	-0.06514128	8.122494054	-0.828701154	0.407739286	0.448806552	-7.260908809	NOT
MAPK14	-0.059352508	10.29581568	-0.810597495	0.418051114	0.458507674	-7.526573437	NOT
APOE	-0.150438345	16.85944263	-0.784796674	0.433010589			

					0.472028643	-7.135162565	NOT
EEF2	-0.067486531	14.92677578	-0.7840225	0.433464211	0.472028643	-7.290444515	NOT
TGFB1	0.151332743	8.926920601	0.731496642	0.464879507	0.50444372	-7.473012273	NOT
MAX	-0.047019744	9.272616807	-0.700772332	0.483829082	0.52315088	-7.576869533	NOT
CDC42	0.040579281	11.35916362	0.693821661	0.488173588	0.525989852	-7.57243186	NOT
HSPA8	-0.077408315	13.68044161	-0.690305602	0.49037931	0.526512522	-7.465504011	NOT
UCP1	0.101203935	-1.606261347	0.639545473	0.522813161	0.559373522	-6.922049492	NOT
TP53	-0.094701022	9.093480537	-0.630929362	0.528426295	0.56340922	-7.604201552	NOT
PPM1D	0.048233852	7.716895466	0.565958789	0.571720879	0.607453434	-7.349633219	NOT
BSCL2	0.067071966	6.32715321	0.558161652	0.577028297	0.610971138	-7.106081222	NOT
TOP2B	0.033691063	10.43205315	0.516144662	0.606022152	0.639457857	-7.719261704	NOT
ATP5O	-0.05636876	10.56902862	-0.480545785	0.631086939	0.663617193	-7.726756961	NOT
PSEN1	0.029669228	9.921290312	0.473149152	0.636350004	0.666859936	-7.750458311	NOT
HSPA9	0.030718481	13.00439553	0.371566743	0.710400865	0.741920357	-7.687545547	NOT
TRPV1	-0.076476535	1.449502088	-0.347947842	0.728051848	0.75776825	-7.038895633	NOT
STAT5A	0.03303589	8.348478832	0.289578629	0.772280018	0.8010769	-7.593026262	NOT
LEP	-0.062366011	-0.884807055	-0.248086128	0.804187746	0.831356251	-7.084794842	NOT

GHRH	-0.035730322	-1.667618104	-0.20628217	0.836669416	0.862023034	-7.10408129	NOT
POLB	0.027120146	8.350573198	0.186126637	0.852434374	0.875318518	-7.615755959	NOT
KL	0.041187783	3.409886231	0.177576105	0.859140604	0.879254263	-7.050278547	NOT
GSR	-0.013304179	10.49559485	-0.115693142	0.907950456	0.926109465	-7.84143694	NOT
TFDP1	0.007789284	9.275268521	0.063639342	0.949287364	0.965056257	-7.818455096	NOT
PEX5	-0.004578909	10.10444922	-0.051043197	0.959315111	0.972021272	-7.860444956	NOT
IL2RG	0.0096405	7.774913474	0.038202463	0.969544219	0.979143666	-7.51840947	NOT
TERC	0.004241904	-1.627523666	0.030979906	0.975300126	0.981716575	-7.124712455	NOT
GDF11	0.003575951	6.070642094	0.020130686	0.983948581	0.987174642	-7.23643318	NOT
SST	0.002004774	-1.542474464	0.007656596	0.993894577	0.993894577	-7.124296253	NOT

Supplementary table 2.

Genes	bate	Hazard_Ratio	lower.95	upper.95	z_pvalue	Wald_pvalue	Likelihood_pvalue
CETP	-0.089916334	0.914007653	0.79703333	1.048149379	0.19812522	0.19812522	0.194438584
ESR1	-0.120908501	0.886115035	0.818664465	0.959122923	0.002760999	0.002760999	0.002761574
NGFR	-0.014148991	0.985950636	0.906491965	1.072374267	0.741368018	0.741368018	0.741661474
SOCS2	-0.243186088	0.784125584	0.699126811	0.879458377	3.26E-05	3.26E-05	3.66E-05
FGF23	0.009574882	1.009620868	0.888304273	1.14750579	0.883452049	0.883452049	0.884184892
PTGS2	0.041946287	1.042838463	0.948222202	1.146895799	0.387380939	0.387380939	0.388487756
BUB1B	0.24246812	1.274390621	1.144287722	1.419285922	1.02E-05	1.02E-05	5.57E-06
FOS	0.050764474	1.052075073	0.958072023	1.15530141	0.287766715	0.287766715	0.288197385
PDGFRA	0.026226321	1.026573257	0.961102993	1.096503351	0.435385293	0.435385293	0.433147791
FOXO1	-0.14878141	0.861757466	0.732022287	1.01448541	0.07390373	0.07390373	0.072560208
RET	0.081107995	1.084488009	1.009651709	1.164871244	0.026198316	0.026198316	0.028509351
TERT	0.01463891	1.014746584	0.957866004	1.075004881	0.618924416	0.618924416	0.620089526
NRG1	0.051619869	1.052975398	0.992596137	1.117027506	0.086655704	0.086655704	0.085493563
IGFBP3	0.189215461	1.208301267	1.076889699	1.355748832	0.001277657	0.001277657	0.001367549
EGR1	0.033281456	1.03384148	0.9328514	1.145764701	0.525693119	0.525693119	0.524622762

MSRA	-0.110667897	0.89523601	0.741378603	1.08102326	0.250050519	0.250050519	0.251731158
MT1E	-0.00663815	0.993383834	0.930794814	1.060181499	0.841542094	0.841542094	0.841548179
CDK1	0.276581995	1.318615068	1.174671268	1.48019769	2.74E-06	2.74E-06	1.63E-06
GHR	-0.142402204	0.867272366	0.786669662	0.956133678	0.004219259	0.004219259	0.004894055
CCNA2	0.229037205	1.25738882	1.136366237	1.391300263	9.18E-06	9.18E-06	1.29E-05
RAD51	0.27717937	1.31940301	1.161504286	1.498767009	2.03E-05	2.03E-05	1.65E-05
TP73	0.083213288	1.086773579	0.990346795	1.19258912	0.079200318	0.079200318	0.077963286
FOXM1	0.229942107	1.258527148	1.129948347	1.401737156	2.89E-05	2.89E-05	1.75E-05
NCOR1	0.01413812	1.014238536	0.840759182	1.223513022	0.88256506	0.88256506	0.882537308
E2F1	0.213635869	1.238171716	1.108256496	1.38331623	0.000158462	0.000158462	0.00012202
BLM	0.200173478	1.221614663	1.086817758	1.373130291	0.000791989	0.000791989	0.000708517
JUN	0.136141558	1.145844086	0.982565758	1.336255266	0.082612126	0.082612126	0.081095176
PCNA	0.399638072	1.491284861	1.228933565	1.809642605	5.16E-05	5.16E-05	5.39E-05
HELLS	0.197570441	1.21843889	1.089013706	1.36324577	0.000564244	0.000564244	0.000597519
IGF1	-0.096249829	0.908237084	0.831549598	0.991996872	0.032476661	0.032476661	0.033440905
FEN1	0.37676799	1.4575661	1.229892183	1.727386324	1.37E-05	1.37E-05	1.44E-05
TOP2A	0.20664072	1.229540743	1.11248621	1.358911621	5.16E-05	5.16E-	

05 3.69E-05
CAT -0.11708488 0.889509688 0.76566453 1.033386626 0.125860049
0.125860049 0.131967566
CDKN2A 0.159551545 1.172984722 1.061886408 1.295706535
0.001673846 0.001673846 0.001379002
POLD1 0.43948911 1.551914158 1.280588513 1.880727127 7.38E-06
7.38E-06 6.31E-06
MAPT 0.184084588 1.2021175031.076381746 1.342540876 0.00109171
0.00109171 0.001055453
RECQL4 0.28660584 1.331899128 1.170922609 1.515006435 1.30E-
05 1.30E-05 1.66E-05
TCF3 0.436052233 1.546589576 1.260676757 1.897345456 2.90E-
05 2.90E-05 3.18E-05
SERPINE1 0.144483004 1.155442057 1.05634236 1.263838694
0.001588463 0.001588463 0.001579207
NUDT1 0.31217801 1.366397904 1.179626826 1.582740568 3.14E-05
3.14E-05 5.97E-05
AGTR1 0.00866313 1.008700763 0.891282645 1.141587616
0.890872788 0.890872788 0.890660982
HMGB2 0.302665988 1.353462316 1.161560391 1.577068447
0.000104581 0.000104581 0.000129517
BRCA2 0.1761111721.192570631 1.0410417 1.366155372 0.011082293
0.0110822930.011342442
CTF1 0.046855808 1.047970889 0.946668704 1.160113333
0.366343165 0.366343165 0.368377746
KCNA3 -0.10104638 0.90389111 0.806774232 1.012698603 0.081442609
0.081442609 0.07876999
H2AFX 0.365376336 1.441056228 1.224378721 1.696079013 1.11E-
05 1.11E-05 9.79E-06
CDKN2B 0.284272272 1.328794675 1.144492358 1.542775953
0.000190297 0.000190297 0.000148623
PCK1 -0.076059115 0.92676142 0.872899492 0.983946877
0.012784805 0.012784805 0.015372707

PDGFB	0.081185605	1.08457218	0.912131816	1.289612744	0.358125299	0.358125299	0.355197905
CLU	-0.06290773	0.939030114	0.806404858	1.093467563	0.418072428	0.418072428	0.427361246
AGPAT2	0.170274069	1.185629751	1.011767247	1.389368861	0.035329983	0.035329983	0.033241159
MYC	0.026062389	1.026404983	0.923629418	1.140616755	0.628277691	0.628277691	0.62686218
RGN	-0.146092849	0.864077471	0.785467961	0.950554208	0.002682337	0.002682337	0.005504494
IL6	0.011214626	1.011277746	0.924543921	1.106148292	0.806359087	0.806359087	0.806725731
A2M	-0.007008084	0.993016416	0.897727178	1.098420128	0.89169764	0.89169764	0.891782806
MAP3K5	0.001143562	1.001144216	0.900843902	1.112612006	0.983061008	0.983061008	0.983059056
AR	-0.107088117	0.898446502	0.832638893	0.969455214	0.005793266	0.005793266	0.008185093
PPARGC1A	-0.112391403	0.893694394	0.834646496	0.956919694	0.001270305	0.001270305	0.002440352
PON1	-0.112774381	0.893352195	0.839934595	0.950167012	0.000337207	0.000337207	0.000904761
C1QA	0.063266159	1.065310343	0.933548299	1.215669429	0.347635834	0.347635834	0.347714763
IGF2	-0.022900527	0.9773597	0.9348728	1.02177749	0.312543902	0.312543902	0.309129852
IL7R	-0.062795105	0.939135878	0.864996487	1.019629803	0.134485812	0.134485812	0.134512833
NGF	0.056311403	1.057927074	0.923102858	1.212443103	0.418175254	0.418175254	0.416365078
LMNB1	0.295168147	1.343352221	1.175723578	1.534880496	1.42E-05	1.42E-05	9.78E-06
ADCY5	0.005903361	1.00592082	0.916289229	1.10432019	0.901333267		

0.901333267 0.901331453
LEPR -0.019703923 0.980488931 0.905062317 1.062201492
0.629485996 0.629485996 0.630967047
APOC3 -0.073418356 0.929212007 0.868642379 0.9940051
0.032777042 0.032777042 0.04526042
NOG 0.12059084 1.128163219 0.994001001 1.280433569
0.061927244 0.061927244 0.072426915
DBN1 0.184557751 1.202686435 1.079022492 1.340523179
0.00085663 0.00085663 0.001072071
SNCG 0.058376189 1.0601137240.983380698 1.142834214 0.12781063
0.12781063 0.138174453
EGF 0.079931029 1.083212355 1.023836777 1.146031313
0.005452999 0.005452999 0.006795497
GHRHR -0.009474371 0.99057037 0.93059273 1.054413629
0.766233685 0.766233685 0.765775076
TFAP2A 0.107745992 1.1137648041.033368474 1.200415989
0.004822826 0.004822826 0.005557719
IGFBP2 -0.024494545 0.975803012 0.896417 1.06221939 0.571550177
0.571550177 0.572347117
UCHL1 0.109661868 1.1159006851.053235709 1.182294076 0.00020009
0.00020009 0.000466401
LRP2 0.077405074 1.080479662 1.021445749 1.142925409
0.006930675 0.006930675 0.007998755
PYCR1 0.108055641 1.1141097341.040786779 1.192598258 0.00186526
0.00186526 0.001450096

Supplementary table 3.

Genes	logFC	AveExpr	t	P.Value	adj.P.Val	B	change	symbol
TICRR	3.140557485	0.132846887	22.24771123	4.23E-54	1.01E-49	112.1417868	UP	TICRR
MYBL2	3.971093623	2.777549815	21.36924071	9.03E-52	1.08E-47	107.2328185	UP	MYBL2
TAT	-6.521314145	7.454683807	-21.24973191	1.89E-51	1.50E-47	106.5881181	DOWN	TAT
PRR11	2.748891814	1.646080832	19.88838201	9.43E-48	5.64E-44	98.00137964	UP	PRR11
FANCI	2.031170382	2.895761346	19.77012755	2.00E-47	9.56E-44	97.3457448	UP	FANCI
GINS1	2.597374568	2.080049741	19.4614371	1.43E-46	5.44E-43	95.35008231	UP	GINS1
KIF18B	3.204689058	1.32323493	19.4444653	1.59E-46	5.44E-43	95.18010697	UP	KIF18B
SLC27A5	-4.604849377	7.372897575	-19.23184328	6.21E-46	1.75E-42	93.94984873	DOWN	SLC27A5
KIF23	2.958076833	1.532237543	19.22143262	6.63E-46	1.75E-42	93.78785716	UP	KIF23
FANCD2	2.153592327	2.108764869	19.20597506	7.33E-46	1.75E-42	93.73293387	UP	FANCD2
PLK1	3.025553499	2.311005177	19.15301516	1.03E-45	2.24E-42	93.40551802	UP	PLK1
CENPA	3.233760991	0.352416953	19.12270677	1.25E-45	2.49E-42	93.03160101	UP	CENPA
GTSE1	3.070408288	1.19253965	19.09902957	1.46E-45	2.68E-42	92.98032148	UP	GTSE1
CEP55	3.05013434	0.896003808	18.93712122	4.13E-45	7.06E-42	91.92001576	UP	CEP55
CHAF1B	2.589734051	1.675641634	18.89624706	5.38E-45	8.57E-42	91.73014958	UP	CHAF1B
ALDH2	-2.384185225	9.037925673	-18.87063056	6.34E-45	9.39E-42			

91.63467186 DOWN ALDH2

TROAP 3.037723681 1.897046572 18.86264781 6.68E-45 9.39E-42

91.53092778 UP TROAP

CES2 -3.372630472 8.245730929 -18.82345613 8.60E-45 1.13E-41

91.32916031 DOWN CES2

RAD51 2.326260194 0.845991557 18.81638196 9.00E-45 1.13E-41

91.13617535 UP RAD51

KIF11 2.224931775 2.294665132 18.72926927 1.58E-44 1.89E-41

90.70094012 UP KIF11

MKI67 2.752002405 3.81249396 18.66567101 2.39E-44 2.72E-41

90.32499203 UP MKI67

KIF2C 3.057190139 2.11587141318.39865419 1.35E-43 1.47E-40

88.56518185 UP KIF2C

ASF1B 2.413395843 2.138177015 18.37052787 1.62E-43 1.69E-40

88.38647871 UP ASF1B

CDCA2 2.8445611790.039323306 18.35771624 1.76E-43 1.69E-40

88.11109207UP CDCA2

TACC3 2.1103956994.093355888 18.35762484 1.76E-43 1.69E-40

88.33510742 UP TACC3

TPX2 2.508393138 3.902471948 18.32322321 2.21E-43 2.03E-40

88.11373057UP TPX2

MCM102.8463125110.741730014 18.27693437 2.98E-43 2.64E-40

87.67899841 UP MCM10

CCNB2 2.808182308 2.192746826 18.09728827 9.64E-43 8.23E-40

86.62205256 UP CCNB2

KIFC1 2.794866651 2.775338251 18.04206689 1.38E-42 1.14E-39

86.28094459 UP KIFC1

BUB1B 2.981620548 1.445563612 17.94756995 2.57E-42 2.05E-39

85.61353952 UP BUB1B

BIRC5 2.977486896 3.333797262 17.90546684 3.38E-42 2.61E-39

85.39921604 UP BIRC5

CDT1 2.582230061 2.493748974 17.86715724 4.35E-42 3.25E-39

85.13821892 UP CDT1

EHHADH -3.113574813 6.888134687 -17.85748055 4.64E-42 3.36E-39 85.06308862 DOWN EHHADH

HAGH -2.056504868 6.907348176 -17.84547745 5.02E-42 3.53E-39 84.98127431 DOWN HAGH

CENPO 1.902882054 2.12220211617.79842479 6.83E-42 4.67E-39 84.67918054 UP CENPO

WDR62 2.3630411831.86896616 17.77241043 8.11E-42 5.38E-39 84.49911438UP WDR62

TRIP13 2.75501737 1.390603481 17.70984793 1.22E-41 7.90E-39 84.06601274 UP TRIP13

GAS2L3 2.159604054 1.816446249 17.63007892 2.07E-41 1.30E-38 83.56904182 UP GAS2L3

GLYATL1 -4.764117569 5.003864008 -17.60698483 2.41E-41 1.48E-38 83.45048442 DOWN GLYATL1

PIF1 2.556413572 0.468376367 17.60226575 2.48E-41 1.48E-38 83.28681333 UP PIF1

HJURP 2.872719271 1.827099076 17.55594857 3.37E-41 1.93E-38 83.08675538 UP HJURP

CCNB1 2.514829693 3.430388843 17.55510573 3.39E-41 1.93E-38 83.11110938UP CCNB1

LDHD -3.071550567 5.697704895 -17.50402257 4.74E-41 2.64E-38 82.75749931 DOWN LDHD

PGRMC1 -1.883236835 8.117346632-17.48721876 5.30E-41 2.88E-38 82.6349527 DOWN PGRMC1

XRCC2 2.446466334 0.261336193 17.44525968 6.99E-41 3.72E-38 82.24221977 UP XRCC2

CDCA5 2.422280046 2.660559294 17.43261328 7.60E-41 3.95E-38 82.30433542 UP CDCA5

CDK1 2.616577943 2.824784966 17.311819361.69E-40 8.45E-38 81.51358409 UP CDK1

CDC6 2.541328974 2.682247599 17.311203241.70E-40 8.45E-38 81.50815833 UP CDC6

TOP2A 2.878929251 4.166858521 17.29381991 1.90E-40 9.28E-38

81.39032804 UP TOP2A

SPC25 2.448108994 0.67110679117.27996047 2.08E-40 9.97E-38

81.20622586 UP SPC25

NCAPH2.520897776 1.582588235 17.2031945 3.47E-40 1.63E-37

80.76738493 UP NCAPH

RAD54L 2.785887065 -0.00099799 17.16142162 4.57E-40 2.10E-37

80.36979355 UP RAD54L

SAPCD2 3.073971078 0.498046814 17.15108838 4.90E-40 2.21E-

37 80.35584856 UP SAPCD2

DLGAP5 2.8471119231.41146492417.115264896.21E-40 2.75E-37

80.18300646 UP DLGAP5

KIF4A 2.734614509 2.177579835 17.08072128 7.81E-40 3.39E-37

79.98421741 UP KIF4A

SLC10A1 -6.615976718 4.573657256 -17.07341087 8.20E-40 3.50E-

37 79.92052893 DOWN SLC10A1

PCK2 -2.790926676 7.842822289 -17.05610386 9.19E-40 3.86E-37

79.7919884 DOWN PCK2

BUB1 2.688851488 1.874771286 16.995745 1.37E-39 5.66E-37

79.41666139 UP BUB1

HRSP12 -2.600204661 7.415508964 -16.89931489 2.61E-39 1.06E-

36 78.75269965 DOWN HRSP12

CENPM2.820436251 1.575863908 16.89488509 2.68E-39 1.07E-36

78.74184605 UP CENPM

RBL1 1.671456605 1.916903717 16.88149099 2.93E-39 1.15E-36

78.663996 UP RBL1

ACSM2A -4.687225295 6.246052024 -16.86779156 3.21E-39 1.23E-

36 78.56828076 DOWN ACSM2A

NUF2 2.924192488 1.435847698 16.86686978 3.23E-39 1.23E-36

78.55146923 UP NUF2

ORC6 2.363163685 0.759049096 16.84954176 3.63E-39 1.36E-36

78.39276391 UP ORC6

EME1 2.318437225 0.287157594 16.83310332 4.05E-39 1.49E-36

78.24395912 UP EME1

PRC1	2.176814597	3.226966449	16.79350229	5.27E-39	1.89E-36	78.09619592	UP	PRC1
MTHFD1	-2.371919519	7.341478783	-16.79289841	5.29E-39	1.89E-36	78.04633001	DOWN	MTHFD1
HADH	-1.729092188	6.553716275	-16.77864149	5.82E-39	2.05E-36	77.95075624	DOWN	HADH
ACAT1	-2.017726103	7.784345255	-16.75624956	6.75E-39	2.34E-36	77.80371904	DOWN	ACAT1
KIAA1524	2.413330832	0.78406498	16.69257893	1.03E-38	3.53E-36	77.36243428	UP	KIAA1524
POLQ	2.547257001	0.372891916	16.68049247	1.12E-38	3.77E-36	77.25181688	UP	POLQ
FAM64A	3.472538547	-0.66222547	16.66509481	1.24E-38	4.12E-36	77.06452701	UP	FAM64A
CDC20	3.175342804	2.843856148	16.6498962	1.37E-38	4.50E-36	77.14702443	UP	CDC20
KIF18A	2.784309578	0.216056184	16.64739587	1.40E-38	4.51E-36	77.02331033	UP	KIF18A
GRHPR	-1.905128544	7.864486131	-16.64386961	1.43E-38	4.56E-36	77.05728111	DOWN	GRHPR
ABAT	-3.107373227	7.129914059	-16.59111401	2.03E-38	6.40E-36	76.70805665	DOWN	ABAT
DEPDC1B	3.562820067	0.920332969	16.54335171	2.80E-38	8.69E-36	76.39406615	UP	DEPDC1B
MLYCD	-1.759557813	4.371493307	-16.48058234	4.25E-38	1.30E-35	76.00345944	DOWN	MLYCD
UBE2C	3.088285976	2.620518681	16.44278926	5.48E-38	1.66E-35	75.77292386	UP	UBE2C
CDCA3	2.451408849	1.908871188	16.431572	5.91E-38	1.77E-35	75.69041546	UP	CDCA3
AR	-4.368808131	4.958659711	-16.39125809	7.73E-38	2.28E-35	75.42609443	DOWN	AR
ETFDH	-1.881776248	5.380933181	-16.3527184	1.00E-37	2.92E-35			

75.12902168 DOWN ETFDH

CCNA2 2.301083337 2.394224882 16.31938624 1.25E-37 3.61E-35 74.95247705 UP CCNA2

MTFR2 2.348609451 -0.268632023 16.30642069 1.37E-37 3.89E-35 74.72645289 UP MTFR2

BLM 2.4114615640.758279561 16.29220747 1.50E-37 4.23E-35 74.71739557 UP BLM

CENPF 2.690160122 3.409182452 16.21534231 2.51E-37 6.99E-35 74.2542443 UP CENPF

KIF20A 2.797085848 2.187919266 16.20275146 2.74E-37 7.52E-35 74.17495645 UP KIF20A

ZWINT 2.032857857 3.198430619 16.187811 3.02E-37 8.22E-35 74.07181922 UP ZWINT

ECT2 2.253560453 2.795293482 16.17924187 3.20E-37 8.61E-35 74.01952191 UP ECT2

KIF15 2.806464062 0.607010906 16.14685076 3.98E-37 1.06E-34 73.74816484 UP KIF15

SKA3 2.522187923 0.989747666 16.14141986 4.13E-37 1.09E-34 73.73223384 UP SKA3

CFHR4 -5.515958182 3.588359899 -16.09907296 5.49E-37 1.42E-34 73.4692828 DOWN CFHR4

NCAPG 2.690889657 2.095452995 16.0986622 5.50E-37 1.42E-34 73.48122296 UP NCAPG

ACSM5 -4.413465021 4.944837597 -16.07927459 6.27E-37 1.59E-34 73.34638348 DOWN ACSM5

TMEM192 -1.374333835 5.065255334 -16.06794902 6.76E-37 1.70E-34 73.22826678 DOWN TMEM192

EXO1 2.55465878 1.087704846 16.06460909 6.92E-37 1.72E-34 73.22694357 UP EXO1

HSD17B6 -4.455802395 7.382013598 -16.05547391 7.35E-37 1.81E-34 73.14083692 DOWN HSD17B6

GCDH -1.958023907 5.547626291 -16.05263012 7.50E-37 1.83E-34 73.12118089DOWN GCDH

TTK 2.836655764 1.083043335 16.05072443 7.59E-37 1.83E-34 73.13578091 UP TTK

PKM 2.675375807 6.584297371 16.02683183 8.92E-37 2.13E-34 72.94454325 UP PKM

RCC2 1.254386449 5.37085475 16.00747887 1.02E-36 2.40E-34 72.81491322 UP RCC2

MCM2 2.086600858 4.152924229 16.00031575 1.07E-36 2.50E-34 72.79830077 UP MCM2

E2F2 2.440536104 0.33117063315.97487877 1.26E-36 2.94E-34 72.58584974 UP E2F2

CDCA8 2.345712676 2.096437257 15.94616219 1.53E-36 3.53E-34 72.46487075 UP CDCA8

CYB5A -2.15950015 8.191146165-15.87011346 2.56E-36 5.83E-34 71.89106189 DOWN CYB5A

CDC45 2.44764006 1.587279143 15.86248601 2.69E-36 6.08E-34 71.89854554 UP CDC45

SGOL2 2.126079354 1.285365261 15.85400877 2.85E-36 6.37E-34 71.83186203 UP SGOL2

IVD -1.626683353 6.988431067 -15.82039558 3.58E-36 7.87E-34 71.55220753 DOWN IVD

PLK4 1.889127739 0.725544915 15.8188556 3.61E-36 7.87E-34 71.569162 UP PLK4

RP11_42O15.3 -3.682822774 0.952843728 -15.81860779 3.62E-36 7.87E-34 71.50180032 DOWN RP11-42O15.3

TRIM59 2.272419979 0.415751569 15.80692889 3.92E-36 8.44E-34 71.47435047 UP TRIM59

PON1 -4.590946105 6.916247986 -15.76598791 5.16E-36 1.10E-33 71.2067628 DOWN PON1

MELK 2.5113801851.71109262915.75319047 5.63E-36 1.19E-33 71.17183105 UP MELK

STIL 1.875354239 1.396869864 15.74887769 5.79E-36 1.22E-33 71.13374924 UP STIL

SERPING1 -2.083763347 10.73215635 -15.73647129 6.30E-36 1.31E-

33 71.01956527 DOWN SERPING1

AP000445.1 -4.276307804 -3.253819885 -15.71534428 7.26E-36 1.49E-33 70.52464014 DOWN AP000445.1

NDUFAF1 -1.196118693 4.3740412 -15.71481488 7.29E-36 1.49E-33 70.87863091 DOWN NDUFAF1

QDPR -1.797538707 6.44500587 -15.68793323 8.73E-36 1.77E-33 70.6642021 DOWN QDPR

AQP9 -5.363699708 6.713038636 -15.6830943 9.02E-36 1.81E-33 70.66560772 DOWN AQP9

LONP2 -1.439823952 6.612413402 -15.66913653 9.92E-36 1.98E-33 70.53616358 DOWN LONP2

C8A -4.050958367 6.7615855 -15.6619343 1.04E-35 2.06E-33 70.50361046 DOWN C8A

ERCC6L 2.538993601 -0.521898544 15.65980107 1.06E-35 2.07E-33 70.42733212 UP ERCC6L

ABCB4 -4.069398197 5.756352468 -15.6483886 1.14E-35 2.22E-33 70.43483003 DOWN ABCB4

ATAD5 1.692210293 0.971856145 15.64115073 1.20E-35 2.31E-33 70.39713564 UP ATAD5

NUSAP1 1.910959597 3.606855852 15.57792509 1.84E-35 3.51E-33 69.9805865 UP NUSAP1

RFC4 1.455804659 3.401316649 15.51409363 2.82E-35 5.33E-33 69.55583595 UP RFC4

MCM6 1.576350035 4.381271983 15.51371625 2.83E-35 5.33E-33 69.52352397 UP MCM6

ECHDC2 -1.895050033 7.040532454 -15.4832326 3.48E-35 6.50E-33 69.28621123 DOWN ECHDC2

CYP8B1 -6.788497356 5.727666202 -15.46707431 3.88E-35 7.20E-33 69.25847102 DOWN CYP8B1

FOXM1 2.582121178 2.867450928 15.45428694 4.23E-35 7.78E-33 69.16966209 UP FOXM1

CKAP2L 2.549981271 1.122015428 15.44919068 4.38E-35 8.00E-33 69.12576547 UP CKAP2L

DTL 2.264706095 2.298332881 15.42635916 5.11E-35 9.26E-33 68.98783954 UP DTL
E2F1 2.259737311 3.046405194 15.41135036 5.66E-35 1.02E-32 68.87713685 UP E2F1
SMIM14 -1.642265977 7.095095168 -15.38228006 6.89E-35 1.23E-32 68.60573155 DOWN SMIM14
UHRF1 2.683291248 1.38053994 15.37649145 7.16E-35 1.27E-32 68.64645135 UP UHRF1
ADH1C -5.079934668 7.191492704 -15.33539277 9.46E-35 1.66E-32 68.31317478 DOWN ADH1C
LINC01430 -3.466694326 -3.539769614 -15.31253448 1.10E-34 1.93E-32 67.84347728 DOWN LINC01430
ORC1 2.228432644 0.982570414 15.28157202 1.36E-34 2.36E-32 67.99832391 UP ORC1
ANLN 2.750950334 2.371313595 15.26805111 1.49E-34 2.57E-32 67.92485091 UP ANLN
SKA1 2.888787484 1.053841816 15.26248222 1.55E-34 2.65E-32 67.875288 UP SKA1
CHEK1 1.745633432 2.422136031 15.2439327 1.76E-34 2.98E-32 67.76195466 UP CHEK1
ARHGAP11A 2.110756573 2.132874471 15.22469451 2.00E-34 3.37E-32 67.63479725 UP ARHGAP11A
ADH1B -5.041955127 8.922734118 -15.2138908 2.15E-34 3.60E-32 67.48646152 DOWN ADH1B
C6 -4.197645202 6.871406844 -15.20132443 2.34E-34 3.89E-32 67.40236031 DOWN C6
2-Mar -1.931591758 6.210854225 -15.19592416 2.43E-34 4.01E-32 67.35304412 DOWN 2-Mar
ETNPPL -5.128408194 4.24141401 -15.18291394 2.66E-34 4.35E-32 67.35391466 DOWN ETNPPL
MMAA -1.369373808 3.960242371 -15.17210664 2.86E-34 4.65E-32 67.24552297 DOWN MMAA
NDC80 2.306494749 1.987669728 15.16584516 2.98E-34 4.82E-32

67.2397115 UP NDC80
AURKB 2.734990378 1.939606198 15.16264098 3.05E-34 4.89E-
32 67.21816114UP AURKB
CDCA4 1.514984591 2.28696609 15.15337999 3.24E-34 5.17E-32
67.15474515 UP CDCA4
LMNB2 1.479354794 4.368483597 15.13228098 3.74E-34 5.93E-32
66.95372982 UP LMNB2
KIAA1841 1.380964938 1.63150811515.13096966 3.78E-34 5.94E-32
67.00253877 UP KIAA1841
NCAPD2 1.537330873 4.418282359 15.10569829 4.48E-34 7.00E-
32 66.77329547 UP NCAPD2
MCM4 1.6161187164.987150624 15.09298802 4.88E-34 7.58E-32
66.67262029 UP MCM4
SPATS2 1.277870542 3.737421977 15.09079332 4.96E-34 7.65E-32
66.69453099 UP SPATS2
ACYP2 -1.372894964 3.466421113-15.08392077 5.19E-34 7.96E-32
66.66984199 DOWN ACYP2
ALDH6A1 -2.648707692 6.866465324 -15.06611003 5.86E-34 8.93E-
32 66.47627862 DOWN ALDH6A1
C11orf84 1.641712075 2.638264373 15.05343332 6.39E-34 9.62E-
32 66.4770253 UP C11orf84
GFOD2 -1.200171448 4.632303773 -15.05314919 6.40E-34 9.62E-32
66.41537427 DOWN GFOD2
SEC14L2 -3.413462182 6.17520848 -15.04834176 6.61E-34 9.88E-32
66.37193953 DOWN SEC14L2
SCP2 -2.055193209 8.025084035 -14.99878602 9.25E-34 1.37E-31
66.0220399 DOWN SCP2
H2AFX 1.663192544 4.237973531 14.98928719 9.87E-34 1.46E-31
65.99414137 UP H2AFX
CENPI 2.300867077 0.046977077 14.95404065 1.25E-33 1.84E-31
65.76219541 UP CENPI
ECM2 -2.632400812 4.029779416 -14.95044533 1.28E-33 1.87E-31
65.76783795 DOWN ECM2

SHMT1	-2.513898512	7.27979753	-14.94115269	1.37E-33	1.98E-31	65.63039621	DOWN	SHMT1
GLYAT	-6.81776936	3.579865641	-14.94056185	1.37E-33	1.98E-31	65.71051171	DOWN	GLYAT
KIAA0101	2.158997718	2.286696027	14.91855909	1.59E-33	2.28E-31	65.57445695	UP	KIAA0101
CRYL1	-2.042599317	6.66476851	-14.9014049	1.79E-33	2.55E-31	65.36075986	DOWN	CRYL1
CCNF	1.780719529	2.379879386	14.89924587	1.82E-33	2.57E-31	65.44308667	UP	CCNF
SGOL1	2.354536997	0.472710123	14.89705822	1.85E-33	2.60E-31	65.40302135	UP	SGOL1
WDHD1	1.882370765	1.415785493	14.8788209	2.09E-33	2.92E-31	65.30649373	UP	WDHD1
PEX11G	-2.112974374	2.574826155	-14.87440402	2.15E-33	2.99E-31	65.28000344	DOWN	PEX11G
HPD	-5.861813678	8.099995172	-14.84222401	2.68E-33	3.70E-31	64.98193856	DOWN	HPD
CYP2C8	-4.794120611	6.561868425	-14.83528982	2.81E-33	3.86E-31	64.94621393	DOWN	CYP2C8
SUCLG2	-1.453992214	6.752623101	-14.82957002	2.92E-33	3.99E-31	64.87265687	DOWN	SUCLG2
RP11_863K10.7	-3.135792271	-1.689548661	-14.82085814	3.10E-33	4.21E-31	64.6676629	DOWN	RP11-863K10.7
FTCDNL1	-1.83185155	1.72871849	-14.81329496	3.26E-33	4.40E-31	64.85835789	DOWN	FTCDNL1
NR1I2	-4.880317345	3.135887199	-14.78188563	4.03E-33	5.42E-31	64.65405991	DOWN	NR1I2
CAT	-1.931369325	7.947668576	-14.75246589	4.93E-33	6.58E-31	64.35537554	DOWN	CAT
NEK2	2.684544537	1.720814014	14.73337377	5.61E-33	7.45E-31	64.33051823	UP	NEK2
CPT2	-1.638671794	5.575162405	-14.73241662	5.65E-33	7.46E-31			

64.22663188 DOWN CPT2
HSD17B4 -1.800931027 7.892147651 -14.71657805 6.29E-33 8.26E-31 64.11197245 DOWN HSD17B4
CENPE 2.349393978 1.398421592 14.7080447 6.66E-33 8.71E-31 64.15827084 UP CENPE
ARHGAP11B 2.021872223 0.07971631 14.6881959 7.63E-33 9.91E-31 63.98092191 UP ARHGAP11B
AKR7A3 -4.220206729 4.784121966 -14.68370273 7.86E-33 1.02E-30 63.96801358 DOWN AKR7A3
EZH2 1.682635144 2.931605174 14.67182761 8.53E-33 1.10E-30 63.89580994 UP EZH2
PON3 -2.777052352 6.275915122 -14.66833447 8.73E-33 1.12E-30 63.79220647 DOWN PON3
ADI1 -1.978606179 8.263743662 -14.66307272 9.05E-33 1.15E-30 63.75258504 DOWN ADI1
RRM2 2.272257377 3.774951694 14.63702337 1.08E-32 1.37E-30 63.63505965 UP RRM2
FDX1 -1.353487237 5.271387189 -14.63537621 1.09E-32 1.37E-30 63.57332589 DOWN FDX1
DHODH -2.17947289 5.176226242 -14.62055278 1.21E-32 1.51E-30 63.48552113 DOWN DHODH
HELLS 2.175064033 1.92814951 14.61997723 1.21E-32 1.51E-30 63.56575185 UP HELLS
PCK1 -5.25920259 7.418235347 -14.61143979 1.29E-32 1.59E-30 63.4208682 DOWN PCK1
HAO1 -4.170727921 6.310868992 -14.57095665 1.69E-32 2.09E-30 63.15178164 DOWN HAO1
C17orf53 1.887685182 0.846586936 14.55298141 1.91E-32 2.35E-30 63.10361315 UP C17orf53
C5orf34 1.563750807 0.08628664 14.42161218 4.68E-32 5.71E-30 62.18931751 UP C5orf34
DEPDC7 -2.771854192 3.841866122 -14.39764364 5.51E-32 6.69E-30 62.04116562 DOWN DEPDC7

GOT2 -1.663580799 7.731818854 -14.39431876 5.63E-32 6.80E-30 61.9270526 DOWN GOT2

PTTG1 2.623558803 2.873606365 14.39164201 5.74E-32 6.89E-30 62.00698197 UP PTTG1

PHF19 1.616572499 3.064412883 14.38356456 6.06E-32 7.25E-30 61.94242194 UP PHF19

RAD51D 1.015962381 2.837322938 14.37556936 6.40E-32 7.62E-30 61.89625694 UP RAD51D

POR -1.72473839 8.804493729 -14.359589 7.14E-32 8.45E-30 61.70141495 DOWN POR

TYRO3 3.1996458 2.040426247 14.31665483 9.56E-32 1.13E-29 61.51793229 UP TYRO3

HIBCH -1.319796162 5.404662797 -14.30771807 1.02E-31 1.19E-29 61.34928216 DOWN HIBCH

KNTC1 1.780697228 2.84939666 14.28749683 1.17E-31 1.36E-29 61.30029649 UP KNTC1

SHCBP1 2.609946336 0.796122034 14.28485655 1.19E-31 1.38E-29 61.30021204 UP SHCBP1

DMGDH -3.425153442 5.443093447 -14.28187948 1.21E-31 1.40E-29 61.20604337 DOWN DMGDH

PKMYT1 2.035413851 1.664895341 14.25539287 1.45E-31 1.67E-29 61.10769888 UP PKMYT1

BARD1 1.777859961 1.249969951 14.23316206 1.69E-31 1.93E-29 60.95691438 UP BARD1

RP11_505K9.4 -2.391710348 -1.514829795 -14.23114475 1.71E-31 1.95E-29 60.75352264 DOWN RP11-505K9.4

LMNB1 1.933274372 3.700027759 14.22978459 1.73E-31 1.96E-29 60.8778532 UP LMNB1

BDH1 -3.016473497 6.491281817 -14.22766169 1.75E-31 1.98E-29 60.80412985 DOWN BDH1

PARPBP 1.957064747 0.888943289 14.21878191 1.86E-31 2.09E-29 60.85532633 UP PARPBP

KLF9 -2.052355166 6.063720356 -14.19971357 2.12E-31 2.37E-29

60.61147525DOWN KLF9

SAR1B -1.505279262 6.711008665-14.18521511 2.34E-31 2.60E-29

60.50531492 DOWN SAR1B

CTC_297N7.9 -2.753898017 0.131238385 -14.15419164 2.89E-31

3.20E-29 60.35061189DOWN CTC-297N7.9

ZNF239 3.12769935 -0.536451319 14.14903808 3.00E-31 3.30E-29

60.34140556 UP ZNF239

METTL7A -1.884307023 8.513583461 -14.14342237 3.11E-31 3.41E-29 60.23143385 DOWN METTL7A

PCCB -1.492291958 6.522043775 -14.14163386 3.15E-31 3.44E-29

60.2099635 DOWN PCCB

CENPK 2.20498683 0.370295499 14.13822416 3.22E-31 3.51E-29

60.30035737 UP CENPK

C1S-2.185285032 10.37433533 -14.10313717 4.10E-31 4.42E-29

59.98214875 DOWN C1S

ACSM2B -4.103508202 6.8099637 -14.10286064 4.10E-31 4.42E-29

59.96555376 DOWN ACSM2B

CENPL 1.652442863 1.477779581 14.09035933 4.47E-31 4.79E-29

59.99436199 UP CENPL

ALDH5A1 -1.920616321 6.378318007 -14.07721997 4.89E-31 5.22E-29 59.77548534 DOWN ALDH5A1

HAAO -2.408667683 6.401381271 -14.04611098 6.04E-31 6.42E-29

59.56730187 DOWN HAAO

RTKN2 3.167253936 -0.762601242 14.02905761 6.79E-31 7.18E-29

59.5251911 UP RTKN2

ANXA10 -4.233901287 2.592052267 -14.01786091 7.33E-31 7.72E-29 59.50425131 DOWN ANXA10

FMO4 -2.392327031 3.942705125 -14.00154906 8.19E-31 8.59E-29

59.34903197 DOWN FMO4

CTD_3076M17.1 -3.710956407 -1.347970308 -13.99997728 8.28E-31

8.64E-29 59.22036737 DOWN CTD-3076M17.1

RHNO1 1.1571134323.22366701113.99910566 8.33E-31 8.66E-29

59.3311088 UP RHNO1

FANCG 1.316776561 2.9183608 13.98673356 9.06E-31 9.35E-29 59.26077949 UP FANCG
TYMS 1.901552763 3.524821068 13.98659468 9.07E-31 9.35E-29 59.23640439 UP TYMS
SEPSECS -1.378355287 4.749311186-13.98209428 9.35E-31 9.58E-29 59.15971041 DOWN SEPSECS
GADD45A -1.817081325 5.498329142 -13.98174174 9.37E-31 9.58E-29 59.14006 DOWN GADD45A
CCNE1 2.401250752 1.00423014 13.9735942 9.91E-31 1.01E-28 59.20484923 UP CCNE1
AZGP1P2 -3.385401436 -3.272326323 -13.9593864 1.09E-30 1.11E-28 58.87735697 DOWN AZGP1P2
TMBIM6 -1.057564382 10.2213064 -13.94972365 1.17E-30 1.18E-28 58.94117144DOWN TMBIM6
DHTKD1 -1.895691455 6.660651393 -13.94473954 1.21E-30 1.21E-28 58.87401694 DOWN DHTKD1
RNASE4 -2.475215481 3.674208971 -13.94244977 1.23E-30 1.23E-28 58.95997461 DOWN RNASE4
INSIG1 -2.83059407 8.011198293-13.93395326 1.30E-30 1.29E-28 58.80574974 DOWN INSIG1
FANCB 2.050473797 -2.037059222 13.92065756 1.42E-30 1.41E-28 58.71350479 UP FANCB
GSG2 2.329995233 -1.291035583 13.91818263 1.45E-30 1.43E-28 58.74325875 UP GSG2
MAD2L1 1.850489548 2.305983567 13.91632092 1.46E-30 1.44E-28 58.8052359 UP MAD2L1
STARD5 -2.348982117 1.775174485 -13.86915665 2.02E-30 1.98E-28 58.49914716 DOWN STARD5
RAD51AP1 1.957439361 1.059078498 13.86856139 2.03E-30 1.98E-28 58.49615145 UP RAD51AP1
UBE2T 1.90615914 2.375322382 13.85875873 2.17E-30 2.11E-28 58.41362589 UP UBE2T
RACGAP1 1.718817726 3.283719449 13.82996908 2.64E-30 2.56E-

28 58.18378729 UP RACGAP1

SLC27A2 -3.287324087 6.245932126 -13.7947777 3.36E-30 3.24E-28 57.87275854 DOWN SLC27A2

POLD1 1.209919019 4.026183487 13.79295878 3.40E-30 3.26E-28 57.89537629 UP POLD1

RMI2 1.945616426 1.755392069 13.79104922 3.44E-30 3.29E-28 57.96968819 UP RMI2

DCXR -2.969164445 8.323197485 -13.78263456 3.65E-30 3.47E-28 57.78025852 DOWN DCXR

HPR -4.651646922 6.800695295 -13.7463664 4.67E-30 4.42E-28 57.55328259 DOWN HPR

MYO1B -1.568220107 7.449290632 -13.7454717 4.70E-30 4.42E-28 57.52006145 DOWN MYO1B

TMEM237 1.385344764 2.480812085 13.74541864 4.70E-30 4.42E-28 57.64324592 UP TMEM237

ECHS1 -1.638023403 8.752547822 -13.72721938 5.32E-30 4.99E-28 57.4079294 DOWN ECHS1

EPHX2 -2.289024657 6.162249206 -13.70800608 6.07E-30 5.67E-28 57.27285176 DOWN EPHX2

TCF3 1.045671904 4.863513345 13.68439682 7.13E-30 6.63E-28 57.12553094 UP TCF3

METTL20 -1.317286479 2.150831045 -13.68351402 7.17E-30 6.65E-28 57.24478778 DOWN METTL20

CLYBL -1.811116461 4.683673604 -13.67542526 7.58E-30 7.00E-28 57.08727537 DOWN CLYBL

ARHGEF2 1.592612459 4.398865199 13.67057217 7.83E-30 7.20E-28 57.05181409 UP ARHGEF2

GPT2 -2.667675202 6.827724592 -13.65853286 8.50E-30 7.77E-28 56.93309278 DOWN GPT2

SPDL1 1.353226731 2.205707051 13.65833898 8.51E-30 7.77E-28 57.06279967 UP SPDL1

PFKFB1 -3.710473604 2.609174386 -13.6394241 9.69E-30 8.81E-28 56.94911885DOWN PFKFB1

ABCA8	-3.736379614	3.336054378	-13.63645925	9.88E-30	8.95E-28	56.91902557	DOWN	ABCA8
HMGA1	1.845771741	5.755800047	13.62753942	1.05E-29	9.48E-28	56.7270057	UP	HMGA1
TMEM220_AS1	-2.610716517	1.552081725	-13.62473679	1.07E-29	9.63E-28	56.84718017	DOWN	TMEM220-AS1
ST3GAL6	-2.243633239	4.057296302	-13.61880522	1.11E-29	9.99E-28	56.74453575	DOWN	ST3GAL6
CENPH	1.504855618	1.546453979	13.61202173	1.17E-29	1.04E-27	56.76291135	UP	CENPH
MIP	-2.637546073	-2.542698689	-13.60960812	1.19E-29	1.06E-27	56.56085029	DOWN	MIP
CTH	-3.681947967	4.707857274	-13.60754982	1.20E-29	1.07E-27	56.67297309	DOWN	CTH
F8	-1.938844058	3.171083439	-13.5858614	1.40E-29	1.23E-27	56.55912849	DOWN	F8
ECI2	-1.467419121	7.254220687	-13.58307255	1.42E-29	1.25E-27	56.41556808	DOWN	ECI2
DNAJC25	-1.348240911	4.029037814	-13.56890504	1.57E-29	1.37E-27	56.38841447	DOWN	DNAJC25
HIBADH	-1.603110502	6.349966354	-13.55506671	1.72E-29	1.50E-27	56.22679454	DOWN	HIBADH
FBP1	-3.19883463	6.829787306	-13.54844408	1.80E-29	1.57E-27	56.18943819	DOWN	FBP1
CYP4F2	-4.647151484	4.951176166	-13.54775377	1.81E-29	1.57E-27	56.27810851	DOWN	CYP4F2
HMGCL	-1.711101703	6.92076783	-13.54283312	1.87E-29	1.62E-27	56.1420022	DOWN	HMGCL
ALAD	-1.72368745	7.203788785	-13.5342401	1.99E-29	1.71E-27	56.08399937	DOWN	ALAD
NRM	1.798675202	3.11176113	13.51984379	2.19E-29	1.87E-27	56.08890989	UP	NRM
RP11_158L12.4	2.287779333	-1.998335064	13.51897905	2.20E-29				

1.88E-27 56.03306751 UP RP11-158L12.4
ARHGEF39 1.740121487 1.750317323 13.51459644 2.27E-29 1.93E-27 56.10071041 UP ARHGEF39
DHRS1 -1.864966566 5.448975333 -13.49524233 2.59E-29 2.19E-27 55.83685373 DOWN DHRS1
PLXNA1 1.630576312 3.955306739 13.48434433 2.79E-29 2.35E-27 55.80718738 UP PLXNA1
FGGY -2.689304504 5.284794019 -13.48287601 2.82E-29 2.37E-27 55.77361609 DOWN FGGY
ASPDH -4.71390008 3.84817022 -13.47632024 2.95E-29 2.47E-27 55.83347799 DOWN ASPDH
MCM8 1.467824564 2.502952713 13.47233328 3.03E-29 2.52E-27 55.79266736 UP MCM8
CLEC3B -2.431347849 2.529248444 -13.46337928 3.22E-29 2.67E-27 55.75478337 DOWN CLEC3B
ESR1 -4.017753318 1.744210113-13.43491893 3.91E-29 3.24E-27 55.56461304 DOWN ESR1
MAPRE1 1.0491161165.343831485 13.42723969 4.12E-29 3.40E-27 55.36715728 UP MAPRE1
AP006216.5 -3.473869608 -2.518277007 -13.42221689 4.27E-29 3.51E-27 55.32084772 DOWN AP006216.5
CDC25A 2.365719232 1.02114217413.41736436 4.41E-29 3.61E-27 55.45055674 UP CDC25A
STMN1 1.566444856 5.586305699 13.39624732 5.09E-29 4.16E-27 55.15565033 UP STMN1
MASP2 -4.326650032 6.227744682 -13.38531371 5.49E-29 4.46E-27 55.11021966DOWN MASP2
RGN -2.844940626 6.382024153 -13.36210974 6.43E-29 5.21E-27 54.92494809 DOWN RGN
APOC4 -3.458745731 -1.999679668 -13.35370082 6.81E-29 5.50E-27 54.87914869 DOWN APOC4
CRHR1_IT12.303737647 0.922335213 13.35135184 6.92E-29 5.57E-27 55.00501223 UP CRHR1-IT1

CYP4V2 -1.808060738 6.042748233 -13.33879398 7.54E-29 6.05E-27 54.76170741 DOWN CYP4V2
RP11_1259L22.2 -4.697379233 -2.731843595 -13.3354478 7.71E-29 6.17E-27 54.75003598 DOWN RP11-1259L22.2
PAFAH1B3 2.275589297 3.310978659 13.33381734 7.80E-29 6.22E-27 54.82192241 UP PAFAH1B3
RIBC2 3.052465746 -1.167226228 13.32829777 8.10E-29 6.43E-27 54.80184594 UP RIBC2
RP11_443B20.1 2.065763613 -1.714988282 13.29950969 9.85E-29 7.80E-27 54.57297459 UP RP11-443B20.1
PSMC3IP 1.521762628 0.35768736 13.28877679 1.06E-28 8.37E-27 54.57632587 UP PSMC3IP
IL27 -3.001408411 1.502224799 -13.28382208 1.10E-28 8.63E-27 54.54667017 DOWN IL27
ACBD4 -1.858881924 5.229327818 -13.27403441 1.17E-28 9.20E-27 54.34070724 DOWN ACBD4
CDC25C 2.442264912 0.842680069 13.26735426 1.23E-28 9.56E-27 54.43811 UP CDC25C
AKR1C6P -4.602907449 0.357544628 -13.26266367 1.27E-28 9.84E-27 54.35916415 DOWN AKR1C6P
LYRM5 -1.247029753 4.383414163 -13.23943965 1.48E-28 1.15E-26 54.13088455 DOWN LYRM5
TAPT1 -1.097807127 4.793947372 -13.23507318 1.53E-28 1.18E-26 54.08116114DOWN TAPT1
TM6SF2 -3.321998109 2.915574164 -13.20014003 1.94E-28 1.49E-26 53.97235658 DOWN TM6SF2
DEPDC1 2.554751391 0.969135875 13.19762377 1.98E-28 1.51E-26 53.96723583 UP DEPDC1
C18orf54 1.8756112130.009660258 13.19726848 1.98E-28 1.51E-26 53.95402635 UP C18orf54
MCM3 1.36158025 5.67199177 13.19443803 2.02E-28 1.54E-26 53.78218768 UP MCM3
BTNL9 -2.568204003 2.354588505 -13.15473216 2.65E-28 2.01E-26

53.67254934 DOWN BTNL9

CTD_2510F5.4 2.797706165 -2.081148304 13.15176877 2.70E-28 2.04E-26 53.58028023 UP CTD-2510F5.4

CLSPN 2.466476733 -0.348549248 13.1493366 2.75E-28 2.07E-26 53.62444625 UP CLSPN

CDCA7 3.875834401 -0.034041659 13.1479281 2.77E-28 2.08E-26 53.6271368 UP CDCA7

RTP3 -5.471173462 2.681788867 -13.1476114 2.78E-28 2.08E-26 53.63015956 DOWN RTP3

MCEE -1.234173248 3.631219969 -13.14405241 2.85E-28 2.13E-26 53.52686228 DOWN MCEE

YEATS2 1.08385035 3.780702858 13.14277344 2.87E-28 2.14E-26 53.49352528 UP YEATS2

NAA40 1.095606965 3.291806733 13.12012118 3.35E-28 2.49E-26 53.36679503 UP NAA40

ACAA2 -1.772239998 7.645461834 -13.1177829 3.41E-28 2.52E-26 53.25640832 DOWN ACAA2

RP11_7M8.2 -4.242432141 -2.60920418 -13.11164914 3.55E-28 2.62E-26 53.25382617 DOWN RP11-7M8.2

PRKAG2 -1.43217929 4.107373523 -13.10964629 3.60E-28 2.65E-26 53.26913787 DOWN PRKAG2

ADHFE1 -2.561778594 4.209515587 -13.10925945 3.61E-28 2.65E-26 53.28690538 DOWN ADHFE1

PDK4 -2.879182514 5.929646973 -13.09408513 4.00E-28 2.93E-26 53.1153497 DOWN PDK4

AGXT2 -4.045525263 3.98032267 -13.07866407 4.45E-28 3.24E-26 53.12669684 DOWN AGXT2

C16orf59 1.957586821 1.321251984 13.0605949 5.03E-28 3.65E-26 53.03931531 UP C16orf59

KCTD17 2.566355955 2.618139129 13.04038789 5.77E-28 4.17E-26 52.8645296 UP KCTD17

CDO1 -3.891494948 6.244632644 -13.04017485 5.78E-28 4.17E-26 52.75797338 DOWN CDO1

BEND3 1.595358471 1.454341008 13.01846145 6.71E-28 4.82E-26 52.75323827 UP BEND3

SPRYD4 -1.360904833 5.453354878 -13.01583078 6.83E-28 4.89E-26 52.5747729 DOWN SPRYD4

ACOX1 -1.490611008 7.429871602 -13.01307453 6.96E-28 4.97E-26 52.54372822 DOWN ACOX1

C3P1 -4.695986979 4.80852292 -13.00812027 7.20E-28 5.12E-26 52.62821436 DOWN C3P1

ADH4 -6.000480271 7.139768828 -12.99532244 7.85E-28 5.56E-26 52.47341313 DOWN ADH4

PRKCD 1.305407361 3.877982851 12.99528616 7.86E-28 5.56E-26 52.48934099 UP PRKCD

ABCA6 -3.062220723 5.073318421 -12.98998206 8.14E-28 5.75E-26 52.44566943 DOWN ABCA6

APOA5 -4.216985451 6.504514852 -12.98161154 8.62E-28 6.07E-26 52.35972911DOWN APOA5

RP11_422N16.3 -4.014107417 -1.874184849 -12.97540506 9.00E-28 6.30E-26 52.3606406 DOWN RP11-422N16.3

RP5_1120P11.1 3.771768859 -2.037997022 12.97475194 9.04E-28 6.30E-26 52.41315433 UP RP5-1120P11.1

F11 -2.908826255 5.072585534 -12.97469612 9.04E-28 6.30E-26 52.33699401 DOWN F11

RANBP10 -1.201653235 4.68579799 -12.96445981 9.69E-28 6.72E-26 52.24982779 DOWN RANBP10

SLC46A3 -2.950029192 4.477336718 -12.96267517 9.81E-28 6.78E-26 52.29062906 DOWN SLC46A3

SRD5A1 -1.850165842 4.962432931 -12.96039159 9.97E-28 6.87E-26 52.22147145 DOWN SRD5A1

PI4K2B -1.183744061 4.612511896-12.95270485 1.05E-27 7.22E-26 52.1730028 DOWN PI4K2B

NAT2 -5.186216926 0.906996102 -12.94183706 1.13E-27 7.75E-26 52.2197303 DOWN NAT2

SLC22A1 -5.781079869 4.922350322 -12.93819983 1.16E-27 7.92E-

26 52.1783866 DOWN SLC22A1

TCF19 1.750050368 3.174895405 12.9371435 1.17E-27 7.96E-26 52.13547852 UP TCF19

C4orf46 1.191518623 1.513793456 12.93612345 1.18E-27 7.99E-26 52.19733742 UP C4orf46

PEBP1 -1.606257044 9.960787982 -12.92821364 1.24E-27 8.41E-26 51.9992159 DOWN PEBP1

ATP5G1P4 2.452871733 -3.411372231 12.91594574 1.35E-27 9.09E-26 51.95048191 UP ATP5G1P4

GSTK1 -1.297790095 7.822222711-12.90874156 1.42E-27 9.52E-26 51.83816226 DOWN GSTK1

PXMP2 -1.854878273 5.456710519 -12.9026707 1.48E-27 9.90E-26 51.81237227 DOWN PXMP2

LINC01485 -5.177320491 3.693786571 -12.90047952 1.50E-27 1.00E-25 51.94867687 DOWN LINC01485

PDE2A -2.002460057 2.734419287 -12.89000744 1.61E-27 1.07E-25 51.86729978 DOWN PDE2A

GAREML 2.45699278 0.372448328 12.88137629 1.71E-27 1.13E-25 51.8343625 UP GAREML

CPEB3 -2.082955453 2.933723056 -12.87999814 1.72E-27 1.14E-25 51.79217706 DOWN CPEB3

RUNDC3B -2.982664263 1.972564561 -12.87568229 1.78E-27 1.17E-25 51.79555898 DOWN RUNDC3B

RCL1 -1.88248832 4.604652841 -12.87251088 1.81E-27 1.20E-25 51.64218373 DOWN RCL1

SUZ12P1 1.193449859 1.452096805 12.86938061 1.85E-27 1.22E-25 51.74793588 UP SUZ12P1

PBK 2.290145198 1.36691747 12.85240452 2.08E-27 1.36E-25 51.63230884 UP PBK

GNA14 -2.230918206 0.577191558 -12.84946613 2.12E-27 1.39E-25 51.60956957 DOWN GNA14

F11_AS1 -2.65114592 1.844298326 -12.84881068 2.13E-27 1.39E-25 51.61459201 DOWN F11-AS1

CENPW	1.938651575	1.77629326	12.84701494	2.16E-27	1.40E-25	51.58623913	UP	CENPW
FAM72D	2.561937989	-2.517281497	12.84033526	2.26E-27	1.46E-25	51.47886778	UP	FAM72D
SALL2	3.191552945	0.59253636	12.83168127	2.40E-27	1.55E-25	51.50012713	UP	SALL2
RBP5	-2.878051364	5.580337002	-12.83118809	2.41E-27	1.55E-25	51.34013643	DOWN	RBP5
PPFIA4	3.122113537	-2.107657109	12.82858496	2.45E-27	1.57E-25	51.42176241	UP	PPFIA4
G6PD	2.193577069	4.364685922	12.82770351	2.46E-27	1.58E-25	51.33896243	UP	G6PD
RP11_407B7.1	-2.710655196	0.263990872	-12.8199899	2.60E-27	1.66E-25	51.40044991	DOWN	RP11-407B7.1
ZNF93	2.371670792	-0.305906251	12.81954293	2.60E-27	1.66E-25	51.40837511	UP	ZNF93
GPSM2	1.585388687	1.733060795	12.80803568	2.82E-27	1.79E-25	51.3254864	UP	GPSM2
MCCC2	-1.161426458	6.706630487	-12.80526608	2.87E-27	1.82E-25	51.13084352	DOWN	MCCC2
CDC37L1	-1.271353129	4.555608545	-12.78821119	3.22E-27	2.04E-25	51.06132281	DOWN	CDC37L1
RP11_336K24.12	2.606551169	-3.181419684	12.78757947	3.24E-27	2.04E-25	51.104821	UP	RP11-336K24.12
CD14	-2.272630728	7.701497878	-12.78476265	3.30E-27	2.08E-25	50.99671602	DOWN	CD14
FAH	-1.659922872	7.023857258	-12.78113876	3.38E-27	2.12E-25	50.96828281	DOWN	FAH
RBP4	-3.25908562	11.40721801	-12.77837558	3.45E-27	2.16E-25	51.00517389	DOWN	RBP4
SORD	-2.323326639	6.875353081	-12.76820739	3.70E-27	2.31E-25	50.8834455	DOWN	SORD
ADRB2	-3.110327366	1.64246431	-12.76749808	3.71E-27	2.31E-25			

51.06797803 DOWN ADRB2

CTSO -1.612893621 5.538101322 -12.7553897 4.03E-27 2.51E-25

50.80816876 DOWN CTSO

MFSD10 1.595132803 4.356297917 12.7501053 4.18E-27 2.59E-25

50.80586592 UP MFSD10

CDC7 1.716334158 1.335880022 12.73363325 4.68E-27 2.89E-25

50.83285837 UP CDC7

FEN1 1.242120967 4.354194998 12.72564238 4.94E-27 3.04E-25

50.63664562 UP FEN1

GPT -2.862589417 5.872062942 -12.7239025 5.00E-27 3.07E-25

50.60351336 DOWN GPT

HGD -2.831317921 7.73732965 -12.72336869 5.02E-27 3.08E-25

50.58205688 DOWN HGD

NAV2_AS4 -2.891996048 -4.446634994 -12.71370542 5.36E-27 3.27E-25 50.56000815 DOWN NAV2-AS4

KLKB1 -2.674097521 5.739951692 -12.71075986 5.47E-27 3.33E-25

50.51499958 DOWN KLKB1

TET1 2.302461996 -0.383285938 12.70923544 5.52E-27 3.35E-25

50.66555609 UP TET1

UGT2B10 -4.386461102 5.961758517 -12.7035953 5.74E-27 3.48E-25

50.49725503 DOWN UGT2B10

GYS2 -5.678167074 3.210268592 -12.69346214 6.15E-27 3.71E-25

50.56516392 DOWN GYS2

KPNA2 1.397249037 5.34629456 12.68471141 6.53E-27 3.93E-25

50.32790294 UP KPNA2

GALNT15 -2.621233028 1.254729961 -12.68037134 6.72E-27 4.04E-25 50.48092163 DOWN GALNT15

N4BP2L1 -1.456986777 4.172561327 -12.67491038 6.98E-27 4.18E-25 50.31750641 DOWN N4BP2L1

ADORA2BP1 -4.540559462 -1.308677508 -12.67196716 7.12E-27

4.26E-25 50.35061289 DOWN ADORA2BP1

NFIA -1.405281193 5.524515293 -12.66807649 7.31E-27 4.36E-25

50.21414568 DOWN NFIA

CDKN3 2.351130349 1.908721083 12.65727879 7.87E-27 4.68E-25 50.2996424 UP CDKN3
SLC6A12 -3.29596621 4.488014815 -12.65501896 7.99E-27 4.74E-25 50.21235871 DOWN SLC6A12
KIF14 2.23339273 1.090833088 12.65258709 8.13E-27 4.81E-25 50.28960492 UP KIF14
DCLRE1C 1.309219389 1.603868104 12.64913183 8.32E-27 4.91E-25 50.25755622 UP DCLRE1C
LRRC1 2.789165801 2.121762741 12.6408619 8.80E-27 5.18E-25 50.18150858 UP LRRC1
SLC16A2 -2.360911967 5.227329358 -12.63835577 8.95E-27 5.26E-25 50.03685939 DOWN SLC16A2
CBR4 -1.344713798 4.666667444 -12.63651799 9.07E-27 5.31E-25 50.02897278 DOWN CBR4
E2F8 2.45694579 0.247826661 12.63624635 9.08E-27 5.31E-25 50.18392283 UP E2F8
HP -4.502462593 11.35750005 -12.62900652 9.54E-27 5.57E-25 49.99063035 DOWN HP
SERPINC1 -4.529541857 9.988496755 -12.60599911 1.12E-26 6.49E-25 49.81224634 DOWN SERPINC1
FLJ22763 -4.997216922 -1.70306558 -12.57410883 1.39E-26 8.05E-25 49.69255663 DOWN FLJ22763
RP11_141M3.6 -3.488963376 -0.687889499 -12.57150326 1.41E-26 8.18E-25 49.69571967 DOWN RP11-141M3.6
ALAS1 -2.01623925 7.56798327 -12.53420359 1.82E-26 1.05E-24 49.29652002 DOWN ALAS1
RHOB -1.671600418 8.525098253 -12.52836695 1.89E-26 1.09E-24 49.26588914 DOWN RHOB
NR1I3 -3.728381048 4.459078787 -12.52801102 1.90E-26 1.09E-24 49.36625333 DOWN NR1I3
B4GALT1_AS1 -1.916170882 -0.061209722 -12.52477327 1.94E-26 1.11E-24 49.41616899 DOWN B4GALT1-AS1
DEXI -1.183189842 2.692027744 -12.51433636 2.08E-26 1.19E-24

49.32015391 DOWN DEXI

NAP1L1 1.173530687 7.237517622 12.51037949 2.14E-26 1.22E-24 49.13778233 UP NAP1L1

SOGA1 1.466154343 3.658190244 12.50682641 2.19E-26 1.25E-24 49.19305521 UP SOGA1

MIR100HG -3.160404513 -2.197376231 -12.49591287 2.36E-26 1.34E-24 49.14663809 DOWN MIR100HG

PIPOX -2.884894907 7.406038331 -12.49515442 2.37E-26 1.34E-24 49.03452937 DOWN PIPOX

TTC36 -5.968431363 1.479868791 -12.4890245 2.48E-26 1.40E-24 49.18774132 DOWN TTC36

H2AFZ 1.142340084 6.246112488 12.47304515 2.76E-26 1.55E-24 48.88188054 UP H2AFZ

ANG -2.840026809 7.807116686 -12.47005796 2.82E-26 1.58E-24 48.86543097 DOWN ANG

TRAIP 1.835323278 1.163166558 12.46536579 2.91E-26 1.63E-24 49.02783277 UP TRAIP

CTSV 3.87536987 -0.604487055 12.46154137 2.98E-26 1.67E-24 49.00397153 UP CTSV

ASPM 2.315859875 3.113827799 12.45949649 3.03E-26 1.69E-24 48.91025121 UP ASPM

ETFA -1.136615365 7.300054073 -12.45778623 3.06E-26 1.70E-24 48.77634949 DOWN ETFA

TTC38 -1.583072028 7.044510951 -12.44742796 3.29E-26 1.82E-24 48.70586482 DOWN TTC38

CTD_2349P21.9 1.542179873 -1.875455149 12.44276319 3.39E-26 1.88E-24 48.82887939 UP CTD-2349P21.9

CCL16 -4.778084287 4.520851519 -12.43570563 3.56E-26 1.97E-24 48.7687846 DOWN CCL16

ALDH7A1 -1.585150413 6.787527122 -12.43538136 3.57E-26 1.97E-24 48.62427448 DOWN ALDH7A1

ZNF296 2.170875434 -0.537305356 12.43361063 3.61E-26 1.98E-24 48.81279425 UP ZNF296

ACSL1 -2.491162243 8.162350242 -12.42967534 3.71E-26 2.03E-24 48.5936069 DOWN ACSL1
HSD11B1 -5.082660601 5.771460009 -12.42727922 3.77E-26 2.06E-24 48.6641099 DOWN HSD11B1
HPX -3.630425253 10.20598618 -12.42641254 3.79E-26 2.07E-24 48.5991308 DOWN HPX
CYP4A22 -4.250327697 4.892576321 -12.41172428 4.19E-26 2.28E-24 48.57265359 DOWN CYP4A22
SPC24 1.945447458 2.471299564 12.41077788 4.22E-26 2.29E-24 48.6117 UP SPC24
CYP2A6 -6.86833804 5.620031528 -12.40888158 4.27E-26 2.32E-24 48.59905937 DOWN CYP2A6
RP11_108L7.15 2.265576709 -3.605642115 12.40389044 4.42E-26 2.39E-24 48.52681313 UP RP11-108L7.15
SLC6A13 -3.584116595 1.83499853 -12.3777101 5.28E-26 2.85E-24 48.44478676 DOWN SLC6A13
AZGP1 -3.39681379 8.662243862 -12.37197716 5.49E-26 2.96E-24 48.2093584 DOWN AZGP1
GABARAPL1 -1.92453707 6.167037343 -12.3613512 5.90E-26 3.17E-24 48.12944807 DOWN GABARAPL1
ACAA1 -1.646365468 7.268999423 -12.35906105 5.99E-26 3.21E-24 48.10832833 DOWN ACAA1
SDHD -1.051192299 6.29761765 -12.32912675 7.35E-26 3.93E-24 47.90511189 DOWN SDHD
SOX4 2.396127849 4.333603854 12.32508617 7.55E-26 4.03E-24 47.94012053 UP SOX4
RP4_539M6.20 -2.573207314 -1.89969148 -12.32013898 7.81E-26 4.16E-24 47.98046052 DOWN RP4-539M6.20
SUOX -1.14864214 5.245604418 -12.3153611 8.07E-26 4.29E-24 47.82983056 DOWN SUOX
ABCC6 -1.965435812 6.545976251 -12.3040052 8.72E-26 4.62E-24 47.73774458 DOWN ABCC6
SPATA3_AS1 2.529979381 -4.071152171 12.30132518 8.88E-26

4.70E-24 47.83602843 UP SPATA3-AS1

EMC3_AS1 1.855970562 -0.852853901 12.30040565 8.93E-26 4.72E-24 47.91231498 UP EMC3-AS1

IKBKE 2.1160261322.038235719 12.28162172 1.01E-25 5.33E-24 47.76060305 UP IKBKE

CFI -1.861554105 8.283258832 -12.26964974 1.10E-25 5.76E-24 47.51153899DOWN CFI

LIG1 1.034953751 4.482529367 12.26442739 1.14E-25 5.96E-24 47.5068436 UP LIG1

SNHG1 1.273694319 4.051829248 12.2626809 1.15E-25 6.02E-24 47.51825507 UP SNHG1

SPDYC -3.127623519 -0.311766672 -12.25699761 1.20E-25 6.24E-24 47.60973764 DOWN SPDYC

EMCN -1.731844681 2.993030001 -12.25496976 1.22E-25 6.31E-24 47.56150055 DOWN EMCN

BTD -1.4340856 5.463343662 -12.25394566 1.22E-25 6.34E-24 47.41134174DOWN BTD

RNFT2 2.33960221 -0.047174045 12.25281236 1.23E-25 6.38E-24 47.60747217 UP RNFT2

UGP2 -1.467250938 7.690977856 -12.25089992 1.25E-25 6.45E-24 47.37891944 DOWN UGP2

CIT 1.733730812 2.161369676 12.24871681 1.27E-25 6.53E-24 47.53403424 UP CIT

DDX11_AS1 1.859397094 -1.905771087 12.24504312 1.30E-25 6.68E-24 47.51197676UP DDX11-AS1

SPHK1 3.739417958 2.555376209 12.23966314 1.35E-25 6.90E-24 47.46362043 UP SPHK1

TMEM201 1.133395237 3.10441118412.2328785 1.41E-25 7.21E-24 47.37395836 UP TMEM201

ACKR2 -3.003093081 2.187579344 -12.22514206 1.49E-25 7.58E-24 47.41063144 DOWN ACKR2

CYP4A11 -4.099721612 6.942507482 -12.21335971 1.61E-25 8.20E-24 47.14259363 DOWN CYP4A11

CES5A -3.976225799 -0.208072014 -12.2094387 1.66E-25 8.40E-24 47.29047965 DOWN CES5A
AC016682.1 -2.631201432 -4.980181801 -12.20113595 1.75E-25 8.87E-24 47.15286631 DOWN AC016682.1
SFXN1 -1.186300934 6.146423012 -12.19153042 1.87E-25 9.45E-24 46.97619241 DOWN SFXN1
TTLL4 1.622304369 3.887900465 12.16987288 2.17E-25 1.09E-23 46.90409897 UP TTLL4
BAK1 1.328443433 3.562161954 12.16207062 2.29E-25 1.15E-23 46.86787766 UP BAK1
MFI2_AS1 2.148505868 -1.072743828 12.15750179 2.36E-25 1.18E-23 46.95321506 UP MFI2-AS1
ZMYND12 -2.57923548 0.682464124 -12.1511117 2.46E-25 1.23E-23 46.92453835 DOWN ZMYND12
SLCO2B1 -2.107719893 6.990992762 -12.13463498 2.76E-25 1.37E-23 46.59176128 DOWN SLCO2B1
DRP2 2.645090248 -4.158552356 12.13369841 2.77E-25 1.38E-23 46.7210921 UP DRP2
RAMP3 -2.177747308 3.25383944 -12.13349162 2.78E-25 1.38E-23 46.73572552 DOWN RAMP3
GSTA12P -3.048370136 -4.117094323 -12.13186624 2.81E-25 1.39E-23 46.69331789 DOWN GSTA12P
PIGS 1.101205201 4.130663141 12.12773328 2.89E-25 1.43E-23 46.60126523 UP PIGS
FAM107A -1.872697968 2.223357013 -12.12673549 2.91E-25 1.43E-23 46.73839128 DOWN FAM107A
NUDT7 -1.826063326 3.004867928 -12.11948715 3.05E-25 1.50E-23 46.64946034 DOWN NUDT7
WHSC1 1.170656018 4.685017513 12.11638262 3.12E-25 1.53E-23 46.49939763 UP WHSC1
RP4_580N22.2 -3.145922205 -3.071205767 -12.08924337 3.75E-25 1.83E-23 46.4232892 DOWN RP4-580N22.2
OTC -4.722479657 4.75257621 -12.08556009 3.84E-25 1.88E-23

46.39683391 DOWN OTC

RP4_798A17.5 -2.404601066 -1.097879628 -12.08139349 3.96E-25 1.93E-23 46.41763492 DOWN RP4-798A17.5

RCOR2 3.089668708 -1.724690278 12.07786755 4.05E-25 1.97E-23 46.41318198 UP RCOR2

DBF4B 1.231424822 1.795146573 12.07593172 4.10E-25 1.99E-23 46.3901443 UP DBF4B

CPED1 -2.746010147 2.782330658 -12.07331644 4.18E-25 2.02E-23 46.36694683 DOWN CPED1

TPMT -1.160685817 5.660579486 -12.06086905 4.55E-25 2.20E-23 46.10017163 DOWN TPMT

CYP2C9 -4.137990196 7.043053922 -12.05504391 4.73E-25 2.28E-23 46.07258466 DOWN CYP2C9

C16orf45 -2.006240899 3.324132423 -12.05268882 4.81E-25 2.31E-23 46.18334443 DOWN C16orf45

BRCA1 1.37660101 2.377813335 12.03825161 5.30E-25 2.55E-23 46.10651968 UP BRCA1

TMED3 2.733165604 4.032135674 12.03499466 5.42E-25 2.60E-23 46.00255213 UP TMED3

GPD1 -3.385360784 4.856705916 -12.0338994 5.46E-25 2.61E-23 45.99788733 DOWN GPD1

MPDZ -2.062732646 4.880765254 -12.02775329 5.69E-25 2.72E-23 45.91751287 DOWN MPDZ

SH3D21 1.60698294 0.392255984 12.02072269 5.97E-25 2.84E-23 46.0522396 UP SH3D21

MTMR2 1.23022942 3.125995482 12.02056438 5.98E-25 2.84E-23 45.94124309 UP MTMR2

TRIM17 3.357145907 -2.721815173 12.01965524 6.01E-25 2.85E-23 46.00844814 UP TRIM17

FAM72A 1.788805858 -1.724807569 12.01151255 6.35E-25 3.01E-23 45.96010069 UP FAM72A

PINK1 -1.554612263 4.760229706 -11.99771021 6.98E-25 3.30E-23 45.70921907 DOWN PINK1

RDH16 -4.404067633 5.471429111-11.98920252 7.39E-25 3.49E-23
45.69442946 DOWN RDH16

RDH5 -2.537639899 2.173327997 -11.98779503 7.46E-25 3.51E-23
45.81273766 DOWN RDH5

COL9A2 2.89808021 0.72911862611.986477047.53E-25 3.54E-23
45.81602957 UP COL9A2

PRKAG2_AS1 -2.305049206 1.450046793 -11.96106685 8.95E-25
4.20E-23 45.64975488 DOWN PRKAG2-AS1

CRAT -1.163453182 6.955805657 -11.95732445 9.18E-25 4.30E-23
45.39210223 DOWN CRAT

REPS2 -1.665205038 3.377223648 -11.95682696 9.21E-25 4.30E-23
45.5270352 DOWN REPS2

CRADD -1.109350246 3.916478616 -11.95489257 9.33E-25 4.35E-23 45.46159961 DOWN CRADD

FAM72B 2.249169512 -2.280146347 11.945162 9.97E-25 4.64E-23
45.50904336 UP FAM72B

CLIC1 1.383099997 7.168749093 11.942484541.01E-24 4.71E-23
45.29927553 UP CLIC1

FAM210B -1.20816363 6.352458313 -11.94206533 1.02E-24 4.72E-23
45.29043341 DOWN FAM210B

RP5_858L17.1 2.440233939 -2.336596111 11.937470781.05E-24 4.86E-23 45.45942584 UP RP5-858L17.1

NEIL3 2.496438887 -0.615486087 11.915545541.22E-24 5.61E-23
45.34670627 UP NEIL3

SDHB -1.174082191 6.558205125 -11.91546774 1.22E-24 5.61E-23
45.1100531 DOWN SDHB

BPHL -1.470269257 5.463603942 -11.90073449 1.35E-24 6.18E-23
45.02735476 DOWN BPHL

ALDH9A1 -1.231568509 6.779153675 -11.89543836 1.40E-24 6.40E-23 44.97481883 DOWN ALDH9A1

C8B -3.243327899 7.375164514 -11.88342462 1.51E-24 6.93E-23
44.90284744 DOWN C8B

UGT2B15 -3.922719332 6.635210865 -11.87984491 1.55E-24 7.08E-

23 44.89629444 DOWN UGT2B15

MUT -1.430390197 6.32917122 -11.87201168 1.64E-24 7.45E-23 44.81936877 DOWN MUT

TST -1.663543576 8.039956881 -11.86687541 1.69E-24 7.70E-23 44.78981256 DOWN TST

SLC31A1 -1.17784707 6.441874721 -11.86579525 1.71E-24 7.74E-23 44.77574902 DOWN SLC31A1

MIS18A 1.037948786 2.552370175 11.86103555 1.76E-24 7.98E-23 44.90571063 UP MIS18A

CCND2P1 -4.729309137 0.662352568 -11.85725404 1.81E-24 8.17E-23 44.95543322 DOWN CCND2P1

F12 -3.600192271 7.804624397 -11.85666797 1.82E-24 8.19E-23 44.72487027 DOWN F12

EPHX1 -2.388946755 10.48777551 -11.85327009 1.86E-24 8.37E-23 44.73597921 DOWN EPHX1

CD300LG -3.938753276 -1.719700909 -11.84583249 1.95E-24 8.77E-23 44.83711671 DOWN CD300LG

CFAP57 -2.85919248 -0.875892014 -11.84149316 2.01E-24 9.01E-23 44.82610176 DOWN CFAP57

FH -1.233192903 7.432095468 -11.82845686 2.20E-24 9.82E-23 44.52568116 DOWN FH

SORBS2 -1.988082979 5.957019599 -11.82533594 2.24E-24 1.00E-22 44.51436341 DOWN SORBS2

MMS22L 1.552713522 1.452280349 11.82368348 2.27E-24 1.01E-22 44.70928961 UP MMS22L

TMEM220 -2.18127012 4.657935157 -11.82181329 2.30E-24 1.02E-22 44.54391645 DOWN TMEM220

RP11_252E2.2 -3.430405197 -3.744592947 -11.82053705 2.32E-24 1.03E-22 44.6344863 DOWN RP11-252E2.2

GNE -1.711041857 5.728807977 -11.80573543 2.56E-24 1.14E-22 44.38405504 DOWN GNE

SELENBP1 -2.383259092 7.361924795 -11.78975902 2.86E-24 1.26E-22 44.26717629 DOWN SELENBP1

DHRS12 -1.36171663 3.634152249 -11.78747769 2.90E-24 1.28E-22 44.36071016 DOWN DHRS12

AC005077.7 -3.058046166 -4.297866201 -11.78493543 2.95E-24 1.30E-22 44.3945013 DOWN AC005077.7

RECQL4 1.857561079 3.541909683 11.78109139 3.03E-24 1.33E-22 44.30757449 UP RECQL4

TMEM88 -1.593751094 1.326629311 -11.76720522 3.33E-24 1.46E-22 44.35176297 DOWN TMEM88

OGDHL -3.302348528 5.375966248 -11.76330354 3.42E-24 1.50E-22 44.14385459 DOWN OGDHL

BAAT -2.899965355 8.440581123 -11.75237088 3.68E-24 1.61E-22 44.02407864 DOWN BAAT

CTD_2194A8.2 -2.558796713 -2.573580669 -11.74883035 3.77E-24 1.64E-22 44.17197047 DOWN CTD-2194A8.2

ACOT2 -1.373589646 4.61014344 -11.74340596 3.91E-24 1.70E-22 43.99989166 DOWN ACOT2

UBE2S 1.579560836 3.364998131 11.74158252 3.96E-24 1.72E-22 44.05071251 UP UBE2S

DNASE1L3 -2.982129725 3.303238139 -11.74019319 3.99E-24 1.73E-22 44.10670899 DOWN DNASE1L3

RMDN2 -1.339896095 2.96202719 -11.73630796 4.10E-24 1.78E-22 44.06733457 DOWN RMDN2

CFHR2 -3.548290903 2.061274663 -11.73450438 4.15E-24 1.80E-22 44.12745697 DOWN CFHR2

UPB1 -3.797126562 6.073063348 -11.73252589 4.21E-24 1.82E-22 43.91835906 DOWN UPB1

PNMA1 1.422780489 3.389587727 11.73218072 4.22E-24 1.82E-22 43.98492312 UP PNMA1

LINC01093 -4.710648414 -0.782134014 -11.7308983 4.25E-24 1.83E-22 44.09112617 DOWN LINC01093

TIGD1 1.426186623 1.164446672 11.73006322 4.28E-24 1.84E-22 44.09296489 UP TIGD1

MPZL1 1.042976358 5.838952975 11.71765927 4.65E-24 1.99E-22

43.78363297 UP MPZL1

RP11_461O7.1 -3.503506307 -3.592883911 -11.71690304 4.68E-24 2.00E-22 43.95110733DOWN RP11-461O7.1

DECR1 -1.398032725 7.375133581 -11.70982667 4.90E-24 2.09E-22 43.72699837 DOWN DECR1

HSD17B8 -1.628089837 4.847600662 -11.70250236 5.15E-24 2.20E-22 43.71814673 DOWN HSD17B8

LINC00634 2.378792874 -4.354327385 11.700409985.23E-24 2.22E-22 43.84268996 UP LINC00634

DEFB132 -4.659519428 -2.290605364 -11.68940172 5.63E-24 2.39E-22 43.79538593 DOWN DEFB132

INPP5J 2.663526614 -0.968410638 11.684173925.83E-24 2.47E-22 43.80126623 UP INPP5J

KIAA2012 -2.735403381 -1.95438138-11.68266947 5.89E-24 2.49E-22 43.74642271 DOWN KIAA2012

RP11_736K20.4 -1.776230673 0.211488384-11.67821647 6.07E-24 2.57E-22 43.76478587 DOWN RP11-736K20.4

SLC28A1 -4.084950687 3.421361038 -11.67644522 6.15E-24 2.59E-22 43.69940344 DOWN SLC28A1

RP11_659E9.2 -3.040716278 -2.781093128 -11.67039612 6.40E-24 2.70E-22 43.65247634 DOWN RP11-659E9.2

KB_68A7.1 -3.219951896 -0.336590052 -11.66513153 6.63E-24 2.79E-22 43.66593916 DOWN KB-68A7.1

DAO -3.708313531 4.25795732 -11.66119757 6.81E-24 2.86E-22 43.5396533 DOWN DAO

ACO1 -1.18197904 6.862595398 -11.64718292 7.49E-24 3.14E-22 43.30383986 DOWN ACO1

F9 -5.209324309 5.674662559 -11.64702044 7.50E-24 3.14E-22 43.40873546 DOWN F9

RP11_963H4.3 -3.042673907 -2.652719778 -11.64220802 7.75E-24 3.23E-22 43.46823448 DOWN RP11-963H4.3

TPPP2 -2.897302793 -0.863397462 -11.63727682 8.01E-24 3.34E-22 43.47071889 DOWN TPPP2

C3orf52 3.603797125 -2.049057013 11.636155598.07E-24 3.36E-22 43.47232589 UP C3orf52

HMGB2 1.264040472 4.944394703 11.633976348.19E-24 3.40E-22 43.24127427 UP HMGB2

PCTP -1.303158428 5.537637351 -11.63213712 8.29E-24 3.44E-22 43.21623513 DOWN PCTP

ENPEP -2.120804699 4.77717197 -11.6243368 8.74E-24 3.62E-22 43.20904864 DOWN ENPEP

AOX1 -3.405088647 8.42656982 -11.62394521 8.76E-24 3.62E-22 43.16179828 DOWN AOX1

PLEKHB1 4.13835174 -0.09547741 11.614261969.36E-24 3.86E-22 43.33739674 UP PLEKHB1

MASP1 -2.450713579 6.044476996 -11.61268733 9.46E-24 3.89E-22 43.08786979 DOWN MASP1

RORA -1.668520293 4.580026962 -11.59540325 1.06E-23 4.37E-22 43.01458642 DOWN RORA

CDC14B -1.816694985 4.527908629 -11.59464689 1.07E-23 4.38E-22 43.01735109 DOWN CDC14B

CCL14 -2.292890026 0.952775673 -11.58586544 1.13E-23 4.63E-22 43.14900243 DOWN CCL14

KCNJ8 -2.464173374 4.540914993 -11.58068389 1.17E-23 4.79E-22 42.93922669 DOWN KCNJ8

RP11_468N14.3 -4.032125237 -0.937686431 -11.57774923 1.20E-23 4.88E-22 43.07228015 DOWN RP11-468N14.3

OIP5 1.865437353 0.182485265 11.559558221.35E-23 5.50E-22 42.97349862 UP OIP5

ZNF695 3.335689314 -3.888560778 11.556708691.38E-23 5.59E-22 42.9138776 UP ZNF695

SOX12 1.533829469 4.333517341 11.551514771.43E-23 5.78E-22 42.71641774 UP SOX12

TFR2 -2.87171728 8.696566434 -11.54970943 1.45E-23 5.85E-22 42.665119 DOWN TFR2

ANKRD24 -1.800667262 1.970375953 -11.53174718 1.63E-23 6.59E-

22 42.76075194 DOWN ANKRD24

MCM5 1.234645012 5.300526579 11.53031126 1.65E-23 6.64E-22 42.53516006 UP MCM5

RP11_74M13.4 -2.788250701 -3.415626392 -11.51960441 1.77E-23 7.11E-22 42.64597553 DOWN RP11-74M13.4

LIMK1 1.354401917 3.737346633 11.5158876 1.82E-23 7.28E-22 42.51051334 UP LIMK1

C12orf49 1.38699498 4.096570623 11.51492754 1.83E-23 7.32E-22 42.48247877 UP C12orf49

IMMP2L -1.33324363 3.80181853 -11.50836176 1.91E-23 7.64E-22 42.47563644 DOWN IMMP2L

KIF20B 1.362707506 1.945516263 11.50600374 1.94E-23 7.74E-22 42.56136051 UP KIF20B

PNCK 4.569895905 -2.942455751 11.50594147 1.94E-23 7.74E-22 42.60620083 UP PNCK

COL18A1 -1.345774494 9.481030199 -11.50109715 2.01E-23 7.98E-22 42.35306506 DOWN COL18A1

CPB2 -2.719225695 8.352026663 -11.50060818 2.02E-23 7.99E-22 42.33162103 DOWN CPB2

LINC01354 -2.986262197 -2.74313591 -11.49231803 2.13E-23 8.44E-22 42.47624686 DOWN LINC01354

ABHD14B -1.354872305 6.821970434 -11.48749162 2.20E-23 8.70E-22 42.23210271 DOWN ABHD14B

CPS1 -4.822730882 8.55756886 -11.4760461 2.38E-23 9.38E-22 42.17706758 DOWN CPS1

AFM -4.109622656 5.824352445 -11.47268192 2.43E-23 9.57E-22 42.19458558 DOWN AFM

KRBA1 1.795452501 1.424950778 11.46840887 2.50E-23 9.83E-22 42.33354005 UP KRBA1

ABCA9 -2.553306312 1.754050969 -11.45357662 2.77E-23 1.08E-21 42.25319276 DOWN ABCA9

MAT1A -3.130313761 8.820059697 -11.45238387 2.79E-23 1.09E-21 42.01426472 DOWN MAT1A

NT5DC2 2.2104611313.848059535 11.444206032.95E-23 1.15E-21
42.03423202 UP NT5DC2
DNMT3A 1.294808103 4.325298695 11.436424783.11E-23 1.21E-21
41.94302371 UP DNMT3A
SARDH -2.251264871 5.984604282 -11.43241317 3.19E-23 1.24E-21
41.87679717 DOWN SARDH
KLHL2 -1.215145411 3.859714586 -11.43138514 3.21E-23 1.25E-21
41.95320138 DOWN KLHL2
WNK2 4.949515767 1.003556851 11.430857093.23E-23 1.25E-21
42.09410834 UP WNK2
FMO3 -3.501717274 7.11433437 -11.42841953 3.28E-23 1.27E-21
41.84913031 DOWN FMO3
A1BG -3.703030379 5.345058375 -11.42295359 3.40E-23 1.31E-21
41.87117048DOWN A1BG
FANCE 1.452067786 1.337349312 11.411574913.67E-23 1.42E-21
41.96011748UP FANCE
TIGD2 -1.715686052 3.575391266 -11.41082735 3.69E-23 1.42E-21
41.85041925 DOWN TIGD2
NREP 1.582200559 4.660722871 11.408453073.75E-23 1.44E-21
41.74303013 UP NREP
AL161668.5 -3.037694097 -0.69474104 -11.40817748 3.76E-23 1.44E-21
41.95662639 DOWN AL161668.5
AMDHD1 -2.680286989 5.023676806 -11.40735255 3.78E-23 1.45E-
21 41.75485922 DOWN AMDHD1
SRSF12 2.336457775 -0.905991552 11.406776873.79E-23 1.45E-21
41.95917001 UP SRSF12
ZNF431 1.71191657 1.36044525 11.405043913.84E-23 1.46E-21
41.91404856 UP ZNF431
SRD5A2 -4.61946944 2.213296343 -11.40127984 3.94E-23 1.50E-21
41.90976549 DOWN SRD5A2
TTPA -3.215284442 4.509691641 -11.39895381 4.00E-23 1.52E-21
41.75136268 DOWN TTPA
BCAS4 1.862968094 1.397313221 11.389621944.26E-23 1.62E-21

41.80887769 UP BCAS4
TSKU -2.537452552 6.887782418 -11.38224524 4.47E-23 1.70E-21 41.53238688 DOWN TSKU
MAPK13 2.975263004 2.626099746 11.380256154.54E-23 1.72E-21 41.68548265 UP MAPK13
GINS3 1.200948777 1.385471223 11.376810934.64E-23 1.75E-21 41.72814278 UP GINS3
OAF -1.318586639 7.426120805 -11.37628739 4.66E-23 1.76E-21 41.48893617 DOWN OAF
ZDHHC13 1.964029189 1.473002167 11.372874824.77E-23 1.80E-21 41.69377372 UP ZDHHC13
GBA3 -4.28771054 2.962764371 -11.37144809 4.81E-23 1.81E-21 41.68493934 DOWN GBA3
DBF4 1.062765356 2.13477306 11.369369934.88E-23 1.83E-21 41.63999424 UP DBF4
MTBP 1.377818757 0.831329332 11.365329565.01E-23 1.88E-21 41.67012831 UP MTBP
ESCO2 1.967264038 0.044810361 11.3599292 5.20E-23 1.95E-21 41.64736014 UP ESCO2
LHFPL3_AS2 4.085957775 -3.172211825 11.345494365.73E-23 2.14E-21 41.53723919 UP LHFPL3-AS2
CYB5D2 -1.32614808 4.275179914 -11.33797933 6.03E-23 2.25E-21 41.30022088 DOWN CYB5D2
DCAF11 -1.177435512 6.764219522 -11.33575856 6.12E-23 2.28E-21 41.21532402 DOWN DCAF11
DHRS3 -1.285654056 7.704915158 -11.33103783 6.32E-23 2.35E-21 41.18830845 DOWN DHRS3
LIMS2 -1.566936196 4.628951581 -11.33031057 6.35E-23 2.36E-21 41.23301913 DOWN LIMS2
ACY1 -1.409658211 3.800750149 -11.32059618 6.78E-23 2.51E-21 41.21982604 DOWN ACY1
ALDOB -4.087982921 10.12515324 -11.30662871 7.44E-23 2.76E-21 41.05909347 DOWN ALDOB

LAMA5_AS1 -4.636406282 0.337019435 -11.30643823 7.45E-23 2.76E-21 41.29333507 DOWN LAMA5-AS1
GSTA7P -3.501205574 -0.708934768 -11.3006608 7.75E-23 2.86E-21 41.24438825 DOWN GSTA7P
ANKRD13D 1.084456969 3.607249418 11.29636706 7.98E-23 2.94E-21 41.04874807 UP ANKRD13D
PPP1R32 -1.549733621 0.662734958 -11.29419659 8.09E-23 2.98E-21 41.2116396 DOWN PPP1R32
DBN1 2.054592102 4.200284902 11.29123266 8.26E-23 3.03E-21 40.98842588 UP DBN1
UCK2 1.157020278 4.581744588 11.28542783 8.59E-23 3.15E-21 40.91903733 UP UCK2
ACADL -4.653716736 1.556976674 -11.27993664 8.91E-23 3.26E-21 41.11553928 DOWN ACADL
TEAD2 1.593586364 4.012931338 11.27906991 8.96E-23 3.28E-21 40.91159467 UP TEAD2
SLC1A5 2.230487387 3.772160673 11.27868657 8.98E-23 3.28E-21 40.93223995 UP SLC1A5
MARCKS 1.373692569 6.108149047 11.27819189 9.01E-23 3.29E-21 40.83618225 UP MARCKS
FBXO5 1.396211824 1.428977851 11.26915713 9.58E-23 3.49E-21 41.00842907 UP FBXO5
ZNF816_ZNF321P 2.356152424 -1.723276145 11.26898914 9.59E-23 3.49E-21 41.03877139 UP ZNF816-ZNF321P
SH3BP1 1.852621786 2.270575673 11.25120173 1.08E-22 3.92E-21 40.84033437 UP SH3BP1
LINC01352 -2.527777483 -2.891785171 -11.23840448 1.18E-22 4.27E-21 40.79950248 DOWN LINC01352
ALDH8A1 -3.121116268 5.213021555 -11.23247968 1.23E-22 4.43E-21 40.58997633 DOWN ALDH8A1
ABHD6 -1.706389684 4.584813898 -11.23104866 1.24E-22 4.47E-21 40.57523271 DOWN ABHD6
IGSF23 -3.740966729 2.343404305 -11.22824909 1.26E-22 4.55E-21

40.74637387	DOWN IGSF23
IGF2BP2	3.307566788	2.224501472	11.21043139 1.42E-22	5.12E-21	40.57573685	UP	IGF2BP2
AC004160.4 -3.086260148	-4.461619693	-11.21001777	1.43E-22	5.13E-21	40.60493491	DOWN AC004160.4
HFE2	-3.952555724	6.366227047	-11.20928388	1.43E-22	5.14E-21	40.40432925	DOWN HFE2
PDE7A 1.259335766	2.948307571	11.20736958 1.45E-22	5.20E-21	40.50297124	UP	PDE7A
BRCA2 1.38245693	1.033924001	11.20668561 1.46E-22	5.22E-21	40.60950457	UP	BRCA2
AACS	1.342351962	3.588910099	11.20172824 1.51E-22	5.38E-21	40.42017889	UP	AACS
WDR76 1.665297522	1.716142825	11.20092491 1.52E-22	5.40E-21	40.53876325	UP	WDR76
RCAN1 -1.623065689	5.251762461	-11.19895768	1.54E-22	5.46E-21	40.32809407	DOWN RCAN1
CEP131 1.064958446	3.434387812	11.19546791 1.57E-22	5.59E-21	40.38768356	UP	CEP131
ASMTL -1.166486023	5.08865812 -11.18775492	1.66E-22	5.86E-21	40.25245839	DOWN ASMTL
AP001469.9 1.692126482	-0.786713988	11.18077155 1.73E-22	6.14E-21	40.46362079	UP	AP001469.9
CYP4F3	-2.835766493	6.584925418	-11.1694608 1.87E-22	6.61E-21	40.11569347DOWN CYP4F3
FAHD2A	-1.089935099	4.729522369	-11.16625259	1.91E-22	6.75E-21	40.12328176	DOWN FAHD2A
MMD	1.488205472	3.615299451	11.16534159 1.92E-22	6.78E-21	40.17729126	UP	MMD
LINC01057 2.525176449	-1.466624602	11.16406488 1.94E-22	6.83E-21	40.3497519 UP LINC01057
SOD1	-1.428370741	8.851665762	-11.16373609	1.94E-22	6.83E-21	40.08548375	DOWN SOD1

TMEM56	-1.92322878 6.043641316	-11.14798279	2.16E-22	7.58E-21	39.97080604	DOWN	TMEM56
RP11_424C20.2	2.508931061	-3.502063454	11.13188988 2.41E-22	8.42E-21	40.11100967 UP	RP11-424C20.2
NFE2L3	1.861989013	2.319799306	11.13188069 2.41E-22	8.42E-21	40.04330177	UP	NFE2L3
C1R	-1.805312382	9.595691265	-11.12748886	2.48E-22	8.66E-21	39.85602369	DOWN	C1R
AIFM1	-1.145976356	6.672555699	-11.10935357	2.80E-22	9.75E-21	39.70323762	DOWN	AIFM1
LHPP	-1.654242848	5.126896544	-11.1076212 2.83E-22	9.85E-21	39.72379747	DOWN	LHPP
DQX1	4.351102451 -2.813692642	11.10692464 2.85E-22	9.88E-21	39.97027507	UP	DQX1
SULT1B1	-3.634577885	2.914143078	-11.10562606	2.87E-22	9.96E-21	39.90662631	DOWN	SULT1B1
GADD45G	-2.388066041	5.316581011 -11.10364185	2.91E-22	1.01E-20	39.7042799	DOWN	GADD45G
ACMSD	-2.75883904 4.992954649	-11.10159353	2.95E-22	1.02E-20	39.71752882	DOWN	ACMSD
GTF2IRD2B	-1.140608982	1.612432734	-11.0906302 3.18E-22	1.10E-20	39.83532337	DOWN	GTF2IRD2B
ZNF714 2.404977988	-0.548574199	11.08842728 3.22E-22	1.11E-20	39.8536645	UP	ZNF714
TONSL 1.369041572	3.497120101	11.08556502 3.29E-22	1.13E-20	39.65287668	UP	TONSL
ACOX2 -2.474449774	5.919239977	-11.08254468	3.35E-22	1.15E-20	39.54407389	DOWN	ACOX2
F13B	-3.473708051	5.649372286	-11.07971927	3.42E-22	1.17E-20	39.55813076	DOWN	F13B
CRY2	-1.255387291	5.920875512	-11.07299101	3.57E-22	1.22E-20	39.467215	DOWN	CRY2
TPRG1_AS1	-3.391914978	0.613461914	-11.07160642	3.61E-22

1.23E-20 39.74250355 DOWN TPRG1-AS1
FITM1 -2.311658552 0.08234769 -11.06303716 3.82E-22 1.30E-20
39.6867202 DOWN FITM1
CYP3A4 -7.17206797 5.904406991 -11.06200708 3.85E-22 1.31E-20
39.56610059 DOWN CYP3A4
LINC00205 1.371233422 0.396670377 11.061408413.86E-22 1.31E-20
39.66514494 UP LINC00205
CHP1 -1.026559299 7.427404112-11.06125687 3.87E-22 1.31E-20
39.38511116DOWN CHP1
MGST1 -2.25608271 8.493665627 -11.04776625 4.23E-22 1.43E-20
39.30647148 DOWN MGST1
CA9 5.427830649 -0.89500707 11.034300094.63E-22 1.56E-20
39.49370752 UP CA9
PROZ -3.43049869 3.211247562-11.01113604 5.41E-22 1.82E-20
39.25858471 DOWN PROZ
MT_ND6 -1.811455857 9.661432366 -11.00607955 5.60E-22 1.89E-20 39.04881952 DOWN MT-ND6
CKAP4 1.278172454 6.496573122 11.000001225.83E-22 1.96E-20
38.97931831 UP CKAP4
LCAT -2.509704672 4.838217691 -10.99418449 6.06E-22 2.03E-20
39.00393085 DOWN LCAT
ALDH1L1 -3.811751048 7.460041786 -10.99314183 6.10E-22 2.04E-20 38.94356603 DOWN ALDH1L1
MSRA -1.40955909 4.326744574 -10.9852524 6.43E-22 2.15E-20
38.94769997 DOWN MSRA
POLA1 1.030825152 2.778809808 10.97669972 6.81E-22 2.28E-20
38.98224968 UP POLA1
CXorf66 -2.725217038 -4.814019342 -10.97525386 6.88E-22 2.29E-20 39.06625325 DOWN CXorf66
RP11_132A1.6 -3.513683032 -1.323882348 -10.96789555 7.23E-22
2.40E-20 39.04594682 DOWN RP11-132A1.6
SNRPB 1.030456432 6.866138312 10.9660815 7.31E-22 2.43E-20
38.75341523 UP SNRPB

MFI2 3.048521721 1.762147953 10.96418772 7.41E-22 2.45E-20
38.9618335 UP MFI2
NRSN2 2.379563347 2.772599249 10.96414162 7.41E-22 2.45E-20
38.90250963 UP NRSN2
DSG1 -4.633328276 0.976024168 -10.96408387 7.41E-22 2.45E-20
39.03297112DOWN DSG1
AC012358.8-1.62346789 0.038667865 -10.96248185 7.49E-22 2.48E-20
39.02394002 DOWN AC012358.8
CYP27A1 -2.266191626 8.407065126 -10.95823084 7.71E-22 2.54E-20 38.7097574 DOWN CYP27A1
MCM7 1.214461516 5.910154173 10.9570737 7.77E-22 2.56E-20
38.69604389 UP MCM7
AC009166.5-2.718234505 -1.267007729 -10.94544496 8.40E-22 2.76E-20 38.89985283 DOWN AC009166.5
BCKDHB -1.562235859 4.354347699 -10.93644946 8.92E-22 2.93E-20 38.62629428 DOWN BCKDHB
RP1_28H20.3 -2.005688229 -1.917066538 -10.93588299 8.95E-22
2.93E-20 38.82753904 DOWN RP1-28H20.3
NADK2-1.605161322 7.134216379 -10.93480287 9.02E-22 2.95E-20
38.54291875 DOWN NADK2
EPM2A -1.135784594 2.062481786 -10.93344928 9.10E-22 2.97E-20
38.77151157DOWN EPM2A
C8orf46-2.414225416 0.939427706 -10.93241504 9.16E-22 2.99E-20
38.81793297 DOWN C8orf46
LINC00959 -1.53306603-0.352608665 -10.92976992 9.32E-22 3.04E-20
38.80887803 DOWN LINC00959
PANK1 -1.547994585 5.070605286 -10.9293633 9.35E-22 3.04E-20
38.53839507 DOWN PANK1
PITX1 4.771279068 -0.570012212 10.92865946 9.39E-22 3.05E-20
38.79619923 UP PITX1
KCNMB3 1.78825619 -0.473421621 10.91850748 1.01E-21 3.26E-20
38.73377161 UP KCNMB3
WDR54 1.628039789 1.65570173 10.91760616 1.01E-21 3.28E-20

38.66336787 UP WDR54

ARID3C -2.773518382 0.359336421 -10.91501035 1.03E-21 3.32E-20 38.71111033 DOWN ARID3C

RP11_286E11.1 -2.342074241 -3.362456257 -10.90944325 1.07E-21 3.44E-20 38.63801846 DOWN RP11-286E11.1

CTB_193M12.5 1.364926026 0.910854372 10.90828701 1.08E-21 3.47E-20 38.63840414 UP CTB-193M12.5

SEC14L3 -3.848322784 -1.920213606 -10.90399177 1.11E-21 3.56E-20 38.62208978 DOWN SEC14L3

IYD -3.411313164 2.864013063 -10.90135807 1.13E-21 3.61E-20 38.55062308 DOWN IYD

NMRK1 -1.070745135 4.256598553 -10.89421743 1.18E-21 3.79E-20 38.34043833 DOWN NMRK1

SULT2A1 -4.415457219 7.157834556 -10.88620525 1.25E-21 3.98E-20 38.24446052 DOWN SULT2A1

CYP2J2 -2.392259092 5.389523988 -10.87980461 1.30E-21 4.15E-20 38.2125371 DOWN CYP2J2

SFI1 1.259271769 3.4701633 10.8767893 1.33E-21 4.23E-20 38.26754829 UP SFI1

PLG -3.528599567 9.188199558 -10.87378253 1.36E-21 4.31E-20 38.16046815 DOWN PLG

SLC13A5 -4.213405251 6.5039219 -10.86314873 1.46E-21 4.61E-20 38.10216812 DOWN SLC13A5

MOCS2 -1.030570328 5.611814915-10.85543476 1.53E-21 4.84E-20 38.02482597 DOWN MOCS2

ACADM -1.607160574 6.171375338 -10.85542504 1.53E-21 4.84E-20 38.01999242 DOWN ACADM

RP11_403I13.5 -4.800942346 -0.314013077 -10.84761378 1.61E-21 5.09E-20 38.26641089 DOWN RP11-403I13.5

RNF24 1.285945996 2.718565264 10.84503876 1.64E-21 5.17E-20 38.1137141 UP RNF24

DCAF16 1.073936274 3.356500694 10.83842065 1.72E-21 5.39E-20 38.02087314 UP DCAF16

RP11_119D9.1 -4.138027118 0.488308569 -10.83780327 1.72E-21
5.41E-20 38.20448162 DOWN RP11-119D9.1
ZBTB16 -2.732550049 2.118232009-10.83447299 1.76E-21 5.52E-20
38.13551006 DOWN ZBTB16
SLC9A3R2 -1.480409521 7.325339608 -10.8326826 1.78E-21 5.58E-20
37.86558508 DOWN SLC9A3R2
SLC25A13 -1.217982335 6.503369813 -10.82721459 1.85E-21 5.78E-20 37.82806642 DOWN SLC25A13
ETNK2 -2.483690919 5.952488522 -10.82250544 1.91E-21 5.95E-20
37.81571717 DOWN ETNK2
IQGAP2 -1.480694699 6.768693221 -10.8174418 1.97E-21 6.15E-20
37.76340672 DOWN IQGAP2
SLC25A15 -2.273277546 4.202638611-10.81494103 2.01E-21 6.24E-20
37.85335391 DOWN SLC25A15
PC -2.045519734 7.310054745 -10.81445081 2.01E-21 6.26E-20
37.74593565 DOWN PC
MSMO1 -1.722187939 7.46328773 -10.812243 2.04E-21 6.34E-20
37.73122842 DOWN MSMO1
ACSM5P1 -2.921156397 -1.657533412 -10.79381341 2.31E-21 7.16E-20 37.90214906 DOWN ACSM5P1
RP11_513G11.3 -3.760863793 -1.017794702 -10.79351288 2.32E-21
7.17E-20 37.90698525 DOWN RP11-513G11.3
HDAC6 -1.116990461 6.027226711-10.78667705 2.42E-21 7.48E-20
37.56280908 DOWN HDAC6
DLX4 3.080388499 -3.285664957 10.783110532.48E-21 7.64E-20
37.83400443 UP DLX4
CFHR3 -4.408205722 4.734420863 -10.77799469 2.57E-21 7.89E-20
37.64881846 DOWN CFHR3
RP3_420J14.1 -3.459700361 -3.235773181 -10.77578926 2.61E-21
7.99E-20 37.77068785 DOWN RP3-420J14.1
AASS -3.085578443 2.741420809 -10.77572877 2.61E-21 7.99E-20
37.72272953 DOWN AASS
LZTS2 1.045574845 4.784501847 10.77487379 2.62E-21 8.02E-20

37.51269683 UP LZTS2
CTD_2619J13.27 -2.001106082 -0.533925984 -10.77333865 2.65E-21 8.09E-20 37.78142796 DOWN CTD-2619J13.27
DNAJB9 -1.245334832 6.082484362 -10.77249571 2.66E-21 8.12E-20 37.46891475 DOWN DNAJB9
NKX3_2 2.939069312 -4.084693969 10.77240176 2.67E-21 8.12E-20 37.7537055 UP NKX3-2
DBT -1.10966539 4.764445687 -10.75561541 2.98E-21 9.06E-20 37.39363832 DOWN DBT
PNPLA7 -1.573338813 3.035111606 -10.75045409 3.09E-21 9.37E-20 37.500358 DOWN PNPLA7
PGM1 -1.331689168 7.011786303 -10.74934624 3.11E-21 9.42E-20 37.312009 DOWN PGM1
IMPDH1 1.743952327 3.147191798 10.74656793 3.17E-21 9.59E-20 37.43245956 UP IMPDH1
C21orf58 1.291173037 1.253274499 10.7383275 3.35E-21 1.01E-19 37.50382615 UP C21orf58
LDLRAD4_AS1 -3.038730756 -4.376193098 -10.72867652 3.57E-21 1.08E-19 37.45728126 DOWN LDLRAD4-AS1
SEPP1 -1.920393711 8.715455059 -10.71964085 3.79E-21 1.14E-19 37.13206616 DOWN SEPP1
CKS2 1.410317769 3.622911962 10.7189899 3.81E-21 1.14E-19 37.21324143 UP CKS2
MXD3 1.28642302 2.648846011 10.71413625 3.93E-21 1.18E-19 37.25393506 UP MXD3
GPHN -1.59767444 4.329948842 -10.71384434 3.94E-21 1.18E-19 37.15346596 DOWN GPHN
IL13RA1 -1.071518143 7.050319645 -10.71226685 3.98E-21 1.19E-19 37.06640532 DOWN IL13RA1
CYP4F11 -2.832058404 5.655909444 -10.70953418 4.05E-21 1.21E-19 37.08441926 DOWN CYP4F11
HIGD1A -1.202838089 5.779188409 -10.70945869 4.06E-21 1.21E-19 37.05593315 DOWN HIGD1A

HSF2BP 1.852803415 -2.066564723 10.707991194.10E-21 1.22E-19 37.34848567 UP HSF2BP
LPCAT1 1.631356834 4.761030548 10.70449076 4.19E-21 1.25E-19 37.05374285 UP LPCAT1
TMEM44 1.1873112522.284954463 10.70372608 4.21E-21 1.25E-19 37.21284157 UP TMEM44
MEX3A1.955534285 2.07979101110.69924763 4.34E-21 1.29E-19 37.1940795 UP MEX3A
STEAP4 -2.930992103 2.72021614 -10.69782146 4.38E-21 1.30E-19 37.20586602 DOWN STEAP4
PBLD -2.131980625 5.819025288 -10.69311282 4.52E-21 1.34E-19 36.95719603 DOWN PBLD
RP3_414A15.10 -1.879572735 -0.955976921 -10.69160747 4.57E-21 1.35E-19 37.2442402 DOWN RP3-414A15.10
FBXO43 1.936599027 -0.705157884 10.68032741 4.92E-21 1.46E-19 37.17171725 UP FBXO43
RP11_295M18.6 -2.437822347 -2.913945746 -10.67882929 4.97E-21 1.47E-19 37.13847387 DOWN RP11-295M18.6
CPN2 -3.547083365 6.780132595 -10.67515575 5.10E-21 1.50E-19 36.8393328 DOWN CPN2
SLC35D1 -1.369714013 5.749668838 -10.67478456 5.11E-21 1.50E-19 36.8287521 DOWN SLC35D1
MOCS1 -1.292472346 5.178236829 -10.67099174 5.24E-21 1.54E-19 36.81860417 DOWN MOCS1
MT_CYB -1.398854131 12.42623708 -10.66718215 5.37E-21 1.58E-19 36.86596642 DOWN MT-CYB
DNMT11.172915098 4.775658563 10.66592183 5.42E-21 1.59E-19 36.79357656 UP DNMT1
BHMT -4.167166096 6.238557955 -10.66297116 5.53E-21 1.62E-19 36.78808497 DOWN BHMT
GNMT -3.793955158 4.196517071 -10.66018959 5.63E-21 1.65E-19 36.88575833 DOWN GNMT
PACRG -2.815144112 -0.035209829 -10.65916922 5.67E-21 1.66E-19

37.03451431 DOWN PACRG

HRG -4.380007014 8.948857348 -10.65914339 5.67E-21 1.66E-19

36.73862428 DOWN HRG

AZGP1P1 -3.321466243 2.067581028 -10.65794437 5.71E-21 1.67E-

19 36.9825037 DOWN AZGP1P1

APOC3 -3.784443999 10.57176181 -10.65659877 5.76E-21 1.68E-19

36.74728991 DOWN APOC3

PQLC1 -1.253982599 6.900507983 -10.65319517 5.90E-21 1.72E-19

36.67581166DOWN PQLC1

HPDL 2.645069621 -2.414561539 10.65281246 5.91E-21 1.72E-19

36.98888957 UP HPDL

RP11_434D9.1 -4.128098981 -0.219692479 -10.65268501 5.92E-21

1.72E-19 36.99184294 DOWN RP11-434D9.1

APOF -4.634439903 4.003815473 -10.65179118 5.95E-21 1.73E-19

36.86850171 DOWN APOF

C1RL -1.324833258 6.683150974 -10.65113026 5.98E-21 1.73E-19

36.66245209 DOWN C1RL

PLAGL2 1.074203003 3.079133081 10.65055551 6.00E-21 1.74E-

19 36.8010903 UP PLAGL2

RP11_736K20.5 -1.831234513 -0.642805786 -10.64915963 6.06E-21

1.75E-19 36.96895316 DOWN RP11-736K20.5

TMEM51 1.943869375 2.317403484 10.64865898 6.08E-21 1.75E-

19 36.84521992 UP TMEM51

C10orf91 3.447570941 -4.223712826 10.63799969 6.52E-21 1.88E-

19 36.88021334 UP C10orf91

LPCAT4 1.509406875 2.392229494 10.63000675 6.88E-21 1.98E-

19 36.71797107 UP LPCAT4

HN1 1.302540864 5.15661546 10.62542559 7.09E-21 2.03E-19

36.51376473 UP HN1

UGT1A2P -4.825171061 -1.602301335 -10.62433356 7.14E-21 2.05E-

19 36.80020009 DOWN UGT1A2P

RP11_166D19.1 -1.98971868 1.012014848 -10.62174892 7.27E-21 2.08E-

19 36.7716675 DOWN RP11-166D19.1

AK3	-1.181697961	6.657319406	-10.62123414	7.29E-21	2.08E-19	36.46468369	DOWN	AK3
GLYCTK	-1.718300978	6.727849951	-10.61847691	7.43E-21	2.12E-19	36.44818702	DOWN	GLYCTK
NDRG2	-1.341433899	7.087484167	-10.61717653	7.49E-21	2.14E-19	36.43853405	DOWN	NDRG2
LPAR2	2.291266129	1.300073749	10.61625675	7.54E-21	2.15E-19	36.69185739	UP	LPAR2
THNSL1	-1.132724921	3.724513013	-10.61229725	7.74E-21	2.20E-19	36.51826449	DOWN	THNSL1
NOP56	1.029546904	5.886904501	10.61030747	7.84E-21	2.23E-19	36.39825927	UP	NOP56
SLC51A	-3.109162302	4.946120225	-10.60947587	7.89E-21	2.24E-19	36.46831449	DOWN	SLC51A
ABCG2	-2.668149548	3.691581464	-10.60900188	7.91E-21	2.24E-19	36.54647129	DOWN	ABCG2
FETUB	-4.341363785	4.286744109	-10.60771212	7.98E-21	2.26E-19	36.55177447	DOWN	FETUB
MFAP3L	-2.917461753	3.107662493	-10.60085368	8.35E-21	2.36E-19	36.54273259	DOWN	MFAP3L
KNG1	-3.225294542	10.12765294	-10.59941211	8.43E-21	2.38E-19	36.36150305	DOWN	KNG1
CTD_3193O13.1	-2.734569079	-2.397155742	-10.59928505	8.44E-21	2.38E-19	36.62675403	DOWN	CTD-3193O13.1
ZFAND5	-1.452562882	7.35393254	-10.58743298	9.13E-21	2.57E-19	36.24361766	DOWN	ZFAND5
PIGV	-1.06610085	4.127586298	-10.58681516	9.17E-21	2.58E-19	36.31671778	DOWN	PIGV
KLC4	-1.095893388	6.222221018	-10.5854186	9.25E-21	2.60E-19	36.23024481	DOWN	KLC4
GHR	-2.582714641	4.746885803	-10.58440209	9.31E-21	2.61E-19	36.30373179	DOWN	GHR
ACOT12	-3.641995357	3.652378121	-10.58312693	9.39E-21	2.63E-			

19 36.40986123 DOWN ACOT12

CYP2B6 -3.930056427 4.976625066 -10.57589032 9.86E-21 2.75E-19 36.27591169DOWN CYP2B6

FOXJ1 4.27305314 -1.613732537 10.56620957 1.05E-20 2.93E-19 36.42688897 UP FOXJ1

TMEM176B -1.921946179 9.572170067 -10.56462141 1.06E-20 2.96E-19 36.12344776 DOWN TMEM176B

HSDL2 -1.26887629 6.745354855 -10.56043954 1.09E-20 3.04E-19 36.06371648 DOWN HSDL2

G6PC -3.704383434 7.579896182 -10.55721952 1.12E-20 3.10E-19 36.05582638 DOWN G6PC

MYRIP -3.621004675 3.21703452 -10.55553825 1.13E-20 3.13E-19 36.25593559 DOWN MYRIP

SLC39A10 1.232486538 2.934076501 10.54843197 1.18E-20 3.27E-19 36.14013565 UP SLC39A10

LINC01537 -1.812687574 -2.425869132 -10.54450705 1.21E-20 3.35E-19 36.26861444 DOWN LINC01537

CDK4 1.027217234 5.10731456 10.54360007 1.22E-20 3.37E-19 35.97364116UP CDK4

BAATP1 -2.797367578 -2.044059797 -10.54337421 1.22E-20 3.37E-19 36.26659163 DOWN BAATP1

GINS4 1.735290769 0.476430627 10.529491121.34E-20 3.69E-19 36.16202232 UP GINS4

C15orf39 1.138343478 4.101048124 10.52752217 1.36E-20 3.74E-19 35.91563787 UP C15orf39

MGST2 -1.180149226 6.477009704 -10.52175729 1.41E-20 3.87E-19 35.80927875 DOWN MGST2

SLCO1B1 -3.368801718 5.626152618 -10.51924722 1.44E-20 3.93E-19 35.84434855 DOWN SLCO1B1

CA5A -3.748735429 2.837483573 -10.51768102 1.45E-20 3.97E-19 36.02964346 DOWN CA5A

ANGPTL6 -2.061016162 1.147950406 -10.51277712 1.50E-20 4.10E-19 36.05415264 DOWN ANGPTL6

RP11_1069G10.1	-3.11784464	0.493719921	-10.50597584	1.57E-20	4.28E-19	36.03033318	DOWN	RP11-1069G10.1
ZBTB12	1.651914832	1.06362123	10.50549829	1.57E-20	4.29E-19	35.98155119	UP	ZBTB12
AIG1	-1.153742838	6.670924324	-10.49541449	1.68E-20	4.57E-19	35.63517566	DOWN	AIG1
SYBU	-2.249603699	4.969182014	-10.49380995	1.70E-20	4.60E-19	35.68034101	DOWN	SYBU
ALDH4A1	-1.70281435	7.739368631	-10.48393451	1.81E-20	4.91E-19	35.56484746	DOWN	ALDH4A1
ZCCHC24	-1.112809002	4.897119275	-10.47924444	1.87E-20	5.05E-19	35.56349552	DOWN	ZCCHC24
ZNF519	1.719448513	-0.445790661	10.47907993	1.87E-20	5.05E-19	35.85438532	UP	ZNF519
NSUN6	-1.055844716	4.22227524	-10.47220443	1.96E-20	5.27E-19	35.55533881	DOWN	NSUN6
SH3D19	-1.136204207	5.682919406	-10.46707617	2.03E-20	5.45E-19	35.45863333	DOWN	SH3D19
NT5DC4	2.252345268	-4.138249226	10.45962388	2.13E-20	5.71E-19	35.71526675	UP	NT5DC4
C4BPA	-3.352076283	9.032275394	-10.45864151	2.14E-20	5.74E-19	35.41555501	DOWN	C4BPA
RP11_35N6.6	-2.571539417	0.383614097	-10.45584212	2.18E-20	5.84E-19	35.70301597	DOWN	RP11-35N6.6
DPYS	-3.74079804	6.472126854	-10.44812851	2.30E-20	6.14E-19	35.35367628	DOWN	DPYS
GPRIN1	1.991135737	1.04057553	10.44625624	2.33E-20	6.21E-19	35.59250098	UP	GPRIN1
GFRA1	-4.272230602	3.341700114	-10.44261558	2.38E-20	6.35E-19	35.52705109	DOWN	GFRA1
BX842568.1	-3.156561069	-3.545201754	-10.44036889	2.42E-20	6.44E-19	35.58851405	DOWN	BX842568.1
GSTZ1	-1.952897682	4.79501238	-10.43949513	2.43E-20	6.47E-19			

35.32394988 DOWN GSTZ1
HMMR 1.809032067 2.003278816 10.43281271 2.54E-20 6.75E-19 35.44906641 UP HMMR
PTGES3L 1.887738163 -2.860120226 10.42631234 2.66E-20 7.04E-19 35.50941637 UP PTGES3L
AADAC -3.189804469 6.900165998 -10.42388122 2.70E-20 7.15E-19 35.17733943 DOWN AADAC
HID1 1.859391449 3.220477831 10.42255646 2.72E-20 7.21E-19 35.29469037 UP HID1
CDH23 -2.020066724 3.034533524 -10.42077261 2.76E-20 7.28E-19 35.3409045 DOWN CDH23
EIF4HP2 1.735549679 -2.708939892 10.41991989 2.77E-20 7.32E-19 35.46848426 UP EIF4HP2
RP11_165F24.2 2.100098527 -4.779167968 10.41848487 2.80E-20 7.38E-19 35.44500488 UP RP11-165F24.2
MYEF2 3.127422509 -0.276607606 10.41632392 2.84E-20 7.48E-19 35.43764968 UP MYEF2
DHRS4L1 -1.987544704 -0.596590433 -10.41608767 2.84E-20 7.48E-19 35.45058963 DOWN DHRS4L1
RP11_178L8.4 -2.35983906 -3.411198042 -10.4147291 2.87E-20 7.54E-19 35.42143217 DOWN RP11-178L8.4
MMP10 3.534445715 -2.8431814 10.41060227 2.95E-20 7.74E-19 35.41331367 UP MMP10
TPM3P9 1.561441515 1.503526929 10.40881915 2.98E-20 7.82E-19 35.32568508 UP TPM3P9
PALMD -1.82272169 4.586072242 -10.40527924 3.05E-20 8.00E-19 35.10911107 DOWN PALMD
MTHFS -1.465609667 3.825897091 -10.39929979 3.18E-20 8.31E-19 35.11832746 DOWN MTHFS
FAM47E_STBD1 -1.451193198 3.445431933 -10.39674342 3.23E-20 8.44E-19 35.13406806 DOWN FAM47E-STBD1
LHFPL3 2.758965421 -4.835217898 10.39315841 3.31E-20 8.64E-19 35.28304584 UP LHFPL3

SUGCT -1.591794156 3.79753305 -10.38690493 3.45E-20 8.99E-19 35.04187413 DOWN SUGCT
RNF125 -2.078551109 2.587887704 -10.38554546 3.48E-20 9.06E-19 35.14686512 DOWN RNF125
RP11_809O17.1 2.571481665 -2.447426437 10.37709917 3.68E-20 9.57E-19 35.19560167 UP RP11-809O17.1
SLC2A2 -3.273201061 7.388061795 -10.37644914 3.69E-20 9.60E-19 34.86350761 DOWN SLC2A2
SLC4A11 2.807090402 -0.582236683 10.35828025 4.17E-20 1.08E-18 35.06692254 UP SLC4A11
RANBP3L -3.321344583 -0.967275042 -10.35636847 4.22E-20 1.09E-18 35.06151086 DOWN RANBP3L
FAM57A 1.521662019 1.846101429 10.35168707 4.35E-20 1.13E-18 34.9306002 UP FAM57A
FER1L4 2.920346365 -0.276848982 10.34257798 4.62E-20 1.19E-18 34.95784616 UP FER1L4
PIK3CD_AS2 2.761885699 -2.337880567 10.3352736 4.85E-20 1.25E-18 34.92539982 UP PIK3CD-AS2
EVC2 2.646803242 -0.163110543 10.33266652 4.93E-20 1.27E-18 34.89202002 UP EVC2
RP5_1074L1.4 1.307448893 0.840678625 10.32347034 5.24E-20 1.35E-18 34.80528425 UP RP5-1074L1.4
DZIP1L 2.250740644 -0.376254031 10.32319117 5.25E-20 1.35E-18 34.8369608 UP DZIP1L
LINC00238 -3.891374756 -3.031869018 -10.32090905 5.33E-20 1.37E-18 34.8216342 DOWN LINC00238
RP9P 1.19987138 0.848742787 10.31672509 5.48E-20 1.41E-18 34.76160783 UP RP9P
RP11_290F5.1 -3.096177765 1.515529699 -10.31247576 5.64E-20 1.45E-18 34.74296527 DOWN RP11-290F5.1
IGFBP4 -1.427257878 9.358843688 -10.31215133 5.65E-20 1.45E-18 34.46156942 DOWN IGFBP4
ROBO4 -1.256551605 3.974493077 -10.30862256 5.78E-20 1.48E-18

34.50591592 DOWN ROBO4

SLC25A25 -1.798083762 5.625156841 -10.30108798 6.08E-20 1.55E-18 34.37823271 DOWN SLC25A25

COLCA2 2.659521883 0.281841801 10.29930345 6.15E-20 1.57E-18 34.66143936 UP COLCA2

SELO -1.145797282 5.801348349 -10.29657176 6.26E-20 1.60E-18 34.33718704 DOWN SELO

ARID3A 2.423047238 2.870762158 10.28954671 6.56E-20 1.67E-18 34.45436451 UP ARID3A

VWA8 -1.24179499 4.670433492 -10.28195297 6.89E-20 1.75E-18 34.28316838 DOWN VWA8

SMUG1P1 -3.611302494 -4.216686746 -10.27866073 7.05E-20 1.79E-18 34.54296309 DOWN SMUG1P1

TNNT1 3.537984772 -2.725028887 10.27716259 7.12E-20 1.81E-18 34.54942498 UP TNNT1

NFIC -1.152871226 7.012266567 -10.27474802 7.23E-20 1.83E-18 34.18685202 DOWN NFIC

PRAME 5.91264926 -1.875723188 10.27062668 7.43E-20 1.88E-18 34.49880088 UP PRAME

CHTF18 1.17062781 2.87473334 10.27056356 7.43E-20 1.88E-18 34.32346742 UP CHTF18

P2RX4 1.138866511 4.462234724 10.26120552 7.90E-20 2.00E-18 34.14705633 UP P2RX4

CFL2 -1.209463055 5.688908576 -10.25909838 8.02E-20 2.02E-18 34.09468098 DOWN CFL2

FAM117B 1.343107932 2.345135992 10.25825409 8.06E-20 2.03E-18 34.28525026 UP FAM117B

ADH6 -2.738646128 6.857835513 -10.25751105 8.10E-20 2.04E-18 34.08190926 DOWN ADH6

MIA3 -1.103358028 6.769960801 -10.25651007 8.15E-20 2.05E-18 34.06706135 DOWN MIA3

TMEM65 1.34326385 3.698028425 10.24969854 8.53E-20 2.14E-18 34.12267801 UP TMEM65

CD302 -1.651593893 4.132419467 -10.24633491 8.72E-20 2.19E-18 34.09586416 DOWN CD302

DECR2 -1.381200185 6.230877597 -10.24582347 8.75E-20 2.19E-18 34.00066533 DOWN DECR2

DDI2 -1.029091497 5.584114648-10.24482245 8.81E-20 2.21E-18 34.00205556 DOWN DDI2

CTC_260E6.4 2.547814056 -2.299522656 10.24212844 8.96E-20 2.24E-18 34.32259391 UP CTC-260E6.4

FUOM -1.782431617 5.84019321 -10.24118511 9.02E-20 2.25E-18 33.9796517 DOWN FUOM

UBXN10 -3.686341213 2.318649902 -10.23975039 9.11E-20 2.27E-18 34.24215369 DOWN UBXN10

AC118754.4-2.490581125 -3.569078027 -10.23801952 9.21E-20 2.30E-18 34.28079687 DOWN AC118754.4

INCENP 1.241770723 2.658900967 10.23517735 9.38E-20 2.34E-18 34.11006398UP INCENP

PLEKHG2 1.198130138 3.580240165 10.23141361 9.62E-20 2.39E-18 34.01110348UP PLEKHG2

AKR7L -2.219771037 2.037786712 -10.22483675 1.00E-19 2.49E-18 34.13703859 DOWN AKR7L

RP11_116B13.1 -2.331613681 -3.708686315 -10.21302435 1.09E-19 2.69E-18 34.11927793DOWN RP11-116B13.1

ZNF14 1.732636559 0.372867421 10.19633169 1.21E-19 2.99E-18 33.99454575 UP ZNF14

SLC6A1 -2.777446224 5.722626387 -10.19137004 1.25E-19 3.09E-18 33.67611983DOWN SLC6A1

GPR19 1.842496785 -2.125462583 10.19130747 1.25E-19 3.09E-18 33.99408541 UP GPR19

RP5_967N21.11 1.693899138 -0.739585536 10.188886111.27E-19 3.13E-18 33.97345918 UP RP5-967N21.11

C6orf223 4.529766622 -1.780653247 10.18826806 1.28E-19 3.14E-18 33.97146194 UP C6orf223

SOCS2 -2.026065744 3.426303977 -10.18770448 1.28E-19 3.15E-18

33.78810949 DOWN SOCS2

AGXT -3.705113992 8.921909184 -10.18756393 1.28E-19 3.15E-18 33.638411 DOWN AGXT

DDX39A 1.0527811425.399585596 10.1800329 1.35E-19 3.30E-18 33.58129109 UP DDX39A

SERPINA10-2.491749669 7.145498061 -10.1701843 1.44E-19 3.52E-18 33.50823742 DOWN SERPINA10

LINC01121 2.730481751 -3.640841675 10.16904268 1.45E-19 3.54E-18 33.84563108 UP LINC01121

MYLK22.046110678-3.431579396 10.16421037 1.50E-19 3.65E-18 33.81306973 UP MYLK2

SRC 1.752099962 4.307881576 10.15825222 1.56E-19 3.80E-18 33.49114288UP SRC

NEURL3 3.149277632 0.76511756810.15780937 1.56E-19 3.80E-18 33.71974616 UP NEURL3

SLC22A25 -3.194674753 2.284518182 -10.15729004 1.57E-19 3.81E-18 33.69928305 DOWN SLC22A25

AC004538.3-3.381186073 -2.274687031 -10.15609365 1.58E-19 3.84E-18 33.7621919 DOWN AC004538.3

LIN9 1.233526354 1.485676076 10.15541591 1.59E-19 3.85E-18 33.67801593 UP LIN9

UGT2B7 -3.387963665 6.651532822 -10.15488864 1.59E-19 3.86E-18 33.42225817 DOWN UGT2B7

C1orf106 3.771696626 0.246716624 10.15246048 1.62E-19 3.92E-18 33.70392187 UP C1orf106

RP11_293M10.6-1.974952862 -0.288570005 -10.14560242 1.69E-19 4.09E-18 33.69677828 DOWN RP11-293M10.6

PNPO -1.401927312 5.420473156 -10.14391023 1.71E-19 4.13E-18 33.35124783 DOWN PNPO

SLC1A2 -4.477079179 3.995620679 -10.13878772 1.77E-19 4.27E-18 33.50704596 DOWN SLC1A2

CES1 -3.10788282 9.804863907 -10.13608219 1.80E-19 4.34E-18 33.31646357 DOWN CES1

PYCR1 3.03999415 2.994874366 10.13600949 1.80E-19 4.34E-18 33.45303187 UP PYCR1

GPD2 1.016738479 3.23060111 10.13437366 1.82E-19 4.38E-18 33.40552777 UP GPD2

RP11_73M7.1 2.363149995 -4.035051527 10.13140893 1.86E-19 4.46E-18 33.59941187UP RP11-73M7.1

SLC38A4 -3.078559063 6.964057037 -10.12895462 1.89E-19 4.53E-18 33.24499336 DOWN SLC38A4

GTF2IRD2 -1.103030997 0.767943035 -10.12748732 1.91E-19 4.57E-18 33.5531551 DOWN GTF2IRD2

DHRS4 -1.222024397 4.389725635 -10.12679602 1.91E-19 4.59E-18 33.28598677 DOWN DHRS4

MOGAT2 -5.421897571 2.394226829 -10.12226011 1.97E-19 4.72E-18 33.50314301 DOWN MOGAT2

SDC1 -1.275804671 9.305786323 -10.11977245 2.00E-19 4.79E-18 33.2039566 DOWN SDC1

C19orf40 1.0711916561.021302361 10.1151642 2.07E-19 4.93E-18 33.4447078 UP C19orf40

FER1L52.100144767 -3.334401397 10.10844998 2.16E-19 5.14E-18 33.45549439 UP FER1L5

MARCKSL1 1.488520535 5.72116483210.10790881 2.17E-19 5.15E-18 33.10809444 UP MARCKSL1

KLC3 3.318569431 -2.799922602 10.10789395 2.17E-19 5.15E-18 33.45677821 UP KLC3

CREG2 2.263774206 -2.940187815 10.1062756 2.19E-19 5.20E-18 33.4441527 UP CREG2

PHYH -1.58137558 7.460572926 -10.10329523 2.23E-19 5.29E-18 33.06982562 DOWN PHYH

FAM227A 2.179628885 -2.404511149 10.10154887 2.26E-19 5.35E-18 33.41545083 UP FAM227A

FHDC1 2.048641801 -0.304966297 10.10149707 2.26E-19 5.35E-18 33.39927605 UP FHDC1

PAPSS2 -1.557094032 5.622807237 -10.08933913 2.45E-19 5.79E-18

32.991843	DOWN PAPSS2
RBKS	-1.193908269	4.124007742	-10.08769952	2.47E-19	5.84E-18
33.05039276	DOWN RBKS
RP11_800A18.4 3.163079021	-4.250106895	10.08505364	2.52E-19
5.94E-18	33.30381518	UP RP11-800A18.4
MTHFD1L 1.244025139	3.266027029	10.08246137	2.56E-19	6.03E-18	33.06551329	UP MTHFD1L
BLMH 1.592974966	4.228350628	10.0798029 2.61E-19	6.13E-18
32.98225614	UP BLMH
DSCC1 1.281417031	1.475109178	10.07871707	2.62E-19	6.17E-18
33.18032724	UP DSCC1
NUGGC	-3.281534122	2.302358648	-10.07870234	2.62E-19	6.17E-18	33.19005928	DOWN NUGGC
KIAA0922 -1.124165058	4.389643124	-10.07606828	2.67E-19	6.27E-18	32.95453426	DOWN KIAA0922
FGA	-2.979328879	12.53282523	-10.06834913	2.81E-19	6.59E-18
32.93842645	DOWN FGA
CHPT1 -1.092248783	6.509243781	-10.06684731	2.84E-19	6.65E-18
32.82974084	DOWN CHPT1
CTD_2231E14.2 1.89815745 -2.940036846	10.06327837	2.90E-19	6.80E-18	33.1668864 UP CTD-2231E14.2
CTSF	-1.607738938	6.495383661	-10.05898711	2.99E-19	6.98E-18
32.78077696	DOWN CTSF
RP11_564D11.3 2.317289111-3.951781316	10.04953136	3.18E-19	7.41E-18	33.07425675	UP RP11-564D11.3
TFAP2E1.970941101-2.135493246	10.0470883 3.23E-19	7.52E-18
33.06567203	UP TFAP2E
PRR7_AS1 1.945640282	-3.4645782 10.047044	3.23E-19	7.52E-18
33.06001843	UP PRR7-AS1
MCIDAS	2.485012059	-4.783854391	10.04570643	3.26E-19	7.57E-18	33.04634163	UP MCIDAS
ACY3	-2.324433809	3.40685523 -10.04262441	3.33E-19	7.72E-18
32.85508207	DOWN ACY3

GAL3ST1 3.699589326 1.990397688 10.04161673 3.35E-19 7.77E-18 32.90385292 UP GAL3ST1
RAB42 1.869573206 -0.250684515 10.03147929 3.58E-19 8.29E-18 32.94651534 UP RAB42
CENPU 1.324380267 2.419334584 10.02273428 3.79E-19 8.76E-18 32.74759262 UP CENPU
TPRG1 -2.144606867 1.354943798 -10.02240101 3.80E-19 8.77E-18 32.85880581 DOWN TPRG1
SLC16A3 2.082449103 3.55054029 10.00618464 4.22E-19 9.73E-18 32.55680756 UP SLC16A3
RP11_164O23.8 -2.416580554 -2.523548497 -9.999821334 4.40E-19 1.01E-17 32.75652081 DOWN RP11-164O23.8
KAT2B -1.37118708 4.626872002 -9.99124202 4.66E-19 1.07E-17 32.39356694 DOWN KAT2B
RP11_115C10.1 -5.005603678 -1.21193211 -9.990193788 4.69E-19 1.08E-17 32.70035485 DOWN RP11-115C10.1
ASNS 2.252728954 2.224702314 9.989769885 4.70E-19 1.08E-17 32.54709738 UP ASNS
DRD1 -3.479869446 -2.205576546 -9.983663148 4.89E-19 1.12E-17 32.65571787 DOWN DRD1
OSBPL3 1.337312269 2.640443457 9.977355374 5.10E-19 1.17E-17 32.4353286 UP OSBPL3
LAPTM4B 1.826217918 6.096128027 9.973940741 5.21E-19 1.19E-17 32.23546788 UP LAPTM4B
MYO16 -2.398537779 0.499542625 -9.964830822 5.53E-19 1.26E-17 32.51919941 DOWN MYO16
ACADSB -1.81230901 7.065037914 -9.960523679 5.69E-19 1.30E-17 32.14045649 DOWN ACADSB
MACROD1 -1.579954779 4.228741077 -9.960400909 5.70E-19 1.30E-17 32.22520009 DOWN MACROD1
RASSF3 1.317845241 4.044029136 9.957277044 5.81E-19 1.32E-17 32.19467659 UP RASSF3
ACRV1 1.982730628 -3.877531653 9.956582101 5.84E-19 1.33E-17

32.4784774 UP ACRV1

NUDT6 -1.779522021 1.631004381 -9.947940736 6.18E-19 1.40E-17

32.36037635 DOWN NUDT6

RP11_250B2.6 -1.479529513 -0.435542453 -9.944147755 6.33E-19

1.44E-17 32.39993878 DOWN RP11-250B2.6

ATP6V0E2 -1.734838345 6.078024706 -9.943879618 6.35E-19 1.44E-

17 32.03859388 DOWN ATP6V0E2

PPARA -1.298734408 5.932493855 -9.941443415 6.45E-19 1.46E-17

32.02152636 DOWN PPARA

SLC7A1 2.164860492 2.429759389 9.936369303 6.67E-19 1.51E-

17 32.18669088 UP SLC7A1

NLRP6 -3.038944589 0.365689241 -9.93611278 6.68E-19 1.51E-17

32.34059101 DOWN NLRP6

SLC25A42 -1.65620515 5.78881044 -9.93395357 6.77E-19 1.53E-17

31.9789395 DOWN SLC25A42

RP11_757F18.5 2.813104674 -4.44625206 9.929305744 6.98E-19 1.57E-

17 32.3039323 UP RP11-757F18.5

HLX -1.249584794 3.662884913 -9.927152668 7.08E-19 1.59E-17

32.04954272 DOWN HLX

NNT -1.326674059 6.718980882 -9.917898707 7.52E-19 1.69E-17

31.86249614 DOWN NNT

LINC00844 -5.839876554 0.314128351 -9.911705386 7.83E-19 1.76E-

17 32.19285742 DOWN LINC00844

FRAS1 4.21900121 0.862114223 9.909520183 7.94E-19 1.78E-17

32.10655062 UP FRAS1

TMEM194A 1.033099964 3.236135265 9.908396306 8.00E-19 1.79E-

17 31.93841396 UP TMEM194A

SSTR3 2.955417039 -3.944554599 9.904778793 8.19E-19 1.83E-17

32.15028617 UP SSTR3

CADM1 -1.631067816 5.956775113 -9.900983362 8.40E-19 1.88E-17

31.76183385 DOWN CADM1

ZNF320 2.241663047 2.003860323 9.900973974 8.40E-19 1.88E-17

31.98837891 UP ZNF320

LINC00402	-2.891566977	-3.41735828	-9.897406419	8.60E-19	1.92E-17	32.09888797	DOWN	LINC00402
CBR1	-1.627965212	7.308159603	-9.896963655	8.62E-19	1.92E-17	31.72865838	DOWN	CBR1
GADD45B	-1.658207357	6.419220908	-9.894528039	8.76E-19	1.95E-17	31.71406192	DOWN	GADD45B
SIAE	-1.488951125	5.191238325	-9.894287562	8.77E-19	1.95E-17	31.73883014	DOWN	SIAE
STARD10	-1.321778325	7.945172608	-9.893788454	8.80E-19	1.96E-17	31.7134899	DOWN	STARD10
GNG12_AS1	-1.812069747	-1.633496947	-9.893279659	8.83E-19	1.96E-17	32.0789983	DOWN	GNG12-AS1
RP11_1182P23.5	-3.066314348	-2.413580236	-9.89318056	8.84E-19	1.96E-17	32.07598043	DOWN	RP11-1182P23.5
MMRN2	-1.159935279	4.225801415	-9.889986097	9.02E-19	2.00E-17	31.75935667	DOWN	MMRN2
HNRNPA1P16	1.315902129	-1.348020154	9.887939926	9.14E-19	2.03E-17	32.04322404	UP	HNRNPA1P16
ESPL1	1.906160721	2.460516869	9.884967232	9.32E-19	2.06E-17	31.85178413	UP	ESPL1
RP11_116D2.1	-2.823597155	4.627637035	-9.884189974	9.37E-19	2.07E-17	31.74138435	DOWN	RP11-116D2.1
TRNP1	2.952441381	2.625625922	9.881345511	9.55E-19	2.11E-17	31.82361767	UP	TRNP1
TK1	1.495579419	4.286632109	9.881010218	9.57E-19	2.11E-17	31.68752667	UP	TK1
LEAP2	-2.621776446	5.782693417	-9.877080296	9.82E-19	2.16E-17	31.62567732	DOWN	LEAP2
ABCC9	-2.581159328	3.565399565	-9.876704863	9.84E-19	2.17E-17	31.77607955	DOWN	ABCC9
ADRA1A	-4.0278229	0.526114802	-9.874094021	1.00E-18	2.20E-17	31.94386932	DOWN	ADRA1A
SRL	-1.586099499	-1.601531404	-9.870145911	1.03E-18	2.26E-17			

31.9313555 DOWN SRL

AC010761.8 1.333741718 -1.07357838 9.86578554 1.06E-18 2.32E-17

31.89856276 UP AC010761.8

PSRC1 1.614241317 1.716327626 9.865431807 1.06E-18 2.32E-17

31.78252806 UP PSRC1

ZNF70 1.208948843 0.702605693 9.859635123 1.10E-18 2.41E-17

31.80941648 UP ZNF70

IGSF3 2.583339816 2.585460579 9.854695648 1.14E-18 2.48E-17

31.65018533 UP IGSF3

TTR -3.309939019 9.46042458 -9.854456947 1.14E-18 2.48E-17

31.48139157 DOWN TTR

RP1_159G19.1 -3.082351546 -3.634353868 -9.845503776 1.21E-18

2.63E-17 31.76768241 DOWN RP1-159G19.1

DLG2 -1.854066757 -0.228540478 -9.845213775 1.21E-18 2.63E-17

31.76379219 DOWN DLG2

RP11_90L1.8 -2.680717256 -1.260276607 -9.841460449 1.24E-18

2.69E-17 31.74835161 DOWN RP11-90L1.8

DTYMK 1.035857366 4.375942293 9.841035785 1.24E-18 2.69E-

17 31.41882843 UP DTYMK

USP30_AS1 -2.006642126 0.968935141 -9.835211394 1.29E-18 2.79E-

17 31.66811152 DOWN USP30-AS1

RNF144B -1.377185516 3.621459778 -9.833560645 1.30E-18 2.82E-

17 31.45214653 DOWN RNF144B

FAM225A 2.133641486 -3.084468135 9.831703421 1.32E-18 2.85E-

17 31.68514522 UP FAM225A

SNHG3 1.557049918 3.164504378 9.828355272 1.35E-18 2.91E-17

31.42981032 UP SNHG3

CTC_537E7.3 -2.756820454 -2.96988818 -9.826889205 1.36E-18 2.94E-

17 31.65085385 DOWN CTC-537E7.3

CSAD -1.600669101 4.537967965 -9.81961228 1.43E-18 3.07E-17

31.2933789 DOWN CSAD

TGFBR3L -2.832611758 0.037752376 -9.815006817 1.47E-18 3.16E-

17 31.5693593 DOWN TGFBR3L

NCK2 1.753733567 3.8672058 9.814360253 1.48E-18 3.16E-17 31.28795856 UP NCK2
RP11_84C13.2 -2.058982625 -1.811149843 -9.813784309 1.48E-18 3.17E-17 31.57096986 DOWN RP11-84C13.2
HACL1 -1.02269056 4.85274981 -9.811803591 1.50E-18 3.21E-17 31.21241427 DOWN HACL1
BX842568.2 -3.292264784 -1.647563843 -9.8113016 1.51E-18 3.22E-17 31.55559404 DOWN BX842568.2
BCYRN1 2.157593849 -2.201799585 9.811023992 1.51E-18 3.22E-17 31.55389011 UP BCYRN1
CFH -2.109661159 9.660894344 -9.808546594 1.53E-18 3.27E-17 31.18990365 DOWN CFH
GBP7 -3.702121237 2.653192608 -9.808265846 1.54E-18 3.27E-17 31.43360181 DOWN GBP7
GINS2 1.364561233 2.281913342 9.802926287 1.59E-18 3.38E-17 31.33824282 UP GINS2
TMEM136 1.516904326 1.146503583 9.802096648 1.60E-18 3.40E-17 31.41313213 UP TMEM136
GATM -2.072944427 9.055620803 -9.801894115 1.60E-18 3.40E-17 31.13540626 DOWN GATM
DDN 2.274914877 -4.013486997 9.798796806 1.63E-18 3.46E-17 31.47237491 UP DDN
VIPR1 -2.763662007 0.507155826 -9.798130493 1.64E-18 3.48E-17 31.45081112 DOWN VIPR1
PLEKHG4 2.558557298 0.181247653 9.796936844 1.65E-18 3.50E-17 31.42024914 UP PLEKHG4
ZNF496 1.610521722 3.438229718 9.796799521 1.66E-18 3.50E-17 31.20482584 UP ZNF496
SLC30A1 -1.42336685 6.234013415 -9.795930595 1.67E-18 3.52E-17 31.07690189 DOWN SLC30A1
E2F3 1.064619925 3.343012967 9.790003628 1.73E-18 3.64E-17 31.16483342 UP E2F3
DDR1 2.530967319 3.904430134 9.789401794 1.74E-18 3.66E-17

31.13781158UP DDR1

C11orf54 -1.266817608 6.093896101 -9.78892306 1.74E-18 3.66E-17

31.03229096 DOWN C11orf54

SORL1 -1.790470436 5.841780639 -9.788253237 1.75E-18 3.67E-17

31.03716551 DOWN SORL1

RP4_763G1.2 -4.382439102 0.377101585 -9.787810516 1.76E-18

3.68E-17 31.39567005 DOWN RP4-763G1.2

BMF 1.727993526 3.277487373 9.785361343 1.78E-18 3.74E-17

31.14511426UP BMF

PLCG2 -1.317100355 4.468351559 -9.780515497 1.84E-18 3.85E-17

31.03844645 DOWN PLCG2

CTB_186H2.2 -2.330866838 -3.920632845 -9.778685185 1.86E-18

3.90E-17 31.3414352 DOWN CTB-186H2.2

CYYR1 -1.239741041 2.784139848 -9.777708167 1.88E-18 3.91E-17

31.16813667 DOWN CYYR1

CCDC74B 2.452062164 -3.417278319 9.776924799 1.88E-18 3.93E-

17 31.33569352 UP CCDC74B

AUNIP 1.479778192 -0.939180352 9.769945221 1.97E-18 4.11E-17

31.28343899 UP AUNIP

CLSTN1 1.267975822 5.507318185 9.7695772 1.98E-18 4.12E-17

30.91662904 UP CLSTN1

ZNF711 2.582654716 -0.458156154 9.76870798 1.99E-18 4.13E-17

31.26086777 UP ZNF711

GPRC5D 1.685244994 -2.717681716 9.767395254 2.01E-18 4.17E-

17 31.27566854 UP GPRC5D

RP11_772C9.1 -2.573529547 -4.178245411 -9.765856322 2.03E-18

4.20E-17 31.2599451 DOWN RP11-772C9.1

KCNB1 -3.984223816 1.239796167 -9.760159414 2.10E-18 4.36E-17

31.19421701 DOWN KCNB1

SETD7 -1.110575663 6.002841249 -9.760117199 2.10E-18 4.36E-17

30.84674195 DOWN SETD7

SLC41A2 -1.595295638 5.008306968 -9.75929466 2.11E-18 4.38E-17

30.87735545 DOWN SLC41A2

FAAH -1.502002062 4.656415228 -9.747542456 2.28E-18 4.72E-17
30.81818052 DOWN FAAH
RP5_966M1.6 -2.077826479 4.243391579 -9.740063751 2.39E-18
4.95E-17 30.81625295 DOWN RP5-966M1.6
XDH -2.745886313 4.562550943 -9.737299031 2.44E-18 5.04E-17
30.80169353 DOWN XDH
ACSL5 -2.2197389 6.118689524-9.730993961 2.54E-18 5.23E-17
30.6672604 DOWN ACSL5
FZD4 -1.074187586 4.777822317 -9.730926836 2.54E-18 5.23E-17
30.69583386 DOWN FZD4
RNF152-1.410996172 4.529462903 -9.72668506 2.61E-18 5.37E-17
30.68975262 DOWN RNF152
BLVRB -1.233554367 7.047437701 -9.726153895 2.62E-18 5.38E-17
30.62366924 DOWN BLVRB
ACOT13 -1.040735086 5.634854612 -9.724436118 2.65E-18 5.44E-
17 30.62265963 DOWN ACOT13
RP11_284B18.3 -2.207443456 -3.613068156 -9.722859457 2.68E-18
5.49E-17 30.98729509 DOWN RP11-284B18.3
PECR -1.464294929 6.195328042 -9.722519136 2.68E-18 5.50E-17
30.60418317 DOWN PECR
STXBP4 1.10776008 2.529384215 9.721891655 2.69E-18 5.51E-17
30.79755899 UP STXBP4
RP4_539M6.21 -2.061079811 -3.1930911 -9.714791665 2.82E-18 5.75E-
17 30.93690873 DOWN RP4-539M6.21
SYNGR3 2.738669522 -2.143527054 9.7118699032.88E-18 5.85E-17
30.92094148 UP SYNGR3
SS18L1 -1.079814129 4.6360892 -9.710232931 2.91E-18 5.91E-17
30.57029587 DOWN SS18L1
ISOC1 -1.013043138 5.151718765 -9.703497233 3.04E-18 6.17E-17
30.50171006 DOWN ISOC1
RP11_418J17.3 -2.891523368 -2.565843691 -9.701108284 3.08E-18
6.26E-17 30.85271371 DOWN RP11-418J17.3
SPP2 -4.647343712 4.027858812 -9.700133072 3.10E-18 6.29E-17

30.67764683 DOWN SPP2

TSC22D3 -1.845292905 5.966439016 -9.694389172 3.22E-18 6.52E-17 30.43033366 DOWN TSC22D3

BOK -1.765976677 6.097716286 -9.69207285 3.27E-18 6.61E-17 30.41184415 DOWN BOK

COBLL1 -1.574845836 5.768067023 -9.690555427 3.30E-18 6.67E-17 30.40677557 DOWN COBLL1

GFOD1 -1.188154967 3.133895292 -9.682450465 3.48E-18 7.01E-17 30.52095177 DOWN GFOD1

DYRK2 1.06415614 3.692334471 9.68243878 3.48E-18 7.01E-17 30.44408391 UP DYRK2

APOC1 -2.346854516 11.26216479 -9.674572664 3.66E-18 7.38E-17 30.36258643 DOWN APOC1

HMGCS2 -2.899498346 9.178079488 -9.673357813 3.69E-18 7.43E-17 30.30886311 DOWN HMGCS2

C19orf48 1.207729349 5.333285918 9.672619228 3.71E-18 7.46E-17 30.2957393 UP C19orf48

TRPC1 1.879935079 0.081481941 9.671309931 3.74E-18 7.51E-17 30.62454059 UP TRPC1

CDK19 1.020115463 3.603896321 9.662879192 3.95E-18 7.92E-17 30.32525513 UP CDK19

RP11_216L13.19 2.048255509 -1.224552329 9.662548986 3.96E-18 7.93E-17 30.60119189 UP RP11-216L13.19

DDX11 1.283524298 3.031593624 9.657726514 4.09E-18 8.17E-17 30.34157709 UP DDX11

KIF24 1.34062826 0.533348393 9.652877873 4.22E-18 8.42E-17 30.4918256 UP KIF24

RP11_96D1.6 -2.600339913 -1.212585001 -9.65283699 4.22E-18 8.42E-17 30.54737406 DOWN RP11-96D1.6

PAH -2.909810183 8.51071059 -9.649674794 4.30E-18 8.59E-17 30.14682094 DOWN PAH

RP11_700H6.4 -2.394498257 -1.975283877 -9.648625693 4.33E-18 8.64E-17 30.52084313 DOWN RP11-700H6.4

HIST3H2A	3.126987953	-1.208061066	9.646868943	4.38E-18	8.73E-17	30.49702114	UP	HIST3H2A
FAXDC2	-1.712762732	5.521405617	-9.641033103	4.55E-18	9.04E-17	30.09701786	DOWN	FAXDC2
ZNF816	1.958592651	0.343353239	9.64074596	4.56E-18	9.05E-17	30.41809109	UP	ZNF816
USHBP1	-1.071575457	1.485290653	-9.639668661	4.59E-18	9.11E-17	30.3805078	DOWN	USHBP1
HMGA2	4.054754587	-3.1313023	9.634222875	4.76E-18	9.43E-17	30.42826734	UP	HMGA2
RP11_1267H10.3	-2.436770092	-4.094384488	-9.617769232	5.29E-18	1.05E-16	30.32153662	DOWN	RP11-1267H10.3
RUNDC3A	2.252907458	-2.30383157	9.612537762	5.47E-18	1.08E-16	30.29142237	UP	RUNDC3A
NXPH4	3.102802026	0.120620111	9.608768026	5.61E-18	1.10E-16	30.21625135	UP	NXPH4
MT_ND5	-1.525349649	10.88260992	-9.603067083	5.82E-18	1.14E-16	29.89723284	DOWN	MT-ND5
RNU1_70P	-2.992769188	3.275646519	-9.601010016	5.90E-18	1.16E-16	30.03812873	DOWN	RNU1-70P
GPLD1	-3.243511462	3.317005273	-9.599303811	5.96E-18	1.17E-16	30.03566069	DOWN	GPLD1
SLC25A20	-1.251883587	5.673339088	-9.597641914	6.03E-18	1.18E-16	29.80866464	DOWN	SLC25A20
SPATC1L	2.330008217	1.350159423	9.593596606	6.19E-18	1.21E-16	30.05788414	UP	SPATC1L
CCM2L	-1.123856257	1.278314667	-9.59075887	6.30E-18	1.23E-16	30.0810227	DOWN	CCM2L
ADH1A	-3.460094462	7.297526819	-9.587296857	6.45E-18	1.26E-16	29.7435823	DOWN	ADH1A
TRAF5	1.297156651	2.333122308	9.583752005	6.60E-18	1.29E-16	29.92814345	UP	TRAF5
TPRG1L	-1.04383379	6.135509908	-9.581732522	6.68E-18	1.30E-16			

29.69789702 DOWN TPRG1L
HAO2 -4.655326889 2.957260494 -9.580375069 6.74E-18 1.31E-16
29.98030021 DOWN HAO2
MPP2 2.262876728 -1.731474693 9.579156855 6.79E-18 1.32E-16
30.07681364 UP MPP2
CES4A -2.80234939 0.901143552-9.578139503 6.84E-18 1.33E-16
30.03464088 DOWN CES4A
RORC -2.451402486 6.164548507 -9.576597188 6.91E-18 1.34E-16
29.67773739 DOWN RORC
LINC00526 -1.520427664 2.169458606 -9.574562391 7.00E-18 1.36E-
16 29.92394273 DOWN LINC00526
RAMP1 -2.621567279 5.56302489 -9.572071105 7.11E-18 1.38E-16
29.67099547 DOWN RAMP1
NANOS1 2.504237923 -0.635757902 9.566489568 7.37E-18 1.43E-
16 29.97674435 UP NANOS1
KHK -2.07353612 6.844773117-9.565700001 7.41E-18 1.43E-16
29.59590289 DOWN KHK
ACOT1 -1.712568611 2.698325209 -9.565290781 7.43E-18 1.44E-16
29.82404644 DOWN ACOT1
TCTN2 1.567801061 1.5115944399.565100824 7.44E-18 1.44E-16
29.87187266 UP TCTN2
VEGFB 1.926173072 4.528284306 9.564336549 7.48E-18 1.44E-16
29.64131536 UP VEGFB
ASL -1.625982906 6.890432163 -9.563940052 7.50E-18 1.45E-16
29.58241697 DOWN ASL
BDNF_AS -1.272211202 0.680764912 -9.56328169 7.53E-18 1.45E-16
29.9359492 DOWN BDNF-AS
ZIC5 4.087528409 -1.591264529 9.56312166 7.54E-18 1.45E-16
29.96496771 UP ZIC5
MYLK -1.461154487 5.824355577 -9.562904885 7.55E-18 1.45E-16
29.58528801 DOWN MYLK
SEC16B -1.767761642 1.122818327 -9.559817515 7.70E-18 1.48E-
16 29.89879078 DOWN SEC16B

CAPG 1.92063652 3.980010738 9.557590666 7.81E-18 1.50E-16 29.63233102 UP CAPG
LINC01127 -2.501952916 1.347425243 -9.556522468 7.86E-18 1.51E-16 29.87501934 DOWN LINC01127
IDNK -1.345052646 3.507023481 -9.554739377 7.95E-18 1.52E-16 29.669935 DOWN IDNK
RP11_796E2.4 1.966280229 -2.123560561 9.552368877 8.08E-18 1.55E-16 29.91009952 UP RP11-796E2.4
RP11_572O17.1 1.5062087 -1.894472804 9.5503896 8.18E-18 1.56E-16 29.89731955 UP RP11-572O17.1
MT_CO1 -1.14646173 13.68809837 -9.550219122 8.19E-18 1.56E-16 29.63461539 DOWN MT-CO1
MFSD2A -4.288747856 3.117788005-9.544595455 8.49E-18 1.62E-16 29.73339086 DOWN MFSD2A
CHML 1.570476851 3.007723697 9.54360276 8.55E-18 1.63E-16 29.61376896 UP CHML
SMIM19 -1.272802576 4.716973324 -9.540195235 8.74E-18 1.66E-16 29.47796865 DOWN SMIM19
RP11_245P10.4 2.026487584 -4.552995435 9.540082762 8.74E-18 1.66E-16 29.83160268 UP RP11-245P10.4
C15orf43 -3.361355261 -3.491131372 -9.533737567 9.11E-18 1.73E-16 29.79232176 DOWN C15orf43
DNA2 1.210520321 1.390057392 9.533183153 9.14E-18 1.74E-16 29.68042564 UP DNA2
PKIA 2.764630512 -1.968955692 9.531809077 9.22E-18 1.75E-16 29.77713269 UP PKIA
GS1_21A4.2 -2.590555694 -2.688034458 -9.531068446 9.27E-18 1.76E-16 29.77637375 DOWN GS1-21A4.2
C3 -1.823572973 12.83936014 -9.529663289 9.35E-18 1.77E-16 29.47690974 DOWN C3
KLF15 -2.079832448 5.68285528 -9.527934456 9.46E-18 1.79E-16 29.37283166 DOWN KLF15
CMBL -2.327419657 6.294886859 -9.524680542 9.66E-18 1.83E-16

29.34110639DOWN CMBL

HINT2 -1.244921476 5.551211171-9.524016845 9.70E-18 1.83E-16

29.34000297 DOWN HINT2

BCORL1 1.130882918 3.235032437 9.523454484 9.73E-18 1.84E-16 29.46399276 UP BCORL1

FSD1L 1.359449361 0.577125221 9.522572804 9.79E-18 1.85E-16

29.65940776 UP FSD1L

FAM20A -1.596983502 5.916043745 -9.520018528 9.95E-18 1.88E-16 29.30984213 DOWN FAM20A

AC016768.1-2.931265397 -0.464491561 -9.514845719 1.03E-17 1.94E-16 29.66742525 DOWN AC016768.1

AC026202.3-1.54162478-1.382254167 -9.512425127 1.05E-17 1.97E-16

29.65814293 DOWN AC026202.3

CERS1 3.160225629 -2.783322948 9.512106069 1.05E-17 1.97E-16

29.65542211UP CERS1

MIR99AHG-2.046157693 0.943494854 -9.503835246 1.10E-17 2.07E-16 29.55414554 DOWN MIR99AHG

OSGIN1 -2.180108241 6.698648629 -9.5024389 1.11E-17 2.09E-16

29.19249842 DOWN OSGIN1

LEPR -2.856514291 5.68812811 -9.500957723 1.13E-17 2.11E-16

29.21801877 DOWN LEPR

RP5_1103B4.3 -2.187545716 -2.470314745 -9.49779376 1.15E-17 2.15E-16 29.56673704 DOWN RP5-1103B4.3

ZNF738 1.919349448 -0.542359888 9.495707959 1.16E-17 2.18E-16

29.52818817 UP ZNF738

ENTPD5 -1.665471214 6.900213743 -9.493899598 1.18E-17 2.20E-16 29.13447571 DOWN ENTPD5

ANO9 2.587221872 0.9211757199.486560631 1.23E-17 2.31E-16

29.40149169 UP ANO9

PIK3R1 -1.315920646 6.014532248 -9.485245413 1.25E-17 2.32E-16

29.08370123 DOWN PIK3R1

HOMER3 1.558584997 3.150667697 9.48163881 1.27E-17 2.38E-16

29.20636252 UP HOMER3

FAM186B -1.336689089 -0.889431416 -9.481297827 1.28E-17 2.38E-16 29.45658558 DOWN FAM186B
ARL6IP6 1.018879724 2.294357338 9.480197695 1.29E-17 2.39E-16 29.27305511UP ARL6IP6
RP5_821D11.7 1.062967109 0.93017863 9.475663694 1.32E-17 2.46E-16 29.34464742 UP RP5-821D11.7
RUSC1 1.000916324 4.0611638639.47006792 1.37E-17 2.55E-16 29.05616878 UP RUSC1
RETSAT -1.186447972 6.920643902 -9.46707191 1.40E-17 2.60E-16 28.96180775 DOWN RETSAT
STX3 1.154702817 3.64979418 9.466781387 1.40E-17 2.60E-16 29.06757776 UP STX3
IQGAP3 1.924348509 3.046832122 9.465895716 1.41E-17 2.62E-16 29.11716682UP IQGAP3
XRCC3 1.003012698 2.437439565 9.46369933 1.43E-17 2.65E-16 29.15498184 UP XRCC3
DNMT3B 1.448269162 0.889736576 9.457123972 1.49E-17 2.76E-16 29.22584518 UP DNMT3B
FAM60A 1.439457877 3.107921272 9.456538936 1.50E-17 2.77E-16 29.0493247 UP FAM60A
TMEM100 -3.281407276 0.1219953 -9.455235066 1.51E-17 2.79E-16 29.28138053 DOWN TMEM100
HSD17B10 -1.020247984 6.678772289 -9.454925971 1.51E-17 2.79E-16 28.88387911DOWN HSD17B10
C2orf27A 1.871711239-0.730737451 9.447725254 1.59E-17 2.92E-16 29.22978879 UP C2orf27A
RP11_372E1.4 -4.106530895 2.135197472 -9.442030755 1.64E-17 3.03E-16 29.12882768 DOWN RP11-372E1.4
RP11_6F2.5 -3.094451548 -2.422660176 -9.441091441 1.65E-17 3.04E-16 29.210002 DOWN RP11-6F2.5
BICD1 1.471557646 1.371476651 9.4366011011.70E-17 3.13E-16 29.06488511UP BICD1
P3H4 1.609107351 3.293612804 9.435015091 1.72E-17 3.16E-16

28.89807379 UP P3H4

TMEM132A2.30058965 2.920007049 9.431291364 1.76E-17 3.22E-16 28.91040603 UP TMEM132A

PCLO 3.670242829 -2.713417224 9.430331471 1.77E-17 3.24E-16 29.13784389 UP PCLO

C4BPB -2.443764162 7.387337634 -9.428103988 1.80E-17 3.28E-16 28.71843159 DOWN C4BPB

KANK3 -1.190175993 2.124060543 -9.42690668 1.81E-17 3.31E-16 28.98176446 DOWN KANK3

ASS1 -1.769909427 8.885763059 -9.424147309 1.85E-17 3.36E-16 28.7095763 DOWN ASS1

LINC01018 -5.848807973 2.241505116-9.422605464 1.86E-17 3.40E-16 29.03196115DOWN LINC01018

RP11_726G1.1 1.658374044 -2.127985052 9.421981442 1.87E-17 3.41E-16 29.08766809 UP RP11-726G1.1

ZDHHC8P1 2.925709375 -3.364573968 9.419617019 1.90E-17 3.46E-16 29.07456023 UP ZDHHC8P1

AP1G2 1.486497128 2.964954993 9.419464635 1.90E-17 3.46E-16 28.82569524 UP AP1G2

PPP4R4 -1.755346578 1.524674901 -9.418488112 1.91E-17 3.48E-16 28.97967547 DOWN PPP4R4

PLGLA -3.762533754 -0.886606805 -9.416928647 1.93E-17 3.51E-16 29.05462858 DOWN PLGLA

RP11_622A1.2 -3.346409102 0.901248653 -9.409737498 2.02E-17 3.67E-16 28.97112272DOWN RP11-622A1.2

RP11_285E23.2 -2.263750934 -3.166360827 -9.40648359 2.07E-17 3.74E-16 28.99144862 DOWN RP11-285E23.2

AC025335.1 1.398929726 -0.132424758 9.404806429 2.09E-17 3.78E-16 28.94249423 UP AC025335.1

MOGAT1 -2.398406559 -2.595297144 -9.404653683 2.09E-17 3.78E-16 28.98070298 DOWN MOGAT1

RAB11B_AS1 -1.513810517 2.995540375 -9.400973508 2.14E-17 3.86E-16 28.74394912 DOWN RAB11B-AS1

MOCOS	-1.327547576	4.759157019	-9.40093831	2.14E-17	3.86E-16	28.58828155	DOWN	MOCOS
SAA4	-3.575351025	4.928355315	-9.400828858	2.14E-17	3.86E-16	28.64852053	DOWN	SAA4
PROS1	-1.452293976	6.98120346	-9.400813991	2.14E-17	3.86E-16	28.54002256	DOWN	PROS1
DIAPH3	1.907843996	0.724021236	9.397782873	2.19E-17	3.93E-16	28.85524763	UP	DIAPH3
APBA1	-2.041030028	2.945592092	-9.397455756	2.19E-17	3.94E-16	28.7436196	DOWN	APBA1
AP000473.5	-2.08843436	-2.393044395	-9.391132497	2.28E-17	4.10E-16	28.89589985	DOWN	AP000473.5
CTNND2	5.29158044	-0.531842018	9.386720657	2.35E-17	4.21E-16	28.81866824	UP	CTNND2
RP11_968A15.2	1.695909284	-2.304632679	9.386528162	2.35E-17	4.21E-16	28.86532317	UP	RP11-968A15.2
BEX2	3.608213597	-0.208754514	9.38423932	2.38E-17	4.26E-16	28.80156616	UP	BEX2
SLC9B2	-1.633114094	4.044953277	-9.380418328	2.44E-17	4.36E-16	28.51755938	DOWN	SLC9B2
COPZ2	-1.484457779	3.747400315	-9.376624666	2.50E-17	4.47E-16	28.51653917	DOWN	COPZ2
TRIM45	1.609058398	1.357218395	9.374922927	2.53E-17	4.51E-16	28.67372637	UP	TRIM45
GIPR	2.540898333	-1.334540441	9.374903236	2.53E-17	4.51E-16	28.77999578	UP	GIPR
ABCG5	-2.709513379	4.383091324	-9.371899397	2.58E-17	4.59E-16	28.47215059	DOWN	ABCG5
FAM72C	2.179600085	-3.04701728	9.36696928	2.66E-17	4.73E-16	28.74387536	UP	FAM72C
FABP4	-3.154905682	1.883743502	-9.366650621	2.67E-17	4.74E-16	28.64968869	DOWN	FABP4
FBXO41	1.931538538	-0.016760793	9.365467295	2.69E-17	4.77E-			

16 28.68614372 UP FBXO41

PIFO 2.569674653 -2.831755523 9.3650397112.70E-17 4.78E-16 28.73060682 UP PIFO

SIAH2_AS1 -2.001509711 -2.733216233 -9.364048856 2.71E-17 4.81E-16 28.72566553 DOWN SIAH2-AS1

S100A11 1.682519549 5.457973404 9.362673148 2.74E-17 4.85E-16 28.31516326 UP S100A11

CACNB1 1.382529052 -0.138118629 9.358422803 2.81E-17 4.98E-16 28.65007788 UP CACNB1

NUDT1 1.277404718 2.870798964 9.35709433 2.84E-17 5.02E-16 28.43728884 UP NUDT1

CTC_260E6.6 1.886888645 -3.304165424 9.355801891 2.86E-17 5.05E-16 28.67425049 UP CTC-260E6.6

EVA1A -2.127876131 5.057914896 -9.350871854 2.95E-17 5.21E-16 28.27106013 DOWN EVA1A

RP11_213H15.1 -2.311421652 -3.405753953 -9.349856682 2.97E-17 5.24E-16 28.63612919 DOWN RP11-213H15.1

RP1_30M3.6 -1.073662901 -1.179552183 -9.345226556 3.06E-17 5.39E-16 28.60208781 DOWN RP1-30M3.6

AGMO -2.234708442 5.193244479 -9.34522206 3.06E-17 5.39E-16 28.23165289 DOWN AGMO

RNASEH2A1.160935638 3.710616856 9.344909164 3.07E-17 5.39E-16 28.28716258 UP RNASEH2A

CFHR5 -4.264094995 5.023379145 -9.344598979 3.08E-17 5.40E-16 28.32071105DOWN CFHR5

GPC2 1.698144868 -2.096202965 9.343845756 3.09E-17 5.42E-16 28.59611518UP GPC2

CD81 -1.144653001 8.725810482 -9.342636765 3.11E-17 5.46E-16 28.18940658 DOWN CD81

LHFPL2 1.476537205 3.099584913 9.337540696 3.22E-17 5.64E-16 28.29380253 UP LHFPL2

AC138430.4 -2.454175406 -3.606755572 -9.337430252 3.22E-17 5.64E-16 28.55820112DOWN AC138430.4

SPAG5	1.339906482	4.102607076	9.336569036	3.24E-17	5.67E-16	28.20706214	UP	SPAG5
BHMT2	-2.526798296	7.191019852	-9.335538443	3.26E-17	5.70E-16	28.12968558	DOWN	BHMT2
RP13_650J16.1	-3.07469974	0.223814511	-9.334435621	3.28E-17	5.73E-16	28.51542323	DOWN	RP13-650J16.1
AC009005.2	1.771790036	-0.734208966	9.33380701	3.30E-17	5.75E-16	28.51132329	UP	AC009005.2
ACADS	-1.258993674	6.105152052	-9.33373692	3.30E-17	5.75E-16	28.11685166	DOWN	ACADS
GPR155	-1.193882802	3.359905222	-9.333040089	3.31E-17	5.77E-16	28.26975717	DOWN	GPR155
IGF2BP3	3.938083077	-1.236696097	9.332574333	3.32E-17	5.78E-16	28.50400635	UP	IGF2BP3
KLHDC8A	2.935985665	-2.805756591	9.332310814	3.33E-17	5.79E-16	28.52400635	UP	KLHDC8A
ELOVL7	3.673364524	0.594749607	9.331432373	3.35E-17	5.82E-16	28.43110974	UP	ELOVL7
DRAM1	1.053569774	3.603241908	9.331250278	3.35E-17	5.82E-16	28.20862559	UP	DRAM1
PER1	-1.272166572	5.640187412	-9.324017306	3.51E-17	6.08E-16	28.06376347	DOWN	PER1
UGT1A1	-3.661246521	4.370875993	-9.323632019	3.52E-17	6.09E-16	28.21218857	DOWN	UGT1A1
LPA	-3.523701934	3.141411515	-9.316859903	3.67E-17	6.35E-16	28.2566035	DOWN	LPA
ALPL	-2.593284212	4.34525903	-9.316127307	3.69E-17	6.37E-16	28.11724602	DOWN	ALPL
RP11_817O13.8	-1.326086023	0.809995047	-9.313817449	3.75E-17	6.46E-16	28.35064055	DOWN	RP11-817O13.8
GSTA1	-3.945124217	7.220019973	-9.313202022	3.76E-17	6.48E-16	28.00159946	DOWN	GSTA1
SAT1	-1.033380417	8.045433211	-9.30908739	3.86E-17	6.65E-16			

27.96565708 DOWN SAT1

EGLN3 2.732434858 1.563293124 9.304169644 3.98E-17 6.86E-16 28.20358221 UP EGLN3

DYSF -1.163550101 5.116692279-9.29833247 4.14E-17 7.11E-16 27.91621033 DOWN DYSF

HDAC11 1.044696346 4.098647801 9.296137277 4.19E-17 7.20E-16 27.94808987 UP HDAC11

MAFG_AS1 1.8297817 0.386727089 9.293310997 4.27E-17 7.32E-16 28.2130323 UP MAFG-AS1

LRRC3 -2.126938776 3.822545164 -9.289506269 4.38E-17 7.50E-16 27.97568827 DOWN LRRC3

RELT 1.252403374 1.158460843 9.284931797 4.51E-17 7.71E-16 28.12163614 UP RELT

CDC37L1_AS1 -1.511677759 -0.567383032 -9.28171893 4.60E-17 7.85E-16 28.19479085 DOWN CDC37L1-AS1

SORD2P -2.500244647 1.965206065 -9.278568474 4.69E-17 8.00E-16 28.07772055 DOWN SORD2P

RIPPLY1 -3.301154231 -0.324807614 -9.276888498 4.74E-17 8.08E-16 28.16688342 DOWN RIPPLY1

ITPR2 -1.431444175 6.234256516 -9.271067105 4.92E-17 8.37E-16 27.71885503 DOWN ITPR2

MYB 2.019725893 -1.799604427 9.271010875 4.93E-17 8.37E-16 28.13551023 UP MYB

RP3_323A16.1 2.515315501 -3.455568204 9.27067114 4.94E-17 8.39E-16 28.1408157 UP RP3-323A16.1

TTF2 1.107998934 2.2610851199.270513679 4.94E-17 8.39E-16 27.94501186UP TTF2

ITIH1 -2.774695121 9.611841921-9.267247257 5.05E-17 8.56E-16 27.72499135 DOWN ITIH1

HYAL1 -1.74658881 6.510029428 -9.264415306 5.14E-17 8.70E-16 27.67613691 DOWN HYAL1

PAOX -1.076403401 3.556166575 -9.259606565 5.30E-17 8.96E-16 27.78028402 DOWN PAOX

RCAN3 1.435202329 2.164428959 9.25528244 5.45E-17 9.20E-16
27.85462853 UP RCAN3

DUSP16 -1.110413093 5.93529563 -9.25203429 5.56E-17 9.39E-16
27.60016403 DOWN DUSP16

TMEM53 -1.014775521 4.553980808 -9.251952303 5.56E-17 9.39E-16 27.64807861 DOWN TMEM53

ZNF233 1.803904092 -0.784390077 9.249704454 5.65E-17 9.51E-16
27.98401571 UP ZNF233

MBL2 -3.520135169 5.088994913 -9.245169148 5.81E-17 9.76E-16
27.64782116DOWN MBL2

CTD_3098H1.2 -4.021518541 -1.117492276 -9.242793089 5.90E-17
9.90E-16 27.96279609 DOWN CTD-3098H1.2

RP11_426C22.7 -2.660704658 -3.375982928 -9.235811054 6.17E-17
1.03E-15 27.923439 DOWN RP11-426C22.7

CCS -1.008333013 5.628013101 -9.232521057 6.30E-17 1.05E-15
27.48159377 DOWN CCS

RBP7 -1.835815686 2.828190928 -9.230089888 6.40E-17 1.07E-15
27.68684878 DOWN RBP7

CKAP2 1.108471083 3.001733761 9.227446996 6.51E-17 1.09E-15
27.60469587 UP CKAP2

C16orf70 -1.073130363 4.634033689 -9.22647226 6.55E-17 1.09E-15
27.48346567 DOWN C16orf70

GCLC -1.209877695 6.21174432 -9.225660182 6.58E-17 1.10E-15
27.4303432 DOWN GCLC

UBE2Q2 1.316440837 3.58673784 9.225079186 6.61E-17 1.10E-15
27.53946282 UP UBE2Q2

EXOC3L2 -1.168457868 2.60704158 -9.223028468 6.69E-17 1.12E-15
27.64807107 DOWN EXOC3L2

RP11_353N14.2 2.565778753 -3.532616528 9.22101616 6.78E-17 1.13E-15 27.83065297 UP RP11-353N14.2

KDM8 -1.97192433 3.348948195 -9.22011908 6.82E-17 1.13E-15
27.57938107 DOWN KDM8

TMSB10 1.749283289 8.344204991 9.217785812 6.92E-17 1.15E-

15 27.40510884 UP TMSB10

HULC -3.390888654 5.913021819 -9.215916849 7.00E-17 1.16E-15 27.40807136 DOWN HULC

FAM81A 2.023893244 -0.915779022 9.215051982 7.04E-17 1.17E-15 27.76932796 UP FAM81A

MISP 4.625671033 -1.453616274 9.2114475277.21E-17 1.19E-15 27.74074815 UP MISP

HNMT -1.058284309 6.407809137 -9.205123546 7.50E-17 1.24E-15 27.29832403 DOWN HNMT

SLCO2A1 -2.365901243 3.004825888 -9.204614285 7.53E-17 1.25E-15 27.52751749 DOWN SLCO2A1

IGDCC4 2.905490359 -1.461837648 9.201734453 7.67E-17 1.27E-15 27.69282043 UP IGDCC4

DAK -1.707368565 6.564182184 -9.199868322 7.76E-17 1.28E-15 27.26718471 DOWN DAK

C10orf35 2.255924057 1.176038262 9.196486361 7.93E-17 1.31E-15 27.55340273 UP C10orf35

RP11_313J2.1 2.729864487 -2.011570201 9.188316147 8.35E-17 1.37E-15 27.6172941 UP RP11-313J2.1

LRRC2 -2.368861456 2.296262872 -9.186553907 8.45E-17 1.39E-15 27.47260049 DOWN LRRC2

RP11_143N13.2 -2.176596467 -3.988732584 -9.183115009 8.63E-17 1.42E-15 27.59488285 DOWN RP11-143N13.2

FGG -2.797819995 11.66969634-9.182066201 8.69E-17 1.43E-15 27.23455916 DOWN FGG

CUX2 -3.453246129 3.877508938 -9.179820937 8.82E-17 1.45E-15 27.33656756 DOWN CUX2

MST1 -2.015341862 7.138728963 -9.178136992 8.91E-17 1.46E-15 27.13011856DOWN MST1

UGT1A3 -4.343382944 0.764200845 -9.175101587 9.09E-17 1.49E-15 27.50748648 DOWN UGT1A3

ALDH1A1 -2.083284623 9.365904599 -9.173373758 9.19E-17 1.50E-15 27.12764595 DOWN ALDH1A1

ARMC5	-1.231941741	4.018475991	-9.165836643	9.64E-17	1.58E-15	27.14941894	DOWN	ARMC5
F10_AS1	-1.831833412	-3.12566329	-9.164748777	9.71E-17	1.59E-15	27.4808119	DOWN	F10-AS1
SFXN2	-1.207686458	3.962471205	-9.162537203	9.84E-17	1.61E-15	27.13273633	DOWN	SFXN2
MMP9	2.714852015	2.537662597	9.155486113	1.03E-16	1.68E-15	27.19748769	UP	MMP9
ANGPTL3	-2.951489058	7.368880934	-9.149861477	1.07E-16	1.74E-15	26.9571564	DOWN	ANGPTL3
ST6GALNAC5	2.811422529	-3.456984733	9.148054559	1.08E-16	1.76E-15	27.37528438	UP	ST6GALNAC5
GNG7	-1.522796635	2.813088999	-9.145814988	1.09E-16	1.78E-15	27.15048524	DOWN	GNG7
RP11_112J3.16	1.53218115	-2.797980791	9.145614838	1.10E-16	1.78E-15	27.36035097	UP	RP11-112J3.16
ZNF525	1.929452592	0.82410368	9.144913798	1.10E-16	1.79E-15	27.25545658	UP	ZNF525
SLC25A30	-1.512289303	5.082801034	-9.141147565	1.13E-16	1.83E-15	26.93005366	DOWN	SLC25A30
CLDN14	-2.074172081	3.664573702	-9.140862426	1.13E-16	1.83E-15	27.05203544	DOWN	CLDN14
PMAIP1	1.990193265	-1.182365686	9.1308864	1.20E-16	1.95E-15	27.24957815	UP	PMAIP1
NCAPG2	1.07760327	3.06244728	9.125495147	1.25E-16	2.01E-15	26.95654588	UP	NCAPG2
ENO2	2.057877511	1.407202818	9.122128962	1.27E-16	2.05E-15	27.07193568	UP	ENO2
ZNF28	2.021210093	1.718364477	9.122080545	1.27E-16	2.05E-15	27.04880119	UP	ZNF28
LECT2	-4.675597173	4.050650886	-9.120320942	1.29E-16	2.08E-15	26.99162648	DOWN	LECT2
MT_ATP6	-1.150033012	11.8572114	-9.114416597	1.34E-16	2.15E-15			

26.81961141DOWN MT-ATP6
ADK -1.124945496 5.298582477 -9.114415856 1.34E-16 2.15E-15
26.74656979 DOWN ADK
UBE2SP1 1.734974904 -2.890370176 9.1141056291.34E-16 2.16E-15
27.16428238 UP UBE2SP1
FAM171A2 1.95139817 -0.325596025 9.1113299491.36E-16 2.19E-15
27.10185712 UP FAM171A2
SIX2 3.256552301 -2.489853156 9.109500559 1.38E-16 2.22E-15
27.12760508 UP SIX2
KISS1R 2.956447472 -4.3730552 9.10840164 1.39E-16 2.23E-15
27.13047381 UP KISS1R
RNF144A 1.452319458 2.202232383 9.102765533 1.44E-16 2.30E-
15 26.89099942 UP RNF144A
LMF1 -1.331948742 3.939726141 -9.10246207 1.44E-16 2.31E-15
26.7599858 DOWN LMF1
S1PR1 -1.290747132 4.044446278 -9.096183986 1.50E-16 2.40E-15
26.71156742DOWN S1PR1
AC005077.14 -2.752302818 0.762794637 -9.09127076 1.55E-16 2.47E-
15 26.96940559 DOWN AC005077.14
RILP -1.222556089 4.102101389 -9.088323191 1.58E-16 2.51E-15
26.65358998 DOWN RILP
C11orf71 -1.051330508 3.278351845 -9.086218 1.60E-16 2.54E-15
26.71513179 DOWN C11orf71
ELFN1 -2.92231749 2.918347111-9.081538655 1.65E-16 2.62E-15
26.77663002 DOWN ELFN1
DDX12P 1.198515164 -0.03776471 9.081424614 1.65E-16 2.62E-15
26.90887307 UP DDX12P
CKMT1B 2.976436263 -4.30517308 9.0807118461.66E-16 2.63E-15
26.95852776 UP CKMT1B
AP001626.2 2.547657591 -4.411906742 9.077997578 1.68E-16 2.67E-
15 26.94209507 UP AP001626.2
SLC38A1 2.16180895 4.330615287 9.072023091 1.75E-16 2.77E-15
26.534259 UP SLC38A1

FAM227B -1.029492083 0.778274424 -9.07167711 1.75E-16 2.77E-15 26.83012638 DOWN FAM227B

TAF1A_AS1 -1.286353091 0.689034496 -9.067975465 1.80E-16 2.84E-15 26.81376631 DOWN TAF1A-AS1

RP5_849H19.3 -2.808869698 1.84223702 -9.065057339 1.83E-16 2.89E-15 26.74947664 DOWN RP5-849H19.3

LINC00665 2.541820459 1.966410836 9.062392938 1.86E-16 2.94E-15 26.65379845 UP LINC00665

VWA5B2 2.130402227 -2.490306485 9.057468793 1.92E-16 3.03E-15 26.8090388 UP VWA5B2

ALDH1L1_AS2 -2.870565672 -0.49997766 -9.055549701 1.94E-16 3.06E-15 26.78607586 DOWN ALDH1L1-AS2

CMTM7 1.235757723 2.8451181619.054873708 1.95E-16 3.07E-15 26.5331589 UP CMTM7

HILPDA 1.382145633 2.090991421 9.048736062 2.03E-16 3.19E-15 26.56270817 UP HILPDA

LRFN1 1.862391649 0.329324629 9.043017995 2.10E-16 3.31E-15 26.64660411UP LRFN1

RP11_258F1.2 -3.473863513 -1.019456728 -9.03986909 2.15E-16 3.37E-15 26.69776542 DOWN RP11-258F1.2

ACACB-1.332669337 5.777836856 -9.036554235 2.19E-16 3.44E-15 26.24637753 DOWN ACACB

MT1X -3.149789985 5.775275837 -9.034405904 2.22E-16 3.48E-15 26.26537364 DOWN MT1X

RP11_178C3.2 -1.619644489 -1.501666576 -9.033181336 2.24E-16 3.50E-15 26.65875356 DOWN RP11-178C3.2

PLXNA3 1.5179511843.35305638 9.032453372 2.25E-16 3.52E-15 26.34867647 UP PLXNA3

RP11_635N19.1 -1.504541916 0.775951337 -9.029126272 2.30E-16 3.59E-15 26.56937448 DOWN RP11-635N19.1

STK39 1.843304362 2.716071342 9.028389463 2.31E-16 3.61E-15 26.37962252 UP STK39

3-Mar 1.556687616 0.78218669 9.0119955282.56E-16 3.99E-15

26.42989458 UP 3-Mar

AKR1C5P -2.589030675 -3.17607955 -9.011824476 2.56E-16 3.99E-15 26.53250748 DOWN AKR1C5P

FOXO1 -1.191962931 4.438940577 -9.006760566 2.65E-16 4.12E-15 26.11607181 DOWN FOXO1

AADACP1 -2.367719607 0.742663535 -9.005099792 2.67E-16 4.15E-15 26.4293917 DOWN AADACP1

CTB_43E15.1 -2.229416262 -5.124448485 -9.004771758 2.68E-16 4.16E-15 26.48911399 DOWN CTB-43E15.1

MMP11 2.176660633 2.936508041 9.004716155 2.68E-16 4.16E-15 26.21611239 UP MMP11

OCEL1 -1.09234241 4.998150015 -9.001084657 2.74E-16 4.25E-15 26.04571692 DOWN OCEL1

MT_CO2 -1.075619055 12.62932272 -9.000350865 2.76E-16 4.27E-15 26.12539715 DOWN MT-CO2

PXDC1 -1.069580969 6.32918539 -8.998280944 2.79E-16 4.32E-15 25.99707409 DOWN PXDC1

NUP210 1.029119285 5.438694576 8.993313246 2.88E-16 4.46E-15 25.97863736 UP NUP210

RNLS -1.387008274 2.849425504 -8.989402644 2.95E-16 4.57E-15 26.16388886 DOWN RNLS

CHAD -2.23658904 3.199224489 -8.98566857 3.02E-16 4.67E-15 26.12782901 DOWN CHAD

MCOLN3 3.549551562 -2.279554095 8.984310811 3.05E-16 4.71E-15 26.34637137 UP MCOLN3

PLCB1 1.624518396 3.13615278 8.97690399 3.20E-16 4.93E-15 26.02050871 UP PLCB1

AC093724.2 1.627538035 -2.325848145 8.971704329 3.30E-16 5.09E-15 26.27847427 UP AC093724.2

ECI1 -1.019741972 6.752265022 -8.968179292 3.38E-16 5.20E-15 25.8070126 DOWN ECI1

ADRA1B -2.42214903 0.879813329 -8.967420099 3.39E-16 5.22E-15 26.18967997 DOWN ADRA1B

MMP1 2.758578083 -1.161235468 8.966453124 3.41E-16 5.25E-15 26.22195432 UP MMP1

ZC3HAV1L 2.202986872 -1.705840806 8.964182544 3.46E-16 5.32E-15 26.22202781 UP ZC3HAV1L

RP11_153K11.3 -2.488066512 -3.031431943 -8.963560145 3.48E-16 5.34E-15 26.23433519 DOWN RP11-153K11.3

PRSS16 3.729550546 -2.811126227 8.963331433 3.48E-16 5.34E-15 26.2205899 UP PRSS16

SMC1B 2.512248604 -2.182652384 8.962293023 3.51E-16 5.37E-15 26.214601 UP SMC1B

SPOCD1 2.198417134 -1.924387578 8.961433654 3.52E-16 5.40E-15 26.20817066 UP SPOCD1

ZNF273 1.175539527 0.293107845 8.956152546 3.64E-16 5.57E-15 26.11374343 UP ZNF273

C11orf49 1.165856922 2.878174088 8.954659024 3.68E-16 5.62E-15 25.90321119 UP C11orf49

SCN4A -1.93720418 0.213621248 -8.953707555 3.70E-16 5.65E-15 26.12803788 DOWN SCN4A

MMP12 3.494974519 -2.071479023 8.950048437 3.79E-16 5.78E-15 26.13135595 UP MMP12

SLC47A1 -2.189084754 5.784308103 -8.949472203 3.80E-16 5.79E-15 25.71166874 DOWN SLC47A1

PM20D2 1.262615945 2.881961188 8.942119596 3.98E-16 6.06E-15 25.82453325 UP PM20D2

ZNF530 1.220236459 0.600912142 8.934102953 4.19E-16 6.35E-15 25.95965578 UP ZNF530

ABCB11 -4.242652846 2.921983476 -8.924551344 4.45E-16 6.73E-15 25.83562995 DOWN ABCB11

LRP8 1.756327932 -0.158464307 8.919485396 4.59E-16 6.94E-15 25.90264209 UP LRP8

RP11_701P16.2 -2.114950146 -4.133723761 -8.917646026 4.65E-16 7.01E-15 25.95192139 DOWN RP11-701P16.2

CES3 -2.532707901 4.015302802 -8.917204424 4.66E-16 7.03E-15

25.63710234	DOWN CES3

DLG3	2.069190296	1.796298923	8.915748621	4.70E-16	7.08E-15	25.75293038	UP	DLG3

CYP11A1	-3.693783264	1.178827464	-8.914638197	4.74E-16	7.12E-15	25.86201225	DOWN CYP11A1

CYP3A43	-3.229861897	0.183149091	-8.914443654	4.74E-16	7.13E-15	25.8953084	DOWN CYP3A43

LA16c_429E7.1 -1.837838891	-2.969200752	-8.909534343	4.89E-16	7.34E-15	25.90157833	DOWN LA16c-429E7.1

PCSK1N	4.256843271	-1.875444305	8.905662904	5.01E-16	7.51E-15	25.84657425	UP	PCSK1N

KIAA1377	1.820568264	-1.399600335	8.902714354	5.11E-16	7.64E-15	25.83833824	UP	KIAA1377

OR7E29P	-2.42707414 -3.96133439 -8.901500675	5.14E-16	7.69E-15	25.85255361	DOWN OR7E29P

ZNF385B	-3.127354673	1.794049619	-8.900537478	5.18E-16	7.73E-15	25.73407541	DOWN ZNF385B

PRR19	2.01261407	-0.908868331	8.895860484	5.33E-16	7.96E-15	25.78207258	UP	PRR19

ABCG8 -3.314896401	4.546164163	-8.89203513 5.46E-16	8.14E-15	25.46595175	DOWN ABCG8

RP11_70C1.3	-2.031444446	-4.113363036	-8.889409166	5.55E-16	8.27E-15	25.7782513	DOWN RP11-70C1.3

CGNL1 -1.644401921	5.794805128	-8.88887626 5.57E-16	8.30E-15	25.32537703	DOWN CGNL1

UBTFL10	-1.721662554	-5.72891585 -8.887957811	5.60E-16	8.34E-15	25.7691668	DOWN UBTFL10

MIR210HG 1.762389479	0.627580569	8.884257434	5.73E-16	8.52E-15	25.64310011 UP	MIR210HG

AC144449.1 -1.534093829	-1.85460857 -8.875892502	6.05E-16	8.97E-15	25.6903092	DOWN AC144449.1

TMEM205	-1.027156997	7.079281191 -8.869308901	6.30E-16	9.34E-15	25.19035569	DOWN TMEM205

AGL -1.207973311 4.668581702 -8.867738721 6.36E-16 9.42E-15 25.23295306 DOWN AGL
RP11_307C12.13 -1.763760723 -3.48475418 -8.861981391 6.60E-16 9.76E-15 25.60965745 DOWN RP11-307C12.13
RP3_337H4.8 1.140562839 -2.126473533 8.860781089 6.65E-16 9.83E-15 25.59393337 UP RP3-337H4.8
TFAP2A 2.812291737 -0.887496565 8.860402576 6.66E-16 9.84E-15 25.55769263 UP TFAP2A
IRS2 -1.46171952 6.005911344 -8.854232503 6.93E-16 1.02E-14 25.10343512 DOWN IRS2
PITPNM2 -1.152938877 4.415328087 -8.853506763 6.96E-16 1.02E-14 25.15984129 DOWN PITPNM2
CFB -1.823097839 8.079768042 -8.849222215 7.15E-16 1.05E-14 25.07447286 DOWN CFB
ARSA -1.01126721 5.810577035 -8.845468842 7.32E-16 1.08E-14 25.04873229 DOWN ARSA
SLC7A10 3.708938218 -3.184367851 8.844804041 7.35E-16 1.08E-14 25.49229601 UP SLC7A10
F7 -2.393274928 6.593139399 -8.843605225 7.41E-16 1.09E-14 25.03780487 DOWN F7
LAP3 -1.158611398 6.639226249 -8.843394702 7.42E-16 1.09E-14 25.02868454 DOWN LAP3
PEMT -1.419568578 5.351310903 -8.841901543 7.49E-16 1.10E-14 25.04250336 DOWN PEMT
FBXO31 -1.449260085 5.616784727 -8.836433491 7.75E-16 1.14E-14 25.00065858 DOWN FBXO31
AVPI1 -1.183755726 4.222203557 -8.834449247 7.84E-16 1.15E-14 25.05605541 DOWN AVPI1
UGT1A4 -5.298028723 3.195480552 -8.828607543 8.14E-16 1.19E-14 25.25514272 DOWN UGT1A4
KIF5A 2.445494133 -3.639733805 8.825537235 8.30E-16 1.21E-14 25.38304338 UP KIF5A
C16orf95 -1.230837602 0.417010752 -8.824413788 8.36E-16 1.22E-

14 25.3136481 DOWN C16orf95
RP11_86H7.7 2.235078569 -3.008269033 8.821523023 8.51E-16
1.24E-14 25.35555156 UP RP11-86H7.7
RP11_251M1.1 -1.788936794 -2.255829072 -8.820643259 8.56E-16
1.25E-14 25.35353697 DOWN RP11-251M1.1
FBXL19_AS1 1.45726398 -0.309977374 8.819368778 8.62E-16 1.26E-
14 25.29177366 UP FBXL19-AS1
RP11_798K3.2 -2.39641932 3.182874782 -8.817087776 8.75E-16 1.28E-
14 25.08445799 DOWN RP11-798K3.2
IGF1 -2.62833978 1.816302164 -8.814826788 8.87E-16 1.29E-14
25.19291774 DOWN IGF1
FTCD -2.929223896 7.289474148 -8.812700257 8.99E-16 1.31E-14
24.8460783 DOWN FTCD
CYP2U1 -1.21076743 2.415754015 -8.811205042 9.08E-16 1.32E-14
25.0936696 DOWN CYP2U1
PCCA -1.078525539 5.387002707 -8.810255017 9.13E-16 1.33E-14
24.84073097 DOWN PCCA
HDC -2.465884105 -1.189416693 -8.810059563 9.14E-16 1.33E-14
25.27929395 DOWN HDC
RP11_48B3.2 -2.502319023 -4.402553936 -8.808326977 9.24E-16
1.34E-14 25.28069094 DOWN RP11-48B3.2
IL4I1 2.0411781740.773298381 8.804477964 9.47E-16 1.37E-14
25.13709853 UP IL4I1
SC5D -1.478270228 6.107508138 -8.804440434 9.47E-16 1.37E-14
24.79224481 DOWN SC5D
CTHRC1 2.479909876 1.620058733 8.800261644 9.72E-16 1.41E-
14 25.04744274 UP CTHRC1
BCL9 1.132647183 3.87061049 8.795489032 1.00E-15 1.45E-14
24.82464352 UP BCL9
PCNXL2 1.899069399 1.095422508 8.794226684 1.01E-15 1.46E-
14 25.05406003 UP PCNXL2
ASB13 -1.061147379 5.303496379 -8.794208769 1.01E-15 1.46E-14
24.74349232 DOWN ASB13

KDR	-1.403323494	4.334791713	-8.790882797	1.03E-15	1.49E-14	24.78385402	DOWN	KDR
TRO	-1.784818428	2.223495377	-8.790044724	1.04E-15	1.49E-14	24.99211377	DOWN	TRO
CTD_2315E11.1	-2.032556583	-4.867688966	-8.789953951	1.04E-15	1.49E-14	25.16821199	DOWN	CTD-2315E11.1
RP11_883G14.1	-3.107669668	-3.143863347	-8.786373825	1.06E-15	1.53E-14	25.14508793	DOWN	RP11-883G14.1
RAPGEF4	-1.323044028	3.612279613	-8.785073929	1.07E-15	1.54E-14	24.81114412	DOWN	RAPGEF4
H6PD	-1.218573708	7.305023774	-8.784990029	1.07E-15	1.54E-14	24.66736612	DOWN	H6PD
RP11_930P14.2	1.737306589	-3.44308249	8.784914813	1.07E-15	1.54E-14	25.13501546	UP	RP11-930P14.2
SEMA4A	1.563252868	1.23627155	8.783622912	1.08E-15	1.55E-14	24.98114642	UP	SEMA4A
RP11_94C24.13	-1.714737088	-0.876239588	-8.777509399	1.12E-15	1.61E-14	25.07087477	DOWN	RP11-94C24.13
OSBP2	1.659483379	1.343683805	8.775693689	1.13E-15	1.62E-14	24.92339761	UP	OSBP2
SMIM2_AS1	-2.083680778	0.901775275	-8.774552618	1.14E-15	1.63E-14	24.9913032	DOWN	SMIM2-AS1
LINC01224	3.297391375	-3.464814356	8.774438983	1.14E-15	1.63E-14	25.06531701	UP	LINC01224
PLIN5	-1.889160803	4.928911425	-8.773410196	1.15E-15	1.64E-14	24.64623955	DOWN	PLIN5
UAP1L1	1.826113072	2.024855201	8.770138569	1.17E-15	1.68E-14	24.8317157	UP	UAP1L1
HDAC7	1.014186562	4.670643828	8.767775075	1.19E-15	1.70E-14	24.5999238	UP	HDAC7
SALL1	-1.851139108	4.896013844	-8.763523353	1.22E-15	1.74E-14	24.58684542	DOWN	SALL1
ELL2	-1.264884024	6.439980699	-8.762380532	1.23E-15	1.76E-14			

24.52662214 DOWN ELL2

RP11_118B18.2 -3.307632538 -2.630424452 -8.761922788 1.24E-15 1.76E-14 24.99375007 DOWN RP11-118B18.2

LDB2 -1.280984688 3.256332431 -8.761610146 1.24E-15 1.76E-14 24.70143857 DOWN LDB2

LINC00494 3.368155811-3.548318347 8.761496679 1.24E-15 1.76E-14 24.98627149 UP LINC00494

CLEC2L 3.552787241 -4.104459519 8.7525119311.31E-15 1.86E-14 24.93396384 UP CLEC2L

ARNT2 3.099688931 0.285188817 8.749529971 1.34E-15 1.89E-14 24.81990361 UP ARNT2

INHBC -2.690828233 4.13136356 -8.745143126 1.37E-15 1.94E-14 24.56609894 DOWN INHBC

FUCA1 -1.034246671 5.964852875 -8.745070869 1.37E-15 1.94E-14 24.42300396 DOWN FUCA1

RP11_475O23.2 -2.355568718 -3.307560681 -8.741663887 1.40E-15 1.98E-14 24.87249997 DOWN RP11-475O23.2

C11orf80 1.246969397 1.863297217 8.7349311841.46E-15 2.07E-14 24.63296623 UP C11orf80

FLVCR1 1.107181094 3.326155548 8.734123025 1.47E-15 2.08E-14 24.49096632 UP FLVCR1

MT_ND4 -1.10095401 13.24488527 -8.733203319 1.48E-15 2.09E-14 24.4805755 DOWN MT-ND4

CEBPB -1.04754903 6.522395313 -8.726242443 1.55E-15 2.18E-14 24.30157617 DOWN CEBPB

ZNF430 1.45576707 0.379801737 8.725185806 1.56E-15 2.19E-14 24.67833149 UP ZNF430

AKR1D1 -3.880472441 4.029490591 -8.723958729 1.57E-15 2.21E-14 24.49525666 DOWN AKR1D1

FAM167B -1.103270515 2.134522578 -8.718450908 1.62E-15 2.28E-14 24.54359346 DOWN FAM167B

CHST10 1.7606211860.934465504 8.714195231 1.67E-15 2.34E-14 24.57279259 UP CHST10

LHX3 -3.598386683 -3.3259568 -8.713918043 1.67E-15 2.35E-14 24.70209164 DOWN LHX3

FHL3 1.020010547 3.279753889 8.712111078 1.69E-15 2.37E-14 24.35894275 UP FHL3

ZNF107 1.351296928 1.244201993 8.71206548 1.69E-15 2.37E-14 24.54241465 UP ZNF107

DHRS4_AS1 -1.117467854 3.939351269 -8.711361639 1.70E-15 2.38E-14 24.31707175 DOWN DHRS4-AS1

ZNF724P 1.661931625 -2.271938344 8.71095252 1.70E-15 2.38E-14 24.67430836 UP ZNF724P

RP5_1065P14.2 -2.043450588 -3.066918946 -8.710888747 1.70E-15 2.38E-14 24.68443894 DOWN RP5-1065P14.2

RP11_131K5.2 -2.63265267 -4.687335719 -8.70870835 1.72E-15 2.41E-14 24.6718462 DOWN RP11-131K5.2

TUBA1C 1.030542602 4.900253971 8.707249245 1.74E-15 2.43E-14 24.21509276 UP TUBA1C

ALDH3B1 1.333698964 3.267888089 8.702937032 1.79E-15 2.50E-14 24.30467093 UP ALDH3B1

ANKS6 1.955930523 2.791361603 8.697936029 1.85E-15 2.57E-14 24.31995773 UP ANKS6

CCNE2 1.442462274 0.161857704 8.696612393 1.86E-15 2.59E-14 24.51500254 UP CCNE2

ASAP1 1.065469753 4.401539413 8.687931764 1.96E-15 2.73E-14 24.12131375 UP ASAP1

RP4_680D5.8 -1.990714443 -0.800841645 -8.687167023 1.97E-15 2.74E-14 24.51681595 DOWN RP4-680D5.8

CDC25B 1.17952028 5.191692334 8.684030598 2.01E-15 2.80E-14 24.06223171 UP CDC25B

RP11_729I10.3 -2.089954919 -4.355987738 -8.68324777 2.02E-15 2.81E-14 24.5168627 DOWN RP11-729I10.3

ATP1B3 1.199660833 4.496738997 8.674483309 2.14E-15 2.96E-14 24.03379448 UP ATP1B3

C12orf75 2.497986886 2.244349564 8.67444042 2.14E-15 2.96E-14

24.22225856 UP C12orf75

RASAL1 3.1010638 -1.611324505 8.674120493 2.14E-15 2.96E-14 24.42949518 UP RASAL1

CYP7B1 -1.807565744 2.203242639 -8.673560304 2.15E-15 2.97E-14 24.27685414 DOWN CYP7B1

DHRS4L2 -1.143485888 4.367532216 -8.671199953 2.18E-15 3.01E-14 24.03247387 DOWN DHRS4L2

ACOT4 -1.357565613 3.130608724 -8.670825681 2.19E-15 3.02E-14 24.15527773 DOWN ACOT4

CLDN18 2.711495742-3.781096065 8.670704648 2.19E-15 3.02E-14 24.43612493 UP CLDN18

LRRC3_AS1 -2.045786513 -3.624969133 -8.669437681 2.20E-15 3.04E-14 24.43261278 DOWN LRRC3-AS1

SLC52A2 1.083287492 4.549379629 8.669040224 2.21E-15 3.05E-14 23.99607691 UP SLC52A2

DMBX1 2.652362254 -4.504251802 8.668746358 2.21E-15 3.05E-14 24.42724965 UP DMBX1

AC004862.6-4.211183428 0.404562439 -8.6668182 2.24E-15 3.09E-14 24.37173186 DOWN AC004862.6

ZNF90 1.94598627 -1.804353584 8.663020361 2.29E-15 3.16E-14 24.37312593 UP ZNF90

ITIH3 -2.658742794 9.692664372 -8.662163906 2.31E-15 3.17E-14 23.94252951 DOWN ITIH3

BNIP3P11 1.674920632 -2.991964485 8.65611886 2.40E-15 3.29E-14 24.34603506 UP BNIP3P11

SNHG4 1.877516086 -1.30807511 8.655472315 2.40E-15 3.30E-14 24.31664114UP SNHG4

WNK3 -2.272650998 2.486415851 -8.64926898 2.50E-15 3.42E-14 24.11260745DOWN WNK3

DISP1 -1.305992651 3.452840946 -8.647823543 2.52E-15 3.45E-14 23.97816022 DOWN DISP1

RP11_483P21.6 -2.361060016 -4.152887203 -8.647196707 2.53E-15 3.46E-14 24.29745963 DOWN RP11-483P21.6

HNF4A_AS1	-3.523725885	0.720650007	-8.646432037	2.54E-15	3.48E-14	24.22759477	DOWN	HNF4A-AS1
GPR160	1.405571377	1.344642898	8.645818621	2.55E-15	3.49E-14	24.12610883	UP	GPR160
PHYHD1	-2.732647474	2.99633616	-8.644482133	2.58E-15	3.52E-14	24.05115043	DOWN	PHYHD1
TMEM47	-1.601645431	3.418408082	-8.643486267	2.59E-15	3.54E-14	23.9675957	DOWN	TMEM47
RP11_115J16.1	-3.389599502	-0.124291571	-8.63678452	2.70E-15	3.68E-14	24.19907261	DOWN	RP11-115J16.1
AMBP	-2.201771581	11.85864546	-8.631838811	2.79E-15	3.79E-14	23.81062216	DOWN	AMBP
AC005592.2	1.85516781	-4.135329159	8.630129411	2.82E-15	3.83E-14	24.19277531	UP	AC005592.2
ARHGAP39	1.19137822	1.740281284	8.62719347	2.87E-15	3.90E-14	23.98124777	UP	ARHGAP39
ZNF283	1.146621597	0.728476551	8.625450714	2.90E-15	3.94E-14	24.0483953	UP	ZNF283
CTD_2184C24.2	-1.496079875	-1.774094016	-8.625031311	2.91E-15	3.95E-14	24.15397431	DOWN	CTD-2184C24.2
DLL3	2.266052685	-4.824394418	8.622707703	2.95E-15	4.01E-14	24.14825517	UP	DLL3
MLXIPL	-1.801352609	7.566827302	-8.619986923	3.00E-15	4.07E-14	23.64983597	DOWN	MLXIPL
MSRB1	-1.000858387	6.636970449	-8.61917287	3.01E-15	4.09E-14	23.64012302	DOWN	MSRB1
ITM2C	1.509947685	5.141205245	8.616874839	3.06E-15	4.14E-14	23.6530662	UP	ITM2C
RP11_567G11.1	2.733830091	-3.926824745	8.616080082	3.07E-15	4.16E-14	24.10430813	UP	RP11-567G11.1
AC008592.8	-2.098843088	-3.66866177	-8.615085119	3.09E-15	4.19E-14	24.10226894	DOWN	AC008592.8
C1QL4	2.77064386	-3.499599215	8.61401494	3.11E-15	4.21E-14			

24.08883876 UP C1QL4
NR3C2 -1.512452087 2.342576084 -8.608940298 3.21E-15 4.34E-14
23.85998501 DOWN NR3C2
PEPD -1.025599292 7.083759236 -8.60354725 3.32E-15 4.49E-14
23.54466902 DOWN PEPD
KMO -2.457054283 3.206426745 -8.594919425 3.51E-15 4.73E-14
23.71647636 DOWN KMO
F5 -1.873684157 8.327976729 -8.591887273 3.57E-15 4.82E-14
23.48565364 DOWN F5
TMC6 1.61195643 3.784093108 8.590751052 3.60E-15 4.85E-14
23.57291268 UP TMC6
CTC_490E21.11 -2.305921729 0.544851282 -8.59042694 3.61E-15 4.85E-14 23.88194756 DOWN CTC-490E21.11
ATP11C -1.281758913 4.261120113 -8.590355096 3.61E-15 4.85E-14
23.54854139 DOWN ATP11C
ZCCHC6 -1.038304925 4.823118954 -8.589903555 3.62E-15 4.86E-14
23.50174585 DOWN ZCCHC6
MTND4P20 -3.29429295 1.415513494 -8.58859927 3.65E-15 4.90E-14
23.8367685 DOWN MTND4P20
CCDC152 -1.519516902 3.22342638 -8.586768439 3.69E-15 4.95E-14
23.63305081 DOWN CCDC152
HNRNPA1P10 1.39145381 -2.548888214 8.586350106 3.70E-15 4.96E-14 23.91923445 UP HNRNPA1P10
PSPH 1.126140874 3.969369131 8.586249138 3.70E-15 4.96E-14
23.5250713 UP PSPH
STEAP1B 2.147990533 -3.137321966 8.576027432 3.94E-15 5.29E-14 23.85811832 UP STEAP1B
RP11_903H12.5 -1.740643309 -0.49266412 -8.574597172 3.98E-15 5.33E-14 23.82022266 DOWN RP11-903H12.5
ORAI2 1.294397234 2.793228422 8.570497173 4.08E-15 5.46E-14
23.53391162 UP ORAI2
SLC6A14 3.299876702 -4.221559696 8.568212537 4.14E-15 5.53E-14 23.81319599 UP SLC6A14

BORA 1.134232742 0.260764523 8.568059347 4.14E-15 5.53E-14 23.72558236 UP BORA

SERPINH1 1.0811974946.340319285 8.565960412 4.20E-15 5.60E-14 23.31704812 UP SERPINH1

DDIAS 1.2952251130.521565875 8.561439373 4.32E-15 5.75E-14 23.66859852 UP DDIAS

RP11_529E10.6 1.5176477 -1.743277482 8.560137569 4.35E-15 5.79E-14 23.74760294 UP RP11-529E10.6

SDS -4.094156255 5.846299408 -8.556916937 4.44E-15 5.91E-14 23.33318238 DOWN SDS

HIC2 1.33545005 2.242183953 8.552537215 4.56E-15 6.06E-14 23.47608017 UP HIC2

NBPF13P -2.80556759 0.128171248 -8.549864799 4.64E-15 6.16E-14 23.65657404 DOWN NBPF13P

RP11_350G8.5 -1.383636676 -1.379038721 -8.54872976 4.67E-15 6.20E-14 23.68365722 DOWN RP11-350G8.5

REEP2 2.235779305 -1.49648037 8.548185624 4.69E-15 6.21E-14 23.66500229 UP REEP2

RP11_114N19.3 -1.529301656 -3.0927983 -8.547669613 4.70E-15 6.23E-14 23.69313637 DOWN RP11-114N19.3

ACOT6 -2.183587934 -2.89014248 -8.547486474 4.71E-15 6.23E-14 23.69091285 DOWN ACOT6

RP11_1293J14.1 -1.462671699 -0.619722082 -8.542777303 4.85E-15 6.41E-14 23.62890238 DOWN RP11-1293J14.1

TMEM51_AS1 2.522759758 -2.513704671 8.541324775 4.89E-15 6.46E-14 23.63911969UP TMEM51-AS1

MPPED1 -3.469288437 1.557101628 -8.539531161 4.94E-15 6.53E-14 23.53034106 DOWN MPPED1

INS_IGF2 -3.718721409 -3.771404784 -8.536003629 5.05E-15 6.67E-14 23.62202836 DOWN INS-IGF2

CDCP1 2.562585669 0.330932966 8.533720134 5.13E-15 6.75E-14 23.49827829 UP CDCP1

LYPD6B 3.481675827 -2.738202398 8.532297007 5.17E-15 6.80E-

14 23.58077105 UP LYPD6B

PTHLH 2.857060846 -0.990706389 8.531032346 5.21E-15 6.85E-14 23.54269718 UP PTHLH

ZNF124 1.219082505 0.705887133 8.52814666 5.31E-15 6.96E-14 23.45448686 UP ZNF124

ATOH8 -2.48635232 2.852557244 -8.527391105 5.33E-15 6.99E-14 23.33914919 DOWN ATOH8

CIB2 1.790877243 0.348255151 8.520747911 5.55E-15 7.28E-14 23.42506062 UP CIB2

KREMEN2 2.00388987 -2.824958035 8.513860906 5.80E-15 7.58E-14 23.47911454 UP KREMEN2

ZNF71 1.103372117 1.696416673 8.513231521 5.82E-15 7.60E-14 23.2885584 UP ZNF71

STX1B -1.623315159 1.385941468 -8.513134547 5.82E-15 7.60E-14 23.35432853 DOWN STX1B

KHDC1 2.111994541 -3.561913702 8.512950412 5.83E-15 7.60E-14 23.479308 UP KHDC1

ZNF488 1.924631586 -4.173473897 8.510722379 5.91E-15 7.70E-14 23.46934889 UP ZNF488

CCT6B -1.534616501 0.461258198 -8.508421363 6.00E-15 7.81E-14 23.37857576 DOWN CCT6B

LINC00261 -1.888621764 6.53965794 -8.507365445 6.03E-15 7.85E-14 22.95809046 DOWN LINC00261

TOB1 -1.259180263 6.431143847 -8.506459601 6.07E-15 7.89E-14 22.94967997 DOWN TOB1

PTPRB -1.329708578 3.834600909 -8.506370138 6.07E-15 7.89E-14 23.0731911 DOWN PTPRB

RP11_498C9.15 1.154444342 0.230914619 8.504083954 6.16E-15 8.00E-14 23.33673157 UP RP11-498C9.15

RP11_210M15.2 3.079598059 -3.293859284 8.502895649 6.20E-15 8.05E-14 23.41178103 UP RP11-210M15.2

HIST3H2BB 2.404476731 -4.253627203 8.502071194 6.24E-15 8.09E-14 23.4161947 UP HIST3H2BB

RP11_230F18.5 1.540313023 -2.871443663 8.497377829 6.42E-15
8.31E-14 23.38250848 UP RP11-230F18.5
ERG -1.074912748 2.669266087 -8.495386305 6.50E-15 8.41E-14
23.12203854 DOWN ERG
DAND5 1.965852175 -3.872978856 8.495319125 6.50E-15 8.41E-14
23.37503204 UP DAND5
KB_1460A1.1 2.195593336 -4.153248233 8.494240524 6.55E-15
8.46E-14 23.36913226 UP KB-1460A1.1
SERPINA6 -1.965572109 8.110490745-8.49167153 6.65E-15 8.58E-14
22.86800961 DOWN SERPINA6
ZNF681 2.072704866 -0.670160226 8.491524865 6.66E-15 8.59E-14
23.29611632UP ZNF681
NAT1 -1.030564363 1.805203993 -8.488313133 6.79E-15 8.75E-14
23.16139889 DOWN NAT1
RHOV 3.237591323 -2.311912731 8.486572331 6.86E-15 8.84E-14
23.29913443 UP RHOV
GOLGA2P7 1.545348073 -2.459643956 8.483509364 6.99E-15 9.00E-
14 23.29438852 UP GOLGA2P7
DUSP1 -1.514624295 7.115603378-8.481299786 7.09E-15 9.12E-14
22.79566788 DOWN DUSP1
PROC -2.168515896 7.609087797 -8.477152634 7.27E-15 9.35E-14
22.77458943 DOWN PROC
MIR621 -2.439587039 1.963808003 -8.475590832 7.35E-15 9.43E-14
23.0968832 DOWN MIR621
RP11_181C3.1 -1.789201738 0.08491847 -8.474881225 7.38E-15 9.47E-
14 23.19420723 DOWN RP11-181C3.1
ZC2HC1A 1.4403115570.7114538 8.47305307 7.46E-15 9.57E-14
23.11655653UP ZC2HC1A
TLDC2 1.9254811550.435304712 8.471708685 7.52E-15 9.64E-14
23.12058175 UP TLDC2
ARSD -1.140636018 5.54545516 -8.468492808 7.67E-15 9.83E-14
22.73062105 DOWN ARSD
NKAIN1 2.288823574 -4.329528424 8.466884151 7.75E-15 9.92E-

14 23.20447343 UP NKAIN1

FAM111B 1.471820151 1.740070358 8.461437465 8.02E-15 1.03E-13 22.96545165 UP FAM111B

SNX25P1 1.611362101-1.968932929 8.458633669 8.16E-15 1.04E-13 23.13649632 UP SNX25P1

ALDOA1.1192873098.331480247 8.457266471 8.23E-15 1.05E-13 22.67142245 UP ALDOA

RP11_136I14.5 -2.164743295 -3.658470164 -8.455826143 8.30E-15 1.06E-13 23.13928109 DOWN RP11-136I14.5

PPARGC1A -2.497777276 4.655431311-8.455655106 8.31E-15 1.06E-13 22.73607166 DOWN PPARGC1A

CCDC158 -1.70964152-0.442694309 -8.454815318 8.35E-15 1.07E-13 23.09199312 DOWN CCDC158

RP11_65J21.4 -2.621180561 -4.699417435 -8.45336168 8.43E-15 1.07E-13 23.12448141 DOWN RP11-65J21.4

PDZD7 1.602693942 -1.999740862 8.451993524 8.50E-15 1.08E-13 23.0969894 UP PDZD7

SLC26A1 -1.33266997 4.016096251 -8.451604413 8.52E-15 1.09E-13 22.7189742 DOWN SLC26A1

SLC35E4 1.31601202 0.464108398 8.449923049 8.61E-15 1.10E-13 22.99325646 UP SLC35E4

CYP2W1 2.143993029 -2.661980469 8.448842673 8.66E-15 1.10E-13 23.08332012 UP CYP2W1

SPACA6P 1.615443511-0.347505066 8.442987216 8.98E-15 1.14E-13 22.99135369 UP SPACA6P

GRIK4 2.7490173 -2.357289495 8.441946092 9.04E-15 1.15E-13 23.0333307 UP GRIK4

MYL6B 1.058258378 4.243125917 8.43245304 9.59E-15 1.22E-13 22.56331465 UP MYL6B

LINC01554 -5.474272069 1.74920678 -8.432205333 9.60E-15 1.22E-13 22.90339343 DOWN LINC01554

CNTNAP1 1.557976933 1.144185522 8.43014107 9.72E-15 1.23E-13 22.82287016 UP CNTNAP1

PSMD10P2 2.016249453 -3.783489683 8.427423899 9.89E-15 1.25E-13 22.96519285 UP PSMD10P2

MSTO2P 1.142215817 0.104145923 8.427191467 9.90E-15 1.25E-13 22.87737688 UP MSTO2P

AC024937.6 1.75024508 -3.648087476 8.425841446 9.99E-15 1.26E-13 22.95584497 UP AC024937.6

RP11_191G24.1 -1.686154466 -2.524465359 -8.41835064 1.05E-14 1.32E-13 22.9105948 DOWN RP11-191G24.1

ABHD2 -1.215787343 7.274868042 -8.415559416 1.06E-14 1.34E-13 22.39462433 DOWN ABHD2

RP11_875O11.3 2.259117125 -2.769449802 8.414767301 1.07E-14 1.35E-13 22.87906333 UP RP11-875O11.3

PAQR4 1.409626768 1.922865685 8.412533564 1.08E-14 1.37E-13 22.65211757 UP PAQR4

SNHG12 1.01275245 2.267574815 8.406194681 1.13E-14 1.42E-13 22.58406944 UP SNHG12

CTC_463A16.1 -1.041677684 -0.1490273 -8.405369737 1.13E-14 1.43E-13 22.7767679 DOWN CTC-463A16.1

ITPR3 2.450226646 2.107564423 8.403987492 1.14E-14 1.44E-13 22.57916301 UP ITPR3

CPB2_AS1 -1.460373924 -0.574509466 -8.401783894 1.16E-14 1.45E-13 22.77384282 DOWN CPB2-AS1

SLC38A3 -2.237189705 8.13872206 -8.401333646 1.16E-14 1.46E-13 22.31720929 DOWN SLC38A3

MROH8 -1.111934806 0.244620809 -8.400608465 1.17E-14 1.46E-13 22.73113864 DOWN MROH8

PAPPA2 -3.926317801 0.029803084 -8.399573865 1.17E-14 1.47E-13 22.75767492 DOWN PAPPA2

SLC39A8 -1.32630769 4.460719993 -8.398746012 1.18E-14 1.48E-13 22.36246482 DOWN SLC39A8

LAMP5 3.079501015 -1.807221942 8.397293941 1.19E-14 1.49E-13 22.75239599 UP LAMP5

SELM 1.910284507 4.1158883 8.395568747 1.20E-14 1.50E-13

22.35928624 UP SELM

ST6GAL1 -1.33684284 8.829347342 -8.394419147 1.21E-14 1.51E-13 22.28691805 DOWN ST6GAL1

LINC01588 -1.507096508 0.953960226 -8.390312702 1.24E-14 1.55E-13 22.63519261 DOWN LINC01588

SMOX 1.493271305 2.9648504 8.38968431 1.25E-14 1.56E-13 22.41443502 UP SMOX

DGUOK_AS1 1.225955484 -2.052041617 8.38677209 1.27E-14 1.58E-13 22.70693093 UP DGUOK-AS1

KIF3C 1.604512256 1.44648242 8.381582153 1.31E-14 1.63E-13 22.50465521 UP KIF3C

TLL2 1.976947137 -2.535259815 8.376275854 1.36E-14 1.68E-13 22.64595612 UP TLL2

SEMA3E 3.825364385 -3.291433807 8.374378132 1.37E-14 1.70E-13 22.63129364 UP SEMA3E

AC006994.3 -2.156007475 -4.338314316 -8.373124536 1.38E-14 1.71E-13 22.64260738 DOWN AC006994.3

UPK1A_AS1 2.574948444 -4.106900393 8.373006675 1.38E-14 1.72E-13 22.63780711 UP UPK1A-AS1

SPIB 2.772393598 -1.528133467 8.371648403 1.39E-14 1.73E-13 22.59422751 UP SPIB

4-Sep -1.985184978 4.352074459 -8.370930113 1.40E-14 1.74E-13 22.22028616 DOWN 4-Sep

OASL -2.022699857 3.496391701 -8.369426517 1.41E-14 1.75E-13 22.29761482 DOWN OASL

DBNDD2 1.397229811 -0.653092951 8.365386787 1.45E-14 1.79E-13 22.5374234 UP DBNDD2

SEL1L3 2.002978864 4.195862757 8.365334073 1.45E-14 1.79E-13 22.17176817 UP SEL1L3

RP11_21L23.2 -2.822203977 2.055088392 -8.363040065 1.47E-14 1.82E-13 22.4148077 DOWN RP11-21L23.2

RAD54B 1.018101477 0.427791715 8.361448678 1.48E-14 1.83E-13 22.46256763 UP RAD54B

CRMP1 1.95616112 1.60377317 8.360510698 1.49E-14 1.84E-13 22.35975416 UP CRMP1

AC004166.6 -1.188572922 -0.772970336 -8.360480278 1.49E-14 1.84E-13 22.52966141 DOWN AC004166.6

FAM110A 1.087073171 2.337235399 8.359192362 1.51E-14 1.86E-13 22.29142062 UP FAM110A

CMTM3 1.217552287 3.677920247 8.352286725 1.57E-14 1.93E-13 22.12096312 UP CMTM3

EPHB3 2.272650839 0.116751489 8.349686076 1.60E-14 1.96E-13 22.39742338 UP EPHB3

ASS1P1 -1.603935181 -0.8471274 -8.349535111 1.60E-14 1.96E-13 22.46854071 DOWN ASS1P1

ASGR1 -2.085614695 8.424151821 -8.348208501 1.61E-14 1.98E-13 21.99796552 DOWN ASGR1

FMO5 -2.040693643 7.262730283 -8.343596518 1.66E-14 2.03E-13 21.95875808 DOWN FMO5

TNFRSF21 1.583293668 4.349047479 8.341253854 1.68E-14 2.06E-13 22.00851254 UP TNFRSF21

HAO2_IT1 -2.266446896 -4.756392297 -8.340675666 1.69E-14 2.07E-13 22.44804139 DOWN HAO2-IT1

ANXA6 -1.207522569 7.327714656 -8.33856767 1.71E-14 2.09E-13 21.92671969 DOWN ANXA6

REN -3.139488059 -1.012013534 -8.338516987 1.71E-14 2.09E-13 22.41321855 DOWN REN

GNAZ 2.479981041 1.950599398 8.337944305 1.71E-14 2.10E-13 22.19164256 UP GNAZ

MTCL1 2.261472167 1.44565582 8.336754701 1.73E-14 2.11E-13 22.22696936 UP MTCL1

USH2A -2.37172501 1.372018451 -8.3332472 1.77E-14 2.15E-13 22.27508829 DOWN USH2A

RP11_94A24.1 -2.480127652 -3.940850477 -8.332714101 1.77E-14 2.16E-13 22.40032651 DOWN RP11-94A24.1

CCNI2 1.881865377 -3.044975633 8.331771419 1.78E-14 2.17E-13

22.38546053 UP CCNI2

ERVMER34_1 3.020189085 -2.69603984 8.330465782 1.80E-14 2.19E-13 22.36562349 UP ERVMER34-1

C1orf168 -2.254814083 2.163633561 -8.326875339 1.84E-14 2.24E-13 22.17525261 DOWN C1orf168

CIDEB -1.827543562 -1.500711258 -8.326753896 1.84E-14 2.24E-13 22.34838835 DOWN CIDEB

OFCC1 2.314141761 -5.311384967 8.325551449 1.85E-14 2.25E-13 22.35714219 UP OFCC1

PPAP2B -1.252096776 6.120038424 -8.323661344 1.87E-14 2.28E-13 21.83865513 DOWN PPAP2B

PAPLN 2.51024479 2.192639136 8.32215104 1.89E-14 2.30E-13 22.0764005 UP PAPLN

TMEM110 -1.090772471 2.761261679 -8.319113713 1.92E-14 2.34E-13 22.03995475 DOWN TMEM110

CTC_505O3.2 -4.115660887 0.563109058 -8.315533151 1.97E-14 2.39E-13 22.23324005 DOWN CTC-505O3.2

IL20RA 3.539874809 -2.985925224 8.315485966 1.97E-14 2.39E-13 22.27550793 UP IL20RA

SERPINF1 -1.753994667 9.085708436 -8.31499119 1.97E-14 2.39E-13 21.80855618 DOWN SERPINF1

PRAP1 -2.946226706 7.725851943 -8.302145711 2.14E-14 2.59E-13 21.71341365 DOWN PRAP1

LINC01344 -2.548829406 -2.405066742 -8.300720481 2.15E-14 2.61E-13 22.20319861 DOWN LINC01344

C2orf48 1.836232905 -3.152560802 8.296806505 2.21E-14 2.67E-13 22.17749194 UP C2orf48

CTD_2240J17.1 -1.814319213 -3.576722774 -8.296412529 2.21E-14 2.67E-13 22.18327954 DOWN CTD-2240J17.1

ELF4 1.536563338 1.968650024 8.295925514 2.22E-14 2.68E-13 21.94072706 UP ELF4

SMOC1 -1.436153992 5.989705246 -8.294619753 2.24E-14 2.70E-13 21.66579131 DOWN SMOC1

GREM2 -4.663496309 1.51379364 -8.29325235 2.26E-14 2.72E-13 22.060546 DOWN GREM2

EPS8L1 2.569912314 -0.099579432 8.29309735 2.26E-14 2.72E-13 22.065925 UP EPS8L1

FCN2 -3.49056364 -1.466530632 -8.292225354 2.27E-14 2.73E-13 22.1425311 DOWN FCN2

CISH -1.347008692 4.614668066 -8.292078926 2.27E-14 2.74E-13 21.70366716 DOWN CISH

TMOD1 -2.160952749 2.556673888 -8.290859516 2.29E-14 2.76E-13 21.92027946 DOWN TMOD1

RP11_496H1.1 1.937265871 -2.814378733 8.286941958 2.34E-14 2.82E-13 22.11437487 UP RP11-496H1.1

GLIS2 1.746439075 2.678770255 8.286061051 2.36E-14 2.83E-13 21.81393167 UP GLIS2

TRAM1L1 2.644671156 -2.186871945 8.279548964 2.45E-14 2.95E-13 22.0558024 UP TRAM1L1

RP11_266L9.8 -1.573766772 0.689675919 -8.278305467 2.47E-14 2.97E-13 21.97555497 DOWN RP11-266L9.8

MICAL1 1.2134564 3.448842731 8.27623541 2.50E-14 3.00E-13 21.68057152 UP MICAL1

VWF -1.576060971 5.866678627 -8.275722379 2.51E-14 3.01E-13 21.55527839 DOWN VWF

OTX1 2.452991134 -1.013427745 8.275553228 2.51E-14 3.01E-13 22.00389734 UP OTX1

AF127936.9 -1.146303758 1.131310847 -8.274041901 2.54E-14 3.04E-13 21.91699674 DOWN AF127936.9

ZPLD1 3.433376307 -3.634939074 8.272284586 2.56E-14 3.07E-13 22.02634551 UP ZPLD1

AURKA 1.29808463 3.489446552 8.272053266 2.57E-14 3.07E-13 21.65230813 UP AURKA

CTC_459F4.9 1.345605135 -1.706277089 8.270672491 2.59E-14 3.09E-13 22.00259528 UP CTC-459F4.9

ZNF85 1.533589811 -0.392476487 8.266866285 2.65E-14 3.16E-13

21.93238913 UP ZNF85

RP11_363N22.3 1.580894143 -3.231507802 8.262543947 2.72E-14 3.24E-13 21.97499782 UP RP11-363N22.3

C7orf31 1.528602962 0.766753724 8.262182734 2.73E-14 3.25E-13 21.83654044 UP C7orf31

TM7SF2 -1.763647176 6.766803219 -8.261990394 2.73E-14 3.25E-13 21.4630729 DOWN TM7SF2

SH3BGRL2 -1.235316453 5.584313299 -8.261393637 2.74E-14 3.26E-13 21.47177626 DOWN SH3BGRL2

NEDD4 -1.296284192 4.881609943 -8.260349442 2.76E-14 3.28E-13 21.49478125 DOWN NEDD4

FCHO1 1.878415316 0.268823694 8.259660392 2.77E-14 3.29E-13 21.85020548 UP FCHO1

RP11_407B7.3 -1.892503034 -3.987033307 -8.257319596 2.81E-14 3.34E-13 21.950387 DOWN RP11-407B7.3

FYN -1.019784827 4.192751558 -8.254480402 2.86E-14 3.39E-13 21.49985246 DOWN FYN

RP11_141C7.3 1.652020234 -4.051640198 8.252982196 2.89E-14 3.42E-13 21.92288554 UP RP11-141C7.3

AP001065.7 -2.195447282 -4.129769149 -8.252373499 2.90E-14 3.43E-13 21.92085923 DOWN AP001065.7

ACSS1 1.639822457 3.441633057 8.251924675 2.90E-14 3.44E-13 21.53844599 UP ACSS1

TTC39C -1.310433498 6.518070838 -8.25105148 2.92E-14 3.45E-13 21.39594192 DOWN TTC39C

RP11_1136G11.8 -1.960416759 -3.841984124 -8.251035936 2.92E-14 3.45E-13 21.91284331 DOWN RP11-1136G11.8

FMO2 -2.100603637 0.327631751 -8.250067694 2.94E-14 3.47E-13 21.83099673 DOWN FMO2

RP11_485O10.2 -2.749035124 -4.076228962 -8.24973771 2.94E-14 3.48E-13 21.90483602 DOWN RP11-485O10.2

TMEM150C -1.713120123 3.645170366 -8.246946237 2.99E-14 3.53E-13 21.52872791 DOWN TMEM150C

MAN1C1 -1.712198955 3.386823252 -8.245617004 3.02E-14 3.56E-13 21.54891957 DOWN MAN1C1
SFXN5 -1.04555413 5.489316426 -8.244919832 3.03E-14 3.58E-13 21.37292664 DOWN SFXN5
CDNF -1.153711999 0.991787615 -8.244055534 3.05E-14 3.59E-13 21.74629918 DOWN CDNF
UPK3A 3.865078055 -1.748160785 8.240757351 3.11E-14 3.66E-13 21.80072012 UP UPK3A
RP11_70D24.2 -1.514416293 -0.510996373 -8.240315599 3.12E-14 3.67E-13 21.80203626 DOWN RP11-70D24.2
CD24 3.46845357 5.059747986 8.237866628 3.16E-14 3.72E-13 21.38254337 UP CD24
RN7SL8P 1.97094382 -5.266325448 8.237444933 3.17E-14 3.73E-13 21.83154641 UP RN7SL8P
ZNF888 2.095368693 -3.727220273 8.23651053 3.19E-14 3.75E-13 21.82127275 UP ZNF888
OBSCN 2.316629049 1.63284735 8.235510041 3.21E-14 3.77E-13 21.60055438 UP OBSCN
SLC1A1 -2.639708678 4.13606206 -8.234655432 3.23E-14 3.78E-13 21.44932721 DOWN SLC1A1
RP11_219C24.6 -3.438765925 -3.759308916 -8.23195777 3.28E-14 3.84E-13 21.79767074 DOWN RP11-219C24.6
TIE1 -1.089683636 4.312214434 -8.231939199 3.28E-14 3.84E-13 21.3564016 DOWN TIE1
AC004076.5 1.344478418 -2.110658633 8.225447365 3.41E-14 3.99E-13 21.74096573 UP AC004076.5
AC144652.1 -1.501157317 0.51574211 -8.225438069 3.41E-14 3.99E-13 21.66756933 DOWN AC144652.1
PTCHD4 -2.654329658 -0.908628185 -8.223695779 3.45E-14 4.03E-13 21.72203317 DOWN PTCHD4
RP11_111M22.4 1.445196197 -3.139042699 8.220395919 3.52E-14 4.11E-13 21.72345494 UP RP11-111M22.4
HLF -2.265806468 5.506680253 -8.220382511 3.52E-14 4.11E-13

21.24348367 DOWN HLF
CTD_2561J22.5 2.127337565 -2.52743575 8.21953584 3.54E-14 4.13E-13
21.70661887 UP CTD-2561J22.5
RP11_72M17.1 -1.735232229 -2.741965911 -8.217278233 3.59E-14
4.18E-13 21.70889394 DOWN RP11-72M17.1
CDCA7L 1.646320076 1.908797768 8.216279636 3.61E-14 4.20E-
13 21.46544362 UP CDCA7L
NAGS -2.401211695 3.880130467 -8.216061829 3.62E-14 4.21E-13
21.3446076 DOWN NAGS
HAVCR1 3.332653194 -3.308839515 8.212035111 3.71E-14 4.30E-13
21.66318331 UP HAVCR1
RARRES2 -1.834111482 9.126834302 -8.209897251 3.75E-14 4.36E-
13 21.17413638 DOWN RARRES2
TAT_AS1 -2.069426835 -1.6719156 -8.208665328 3.78E-14 4.38E-13
21.6463601 DOWN TAT-AS1
SAA1 -4.441425925 6.360413883 -8.20684688 3.82E-14 4.43E-13
21.18529984 DOWN SAA1
ZNF43 1.648659656 1.003982619 8.206193439 3.84E-14 4.44E-13
21.48164321 UP ZNF43
TMPRSS6 -2.221387981 6.782039511 -8.203682777 3.90E-14 4.51E-13
21.11422647 DOWN TMPRSS6
SYT8 3.2934615 -1.922603252 8.20260765 3.92E-14 4.54E-13
21.58468922 UP SYT8
BNIP3P17 1.917897764 -4.881895024 8.198590213 4.02E-14 4.64E-
13 21.60048697 UP BNIP3P17
AP000648.5 1.102678953 -0.240510724 8.1969064 4.06E-14 4.69E-13
21.50990898 UP AP000648.5
TRAM2_AS1 -1.009346563 2.610303508 -8.193902243 4.14E-14
4.77E-13 21.3013144 DOWN TRAM2-AS1
RDM1 1.916031774 -1.56732924 8.193429082 4.15E-14 4.78E-13
21.53388467 UP RDM1
PRR36 2.205733632 -0.314717557 8.192270317 4.18E-14 4.81E-13
21.47607194 UP PRR36

SALL4 3.054866419 -1.776228934 8.192068412 4.18E-14 4.81E-13 21.52131029 UP SALL4

RP11_883G14.2 -2.08060378 -3.877718607 -8.191823885 4.19E-14 4.82E-13 21.560657 DOWN RP11-883G14.2

SERPINA11 -3.220188994 5.649412224 -8.188017067 4.29E-14 4.93E-13 21.06580733 DOWN SERPINA11

DKFZp779M0652 -2.049535293 0.672077605 -8.187887847 4.29E-14 4.93E-13 21.44035628 DOWN DKFZp779M0652

SLFN13 2.488031349 1.214817524 8.187068362 4.31E-14 4.95E-13 21.34230879 UP SLFN13

FHOD3 2.98105288 -0.973756793 8.185769199 4.35E-14 4.98E-13 21.461577 UP FHOD3

RP11_332H14.1 -1.523241024 -2.087836574 -8.185154909 4.36E-14 5.00E-13 21.51204949 DOWN RP11-332H14.1

FNDC5 -4.020959876 2.285295773 -8.183640288 4.41E-14 5.04E-13 21.34584945 DOWN FNDC5

DMKN 3.931620303 0.372498508 8.179000581 4.53E-14 5.19E-13 21.34244332 UP DMKN

BMX -1.739243887 -1.353389457 -8.177679126 4.57E-14 5.22E-13 21.45456705 DOWN BMX

ALDH3A2 -1.188360307 7.863887816 -8.173194024 4.69E-14 5.36E-13 20.93296202 DOWN ALDH3A2

FGB -2.734551126 11.71512064-8.171959335 4.73E-14 5.39E-13 21.00664943 DOWN FGB

SGPP2 2.930906759 -0.825508029 8.169347678 4.81E-14 5.48E-13 21.35741084 UP SGPP2

MYCL -1.654598823 3.523751861 -8.167719507 4.85E-14 5.52E-13 21.0620483 DOWN MYCL

MYCT1 -1.020034929 2.356253892 -8.167469992 4.86E-14 5.53E-13 21.16989356 DOWN MYCT1

MTL5 1.714878026 0.588326858 8.156462238 5.20E-14 5.90E-13 21.21303118UP MTL5

GCSH -1.015547961 2.936876162 -8.152416617 5.33E-14 6.05E-13

21.01492091 DOWN GCSH
GPIHBP1 -1.56857032 1.523871254 -8.151253102 5.36E-14 6.08E-13 21.15825153 DOWN GPIHBP1
MAOB -1.568633723 7.371073853 -8.145584565 5.55E-14 6.29E-13 20.76238334 DOWN MAOB
ARMC9 1.290575298 1.162329701 8.144645236 5.58E-14 6.32E-13 21.10518226 UP ARMC9
RP11_303E16.8 -1.677750271 -2.509863225 -8.143246664 5.63E-14 6.37E-13 21.26782468 DOWN RP11-303E16.8
FRMD5 1.957269636 -4.552754621 8.141155468 5.70E-14 6.45E-13 21.25943424 UP FRMD5
SLC17A4 -2.133710738 4.661116125 -8.140510752 5.73E-14 6.47E-13 20.81111129 DOWN SLC17A4
BOLA3_AS1 1.534489595 -1.920095015 8.136975872 5.85E-14 6.61E-13 21.20948481 UP BOLA3-AS1
CYP26B1 2.67649456 0.02471756 8.136801852 5.86E-14 6.61E-13 21.12170052 UP CYP26B1
SYTL4 -1.501577685 3.625665389 -8.135424297 5.91E-14 6.67E-13 20.85446915 DOWN SYTL4
CREG1 -1.123196335 7.482765298 -8.13390173 5.96E-14 6.72E-13 20.69279486 DOWN CREG1
UQCRQ -1.140673765 7.805756716 -8.133654254 5.97E-14 6.73E-13 20.69477763 DOWN UQCRQ
THRSP -5.469533385 1.855567294 -8.133149122 5.99E-14 6.75E-13 21.09786904 DOWN THRSP
BOK_AS1 -2.552210922 -2.912093348 -8.13263862 6.01E-14 6.77E-13 21.20611334 DOWN BOK-AS1
SLC29A4 2.653613503 2.465117079 8.128792744 6.15E-14 6.92E-13 20.89255046 UP SLC29A4
ILDR1 3.035028955 -1.669634712 8.126816399 6.22E-14 7.00E-13 21.13077876 UP ILDR1
C5orf30 1.925684734 0.657664153 8.125520514 6.27E-14 7.05E-13 21.02189299 UP C5orf30

CCDC34	1.029736707	2.911415704	8.125247857	6.28E-14	7.06E-13	20.8241925	UP	CCDC34
RGS17	1.623209732	-1.967927945	8.121506661	6.43E-14	7.21E-13	21.1180664	UP	RGS17
ESPN	-1.892467854	5.736375759	-8.121309215	6.43E-14	7.22E-13	20.63259652	DOWN	ESPN
ABCC1	1.51015674	3.341115903	8.120916305	6.45E-14	7.23E-13	20.75840241	UP	ABCC1
ASB4	-2.693017129	-0.336805542	-8.120324116	6.47E-14	7.25E-13	21.08924961	DOWN	ASB4
NDRG1	1.466874591	7.083580824	8.119498544	6.51E-14	7.29E-13	20.61224197	UP	NDRG1
ABTB2	-1.095659526	3.904580535	-8.119135305	6.52E-14	7.30E-13	20.71481119	DOWN	ABTB2
NFKBIE	1.105361138	3.950331849	8.118721364	6.54E-14	7.32E-13	20.68888086	UP	NFKBIE
RP11_386G11.5	1.677993388	-3.531286972	8.11725547	6.59E-14	7.37E-13	21.11355501	UP	RP11-386G11.5
SPINT1	3.441150525	2.244304876	8.115309257	6.67E-14	7.46E-13	20.83537674	UP	SPINT1
CYP39A1	-3.175445175	2.500274249	-8.113861185	6.73E-14	7.52E-13	20.88868225	DOWN	CYP39A1
KIAA1522	1.026978015	5.839903404	8.106770028	7.03E-14	7.85E-13	20.53412363	UP	KIAA1522
NUBPL	-1.049493094	3.221178273	-8.103312464	7.18E-14	8.01E-13	20.69052762	DOWN	NUBPL
SPTSSB	2.809063046	-2.26831428	8.103019563	7.19E-14	8.02E-13	21.00391792	UP	SPTSSB
E2F5	1.138643267	2.072562928	8.093149555	7.63E-14	8.47E-13	20.71707031	UP	E2F5
RP11_398K22.12	1.737087393	-2.647197723	8.08752774	7.90E-14	8.76E-13	20.92755159	UP	RP11-398K22.12
ANKRD13B	1.349635159	0.75081094	8.083892636	8.07E-14	8.95E-13			

20.77307105 UP ANKRD13B

DHX58 -1.06708803 3.940154786 -8.083750148 8.08E-14 8.95E-13 20.49860751 DOWN DHX58

RP11_138H11.1 -2.29289311 -4.923817151 -8.08078822 8.23E-14 9.11E-13 20.90318807 DOWN RP11-138H11.1

CBX6 1.395261598 3.965880415 8.080330567 8.25E-14 9.13E-13 20.4616901 UP CBX6

CRHBP -2.941611594 0.067986529 -8.079059656 8.31E-14 9.20E-13 20.83097733 DOWN CRHBP

IRF6 -2.147300443 5.56517268 -8.075875064 8.47E-14 9.37E-13 20.37258508 DOWN IRF6

MLIP -2.678991006 2.570805445 -8.075546233 8.49E-14 9.38E-13 20.64103176 DOWN MLIP

DHDH 1.886767455 -2.498486333 8.072298193 8.66E-14 9.57E-13 20.83373294 UP DHDH

PFKFB4 1.488436862 1.252800906 8.065743735 9.01E-14 9.94E-13 20.62408476 UP PFKFB4

ZNF391 2.314432408 -1.298283302 8.064734524 9.07E-14 1.00E-12 20.7583902 UP ZNF391

RP11_813N20.1 -2.983300383 0.123094048 -8.063745581 9.12E-14 1.01E-12 20.73792068 DOWN RP11-813N20.1

SLC23A2 -1.30165968 5.883581646 -8.0629848 9.16E-14 1.01E-12 20.27336623 DOWN SLC23A2

ZMAT1 -1.551908302 2.231291781 -8.062882514 9.17E-14 1.01E-12 20.57042694 DOWN ZMAT1

SH3BP4 -1.120083308 5.915528298 -8.057605017 9.46E-14 1.04E-12 20.23930242 DOWN SH3BP4

MIR135A1 -1.659325212 -0.174254637 -8.055467513 9.59E-14 1.05E-12 20.68938921 DOWN MIR135A1

CYP19A1 3.197041203 -3.234696054 8.054718474 9.63E-14 1.06E-12 20.72976333 UP CYP19A1

AOC4P -2.576758973 1.817151325 -8.054459031 9.65E-14 1.06E-12 20.57623436 DOWN AOC4P

LINC00992 2.972911553-3.443129814 8.050158637 9.90E-14 1.09E-12 20.70789337 UP LINC00992

PCSK6 -1.750873645 6.034125232 -8.049459866 9.94E-14 1.09E-12 20.19426325 DOWN PCSK6

SERPINF2 -2.236936539 9.414999284 -8.048851714 9.98E-14 1.09E-12 20.21343813 DOWN SERPINF2

UGT2B4 -2.589104393 8.481250939 -8.047695048 1.00E-13 1.10E-12 20.18958605 DOWN UGT2B4

DUSP10 -1.141961806 4.67615343 -8.047059983 1.01E-13 1.10E-12 20.22260854 DOWN DUSP10

RP11_109D9.4 -1.975519272 -3.888417674 -8.043651161 1.03E-13 1.13E-12 20.68460351 DOWN RP11-109D9.4

C18orf42 -2.107846576 -4.988890798 -8.04363827 1.03E-13 1.13E-12 20.68427559 DOWN C18orf42

LINC00324 -1.049941312 1.389268397 -8.041502828 1.04E-13 1.14E-12 20.50672973 DOWN LINC00324

CTD_2012K14.8-1.697055757 1.238505519 -8.036302519 1.08E-13 1.18E-12 20.4974403 DOWN CTD-2012K14.8

RP11_540A21.2 1.606104401 -2.018521156 8.036207863 1.08E-13 1.18E-12 20.61479382 UP RP11-540A21.2

PCYOX1L 1.085946629 1.159257752 8.035791897 1.08E-13 1.18E-12 20.4594883 UP PCYOX1L

EMILIN2 1.41032159 2.186515907 8.034002505 1.09E-13 1.19E-12 20.35049558 UP EMILIN2

CTD_2162K18.42.110111588-3.89449802 8.03111033 1.11E-13 1.21E-12 20.60576248 UP CTD-2162K18.4

CHN2 -1.430699288 4.600868645 -8.0301833 1.12E-13 1.22E-12 20.13267539 DOWN CHN2

EBPL -1.151566825 5.520710334 -8.029262358 1.12E-13 1.22E-12 20.0789724 DOWN EBPL

TEK -1.775210468 1.92919166 -8.024106981 1.16E-13 1.26E-12 20.37232923 DOWN TEK

SFXN3 1.205342427 3.412736494 8.023601023 1.16E-13 1.26E-12

20.16840584 UP SFXN3
C1orf145 1.994703429 -2.865247406 8.022746874 1.17E-13 1.27E-12 20.54595966 UP C1orf145
CCDC163P 1.03470604 0.606878386 8.022692123 1.17E-13 1.27E-12 20.42367114UP CCDC163P
AC159540.1 1.644251249 -3.828820939 8.02164534 1.18E-13 1.28E-12 20.55184957 UP AC159540.1
C6orf132 3.045084248 -0.689326513 8.021381501 1.18E-13 1.28E-12 20.47175861 UP C6orf132
KIAA1244 2.702803163 1.166469493 8.020531799 1.18E-13 1.28E-12 20.35113958UP KIAA1244
GSTO1 -1.099905013 7.72640705 -8.019924558 1.19E-13 1.29E-12 20.01375666 DOWN GSTO1
RP11_158H5.7 1.268192038 -1.312066453 8.01978633 1.19E-13 1.29E-12 20.5006471 UP RP11-158H5.7
IFT80 1.077527003 2.16318249 8.01240785 1.24E-13 1.34E-12 20.22836052 UP IFT80
SLC35F2 1.806284624 -0.129733456 8.00527263 1.30E-13 1.40E-12 20.35824793 UP SLC35F2
GAMT -1.605357779 7.679246308 -8.003558077 1.31E-13 1.42E-12 19.91571366 DOWN GAMT
MTND5P1 -2.318699941 -3.350289749 -8.002522422 1.32E-13 1.42E-12 20.4412665 DOWN MTND5P1
ZNF66 1.966628635 -2.666749727 7.999778281 1.34E-13 1.45E-12 20.40826454 UP ZNF66
APCS -2.759025861 8.560649315 -7.998059904 1.36E-13 1.46E-12 19.89508027 DOWN APCS
RP11_532F12.5 2.966550388 -2.165282866 7.995226295 1.38E-13 1.48E-12 20.36359872 UP RP11-532F12.5
CTD_3138B18.6 1.428674742 -3.319723863 7.994822575 1.38E-13 1.49E-12 20.39091863 UP CTD-3138B18.6
PDLIM1P4 -1.509906222 -2.723133696 -7.994313673 1.39E-13 1.49E-12 20.39094105 DOWN PDLIM1P4

CTD_2199O4.6 1.548977559 -3.822748065 7.992250364 1.40E-13 1.51E-12 20.37938644 UP CTD-2199O4.6
TPH1 1.827130832 -2.576113481 7.992047063 1.41E-13 1.51E-12 20.36260445 UP TPH1
LINGO4 -2.656921211 -0.837599264 -7.984732581 1.47E-13 1.58E-12 20.30337568 DOWN LINGO4
SLC2A10 -1.622614662 4.6581382 -7.981858486 1.49E-13 1.60E-12 19.84697098 DOWN SLC2A10
DOK1 1.012972018 1.731306364 7.977999562 1.53E-13 1.64E-12 20.0670741 UP DOK1
RP11_802E16.3 1.203942039 -0.750653721 7.976288028 1.55E-13 1.65E-12 20.22279709 UP RP11-802E16.3
LRCOL1 -3.63198394 0.267862319 -7.97536302 1.55E-13 1.66E-12 20.21511227 DOWN LRCOL1
RP11_66A2.2 -2.22978688 -4.097002572 -7.975077658 1.56E-13 1.66E-12 20.2816904 DOWN RP11-66A2.2
DDT -1.164105513 6.487406383 -7.973391824 1.57E-13 1.68E-12 19.73164276 DOWN DDT
FAS -1.430128123 3.175087794 -7.971893008 1.59E-13 1.69E-12 19.92653231 DOWN FAS
C8G -1.914098845 7.505121011 -7.971666953 1.59E-13 1.69E-12 19.72505999 DOWN C8G
ARMC3 1.868170229 -4.920178988 7.968161943 1.62E-13 1.73E-12 20.24070374 UP ARMC3
DSG2 2.19289249 4.002508534 7.967201424 1.63E-13 1.74E-12 19.79854096 UP DSG2
EFNA5 2.976001456 -0.725643087 7.967062382 1.63E-13 1.74E-12 20.1529543 UP EFNA5
SHPK -1.04339701 2.484871154 -7.96389813 1.67E-13 1.77E-12 19.9430288 DOWN SHPK
TMEM191C 1.416648517 -2.826020606 7.962468572 1.68E-13 1.78E-12 20.1951959 UP TMEM191C
RP11_624M8.1 -2.33095761 -2.55991667 -7.961971931 1.68E-13 1.79E-12

20.19844574 DOWN RP11-624M8.1

SH2D5 2.019609171 -3.347715173 7.961254124 1.69E-13 1.79E-12 20.19044258 UP SH2D5

RPS4XP5 -2.115098973 -4.037120782 -7.960694127 1.70E-13 1.80E-12 20.19742341 DOWN RPS4XP5

GRIN2D 1.471370826 -0.444806913 7.959067585 1.71E-13 1.82E-12 20.10493937 UP GRIN2D

ZNF611 1.468125352 0.902631848 7.95755054 1.73E-13 1.83E-12 20.01141039UP ZNF611

L3MBTL1 1.084384267 0.859613189 7.955901231 1.75E-13 1.85E-12 20.00973402 UP L3MBTL1

CCNJL 1.830222513 -0.703798481 7.947999324 1.83E-13 1.93E-12 20.04938725 UP CCNJL

IQCD 1.62619156 -0.592437261 7.947949206 1.83E-13 1.93E-12 20.04525355 UP IQCD

RAB6B 1.167940401 1.669216187 7.947801065 1.83E-13 1.94E-12 19.89224285 UP RAB6B

NECAB2 -2.953654435 1.959398633 -7.940092813 1.92E-13 2.02E-12 19.89488413 DOWN NECAB2

RP11_669E14.4 -2.83238007 -2.314143142 -7.929970615 2.04E-13 2.14E-12 20.00746971 DOWN RP11-669E14.4

PRAMEF10 -3.743541682 -2.860711648 -7.929441174 2.05E-13 2.15E-12 20.00625506 DOWN PRAMEF10

MTTP -2.735186463 6.224214272 -7.927887504 2.07E-13 2.17E-12 19.48027116DOWN MTTP

RP11_307C19.2 1.963146084 -3.37717523 7.927847218 2.07E-13 2.17E-12 19.99515212 UP RP11-307C19.2

PQLC2L 2.500533583 -4.552789499 7.927419145 2.07E-13 2.17E-12 20.00014017 UP PQLC2L

EPPK1 2.839283665 0.962347352 7.927215516 2.08E-13 2.17E-12 19.81266651 UP EPPK1

PLP2 1.486086934 4.401000106 7.926085019 2.09E-13 2.19E-12 19.51357851 UP PLP2

AC007182.6-2.216022393 -3.205946779 -7.923946199 2.12E-13 2.21E-12 19.98024755 DOWN AC007182.6

STX1A 1.039135307 0.799986862 7.919481913 2.17E-13 2.27E-12 19.79988993 UP STX1A

IFIT1 -1.756160458 4.583035938 -7.919152058 2.18E-13 2.28E-12 19.48512854 DOWN IFIT1

RP11_347E10.1 -2.809247064 -2.977251988 -7.918989022 2.18E-13 2.28E-12 19.94869556 DOWN RP11-347E10.1

LINC00574 -1.694685973 -0.449631728 -7.918901015 2.18E-13 2.28E-12 19.89569299 DOWN LINC00574

RP11_185E8.2 2.124216451 -2.539741824 7.917169641 2.21E-13 2.30E-12 19.92012804 UP RP11-185E8.2

CCDC177 -3.176497443 -2.582270169 -7.912777048 2.26E-13 2.36E-12 19.90826569 DOWN CCDC177

DPF3 -1.817884613 -0.151057985 -7.909862941 2.30E-13 2.40E-12 19.8315329 DOWN DPF3

FOXO6 2.691981284 -1.773045717 7.902073438 2.41E-13 2.51E-12 19.81073071 UP FOXO6

RP13_20L14.6 -1.035245816 -0.157142419 -7.899041486 2.46E-13 2.56E-12 19.76067809 DOWN RP13-20L14.6

RP11_314N2.2 -1.896681836 -5.572935472 -7.897897513 2.48E-13 2.57E-12 19.82932258 DOWN RP11-314N2.2

RP11_817I4.1 1.555341726 -2.377556639 7.895507295 2.51E-13 2.61E-12 19.79533103 UP RP11-817I4.1

F2 -2.389076654 9.558000723 -7.894966429 2.52E-13 2.61E-12 19.30193178 DOWN F2

TRIM46 1.60154359 -1.65745746 7.894427448 2.53E-13 2.62E-12 19.77213135 UP TRIM46

ZNF83 1.726228704 2.756689253 7.889886753 2.60E-13 2.69E-12 19.44077411UP ZNF83

PMS2P5 -1.324657792 -0.89349756 -7.889791093 2.60E-13 2.69E-12 19.73915498 DOWN PMS2P5

LIPG -1.869506296 4.280078015 -7.888096766 2.63E-13 2.72E-12

19.32959185 DOWN LIPG

PTP4A3 1.627682121 4.08176159 7.887793753 2.63E-13 2.72E-12 19.31186611 UP PTP4A3

RP3_399L15.3 1.244109855 -2.947883531 7.88673646 2.65E-13 2.74E-12 19.75476186 UP RP3-399L15.3

TDRD5 2.797952972 -4.453047372 7.884463173 2.68E-13 2.78E-12 19.74727974 UP TDRD5

AKAP6 -1.438434094 1.334789312 -7.883719865 2.69E-13 2.79E-12 19.58589129 DOWN AKAP6

NEURL1 2.197535658 -0.658969346 7.882624882 2.71E-13 2.80E-12 19.65936497 UP NEURL1

TMEM61 2.553229071 -3.368401188 7.882058728 2.72E-13 2.81E-12 19.72346972 UP TMEM61

ZBTB7C -2.216900015 -0.021092367 -7.875246146 2.84E-13 2.92E-12 19.6264456 DOWN ZBTB7C

FABP6 2.528041277 -4.501681516 7.873796642 2.86E-13 2.94E-12 19.68654099 UP FABP6

FCGBP 2.010356685 2.048883232 7.872408942 2.88E-13 2.97E-12 19.40381675 UP FCGBP

AF064858.6 -2.685429791 -0.330328924 -7.871283367 2.90E-13 2.99E-12 19.62073084 DOWN AF064858.6

DMRTA1 -2.02400773 2.563233198 -7.869708816 2.93E-13 3.01E-12 19.40539723 DOWN DMRTA1

ATF7IP2 -1.258024902 3.918822176 -7.862907058 3.05E-13 3.13E-12 19.19325224 DOWN ATF7IP2

MFAP2 2.523823626 0.037916514 7.86258856 3.06E-13 3.13E-12 19.50047043 UP MFAP2

DNMBP_AS1 -2.012435889 -0.815242161 -7.862466034 3.06E-13 3.14E-12 19.58173786 DOWN DNMBP-AS1

IGF2BP1 4.43677077 0.313124686 7.859268669 3.12E-13 3.20E-12 19.4487567 UP IGF2BP1

MYLK_AS1 1.209751008 -1.270246902 7.85871457 3.13E-13 3.20E-12 19.55308033 UP MYLK-AS1

SCCPDH -1.213303837 7.212301371 -7.857518948 3.15E-13 3.22E-12 19.04604029 DOWN SCCPDH
ANGPTL4 -1.819341456 6.105167417 -7.856244102 3.18E-13 3.25E-12 19.04674735 DOWN ANGPTL4
XXbac_BPG283O16.9 1.100094542 1.520260977 7.847130947 3.35E-13 3.43E-12 19.31330267 UP XXbac-BPG283O16.9
B4GALNT4 3.587584872 -1.668648373 7.84160659 3.47E-13 3.54E-12 19.44322038 UP B4GALNT4
FAM110D -1.395951138 -0.168348961 -7.840635279 3.49E-13 3.56E-12 19.4227176 DOWN FAM110D
GSTA2 -3.905950431 4.23331484 -7.840591806 3.49E-13 3.56E-12 19.14596738 DOWN GSTA2
TMEM56_RWDD3 -1.243742364 -0.148527348 -7.84007858 3.50E-13 3.57E-12 19.41724153 DOWN TMEM56-RWDD3
HPN -1.780635846 8.347933753 -7.839015202 3.52E-13 3.59E-12 18.94958154 DOWN HPN
RP11_206P5.2 2.122122151 -5.217722815 7.838647337 3.53E-13 3.60E-12 19.48434756 UP RP11-206P5.2
SNED1 -1.283042161 2.937252499 -7.83730475 3.56E-13 3.62E-12 19.15262649 DOWN SNED1
FAM159A 2.167760297 -2.113327659 7.836646764 3.57E-13 3.64E-12 19.4403506 UP FAM159A
ESYT3 2.390432891 -1.35061431 7.834733454 3.61E-13 3.67E-12 19.40702899 UP ESYT3
GLS 1.359848628 4.574634092 7.832706654 3.66E-13 3.72E-12 18.95039906 UP GLS
ST6GALNAC4 1.177610333 2.542937431 7.832500956 3.66E-13 3.72E-12 19.12442377 UP ST6GALNAC4
RSPH14 1.90849606 -2.426950187 7.829167484 3.73E-13 3.79E-12 19.4056309 UP RSPH14
RP11_15B24.5 -2.588730172 -3.471225884 -7.828140351 3.76E-13 3.81E-12 19.42227726 DOWN RP11-15B24.5
RP11_286H15.1 -1.791236033 -2.992272265 -7.825446696 3.82E-13

3.87E-12 19.40562596 DOWN RP11-286H15.1

RP11_152N13.5 1.035193721 -1.029564418 7.822504075 3.89E-13 3.94E-12 19.33379661 UP RP11-152N13.5

RASGEF1A 2.673778709 -0.915571711 7.821296521 3.91E-13 3.96E-12 19.30909441 UP RASGEF1A

ATP1A1 1.023798162 8.522368345 7.813078194 4.11E-13 4.16E-12 18.81071977 UP ATP1A1

ACAD11 -1.660017813 0.185317839 -7.811508773 4.15E-13 4.19E-12 19.23750949 DOWN ACAD11

RP11_883G14.3 -2.200410156 -3.01674922 -7.809219218 4.21E-13 4.25E-12 19.31074722 DOWN RP11-883G14.3

REEP6 -1.922112795 7.191835287 -7.809089553 4.21E-13 4.25E-12 18.76230127 DOWN REEP6

ZNF532 1.268758092 2.967857041 7.808743174 4.22E-13 4.26E-12 18.94116784UP ZNF532

LINC01252 -2.04835695 -1.673223817 -7.804595385 4.32E-13 4.36E-12 19.26804432 DOWN LINC01252

ZNF600 1.245785566 1.894542491 7.803891327 4.34E-13 4.38E-12 19.02206566 UP ZNF600

RP3_412A9.16 1.352990453 -2.272409589 7.802147933 4.39E-13 4.42E-12 19.25021514 UP RP3-412A9.16

PPAP2C3.306023857 2.150132126 7.797231744 4.52E-13 4.55E-12 18.95751274 UP PPAP2C

CKMT1A 2.596019801 -4.183846247 7.7963711974.54E-13 4.57E-12 19.23317772 UP CKMT1A

CCDC74A 1.777057544 -0.884350214 7.793170122 4.63E-13 4.65E-12 19.15107239 UP CCDC74A

FAM217B 1.218083305 1.994471899 7.79293113 4.63E-13 4.66E-12 18.94817597 UP FAM217B

AC008592.4-2.552595334 -2.873793538 -7.79258136 4.64E-13 4.66E-12 19.21227162 DOWN AC008592.4

ORM2 -2.348693933 8.554949388 -7.792543742 4.64E-13 4.66E-12 18.67924792 DOWN ORM2

KCNF1 2.988526364 -1.862765235 7.79111179 4.68E-13 4.70E-12 19.16043274 UP KCNF1

ARL2 1.054172401 4.054038199 7.788838838 4.75E-13 4.76E-12 18.72358055 UP ARL2

PTGR1 -1.95551014 7.150250533 -7.786072089 4.83E-13 4.84E-12 18.62703418 DOWN PTGR1

SLC22A7 -3.295992636 6.112496434-7.785671068 4.84E-13 4.85E-12 18.65694047 DOWN SLC22A7

DLC1 -1.024859961 4.704839482 -7.784332791 4.88E-13 4.88E-12 18.66342613 DOWN DLC1

ENG -1.0648968 6.193699676 -7.784168916 4.88E-13 4.89E-12 18.61543767 DOWN ENG

CCL15_CCL14 -1.895930163 0.282374011-7.780700155 4.98E-13 4.99E-12 19.05503471 DOWN CCL15-CCL14

RP11_834C11.4 1.958655056 -0.000253316 7.776932344 5.10E-13 5.10E-12 19.00696912 UP RP11-834C11.4

CFAP45 1.638110409-2.214784913 7.773794378 5.19E-13 5.19E-12 19.08215737 UP CFAP45

SPACA7 -2.182131625 -5.105151244 -7.773784749 5.19E-13 5.19E-12 19.10814538 DOWN SPACA7

AC099552.2-1.609087514 -5.82545587 -7.770726225 5.29E-13 5.28E-12 19.08971681 DOWN AC099552.2

LIPC -2.018766163 6.067851669 -7.76585871 5.44E-13 5.43E-12 18.51761593 DOWN LIPC

RP11_573D15.8 -1.656389672 1.643929198 -7.764535978 5.49E-13 5.47E-12 18.86501767 DOWN RP11-573D15.8

HS6ST23.50670176 -2.38926247 7.761397417 5.59E-13 5.57E-12 18.99426913 UP HS6ST2

TMC3_AS1 -1.546835616 -1.025263165 -7.761228308 5.60E-13 5.57E-12 18.99576211DOWN TMC3-AS1

ASPHD2 1.269278814 -0.516001096 7.757966255 5.71E-13 5.68E-12 18.93261783 UP ASPHD2

RP11_770E5.1 -2.129175374 -4.026647688 -7.757197171 5.73E-13

5.69E-12 19.01266732 DOWN RP11-770E5.1

RP11_432J24.3 2.354125406 -4.824642943 7.755295408 5.80E-13

5.75E-12 19.00017284 UP RP11-432J24.3

CAPN11 -1.120993542 -1.071896076 -7.752007577 5.91E-13 5.86E-

12 18.9409154 DOWN CAPN11

RP11_352D13.6 2.154594021 -4.337790313 7.747844763 6.06E-13

6.00E-12 18.95591108UP RP11-352D13.6

C2orf81 1.169540534 -0.224537935 7.746264779 6.12E-13 6.05E-12

18.85013385 UP C2orf81

CTD_2207P18.2 1.624766192 -4.898766302 7.743792267 6.21E-13

6.13E-12 18.93484433 UP CTD-2207P18.2

PPP1R14C 2.734139678 -3.586969965 7.743287952 6.22E-13 6.14E-

12 18.91800027 UP PPP1R14C

GPR146-1.716390588 0.035632208 -7.741784693 6.28E-13 6.19E-12

18.83983208 DOWN GPR146

ACAN 2.283344256 -0.886947344 7.740452894 6.33E-13 6.24E-12

18.83998878 UP ACAN

CYBA 1.638493869 5.150691922 7.739722453 6.36E-13 6.26E-12

18.38136919 UP CYBA

KLF12 -1.122589982 4.080320597 -7.739584933 6.36E-13 6.27E-12

18.45056826 DOWN KLF12

CATIP_AS1 -1.683633944 -1.775412013 -7.7373202 6.45E-13 6.34E-12

18.87913851 DOWN CATIP-AS1

RPL17P11 -2.337486605 -3.621105297 -7.736201145 6.49E-13 6.38E-

12 18.89043585 DOWN RPL17P11

ADRA2B -1.399239773 0.478366081 -7.735560879 6.52E-13 6.40E-

12 18.77583112DOWN ADRA2B

ETS2 -1.031815549 7.04809266 -7.7344383 6.56E-13 6.44E-12

18.32165197 DOWN ETS2

ANO1 -2.316038506 4.613473857 -7.732868645 6.62E-13 6.50E-12

18.40902927 DOWN ANO1

ZNF468 1.806075643 2.106862755 7.731610816 6.67E-13 6.54E-12

18.57377304 UP ZNF468

TNFSF15 1.939703999 0.063364506 7.730539062 6.71E-13 6.58E-12 18.73355003 UP TNFSF15

RP11_333B6.1 -2.244033253 -3.723095897 -7.724519588 6.96E-13 6.82E-12 18.82333866 DOWN RP11-333B6.1

SOX11 2.503399498 -3.86452971 7.723268756 7.01E-13 6.87E-12 18.80752144 UP SOX11

KIAA1683 -1.398416444 1.025126536 -7.722968923 7.02E-13 6.87E-12 18.66731045 DOWN KIAA1683

LYPD6 2.682176124 -2.009947067 7.721027962 7.10E-13 6.94E-12 18.76216327 UP LYPD6

H2AFY2 1.914966498 3.0181125567.719334479 7.18E-13 7.01E-12 18.41655078 UP H2AFY2

RP11_972P1.10 1.417868125 -3.734131368 7.719023288 7.19E-13 7.02E-12 18.78821887 UP RP11-972P1.10

EIF4EBP3 -1.223159927 2.564399171 -7.716989132 7.28E-13 7.10E-12 18.4865532 DOWN EIF4EBP3

RP11_770J1.3 -1.66723664 -1.691529757 -7.714140768 7.40E-13 7.21E-12 18.74306534 DOWN RP11-770J1.3

RP11_484N16.1 -2.735428845 -0.262880497 -7.712891664 7.45E-13 7.26E-12 18.69527759 DOWN RP11-484N16.1

RP3_434P1.6 -2.965761838 -1.792330503 -7.712857514 7.46E-13 7.26E-12 18.73839542 DOWN RP3-434P1.6

RP5_888M10.2 -1.766277609 -1.878379772 -7.711173082 7.53E-13 7.33E-12 18.73030815 DOWN RP5-888M10.2

A4GNT 2.629339313 -4.504950372 7.7078191177.68E-13 7.47E-12 18.72356651 UP A4GNT

CLRN1_AS1 -2.122325092 -4.612192115 -7.707173432 7.71E-13 7.49E-12 18.72372819 DOWN CLRN1-AS1

DKK1 3.954506132 -0.366143037 7.706395796 7.75E-13 7.53E-12 18.59999862 UP DKK1

S100A6 1.7117861155.381991641 7.705546337 7.79E-13 7.56E-12 18.17479241 UP S100A6

RP11_342K6.4 -1.710397445 -3.237015002 -7.70490233 7.82E-13 7.59E-

12 18.70920147 DOWN RP11-342K6.4

CPS1_IT1 -2.441569273 -3.597378594 -7.703891111 7.86E-13 7.63E-12 18.70372104 DOWN CPS1-IT1

RAVER2 1.618613373 1.740518889 7.70180588 7.96E-13 7.72E-12 18.43709555 UP RAVER2

IRS1 -1.185908145 5.737007297 -7.699850965 8.05E-13 7.81E-12 18.12971699 DOWN IRS1

GLRX -1.307672687 5.148948422 -7.699573329 8.07E-13 7.82E-12 18.14866249 DOWN GLRX

RP6_65G23.3 1.468426207 -1.40208357 7.696630783 8.21E-13 7.95E-12 18.61341992 UP RP6-65G23.3

SMPDL3B 2.39179014 -1.210076236 7.694589325 8.31E-13 8.04E-12 18.58687267 UP SMPDL3B

ZNF730 1.892559792 -4.526743954 7.691605451 8.46E-13 8.18E-12 18.6328562 UP ZNF730

MT_TP -1.059893442 5.157073571 -7.691380045 8.47E-13 8.19E-12 18.09689953 DOWN MT-TP

CYP2A7 -5.641713577 0.988812988 -7.689848596 8.54E-13 8.26E-12 18.5369979 DOWN CYP2A7

TMEM82 -2.899537839 2.108527883 -7.686636838 8.71E-13 8.40E-12 18.39698391 DOWN TMEM82

MAOA -1.274139481 6.788093242 -7.686370297 8.72E-13 8.41E-12 18.0406417 DOWN MAOA

AC016735.1 2.597683159 -3.391950255 7.686280563 8.73E-13 8.42E-12 18.58681011 UP AC016735.1

HORMAD2 -2.994232739 -2.224034598 -7.685836143 8.75E-13 8.43E-12 18.58754824 DOWN HORMAD2

TRMT112P4 -1.862266598 -2.512285926 -7.684534279 8.82E-13 8.49E-12 18.58614218 DOWN TRMT112P4

GLOD5 -2.281654304 -0.622649088 -7.682463816 8.93E-13 8.58E-12 18.53018358 DOWN GLOD5

RP11_176H8.1 -1.175435559 -1.440185133 -7.68048158 9.03E-13 8.68E-12 18.53934088 DOWN RP11-176H8.1

GC -1.916450172 10.72467421 -7.679318591 9.09E-13 8.74E-12 18.0654069 DOWN GC
ASCL5 1.663891379 -4.686158299 7.678398212 9.14E-13 8.78E-12 18.55751473 UP ASCL5
LINC00886 -1.825977461 1.262722253 -7.670870057 9.56E-13 9.16E-12 18.35348019 DOWN LINC00886
CRYAA -1.831236545 -5.268048526 -7.669824822 9.62E-13 9.22E-12 18.50817128 DOWN CRYAA
AMACR -1.87102665 4.372573818 -7.667915973 9.73E-13 9.31E-12 18.02919475 DOWN AMACR
COL22A1 3.107211898 -1.563380733 7.667901391 9.73E-13 9.31E-12 18.43739241 UP COL22A1
ICA1L 1.181119606 -0.547559827 7.666848942 9.79E-13 9.36E-12 18.40670758 UP ICA1L
ISPD -1.058563844 1.033156242 -7.66635029 9.82E-13 9.39E-12 18.33275614 DOWN ISPD
FAR2P1 3.400951465 -4.191343482 7.665566818 9.86E-13 9.43E-12 18.47123955 UP FAR2P1
RP11_205M3.3 -2.664257895 -3.709475168 -7.664199502 9.94E-13 9.50E-12 18.47520283 DOWN RP11-205M3.3
RP11_307C18.1 -1.593582183 -3.17098321 -7.65839342 1.03E-12 9.81E-12 18.44127957 DOWN RP11-307C18.1
PHEX 2.17775221 -2.277145328 7.658011187 1.03E-12 9.83E-12 18.40875395 UP PHEX
RP11_394B2.6 -1.631917158 -3.251499462 -7.65725113 1.04E-12 9.87E-12 18.43502588 DOWN RP11-394B2.6
CHD3 1.290919076 4.448049671 7.650979918 1.08E-12 1.02E-11 17.89323737 UP CHD3
ZNF648 -3.180776571 -1.580064819 -7.650879626 1.08E-12 1.02E-11 18.37750034 DOWN ZNF648
RP11_753A21.1 -2.022364105 -5.205777857 -7.648082652 1.09E-12 1.04E-11 18.38313849 DOWN RP11-753A21.1
C2orf50 1.58383177 -4.670737939 7.644434446 1.12E-12 1.06E-11

18.36234973 UP C2orf50

C12orf79 1.869183578 -2.192174441 7.642516921 1.13E-12 1.07E-11 18.32076206 UP C12orf79

MYBL1 1.000336159 1.359814166 7.63777469 1.16E-12 1.10E-11 18.10861788 UP MYBL1

KCNH2 2.826041546 -1.789485884 7.636812914 1.17E-12 1.11E-11 18.26702135 UP KCNH2

TMEM55A 1.460523788 1.973606227 7.636293269 1.17E-12 1.11E-11 18.03481287 UP TMEM55A

UGT2B17 -4.145015038 -0.243112888 -7.635071409 1.18E-12 1.12E-11 18.25591494 DOWN UGT2B17

SUSD1 1.062391742 1.90517101 7.633469603 1.19E-12 1.13E-11 18.02987199 UP SUSD1

ZCCHC16 -3.29879936 -3.003854059 -7.633467203 1.19E-12 1.13E-11 18.29264834 DOWN ZCCHC16

GMNC -2.988493241 -0.75443604 -7.630408954 1.21E-12 1.15E-11 18.23985423 DOWN GMNC

GPR182 -2.411353243 -1.836489691 -7.627305022 1.24E-12 1.17E-11 18.246873 DOWN GPR182

GJA3 1.968163892 -4.131499752 7.627168566 1.24E-12 1.17E-11 18.26013918 UP GJA3

SNAP25 3.072875117 0.084260743 7.62670664 1.24E-12 1.17E-11 18.11929222 UP SNAP25

RHOF 1.449102757 -0.547088724 7.624228085 1.26E-12 1.19E-11 18.1578748 UP RHOF

MIR181A2HG 1.706986188 -3.459524301 7.623587724 1.26E-12 1.19E-11 18.23438299 UP MIR181A2HG

C4B -1.660575057 7.272355518 -7.620456104 1.29E-12 1.21E-11 17.65899941 DOWN C4B

SLC22A15 2.55560856 0.032179181 7.619101107 1.30E-12 1.22E-11 18.08374552 UP SLC22A15

APOL6 -1.109156291 6.271996814 -7.613352281 1.34E-12 1.26E-11 17.61728941 DOWN APOL6

MGC32805 -2.948037645 0.11001085 -7.612008049 1.35E-12 1.27E-11
18.0986743 DOWN MGC32805

CASQ2 -1.783245301 -0.548049612 -7.610647002 1.36E-12 1.28E-11
18.1089157 DOWN CASQ2

AGMAT -1.585450294 5.476545676 -7.607852322 1.39E-12 1.30E-
11 17.60596746 DOWN AGMAT

SLC25A47 -4.549829096 3.830721716 -7.607540535 1.39E-12 1.31E-
11 17.84669665 DOWN SLC25A47

SLC6A8 2.124429714 3.470125984 7.606451909 1.40E-12 1.31E-
11 17.7228324 UP SLC6A8

KLHDC1 -1.272040901 -0.007008534 -7.605418608 1.41E-12 1.32E-
11 18.04852141 DOWN KLHDC1

ATP8A2 2.201631787 -2.527188154 7.604661316 1.41E-12 1.33E-
11 18.10667111UP ATP8A2

PGM5 -2.209557832 1.464564788 -7.604373384 1.41E-12 1.33E-11
17.96078863 DOWN PGM5

E2F7 1.755945237 -0.404673822 7.603464552 1.42E-12 1.33E-11
18.02835491 UP E2F7

RP11_465N4.5 1.145549263 -0.418352966 7.601472731 1.44E-12
1.35E-11 18.02265794 UP RP11-465N4.5

ACSF2 -1.090444312 5.683955706 -7.594906439 1.50E-12 1.40E-11
17.51914176 DOWN ACSF2

RAP2C_AS1 -1.12849431-0.829842011 -7.592193336 1.52E-12 1.42E-
11 18.00916524 DOWN RAP2C-AS1

SCUBE1 -3.331266694 1.853878573 -7.591190018 1.53E-12 1.43E-
11 17.87485854 DOWN SCUBE1

CCDC112 1.261883198 0.658456847 7.59061214 1.53E-12 1.44E-11
17.89032058 UP CCDC112

RP11_488L18.10 1.161028964 1.6551148447.58656275 1.57E-12 1.47E-
11 17.78213816 UP RP11-488L18.10

RP11_248E9.6 -2.279514298 -4.801645115 -7.586390876 1.57E-12
1.47E-11 18.02981437 DOWN RP11-248E9.6

RP11_57A19.2 1.724719183 -4.537273142 7.581750896 1.62E-12

1.51E-11 18.00272162 UP RP11-57A19.2

CD1A 2.099666837 -3.456061299 7.578313332 1.65E-12 1.54E-11 17.97188947 UP CD1A

VSTM4 -1.687214311 3.430898639 -7.576644802 1.67E-12 1.55E-11 17.58832271 DOWN VSTM4

RAB17 -1.609485138 5.775575796 -7.574220458 1.69E-12 1.57E-11 17.40267127 DOWN RAB17

FAM83A_AS1 -4.629776982 -1.155552782 -7.571402419 1.72E-12 1.60E-11 17.91037969 DOWN FAM83A-AS1

ANKRD18B2.299276896 -4.232426722 7.571244345 1.72E-12 1.60E-11 17.93904 UP ANKRD18B

LIF 2.414473125 1.103734328 7.570630485 1.73E-12 1.60E-11 17.72535466 UP LIF

NQO2 -1.11722302 5.991024377 -7.570316373 1.73E-12 1.61E-11 17.37112444DOWN NQO2

RP11_65J21.3 -1.580851236 -0.800984723 -7.569144979 1.74E-12 1.62E-11 17.87918914 DOWN RP11-65J21.3

ORM1 -2.39356122 10.18478179 -7.566384471 1.77E-12 1.64E-11 17.39412337 DOWN ORM1

STK26 1.633066056 1.66602843 7.56561978 1.78E-12 1.65E-11 17.65456766 UP STK26

NR0B1 2.902917227 -4.692632199 7.564569849 1.79E-12 1.66E-11 17.90089537 UP NR0B1

VN1R81P 1.785658001 -4.300752075 7.56241717 1.81E-12 1.68E-11 17.89126289 UP VN1R81P

PALM2 -1.663519869 -0.749876438 -7.561781701 1.82E-12 1.68E-11 17.83578807 DOWN PALM2

EIF3EP1 1.312860117-2.535484998 7.561600167 1.82E-12 1.69E-11 17.86851226 UP EIF3EP1

RHCE -1.97137964 -0.899272534 -7.56043114 1.83E-12 1.70E-11 17.83589102 DOWN RHCE

HAGHL1.700266468 0.079841771 7.560414608 1.83E-12 1.70E-11 17.75094725 UP HAGHL

SDPR -1.643520503 3.597749032 -7.557825898 1.86E-12 1.72E-11 17.46211097DOWN SDPR

5-Sep 1.551753901 2.243716176 7.555405291 1.89E-12 1.74E-11 17.53900154 UP 5-Sep

RP11_680F8.3 -1.113508019 -1.212458997 -7.555045529 1.89E-12 1.75E-11 17.81035907 DOWN RP11-680F8.3

MFSD6 1.22876691 3.033639814 7.55064929 1.94E-12 1.79E-11 17.43139811UP MFSD6

CBX2 1.48275371 1.580208696 7.550551252 1.94E-12 1.79E-11 17.57741159UP CBX2

RP11_3M1.1 -1.942522452 -3.71055012 -7.545447694 2.00E-12 1.84E-11 17.79600088 DOWN RP11-3M1.1

ARG1 -3.065845864 6.668202302 -7.544791341 2.01E-12 1.85E-11 17.23377146 DOWN ARG1

SLC39A14 -1.141302608 7.819641421 -7.544309894 2.01E-12 1.85E-11 17.22241052 DOWN SLC39A14

SLC6A6 1.847308393 2.555265219 7.544065844 2.02E-12 1.85E-11 17.44231878 UP SLC6A6

LPIN2 -1.11032913 6.829682521 -7.543319841 2.03E-12 1.86E-11 17.20951547 DOWN LPIN2

PLAUR 1.442715791 2.087437966 7.542930286 2.03E-12 1.86E-11 17.48396584 UP PLAUR

HS3ST3B1 -1.730932725 4.235319 -7.540295322 2.06E-12 1.89E-11 17.29545461 DOWN HS3ST3B1

RP11_555M1.3 -1.368012697 -2.045972765 -7.538770897 2.08E-12 1.91E-11 17.74279143 DOWN RP11-555M1.3

ZNF883 2.208951196-1.672306096 7.534926541 2.13E-12 1.95E-11 17.68597505 UP ZNF883

FXYD2 4.11385399 0.575263774 7.534649229 2.13E-12 1.95E-11 17.54694198 UP FXYD2

RP11_435O5.2 1.272213621 -0.840459307 7.531446494 2.17E-12 1.99E-11 17.64120928 UP RP11-435O5.2

FAM155B 2.725051141-1.183656163 7.529218761 2.20E-12 2.01E-11

17.63015322 UP FAM155B

PKP3 3.059338909 -2.031707339 7.527107532 2.23E-12 2.04E-11 17.64142545 UP PKP3

RP4_575N6.4 -1.434343589 -2.45112568 -7.525080817 2.25E-12 2.06E-11 17.6720398 DOWN RP4-575N6.4

ALDH1B1 -1.247806304 6.597271584 -7.524312308 2.26E-12 2.07E-11 17.10041794 DOWN ALDH1B1

C9orf173 -1.861113288 -0.695932041 -7.523059457 2.28E-12 2.08E-11 17.61338914 DOWN C9orf173

CTB_50E14.5 -1.46992375 -1.193956573 -7.520678926 2.31E-12 2.11E-11 17.61576957 DOWN CTB-50E14.5

LRRC37A7P -3.082652666 -0.290501346 -7.519321674 2.33E-12 2.13E-11 17.58587852 DOWN LRRC37A7P

RP11_612B6.2 -1.335734632 0.133659824 -7.51777388 2.35E-12 2.14E-11 17.53781258 DOWN RP11-612B6.2

BCO2 -2.350433495 1.671799747 -7.517538324 2.36E-12 2.15E-11 17.44717137 DOWN BCO2

SMARCA2 -1.103626649 5.460114287 -7.515077947 2.39E-12 2.18E-11 17.06404628 DOWN SMARCA2

ACOT11 1.859178375 -1.242611431 7.514398138 2.40E-12 2.18E-11 17.55649653 UP ACOT11

FUT4 1.737460406 1.757674529 7.512845406 2.42E-12 2.20E-11 17.34077749 UP FUT4

RP11_108O10.2 -1.896374154 -0.571799015 -7.512548901 2.42E-12 2.21E-11 17.54820457 DOWN RP11-108O10.2

RP11_317J10.2 -1.586069958 -0.430785612 -7.510940575 2.45E-12 2.22E-11 17.53014733 DOWN RP11-317J10.2

RP11_368I7.4 -1.063940832 -0.36959062 -7.510876313 2.45E-12 2.22E-11 17.52154888 DOWN RP11-368I7.4

UBE2QL1 -2.596447666 0.742315945 -7.510753741 2.45E-12 2.23E-11 17.4773299 DOWN UBE2QL1

BFSP1 1.147355226 -0.277782509 7.507990525 2.49E-12 2.26E-11 17.47835245 UP BFSP1

TNFAIP6 2.361160525-2.402753052 7.506729341 2.51E-12 2.28E-11 17.5424379 UP TNFAIP6

RP1_276N6.2 -3.036964795 -3.248595083 -7.506227536 2.52E-12 2.28E-11 17.56806571 DOWN RP1-276N6.2

CYP7A1 -4.509890013 3.202458926 -7.502752115 2.57E-12 2.33E-11 17.29506734 DOWN CYP7A1

PAQR5 2.820554074 1.459889792 7.501044716 2.59E-12 2.35E-11 17.29244668 UP PAQR5

FZD2 1.650351389 -0.502637977 7.499430428 2.62E-12 2.37E-11 17.43702811UP FZD2

GRAMD1A 1.194262749 4.965260088 7.4985071192.63E-12 2.38E-11 16.98260989 UP GRAMD1A

MICU3 -1.589522307 1.350307736 -7.491434465 2.74E-12 2.48E-11 17.30891594 DOWN MICU3

MROH2A -3.805828019 -0.334207892 -7.491207829 2.75E-12 2.48E-11 17.43217167 DOWN MROH2A

CLMN -1.002339563 5.271282513 -7.49100023 2.75E-12 2.48E-11 16.93026763 DOWN CLMN

PKIB 2.12882421 1.561573765 7.490152876 2.76E-12 2.49E-11 17.22453282 UP PKIB

FOXD2_AS1 1.530999952 0.910593075 7.48955648 2.77E-12 2.50E-11 17.28587738 UP FOXD2-AS1

CASC15 2.064710221 -0.908388223 7.489008459 2.78E-12 2.51E-11 17.39483864 UP CASC15

TUBAP2 1.137295209 -2.930689211 7.488998759 2.78E-12 2.51E-11 17.46270466 UP TUBAP2

OSBPL7 1.149801729 1.416381903 7.487408991 2.81E-12 2.53E-11 17.23399818 UP OSBPL7

ZNF850 1.10289232 0.312740651 7.487262718 2.81E-12 2.53E-11 17.32287135 UP ZNF850

RP11_243A14.1 -2.093574835 -1.53208471 -7.485451348 2.84E-12 2.55E-11 17.42855647 DOWN RP11-243A14.1

ZNF880 1.680839259 0.469022151 7.483567294 2.87E-12 2.58E-11

17.2837645 UP ZNF880
GOT1 -1.319757772 7.577117088-7.481225299 2.91E-12 2.62E-11
16.85582479 DOWN GOT1
GPM6A -2.484777741 -1.0512136 -7.480514436 2.92E-12 2.63E-11
17.38819351 DOWN GPM6A
COQ10A -1.045897903 3.824577628 -7.475996531 3.00E-12 2.69E-11 16.94411954DOWN COQ10A
TEX15 2.609373895 -4.674120572 7.475948668 3.00E-12 2.69E-11
17.39760589 UP TEX15
ATAD2 1.103399591 4.895025314 7.474727918 3.02E-12 2.71E-11
16.84794266 UP ATAD2
PLEKHH2 1.953968519 -0.022533074 7.474012363 3.04E-12 2.72E-11 17.26036295 UP PLEKHH2
CWH43 -2.234348633 -4.8602281 -7.473904645 3.04E-12 2.72E-11
17.3888809 DOWN CWH43
FAXC 2.401814114-3.32117033 7.472795023 3.06E-12 2.74E-11
17.36575765 UP FAXC
SV2A 1.552222653 -0.008806359 7.472707695 3.06E-12 2.74E-11
17.25596328 UP SV2A
GPR84 2.050105618 -3.000871569 7.471415627 3.08E-12 2.76E-11
17.3554633 UP GPR84
RP3_414A15.12 -1.255531945 -0.295267596 -7.471180954 3.09E-12
2.76E-11 17.29310694 DOWN RP3-414A15.12
MPP3 1.604951799 0.414480881 7.471004763 3.09E-12 2.76E-11
17.21742909 UP MPP3
CTD_2286N8.2 1.164173474 -2.553276989 7.470332697 3.10E-12
2.77E-11 17.34971885 UP CTD-2286N8.2
BAI2 1.942445081 -1.6123171 7.469392351 3.12E-12 2.79E-11
17.31293308 UP BAI2
HOMER2 -1.628324614 5.03021363 -7.465809846 3.19E-12 2.84E-11
16.80780877 DOWN HOMER2
IL12A_AS1 1.492371887 -5.295979829 7.464341776 3.21E-12 2.86E-11 17.33460473 UP IL12A-AS1

PVRL1 1.018622316 3.643998318 7.464245564 3.22E-12 2.86E-11 16.8732138 UP PVRL1

TTC39B -1.157219417 2.642605536 -7.45952749 3.30E-12 2.94E-11 16.98922363 DOWN TTC39B

AC005336.4 -2.625476932 0.741150298 -7.458507752 3.32E-12 2.96E-11 17.17944358 DOWN AC005336.4

CDK5R1 1.095513617 0.56089876 7.457150142 3.35E-12 2.98E-11 17.13316564 UP CDK5R1

EDNRB -1.200682778 4.034640569 -7.456651712 3.36E-12 2.99E-11 16.81847831 DOWN EDNRB

PEBP1P2 -1.52267113 -2.832787809 -7.456610257 3.36E-12 2.99E-11 17.28698646 DOWN PEBP1P2

ZNF761 1.105377264 2.594611499 7.454313418 3.41E-12 3.02E-11 16.92374474 UP ZNF761

FBLIM1 1.365866788 4.16374524 7.451802592 3.46E-12 3.07E-11 16.7621416 UP FBLIM1

ENPP7 -2.872025386 2.765975213 -7.44887384 3.52E-12 3.12E-11 16.96487717 DOWN ENPP7

ZNF506 1.14253492 1.922990295 7.448618604 3.52E-12 3.12E-11 16.96236307 UP ZNF506

TRPM8 -2.688132957 2.869312839 -7.447972334 3.53E-12 3.13E-11 16.94711008 DOWN TRPM8

CRYM -1.855250808 2.902073481 -7.44324161 3.63E-12 3.22E-11 16.88650669 DOWN CRYM

RP11_530N7.3 -1.875097995 -3.217900414 -7.442868177 3.64E-12 3.22E-11 17.2111833 DOWN RP11-530N7.3

GCKR -1.893368575 4.90103617 -7.442077391 3.66E-12 3.24E-11 16.68526236 DOWN GCKR

SLC25A34 -1.496755461 0.512248158 -7.441045829 3.68E-12 3.25E-11 17.07932395 DOWN SLC25A34

LILRB5 -1.405131372 2.046853702 -7.440710316 3.69E-12 3.26E-11 16.95121801 DOWN LILRB5

SPARCL1 -1.953704264 5.833743584 -7.437346175 3.76E-12 3.32E-

11 16.61749756 DOWN SPARCL1

POF1B 3.327676205 -1.178821483 7.435939983 3.79E-12 3.34E-11 17.09220572 UP POF1B

ZIC2 2.998737017 0.397974462 7.434625379 3.82E-12 3.37E-11 16.99473322 UP ZIC2

NKPD1 1.78840042 -3.873877572 7.433845067 3.84E-12 3.38E-11 17.15676671 UP NKPD1

APOB -1.957384124 11.5075388 -7.430680417 3.91E-12 3.44E-11 16.64968341 DOWN APOB

MIXL1 1.994614476 -4.434944836 7.429386143 3.94E-12 3.47E-11 17.13479754 UP MIXL1

SLC4A5 1.246148879 -2.276792102 7.428007828 3.97E-12 3.49E-11 17.10273038 UP SLC4A5

APOLD1 -1.232030322 4.318543021 -7.42209064 4.11E-12 3.61E-11 16.59524934 DOWN APOLD1

RP1_18D14.7 -1.62309523 -3.552548427 -7.420825387 4.14E-12 3.64E-11 17.08817415 DOWN RP1-18D14.7

RP5_1182A14.5 -2.672487476 -2.724482889 -7.420075286 4.16E-12 3.65E-11 17.07584504 DOWN RP5-1182A14.5

HS1BP3_IT1 -2.125525912 0.705536854 -7.419297485 4.18E-12 3.66E-11 16.95082088 DOWN HS1BP3-IT1

TCF24 1.866239888 -5.360759778 7.419275074 4.18E-12 3.66E-11 17.07909267 UP TCF24

APOC4_APOC2 -2.022736161 3.424788046 -7.418162771 4.20E-12 3.68E-11 16.68781981 DOWN APOC4-APOC2

C1orf115 -1.004173314 6.832189384 -7.417008428 4.23E-12 3.71E-11 16.48264834 DOWN C1orf115

ITIH4 -1.766408037 4.649447802 -7.415111363 4.28E-12 3.75E-11 16.54215375 DOWN ITIH4

C16orf86 -1.107370137 1.128184295 -7.414784132 4.29E-12 3.75E-11 16.88041174DOWN C16orf86

FAM24B 1.735583662 -1.873527452 7.410925284 4.38E-12 3.83E-11 16.99067268 UP FAM24B

CTB_33O18.3 -1.459732026 -2.14858493 -7.410602826 4.39E-12 3.84E-11 17.01680627 DOWN CTB-33O18.3
GCHFR -1.154553567 3.49680511 -7.408846764 4.44E-12 3.88E-11 16.59725463 DOWN GCHFR
RP11_46A10.5 1.085484076 -1.1526992 7.407753413 4.47E-12 3.90E-11 16.95145849 UP RP11-46A10.5
PPP1R3B -1.377730651 5.666936124 -7.403364151 4.58E-12 3.99E-11 16.41956007 DOWN PPP1R3B
TGFA 2.079695354 1.283749875 7.398771793 4.71E-12 4.09E-11 16.72836409 UP TGFA
CDHR5 -2.702103661 6.624557678 -7.398649604 4.71E-12 4.09E-11 16.38951538 DOWN CDHR5
ROCK1P1 1.464120219 -2.189275791 7.398647833 4.71E-12 4.09E-11 16.93282451 UP ROCK1P1
DNAH17 1.069442981 -0.089919944 7.397557282 4.74E-12 4.12E-11 16.83814986 UP DNAH17
AP3B2 2.15652502 -3.940892004 7.395910886 4.78E-12 4.15E-11 16.94050092 UP AP3B2
RASGEF1B -1.336655148 3.616870802 -7.39578697 4.79E-12 4.16E-11 16.51830717 DOWN RASGEF1B
GPR17 -2.094802934 -2.204530926 -7.395697538 4.79E-12 4.16E-11 16.93348617 DOWN GPR17
RP1_28O10.1 -1.949047339 -0.492156245 -7.395336898 4.80E-12 4.16E-11 16.87838547 DOWN RP1-28O10.1
LPAL2 -1.669564742 1.12588639 -7.39474024 4.82E-12 4.18E-11 16.77496279 DOWN LPAL2
RP11_162A12.2 -1.003829408 -0.045705064 -7.39440503 4.83E-12 4.18E-11 16.84086037 DOWN RP11-162A12.2
FDXR -1.023793741 4.524481352 -7.393904264 4.84E-12 4.19E-11 16.41279451 DOWN FDXR
SLC37A4 -1.031445231 6.870561118 -7.391046193 4.92E-12 4.26E-11 16.33420157 DOWN SLC37A4
RP11_80A15.1 -1.515918137 -2.070200617 -7.390066151 4.95E-12

4.28E-11 16.89915388 DOWN RP11-80A15.1

TECTB -2.272181472 -2.397171722 -7.389865709 4.96E-12 4.28E-11 16.90277671 DOWN TECTB

CTD_2095E4.3 1.157247694 -2.684081657 7.388395136 5.00E-12 4.32E-11 16.88768765 UP CTD-2095E4.3

RP11_830F9.5 -2.79278874 -2.628619934 -7.387861386 5.01E-12 4.33E-11 16.89246166 DOWN RP11-830F9.5

PSD3 -1.312811512 4.14803323 -7.387259212 5.03E-12 4.34E-11 16.41478801 DOWN PSD3

RP1_102K2.8 2.249657326 -3.717513948 7.385946343 5.07E-12 4.37E-11 16.88058269 UP RP1-102K2.8

ZNF280B 2.03775137 -3.008138002 7.383854457 5.13E-12 4.42E-11 16.85927139 UP ZNF280B

OIT3 -1.810773482 2.730762588 -7.383432618 5.14E-12 4.43E-11 16.56123837 DOWN OIT3

SHH -1.602097221 3.641742985 -7.382108225 5.18E-12 4.46E-11 16.4424099 DOWN SHH

RP11_12A2.3 -2.671418267 -3.392673717 -7.381481721 5.20E-12 4.48E-11 16.86352576 DOWN RP11-12A2.3

MGMT -1.067889863 6.059108574 -7.38002488 5.25E-12 4.51E-11 16.27549276 DOWN MGMT

GPD1L 1.155297114 2.485805382 7.379732157 5.26E-12 4.52E-11 16.50898574 UP GPD1L

GCH1 -1.446648056 4.573652513 -7.377019883 5.34E-12 4.59E-11 16.32329564 DOWN GCH1

SEMA4D 1.192510093 2.427942528 7.372688554 5.47E-12 4.70E-11 16.4748139 UP SEMA4D

LAMC1 1.037596435 6.883010065 7.372260733 5.49E-12 4.71E-11 16.23213379 UP LAMC1

LINC00628 1.572463565 -3.979506957 7.370486196 5.54E-12 4.75E-11 16.80115847 UP LINC00628

RP11_574F21.3 -1.703524828 -2.925372325 -7.368783921 5.60E-12 4.80E-11 16.79099945 DOWN RP11-574F21.3

CDH5	-1.009005296	4.752415414	-7.364777097	5.73E-12	4.90E-11
16.2318308	DOWN	CDH5
DNM1	1.858632917	1.775968304	7.364229553	5.75E-12	4.91E-11
16.48782973	UP	DNM1
SLC45A4	1.577397141	2.385151579	7.363504831	5.77E-12	4.93E-11	16.42473196	UP	SLC45A4
RP11_903H12.3	-1.503633029	-1.707226834	-7.362321397	5.81E-12
4.96E-11	16.7332672	DOWN	RP11-903H12.3
ETV4	2.529655912	2.732807999	7.360419386	5.88E-12	5.01E-11
16.37899529	UP	ETV4
GUSBP11	1.200266886	-0.348022236	7.357734227	5.97E-12	5.09E-11	16.62629788	UP	GUSBP11
RP5_857K21.7	-1.10301211	1.249645444	-7.356851282	6.00E-12	5.11E-11	16.54092713	DOWN	RP5-857K21.7
OSTN_AS1	-2.693122088	-2.912443745	-7.355435074	6.05E-12	5.15E-11	16.71274688	DOWN	OSTN-AS1
TMEM176A-1.554441789	8.968430326	-7.354500578	6.08E-12	5.18E-11	16.15175303	DOWN	TMEM176A
OR10J6P	-2.598558046	-1.095100713	-7.353751105	6.11E-12	5.20E-11	16.67145957	DOWN	OR10J6P
KRT19	3.879371582	1.1140718027.352782813	6.14E-12	5.22E-11
16.47063629	UP	KRT19
MT_ND2	-1.022419989	11.90807437-7.352404809	6.16E-12	5.23E-11
16.21878417	DOWN	MT-ND2
CDH24	1.147665957	1.217570064	7.350637804	6.22E-12	5.29E-11
16.47267866	UP	CDH24
BMP8B	2.1172583150.14562501	7.350546608	6.22E-12	5.29E-11
16.54454607	UP	BMP8B
FBXL2	1.30389887	-0.16283546	7.350268306	6.23E-12	5.29E-11
16.57245639	UP	FBXL2
GOLGA6A	-2.064496834	-4.753197208	-7.347189251	6.34E-12	5.39E-11	16.67324914	DOWN	GOLGA6A
TUBA1B	1.09736877	5.976256399	7.34453603	6.44E-12	5.47E-11

16.0754473 UP TUBA1B

IL1RN -1.709343074 4.753720402 -7.343727012 6.47E-12 5.49E-11 16.12637838 DOWN IL1RN

AC006538.1 1.499196156 -2.445865927 7.342891846 6.50E-12 5.52E-11 16.62295852 UP AC006538.1

SERPIND1 -2.44915453 8.942951871 -7.34225114 6.53E-12 5.53E-11 16.08009576 DOWN SERPIND1

CYP2D6 -2.571188391 6.340064235 -7.339151545 6.65E-12 5.63E-11 16.05263889 DOWN CYP2D6

FSCN2 1.541608303 -3.715488622 7.337943709 6.69E-12 5.67E-11 16.61565214 UP FSCN2

DIO1 -2.528834154 5.089432881 -7.337565723 6.71E-12 5.68E-11 16.09425578 DOWN DIO1

RAB3IL1 1.310502758 2.658271151 7.337530361 6.71E-12 5.68E-11 16.24962167 UP RAB3IL1

PDE4A 1.273606809 2.279852241 7.33268081 6.90E-12 5.83E-11 16.26266087 UP PDE4A

ZNF702P 1.812376251 -1.148115143 7.331051713 6.97E-12 5.88E-11 16.51167454UP ZNF702P

TEX19 1.981304481 -3.37726297 7.325684873 7.18E-12 6.06E-11 16.53807958 UP TEX19

GOLGA6B -2.083111876 -4.775160585 -7.322916169 7.30E-12 6.15E-11 16.53682965 DOWN GOLGA6B

MIR600HG 1.213315471 0.659122172 7.322006143 7.34E-12 6.18E-11 16.35603156 UP MIR600HG

ADORA2B 1.967143272 -0.986091695 7.321388244 7.36E-12 6.20E-11 16.44834466 UP ADORA2B

NRSN2_AS1 1.03669248 -0.789389718 7.320820662 7.39E-12 6.22E-11 16.44366909 UP NRSN2-AS1

DMBT1 3.165821782 -3.027770327 7.320476514 7.40E-12 6.23E-11 16.48969682 UP DMBT1

GAS6 -1.217905946 5.499935823 -7.318446369 7.49E-12 6.30E-11 15.93809325 DOWN GAS6

SPATA12 1.682384749 -4.745996182 7.316709451 7.57E-12 6.36E-11 16.50157977 UP SPATA12

PACSIN1 2.25084251 -0.896659839 7.316360967 7.58E-12 6.37E-11 16.41205492 UP PACSIN1

MTATP6P1 -1.085434745 8.841646072 -7.309720572 7.88E-12 6.61E-11 15.89567161 DOWN MTATP6P1

TAL1 -1.151798954 0.421942458 -7.308790423 7.92E-12 6.64E-11 16.33054003 DOWN TAL1

RP11_728F11.3 2.525812858 -4.164344264 7.304592664 8.11E-12 6.80E-11 16.42625375 UP RP11-728F11.3

RP11_806H10.4 2.04022918 -3.035881344 7.304271835 8.13E-12 6.81E-11 16.41151387UP RP11-806H10.4

CTD_2256P15.2 -1.08641679 0.820112655-7.303955754 8.14E-12 6.82E-11 16.27497501 DOWN CTD-2256P15.2

FKBP10 1.772880886 4.082522526 7.301263306 8.27E-12 6.92E-11 15.91621925 UP FKBP10

CAPN10_AS1 1.048914611-0.180062574 7.299410537 8.36E-12 7.00E-11 16.28867119UP CAPN10-AS1

CYP1A2 -6.43988501 1.455905798 -7.298866633 8.39E-12 7.02E-11 16.29229763 DOWN CYP1A2

VAC14_AS1 2.232235751 -1.228721077 7.2974464118.46E-12 7.07E-11 16.32124386 UP VAC14-AS1

ASRGL1 1.629217625 1.769628867 7.296965528 8.48E-12 7.09E-11 16.10901451 UP ASRGL1

RP11_41O4.1 -1.292251579 0.944780609 -7.296859466 8.48E-12 7.09E-11 16.22833266 DOWN RP11-41O4.1

N4BP3 1.347074997 0.401924805 7.294173192 8.62E-12 7.19E-11 16.21661345 UP N4BP3

CYP2E1 -4.100620063 8.683657673 -7.293309345 8.66E-12 7.23E-11 15.8014362 DOWN CYP2E1

OR7E102P -1.826697744 -3.997561499 -7.292449637 8.70E-12 7.26E-11 16.36634568 DOWN OR7E102P

ABCC11 -1.648913874 1.125162051 -7.292280587 8.71E-12 7.26E-

11	16.19429624	DOWN	ABCC11
CHST11	1.379455294	2.771738669	7.291377505	8.76E-12	7.30E-11	15.97585329	UP	CHST11
BANF1P2	-1.880372727	-0.618141826	-7.285200242	9.07E-12	7.56E-11	16.26136947	DOWN	BANF1P2
ZNF813	1.746354157	1.036458027	7.284968988	9.09E-12	7.57E-11	16.10754891	UP	ZNF813
LINC00654	1.648349217	-0.982758798	7.284746638	9.10E-12	7.57E-11	16.24432621	UP	LINC00654
SLMO1	1.521525969	-1.592631826	7.283424847	9.17E-12	7.63E-11	16.26416282	UP	SLMO1
LINC00941	2.189011879	-2.984982453	7.282218465	9.23E-12	7.68E-11	16.2856228	UP	LINC00941
ACSS3	-2.241236693	5.331125322	-7.279695824	9.37E-12	7.79E-11	15.74356947	DOWN	ACSS3
QSOX1	1.88349494	5.636633438	7.278556376	9.43E-12	7.83E-11	15.71294195	UP	QSOX1
C1orf116	2.652858831	0.126493025	7.277185191	9.50E-12	7.89E-11	16.12613814	UP	C1orf116
THSD1	-1.354058479	1.593485173	-7.277131458	9.51E-12	7.89E-11	16.06530399	DOWN	THSD1
MT2A	-2.213922044	7.652961963	-7.271035157	9.84E-12	8.16E-11	15.65779687	DOWN	MT2A
IBSP	2.682869955	-3.989359699	7.26921186	9.95E-12	8.24E-11	16.22486045	UP	IBSP
CTNNA3	-2.327868813	-1.171944047	-7.26906926	9.96E-12	8.24E-11	16.19629478	DOWN	CTNNA3
NAMPT	-1.334611294	6.606459124	-7.268657709	9.98E-12	8.26E-11	15.63888035	DOWN	NAMPT
CYP2A7P1	-1.925216956	-5.496580744	-7.268292579	1.00E-11	8.27E-11	16.22979078	DOWN	CYP2A7P1
KCNH3	2.194724197	-1.778599438	7.26257732	1.03E-11	8.54E-11	16.14707477	UP	KCNH3

CCR10 1.345578786 -1.863664862 7.261644694 1.04E-11 8.58E-11 16.15249776 UP CCR10

RP11_164O23.7 -1.561588642 -5.006364271 -7.261552453 1.04E-11 8.59E-11 16.19329288 DOWN RP11-164O23.7

GPR128-2.472671267 0.183795339 -7.26097313 1.04E-11 8.61E-11 16.09142934 DOWN GPR128

AKR1C8P -2.766960266 -1.065870143 -7.260948114 1.04E-11 8.61E-11 16.14971714 DOWN AKR1C8P

RP11_669I1.1 -1.401899075 -5.798170644 -7.260794507 1.04E-11 8.61E-11 16.18763905 DOWN RP11-669I1.1

GMNN 1.087374529 4.858284424 7.26033314 1.05E-11 8.63E-11 15.62625787 UP GMNN

RP11_439C15.4 -2.19474779 -3.634839211 -7.258894289 1.06E-11 8.70E-11 16.17759409 DOWN RP11-439C15.4

RP11_776H12.1 2.52391707 -4.350089063 7.258892481 1.06E-11 8.70E-11 16.17270884 UP RP11-776H12.1

IL6R -1.214794605 6.258711334-7.256215128 1.07E-11 8.83E-11 15.57020219 DOWN IL6R

TGFBR3 -1.300154203 3.923612753 -7.254561958 1.08E-11 8.90E-11 15.68083136 DOWN TGFBR3

RP13_890H12.2 1.516121504 -2.226136884 7.251348293 1.10E-11 9.06E-11 16.1035976 UP RP13-890H12.2

MAMSTR 1.370382938 -0.348778085 7.247220525 1.13E-11 9.27E-11 16.00151926 UP MAMSTR

RP11_290H9.4 -1.261691625 -2.471701295 -7.247140504 1.13E-11 9.27E-11 16.10374241 DOWN RP11-290H9.4

ZNF670 1.629090037 -4.435393466 7.243783566 1.15E-11 9.44E-11 16.09293976 UP ZNF670

LINC00632 2.200884789 -4.867808598 7.243057269 1.16E-11 9.48E-11 16.08855254 UP LINC00632

CNIH2 1.243033747 -1.654685144 7.24296805 1.16E-11 9.48E-11 16.04185814 UP CNIH2

NAP1L5 -1.302047414 1.199515042 -7.240263038 1.17E-11 9.62E-

11 15.89064148 DOWN NAP1L5

RP11_200A1.1 -1.981323351 -3.364372199 -7.239874162 1.18E-11 9.64E-11 16.07055765 DOWN RP11-200A1.1

EXPH5 -2.589812381 1.155078836 -7.235767234 1.21E-11 9.86E-11 15.89031004 DOWN EXPH5

PGLYRP2 -2.862046254 5.333183197 -7.232689925 1.23E-11 1.00E-10 15.49635337 DOWN PGLYRP2

SMIM22 2.643163704 -2.523790594 7.230723141 1.24E-11 1.01E-10 15.98253488 UP SMIM22

MTND6P4 -1.68833892 -1.908647135 -7.230418379 1.24E-11 1.01E-10 15.9991215 DOWN MTND6P4

AC129492.6 1.778008973 -4.61303934 7.225989153 1.28E-11 1.04E-10 15.99383816 UP AC129492.6

DIRAS3 -2.201453505 -0.900769948 -7.224670618 1.28E-11 1.05E-10 15.9373729 DOWN DIRAS3

MNX1_AS1 2.919955791 -4.484934532 7.219535497 1.32E-11 1.08E-10 15.95102531 UP MNX1-AS1

RP11_14N7.2 1.758824326 -3.043706812 7.219419177 1.32E-11 1.08E-10 15.93953863 UP RP11-14N7.2

ZNF439 1.331277788 -0.439504588 7.217514702 1.34E-11 1.09E-10 15.84108504 UP ZNF439

IGFBP1 -2.399953941 8.016074745 -7.2159119 1.35E-11 1.10E-10 15.35006944 DOWN IGFBP1

RP11_705C15.2 1.02172736 0.233262839 7.214450729 1.36E-11 1.11E-10 15.78418893 UP RP11-705C15.2

AC008592.3 -1.232577896 -1.351010711 -7.213049937 1.37E-11 1.11E-10 15.88225946 DOWN AC008592.3

PRIM1 1.01057031 2.46868644 7.212046108 1.38E-11 1.12E-10 15.56223243 UP PRIM1

EIF5A2 1.750396813 1.498568224 7.210163431 1.40E-11 1.13E-10 15.64498978 UP EIF5A2

TPM4 1.040444939 6.69743487 7.203750328 1.45E-11 1.17E-10 15.27541714 UP TPM4

ARRDC4 -1.723952359 3.635498718 -7.202925232 1.46E-11 1.18E-10 15.43606955 DOWN ARRDC4

FAM134B -2.681191062 2.15630922 -7.199030344 1.49E-11 1.21E-10 15.60600303 DOWN FAM134B

LINC01044 -1.431363417 -5.838084415 -7.188590669 1.58E-11 1.28E-10 15.78511614DOWN LINC01044

LINC00570 -2.364715606 -3.155960175 -7.188065227 1.58E-11 1.28E-10 15.77964891 DOWN LINC00570

RGSL1 -3.661307674 -2.60752463 -7.186359552 1.60E-11 1.29E-10 15.75924346 DOWN RGSL1

RP11_394I13.2 1.340886035 -3.080292032 7.183987313 1.62E-11 1.31E-10 15.74744602 UP RP11-394I13.2

ASGR2 -1.85557122 8.606367499 -7.183702131 1.62E-11 1.31E-10 15.17694812 DOWN ASGR2

YJEFN3 1.302519453 0.176337562 7.178176579 1.68E-11 1.35E-10 15.58166867 UP YJEFN3

PLBD1 1.841673672 1.939006848 7.176450303 1.69E-11 1.36E-10 15.41156539UP PLBD1

TSPAN15 1.397606585 3.39609968 7.173802256 1.72E-11 1.38E-10 15.24970811UP TSPAN15

RP5_908M14.10 1.402327136 -2.497946691 7.170906052 1.75E-11 1.41E-10 15.66274331 UP RP5-908M14.10

RNF144A_AS1 2.166820691 -2.939421977 7.170720757 1.75E-11 1.41E-10 15.66243837 UP RNF144A-AS1

CYP2C18 -2.40169334 4.29581118 -7.170040126 1.76E-11 1.41E-10 15.21087742 DOWN CYP2C18

ABCC6P1 -1.556857903 3.571655608 -7.163714706 1.82E-11 1.46E-10 15.21319384 DOWN ABCC6P1

LCN12 -1.302316047 2.489285694 -7.159814649 1.86E-11 1.49E-10 15.31051146DOWN LCN12

ZNF607 1.446918238 0.611491493 7.157717643 1.88E-11 1.51E-10 15.43250985 UP ZNF607

IGFBPL1 2.593605289 -3.315106246 7.154993155 1.91E-11 1.53E-

10 15.57715484 UP IGFBPL1

AL133493.2 2.238075547 -3.496851537 7.154739816 1.92E-11 1.53E-10 15.58319941 UP AL133493.2

RP4_782L23.1 -1.525306369 -1.735200591 -7.152520147 1.94E-11 1.55E-10 15.56013296 DOWN RP4-782L23.1

LYPD1 2.463734663 0.538525653 7.151041321 1.96E-11 1.56E-10 15.38926491 UP LYPD1

RP11_736K20.6 -1.343608869 -0.758193821 -7.150556724 1.96E-11 1.57E-10 15.51030293 DOWN RP11-736K20.6

DNMT3L -2.47925469 -2.902963732 -7.149894549 1.97E-11 1.57E-10 15.56515133 DOWN DNMT3L

HNF4A -1.468385754 7.625899059 -7.147413012 2.00E-11 1.60E-10 14.95926553 DOWN HNF4A

RP11_103B5.4 1.600169502 -3.877168495 7.147122941 2.00E-11 1.60E-10 15.55178739 UP RP11-103B5.4

RP11_7F17.3 -1.753649608 -1.503984501 -7.145751887 2.02E-11 1.61E-10 15.516203 DOWN RP11-7F17.3

VEPH1 2.943128898 -1.220826188 7.143165933 2.05E-11 1.63E-10 15.45137025 UP VEPH1

RP11_746P2.3 1.249332872 -5.615377128 7.143036385 2.05E-11 1.63E-10 15.53339864 UP RP11-746P2.3

CTA_941F9.10 1.044360911 -1.56691078 7.139150757 2.09E-11 1.67E-10 15.46132861 UP CTA-941F9.10

RGS20 1.678795956 -3.675012899 7.133167948 2.17E-11 1.72E-10 15.47150552 UP RGS20

TOR4A 1.285449637 2.323429313 7.132548807 2.17E-11 1.73E-10 15.12994991 UP TOR4A

ADAM12 2.132852733 -0.013156599 7.131219676 2.19E-11 1.74E-10 15.32330829 UP ADAM12

RP11_449J21.5 2.25756566 -2.10373617 7.130487799 2.20E-11 1.75E-10 15.4184504 UP RP11-449J21.5

FOLH1B -3.51082581 -1.180776818 -7.128714542 2.22E-11 1.76E-10 15.41767296 DOWN FOLH1B

SLC7A7 1.225034629 2.757687827 7.128685845 2.22E-11 1.76E-10 15.06183944 UP SLC7A7
KCNK12 1.746953405 -4.608312544 7.123588308 2.29E-11 1.81E-10 15.42544592 UP KCNK12
SLC16A11 -2.509764764 3.035851326 -7.120498205 2.33E-11 1.84E-10 15.06561915 DOWN SLC16A11
SLC2A6 1.493701867 2.486120695 7.1199143382.34E-11 1.85E-10 15.04090488 UP SLC2A6
RP11_261N11.8 -3.189488526 -2.74989466 -7.118681242 2.35E-11 1.86E-10 15.38755885 DOWN RP11-261N11.8
TBC1D30 1.490368802 1.127600532 7.1165187832.38E-11 1.88E-10 15.15873399 UP TBC1D30
RP11_77H9.8 -1.521190773 -3.961475262 -7.116328343 2.39E-11 1.88E-10 15.38665677 DOWN RP11-77H9.8
ZNF594 1.130201246 0.049828668 7.1143983152.41E-11 1.90E-10 15.23697435 UP ZNF594
RP11_89K21.1 2.796939264 -4.066654721 7.1118162062.45E-11 1.93E-10 15.3488916 UP RP11-89K21.1
TESC 2.280864087 3.1082112797.111771711 2.45E-11 1.93E-10 14.93979683 UP TESC
SLC17A2 -2.89519069 3.918240533 -7.111450694 2.45E-11 1.93E-10 14.93784592 DOWN SLC17A2
RP11_959F10.6 -2.186827954 -1.817624882 -7.10695961 2.52E-11 1.98E-10 15.31165031DOWN RP11-959F10.6
APOC1P1 -2.774106064 3.908211382-7.106015293 2.53E-11 1.99E-10 14.89873685 DOWN APOC1P1
RP11_179K3.2 -1.835024291 -4.29514561 -7.105029027 2.54E-11 2.00E-10 15.32424129 DOWN RP11-179K3.2
AC007326.10 -1.996086402 -5.0752767 -7.103877497 2.56E-11 2.01E-10 15.3170389 DOWN AC007326.10
MT_RNR2 -1.010776086 12.8424486 -7.103844357 2.56E-11 2.01E-10 14.84558732 DOWN MT-RNR2
FZD7 1.692141482 0.951615539 7.10326049 2.57E-11 2.02E-10

15.09791281 UP FZD7

RP11_538D16.2 -2.68376736 -2.19362836 -7.102580728 2.58E-11 2.03E-10 15.29361857 DOWN RP11-538D16.2

SOWAHB -1.667508616 2.871747327 -7.098422892 2.64E-11 2.07E-10 14.93568811 DOWN SOWAHB

SULT1C2 2.963572436 1.81978687 7.095469553 2.69E-11 2.10E-10 14.96732549 UP SULT1C2

SLC22A2 -1.94333819 -3.928615229 -7.094827705 2.70E-11 2.11E-10 15.26767192 DOWN SLC22A2

ZNF665 1.538806455 -1.445012705 7.089640038 2.78E-11 2.17E-10 15.17745896 UP ZNF665

CBLN1 -2.429182776 -0.013490797 -7.08870207 2.79E-11 2.18E-10 15.1397837 DOWN CBLN1

AP000695.4 1.540829308 -3.422314306 7.084585829 2.86E-11 2.23E-10 15.20074377 UP AP000695.4

RP11_551L14.1 1.766180236 -4.236062393 7.083931661 2.87E-11 2.24E-10 15.20480087 UP RP11-551L14.1

TGFB1 1.313633506 4.646651684 7.083194687 2.88E-11 2.25E-10 14.64401713 UP TGFB1

RP11_69L16.5 1.313915439 -2.748793126 7.082308504 2.89E-11 2.26E-10 15.17820761 UP RP11-69L16.5

LINC01106 1.655241641 -2.54189175 7.08129699 2.91E-11 2.27E-10 15.16453838 UP LINC01106

BLVRA 1.157529033 3.730168435 7.076097194 3.00E-11 2.33E-10 14.6694886 UP BLVRA

ECHDC3 -1.859101063 5.625569619 -7.07306436 3.05E-11 2.37E-10 14.56147888 DOWN ECHDC3

AC002398.13 1.02255137 -1.835822038 7.073039487 3.05E-11 2.37E-10 15.10549948 UP AC002398.13

DAGLA 1.747458824 1.193946752 7.072599932 3.06E-11 2.38E-10 14.90454098 UP DAGLA

SELE -2.233115056 0.039846729 -7.072120315 3.07E-11 2.38E-10 15.04356038 DOWN SELE

ST6GALNAC3 -1.120932674 0.537039043 -7.068957372 3.12E-11 2.43E-10 14.98130187 DOWN ST6GALNAC3

RP11_599J14.2 1.676655526 -3.087280984 7.068424068 3.13E-11 2.43E-10 15.10404939 UP RP11-599J14.2

ADAP1 1.763255279 0.74372534 7.068315219 3.13E-11 2.43E-10 14.91983784 UP ADAP1

LIMD2 1.352249826 3.32924086 7.068292846 3.13E-11 2.43E-10 14.66627001 UP LIMD2

PKLR -2.506359132 5.726177251 -7.065251474 3.19E-11 2.47E-10 14.52615213 DOWN PKLR

RP11_1334A24.5 1.359740132 -3.247827958 7.064502002 3.20E-11 2.48E-10 15.08881088 UP RP11-1334A24.5

ACER1 -2.02006367 -4.76647241 -7.062733515 3.23E-11 2.51E-10 15.09070184 DOWN ACER1

AANAT 1.411064945 -4.017305753 7.060644382 3.27E-11 2.54E-10 15.07654171 UP AANAT

CTA_941F9.9 1.993749082 -4.40960858 7.059269075 3.30E-11 2.55E-10 15.06908742 UP CTA-941F9.9

RP11_25E2.1 -1.78052856 -4.695038784 -7.058801187 3.31E-11 2.56E-10 15.06935239 DOWN RP11-25E2.1

SOX7 -1.268466742 1.296783845 -7.056231482 3.35E-11 2.60E-10 14.85380399 DOWN SOX7

RP11_1109F11.3 1.289480206 -2.05125446 7.055152405 3.38E-11 2.61E-10 15.01122105 UP RP11-1109F11.3

LINC01353 1.499385971 -3.862035457 7.05224119 3.43E-11 2.65E-10 15.02843819 UP LINC01353

RP11_248E9.7 -1.876744119 -4.938928036 -7.05190337 3.44E-11 2.66E-10 15.0310555 DOWN RP11-248E9.7

DOCK3 1.19239278 -2.042400307 7.050579552 3.46E-11 2.67E-10 14.98688663 UP DOCK3

CLU -1.245405577 11.56264048 -7.049628376 3.48E-11 2.69E-10 14.50262047 DOWN CLU

RP11_545J16.1 -2.431110304 -3.68990085 -7.049337453 3.49E-11 2.69E-

10 15.01600073 DOWN RP11-545J16.1

QRFPR -2.048133803 -4.111920784 -7.048049788 3.51E-11 2.71E-10 15.01020321 DOWN QRFPR

RP4_583P15.14 -1.802172453 -3.512258303 -7.046430569 3.55E-11 2.73E-10 15.00033373 DOWN RP4-583P15.14

RP11_49I11.4 1.273153002 -2.104025657 7.045827689 3.56E-11 2.74E-10 14.96170749 UP RP11-49I11.4

FRMD7 -2.097466293 -4.701021026 -7.043630606 3.60E-11 2.78E-10 14.98567676 DOWN FRMD7

B3GALNT1 1.295612317 0.891414538 7.039131132 3.70E-11 2.85E-10 14.75349885 UP B3GALNT1

F10 -1.628697127 7.062956077 -7.038836921 3.70E-11 2.85E-10 14.34941402 DOWN F10

RP11_533E19.7 1.16741763 -1.899386476 7.035528867 3.77E-11 2.90E-10 14.89936256 UP RP11-533E19.7

RP11_480A16.1 1.142628753 -1.494512759 7.034368787 3.80E-11 2.92E-10 14.8781935 UP RP11-480A16.1

ACR -1.565146028 -2.319718166 -7.03339777 3.82E-11 2.93E-10 14.91681393 DOWN ACR

FAM90A1 1.759394547 -3.149983774 7.03332654 3.82E-11 2.93E-10 14.9117701 UP FAM90A1

CCDC109B 1.403217686 1.333996748 7.032777499 3.83E-11 2.94E-10 14.67583113 UP CCDC109B

TNFRSF11A 1.551010546 0.676148198 7.032181173 3.84E-11 2.95E-10 14.72883309 UP TNFRSF11A

CYP1A1 -4.833257817 1.68840363 -7.031838473 3.85E-11 2.95E-10 14.75842501 DOWN CYP1A1

CASP16 -2.495160701 -0.43532636 -7.02628735 3.97E-11 3.04E-10 14.81810014 DOWN CASP16

RP11_268J15.5 1.232555132 -1.060687024 7.025612511 3.99E-11 3.05E-10 14.80907312 UP RP11-268J15.5

CXCL5 3.701633582 -1.861811118 7.024274723 4.02E-11 3.07E-10 14.80565914 UP CXCL5

BCAT1 1.396816141 2.017145615 7.023437384 4.04E-11 3.08E-10 14.55470292 UP BCAT1

SLC44A3 1.723234992 3.2175711947.021231423 4.09E-11 3.12E-10 14.41864931 UP SLC44A3

C15orf56 1.503893566 -5.235985184 7.0208911224.10E-11 3.12E-10 14.86053685 UP C15orf56

RP11_1246C19.1 1.154030247 -0.101087536 7.020746415 4.10E-11 3.12E-10 14.72811212UP RP11-1246C19.1

DLX6 2.670900524 -4.482444964 7.019089249 4.14E-11 3.15E-10 14.84468307 UP DLX6

AP000347.4 1.354879445 -2.01972625 7.018341425 4.16E-11 3.16E-10 14.8069888 UP AP000347.4

MIOX 2.493875149 -2.409032141 7.016406947 4.20E-11 3.20E-10 14.79282129 UP MIOX

RP11_37C7.3 1.304183867 -2.809023897 7.015020062 4.23E-11 3.22E-10 14.80936134 UP RP11-37C7.3

PYGL -1.099859567 6.650319095 -7.014344188 4.25E-11 3.23E-10 14.2116668 DOWN PYGL

FAM99A -4.090875761 0.703007164 -7.012996511 4.28E-11 3.25E-10 14.70299115DOWN FAM99A

CTD_2589H19.61.575509316 -1.185534739 7.010999624 4.33E-11 3.29E-10 14.73156948 UP CTD-2589H19.6

DBH_AS1 -1.51885094 3.030890965 -7.009309715 4.37E-11 3.32E-10 14.41431395 DOWN DBH-AS1

HM13_AS1 -1.293985715 -2.753336476 -7.006307609 4.45E-11 3.37E-10 14.77526247 DOWN HM13-AS1

KLHL32 -1.480032039 -1.569530883 -7.005100381 4.48E-11 3.39E-10 14.74068125 DOWN KLHL32

MIR325HG -2.625583823 -3.337563667 -6.99753757 4.67E-11 3.54E-10 14.72926076 DOWN MIR325HG

C3orf80 1.938242002 -1.769382904 6.995043045 4.74E-11 3.58E-10 14.66525496 UP C3orf80

DIRAS1 2.527121069 -2.152447628 6.994655709 4.75E-11 3.59E-

10	14.66659666	UP	DIRAS1
KANSL1_AS1	-1.256414802	0.789101057	-6.993357088	4.79E-11	3.61E-10	14.54643924	DOWN	KANSL1-AS1
SULT1A1	-1.416744583	5.763373743	-6.989971569	4.88E-11	3.68E-10	14.08894949	DOWN	SULT1A1
RP11_563D10.1	-2.414428495	-3.068566956	-6.988912664	4.91E-11	3.70E-10	14.68038365	DOWN	RP11-563D10.1
RP11_545I5.3	-1.076117942	0.031879194	-6.988035208	4.93E-11	3.72E-10	14.56653669	DOWN	RP11-545I5.3
LRRC37A5P	-1.549048693	-1.796345332	-6.986114447	4.98E-11	3.75E-10	14.64426149	DOWN	LRRC37A5P
LINC00511	2.43986247	0.064860431	6.983278352	5.06E-11	3.81E-10	14.49434966	UP	LINC00511
EVC	2.290563432	1.642152608	6.981424039	5.12E-11	3.85E-10	14.35227271	UP	EVC
CCL26	2.086222295	-3.177688287	6.979682007	5.17E-11	3.89E-10	14.61410084	UP	CCL26
S100A2	2.002610896	-1.82310231	6.978247779	5.21E-11	3.92E-10	14.57359361	UP	S100A2
CTD_3099C6.9	1.976396546	-2.775850291	6.977540354	5.23E-11	3.93E-10	14.59596813	UP	CTD-3099C6.9
AADAT	-1.709743352	2.011791169	-6.977133116	5.24E-11	3.94E-10	14.35881024	DOWN	AADAT
C12orf56	2.573892029	-3.819491296	6.972440838	5.38E-11	4.04E-10	14.58111988	UP	C12orf56
HMGN5	-1.641866161	1.188048935	-6.972296469	5.39E-11	4.04E-10	14.40720276	DOWN	HMGN5
MLIP_AS1	-2.135797936	-3.811180614	-6.970128197	5.45E-11	4.09E-10	14.58286479	DOWN	MLIP-AS1
IL12A	1.588835798	-3.052165663	6.969077686	5.49E-11	4.11E-10	14.55932495	UP	IL12A
RNF128	-1.021968853	6.191056649	-6.967915541	5.52E-11	4.14E-10	13.95689655	DOWN	RNF128

RIPPLY3 1.937008424 -2.600887964 6.966536955 5.56E-11 4.17E-10 14.53197525 UP RIPPLY3
EPB41L4B -1.42636259 5.141077825 -6.964434551 5.63E-11 4.21E-10 13.96993441 DOWN EPB41L4B
RP4_601P9.2 -2.823184203 -2.343090944 -6.960753065 5.75E-11 4.30E-10 14.51580037 DOWN RP4-601P9.2
FMNL2 1.333424062 3.123009271 6.960502811 5.76E-11 4.30E-10 14.08898674 UP FMNL2
IZUMO4 -1.288746911 0.592275268 -6.958467035 5.82E-11 4.35E-10 14.36990355 DOWN IZUMO4
JMJD1C_AS1 1.221787032 -2.49306639 6.957755313 5.85E-11 4.36E-10 14.48942279 UP JMJD1C-AS1
SLC22A20 1.346353144 -2.685013107 6.95675477 5.88E-11 4.38E-10 14.48727723 UP SLC22A20
SPATA17 2.130147349 -2.33773783 6.954329616 5.96E-11 4.44E-10 14.45646289 UP SPATA17
AMPD3 1.141135284 1.228704996 6.954084147 5.97E-11 4.45E-10 14.25553814 UP AMPD3
RP11_519G16.5 1.929131479 -4.71709608 6.953861119 5.98E-11 4.45E-10 14.49317247 UP RP11-519G16.5
C16orf93 1.206419393 -2.560011503 6.951372179 6.06E-11 4.51E-10 14.45663822 UP C16orf93
APOE -1.427572154 12.17553439 -6.950044431 6.11E-11 4.54E-10 13.96840315 DOWN APOE
GUCY1B2 2.409808554 -3.198652312 6.948864636 6.15E-11 4.57E-10 14.44192215 UP GUCY1B2
LINC00664 1.759509697 -3.726106034 6.947145618 6.21E-11 4.61E-10 14.44947428 UP LINC00664
RP11_501C14.7 2.070225881 -4.286376349 6.947084268 6.21E-11 4.61E-10 14.45321574 UP RP11-501C14.7
RP11_1055B8.9 1.63456251 -2.839563252 6.946539669 6.23E-11 4.62E-10 14.430773 UP RP11-1055B8.9
AQP11 -1.222990888 3.174530837 -6.944676949 6.29E-11 4.67E-10

14.03119599DOWN AQP11

AOX3P -2.259094371 -2.297784095 -6.944456259 6.30E-11 4.67E-10 14.42830234 DOWN AOX3P

SLC41A1 1.267689539 3.1708411466.942677248 6.36E-11 4.72E-10 13.98539832 UP SLC41A1

JAM2 -1.209209348 2.102033677 -6.941928862 6.39E-11 4.74E-10 14.14346 DOWN JAM2

RP1_292L20.3 -1.173433093 -1.26780563 -6.94173521 6.40E-11 4.74E-10 14.37981787 DOWN RP1-292L20.3

BAIAP2 -1.042161571 5.861273571 -6.940653227 6.44E-11 4.77E-10 13.81097033 DOWN BAIAP2

LRRC8B 1.219034427 2.422457273 6.939662058 6.47E-11 4.79E-10 14.04982186 UP LRRC8B

UPP2 -3.186462515 0.072146075 -6.936941992 6.57E-11 4.86E-10 14.31005075 DOWN UPP2

AC002116.7 1.180559712 -2.556860175 6.936831744 6.58E-11 4.86E-10 14.37710898 UP AC002116.7

SIX3 1.901664263 -5.347007921 6.935080653 6.64E-11 4.91E-10 14.39116919UP SIX3

RP5_1021I20.1 -1.022227047 0.219154885 -6.934906493 6.65E-11 4.91E-10 14.26229782 DOWN RP5-1021I20.1

BCL11A 1.944881733 -1.221078353 6.93320696 6.71E-11 4.95E-10 14.30305474 UP BCL11A

DTX3 1.231421228 2.1159870236.93223784 6.75E-11 4.98E-10 14.04232844 UP DTX3

SLC25A18 -1.911124119 4.644476419 -6.930651947 6.81E-11 5.02E-10 13.8254697 DOWN SLC25A18

KIAA1614 1.213527318 0.177374675 6.929779264 6.84E-11 5.04E-10 14.20761346 UP KIAA1614

FOLH1 -1.631992787 2.593881452 -6.926214226 6.98E-11 5.14E-10 14.01377407 DOWN FOLH1

RP11_104J23.1 -1.790780432 -1.555153805 -6.92393223 7.07E-11 5.21E-10 14.29765568 DOWN RP11-104J23.1

ABCB5	2.663393993	-3.678950459	6.922594242	7.12E-11	5.24E-10	14.30493374	UP	ABCB5
NPTX1	2.247278797	-3.634071514	6.920078813	7.22E-11	5.31E-10	14.29577009	UP	NPTX1
NCS1	1.441944324	2.524978754	6.91772099	7.32E-11	5.38E-10	13.91664462	UP	NCS1
LRRC75A	1.06390039	-0.967603878	6.917416252	7.33E-11	5.39E-10	14.21272264	UP	LRRC75A
MGC27382	-1.751207694	-1.723457395	-6.914707067	7.44E-11	5.47E-10	14.25299372	DOWN	MGC27382
DLK2	1.321496362	0.01338713	6.913486246	7.49E-11	5.50E-10	14.12823731	UP	DLK2
ZNF826P	2.110555588	-0.748442801	6.910381022	7.63E-11	5.59E-10	14.1520492	UP	ZNF826P
LINC00675	-1.755633537	-4.024743696	-6.909676443	7.66E-11	5.61E-10	14.25423648	DOWN	LINC00675
NTN1	-1.804128383	2.818259363	-6.907547194	7.75E-11	5.67E-10	13.88925024	DOWN	NTN1
TRPC5	-2.514530795	-3.426430525	-6.906119936	7.81E-11	5.71E-10	14.23172142	DOWN	TRPC5
RP11_705C15.3	1.210463281	-1.049756188	6.905457093	7.84E-11	5.73E-10	14.15037952	UP	RP11-705C15.3
ZNF675	1.090781226	0.876947566	6.905342061	7.84E-11	5.73E-10	14.02010723	UP	ZNF675
ACPT	2.150906097	-3.642885199	6.905165409	7.85E-11	5.74E-10	14.21515782	UP	ACPT
HHLA3	-1.050108358	2.805599785	-6.904553343	7.88E-11	5.75E-10	13.85164183	DOWN	HHLA3
BPIFA2	2.44889046	-4.520903717	6.904532752	7.88E-11	5.75E-10	14.22128043	UP	BPIFA2
RP11_749I16.3	1.41585856	-4.049858554	6.899798473	8.09E-11	5.90E-10	14.19758599	UP	RP11-749I16.3
PRSS36	-1.018455996	0.288393003	-6.893780813	8.37E-11	6.09E-10			

14.03297644 DOWN PRSS36

RP11_122K13.7 -1.847897626 -2.228750723 -6.893614615 8.38E-11 6.09E-10 14.15124684 DOWN RP11-122K13.7

GNAO1 -2.664514946 1.494086118 -6.89360797 8.38E-11 6.09E-10 13.96883851 DOWN GNAO1

ARHGAP4 1.129321352 4.371429719 6.892011133 8.45E-11 6.14E-10 13.59989697 UP ARHGAP4

LL22NC03_104C7.1 -2.537328869 -2.61433547 -6.891655573 8.47E-11 6.15E-10 14.14452136 DOWN LL22NC03-104C7.1

STOX1 1.871768094 -1.45803869 6.885362847 8.77E-11 6.37E-10 14.05366641 UP STOX1

CYP17A1 -3.567895482 2.467521334 -6.88428181 8.82E-11 6.41E-10 13.85742827 DOWN CYP17A1

TEAD4 1.32106171 2.044160443 6.883433822 8.87E-11 6.43E-10 13.78112891 UP TEAD4

RP11_231P20.2 -2.290029208 1.159149589 -6.880155229 9.03E-11 6.54E-10 13.91465881 DOWN RP11-231P20.2

CES1P1 -2.583766491 1.819368862 -6.875552196 9.26E-11 6.71E-10 13.83864177 DOWN CES1P1

GRM4 1.991760877 -4.499708649 6.874861432 9.30E-11 6.74E-10 14.06271497 UP GRM4

CTD_2024P10.2 -1.238202565 -2.718903982 -6.87242368 9.43E-11 6.82E-10 14.04531095 DOWN CTD-2024P10.2

PID1 -1.169710536 4.856752522 -6.870907504 9.51E-11 6.88E-10 13.46532763 DOWN PID1

PRAMEF11 -1.406015232 -5.919523932 -6.869129285 9.60E-11 6.95E-10 14.03190277 DOWN PRAMEF11

C20orf62 -1.437882545 -5.415640093 -6.868737256 9.62E-11 6.96E-10 14.03120691 DOWN C20orf62

A1CF -1.601166068 6.94142887 -6.867808137 9.67E-11 6.99E-10 13.40458742 DOWN A1CF

AOX2P -1.847499046 -3.943626674 -6.867763568 9.68E-11 6.99E-10 14.02678074 DOWN AOX2P

TDO2 -2.603781795 5.870374947 -6.86669138 9.73E-11 7.03E-10
13.42694885 DOWN TDO2
USP2 -1.588105785 2.536587209 -6.865865475 9.78E-11 7.06E-10
13.6890301 DOWN USP2
ZNF845 1.093712672 1.165784925 6.86543648 9.80E-11 7.08E-10
13.77610561 UP ZNF845
CCDC136 1.333134267 -2.162379429 6.86325389 9.92E-11 7.16E-10
13.96517594 UP CCDC136
AC092071.1 -2.348094258 -4.411383401 -6.862162787 9.98E-11 7.20E-
10 13.99616317 DOWN AC092071.1
ABCA12 1.595993574 -3.957908658 6.861063485 1.00E-10 7.24E-
10 13.985849 UP ABCA12
SMC4 1.311396644 3.319022203 6.857998918 1.02E-10 7.36E-10
13.50562377 UP SMC4
SLC38A2 -1.00010288 7.441178001 -6.854851448 1.04E-10 7.48E-10
13.33527617 DOWN SLC38A2
C6orf201 -1.094128395 -2.261874691 -6.852008842 1.06E-10 7.59E-
10 13.92566374 DOWN C6orf201
SUCLG2P2 -1.551034929 -2.201671075 -6.851886 1.06E-10 7.60E-10
13.92438697 DOWN SUCLG2P2
MMP14 1.196455468 5.797901516 6.851255197 1.06E-10 7.62E-10
13.32194681 UP MMP14
TBX3 -2.013736271 4.638217591 -6.849600933 1.07E-10 7.69E-10
13.38487643 DOWN TBX3
RP11_264M12.2 -1.371319331 -1.379661643 -6.849353189 1.07E-10
7.69E-10 13.88321569 DOWN RP11-264M12.2
TRPM2 1.130453131 1.4996766 6.848874048 1.08E-10 7.71E-10
13.65232423 UP TRPM2
DLX6_AS1 2.713751693 -4.093260048 6.84798914 1.08E-10 7.75E-10
13.90687572 UP DLX6-AS1
RP11_685F15.1 -1.593228113 -5.882417887 -6.846549867 1.09E-10
7.80E-10 13.90968537 DOWN RP11-685F15.1
FAM13A -1.588645676 3.581225605 -6.842367337 1.11E-10 7.98E-

10	13.43760468	DOWN FAM13A

UBASH3B	1.358983456	1.210444701	6.842085048	1.12E-10	7.99E-10	13.64041753	UP	UBASH3B

MTHFD2	1.122485041	2.078970316	6.841315266	1.12E-10	8.02E-10	13.54920171	UP	MTHFD2

FAT4	-1.716653078	1.812904716	-6.839758645	1.13E-10	8.09E-10	13.6267001	DOWN FAT4

RPSAP54	1.136662391	-2.739531363	6.839286199	1.13E-10	8.10E-10	13.85200402	UP	RPSAP54

TTC39A	1.959124083	2.300301875	6.839091176 1.14E-10	8.11E-10	13.50820565	UP	TTC39A

AC073321.4 -1.760797814	-4.971447254	-6.837871323	1.14E-10	8.16E-10	13.86485191	DOWN AC073321.4

ZNF300 1.622355596	0.201448858	6.836538895	1.15E-10	8.22E-10	13.69187089	UP	ZNF300

CCDC185	1.694078266	-5.348987209	6.832338238	1.18E-10	8.41E-10	13.83436749	UP	CCDC185

NCEH1 1.427916433	2.499207661	6.829481576	1.20E-10	8.54E-10	13.43646345	UP	NCEH1

RP5_881L22.6	-1.802863502	-3.833030764	-6.828376869	1.21E-10	8.59E-10	13.81377675	DOWN RP5-881L22.6

APOH	-2.409059086	10.94869881	-6.828067434	1.21E-10	8.60E-10	13.2585099	DOWN APOH

UCHL1 2.91789255	0.555685489	6.827836602	1.21E-10	8.61E-10	13.60078642	UP	UCHL1

OLFM2 -1.521310461	5.490947098	-6.826579017	1.22E-10	8.67E-10	13.19987874	DOWN OLFM2

ZNF701 1.338375253	0.462636803	6.825290608	1.23E-10	8.72E-10	13.61422839	UP	ZNF701

FAM182B	1.50607162	-2.987553128	6.823538387	1.24E-10	8.80E-10	13.76825811 UP	FAM182B

RP11_685B14.3 -1.704793426	-4.526044651	-6.821691832	1.25E-10	8.89E-10	13.77808626	DOWN RP11-685B14.3

PSAPL1	2.041110146	-4.937542642	6.821655019	1.25E-10	8.89E-10	13.77625351	UP	PSAPL1
B3GNT8	1.496995279	-0.429964856	6.819153946	1.27E-10	9.00E-10	13.642026	UP	B3GNT8
JAKMIP2_AS1	-2.220836869	-4.47592811	-6.818396924	1.27E-10	9.04E-10	13.75988381	DOWN	JAKMIP2-AS1
IFI27L2	1.304473828	2.10666885	6.817839494	1.28E-10	9.06E-10	13.41614334	UP	IFI27L2
RP5_834N19.1	-2.106317892	-2.394104831	-6.816988621	1.28E-10	9.10E-10	13.73878902	DOWN	RP5-834N19.1
VTN	-1.67229519	11.81703008	-6.81452025	1.30E-10	9.22E-10	13.21280995	DOWN	VTN
SGSM1	1.672637322	-0.620688088	6.813423441	1.31E-10	9.27E-10	13.62145308	UP	SGSM1
CA2	-1.46334534	5.160276161	-6.811543359	1.32E-10	9.36E-10	13.13062305	DOWN	CA2
SLCO4C1	2.880582808	-1.109308144	6.811540963	1.32E-10	9.36E-10	13.62567853	UP	SLCO4C1
MNX1	2.885716488	-3.899693204	6.811269293	1.33E-10	9.36E-10	13.70341815	UP	MNX1
TRIM55	-3.826000651	2.747354212	-6.810290035	1.33E-10	9.41E-10	13.43147712	DOWN	TRIM55
MIR1295A	-1.800283876	-4.097928737	-6.809723592	1.34E-10	9.44E-10	13.71352623	DOWN	MIR1295A
FP325317.1	-1.991741758	-4.308782377	-6.806684853	1.36E-10	9.59E-10	13.69706501	DOWN	FP325317.1
RP11_1151B14.3	-1.89522995	2.939669	-6.804137208	1.38E-10	9.72E-10	13.31291089	DOWN	RP11-1151B14.3
EPHA6	2.452746452	-4.661222101	6.803692212	1.38E-10	9.74E-10	13.67710942	UP	EPHA6
LINC01558	-1.883694881	1.069243583	-6.801945155	1.40E-10	9.83E-10	13.48918785	DOWN	LINC01558
PAQR8	1.329873272	1.862806709	6.799238868	1.42E-10	9.97E-10

13.34099459 UP PAQR8
STEAP3 -1.527688886 5.990848339 -6.798767154 1.42E-10 9.99E-10 13.03471575 DOWN STEAP3
ITIH4_AS1 -1.499615223 -2.064518915 -6.79717844 1.43E-10 1.01E-09 13.62539509 DOWN ITIH4-AS1
OR13A1 1.688796706 -5.123642145 6.796954484 1.43E-10 1.01E-09 13.64381423 UP OR13A1
RP11_326C3.12 -1.488737191 -1.607711133 -6.796814859 1.44E-10 1.01E-09 13.6088424 DOWN RP11-326C3.12
SLC35F3 2.275660749 -3.500338652 6.79354996 1.46E-10 1.03E-09 13.60802007 UP SLC35F3
WASF1 1.072634731 2.761052848 6.791324088 1.48E-10 1.04E-09 13.20102441 UP WASF1
GPR125 -1.07830399 5.098493804 -6.789044719 1.50E-10 1.05E-09 13.00429688 DOWN GPR125
CYP3A5 -1.814497706 7.061077369 -6.787759225 1.51E-10 1.06E-09 12.96813064 DOWN CYP3A5
RNF217_AS1 -1.543929783 -2.701556512 -6.784846183 1.53E-10 1.08E-09 13.5719147 DOWN RNF217-AS1
RP1_65J11.1 -1.715833131 -3.714738487 -6.784777002 1.54E-10 1.08E-09 13.5787943 DOWN RP1-65J11.1
IDO2 -2.600664586 -1.95253741 -6.783377953 1.55E-10 1.08E-09 13.54824219 DOWN IDO2
URAHP -1.224359545 1.380667865 -6.781890324 1.56E-10 1.09E-09 13.34159896 DOWN URAHP
LINC00342 1.018772743 0.985879428 6.778885847 1.59E-10 1.11E-09 13.32286124 UP LINC00342
LRG1 -1.77775002 8.219314556 -6.777730763 1.60E-10 1.12E-09 12.92374269 DOWN LRG1
RP13_616I3.1 -1.831560061 -0.952476679 -6.776967383 1.60E-10 1.12E-09 13.47727328 DOWN RP13-616I3.1
GJB1 -1.564759446 8.028778671 -6.774703643 1.62E-10 1.13E-09 12.90457291 DOWN GJB1

RGS2 1.552144077 3.3575175116.771887624 1.65E-10 1.15E-09
13.03458033 UP RGS2
TNFRSF18 1.557080115-0.226204651 6.7701241161.66E-10 1.16E-09
13.3627356 UP TNFRSF18
CTD_2201E18.5 1.349165812 -3.975801421 6.769880623 1.67E-10
1.16E-09 13.49584109 UP CTD-2201E18.5
CLRN2 -1.359749019 -5.717183998 -6.769212056 1.67E-10 1.17E-09
13.49386942 DOWN CLRN2
PROM1 3.45391033 -1.334576789 6.767506316 1.69E-10 1.18E-09
13.39171348 UP PROM1
PLAC1 1.621479845 -5.052643104 6.76596891 1.70E-10 1.19E-09
13.47741789 UP PLAC1
LL22NC03_32F9.1 -1.664132448 -3.786624165 -6.762819852 1.73E-10
1.21E-09 13.46108921 DOWN LL22NC03-32F9.1
IFNLR1 -1.786173756 2.190733666 -6.76199685 1.74E-10 1.21E-09
13.16637993 DOWN IFNLR1
U91324.1 -1.959398066 -0.737450991 -6.761513255 1.75E-10 1.21E-
09 13.38517653 DOWN U91324.1
CLDN2 -3.085082008 4.173353895 -6.761472169 1.75E-10 1.21E-09
12.99871575 DOWN CLDN2
NCOA7_AS1 -1.636524526 -5.149750228 -6.760296771 1.76E-10
1.22E-09 13.44717138 DOWN NCOA7-AS1
ADM2 1.662137892 2.952413034 6.760087463 1.76E-10 1.22E-09
13.01175084UP ADM2
ULBP1 1.872156663 -2.912842626 6.758558294 1.77E-10 1.23E-09
13.41256264 UP ULBP1
AP000347.2 1.024330639 -1.392625329 6.755634058 1.80E-10 1.25E-
09 13.3570853 UP AP000347.2
CYP2A13 -3.362848176 -2.235605629 -6.754583863 1.81E-10 1.26E-
09 13.39505834 DOWN CYP2A13
DMC1 1.536202776 -2.775959531 6.753946636 1.82E-10 1.26E-09
13.3884996 UP DMC1
HRK 1.641184093-4.979334965 6.751393439 1.85E-10 1.28E-09

13.39923497 UP HRK

GXYLT1P6 -1.883044631 -4.972056815 -6.750335188 1.86E-10 1.29E-09 13.3938204 DOWN GXYLT1P6

ATP5A1P3 -1.412549362 -3.044111145 -6.748980708 1.87E-10 1.29E-09 13.38352192 DOWN ATP5A1P3

ARL4C 1.222267493 3.978351458 6.748393991 1.88E-10 1.30E-09 12.84665659 UP ARL4C

CTD_2561B21.8 1.070441127-1.754402865 6.74752353 1.89E-10 1.30E-09 13.32846633 UP CTD-2561B21.8

SP8 2.826704572 -4.335770937 6.746619491 1.90E-10 1.31E-09 13.36393006 UP SP8

PABPC1L 1.024292315 3.833443898 6.742649581 1.94E-10 1.34E-09 12.82598703 UP PABPC1L

PFKP 1.692710425 3.133132567 6.742236856 1.94E-10 1.34E-09 12.89681669 UP PFKP

RPS6KA2 -1.00644125 3.602066289 -6.741755826 1.95E-10 1.34E-09 12.8670295 DOWN RPS6KA2

CTD_2026K11.4-1.178244437 -2.185582557 -6.737518347 1.99E-10 1.37E-09 13.30729204 DOWN CTD-2026K11.4

MDK 1.9206111566.1366916116.737385194 1.99E-10 1.37E-09 12.70411388UP MDK

DKKL1 1.559180048 -2.916790944 6.736807905 2.00E-10 1.38E-09 13.29925618 UP DKKL1

RENBP 1.416020443 2.658159137 6.73628191 2.01E-10 1.38E-09 12.91283805 UP RENBP

NDUFA6_AS1 -1.5216507 1.635607976 -6.733993641 2.03E-10 1.40E-09 13.06484748 DOWN NDUFA6-AS1

CLEC1A -1.041167276 0.0792112 -6.733080557 2.04E-10 1.40E-09 13.17624607 DOWN CLEC1A

LRRC56 1.00216098 0.1140886676.73191824 2.06E-10 1.41E-09 13.14043273 UP LRRC56

KB_1572G7.2 1.14533436 -1.704226173 6.731646032 2.06E-10 1.41E-09 13.24028264 UP KB-1572G7.2

HTR2A -1.575753666 -3.98418574 -6.728486251 2.10E-10 1.44E-09 13.27752541 DOWN HTR2A
RP11_95O2.5 1.373131618 -3.798505752 6.72845863 2.10E-10 1.44E-09 13.27189382 UP RP11-95O2.5
EPCAM 4.135757843 1.257763282 6.726638257 2.12E-10 1.45E-09 12.99198521 UP EPCAM
AC073842.19 -1.907685129 -2.318042045 -6.726061935 2.12E-10 1.46E-09 13.2493648 DOWN AC073842.19
TMEM243 1.031874811 1.557917283 6.723975977 2.15E-10 1.47E-09 12.96996304 UP TMEM243
PTPRN 1.645205122 -4.140027065 6.721583751 2.18E-10 1.49E-09 13.23687845 UP PTPRN
FXYD3 2.780539678 -0.447378432 6.720878968 2.19E-10 1.50E-09 13.09806329 UP FXYD3
ADRB1 -1.955043475 -2.403110586 -6.720697456 2.19E-10 1.50E-09 13.2220855 DOWN ADRB1
CCDC64 1.53235257 2.555949003 6.719951368 2.20E-10 1.50E-09 12.83538573 UP CCDC64
RP11_107N15.1 1.363835911 -5.345003091 6.719401157 2.20E-10 1.51E-09 13.22807442 UP RP11-107N15.1
PRAMEF2 -2.048230314 -5.190185405 -6.71883636 2.21E-10 1.51E-09 13.22463018 DOWN PRAMEF2
CREB3L3 -2.110374264 6.975730179 -6.717604957 2.22E-10 1.52E-09 12.58883632 DOWN CREB3L3
AC104809.2 -3.275739296 -2.875426573 -6.715456034 2.25E-10 1.54E-09 13.19508551 DOWN AC104809.2
DPH6_AS1 -1.74674321 -2.446173209 -6.714024586 2.27E-10 1.55E-09 13.18769144 DOWN DPH6-AS1
PDE9A 1.779467963 2.347341188 6.711862061 2.30E-10 1.57E-09 12.81309028 UP PDE9A
RP11_361L15.5 -1.723385531 -3.333838222 -6.711569109 2.30E-10 1.57E-09 13.18495219 DOWN RP11-361L15.5
RP11_1113L8.1 -1.663438155 -1.401334955 -6.711125733 2.31E-10

1.57E-09 13.14136947 DOWN RP11-1113L8.1

RP11_74C13.3 -1.642892064 -3.869801998 -6.710825862 2.31E-10

1.57E-09 13.18295984 DOWN RP11-74C13.3

AC114812.10 -1.709143272 -4.546462505 -6.71025376 2.32E-10 1.58E-09 13.18009135 DOWN AC114812.10

PPP2R3A 1.269655563 1.116260523 6.707785919 2.35E-10 1.60E-09

12.92385734 UP PPP2R3A

MBL1P -1.980632685 -1.735415378 -6.706974763 2.36E-10 1.61E-09

13.13305202 DOWN MBL1P

DNAJB13 1.632408154 -3.878259511 6.70642356 2.37E-10 1.61E-09

13.15324304 UP DNAJB13

LMOD1 -1.481165003 2.542597415 -6.705012664 2.38E-10 1.62E-09

12.81052261 DOWN LMOD1

DUSP13 2.065976966 -4.321160204 6.704172917 2.40E-10 1.63E-09 13.14282992 UP DUSP13

GAS5 1.011346989 6.497189534 6.703454126 2.41E-10 1.64E-09

12.51204927 UP GAS5

ASPA -1.617508571 0.642304664 -6.701519331 2.43E-10 1.65E-09

12.97518081 DOWN ASPA

LINC00676 -1.346865296 -5.830559821 -6.700373324 2.45E-10 1.66E-09 13.12508692 DOWN LINC00676

RP11_326C3.15 -1.503869431 -2.925035866 -6.699920164 2.45E-10

1.67E-09 13.11973476 DOWN RP11-326C3.15

CLEC5A 1.983356159 -2.293002341 6.699128662 2.46E-10 1.67E-09 13.07734735 UP CLEC5A

RND1 -1.462791487 4.879160684 -6.698040688 2.48E-10 1.68E-09

12.52953558 DOWN RND1

DLEC1 -1.046774549 -0.854941905 -6.696717959 2.50E-10 1.69E-09

13.03399793 DOWN DLEC1

ZNF256 1.140840644 0.656352593 6.695831734 2.51E-10 1.70E-09

12.90153695 UP ZNF256

RP4_798P15.3 -1.680674428 0.767273377 -6.694102331 2.53E-10

1.72E-09 12.92746356 DOWN RP4-798P15.3

FAM149A	-1.296693624	3.478230299	-6.693113188	2.55E-10	1.73E-09	12.62765856	DOWN	FAM149A
SLC7A2	-1.54323251	6.384179684	-6.686670692	2.64E-10	1.78E-09	12.42139459	DOWN	SLC7A2
PTK7	1.869476362	2.53874354	6.686411089	2.64E-10	1.79E-09	12.65638509	UP	PTK7
RP4_564F22.6	-2.010614561	0.860537012	-6.685330838	2.66E-10	1.80E-09	12.87831074	DOWN	RP4-564F22.6
TRAPPC13P1	-2.491625428	-3.430199136	-6.684505354	2.67E-10	1.80E-09	13.03926963	DOWN	TRAPPC13P1
CTC_435M10.12	1.168262581	-2.637260172	6.678493298	2.76E-10	1.86E-09	12.98572216	UP	CTC-435M10.12
AP000355.2	-2.270031253	-1.022222048	-6.678150502	2.76E-10	1.86E-09	12.95411425	DOWN	AP000355.2
AC108078.1	-2.258043357	-1.303616444	-6.678013737	2.77E-10	1.87E-09	12.96457905	DOWN	AC108078.1
TUSC8	-2.542026754	0.422284852	-6.669807007	2.89E-10	1.95E-09	12.83348174	DOWN	TUSC8
ZNF737	1.542856574	-0.62370399	6.669786052	2.89E-10	1.95E-09	12.85002395	UP	ZNF737
CECR6	1.154294828	-1.980462828	6.669178795	2.90E-10	1.96E-09	12.91688193	UP	CECR6
BNIP3P1	-1.106232463	-1.993105193	-6.666322773	2.95E-10	1.99E-09	12.92068073	DOWN	BNIP3P1
EFNA3	1.290938314	0.728088926	6.665386163	2.96E-10	2.00E-09	12.72979334	UP	EFNA3
ISYNA1	1.531120346	3.805574461	6.661989649	3.02E-10	2.03E-09	12.39896881	UP	ISYNA1
CLTCL1	-1.09370744	3.305232528	-6.659775503	3.06E-10	2.05E-09	12.46179076	DOWN	CLTCL1
QRFP	1.202863047	-3.715938969	6.658895506	3.07E-10	2.06E-09	12.90118173	UP	QRFP
CCDC170	-2.02051173	1.518688398	-6.658652759	3.08E-10	2.07E-09			

12.68149698 DOWN CCDC170
SPATA18 -2.051006949 0.765347511-6.654693196 3.14E-10 2.11E-09 12.72275541 DOWN SPATA18
RABEPK -1.019097632 5.113459495-6.654204565 3.15E-10 2.11E-09 12.27222609 DOWN RABEPK
AJ006998.2 -2.663847708 -2.150920486 -6.65159899 3.20E-10 2.14E-09 12.84682244 DOWN AJ006998.2
RP11_488L18.4 1.169396522 2.279814331 6.649726644 3.23E-10 2.17E-09 12.49028799 UP RP11-488L18.4
CTB_78F1.2 -2.475482549 -3.445380925 -6.647431134 3.27E-10 2.19E-09 12.84222328 DOWN CTB-78F1.2
LINC00399 -1.640793639 -4.595839726 -6.646634889 3.29E-10 2.20E-09 12.8413837 DOWN LINC00399
RP11_404P21.3 2.677960931 -1.300559632 6.646274678 3.29E-10 2.20E-09 12.74918357 UP RP11-404P21.3
RP11_15A1.4 1.31371376 -3.242975947 6.645481289 3.31E-10 2.21E-09 12.82151443 UP RP11-15A1.4
GDPD3 1.136055916 0.062255574 6.644474557 3.32E-10 2.22E-09 12.67255551 UP GDPD3
HOMER1 1.671695251 0.946796144 6.642880739 3.35E-10 2.24E-09 12.58407906 UP HOMER1
VN1R48P 1.818392296 -4.251493118 6.64279513 3.36E-10 2.24E-09 12.81709548 UP VN1R48P
CYP2B7P -3.604704223 2.88900009 -6.642427756 3.36E-10 2.25E-09 12.50987855 DOWN CYP2B7P
ENPP5 2.802467077 -1.000767903 6.641844718 3.37E-10 2.25E-09 12.70919923 UP ENPP5
ST3GAL6_AS1 -1.257815282 -1.812848103 -6.640249935 3.40E-10 2.27E-09 12.77659505 DOWN ST3GAL6-AS1
PEG10 4.029702474 2.823238126 6.639570982 3.42E-10 2.28E-09 12.41110824UP PEG10
RP11_104J23.2 -1.614266706 -2.93271764 -6.639446501 3.42E-10 2.28E-09 12.79775026 DOWN RP11-104J23.2

HIST1H2BH 1.553241219 -4.661728192 6.638047677 3.44E-10
2.30E-09 12.79475454 UP HIST1H2BH
NAT14 1.108497099 2.4254481166.637945252 3.45E-10 2.30E-09
12.41051606 UP NAT14
DPF1 1.432360627 -3.338174764 6.637786607 3.45E-10 2.30E-09
12.78116276UP DPF1
RP11_108K14.12 -2.405378095 -2.297921771 -6.63655004 3.47E-10
2.31E-09 12.77027526 DOWN RP11-108K14.12
PRTFDC1 1.212240178 0.673754414 6.636133475 3.48E-10 2.32E-09 12.57992539 UP PRTFDC1
KCP 1.444233672 -1.107719131 6.634195445 3.52E-10 2.34E-09
12.68845847 UP KCP
CCDC183 1.172540651 -0.111714117 6.633858552 3.52E-10 2.34E-09 12.62789698 UP CCDC183
RNU6ATAC35P -1.499135831 -2.805301306 -6.633004885 3.54E-10
2.35E-09 12.76232179 DOWN RNU6ATAC35P
LRRC16A 1.366473443 2.2831145416.632709571 3.55E-10 2.36E-09
12.39672412 UP LRRC16A
TTBK1 -1.81637627 1.215311791-6.630247123 3.59E-10 2.39E-09
12.55125594 DOWN TTBK1
LA16c_313D11.12 1.074096414 -2.45971342 6.6299110143.60E-10 2.39E-09 12.72411053UP LA16c-313D11.12
SHD -2.544767463 0.610096615 -6.629181359 3.62E-10 2.40E-09
12.60479282 DOWN SHD
SLC22A10 -2.60382259 2.410331345 -6.628298067 3.63E-10 2.41E-09
12.44493112DOWN SLC22A10
ZFP82 1.361981086 0.333514194 6.627043684 3.66E-10 2.42E-09
12.55652002 UP ZFP82
ADORA2A_AS1 -1.799232991 3.689835426 -6.622836743 3.74E-10
2.48E-09 12.24075957 DOWN ADORA2A-AS1
ZBTB32 1.24798691 -2.370019503 6.621023908 3.78E-10 2.50E-09
12.67187138 UP ZBTB32
CRYZ -1.054797871 6.853569396 -6.62095714 3.78E-10 2.50E-09

12.06414064 DOWN CRYZ

SPTBN2 -1.66803095 4.143476243 -6.618884376 3.82E-10 2.53E-09 12.16912987 DOWN SPTBN2

CTC_559E9.5 1.147755312 -0.806718885 6.616779503 3.87E-10 2.56E-09 12.58232872 UP CTC-559E9.5

FBLN1 1.656258628 4.604770009 6.616296128 3.88E-10 2.56E-09 12.09692086 UP FBLN1

CCDC114 1.341310916 -2.413942364 6.615693727 3.89E-10 2.57E-09 12.64420476 UP CCDC114

XYLB -1.199537451 4.57020687 -6.613289331 3.94E-10 2.60E-09 12.08611413 DOWN XYLB

NPAS1 1.582795342 -1.598728782 6.613149012 3.95E-10 2.60E-09 12.59839955 UP NPAS1

CTD_2008L17.2 2.177800049 -4.48874954 6.608480709 4.05E-10 2.67E-09 12.63513181 UP CTD-2008L17.2

CCDC146 -1.244378506 1.77665953 -6.603857159 4.15E-10 2.73E-09 12.3471532 DOWN CCDC146

DGAT2 -1.559575523 6.389618109 -6.602980459 4.17E-10 2.74E-09 11.97149354 DOWN DGAT2

GALNT6 1.039790338 0.841694611 6.602437167 4.18E-10 2.75E-09 12.38738664 UP GALNT6

EPS8L3 3.03650401 1.923794671 6.60231636 4.19E-10 2.75E-09 12.26749646 UP EPS8L3

LINC01138 1.010306982 1.392254563 6.601930504 4.20E-10 2.76E-09 12.33300405 UP LINC01138

AC084219.4 1.452560178 -3.81624243 6.600867604 4.22E-10 2.77E-09 12.59278645 UP AC084219.4

PDE7B -1.149643807 1.051754568 -6.59943605 4.25E-10 2.79E-09 12.39005448 DOWN PDE7B

MAP7D2 2.553635822 -1.367985993 6.599324611 4.26E-10 2.79E-09 12.50365581 UP MAP7D2

KCNJ11 1.877107872 0.297099863 6.598866604 4.27E-10 2.80E-09 12.40260388 UP KCNJ11

CPNE9 1.351945981 -3.975367971 6.597831065 4.29E-10 2.81E-09 12.57906723 UP CPNE9

PDP1 1.325167006 2.129915472 6.596410356 4.32E-10 2.83E-09 12.22024122 UP PDP1

GCAT -1.273661952 4.968030256 -6.596061224 4.33E-10 2.84E-09 11.97123837DOWN GCAT

RP11_881M11.2 -1.309291191 -2.207569372 -6.594165737 4.38E-10 2.86E-09 12.54498831 DOWN RP11-881M11.2

SOWAHD 1.207717932 -1.483822354 6.593726561 4.39E-10 2.87E-09 12.49485532 UP SOWAHD

AP001626.1 2.3608573 -1.733736234 6.590755799 4.46E-10 2.92E-09 12.47630825 UP AP001626.1

THRB_AS1 -1.135106272 -1.104504644 -6.589406248 4.49E-10 2.94E-09 12.4756612 DOWN THRB-AS1

AKR1C4 -2.365917957 6.582126293 -6.586273849 4.57E-10 2.99E-09 11.88685651DOWN AKR1C4

RAB34 1.712898539 3.221028024 6.583520313 4.64E-10 3.03E-09 12.03589105 UP RAB34

MT_ND4L -1.306173617 8.789297762 -6.583149634 4.65E-10 3.03E-09 11.88554369DOWN MT-ND4L

RUNDC3A_AS1 1.350016379 -4.43428046 6.582385888 4.67E-10 3.04E-09 12.50017061 UP RUNDC3A-AS1

BCAS1 2.458493407 -0.795115502 6.581491475 4.69E-10 3.06E-09 12.37962754 UP BCAS1

LRRIQ1 1.812662867 -4.903267962 6.580734419 4.71E-10 3.07E-09 12.49126964 UP LRRIQ1

GCNT3 2.634657283 0.459988923 6.577000148 4.81E-10 3.13E-09 12.26371876 UP GCNT3

ESRP1 3.451481681 -1.96428088 6.576663292 4.81E-10 3.13E-09 12.39334489 UP ESRP1

LINC00961 -1.008126401 -0.577525969 -6.575179009 4.85E-10 3.16E-09 12.37183695 DOWN LINC00961

TPBG 1.877856865 0.233710805 6.5749011824.86E-10 3.16E-09

12.28095389 UP TPBG
CACNA1G 1.823296854 -4.317628652 6.570526684 4.98E-10 3.23E-09 12.43526172 UP CACNA1G
LAMB1 1.176599331 5.887696639 6.569796671 5.00E-10 3.24E-09 11.79875009UP LAMB1
IGFBP2 -2.020657597 7.419651055 -6.569753371 5.00E-10 3.24E-09 11.79468355DOWN IGFBP2
AC114812.5 -1.557505288 -4.696141028 -6.568835837 5.02E-10 3.26E-09 12.42981055 DOWN AC114812.5
ISL2 2.136602935 -3.272081359 6.567440296 5.06E-10 3.28E-09 12.39920338 UP ISL2
RP11_259N19.1 1.710458547 -2.605175486 6.566619302 5.09E-10 3.30E-09 12.38438778 UP RP11-259N19.1
TMEM252 -2.621648767 -3.542003442 -6.566511103 5.09E-10 3.30E-09 12.41440741 DOWN TMEM252
RP11_46F15.2 1.110708044-2.97000841 6.564382764 5.15E-10 3.33E-09 12.388899 UP RP11-46F15.2
TRIM71 3.86236086 -1.472372257 6.563369066 5.18E-10 3.35E-09 12.29320068 UP TRIM71
RP11_799B12.1 -1.323901659 2.221093123 -6.562247623 5.21E-10 3.37E-09 12.07892755 DOWN RP11-799B12.1
RP11_71E19.2 -1.703391537 -2.996486612 -6.561709532 5.22E-10 3.38E-09 12.38710615 DOWN RP11-71E19.2
MEP1B -2.243504504 -3.675816808 -6.560260044 5.26E-10 3.40E-09 12.38316229 DOWN MEP1B
ARHGAP40 2.279664806 -3.917436665 6.558555898 5.31E-10 3.43E-09 12.36289118UP ARHGAP40
CTA_390C10.10 1.315119625-1.713737931 6.556745857 5.37E-10 3.46E-09 12.30841679 UP CTA-390C10.10
C6orf163 1.131329323 -2.46103008 6.555246144 5.41E-10 3.49E-09 12.32813259 UP C6orf163
AC093375.1 1.962978263 -3.049473134 6.5511133185.53E-10 3.57E-09 12.31029323 UP AC093375.1

GNB3 1.406417825 -3.26781284 6.551019494 5.54E-10 3.57E-09 12.32126801 UP GNB3
HSD17B13 -4.580754171 3.132382302 -6.54993656 5.57E-10 3.59E-09 12.03327766 DOWN HSD17B13
KCNQ1OT1 1.189527386 0.71245796 6.549820735 5.57E-10 3.59E-09 12.1163609 UP KCNQ1OT1
HEPACAM -4.085743547 -1.510653102 -6.547444326 5.64E-10 3.63E-09 12.27722914 DOWN HEPACAM
MEP1A 3.265269213 -1.909871109 6.547181894 5.65E-10 3.64E-09 12.23639642 UP MEP1A
CTD_2619J13.8 -1.738649314 2.058425559 -6.54610216 5.68E-10 3.66E-09 12.0191302 DOWN CTD-2619J13.8
PLCD3 1.358566558 2.178429963 6.544136733 5.75E-10 3.70E-09 11.9362211 UP PLCD3
RP5_881L22.5 -2.003656432 -2.408931588 -6.542258611 5.80E-10 3.73E-09 12.27525062 DOWN RP5-881L22.5
CNDP1 -3.624635547 -0.11575206 -6.541639476 5.82E-10 3.74E-09 12.19915974 DOWN CNDP1
CCDC38 -1.528211868 -0.810335113 -6.53656899 5.99E-10 3.85E-09 12.18591602 DOWN CCDC38
SLC25A30_AS1 -1.263462703 -2.028913321 -6.535516277 6.02E-10 3.87E-09 12.23001914 DOWN SLC25A30-AS1
NRG3 2.324543368 -2.84480731 6.533806482 6.08E-10 3.90E-09 12.2094252 UP NRG3
RP11_651L5.3 1.074528892 -1.705183634 6.533449575 6.09E-10 3.91E-09 12.18731296 UP RP11-651L5.3
SORCS1 2.286673354 -4.365788466 6.531355527 6.16E-10 3.94E-09 12.22649091 UP SORCS1
RP11_498D10.8 -1.540180161 -4.454748961 -6.530897982 6.17E-10 3.95E-09 12.23037243 DOWN RP11-498D10.8
CSPG4P12 1.239890744 -2.939410253 6.52458801 6.39E-10 4.08E-09 12.1774656 UP CSPG4P12
PLA2G12B -2.14745292 4.535839625 -6.521803821 6.49E-10 4.14E-09

11.6276989 DOWN PLA2G12B

MUC1 2.227592258 0.435020499 6.513951814 6.77E-10 4.31E-09

11.93688246UP MUC1

STRA6 2.270022306 -2.421912654 6.512156552 6.83E-10 4.35E-09

12.08474437 UP STRA6

ZNF286B 1.08384363 -1.739741846 6.512034133 6.84E-10 4.36E-09

12.07602207 UP ZNF286B

SNORA59B -1.784399872 -3.141810321 -6.509976258 6.91E-10 4.40E-09 12.11642632DOWN SNORA59B

SLC2A1 1.514581503 2.535835279 6.50917352 6.94E-10 4.42E-09

11.71017772UP SLC2A1

KRT80 2.716227674 -0.610819247 6.508850202 6.96E-10 4.43E-09

11.98305926UP KRT80

CYP2D7 -2.04621722 2.597555234 -6.508835317 6.96E-10 4.43E-09

11.7723366 DOWN CYP2D7

ANKRD37 -1.312504743 1.660196887 -6.507566929 7.01E-10 4.45E-09 11.84868861DOWN ANKRD37

CD101 1.021192446-0.490132931 6.505079514 7.10E-10 4.51E-09

11.97297292UP CD101

ACSL6 -2.204574524 2.008093736 -6.501713305 7.23E-10 4.59E-09

11.80213467DOWN ACSL6

WNT7B2.876198029 -2.858777257 6.500421374 7.28E-10 4.62E-09

12.0268415 UP WNT7B

ATP6V0D2 2.182606958 -2.015044938 6.49921722 7.33E-10 4.65E-09

12.00540507 UP ATP6V0D2

TMEM72 2.482760675 -3.09844201 6.498521373 7.36E-10 4.67E-09

12.02877646 UP TMEM72

PRRX2 1.979704707 -3.519341674 6.497633574 7.39E-10 4.69E-09

12.03882098 UP PRRX2

ALB -2.175343208 14.71131401-6.497524065 7.40E-10 4.69E-09

11.59041036DOWN ALB

KRT17P4 -2.037836647 -4.278496421 -6.49689317 7.42E-10 4.70E-09

12.05170559 DOWN KRT17P4

RP11_674N23.4 1.292955845 -5.528390755 6.494351095 7.52E-10 4.77E-09 12.03743846 UP RP11-674N23.4

ANKLE1 1.305393092 -1.973541574 6.489609842 7.72E-10 4.88E-09 11.96514677UP ANKLE1

ZNF528_AS1 1.240236167 -0.082598023 6.488489652 7.77E-10 4.91E-09 11.85532902UP ZNF528-AS1

TCP11 -1.558148145 -3.870417661 -6.486074366 7.87E-10 4.97E-09 11.99514591DOWN TCP11

ST3GAL4_AS1 1.04548129 0.27136359 6.486036952 7.87E-10 4.97E-09 11.81815216UP ST3GAL4-AS1

ACSM3 -1.809740695 3.789565698 -6.48104845 8.08E-10 5.10E-09 11.47789371DOWN ACSM3

SLC16A13 -1.213288885 4.211559454-6.480646605 8.10E-10 5.10E-09 11.41006229DOWN SLC16A13

METTL7B -1.235050435 6.673856438 -6.478961971 8.18E-10 5.15E-09 11.30811652DOWN METTL7B

GTF2IRD1P1 -1.367621544 -3.488593444 -6.477250788 8.25E-10 5.20E-09 11.94813151DOWN GTF2IRD1P1

VSTM5 1.38512513 -3.655185543 6.473168475 8.44E-10 5.30E-09 11.91993235UP VSTM5

RXFP4 1.502583747 -5.274330509 6.471389954 8.52E-10 5.35E-09 11.91753377UP RXFP4

KSR2 1.886499381 -3.297516748 6.470799797 8.54E-10 5.37E-09 11.89524006UP KSR2

RPLP0P2 1.602577873 -1.995716199 6.469101855 8.62E-10 5.41E-09 11.85488719UP RPLP0P2

LINC00152 1.2112163033.174594131 6.469059009 8.62E-10 5.41E-09 11.42850096UP LINC00152

RP11_675F6.4 1.301647128 -5.78111263 6.468908958 8.63E-10 5.42E-09 11.90400247UP RP11-675F6.4

CDS1 2.305103427 0.336283408 6.468614478 8.65E-10 5.42E-09 11.70531858UP CDS1

MBOAT2 1.566964816 0.568909929 6.468599525 8.65E-10 5.42E-

09 11.69532676UP MBOAT2
RHPN1 1.144575568 2.342308288 6.4684135 8.65E-10 5.43E-09
11.51841043UP RHPN1
RNF39 1.318512288 -1.531305743 6.467928866 8.68E-10 5.44E-09
11.83315065UP RNF39
RP11_803D5.1 1.295244781 -4.10802068 6.466587705 8.74E-10 5.48E-09 11.89095188UP RP11-803D5.1
RP11_314C16.1 -1.466765101 -2.957194769 -6.465592801 8.79E-10
5.50E-09 11.88313384DOWN RP11-314C16.1
HAGLR2.490667856 0.379435107 6.463951527 8.87E-10 5.54E-09
11.67346967UP HAGLR
SAA2 -4.122064413 4.544918067 -6.463458066 8.89E-10 5.56E-09
11.42455041DOWN SAA2
RP11_267D19.2 -1.322234314 -4.985782034 -6.462815367 8.92E-10
5.57E-09 11.8736013 DOWN RP11-267D19.2
SYT9 -3.047793231 -3.078586788 -6.462248334 8.95E-10 5.59E-09
11.86099331DOWN SYT9
NMB 1.205697064 1.470224178 6.462132975 8.95E-10 5.59E-09
11.5800509 UP NMB
RP11_521M14.1 -2.73109009 -1.447470917 -6.458820419 9.11E-10 5.69E-09 11.81374718DOWN RP11-521M14.1
CDH10 2.346579224 -5.165392358 6.455444835 9.28E-10 5.79E-09
11.8330284 UP CDH10
COL25A1 -1.838611983 -3.024204095 -6.454845416 9.31E-10 5.81E-09 11.82685217DOWN COL25A1
SAPCD1 1.316761127 -1.9651213 6.453448034 9.38E-10 5.85E-09
11.77528621UP SAPCD1
FCHSD1 1.058484563 1.545583937 6.453230872 9.39E-10 5.85E-09 11.52831579UP FCHSD1
HSD3B2 -1.668214094 -4.102932714 -6.451918073 9.46E-10 5.89E-09 11.81700365DOWN HSD3B2
FMN1 1.548919573 -0.106616629 6.450838507 9.51E-10 5.92E-09
11.65608928UP FMN1

COLCA1 1.762313484 0.274823804 6.450503682 9.53E-10 5.93E-09 11.62131654UP COLCA1
AGPAT4 1.27287769 1.4118367766.450040954 9.56E-10 5.95E-09 11.52247414UP AGPAT4
ZNF486 1.504888583 -0.533733142 6.449269694 9.60E-10 5.97E-09 11.67760707UP ZNF486
SHF -1.245400639 3.779734599 -6.449071041 9.61E-10 5.97E-09 11.28656893DOWN SHF
PDE3B -1.15166418 4.270774824 -6.445195014 9.81E-10 6.09E-09 11.21676563DOWN PDE3B
SLC22A18 -1.395036819 6.065408132 -6.444138138 9.86E-10 6.13E-09 11.13026432DOWN SLC22A18
AC018647.3 -1.327870146 -2.311913072 -6.443353096 9.91E-10 6.15E-09 11.75582915DOWN AC018647.3
MLLT3 1.416743615 1.34112838 6.442182167 9.97E-10 6.19E-09 11.48672621UP MLLT3
3-Sep 1.743781131-0.980309295 6.44203795 9.98E-10 6.19E-09 11.66446872UP 3-Sep
MT_ATP8 -1.212977964 8.330275637 -6.44134105 1.00E-09 6.21E-09 11.12428624DOWN MT-ATP8
ADAMTSL3 -1.749004889 2.812367953 -6.440157233 1.01E-09 6.25E-09 11.37718794DOWN ADAMTSL3
NRCAM 2.682758384 0.401589261 6.440126663 1.01E-09 6.25E-09 11.54457629UP NRCAM
AKAP3 -1.400143205 -1.430138358 -6.438890142 1.01E-09 6.29E-09 11.70160027DOWN AKAP3
RP11_19D2.1 -2.435704717 -0.931639808 -6.437969983 1.02E-09 6.31E-09 11.68294345DOWN RP11-19D2.1
GLIPR1L2 -1.352304762 -1.639135927 -6.437720507 1.02E-09 6.32E-09 11.70399505DOWN GLIPR1L2
FA2H 2.577347075 -2.75253323 6.436395343 1.03E-09 6.36E-09 11.69301407UP FA2H
TNMD -1.56224387 -5.010122933 -6.435956015 1.03E-09 6.38E-09

11.73335898DOWN TNMD
CASC8 1.814147825 -4.499873177 6.434844178 1.04E-09 6.41E-09
11.72562083UP CASC8
GOT2P2 -2.119987543 -2.884901846 -6.433039334 1.05E-09 6.47E-09 11.71054861DOWN GOT2P2
PKDCC 1.299582583 3.596190662 6.429462293 1.07E-09 6.59E-09
11.17791814UP PKDCC
OR7E47P -1.863240425 -3.724803291 -6.427058784 1.08E-09 6.67E-09 11.68679302DOWN OR7E47P
ZNF860 1.614898917 -3.911079427 6.425751774 1.09E-09 6.72E-09
11.67441952UP ZNF860
ATP2C2 2.158089335 -3.239533334 6.425140616 1.09E-09 6.74E-09
11.65211733UP ATP2C2
HK2 1.968439923 1.849292469 6.424599305 1.10E-09 6.75E-09
11.33496848UP HK2
WBP5 1.239438239 3.760890391 6.423349011 1.10E-09 6.79E-09
11.12921754UP WBP5
PLA2G4F 2.07868758 -4.321764752 6.42247149 1.11E-09 6.82E-09
11.65850507UP PLA2G4F
CNTNAP5 1.507007203 -5.537763688 6.422318615 1.11E-09 6.83E-09 11.66153003UP CNTNAP5
KCND3 -2.7427961 2.126948002 -6.421773699 1.11E-09 6.85E-09
11.38451859DOWN KCND3
LHX4 1.292395052 -1.249459822 6.421689556 1.11E-09 6.85E-09
11.57725375UP LHX4
RP11_128A17.2 1.44601407 -3.816972828 6.421620862 1.11E-09 6.85E-09 11.6528265 UP RP11-128A17.2
RP11_119F7.5 1.218998323 -0.784128835 6.421526958 1.11E-09
6.85E-09 11.55095222UP RP11-119F7.5
TNNI3 2.139388032 -4.393871528 6.420982851 1.12E-09 6.87E-09
11.65095125UP TNNI3
SPX -1.996101682 0.509545493 -6.420338001 1.12E-09 6.89E-09
11.50124747DOWN SPX

RP11_344P13.4	-1.625390581	-0.679769926	-6.419294434	1.13E-09	6.93E-09	11.56485978	DOWN	RP11-344P13.4
CH507_9B2.3	-1.412667402	0.789012413	-6.418048115	1.13E-09	6.97E-09	11.45762953	DOWN	CH507-9B2.3
CFHR1	-2.843461962	7.139473157	-6.41787483	1.14E-09	6.98E-09	10.99449857	DOWN	CFHR1
RP11_550P17.5	-1.595298874	-4.908494553	-6.417312287	1.14E-09	6.99E-09	11.63655815	DOWN	RP11-550P17.5
CYP4F12	-1.803062423	4.13625518	-6.415741643	1.15E-09	7.05E-09	11.093229	DOWN	CYP4F12
PRR7	1.186211281	0.860243902	6.415106698	1.15E-09	7.07E-09	11.39327922	UP	PRR7
RBL2	-1.002749258	4.959544962	-6.410430215	1.18E-09	7.24E-09	10.9839079	DOWN	RBL2
RP11_499E18.1	-1.243935949	-0.629478058	-6.408879124	1.19E-09	7.30E-09	11.50339884	DOWN	RP11-499E18.1
LINC01348	-2.579109255	2.172602749	-6.408794212	1.19E-09	7.30E-09	11.30669389	DOWN	LINC01348
MST1P2	-1.69884121	3.623922077	-6.408182714	1.20E-09	7.32E-09	11.10597921	DOWN	MST1P2
C9orf173_AS1	-1.565783637	-0.427971257	-6.405736052	1.21E-09	7.41E-09	11.47932176	DOWN	C9orf173-AS1
GRIN1	1.604248986	-4.339536435	6.405026121	1.22E-09	7.43E-09	11.5706852	UP	GRIN1
SH2D3A	1.48097782	1.225228025	6.404851729	1.22E-09	7.44E-09	11.300984	UP	SH2D3A
PHLDA2	1.628202682	1.738649894	6.401139178	1.24E-09	7.58E-09	11.22607982	UP	PHLDA2
RP11_545A16.3	1.552583084	-5.046902189	6.400743533	1.25E-09	7.60E-09	11.54995334	UP	RP11-545A16.3
RP4_734G22.3	1.081056271	-1.835481213	6.400343842	1.25E-09	7.61E-09	11.49608087	UP	RP4-734G22.3
SHOX2	1.765197587	-2.976422625	6.399042972	1.26E-09	7.66E-09			

11.51579657UP SHOX2
SLC17A3 -1.933251858 2.324415018 -6.398644254 1.26E-09 7.68E-09 11.22026544DOWN SLC17A3
AC005077.12 -1.829846826 -2.037456275 -6.398202197 1.26E-09 7.69E-09 11.51406471DOWN AC005077.12
IL1RAPL2 -1.993613986 -2.832668995 -6.395634453 1.28E-09 7.79E-09 11.5159348 DOWN IL1RAPL2
KIAA1024 1.651260018 -2.665881242 6.39515383 1.28E-09 7.81E-09 11.48935565UP KIAA1024
CTA_292E10.6 -1.017253365 -0.185008934 -6.395137157 1.28E-09 7.81E-09 11.4021785 DOWN CTA-292E10.6
FAM99B -2.877711786 -1.404402837 -6.394480886 1.29E-09 7.83E-09 11.4773236 DOWN FAM99B
SAA2_SAA4 -4.151761338 1.630283077 -6.39342407 1.29E-09 7.87E-09 11.31667023DOWN SAA2-SAA4
ERP27 1.886488353 -0.856247352 6.387146926 1.34E-09 8.13E-09 11.36925386UP ERP27
EPN3 2.066326773 -2.664515921 6.386097361 1.35E-09 8.18E-09 11.43657504UP EPN3
CTD_2350C19.2-1.486939941 0.503228728 -6.385350557 1.35E-09 8.21E-09 11.3105754 DOWN CTD-2350C19.2
APOA1 -2.79989306 11.20805809-6.383684485 1.36E-09 8.28E-09 10.88601326 DOWN APOA1
FABP5 1.188978134 2.149138359 6.383580313 1.36E-09 8.28E-09 11.09424139UP FABP5
C19orf26 1.265127049 -1.927821713 6.383237029 1.37E-09 8.29E-09 11.40882921UP C19orf26
GPR35 1.923798192 0.564833957 6.381558693 1.38E-09 8.36E-09 11.23351903UP GPR35
RP11_524F11.2 -1.410381331 -3.297486089 -6.380201366 1.39E-09 8.42E-09 11.44232318DOWN RP11-524F11.2
LLNLR_268E12.1 1.211712809-3.394869218 6.379897472 1.39E-09 8.43E-09 11.4318185 UP LLNLR-268E12.1

AP000997.2 -1.682881291 -4.829462695 -6.379809276 1.39E-09 8.43E-09 11.4421185 DOWN AP000997.2

C5 -1.24859467 8.482549512 -6.377697025 1.41E-09 8.52E-09 10.79251379 DOWN C5

HEY2 -1.095873602 1.883230959 -6.377372507 1.41E-09 8.53E-09 11.13697974DOWN HEY2

RP11_255M6.1 -1.566393285 -4.715188345 -6.377151426 1.41E-09 8.54E-09 11.42863061DOWN RP11-255M6.1

LINC01191 1.501618047 -4.609480595 6.375265327 1.43E-09 8.62E-09 11.4177895 UP LINC01191

CDIPT_AS1 1.555596087 -3.890874658 6.374487194 1.43E-09 8.65E-09 11.4084448 UP CDIPT-AS1

RAB3D 1.546082344 1.749064366 6.374263138 1.43E-09 8.66E-09 11.08579264UP RAB3D

HCG15 1.417885291 -2.980939298 6.373684388 1.44E-09 8.68E-09 11.38890141UP HCG15

BEST1 1.100228584 0.296986655 6.373282755 1.44E-09 8.70E-09 11.22511408UP BEST1

SLCO1B7 -1.959935559 -4.455083943 -6.373084161 1.44E-09 8.70E-09 11.40744444DOWN SLCO1B7

C4orf48 1.594286564 -0.218310379 6.372470379 1.45E-09 8.73E-09 11.25285996UP C4orf48

HTR3A 2.077524702 -4.475552495 6.371303022 1.46E-09 8.78E-09 11.39447904UP HTR3A

KLF2P1 1.797885085 -5.477535865 6.369322571 1.47E-09 8.87E-09 11.38657748UP KLF2P1

USH1C 3.615270368 0.486842876 6.368821395 1.48E-09 8.89E-09 11.15578285UP USH1C

RP5_857K21.6 -1.205301917 6.43759028 -6.368674327 1.48E-09 8.89E-09 10.72860594 DOWN RP5-857K21.6

GDF11 1.07010848 1.806965505 6.366487074 1.50E-09 8.99E-09 11.04500528UP GDF11

TREM2 1.464627294 1.351467558 6.365855551 1.50E-09 9.02E-09

11.08471229UP TREM2

TBX10 -1.699001095 -1.340223294 -6.363951843 1.52E-09 9.10E-09 11.31025033DOWN TBX10

MYCBPAP 1.227065283 -2.832295249 6.361781545 1.53E-09 9.20E-09 11.32626411UP MYCBPAP

CD177 2.038841489 -3.182730834 6.360188682 1.55E-09 9.26E-09 11.3149803 UP CD177

RP11_1070N10.3 -1.580809925 -2.442574338 -6.359754619 1.55E-09 9.28E-09 11.3248888 DOWN RP11-1070N10.3

FUT2 1.864677236 -0.561128698 6.353738215 1.60E-09 9.57E-09 11.17626484UP FUT2

SLC26A8 -1.320731068 -2.891261216 -6.353377569 1.60E-09 9.58E-09 11.29995723DOWN SLC26A8

RIMS3 1.139616544 -0.025775918 6.353347298 1.60E-09 9.58E-09 11.14593115UP RIMS3

CASKIN1 1.83710166 -3.473096636 6.351803588 1.62E-09 9.65E-09 11.28103204UP CASKIN1

GDAP1 1.124338442 1.579874872 6.349707237 1.64E-09 9.76E-09 10.98161553 UP GDAP1

C15orf48 2.128329418 0.757768661 6.34948567 1.64E-09 9.76E-09 11.04805742UP C15orf48

XXbac_BPG116M5.17 -1.569486963 -3.645744704 -6.348871751 1.64E-09 9.79E-09 11.28184916DOWN XXbac-BPG116M5.17

RP11_244M2.1 2.255585206 -2.867579476 6.34860339 1.65E-09 9.81E-09 11.24526188UP RP11-244M2.1

RP11_53O19.1 1.076449648 -2.09045967 6.34693433 1.66E-09 9.89E-09 11.22909622UP RP11-53O19.1

RP11_766F14.1 -1.645694803 -2.481719726 -6.346779583 1.66E-09 9.89E-09 11.25852704DOWN RP11-766F14.1

SPATA41 -1.652888813 0.364139594 -6.345686985 1.67E-09 9.95E-09 11.11747347DOWN SPATA41

LINC01535 1.903468369 -2.949704163 6.344999525 1.68E-09 9.98E-09 11.23278533UP LINC01535

PEBP4 -2.672634866 -3.212301327 -6.343032864 1.69E-09 1.01E-08 11.24564238DOWN PEBP4
EFCAB6 -1.252259914 -1.16237027 -6.341684478 1.71E-09 1.01E-08 11.18300822DOWN EFCAB6
CDK5R2 1.463510037 -5.619105239 6.341558637 1.71E-09 1.02E-08 11.24332825UP CDK5R2
RP11_333I13.1 1.179103796 -3.435424589 6.335157724 1.77E-09 1.05E-08 11.20166032UP RP11-333I13.1
CH17_13I23.3 1.679754123 -3.883658441 6.335098062 1.77E-09 1.05E-08 11.20371435UP CH17-13I23.3
RP11_170N16.3 -1.272611841 -2.334539685 -6.335021242 1.77E-09 1.05E-08 11.19460659DOWN RP11-170N16.3
SHE -1.116423208 2.770696666 -6.33449233 1.77E-09 1.05E-08 10.81051564 DOWN SHE
CCDC64B 2.63561905 -2.77804333 6.333620998 1.78E-09 1.06E-08 11.15924627UP CCDC64B
EPHX4 1.433852392 -2.638738682 6.330817304 1.81E-09 1.07E-08 11.15860289UP EPHX4
ROPN1B -1.882927908 -2.692632158 -6.3303508 1.81E-09 1.07E-08 11.17680546DOWN ROPN1B
RP11_278H7.3 -2.062228709 -4.970819704 -6.330180562 1.81E-09 1.08E-08 11.18517334DOWN RP11-278H7.3
RP1_120G22.11 -1.326295836 -2.080557161 -6.329543874 1.82E-09 1.08E-08 11.1589746 DOWN RP1-120G22.11
FOXN1 1.484127588 -5.310112499 6.325523101 1.86E-09 1.10E-08 11.16112478UP FOXN1
IAPP -1.747312774 -3.35847971 -6.324888945 1.87E-09 1.10E-08 11.15657751DOWN IAPP
KCNE2 -1.227558763 -1.721683563 -6.319461607 1.92E-09 1.14E-08 11.09320379DOWN KCNE2
GFRA3 2.062490472 -4.43815812 6.319378367 1.92E-09 1.14E-08 11.12639834UP GFRA3
KCNK9 2.514346111 -3.659428966 6.318095115 1.94E-09 1.14E-08

11.10242496UP KCNK9

RP11_15I11.3 -2.4573919 -0.389661159 -6.317435295 1.94E-09 1.15E-08 11.0301485 DOWN RP11-15I11.3

RAP1GAP2 1.617856782 1.384092284 6.316878076 1.95E-09 1.15E-08 10.82409159 UP RAP1GAP2

GOT2P3 -1.804609279 -3.86293059 -6.31509135 1.97E-09 1.16E-08 11.10817757DOWN GOT2P3

FZD1 1.369398079 2.40707968 6.314785224 1.97E-09 1.16E-08 10.70564465 UP FZD1

TRIM47 1.007844218 4.476719992 6.314006507 1.98E-09 1.17E-08 10.49891757 UP TRIM47

CYP4F23P 1.941994347 -4.093424453 6.31377847 1.98E-09 1.17E-08 11.09471902UP CYP4F23P

ABHD1 -1.915338114 0.567951452 -6.311792409 2.00E-09 1.18E-08 10.93039109 DOWN ABHD1

KB_1125A3.10 -1.519599748 -3.707413498 -6.311346503 2.01E-09 1.18E-08 11.08880908DOWN KB-1125A3.10

BCRP3 1.525840567 -1.594176515 6.310763466 2.01E-09 1.18E-08 11.01628757UP BCRP3

RP11_353N14.4 1.660655357 -3.507052151 6.308807778 2.03E-09 1.20E-08 11.06232132UP RP11-353N14.4

RP11_799B12.2 -1.380546409 -2.428209318 -6.308498357 2.04E-09 1.20E-08 11.06041302DOWN RP11-799B12.2

HGFAC -3.865945286 3.857575757 -6.307475576 2.05E-09 1.20E-08 10.65629517 DOWN HGFAC

RP11_187E13.1 1.681367213 -4.311702319 6.305573723 2.07E-09 1.21E-08 11.05657021UP RP11-187E13.1

HUNK 2.415961622 0.280675551 6.304782022 2.08E-09 1.22E-08 10.85374868 UP HUNK

LINC01094 1.404990458 -1.981571726 6.303896185 2.09E-09 1.22E-08 10.9995592 UP LINC01094

PROL1 -3.147693836 -2.685479142 -6.301183938 2.12E-09 1.24E-08 11.02025206DOWN PROL1

FGFR3 1.304869142 5.61514217 6.3011011922.12E-09 1.24E-08 10.38852383 UP FGFR3
RP11_1399P15.11.707217363 -3.972255146 6.299193743 2.14E-09 1.25E-08 11.02008624UP RP11-1399P15.1
CECR7 2.498012612 -3.626504383 6.296925516 2.17E-09 1.27E-08 10.99340108 UP CECR7
TSLP -2.194440954 -0.656137519 -6.295653764 2.18E-09 1.28E-08 10.92931344 DOWN TSLP
AP001505.10 1.033978743 0.159189855 6.29182878 2.23E-09 1.30E-08 10.81416132 UP AP001505.10
AACSP1 1.809780993 -5.222666395 6.290037506 2.25E-09 1.31E-08 10.97844885 UP AACSP1
DUSP9 2.54246999 2.490409017 6.287390636 2.28E-09 1.33E-08 10.55704154 UP DUSP9
LINC01151 -2.531250291 -1.252916658 -6.287048891 2.28E-09 1.33E-08 10.91575279 DOWN LINC01151
SGK494 1.048094126 -0.511419577 6.282313134 2.34E-09 1.36E-08 10.81365925 UP SGK494
DUOX1 1.386954305 0.815593909 6.278682495 2.39E-09 1.39E-08 10.6860141 UP DUOX1
SPP1 3.17437009 6.28276374 6.278401748 2.39E-09 1.39E-08 10.2851614 UP SPP1
FAM178B 2.315941478 -3.799879255 6.278315556 2.39E-09 1.39E-08 10.90389601 UP FAM178B
LINGO1 1.628994876 1.754466185 6.277234534 2.40E-09 1.40E-08 10.57953411UP LINGO1
AP000472.2 -2.061965783 -2.710211026 -6.276883628 2.41E-09 1.40E-08 10.90167309 DOWN AP000472.2
STARD6 1.229198879 -5.45926478 6.27672127 2.41E-09 1.40E-08 10.91054407 UP STARD6
RP5_968J1.1 1.229590624 -5.122389839 6.276567553 2.41E-09 1.40E-08 10.91020622 UP RP5-968J1.1
AC006369.2 -1.488230254 -3.838605607 -6.27637001 2.42E-09 1.40E-08

10.90964462 DOWN AC006369.2
RP11_37N22.1 1.4162033 -5.069363422 6.274669607 2.44E-09 1.41E-08 10.90031044 UP RP11-37N22.1
SLC10A6 -1.435464879 -3.466225876 -6.273926207 2.45E-09 1.42E-08 10.89602966 DOWN SLC10A6
UGT8 2.258596716 -3.637367671 6.273462518 2.45E-09 1.42E-08 10.87643067 UP UGT8
BIRC7 1.989936744 -3.253729608 6.273357202 2.45E-09 1.42E-08 10.87011202UP BIRC7
SLCO4A1_AS1 2.014899574 -4.617998752 6.273041774 2.46E-09 1.42E-08 10.88992217 UP SLCO4A1-AS1
ITPKA 1.632786229 1.748187739 6.271512107 2.48E-09 1.44E-08 10.5507994 UP ITPKA
DLX5 2.069756428 -3.76417684 6.269490327 2.50E-09 1.45E-08 10.86058601 UP DLX5
RP3_402G11.26 -1.030870414 -0.518833955 -6.269450264 2.51E-09 1.45E-08 10.77221251 DOWN RP3-402G11.26
COL11A1 2.781507862 -1.899573295 6.269006451 2.51E-09 1.45E-08 10.7984651 UP COL11A1
GAS6_AS1 -1.618355968 1.097895285 -6.268300301 2.52E-09 1.46E-08 10.65971845 DOWN GAS6-AS1
ADH7 -1.900335535 -3.680439049 -6.266423292 2.55E-09 1.47E-08 10.85792023 DOWN ADH7
RP11_848P1.9 1.161793684 -1.453868509 6.26493351 2.57E-09 1.48E-08 10.77826179 UP RP11-848P1.9
TREM1 1.788487285 -1.659313846 6.263413385 2.59E-09 1.49E-08 10.77320911UP TREM1
C3orf36 -1.658128207 -3.202389767 -6.263310688 2.59E-09 1.49E-08 10.83956591 DOWN C3orf36
CEACAM1 -1.17492587 5.588532144 -6.261450274 2.61E-09 1.51E-08 10.18381216 DOWN CEACAM1
CYP26A1 -2.75452386 -1.166802572 -6.259388437 2.64E-09 1.52E-08 10.77143721 DOWN CYP26A1

AP000240.9 1.181081966 -2.497296415 6.258814939 2.65E-09 1.53E-08 10.78743431 UP AP000240.9

ENPP1 -1.074243632 5.567137464 -6.256085951 2.69E-09 1.55E-08 10.15568239 DOWN ENPP1

KIAA1324 1.533279194 -0.416445213 6.251235694 2.76E-09 1.59E-08 10.64101386 UP KIAA1324

PRRT2 1.067021968 -1.388461107 6.251083287 2.76E-09 1.59E-08 10.70475952 UP PRRT2

CD97 1.074086982 4.674335943 6.248481778 2.80E-09 1.61E-08 10.14804897 UP CD97

ADAMTS141.457961274 -0.629527594 6.247779557 2.81E-09 1.62E-08 10.6391556 UP ADAMTS14

COL5A3 -1.483717688 4.976000335 -6.245260594 2.85E-09 1.64E-08 10.13148766 DOWN COL5A3

POU2AF1 1.892987452 -0.426953302 6.243664948 2.87E-09 1.65E-08 10.59834996 UP POU2AF1

RP11_505K9.1 -1.20692795 -0.009305225 -6.242456792 2.89E-09 1.66E-08 10.60341981 DOWN RP11-505K9.1

RP11_219B17.3 -1.478867242 -2.713387984 -6.241206963 2.91E-09 1.67E-08 10.72103165 DOWN RP11-219B17.3

RP11_872D17.8 -1.940140757 -1.168300937 -6.241113263 2.91E-09 1.67E-08 10.67228309 DOWN RP11-872D17.8

RNA5SP334-1.35614116 -5.873621366 -6.238363104 2.95E-09 1.69E-08 10.71269384 DOWN RNA5SP334

RP5_1009E24.8 1.451881176-3.679444438 6.237754198 2.96E-09 1.70E-08 10.70351436 UP RP5-1009E24.8

PRAMENP -2.156120444 -3.590706794 -6.237086226 2.97E-09 1.70E-08 10.7071354 DOWN PRAMENP

FGD3 1.074166608 1.97734814 6.236381007 2.98E-09 1.71E-08 10.35138202 UP FGD3

MAP7 -1.076175681 4.089884862 -6.236274368 2.99E-09 1.71E-08 10.14291025 DOWN MAP7

CTC_498J12.1 -1.605923191 -2.220531162 -6.234560476 3.01E-09

1.72E-08	10.67638027	DOWN CTC-498J12.1

MAFA_AS1 1.981014116-4.536331744	6.234479528	3.01E-09	1.72E-08	10.69255662	UP MAFA-AS1

TNFSF9	1.745908352	-1.586063751	6.232967696	3.04E-09	1.74E-08	10.61409706	UP TNFSF9

NCAPD2P1 2.314658375	-3.706625284	6.232021437	3.05E-09	1.74E-08	10.66471609	UP NCAPD2P1

CTB_96E2.2	-1.560032276	-3.521683629	-6.231685742	3.06E-09	1.75E-08	10.68028438	DOWN CTB-96E2.2

CES1P2 -1.653555691	-3.668239456	-6.229049783	3.10E-09	1.77E-08	10.66730107	DOWN CES1P2

KCNG1 2.180136789	-2.557406654	6.227127052	3.13E-09	1.79E-08	10.61314015	UP KCNG1

RP11_626G11.4 1.233327583	-4.356021308	6.22708559 3.13E-09	1.79E-08	10.65681543	UP RP11-626G11.4

TTC34	1.528063938	-2.898890427	6.225473356	3.16E-09	1.80E-08	10.62320317	UP TTC34

ERRFI1 -1.180614267	7.494253504	-6.225041316	3.17E-09	1.80E-08	9.984579687	DOWN ERRFI1

PROX1 -1.150117972	6.737543077	-6.223564425	3.19E-09	1.82E-08	9.973347005	DOWN PROX1

AP000344.4 1.626799647	-4.81451695 6.223054153	3.20E-09	1.82E-08	10.6363337 UP AP000344.4

DPY19L2P2 1.269548557	-2.175791346	6.222540593	3.21E-09	1.83E-08	10.59129036	UP DPY19L2P2

KCNQ4 1.364533578	-1.361825068	6.222309753	3.21E-09	1.83E-08	10.55266389	UP KCNQ4

HNRNPA1P66	-1.703077101	-4.488229365	-6.215586414	3.33E-09	1.89E-08	10.59949461	DOWN HNRNPA1P66

SCRN1	1.490979905	2.61763408 6.21289889 3.38E-09	1.92E-08	10.1546579 UP SCRN1

CTD_2555C10.3 1.884922228	-4.475338334	6.2114976553.40E-09	1.93E-08	10.57553951	UP CTD-2555C10.3

SOX9 1.925228341 4.05229525 6.2101737 3.43E-09 1.94E-08 10.00642998 UP SOX9

RP11_1151B14.2 -1.56479618 -0.088114837 -6.208770863 3.45E-09 1.96E-08 10.44138057 DOWN RP11-1151B14.2

NLRP14 -1.631678637 -3.819094175 -6.208483993 3.46E-09 1.96E-08 10.56300538 DOWN NLRP14

FIBCD1 2.220962092 -3.800775702 6.208249643 3.46E-09 1.96E-08 10.54687231 UP FIBCD1

AXDND1 1.440532685 -3.487444969 6.20488252 3.52E-09 1.99E-08 10.53279276 UP AXDND1

AC006946.16 1.745979888 -5.004835097 6.202839867 3.56E-09 2.01E-08 10.53331771 UP AC006946.16

PARK2 -1.183194356 1.398816356 -6.201587517 3.58E-09 2.02E-08 10.27889109 DOWN PARK2

SLC4A4 -1.773755352 3.990301489 -6.199388956 3.63E-09 2.05E-08 9.990786508 DOWN SLC4A4

NSUN7 2.281724861 -2.413614061 6.1950478 3.71E-09 2.09E-08 10.4440242 UP NSUN7

KLHL35 1.493607973 -2.878031798 6.193093529 3.75E-09 2.11E-08 10.45793263 UP KLHL35

OGFR_AS1 -1.130141851 -2.062776038 -6.19229011 3.76E-09 2.12E-08 10.4545884 DOWN OGFR-AS1

FFAR2 1.675893226 -2.154439503 6.189794886 3.81E-09 2.15E-08 10.41752989 UP FFAR2

AC027612.6 1.44214277 -0.61708836 6.188926472 3.83E-09 2.16E-08 10.33699698 UP AC027612.6

AC111186.1 -1.665906725 -0.351501493 -6.184673248 3.92E-09 2.20E-08 10.33597741 DOWN AC111186.1

CTB_113P19.5 -1.194486445 -0.841885446 -6.184182857 3.93E-09 2.21E-08 10.35674429 DOWN CTB-113P19.5

RP11_77K12.9 -1.199247126 -1.148880199 -6.182694423 3.96E-09 2.23E-08 10.36610136 DOWN RP11-77K12.9

BICC1 2.237269484 2.926714215 6.182679583 3.96E-09 2.23E-08

9.972174257 UP BICC1

LARP6 1.662888422 1.103034547 6.182209138 3.97E-09 2.23E-08 10.15998306 UP LARP6

CTD_2368P22.1 1.015973998 0.1892117466.178481752 4.05E-09 2.27E-08 10.23036403 UP CTD-2368P22.1

RP11_285J16.1 1.15419717 -3.501769095 6.175974719 4.10E-09 2.30E-08 10.38913664 UP RP11-285J16.1

A1BG_AS1 -1.204443735 0.290075701 -6.175730726 4.10E-09 2.30E-08 10.24116061DOWN A1BG-AS1

DMD -1.149396005 4.426808674 -6.175228048 4.12E-09 2.31E-08 9.801113623DOWN DMD

IER3 1.413802712 4.201451629 6.175106218 4.12E-09 2.31E-08 9.805503776 UP IER3

AHSG -2.642808911 10.07147938 -6.171536049 4.20E-09 2.35E-08 9.755920646 DOWN AHSG

CH17_437K3.1 -1.590931205 -1.754010057 -6.169870618 4.23E-09 2.37E-08 10.33170008 DOWN CH17-437K3.1

GAPDHP74 -1.607933073 -4.691010606 -6.168080779 4.27E-09 2.39E-08 10.35820308 DOWN GAPDHP74

ADAMTS6 1.351949405 -1.262299629 6.167839258 4.28E-09 2.40E-08 10.27041691 UP ADAMTS6

HIST1H1E 1.218153445 -2.920868909 6.165435 4.33E-09 2.42E-08 10.32266736 UP HIST1H1E

C11orf45 1.259769135 -0.788816125 6.165397971 4.33E-09 2.42E-08 10.23064676 UP C11orf45

TRIM60P18 1.136236081 -1.513878384 6.161864995 4.41E-09 2.47E-08 10.25515914 UP TRIM60P18

CA4 -2.439640615 -1.493355012 -6.161334423 4.43E-09 2.47E-08 10.2823874 DOWN CA4

GPRIN2 2.466461578 -4.066726724 6.1611921044.43E-09 2.47E-08 10.31063506 UP GPRIN2

GRID2IP 1.452105683 -3.800785601 6.1603951144.45E-09 2.48E-08 10.31224271 UP GRID2IP

ASIC1 1.940767227 -0.104597766 6.159708368 4.46E-09 2.49E-08 10.14500309 UP ASIC1

CDC42BPG 1.750632362 0.947215243 6.159504319 4.47E-09 2.49E-08 10.05664626 UP CDC42BPG

RP13_735L24.1 1.372803583 -2.668497524 6.159452157 4.47E-09 2.49E-08 10.28311075UP RP13-735L24.1

ITGB4 1.519414304 3.29567975 6.157894833 4.51E-09 2.51E-08 9.800203081 UP ITGB4

CADPS 2.543746058 -2.957446722 6.15631054 4.54E-09 2.53E-08 10.25790814 UP CADPS

EGFL6 1.710692982 -2.761353209 6.151644862 4.66E-09 2.59E-08 10.24221743 UP EGFL6

GOLGA2P10 1.071701034 -0.063874958 6.149306102 4.71E-09 2.62E-08 10.1014258 UP GOLGA2P10

AC135048.13 -1.283362833 -1.44650403 -6.148064917 4.74E-09 2.64E-08 10.20547111DOWN AC135048.13

PFN1P11 -2.488850992 -2.829866632 -6.146722282 4.78E-09 2.66E-08 10.23870595 DOWN PFN1P11

RP11_124N2.1 -1.283035343 -2.805863431 -6.145523064 4.81E-09 2.67E-08 10.23678878 DOWN RP11-124N2.1

MAGIX -1.221132946 3.065623304 -6.144885598 4.82E-09 2.68E-08 9.795750951 DOWN MAGIX

CTC_1337H24.4 1.300705427 -3.265890889 6.143300051 4.86E-09 2.70E-08 10.21736337 UP CTC-1337H24.4

SLC39A4 2.064455745 2.482380996 6.142993018 4.87E-09 2.70E-08 9.811877456UP SLC39A4

DCHS2 1.840683942 -3.878387244 6.142079353 4.90E-09 2.72E-08 10.21734768 UP DCHS2

MRO -1.48540253 0.235687666 -6.14124532 4.92E-09 2.73E-08 10.0745249 DOWN MRO

C16orf96 -1.469802459 -2.616825577 -6.139196235 4.97E-09 2.75E-08 10.20123898 DOWN C16orf96

RP4_665J23.1 -1.034126382 2.821520922 -6.137247957 5.02E-09

2.78E-08 9.782154585 DOWN RP4-665J23.1
CTD_2201I18.1 -1.341159807 -1.412793887 -6.13670274 5.03E-09 2.79E-08 10.1469974 DOWN CTD-2201I18.1
TCTEX1D4 -1.455584386 -3.087063213 -6.136084801 5.05E-09 2.80E-08 10.19231375 DOWN TCTEX1D4
VANGL2 2.058894705 -0.258128725 6.13595431 5.05E-09 2.80E-08 10.0345784 UP VANGL2
OLFML2B 1.15272249 2.69479685 6.134544153 5.09E-09 2.82E-08 9.745649542 UP OLFML2B
LOXL1_AS1 1.7534797 -1.750120364 6.133821303 5.11E-09 2.83E-08 10.11698115UP LOXL1-AS1
APLP1 1.973000789 -0.023255153 6.133223896 5.13E-09 2.84E-08 10.0046972 UP APLP1
PRELID2 1.154880748 0.242746833 6.1311706065.18E-09 2.87E-08 9.983674155 UP PRELID2
GRB7 1.258458939 3.270676044 6.130060746 5.21E-09 2.88E-08 9.658778003 UP GRB7
SERPINE2 1.697520577 3.549547461 6.130052441 5.21E-09 2.88E-08 9.6353651 UP SERPINE2
PGM5_AS1 -1.496818628 -4.92862378 -6.12908401 5.24E-09 2.89E-08 10.16087339 DOWN PGM5-AS1
RP11_290F5.2 -1.292522597 -1.172160026 -6.128795771 5.25E-09 2.90E-08 10.09486972 DOWN RP11-290F5.2
PRSS22 2.724540217 -2.920863231 6.12778091 5.27E-09 2.91E-08 10.11026748UP PRSS22
GLDN 2.315687555 -0.461013405 6.127241554 5.29E-09 2.92E-08 10.00236451 UP GLDN
PPP2R2C 3.130051783 -1.51177769 6.126959612 5.30E-09 2.92E-08 10.05174269 UP PPP2R2C
RP11_118B22.4 -1.609337366 -4.258378633 -6.126862602 5.30E-09 2.92E-08 10.15011552DOWN RP11-118B22.4
RP11_396O20.1 -2.711206143 -3.561871144 -6.126441144 5.31E-09 2.93E-08 10.14395967 DOWN RP11-396O20.1

GALNT16 -1.393732373 0.693859472 -6.124978721 5.35E-09 2.95E-08 9.955312452 DOWN GALNT16
QPRT -1.05492574 7.178742923 -6.124580098 5.36E-09 2.95E-08 9.466603869 DOWN QPRT
MT_RNR1 -1.007775523 10.11639467-6.123259606 5.40E-09 2.97E-08 9.516025467 DOWN MT-RNR1
LIN28B 2.920335336 -4.515304492 6.122951202 5.41E-09 2.98E-08 10.11931976UP LIN28B
OPRD1 1.304922494 -2.565307351 6.122246622 5.43E-09 2.99E-08 10.09284538 UP OPRD1
RP11_328K4.1 -2.918262647 -0.031383053 -6.121482909 5.45E-09 3.00E-08 10.01287211DOWN RP11-328K4.1
KB_1980E6.3 1.293891557 -5.458291873 6.1165988075.59E-09 3.07E-08 10.09705305 UP KB-1980E6.3
EFHD1 -1.714061809 3.146202294 -6.116011855 5.61E-09 3.08E-08 9.657405972 DOWN EFHD1
TATDN2P2 1.333064373 -2.918148673 6.1151151985.63E-09 3.09E-08 10.06700902 UP TATDN2P2
1-Mar -1.215256375 5.038504085 -6.113573439 5.68E-09 3.12E-08 9.445880389 DOWN 1-Mar
P2RX5 1.745428087 -1.543064605 6.1122706055.72E-09 3.14E-08 9.997787907 UP P2RX5
ERO1LB -1.051506109 4.584137655 -6.111728672 5.74E-09 3.14E-08 9.46137348 DOWN ERO1LB
ARHGEF26 -1.400706323 3.816371787 -6.111266372 5.75E-09 3.15E-08 9.540053834 DOWN ARHGEF26
SELP -1.730146594 -0.189804407 -6.110780161 5.76E-09 3.16E-08 9.952110686DOWN SELP
AC003075.4-1.107761775 -0.900870587 -6.10893619 5.82E-09 3.19E-08 9.977633005 DOWN AC003075.4
RP11_173M11.2 -1.097052275 0.553499328 -6.106962246 5.88E-09 3.22E-08 9.870615201 DOWN RP11-173M11.2
XAGE3 -1.62553611 -4.038737614 -6.106350519 5.90E-09 3.22E-08

10.04666232 DOWN XAGE3
LINC01123 1.665572435 -3.430066023 6.104379982 5.96E-09 3.25E-08 10.0203984 UP LINC01123
CA14 -1.77083055 1.776737701 -6.104257604 5.96E-09 3.26E-08 9.756812407 DOWN CA14
CDHR4 1.380540007 -4.454058109 6.10313392 6.00E-09 3.27E-08 10.02914202 UP CDHR4
LINC00884 -1.036042054 1.280966416 -6.103016033 6.00E-09 3.27E-08 9.785365642 DOWN LINC00884
GULP1 2.418298685 -0.493638827 6.101823337 6.04E-09 3.29E-08 9.876003212 UP GULP1
WNT10A 1.635087643 -2.429110026 6.101044912 6.06E-09 3.31E-08 9.977607639 UP WNT10A
PRKX 1.190913278 2.270395736 6.098713109 6.14E-09 3.35E-08 9.612508324 UP PRKX
TFAP2A_AS1 1.956582804 -3.067952275 6.097576328 6.17E-09 3.37E-08 9.973198909 UP TFAP2A-AS1
BECN1P1 1.198359706 -5.590869547 6.095856668 6.23E-09 3.39E-08 9.992522201 UP BECN1P1
RP11_520H14.7 1.2997414 -2.887362559 6.0932230116.32E-09 3.44E-08 9.956267595 UP RP11-520H14.7
EP300_AS1 -1.547149516 -1.950512515 -6.091704236 6.37E-09 3.46E-08 9.943682097 DOWN EP300-AS1
RP11_4B16.4 1.000330625 -2.621264807 6.089321877 6.44E-09 3.50E-08 9.93344949 UP RP11-4B16.4
ALOX5 1.438970173 1.798039871 6.088170677 6.48E-09 3.52E-08 9.610041043 UP ALOX5
G0S2 -1.884695349 4.806668932 -6.087773076 6.50E-09 3.53E-08 9.347312343 DOWN G0S2
RAB36 1.705170418 -1.170487337 6.086178495 6.55E-09 3.56E-08 9.847139578 UP RAB36
AFP 3.97002863 3.840331949 6.085879851 6.56E-09 3.56E-08 9.441167308UP AFP

CYP4Z1 -1.605178616 -4.003273925 -6.085749336 6.57E-09 3.56E-08 9.943104873 DOWN CYP4Z1
GOLGA7B 2.190944594 0.12359638 6.081608451 6.71E-09 3.63E-08 9.728353041 UP GOLGA7B
NPM3 1.051664218 3.262667819 6.080963292 6.73E-09 3.65E-08 9.409058792 UP NPM3
RP11_626H12.1 -2.049383356 -3.563786748 -6.080877784 6.73E-09 3.65E-08 9.916793116DOWN RP11-626H12.1
IL1RL1 -1.979614976 -1.53715334 -6.079080626 6.80E-09 3.68E-08 9.866634424 DOWN IL1RL1
SYNPO2 -1.142641967 2.64014299 -6.079031607 6.80E-09 3.68E-08 9.513238695 DOWN SYNPO2
ZNF350_AS1 1.14040566 -4.108762826 6.078757278 6.81E-09 3.68E-08 9.90586877 UP ZNF350-AS1
CP -1.99044793 8.399235143 -6.078328324 6.82E-09 3.69E-08 9.245705831 DOWN CP
CSPG4P8 1.061656232 -0.594628799 6.077962077 6.84E-09 3.69E-08 9.77795186 UP CSPG4P8
RP11_182J1.14 1.556073262 -4.523038852 6.075673819 6.92E-09 3.73E-08 9.890966954 UP RP11-182J1.14
GPR115 1.885975239 -4.923413073 6.075560416 6.92E-09 3.73E-08 9.8903106 UP GPR115
UPK1A 1.881329009 -2.878205911 6.07492673 6.95E-09 3.75E-08 9.854978071 UP UPK1A
CERCAM 1.47673545 2.293473775 6.074375176 6.97E-09 3.76E-08 9.483921842 UP CERCAM
ZFHX2 1.04290188 -0.598775317 6.073268029 7.01E-09 3.78E-08 9.754073393 UP ZFHX2
MT2P1 -2.142883261 -1.460169183 -6.072511584 7.03E-09 3.79E-08 9.831519731 DOWN MT2P1
CTD_2284J15.1 -1.068073797 1.643413533 -6.071983775 7.05E-09 3.80E-08 9.59249823 DOWN CTD-2284J15.1
GJB2 -1.306239736 4.050657647 -6.070730958 7.10E-09 3.82E-08

9.306644841	DOWN GJB2

FER1L62.877206378	-3.338241319	6.0696118727.14E-09	3.84E-08

9.825951781	UP FER1L6

RP11_320N7.2	-3.315056869	-1.988249056	-6.068739123	7.17E-09

3.86E-08	9.826420758	DOWN RP11-320N7.2

TRPM1 -1.448270421	-3.877134291	-6.061844048	7.43E-09	3.99E-08

9.823166511DOWN TRPM1

RP11_301L8.2	-1.803046624	-2.367569379	-6.061782146	7.44E-09

3.99E-08	9.805867635	DOWN RP11-301L8.2

AC114803.3 1.1114208	-5.90121876 6.059906209	7.51E-09	4.03E-08

9.811568687UP AC114803.3

RP11_309L24.4 1.141562724	-1.40716623 6.059609302	7.52E-09	4.03E-08	9.732946857	UP RP11-309L24.4

RP11_1012E15.2	1.670713884	-4.6260933 6.055835525	7.67E-09

4.11E-08	9.791540873	UP RP11-1012E15.2

TNNT2 1.957404823	-2.443272863	6.055367131	7.69E-09	4.12E-08

9.743299851	UP TNNT2

MKRN2OS 1.523555268	-2.594595349	6.055214763	7.69E-09	4.12E-08	9.754296326	UP MKRN2OS

RAB26 -1.425574626	2.910189827	-6.053954343	7.74E-09	4.14E-08

9.360523986	DOWN RAB26

UBXN10_AS1	-1.77820033 -2.965374472	-6.053250746	7.77E-09	4.16E-08	9.773609812	DOWN UBXN10-AS1

FCN3	-2.148149186	1.559986571	-6.053123189	7.78E-09	4.16E-08

9.52972176 DOWN FCN3

CYP4X1	-1.344757329	0.763822844	-6.051880815	7.83E-09	4.18E-08	9.579822874	DOWN CYP4X1

FCAMR	-3.066791421	0.010771692	-6.049935176	7.91E-09	4.22E-08	9.651540128	DOWN FCAMR

SGCE	1.498267647	3.24802469 6.049444355	7.93E-09	4.23E-08

9.253897805	UP SGCE

KCNJ4 -2.430187473	-0.380329466	-6.04764898 8.00E-09	4.27E-08

9.655388693	DOWN KCNJ4

CTC_471F3.5 1.220335523 -1.467729482 6.04555689 8.09E-09 4.31E-08 9.665245003 UP CTC-471F3.5
MIR137HG 1.347609508 -5.774763462 6.044182032 8.14E-09 4.34E-08 9.733082358 UP MIR137HG
ST3GAL1 -1.108408348 6.597702504 -6.043954746 8.15E-09 4.35E-08 9.055994131 DOWN ST3GAL1
RP11_1100L3.8 -1.392300545 -3.461046607 -6.043307371 8.18E-09 4.36E-08 9.729198475 DOWN RP11-1100L3.8
PYCARD 1.246679125 2.920157141 6.042710585 8.21E-09 4.37E-08 9.253675208 UP PYCARD
ZG16B 2.197439365 -1.65843511 6.042490262 8.22E-09 4.38E-08 9.647260348 UP ZG16B
ATP6V0A4 1.316575179 -5.209541279 6.04164951 8.25E-09 4.39E-08 9.721381076 UP ATP6V0A4
CTD_3162L10.1 1.469115726-3.554245415 6.040732781 8.29E-09 4.41E-08 9.705643041 UP CTD-3162L10.1
POU3F2 1.586681634 -4.123430967 6.03675492 8.46E-09 4.50E-08 9.693404833 UP POU3F2
FDCSP 2.991426831 -2.923032652 6.034649341 8.56E-09 4.55E-08 9.637585684 UP FDCSP
RP11_418I22.2 -1.240713091 -5.493779813 -6.034334124 8.57E-09 4.55E-08 9.684312807 DOWN RP11-418I22.2
SPRED3 1.203929893 -1.507770304 6.033806484 8.59E-09 4.56E-08 9.608468117UP SPRED3
CALCRL -1.07432659 3.597714201 -6.032176748 8.67E-09 4.60E-08 9.154846737 DOWN CALCRL
HOXB13 2.608185725 -4.199253329 6.030735362 8.73E-09 4.63E-08 9.654630477 UP HOXB13
OVOL2 2.234505131 -3.793108311 6.028918108 8.81E-09 4.68E-08 9.642596381 UP OVOL2
NRN1 -1.11947462 1.215243719 -6.02759542 8.87E-09 4.71E-08 9.413174204 DOWN NRN1
RP11_396O20.2 -1.25105505 -5.912858678 -6.027550108 8.88E-09 4.71E-

08 9.648813254 DOWN RP11-396O20.2

RP11_20J15.2 1.694361648 -4.964227508 6.026078412 8.94E-09 4.74E-08 9.643247043 UP RP11-20J15.2

LRP12 1.277629688 1.319057594 6.023441003 9.07E-09 4.80E-08 9.338237526 UP LRP12

RP11_618I10.2 2.661025265 -3.704215561 6.021747451 9.15E-09 4.84E-08 9.598996882 UP RP11-618I10.2

CTD_3128G10.61.29245763 -3.656377439 6.021676518 9.15E-09 4.84E-08 9.614429502 UP CTD-3128G10.6

AP001331.1 -2.070781014 -4.722531148 -6.02138433 9.16E-09 4.85E-08 9.620461016 DOWN AP001331.1

CXCL2 -1.644461591 4.288461025 -6.018662245 9.29E-09 4.91E-08 9.032956039 DOWN CXCL2

IGDCC3 2.36334955 -3.166091357 6.018573485 9.30E-09 4.91E-08 9.572215397 UP IGDCC3

RP4_794I6.4 1.425514682 -2.917047571 6.018014743 9.32E-09 4.93E-08 9.578597199 UP RP4-794I6.4

P2RX3 -1.665394675 -2.405441955 -6.016902046 9.38E-09 4.95E-08 9.582575992 DOWN P2RX3

AUTS2 -1.347554119 3.66193812 -6.015045777 9.47E-09 5.00E-08 9.069810422 DOWN AUTS2

XAF1 -1.498668666 1.780491953 -6.011504468 9.64E-09 5.08E-08 9.284472972 DOWN XAF1

MT1CP -1.931681218 -4.907490805 -6.008814708 9.78E-09 5.15E-08 9.557712146 DOWN MT1CP

ANGPTL1 -1.948003635 1.381897843 -6.007632462 9.84E-09 5.18E-08 9.314824209 DOWN ANGPTL1

TF -2.03527043 11.82576774-6.007016844 9.87E-09 5.19E-08 8.971977443 DOWN TF

C2 -1.328477501 8.523129215 -6.002043764 1.01E-08 5.32E-08 8.863070435 DOWN C2

MND1 1.033674 0.629721928 6.001558431 1.01E-08 5.34E-08 9.298480418 UP MND1

CLCF1 1.135403265 1.34582277 6.001026863 1.02E-08 5.35E-08
9.224955471 UP CLCF1
AC006273.4 1.565870472 -3.623865127 6.000484453 1.02E-08 5.36E-08 9.505102753 UP AC006273.4
ADAM9 1.004313057 4.598914584 5.997308554 1.04E-08 5.45E-08 8.870553536 UP ADAM9
CCDC28B 1.059105081 1.660480989 5.996287581 1.04E-08 5.47E-08 9.167283295 UP CCDC28B
GLS2 -2.908954135 -0.080553559 -5.996222826 1.04E-08 5.47E-08
9.386073634 DOWN GLS2
RGS6 -1.553281995 -2.884919815 -5.993187002 1.06E-08 5.56E-08
9.473668625 DOWN RGS6
SLCO1A2 -1.872315231 1.327306892 -5.991336674 1.07E-08 5.61E-08 9.235272429 DOWN SLCO1A2
GJC3 -1.368032429 -1.32245092 -5.990401118 1.07E-08 5.63E-08
9.408218779 DOWN GJC3
C19orf84 1.353031606 -4.454046949 5.990031917 1.08E-08 5.64E-08 9.463835036 UP C19orf84
RP11_456H18.2 -1.87998298 -1.227870852 -5.989978163 1.08E-08 5.64E-08 9.406299732 DOWN RP11-456H18.2
ATP8B2 1.018384577 3.098673096 5.98990338 1.08E-08 5.64E-08
8.967565677 UP ATP8B2
FOLR1 2.507877549 -2.104893435 5.988046582 1.09E-08 5.69E-08
9.387063376 UP FOLR1
SOX5 -1.31459331 2.423865813 -5.987681786 1.09E-08 5.70E-08
9.084174365 DOWN SOX5
ATF3 -1.090338789 4.737216789 -5.987118888 1.09E-08 5.72E-08
8.821231302 DOWN ATF3
ARHGAP22 1.039130891 0.383983319 5.986301738 1.10E-08 5.74E-08 9.243846427 UP ARHGAP22
LAMP3 1.475597631 0.583782852 5.986107874 1.10E-08 5.74E-08
9.219131936 UP LAMP3
SERPINI1 1.059791221 1.867783349 5.985377202 1.10E-08 5.76E-

08 9.088936302 UP SERPINI1
PTPRG_AS1 1.445948307 -2.917067736 5.984499052 1.11E-08 5.79E-08 9.411279531UP PTPRG-AS1
DPP4 -1.465950515 5.701364055 -5.9825847 1.12E-08 5.84E-08 8.761835957 DOWN DPP4
EPHA10 2.479611109-2.384111898 5.982302032 1.12E-08 5.85E-08 9.368654214 UP EPHA10
RP11_130L8.2 -1.774575632 1.077098454 -5.980397539 1.13E-08 5.90E-08 9.200438538 DOWN RP11-130L8.2
KIF12 1.697027752 3.752852902 5.978621961 1.14E-08 5.95E-08 8.850228255 UP KIF12
RP11_169K16.6 -2.049105348 -1.140667292 -5.975391002 1.16E-08 6.05E-08 9.330993776 DOWN RP11-169K16.6
LINC01428 -1.695355349 -3.313269338 -5.97475676 1.17E-08 6.07E-08 9.386457469 DOWN LINC01428
RP11_250B2.3 -1.115857638 -2.037860232 -5.9744794 1.17E-08 6.08E-08 9.359090553 DOWN RP11-250B2.3
EVPL 2.380364966 -1.352096854 5.971962586 1.18E-08 6.15E-08 9.276461806 UP EVPL
OXTR 1.650530209 -0.512481529 5.970765674 1.19E-08 6.19E-08 9.227307341 UP OXTR
EFCAB1 -1.620058825 -3.168443214 -5.970044973 1.19E-08 6.21E-08 9.361986048 DOWN EFCAB1
HES4 1.08975774 1.362624903 5.968996794 1.20E-08 6.24E-08 9.063111176UP HES4
RP11_167N24.3 -1.467930535 -5.451021735 -5.967283321 1.21E-08 6.30E-08 9.350669964 DOWN RP11-167N24.3
TMEM155 1.527493534 -4.422424525 5.967062207 1.21E-08 6.30E-08 9.349205607 UP TMEM155
MBOAT4 1.895060505 -1.924989235 5.966294801 1.22E-08 6.32E-08 9.282199912 UP MBOAT4
RP11_611O2.2 -1.951267124 -0.864649117 -5.965951478 1.22E-08 6.33E-08 9.268747561 DOWN RP11-611O2.2

HERC5 -1.34737683 2.496036809 -5.965257507 1.22E-08 6.35E-08 8.9681006 DOWN HERC5

AP000344.3 1.588213434 -4.961169631 5.964045216 1.23E-08 6.39E-08 9.335104648 UP AP000344.3

RP11_380M21.2 -1.13468364 -2.627353016 -5.96346939 1.23E-08 6.41E-08 9.322356075 DOWN RP11-380M21.2

RP11_455O6.9 1.26031529 -3.428116916 5.962924395 1.24E-08 6.42E-08 9.318330301 UP RP11-455O6.9

SYT13 3.327119323-1.733123111 5.961812753 1.25E-08 6.46E-08 9.230118085UP SYT13

CTC_336P14.1 -1.151143688 -2.845092068 -5.961005611 1.25E-08 6.48E-08 9.314185003 DOWN CTC-336P14.1

GLP1R 2.318500672 -3.993392074 5.960309157 1.25E-08 6.51E-08 9.303846172 UP GLP1R

CTC_265F19.2 -1.428668071 -4.226395403 -5.958205627 1.27E-08 6.57E-08 9.307561499 DOWN CTC-265F19.2

CTC_231O11.1 1.512172218 -2.250592349 5.95803445 1.27E-08 6.58E-08 9.258373623 UP CTC-231O11.1

GOLGA8M -1.473724068 -2.557184072 -5.956310926 1.28E-08 6.63E-08 9.285231196DOWN GOLGA8M

PDGFRL 1.236444104 0.01013469 5.956144355 1.28E-08 6.63E-08 9.121039882 UP PDGFRL

RP3_329A5.8 1.003050533 -2.024352774 5.95555959 1.29E-08 6.65E-08 9.245020089 UP RP3-329A5.8

SLC4A3 2.290252902 -0.857984664 5.95358122 1.30E-08 6.71E-08 9.157606309 UP SLC4A3

HOXB9 2.097380257 -4.152060736 5.952880212 1.30E-08 6.74E-08 9.272568027 UP HOXB9

GPAM -1.631382963 6.740178492 -5.950159065 1.32E-08 6.83E-08 8.585731226 DOWN GPAM

LTB 1.542955765 2.453952533 5.9452136 1.36E-08 7.00E-08 8.816008226 UP LTB

RP4_796I8.1 -1.191228836 -6.184404153 -5.943987356 1.36E-08

7.04E-08	9.232598435	DOWN RP4-796I8.1
RP11_161I6.2	1.611275446-4.996838819	5.943279203	1.37E-08	7.06E-08	9.232328875	UP	RP11-161I6.2
RP11_548L20.1 -2.328037392	-2.300346924	-5.942855287	1.37E-08	7.08E-08	9.210408359	DOWN RP11-548L20.1
GPX3	-1.453017167	8.738940428	-5.94257983 1.37E-08	7.08E-08	8.568666767	DOWN GPX3
BBOX1 -2.67776708 1.667303084	-5.939271827	1.40E-08	7.19E-08	8.963466809	DOWN BBOX1
SCG3	1.755549789	-4.071502276	5.938216778	1.41E-08	7.23E-08	9.202020447	UP	SCG3
RP11_15A1.2	1.685411529-3.757564877	5.937891473	1.41E-08	7.24E-08	9.195906364	UP	RP11-15A1.2
DAB1	-1.702207252	0.926354832	-5.937015781	1.41E-08	7.27E-08	8.99733251 DOWN DAB1
BSND	1.592687271	-4.963708844	5.935813925	1.42E-08	7.31E-08	9.195476973	UP	BSND
APOA2 -2.386490346	12.12609976	-5.934411423	1.43E-08	7.37E-08	8.615766037	DOWN APOA2
ANO7	1.000709343	0.856478233	5.934219301	1.43E-08	7.37E-08	8.941678903	UP	ANO7
HAPLN3	1.289815774	1.037318159	5.933974477	1.44E-08	7.38E-08	8.918011409UP	HAPLN3
PDXP	-1.160478028	0.698828793	-5.933945482	1.44E-08	7.38E-08	8.991496136	DOWN PDXP
RGS1	1.599464909	3.168236844	5.932865908	1.44E-08	7.42E-08	8.676879069	UP	RGS1
CCL20	2.523550723	2.908879549	5.932682722	1.45E-08	7.42E-08	8.713390869	UP	CCL20
COLEC10	-2.189762765	-0.424956821	-5.928111526	1.48E-08	7.59E-08	9.058495886	DOWN COLEC10
LINC01564 -1.554565042	-0.069479874	-5.924511704	1.51E-08	7.73E-08	9.009041876	DOWN LINC01564

RP11_284H18.1	-1.755691374	-4.843967512	-5.921121155	1.53E-08	7.86E-08	9.123657414	DOWN	RP11-284H18.1
PRAMEF9	-2.321854908	-4.675650312	-5.921119493	1.53E-08	7.86E-08	9.123138399	DOWN	PRAMEF9
RP11_360L9.4	-1.18503829	-3.986701367	-5.917520814	1.56E-08	8.00E-08	9.106560136	DOWN	RP11-360L9.4
CTXN1	1.66972717	-1.39118072	5.916773412	1.57E-08	8.03E-08	9.014256838	UP	CTXN1
USH1G	1.489185149	-5.144071185	5.914980119	1.58E-08	8.10E-08	9.092807188	UP	USH1G
MT1A	-2.427837132	1.587156329	-5.910950378	1.62E-08	8.25E-08	8.822813444	DOWN	MT1A
EN2	2.227806322	-3.965213183	5.910429759	1.62E-08	8.27E-08	9.058127726	UP	EN2
CHRNA4	-3.931005768	0.381453978	-5.909269093	1.63E-08	8.32E-08	8.938238382	DOWN	CHRNA4
AQP7P1	-2.024139384	-0.886053175	-5.909096214	1.63E-08	8.33E-08	8.988593659	DOWN	AQP7P1
SNORA73B	1.351436829	-3.08274387	5.90520949	1.66E-08	8.49E-08	9.024132422	UP	SNORA73B
S100A3	1.44391007	-2.010618511	5.903572622	1.68E-08	8.55E-08	8.981212213	UP	S100A3
SLC30A8	1.772397253	-5.168058942	5.902798603	1.68E-08	8.58E-08	9.032409243	UP	SLC30A8
RP11_1221G12.3	-1.639622175	-4.730760984	-5.901322959	1.70E-08	8.65E-08	9.026494207	DOWN	RP11-1221G12.3
FYTTD1P1	-2.043847926	-2.06135095	-5.900607783	1.70E-08	8.68E-08	8.996987675	DOWN	FYTTD1P1
RP3_342P20.2	-1.483294769	-2.812410432	-5.899646058	1.71E-08	8.72E-08	9.010217753	DOWN	RP3-342P20.2
SERPINA4	-2.353986871	6.600295153	-5.898481053	1.72E-08	8.76E-08	8.334153842	DOWN	SERPINA4
CORO6	1.161856851	-1.168552418	5.897085677	1.73E-08	8.82E-08			

8.910326492 UP CORO6
TNFSF4 1.060219939 1.1175403115.897005264 1.73E-08 8.82E-08
8.730711943UP TNFSF4
RP11_172H24.4 1.364998469 -3.739569689 5.896681264 1.74E-08
8.84E-08 8.996015371 UP RP11-172H24.4
CAGE1 1.616614182 -4.44329589 5.895229039 1.75E-08 8.90E-08
8.994434004 UP CAGE1
RP11_91I8.2 1.517918397 -5.503343556 5.894554157 1.76E-08
8.92E-08 8.991822597 UP RP11-91I8.2
GLTPD2 -1.715865387 3.809414604 -5.89443957 1.76E-08 8.93E-08
8.458232861 DOWN GLTPD2
RP11_744D14.1 -1.064902016 -1.961151252 -5.89277753 1.77E-08 9.00E-08 8.951764162 DOWN RP11-744D14.1
SPINT2 2.0281194973.06341689 5.886979459 1.83E-08 9.26E-08
8.464256161 UP SPINT2
YBX3 1.11629435 4.470140243 5.882048273 1.87E-08 9.48E-08
8.303471945 UP YBX3
CHST6 1.442193461 -3.228565961 5.880405054 1.89E-08 9.56E-08
8.904500927 UP CHST6
RP11_399O19.9 -1.013362071 -1.67476689 -5.879919541 1.89E-08 9.58E-08 8.87576616 DOWN RP11-399O19.9
RP11_848P1.5 1.012588402 -2.838737503 5.877620063 1.91E-08
9.68E-08 8.886723841 UP RP11-848P1.5
AC092667.2 1.633959351 -3.010350261 5.875953856 1.93E-08 9.76E-08 8.874611172UP AC092667.2
RP11_69L16.6 1.229763728 -5.736064942 5.87290189 1.96E-08 9.91E-08 8.885338952 UP RP11-69L16.6
USP50 -1.062166148 -4.000101714 -5.871093594 1.98E-08 1.00E-07
8.878475462 DOWN USP50
CKM 1.297356576 -4.074553829 5.869619781 1.99E-08 1.01E-07
8.867751775 UP CKM
RP11_15F12.1 1.289446125 -4.402889829 5.868694574 2.00E-08
1.01E-07 8.865182229 UP RP11-15F12.1

TMPRSS13 1.681264959 -2.132089449 5.8684711572.01E-08 1.01E-07 8.809464533 UP TMPRSS13

AL163953.3 1.233552057 -3.114614344 5.866271264 2.03E-08 1.02E-07 8.835707463 UP AL163953.3

BCKDHA -1.000499463 2.08852275 -5.866101905 2.03E-08 1.02E-07 8.511876065DOWN BCKDHA

SLCO5A1 1.677250321 -2.206911641 5.863093423 2.06E-08 1.04E-07 8.786397495 UP SLCO5A1

C16orf74 1.275321453 -1.071021684 5.860230173 2.09E-08 1.05E-07 8.721597516 UP C16orf74

DDX60 -1.089550658 3.775682251 -5.859853358 2.09E-08 1.05E-07 8.273438489 DOWN DDX60

SLC12A1 -2.999524335 -2.166826312 -5.858238409 2.11E-08 1.06E-07 8.787998658 DOWN SLC12A1

RP1_228P16.1 1.1574058 -2.988085232 5.855657457 2.14E-08 1.08E-07 8.781352537 UP RP1-228P16.1

PMEL -1.111997501 1.98328867 -5.854348758 2.15E-08 1.08E-07 8.468817724 DOWN PMEL

WNT9B-1.621200154 -4.034332056 -5.852374043 2.18E-08 1.09E-07 8.786625093 DOWN WNT9B

RP11_22C11.2 1.797390161 -5.019175282 5.851484959 2.19E-08 1.10E-07 8.780712075 UP RP11-22C11.2

AC004019.13 -1.403992336 -1.472425225 -5.850869398 2.19E-08 1.10E-07 8.726325768 DOWN AC004019.13

SLC17A1 -1.846171973 2.931172038-5.849441374 2.21E-08 1.11E-07 8.350089315 DOWN SLC17A1

QPCT 1.309035159 0.577833749 5.848141541 2.22E-08 1.11E-07 8.537330137 UP QPCT

CACNG4 2.712173931 -1.427778509 5.847124501 2.23E-08 1.12E-07 8.657868564 UP CACNG4

NRIP3 1.11238918 -2.302020491 5.84643847 2.24E-08 1.12E-07 8.716252987 UP NRIP3

RP11_400G3.5 -1.665568767 -3.71278092 -5.845514772 2.25E-08 1.13E-

07 8.752399936 DOWN RP11-400G3.5
SATB1_AS1 1.654323475 -3.106415342 5.845194518 2.26E-08 1.13E-07 8.726430635 UP SATB1-AS1
LINC01096 1.249218392 -5.842319458 5.843614763 2.27E-08 1.14E-07 8.741779427 UP LINC01096
HOXC6 2.277368662 -3.507720944 5.84343845 2.28E-08 1.14E-07 8.717683449 UP HOXC6
ERICH3 -1.618625241 -3.130610295 -5.841929941 2.29E-08 1.15E-07 8.731106992 DOWN ERICH3
C7 -2.618156814 4.418830423 -5.841456856 2.30E-08 1.15E-07 8.178319896 DOWN C7
RP11_430H12.2 -1.548062576 -4.354746596 -5.841151732 2.30E-08 1.15E-07 8.73197318 DOWN RP11-430H12.2
RP11_1008C21.1 1.301839353 -4.556222932 5.840307051 2.31E-08 1.16E-07 8.726717207 UP RP11-1008C21.1
ARHGEF38 1.746936459 -1.684705325 5.836709389 2.36E-08 1.18E-07 8.634982939 UP ARHGEF38
HCN4 1.961466117 -4.194165112 5.83467799 2.38E-08 1.19E-07 8.693714052 UP HCN4
ABCC2 -1.697970804 6.276905955 -5.832898648 2.40E-08 1.20E-07 8.006901903 DOWN ABCC2
WDR66 1.047061007 -0.431942775 5.829643868 2.44E-08 1.22E-07 8.53191049 UP WDR66
RP11_478P10.1 -1.528446066 -4.723956474 -5.828776126 2.45E-08 1.22E-07 8.67134671 DOWN RP11-478P10.1
CDH17 2.278595942 -3.157893756 5.826170671 2.48E-08 1.24E-07 8.625999612 UP CDH17
TAF7L -1.577505732 -3.008987765 -5.825994778 2.49E-08 1.24E-07 8.65203924 DOWN TAF7L
ZNF610 1.378300444 -1.319603147 5.824804427 2.50E-08 1.25E-07 8.561092925 UP ZNF610
ABCB1 -1.808763749 4.70203554 -5.823598153 2.52E-08 1.25E-07 8.029152161 DOWN ABCB1

PODXL2 2.476962007 0.779363287 5.822940731 2.53E-08 1.26E-07 8.37471835 UP PODXL2
NPTX2 3.12383797 -0.187742312 5.821401569 2.55E-08 1.27E-07 8.446229267 UP NPTX2
CALCR 1.701691836 -4.226482675 5.815622031 2.62E-08 1.30E-07 8.602934286 UP CALCR
RP11_308D16.2 1.134322841 -2.43012336 5.814660909 2.63E-08 1.31E-07 8.56582085 UP RP11-308D16.2
STK33 2.125525169 -2.935662359 5.813324318 2.65E-08 1.32E-07 8.558691683 UP STK33
ZNF790_AS1 1.240524823 -1.665843238 5.812912722 2.66E-08 1.32E-07 8.52321524 UP ZNF790-AS1
RP3_523K23.2 2.315380131 -4.306134616 5.812464809 2.66E-08 1.32E-07 8.58379365 UP RP3-523K23.2
CTD_2291D10.41.409920563 -5.143167196 5.8114014372.68E-08 1.33E-07 8.585760012 UP CTD-2291D10.4
CTD_2547L16.1-1.225694537 -2.440854902 -5.810213456 2.69E-08 1.34E-07 8.56497744 DOWN CTD-2547L16.1
ACSM1 -2.675946071 2.764652522 -5.809199931 2.71E-08 1.34E-07 8.205066869 DOWN ACSM1
RNU1_138P-1.192178581 -5.516924459 -5.808147605 2.72E-08 1.35E-07 8.569379428 DOWN RNU1-138P
SIX4 1.664619032 -0.856260332 5.807427798 2.73E-08 1.35E-07 8.444903848 UP SIX4
APCDD1 2.139404545 1.075167086 5.804299564 2.77E-08 1.37E-07 8.261410091 UP APCDD1
CUZD1 1.483380055 -2.311347884 5.803597487 2.78E-08 1.38E-07 8.502257617 UP CUZD1
MIR3685 1.023407656 -2.933988572 5.803577933 2.78E-08 1.38E-07 8.527708168 UP MIR3685
RP11_546J1.1 1.037698473 -2.645279603 5.801929601 2.81E-08 1.39E-07 8.511210906UP RP11-546J1.1
RP11_59H7.3 1.163481697 -4.158351776 5.800773664 2.82E-08

1.40E-07 8.532965976 UP RP11-59H7.3

NPHP1 1.003560785 -0.083929065 5.800715721 2.83E-08 1.40E-07 8.365262677 UP NPHP1

FMO1 2.021009425 -0.534954824 5.799389615 2.84E-08 1.40E-07 8.379400384 UP FMO1

SOCS2_AS1 -1.275919811 0.086631768 -5.797631585 2.87E-08 1.42E-07 8.370867985 DOWN SOCS2-AS1

RP11_469A15.2 -1.747961251 0.921976243 -5.793325844 2.93E-08 1.45E-07 8.290449243 DOWN RP11-469A15.2

S100A14 2.323750228 2.334937639 5.793275833 2.93E-08 1.45E-07 8.07725644 UP S100A14

VWDE 2.024915096 -4.224350908 5.793038456 2.94E-08 1.45E-07 8.490892485 UP VWDE

LGALS3BP 1.483267689 7.870030501 5.792161306 2.95E-08 1.45E-07 7.82066876 UP LGALS3BP

RP11_465B22.3 1.600155803 -0.16902041 5.79209439 2.95E-08 1.45E-07 8.320888034 UP RP11-465B22.3

FBLN5 -1.45728169 3.919739344 -5.790835491 2.97E-08 1.46E-07 7.929183105 DOWN FBLN5

PAK7 1.819956019 -5.101466286 5.790556343 2.97E-08 1.46E-07 8.483804158 UP PAK7

RP11_1260E13.4 -1.293658094 -2.471898062 -5.788873175 3.00E-08 1.48E-07 8.46205861 DOWN RP11-1260E13.4

SULT1A2 -1.656830685 2.963043036 -5.786268356 3.04E-08 1.50E-07 8.027540773 DOWN SULT1A2

GAS2 -1.212795052 3.199394275 -5.785849965 3.05E-08 1.50E-07 7.980759137 DOWN GAS2

MSH5 1.09590728 0.29813781 5.785216171 3.05E-08 1.50E-07 8.256660247 UP MSH5

SH2D7 1.08965516 -5.170754718 5.783808856 3.08E-08 1.51E-07 8.452048051 UP SH2D7

SPERT 1.773654083 -5.111091035 5.782774461 3.09E-08 1.52E-07 8.446114825UP SPERT

GPR63 1.40231945 -3.495105048 5.781940982 3.11E-08 1.52E-07 8.430858234 UP GPR63

ARHGEF7_AS2 -1.438884442 -5.024627706 -5.781504406 3.11E-08 1.53E-07 8.441038561 DOWN ARHGEF7-AS2

RP11_19E11.1 2.221219021 -4.364688118 5.777355069 3.18E-08 1.56E-07 8.414399842 UP RP11-19E11.1

TCF15 -1.118297943 -1.031263691 -5.777023011 3.18E-08 1.56E-07 8.339832399 DOWN TCF15

RGMB_AS1-1.386918926 -1.040802931 -5.775455765 3.21E-08 1.57E-07 8.335578168 DOWN RGMB-AS1

RP11_24F11.2 1.281125226-3.46711882 5.774684285 3.22E-08 1.58E-07 8.396448989 UP RP11-24F11.2

PPP1R9A 1.774197381 1.0435711775.77412673 3.23E-08 1.58E-07 8.122756507 UP PPP1R9A

SLC31A2 -1.115211526 -0.305335629 -5.773827793 3.23E-08 1.58E-07 8.279006063 DOWN SLC31A2

ANXA13 2.5181112222.61844051 5.7734511913.24E-08 1.59E-07 7.953270348 UP ANXA13

TNIK 1.535333697 1.724668799 5.772597276 3.26E-08 1.59E-07 8.046604135 UP TNIK

FAM83D 1.043781994 2.7175301155.771579395 3.27E-08 1.60E-07 7.92947921 UP FAM83D

SLC30A10 -1.429455078 4.713422126 -5.768946038 3.32E-08 1.62E-07 7.747253904 DOWN SLC30A10

MS4A8 2.311134936-4.51323421 5.768497768 3.32E-08 1.62E-07 8.372532085 UP MS4A8

RP11_350E12.4 1.623539818 -4.236627087 5.768078344 3.33E-08 1.63E-07 8.372695724 UP RP11-350E12.4

SCN4B -1.339737347 0.974134932 -5.767480743 3.34E-08 1.63E-07 8.15164881 DOWN SCN4B

MAGI2_AS3 -1.741402693 2.220271376 -5.766816499 3.35E-08 1.63E-07 8.029340177 DOWN MAGI2-AS3

ELOVL3 1.6651249 -3.892495944 5.7661151923.36E-08 1.64E-07

8.358446162 UP ELOVL3
MYBPC2 1.596848436 -4.150472026 5.765763305 3.37E-08 1.64E-07 8.360844035 UP MYBPC2
IFIT2 -1.038584503 3.687217752 -5.764612851 3.39E-08 1.65E-07 7.812865629 DOWN IFIT2
ZNF257 1.606188602 -1.884740147 5.763723754 3.40E-08 1.66E-07 8.290022681 UP ZNF257
INSL3 1.356163011 -4.003037299 5.763477638 3.41E-08 1.66E-07 8.349676351 UP INSL3
CHGB 2.139585837 -3.830788037 5.757237194 3.52E-08 1.71E-07 8.308390902 UP CHGB
MSI1 2.349283643 0.163853604 5.757075565 3.52E-08 1.71E-07 8.110760709 UP MSI1
AVPR2 -1.343911044 -2.443070419 -5.756581304 3.53E-08 1.72E-07 8.304786987 DOWN AVPR2
HKDC1 2.223381648 3.606863432 5.754315124 3.57E-08 1.74E-07 7.761748693 UP HKDC1
ADAM22 1.466451798 -0.480278643 5.754306175 3.57E-08 1.74E-07 8.162500363 UP ADAM22
INMT -1.557378759 2.254824031 -5.753699414 3.58E-08 1.74E-07 7.955344453 DOWN INMT
CH17_125A10.1 -1.087335718 -6.223033068 -5.752235254 3.61E-08 1.75E-07 8.29510705 DOWN CH17-125A10.1
TUB 1.820153517 -0.192079367 5.752189282 3.61E-08 1.75E-07 8.12561265 UP TUB
LRRC37A6P 1.591478584 -1.947103296 5.751785743 3.61E-08 1.76E-07 8.235568522 UP LRRC37A6P
TMEM246 1.79739277 1.690318734 5.751268281 3.62E-08 1.76E-07 7.94299467 UP TMEM246
RP11_172E9.2 -1.669775166 -4.930765542 -5.750019674 3.65E-08 1.77E-07 8.288554915 DOWN RP11-172E9.2
ZG16 -2.701844733 1.381475197 -5.74822617 3.68E-08 1.79E-07 8.046827282 DOWN ZG16

SEMA4F 1.295857786 1.549569261 5.747487439 3.69E-08 1.79E-07 7.946642945 UP SEMA4F
GRIK5 1.197494542 -2.223599249 5.745890595 3.72E-08 1.80E-07 8.222420922 UP GRIK5
ITIH2 -1.497801705 10.39195951 -5.744262319 3.75E-08 1.82E-07 7.628731549 DOWN ITIH2
SLC22A3 -1.633020576 4.290019129 -5.743598495 3.76E-08 1.82E-07 7.663075782 DOWN SLC22A3
AC016700.5 -1.254405542 0.210311309 -5.742970083 3.78E-08 1.83E-07 8.093646905 DOWN AC016700.5
TRIM6 1.463939165 -0.022686434 5.742496048 3.79E-08 1.83E-07 8.070047339 UP TRIM6
CLDN15 -1.381295917 4.434930974 -5.739592921 3.84E-08 1.86E-07 7.623692833 DOWN CLDN15
B4GALNT2 2.973425328 -2.97401071 5.739411543 3.84E-08 1.86E-07 8.185813261 UP B4GALNT2
RP11_209K10.2 -1.534199791 0.554599994 -5.739408446 3.84E-08 1.86E-07 8.053938582 DOWN RP11-209K10.2
TTYH1 2.117063243 -0.925104052 5.735123893 3.93E-08 1.90E-07 8.091739438 UP TTYH1
CTC_277H1.7 1.185136644 -3.148680077 5.734417977 3.94E-08 1.90E-07 8.195594873 UP CTC-277H1.7
TBC1D3L 1.281088615 -1.499827727 5.733137389 3.97E-08 1.91E-07 8.126674106 UP TBC1D3L
CTC_510F12.4 1.28247165 -3.753076302 5.73081421 4.01E-08 1.94E-07 8.189115217 UP CTC-510F12.4
RP11_64D22.5 -1.667824326 -3.825535244 -5.730777695 4.01E-08 1.94E-07 8.195862137 DOWN RP11-64D22.5
RP11_260M19.2 -1.656139097 -4.17066723 -5.72955494 4.04E-08 1.95E-07 8.190449579 DOWN RP11-260M19.2
SBK1 1.31558696 -0.482725708 5.729473347 4.04E-08 1.95E-07 8.044098606 UP SBK1
RP11_12A20.7 1.395370848 -4.341372789 5.721799484 4.20E-08

2.02E-07 8.150984799 UP RP11-12A20.7

AC092198.1 1.300729586 -5.141310264 5.720682684 4.22E-08 2.03E-07 8.146769228 UP AC092198.1

SLC25A36 1.065150069 2.027221775 5.720079929 4.24E-08 2.04E-07 7.760830474 UP SLC25A36

VWFP1 -1.499747044 -4.275828387 -5.718276676 4.27E-08 2.05E-07 8.136284531 DOWN VWFP1

FAHD2B 1.548082865 0.525197515 5.717879374 4.28E-08 2.06E-07 7.902069855 UP FAHD2B

TFPI -1.297552097 7.224009179 -5.71646963 4.31E-08 2.07E-07 7.431525303 DOWN TFPI

HMGN2P47 -1.396648066 -3.08449198 -5.715657296 4.33E-08 2.08E-07 8.118896448 DOWN HMGN2P47

ASCL2 1.417408554 -1.509660521 5.715612176 4.33E-08 2.08E-07 8.040250604 UP ASCL2

PLIN1 -1.702363967 2.071542574 -5.714273297 4.36E-08 2.09E-07 7.786451157 DOWN PLIN1

APOC2 -1.753052107 5.019232976 -5.713128961 4.38E-08 2.10E-07 7.462008105 DOWN APOC2

DMRT2 1.666953053 -5.392434353 5.712212472 4.40E-08 2.11E-07 8.10522783 UP DMRT2

RP11_599B13.9 1.493287785 -5.424195056 5.709523949 4.46E-08 2.14E-07 8.092462796 UP RP11-599B13.9

HOXC9 2.478628376 -3.392697011 5.707037732 4.52E-08 2.16E-07 8.048884135 UP HOXC9

KIAA1549L 1.928673134 -2.778055915 5.707036899 4.52E-08 2.16E-07 8.043047361 UP KIAA1549L

CCDC78 1.276992286 -1.238468447 5.706130737 4.54E-08 2.17E-07 7.981232936 UP CCDC78

RP4_631H13.6 -1.463466795 -2.378000927 -5.705388456 4.56E-08 2.18E-07 8.056089035 DOWN RP4-631H13.6

ZIM2_AS1 1.597751035 -4.2210595 5.704668093 4.57E-08 2.19E-07 8.066675426 UP ZIM2-AS1

TLDC1 1.122637476 1.585009476 5.703377926 4.60E-08 2.20E-07 7.7308431 UP TLDC1

RP11_401P9.5 -1.699378633 -2.6810698 -5.702538833 4.62E-08 2.21E-07 8.048793792 DOWN RP11-401P9.5

RP11_777B9.5 -1.137789528 -5.123841855 -5.702321785 4.63E-08 2.21E-07 8.058972242 DOWN RP11-777B9.5

NTNG2 1.266316979 -1.66410678 5.7023215 4.63E-08 2.21E-07 7.986685442 UP NTNG2

RP11_1260E13.2 -1.522869741 -0.824519255 -5.70219469 4.63E-08 2.21E-07 7.970282925 DOWN RP11-1260E13.2

OCIAD2 -1.246387404 5.222495647 -5.700888221 4.66E-08 2.22E-07 7.383564069 DOWN OCIAD2

TMCC2 1.079576797 -0.763372089 5.699961277 4.68E-08 2.23E-07 7.923670995 UP TMCC2

UPP2_IT1 -1.74663423 -5.153704424 -5.698116287 4.73E-08 2.25E-07 8.037951576 DOWN UPP2-IT1

ACE2 -2.729293794 0.271315786 -5.697442092 4.74E-08 2.26E-07 7.892924572 DOWN ACE2

RP11_545A16.4 1.115134472 -5.603991186 5.696550677 4.76E-08 2.27E-07 8.030146837 UP RP11-545A16.4

CTC_510F12.7 -1.025689382 -2.014045732 -5.696244453 4.77E-08 2.27E-07 7.99775962 DOWN CTC-510F12.7

RP1_239B22.5 1.572164971 -0.682631469 5.694504416 4.81E-08 2.29E-07 7.886004386 UP RP1-239B22.5

AKR1C2 -1.88707959 7.048263195 -5.694401114 4.81E-08 2.29E-07 7.325329834 DOWN AKR1C2

SMARCD3 1.195488212 2.469045079 5.692341346 4.86E-08 2.31E-07 7.572450906 UP SMARCD3

CTD_2331H12.5 1.832657929 -2.461370531 5.691721811 4.88E-08 2.32E-07 7.960529562 UP CTD-2331H12.5

RP11_339B21.10 1.256093782 -4.591176735 5.690368286 4.91E-08 2.33E-07 8.00115084 UP RP11-339B21.10

PLEKHS1 1.977770611 -3.536621871 5.689511096 4.93E-08 2.34E-07

7.978082092 UP PLEKHS1
SLC34A2 3.299022321 -0.981016967 5.6863132 5.01E-08 2.38E-07
7.845013052 UP SLC34A2
DPYSL4 1.763435184 -2.027094187 5.685563307 5.03E-08 2.39E-07 7.915034327 UP DPYSL4
IL11 1.543294123 -3.491902617 5.683045515 5.09E-08 2.42E-07
7.951822369 UP IL11
CNKSR1 2.228028853 -0.954244477 5.682874865 5.10E-08 2.42E-07 7.841065627 UP CNKSR1
AQP7P2 -1.658538644 -4.472023709 -5.680574595 5.16E-08 2.44E-07 7.955089493 DOWN AQP7P2
LINC00671 -1.46244092 0.742549461 -5.68030591 5.16E-08 2.45E-07
7.751211839DOWN LINC00671
CTD_2566J3.1 1.589294823 -5.142108906 5.6801511075.17E-08 2.45E-07 7.951708761 UP CTD-2566J3.1
MAB21L3 -1.803552107 -1.370978072 -5.679025835 5.20E-08 2.46E-07 7.891571378 DOWN MAB21L3
CTLA4 1.592681114-1.055715074 5.678304122 5.22E-08 2.47E-07
7.832183393 UP CTLA4
ASXL3 -2.141581489 -2.694016136 -5.677086361 5.25E-08 2.48E-07
7.924918141 DOWN ASXL3
B3GALT5 2.266071686 -3.146754045 5.676467762 5.26E-08 2.49E-07 7.901016066 UP B3GALT5
PRR18 -1.637405906 0.803836712 -5.675809626 5.28E-08 2.50E-07
7.726867915 DOWN PRR18
RP11_715H19.2 -1.973109785 -4.295253422 -5.674742299 5.31E-08
2.51E-07 7.92689788 DOWN RP11-715H19.2
SCN5A 1.439306092 -3.887679283 5.6711000685.41E-08 2.55E-07
7.903051625 UP SCN5A
DBH -1.621106797 0.88545462 -5.670120118 5.43E-08 2.56E-07
7.692454079 DOWN DBH
RP11_379B8.1 -1.510952796 0.690555337 -5.669140622 5.46E-08
2.58E-07 7.702682574 DOWN RP11-379B8.1

ZNF425 -1.036615298 0.391324728 -5.66864624 5.47E-08 2.58E-07 7.71760925 DOWN ZNF425
LINC00265 1.162328026 -1.173329901 5.664637658 5.58E-08 2.63E-07 7.779386138 UP LINC00265
IL17RD 1.295477724 -0.692049002 5.660690275 5.69E-08 2.68E-07 7.727434931 UP IL17RD
SNHG14 1.501488141 3.402689961 5.659121217 5.74E-08 2.70E-07 7.308641001 UP SNHG14
SNX31 -1.275611571 -3.841359943 -5.657119664 5.79E-08 2.73E-07 7.842722991 DOWN SNX31
AVPR1A -2.695521489 1.246481337 -5.656268444 5.82E-08 2.74E-07 7.616395185 DOWN AVPR1A
ZNF492 1.508603395 -4.251024892 5.653517684 5.90E-08 2.77E-07 7.822502639 UP ZNF492
RP11_51F16.1 1.007729543 -1.008763979 5.65172359 5.95E-08 2.79E-07 7.708405856 UP RP11-51F16.1
CTSE 2.510505277 -2.765175618 5.651706117 5.95E-08 2.79E-07 7.767568775 UP CTSE
LRRC36 1.54256598 -3.590269203 5.651035861 5.97E-08 2.80E-07 7.800627845 UP LRRC36
ANKRD35 -2.013764639 0.244884589 -5.649826235 6.01E-08 2.82E-07 7.653927743 DOWN ANKRD35
DUXAP10 1.551257909 -3.036073505 5.648729623 6.04E-08 2.83E-07 7.776137016 UP DUXAP10
CHRNA1 1.750652409 -3.90216353 5.6486958 6.04E-08 2.83E-07 7.793026613 UP CHRNA1
MRC1 -1.359681037 3.526519982 -5.648052959 6.06E-08 2.84E-07 7.276327154 DOWN MRC1
BAIAP2L2 1.700213223 2.311481292 5.648023562 6.06E-08 2.84E-07 7.37334626 UP BAIAP2L2
RP5_1050E16.1 -1.840032473 -4.696778972 -5.647989325 6.06E-08 2.84E-07 7.798863879 DOWN RP5-1050E16.1
S100B 1.410068999 -1.250595152 5.646404905 6.11E-08 2.86E-07

7.693544799 UP S100B
ARHGEF19 1.023590346 1.184507622 5.645337805 6.14E-08 2.88E-07 7.495996041 UP ARHGEF19
BMP10 -1.491987708 -5.721106804 -5.643140587 6.21E-08 2.91E-07 7.773283111 DOWN BMP10
IL2RG 1.241586622 3.33396481 5.641966746 6.25E-08 2.92E-07 7.230054438 UP IL2RG
SLC25A24 1.320956333 1.703890867 5.641433074 6.26E-08 2.93E-07 7.415910101 UP SLC25A24
RP11_96B2.1 -1.885539122 -3.664014127 -5.640328683 6.30E-08 2.94E-07 7.761458897 DOWN RP11-96B2.1
GCGR -3.607495977 2.277971992 -5.639604213 6.32E-08 2.95E-07 7.460808446 DOWN GCGR
MDFI 1.663809651 0.607382145 5.6391033 6.34E-08 2.96E-07 7.512108454 UP MDFI
AP000695.6 -1.08446869 -1.310980363 -5.638783105 6.35E-08 2.96E-07 7.688968701 DOWN AP000695.6
DUSP5P1 1.51738025 -4.753119696 5.638607148 6.35E-08 2.97E-07 7.753346188 UP DUSP5P1
KB_1552D7.2 -1.397197818 -3.37628434 -5.635989466 6.43E-08 3.00E-07 7.739986635 DOWN KB-1552D7.2
CHST1 1.252838632 1.357748491 5.635673816 6.44E-08 3.01E-07 7.427186569 UP CHST1
PLXDC1 1.019558122 2.302601396 5.635392293 6.45E-08 3.01E-07 7.319495468 UP PLXDC1
DGCR5 -2.033243615 1.967776743 -5.635252016 6.46E-08 3.01E-07 7.427143048 DOWN DGCR5
KIRREL2 1.772925602 -4.547984259 5.635101882 6.46E-08 3.01E-07 7.73524842 UP KIRREL2
AC006126.4 -1.292659096 -0.88703806 -5.633591863 6.51E-08 3.03E-07 7.641867612 DOWN AC006126.4
PRR15L 2.328951945 0.399522517 5.632862824 6.53E-08 3.04E-07 7.491280842 UP PRR15L

RP11_2L8.1 -1.543237392 -3.016529048 -5.630018209 6.63E-08 3.08E-07 7.707725535 DOWN RP11-2L8.1

ITGB8 2.339724742 -1.050367257 5.628809649 6.67E-08 3.10E-07 7.586295266 UP ITGB8

RP3_407E4.2 -1.214806111 -5.403224311 -5.627082479 6.72E-08 3.13E-07 7.698371748 DOWN RP3-407E4.2

PRSS21 1.889261219 -3.918655434 5.626691439 6.74E-08 3.13E-07 7.686851275 UP PRSS21

PTPLA 1.708203051 -1.346689261 5.626617877 6.74E-08 3.13E-07 7.600809924 UP PTPLA

FAM105A 1.03739449 0.949619694 5.623795656 6.83E-08 3.18E-07 7.41621099 UP FAM105A

AC093609.1 -1.247253959 -2.78381237 -5.623672521 6.84E-08 3.18E-07 7.674634397 DOWN AC093609.1

KBTBD11 -1.709728088 1.620550933 -5.622823572 6.87E-08 3.19E-07 7.397492957 DOWN KBTBD11

ZFY_AS1 -1.851333761 -4.142809954 -5.620802141 6.94E-08 3.22E-07 7.669619889 DOWN ZFY-AS1

PRODH2 -2.326224537 5.072016877 -5.620591556 6.94E-08 3.22E-07 7.028076154 DOWN PRODH2

SVOP -1.623100077 -2.180263776 -5.619364837 6.99E-08 3.24E-07 7.638702929 DOWN SVOP

GDF10 2.278685682 -3.412781748 5.617152527 7.06E-08 3.27E-07 7.623393224 UP GDF10

RP11_113I22.1 -1.499521327 -5.614086529 -5.6162901 7.09E-08 3.29E-07 7.645880939 DOWN RP11-113I22.1

ARNTL2 1.366779903 2.436227988 5.616020489 7.10E-08 3.29E-07 7.207115313UP ARNTL2

CFAP70 -1.035132976 0.308063025 -5.614619578 7.15E-08 3.31E-07 7.465233714 DOWN CFAP70

SUSD4 2.488589891 1.4611842495.614065789 7.17E-08 3.32E-07 7.297197742 UP SUSD4

RPS2P32 1.226507119-2.343515403 5.6116671447.26E-08 3.36E-07

7.582223476 UP RPS2P32

C8orf88 1.560507448 -2.802147734 5.609624363 7.33E-08 3.39E-07 7.582966848 UP C8orf88

CD80 1.184352618 -2.147277443 5.609129224 7.35E-08 3.39E-07 7.563784824 UP CD80

AP000351.3 -1.824635863 -1.427440992 -5.60809927 7.38E-08 3.41E-07 7.555081579 DOWN AP000351.3

ELOVL4 1.43346432 -2.454964141 5.607308538 7.41E-08 3.42E-07 7.562636653 UP ELOVL4

HMCN2 -1.99773383 0.936536108 -5.60515874 7.49E-08 3.46E-07 7.383516022 DOWN HMCN2

SHBG -2.158393871 3.0050896 -5.604858411 7.50E-08 3.46E-07 7.161863449 DOWN SHBG

MTRNR2L6 -1.195531349 -3.940760996 -5.604695157 7.51E-08 3.46E-07 7.593325818 DOWN MTRNR2L6

CECR2 -1.608444061 2.433897842 -5.604275145 7.53E-08 3.47E-07 7.21004001 DOWN CECR2

NR1H4 -1.189661865 5.662818682 -5.603834347 7.54E-08 3.48E-07 6.898392266 DOWN NR1H4

CHD5 1.340059198 -4.143488888 5.601673886 7.62E-08 3.51E-07 7.575716751 UP CHD5

MARVELD1 1.058178321 2.579044539 5.601097721 7.64E-08 3.52E-07 7.120179033 UP MARVELD1

RP11_408B11.2 1.650747895 -5.256578075 5.595606228 7.85E-08 3.62E-07 7.548609558 UP RP11-408B11.2

GALP -1.730887667 -4.027732345 -5.594282035 7.91E-08 3.64E-07 7.543731388 DOWN GALP

GS1_306C12.1 -1.167188735 -5.775602435 -5.593252421 7.95E-08 3.65E-07 7.536302 DOWN GS1-306C12.1

XXbac_B444P24.10 -1.228148781 -1.700495742 -5.592118929 7.99E-08 3.67E-07 7.487812738 DOWN XXbac-B444P24.10

ZNF204P 1.758380043 -1.141469847 5.591767794 8.00E-08 3.68E-07 7.422033669 UP ZNF204P

GPC4 1.885892012 1.427004625 5.591754523 8.00E-08 3.68E-07 7.199874441 UP GPC4

SIGLEC10 1.173278421 0.640949083 5.589829801 8.08E-08 3.71E-07 7.281562204 UP SIGLEC10

RP11_406A20.4 -1.753834566 -4.464952374 -5.588112632 8.15E-08 3.74E-07 7.514590267 DOWN RP11-406A20.4

DFNA5 1.294528833 2.296668504 5.587860784 8.16E-08 3.74E-07 7.089123646 UP DFNA5

CTD_2026K11.5-1.204473496 -2.947686064 -5.587115069 8.19E-08 3.75E-07 7.503790294 DOWN CTD-2026K11.5

ESRP2 -1.072506373 5.171984598 -5.586203095 8.23E-08 3.77E-07 6.829457323 DOWN ESRP2

JAK3 1.100450983 2.335500027 5.583685956 8.33E-08 3.81E-07 7.065956706 UP JAK3

C4A -1.350761022 6.927029536 -5.583440742 8.34E-08 3.82E-07 6.788067938 DOWN C4A

ADCY10 -1.432057421 1.46732337 -5.580749875 8.45E-08 3.87E-07 7.204053693 DOWN ADCY10

NEFH 1.319572591 -1.625032981 5.579419202 8.51E-08 3.89E-07 7.396192792 UP NEFH

RP11_305L7.3 1.352568508 -4.099462248 5.579280187 8.51E-08 3.89E-07 7.469131839 UP RP11-305L7.3

RP3_468B3.2 -1.278703559 -3.687852451 -5.57822515 8.56E-08 3.91E-07 7.467325604 DOWN RP3-468B3.2

LA16c_60H5.7 1.369650505 -3.944121252 5.576234851 8.64E-08 3.95E-07 7.453076255 UP LA16c-60H5.7

RP11_380P13.1 -1.413195366 -4.616833149 -5.575664677 8.66E-08 3.96E-07 7.455893409 DOWN RP11-380P13.1

ADAM32 1.503794153 -3.233490134 5.575534869 8.67E-08 3.96E-07 7.434355591 UP ADAM32

B3GNT4 1.039337582 -2.012879128 5.57392531 8.74E-08 3.99E-07 7.393045279 UP B3GNT4

MLF1 1.418890503 -0.521714033 5.572629791 8.79E-08 4.01E-07

7.292876162 UP MLF1

RP11_725G5.2 -1.427974033 -3.023539501 -5.572444442 8.80E-08 4.02E-07 7.434940532 DOWN RP11-725G5.2

STXBP5_AS1 1.073738856 -1.969173234 5.57165987 8.84E-08 4.03E-07 7.379539187 UP STXBP5-AS1

BLACAT1 1.44116318 -5.175953153 5.56880976 8.96E-08 4.08E-07 7.422179183 UP BLACAT1

RP11_96H17.1 1.481416556 -5.627163722 5.564777062 9.14E-08 4.16E-07 7.402376241 UP RP11-96H17.1

CLIC3 1.395900962 -0.999620423 5.5631411289.21E-08 4.19E-07 7.280855599 UP CLIC3

NAMPTP1 -1.567739706 1.205566518 -5.562192194 9.26E-08 4.21E-07 7.146909264 DOWN NAMPTP1

PLS3_AS1 -1.101386846 -1.638637218 -5.561905493 9.27E-08 4.22E-07 7.340629516 DOWN PLS3-AS1

SLC9A7P1 1.479135786 -2.597475924 5.560779038 9.32E-08 4.24E-07 7.346667941 UP SLC9A7P1

AC069513.4 1.10588998 -3.455450644 5.560652177 9.33E-08 4.24E-07 7.373607249 UP AC069513.4

RP4_669P10.16 -1.789454563 -2.76305329 -5.559647005 9.37E-08 4.26E-07 7.369225644 DOWN RP4-669P10.16

MAGEA10 1.852344692 -5.355910416 5.558804067 9.41E-08 4.27E-07 7.374142802 UP MAGEA10

FOXP4_AS1 1.452222313 -2.332979468 5.558557443 9.42E-08 4.28E-07 7.325983742 UP FOXP4-AS1

LINC00840 -1.37729873 -4.158288408 -5.557634509 9.47E-08 4.29E-07 7.370823553 DOWN LINC00840

GABRR1 1.224873175 -5.328172225 5.557581864 9.47E-08 4.30E-07 7.369231502 UP GABRR1

CTD_2588C8.8 -1.169735747 -4.544474011 -5.556799461 9.50E-08 4.31E-07 7.367016086 DOWN CTD-2588C8.8

RP11_38H17.1 -1.592069457 -4.870117353 -5.55650018 9.52E-08 4.32E-07 7.364838749 DOWN RP11-38H17.1

MT1XP1 -1.727862756 -2.891028328 -5.555612304 9.56E-08 4.33E-07 7.35254014 DOWN MT1XP1

RP11_701H24.4 1.58913112 -3.952569742 5.555054134 9.59E-08 4.34E-07 7.351216601 UP RP11-701H24.4

LINC01572 1.284909162 -3.312471092 5.554477685 9.61E-08 4.36E-07 7.3396609 UP LINC01572

CTC_471J1.11 1.047032556 -2.41381093 5.553891404 9.64E-08 4.37E-07 7.313664262 UP CTC-471J1.11

RP11_274B18.4 -1.225153744 -5.860274532 -5.552554172 9.70E-08 4.39E-07 7.34344036 DOWN RP11-274B18.4

RP11_909N17.2 1.723953321 -4.624101651 5.552242831 9.72E-08 4.40E-07 7.343053703 UP RP11-909N17.2

LINC00501 1.430657284 -5.06160959 5.551700947 9.74E-08 4.41E-07 7.341574033 UP LINC00501

CELF4 1.509202957 -3.0256796 5.549312414 9.86E-08 4.46E-07 7.304509003 UP CELF4

OR7E126P 1.038918761 -4.440672754 5.548695966 9.89E-08 4.47E-07 7.327722107 UP OR7E126P

DNAH3 1.414250722 -3.659235557 5.546058554 1.00E-07 4.53E-07 7.305338431 UP DNAH3

EVPLL -1.740896112 -3.946295525 -5.545472247 1.00E-07 4.54E-07 7.312990577 DOWN EVPLL

RP11_44N12.5 1.372942003 -5.131421215 5.544515242 1.01E-07 4.56E-07 7.307733211UP RP11-44N12.5

LRRC70 -1.080959161 -2.421931745 -5.543227607 1.02E-07 4.59E-07 7.284805135 DOWN LRRC70

TAC3 1.48593567 -4.37474154 5.540860542 1.03E-07 4.64E-07 7.289189598 UP TAC3

ROR1 1.711368659-0.483812643 5.538082759 1.04E-07 4.70E-07 7.123027427 UP ROR1

SLC4A8 1.022878709 -1.756913752 5.537984067 1.04E-07 4.70E-07 7.210954645 UP SLC4A8

AC007952.5 1.330844143 -5.149798988 5.536625843 1.05E-07 4.73E-

07 7.270630643 UP AC007952.5

CTD_2562J17.6 1.005728147 1.572124832 5.536386818 1.05E-07 4.73E-07 6.932455628 UP CTD-2562J17.6

ZIM2 1.402110898-5.198799806 5.535253272 1.06E-07 4.76E-07 7.264014579 UP ZIM2

LINC01063 1.127775118-4.14382108 5.535252198 1.06E-07 4.76E-07 7.262909954 UP LINC01063

PIWIL4 1.495548189 -0.249353609 5.534830822 1.06E-07 4.77E-07 7.092684669 UP PIWIL4

KLRF1 -1.095494137 -2.140636146 -5.533167592 1.07E-07 4.80E-07 7.22760137 DOWN KLRF1

AL161645.1 -1.242197452 -5.47956641 -5.532086474 1.07E-07 4.83E-07 7.248663219 DOWN AL161645.1

LYPD2 -1.826068671 -4.499619604 -5.531366643 1.08E-07 4.84E-07 7.246722632 DOWN LYPD2

GCSHP5 -1.112617461 -3.397088575 -5.528815086 1.09E-07 4.90E-07 7.233208189 DOWN GCSHP5

NUDT12 -1.098094671 3.722642254 -5.528232577 1.09E-07 4.91E-07 6.670649115DOWN NUDT12

LINC00639 -1.110782944 -2.285636053 -5.527874456 1.10E-07 4.92E-07 7.20811263 DOWN LINC00639

HES2 1.543106179 -2.183135821 5.527620986 1.10E-07 4.92E-07 7.173926584 UP HES2

ARPP21 -2.178934117 -2.653060207 -5.524541628 1.11E-07 4.99E-07 7.199951315 DOWN ARPP21

RRS1_AS1 -1.232279302 -2.998812333 -5.523836763 1.12E-07 5.00E-07 7.20595214 DOWN RRS1-AS1

RP11_475O6.1 -1.28002953 -1.59280395 -5.523420479 1.12E-07 5.01E-07 7.158482439 DOWN RP11-475O6.1

PFN2 1.396195813 2.865901365 5.523387593 1.12E-07 5.01E-07 6.715099987 UP PFN2

AC005682.5 1.133327726 -1.180200177 5.522522399 1.12E-07 5.03E-07 7.103506294 UP AC005682.5

CNGB1	1.549066112	-4.166799267	5.520981411	1.13E-07	5.07E-07	7.193912061	UP	CNGB1
UNC13A	1.803185102	-2.27888996	5.516948645	1.16E-07	5.17E-07	7.122987355	UP	UNC13A
SLIT1	1.148573778	-2.201164541	5.514426657	1.17E-07	5.23E-07	7.118553588	UP	SLIT1
RP11_503C24.4	-1.515105901	-2.964245409	-5.514071799	1.17E-07	5.24E-07	7.158958897	DOWN	RP11-503C24.4
KRT18P34	-1.268233373	-0.864610328	-5.514010209	1.17E-07	5.24E-07	7.072910635	DOWN	KRT18P34
RP11_253E3.3	1.183877594	-1.26610502	5.513850691	1.17E-07	5.24E-07	7.068069782	UP	RP11-253E3.3
UNC13D	1.047106463	2.588486499	5.513209851	1.18E-07	5.25E-07	6.70068739	UP	UNC13D
SNX29P2	-1.321039812	-2.773042425	-5.512509986	1.18E-07	5.27E-07	7.149013507	DOWN	SNX29P2
CTC_459F4.1	1.062280508	-2.891163362	5.511348179	1.19E-07	5.30E-07	7.128544434	UP	CTC-459F4.1
LRRFIP1P1	1.268861578	-2.258857049	5.509325322	1.20E-07	5.35E-07	7.096036574	UP	LRRFIP1P1
RP1_197B17.4	-1.122532074	-2.711728444	-5.507885211	1.21E-07	5.39E-07	7.126276831	DOWN	RP1-197B17.4
MMP7	2.814565437	0.483121785	5.50768614	1.21E-07	5.39E-07	6.882838107	UP	MMP7
SLC2A4	-1.430368988	1.744757141	-5.506839536	1.21E-07	5.41E-07	6.823271322	DOWN	SLC2A4
RP11_1220K2.2	2.417748295	-3.170156378	5.506706854	1.21E-07	5.41E-07	7.093714169	UP	RP11-1220K2.2
TMEM74B	1.139305078	1.459573203	5.5063731	1.22E-07	5.42E-07	6.800732824	UP	TMEM74B
LINC01506	-1.756898723	-3.390849092	-5.506310757	1.22E-07	5.42E-07	7.126534892	DOWN	LINC01506
NCF2	1.017267023	2.638775885	5.505117708	1.22E-07	5.45E-07			

6.656579754 UP NCF2

DSCAML1 1.943272819 -2.195882064 5.502066225 1.24E-07 5.53E-07 7.047625584 UP DSCAML1

PRODH -2.109114412 0.99933676 -5.500134337 1.25E-07 5.57E-07 6.883118421 DOWN PRODH

CTC_281M20.1 -1.297443896 -6.058072086 -5.498877471 1.26E-07 5.61E-07 7.090088313 DOWN CTC-281M20.1

RP11_283I3.4 -2.09189192 -2.573211633 -5.497588253 1.27E-07 5.64E-07 7.072361783 DOWN RP11-283I3.4

FAM35DP -1.377658967 -0.910484212 -5.497147571 1.27E-07 5.65E-07 6.997650503 DOWN FAM35DP

SLC2A9 -1.810220359 2.603016611 -5.496660575 1.28E-07 5.66E-07 6.685263371 DOWN SLC2A9

DGCR6 -1.118738123 2.006519096 -5.491005837 1.31E-07 5.81E-07 6.712976605 DOWN DGCR6

OSBPL10 1.049773954 1.360856872 5.48965139 1.32E-07 5.85E-07 6.734766285 UP OSBPL10

ST8SIA6 -1.472319875 -3.790764642 -5.48800762 1.33E-07 5.90E-07 7.043225005 DOWN ST8SIA6

RP11_756H6.1 -1.445652655 -2.978440726 -5.481874698 1.37E-07 6.07E-07 7.008629863 DOWN RP11-756H6.1

GCSAM 1.300022018 -1.994256273 5.479832861 1.38E-07 6.13E-07 6.945577013 UP GCSAM

RFPL4A -1.578571834 -4.174600499 -5.479420152 1.39E-07 6.14E-07 7.00358831 DOWN RFPL4A

CDH6 1.483594993 2.254129182 5.477875758 1.40E-07 6.18E-07 6.570452866 UP CDH6

ZNF382 1.089766478 0.078295551 5.476982457 1.40E-07 6.21E-07 6.798479372 UP ZNF382

CPEB1 1.750550686 -3.437706274 5.476023869 1.41E-07 6.23E-07 6.967192877 UP CPEB1

CSF3R 1.199684816 1.889105891 5.475028695 1.42E-07 6.26E-07 6.602846882 UP CSF3R

RP1_45I4.3 -1.482140432 -2.064306559 -5.474730848 1.42E-07 6.27E-07 6.952167347 DOWN RP1-45I4.3

ART5 1.804221375 -3.738434329 5.474668263 1.42E-07 6.27E-07 6.966689134 UP ART5

WNT9A 1.793414808 -2.37105958 5.470737241 1.45E-07 6.38E-07 6.910355069 UP WNT9A

PTGES 2.089787888 -0.174770481 5.470352818 1.45E-07 6.39E-07 6.774440999 UP PTGES

MTATP6P2 -1.20791709 -3.881525037 -5.469456838 1.46E-07 6.42E-07 6.956943217 DOWN MTATP6P2

ANKRD36C 1.000034555 -1.32519769 5.467968841 1.47E-07 6.46E-07 6.85849868 UP ANKRD36C

RP11_1008C21.2 1.085820737 -1.72526695 5.4678025 1.47E-07 6.47E-07 6.878784831 UP RP11-1008C21.2

TRPM5 1.367477083 -4.70740754 5.466731531 1.47E-07 6.50E-07 6.94330627 UP TRPM5

LRFN4 1.09707429 0.530722889 5.464474245 1.49E-07 6.57E-07 6.699271393 UP LRFN4

IGFL2 1.375384375 -4.883153543 5.464333296 1.49E-07 6.57E-07 6.932208159 UP IGFL2

RP3_439F8.1 1.363309851 -2.880601529 5.460536677 1.52E-07 6.69E-07 6.885969046 UP RP3-439F8.1

LINC00659 -2.409928066 -1.329906683 -5.457008806 1.55E-07 6.80E-07 6.843025249 DOWN LINC00659

ZNF347 1.059064657 0.842960902 5.456048312 1.55E-07 6.83E-07 6.630891728 UP ZNF347

BCRP2 1.247006785 -5.428717234 5.454732715 1.56E-07 6.87E-07 6.887030691 UP BCRP2

HOXD1 1.746231739 -4.138165651 5.453650095 1.57E-07 6.90E-07 6.877165858 UP HOXD1

CTC_490E21.13 -1.432795593 -5.344470482 -5.453321749 1.57E-07 6.91E-07 6.880387425 DOWN CTC-490E21.13

RP11_2E11.9 1.051793149 -2.90388046 5.452487098 1.58E-07 6.94E-

07 6.853918583 UP RP11-2E11.9

RAP1GAP 1.226338959 4.897232055 5.45179789 1.59E-07 6.96E-07 6.199641859 UP RAP1GAP

LGSN -2.20587764 -0.591965701 -5.45094324 1.59E-07 6.98E-07 6.772178279 DOWN LGSN

RP11_54O7.17 1.363367479 -1.272277264 5.450214167 1.60E-07 7.01E-07 6.768232474 UP RP11-54O7.17

ZBED2 1.735135181 -3.337515738 5.448866145 1.61E-07 7.05E-07 6.838049964 UP ZBED2

TMSB10P1 1.131512086 -4.507189962 5.448827293 1.61E-07 7.05E-07 6.86009633 UP TMSB10P1

PRSS30P 1.28451149 -3.046055204 5.448117592 1.61E-07 7.07E-07 6.833989348 UP PRSS30P

MX2 1.155695227 2.398614365 5.448033337 1.61E-07 7.07E-07 6.415522982 UP MX2

ASS1P5 -1.142175669 -5.220688476 -5.447682259 1.62E-07 7.08E-07 6.854878649 DOWN ASS1P5

FLNC 2.075311353 1.75529684 5.447620975 1.62E-07 7.08E-07 6.479643349 UP FLNC

CTD_2008P7.8 2.029419023 -5.043788019 5.444938195 1.64E-07 7.17E-07 6.840496123 UP CTD-2008P7.8

RP11_126L15.4 1.178222625 -3.251122923 5.443234221 1.65E-07 7.22E-07 6.817533913 UP RP11-126L15.4

LHFPL5 1.195167365 -5.548786082 5.441915235 1.66E-07 7.27E-07 6.827212363 UP LHFPL5

RP11_465B22.8 1.856631915 -3.549596751 5.441080035 1.67E-07 7.30E-07 6.805736546 UP RP11-465B22.8

PITX2 1.994409451 -4.41793491 5.440854439 1.67E-07 7.30E-07 6.818842629 UP PITX2

EPO 2.456761873 -1.747670489 5.439057977 1.69E-07 7.36E-07 6.723571825 UP EPO

TNRC6C_AS1 1.058287633 0.651758237 5.438947529 1.69E-07 7.37E-07 6.568870793 UP TNRC6C-AS1

VIL1 2.167908253 3.823706386 5.433327564 1.73E-07 7.56E-07 6.205579849 UP VIL1
KRTAP5_6 -1.571306801 -2.220848231 -5.431544258 1.75E-07 7.62E-07 6.756680015 DOWN KRTAP5-6
SPINT3 -1.030465877 -6.247926893 -5.428987328 1.77E-07 7.71E-07 6.763563683 DOWN SPINT3
RP11_356M20.1 1.761673306 -2.096132496 5.42778085 1.78E-07 7.76E-07 6.700445373 UP RP11-356M20.1
NAALADL1 1.405488146 0.939784155 5.424704063 1.81E-07 7.87E-07 6.467147545 UP NAALADL1
LINC00622 1.078346359 -2.151455859 5.423517698 1.82E-07 7.91E-07 6.692874105 UP LINC00622
KIAA1324L 1.342667289 0.62985496 5.423473553 1.82E-07 7.91E-07 6.49487033 UP KIAA1324L
KRT8P36 1.061222994 -3.095128716 5.422837236 1.82E-07 7.94E-07 6.721282428 UP KRT8P36
LRRTM2 -1.080985542 -3.250845157 -5.420469206 1.84E-07 8.03E-07 6.726498648 DOWN LRRTM2
HPSE 1.051549536 0.512073909 5.420407927 1.85E-07 8.03E-07 6.496568271 UP HPSE
YWHAEP7 1.716926684 -4.383316191 5.418851955 1.86E-07 8.09E-07 6.718258787 UP YWHAEP7
PIP5KL1 1.202704561 -2.544833097 5.418590492 1.86E-07 8.09E-07 6.682821153UP PIP5KL1
CHRNA5 1.317483659 -2.272397243 5.416894686 1.88E-07 8.16E-07 6.663798898 UP CHRNA5
RP11_320G24.1 1.169574458 -4.448616696 5.41565851 1.89E-07 8.20E-07 6.705931848 UP RP11-320G24.1
SLC7A11 1.795213569 0.902579308 5.415575207 1.89E-07 8.20E-07 6.423140349 UP SLC7A11
RP1_27K12.2 -2.423742131 0.026652236 -5.415222914 1.89E-07 8.22E-07 6.567608717 DOWN RP1-27K12.2
ARSI 1.548988414 -0.949850243 5.414251807 1.90E-07 8.25E-07

6.577375843 UP ARSI

GRAMD1B 1.69670176 1.462518554 5.41411771 1.90E-07 8.26E-07 6.358660815 UP GRAMD1B

NXNL2 1.38138125 -4.430456807 5.41098928 1.93E-07 8.38E-07 6.683645096 UP NXNL2

ITGA3 1.344079831 3.347027922 5.410721565 1.93E-07 8.39E-07 6.130839577 UP ITGA3

RP5_1132H15.3 -1.727932567 -3.115082008 -5.410620423 1.93E-07 8.39E-07 6.678065093 DOWN RP5-1132H15.3

AC005682.6 1.241417219 -1.953565559 5.408401048 1.96E-07 8.48E-07 6.610457526 UP AC005682.6

JAKMIP2 -1.548991147 -0.122641214 -5.408369902 1.96E-07 8.48E-07 6.533386169 DOWN JAKMIP2

TPM3P6 1.184695908 -2.595153911 5.408176028 1.96E-07 8.48E-07 6.63644299 UP TPM3P6

BBOX1_AS1 2.062063815 -3.949599976 5.406497409 1.97E-07 8.55E-07 6.651405969 UP BBOX1-AS1

AQP8 -2.782353388 -1.360438336 -5.406260343 1.98E-07 8.56E-07 6.610205922 DOWN AQP8

MST1R 1.714048329 -0.45814246 5.403894215 2.00E-07 8.65E-07 6.49215503 UP MST1R

AC006037.2 -1.411407211 -4.962684399 -5.403327446 2.00E-07 8.67E-07 6.649523929 DOWN AC006037.2

FOXQ1 2.401719746 0.03711208 5.401768173 2.02E-07 8.73E-07 6.431269049 UP FOXQ1

CTD_2114J12.1 -1.729747911 -0.807381861 -5.401635553 2.02E-07 8.74E-07 6.549990175 DOWN CTD-2114J12.1

SLC34A1 -1.709301409 -1.960662428 -5.399277024 2.04E-07 8.83E-07 6.59808073 DOWN SLC34A1

ZNF404 1.128477815 -0.103360036 5.398897194 2.05E-07 8.85E-07 6.447820664 UP ZNF404

FAR2P4 1.651609077 -5.265272526 5.397594159 2.06E-07 8.90E-07 6.621780528 UP FAR2P4

RP11_369E15.3 -1.033835365 -5.922625908 -5.396693373 2.07E-07
8.94E-07 6.615857238 DOWN RP11-369E15.3

ALOXE3 1.320912743 -5.1528342 5.396564563 2.07E-07 8.94E-07
6.617640517 UP ALOXE3

CTD_2619J13.5 -1.505946579 -0.547569593 -5.396260853 2.07E-07
8.95E-07 6.504962128 DOWN CTD-2619J13.5

NAT8L 1.840763061 -1.791303477 5.394594965 2.09E-07 9.02E-07
6.529691944 UP NAT8L

INHA 2.219945947 -2.361786493 5.391058627 2.13E-07 9.17E-07
6.53062612 UP INHA

VTCN1 2.741597094 -1.90339106 5.38948322 2.14E-07 9.23E-07
6.498651198UP VTCN1

FNDC4 -1.317190892 4.846081972 -5.389315761 2.14E-07 9.23E-07
5.920101831 DOWN FNDC4

DNM1P51 1.138106141 -4.382565913 5.389127477 2.14E-07 9.24E-07 6.583171202 UP DNM1P51

RP11_478K15.6 -1.554518669 -3.923249693 -5.387368076 2.16E-07
9.31E-07 6.576039869 DOWN RP11-478K15.6

HCG14 -1.330575505 -3.843502677 -5.38544708 2.18E-07 9.40E-07
6.567165315 DOWN HCG14

TREH -2.013460923 0.070907577 -5.385238512 2.19E-07 9.41E-07
6.418954837 DOWN TREH

ACKR1 -2.044682104 -0.664699109 -5.385026644 2.19E-07 9.41E-07
6.468644123 DOWN ACKR1

GUCA2A 2.200368225 -3.594750157 5.377271079 2.27E-07 9.75E-07 6.505606832 UP GUCA2A

CTB_36O1.4 -1.234720944 -4.318355122 -5.377258404 2.27E-07
9.75E-07 6.529968806 DOWN CTB-36O1.4

PLIN4 -1.310004096 4.6323963 -5.376013753 2.28E-07 9.81E-07
5.871499075 DOWN PLIN4

SERPINA1 -1.212304508 13.69468689 -5.374040831 2.31E-07 9.90E-07 5.970111849DOWN SERPINA1

MIR663AHG 1.842713815 -5.206755218 5.373260685 2.31E-07

9.93E-07 6.509268441 UP MIR663AHG
RP11_317N12.1 1.899308898 -5.151920213 5.372650314 2.32E-07
9.96E-07 6.506366313 UP RP11-317N12.1
ZNF556 1.456577603 -3.909553995 5.370518941 2.35E-07 1.01E-06
6.491530606 UP ZNF556
TMPRSS2 -1.301607768 4.526953293 -5.367785593 2.38E-07 1.02E-
06 5.843438734 DOWN TMPRSS2
AC007191.4 1.140175812 -0.919342337 5.366189848 2.39E-07 1.03E-
06 6.357066697 UP AC007191.4
ADAM28 1.419302083 0.216873951 5.36602763 2.40E-07 1.03E-06
6.264668184 UP ADAM28
FOXL1 1.3775598 -1.315238822 5.366015485 2.40E-07 1.03E-06
6.379211409UP FOXL1
GLB1L2 1.855181472 -1.632323987 5.365960721 2.40E-07 1.03E-
06 6.390003569 UP GLB1L2
SNORD17 1.117603378-3.155204474 5.364095717 2.42E-07 1.04E-06
6.450176599 UP SNORD17
MMP16 1.622626151 -1.826076968 5.363898283 2.42E-07 1.04E-06
6.39474286 UP MMP16
ZNF578 1.262802972 -3.691388097 5.361906939 2.44E-07 1.05E-06
6.450390569 UP ZNF578
RSPH1 1.658261604 -1.815926301 5.361329519 2.45E-07 1.05E-06
6.381566168 UP RSPH1
PNMAL1 2.135424073 -1.764074203 5.35877158 2.48E-07 1.06E-06
6.358962657 UP PNMAL1
SYT1 2.031299785 -0.544995281 5.355726249 2.52E-07 1.08E-06
6.271060597 UP SYT1
CNGA1 -1.885580384 2.017475558 -5.355224586 2.52E-07 1.08E-06
6.095883222 DOWN CNGA1
CTD_2366F13.2 -1.039019961 -2.429867224 -5.354859629 2.53E-07
1.08E-06 6.408131012 DOWN CTD-2366F13.2
CAPN3 -1.143716443 -0.774471321 -5.354063571 2.54E-07 1.08E-06
6.320565068 DOWN CAPN3

GAD1 2.08476254 -2.405912983 5.353315247 2.55E-07 1.09E-06 6.361947064 UP GAD1

PWAR6 1.510357098 0.776024649 5.353313521 2.55E-07 1.09E-06 6.150682869 UP PWAR6

PSORS1C1 1.566998337 -1.810092349 5.352131741 2.56E-07 1.09E-06 6.340341465 UP PSORS1C1

USP2_AS1 -1.490350771 -0.453921682 -5.351953827 2.56E-07 1.09E-06 6.294205368 DOWN USP2-AS1

S100A4 1.073729183 3.660600856 5.351498706 2.57E-07 1.09E-06 5.820676897 UP S100A4

RP11_263G22.1 -1.930349133 -3.18040184 -5.350946536 2.58E-07 1.10E-06 6.403013894 DOWN RP11-263G22.1

TNFRSF13C 1.254497312 -1.699164891 5.349382819 2.60E-07 1.10E-06 6.325616551 UP TNFRSF13C

PAGE4 -3.922845166 -1.894646 -5.346891977 2.63E-07 1.12E-06 6.346715826 DOWN PAGE4

MAGEE1 1.129163157 -0.46868799 5.34685905 2.63E-07 1.12E-06 6.235965082 UP MAGEE1

AC012668.2 -1.504684335 -3.250243768 -5.346067739 2.64E-07 1.12E-06 6.382743193 DOWN AC012668.2

BACE2 1.593683838 3.473594078 5.343923065 2.66E-07 1.13E-06 5.809351242 UP BACE2

LMX1B 2.173504945 -3.817381918 5.343144685 2.67E-07 1.14E-06 6.355004088 UP LMX1B

RP11_395B7.4 1.45890421 -4.152372478 5.34123596 2.70E-07 1.15E-06 6.35993051 UP RP11-395B7.4

SYT5 1.485634118 -3.299679836 5.341049302 2.70E-07 1.15E-06 6.342366399 UP SYT5

TMEM145 1.703109309 -1.653843107 5.340896403 2.70E-07 1.15E-06 6.277808264 UP TMEM145

DUSP15 1.442406552 -1.798811623 5.339609108 2.72E-07 1.15E-06 6.283342542 UP DUSP15

SNORD99 1.048233853 -3.210494197 5.339519533 2.72E-07 1.15E-

06 6.339444766 UP SNORD99

AC079776.2 -1.128800909 -5.181481057 -5.339308056 2.72E-07 1.15E-06 6.354614045 DOWN AC079776.2

RP11_428O18.6 -1.280535983 -2.635261068 -5.338590801 2.73E-07 1.16E-06 6.339346121 DOWN RP11-428O18.6

HTR1D 2.203637605 -2.399299943 5.338506341 2.73E-07 1.16E-06 6.290057432 UP HTR1D

CLIP2 1.102737716 3.566975525 5.338058151 2.74E-07 1.16E-06 5.768067166 UP CLIP2

GNG4 2.879407107 -0.638017529 5.337304991 2.75E-07 1.16E-06 6.179233691 UP GNG4

VPS37D -1.019243282 1.856776632 -5.336050237 2.77E-07 1.17E-06 6.003851641 DOWN VPS37D

PPL -1.151233203 3.989798602 -5.335994364 2.77E-07 1.17E-06 5.741407289 DOWN PPL

GS1_600G8.5 1.416463473 -5.463954507 5.335572675 2.77E-07 1.17E-06 6.336448384 UP GS1-600G8.5

C19orf33 1.726532403 -1.563867662 5.335236938 2.78E-07 1.17E-06 6.247213324 UP C19orf33

TLR10 1.514944234 -2.068282579 5.334955104 2.78E-07 1.18E-06 6.274053435 UP TLR10

CLECL1 1.240217639 -2.523193326 5.33428861 2.79E-07 1.18E-06 6.292173572 UP CLECL1

ZNF571_AS1 1.141161412 -2.809051692 5.334180394 2.79E-07 1.18E-06 6.302772868 UP ZNF571-AS1

RP11_559N14.5 1.071388865 -4.979615749 5.334022055 2.79E-07 1.18E-06 6.330488516 UP RP11-559N14.5

KCNQ3 1.228013422 -1.84067436 5.332974616 2.81E-07 1.19E-06 6.258524436 UP KCNQ3

HERC6 -1.037484159 2.516682701 -5.331971502 2.82E-07 1.19E-06 5.906083155 DOWN HERC6

SH2D2A 1.13422932 0.806281567 5.331939818 2.82E-07 1.19E-06 6.055221902 UP SH2D2A

MT_TC -1.197568052 -2.111219382 -5.327556104 2.88E-07 1.22E-06 6.271794747 DOWN MT-TC
IL1A 1.048510865 -4.986256844 5.325608329 2.91E-07 1.23E-06 6.291999766 UP IL1A
BCHE -2.04758567 3.873168007 -5.325100311 2.91E-07 1.23E-06 5.742148843 DOWN BCHE
LINC01482 -1.401721662 -2.425190121 -5.324122146 2.93E-07 1.23E-06 6.267542527 DOWN LINC01482
ZNF521 -1.079146937 0.500286339 -5.32305362 2.94E-07 1.24E-06 6.082923468 DOWN ZNF521
PLA2G4E 1.349267931 -4.380298023 5.321134204 2.97E-07 1.25E-06 6.270164178 UP PLA2G4E
CEACAM7 2.619862302 -4.427010689 5.320129294 2.98E-07 1.26E-06 6.256287011 UP CEACAM7
RP11_286N22.16 -1.691488548 -3.737713348 -5.319761694 2.99E-07 1.26E-06 6.264959811 DOWN RP11-286N22.16
OCA2 2.523557779 -2.246790875 5.315500186 3.05E-07 1.28E-06 6.172854272 UP OCA2
ACOXL 1.152742309 -3.758242198 5.314719672 3.06E-07 1.29E-06 6.236112324 UP ACOXL
GPR98 -1.432151095 3.130928233 -5.313938271 3.07E-07 1.29E-06 5.753262254 DOWN GPR98
NAALAD2 1.069937513 -0.875556853 5.313247632 3.08E-07 1.30E-06 6.111871614 UP NAALAD2
RP4_806M20.4 1.32817286 -4.991609674 5.313185648 3.08E-07 1.30E-06 6.234807419 UP RP4-806M20.4
APOM -1.646334567 7.129551788 -5.313173657 3.08E-07 1.30E-06 5.517546201 DOWN APOM
RP11_739N20.2 1.190539437 -2.503733307 5.311047425 3.12E-07 1.31E-06 6.186644364 UP RP11-739N20.2
CRACR2B 1.109772858 1.948572592 5.31022476 3.13E-07 1.31E-06 5.829569811 UP CRACR2B
HTR2B -1.68957529 -0.071447627 -5.309246147 3.14E-07 1.32E-06

6.073787845 DOWN HTR2B
ZNF280A 1.242375784 -5.621530216 5.309046151 3.15E-07 1.32E-06 6.214979522 UP ZNF280A
LINC00398 -1.398750957 -4.274831056 -5.305089417 3.21E-07 1.34E-06 6.199007233 DOWN LINC00398
NPHP4 1.154233585 1.55145929 5.303877969 3.22E-07 1.35E-06 5.846742702 UP NPHP4
ZIK1 1.184611115 -0.443530308 5.300308068 3.28E-07 1.37E-06 6.019883381 UP ZIK1
KLHL38 1.286276137 -4.15550144 5.299628158 3.29E-07 1.38E-06 6.170850336 UP KLHL38
RP11_465O11.1 1.158920216 -5.550503706 5.298930407 3.30E-07 1.38E-06 6.169075598 UP RP11-465O11.1
IL21R 1.331952017 -0.889973395 5.295219666 3.36E-07 1.40E-06 6.026516551 UP IL21R
POPDC3 2.305954046 -4.057519089 5.295193612 3.36E-07 1.40E-06 6.138922213 UP POPDC3
SCTR 2.177055742 -0.176256272 5.29045663 3.44E-07 1.43E-06 5.939867686 UP SCTR
NR4A1 -1.241098581 3.812660016 -5.288643992 3.47E-07 1.44E-06 5.544899165 DOWN NR4A1
RP11_513G11.2 -1.462456221 -4.8420728 -5.287955612 3.48E-07 1.45E-06 6.120449783 DOWN RP11-513G11.2
MME -3.551753231 2.057053205 -5.286368895 3.50E-07 1.46E-06 5.822926601 DOWN MME
CTD_2034I21.2 -2.322166167 -1.64783627 -5.285534105 3.52E-07 1.46E-06 6.065188725 DOWN CTD-2034I21.2
MESP2 1.169376584 -0.918613014 5.28538048 3.52E-07 1.46E-06 5.985675158 UP MESP2
BOC -1.174842084 0.241660171 -5.284157919 3.54E-07 1.47E-06 5.927114975DOWN BOC
ZFHX4 -1.244130365 2.713182263 -5.282199962 3.57E-07 1.48E-06 5.655007083 DOWN ZFHX4

LGALS2 1.336298533 0.462065061 5.28035881 3.60E-07 1.50E-06 5.847678585 UP LGALS2

SCIN 1.556754759 -1.595323824 5.280209778 3.61E-07 1.50E-06 6.000019731 UP SCIN

RP11_563J2.2 1.434278799 -3.286587111 5.279863475 3.61E-07 1.50E-06 6.064276358 UP RP11-563J2.2

UROC1 -3.147180823 2.73314051 -5.279069281 3.63E-07 1.50E-06 5.702387332 DOWN UROC1

CCT8P1 -1.062957964 3.367845583 -5.277928278 3.65E-07 1.51E-06 5.541956443 DOWN CCT8P1

PFKFB3 1.27901447 4.256581347 5.277738847 3.65E-07 1.51E-06 5.429659945 UP PFKFB3

DHH -1.346013831 -2.955525442 -5.274963203 3.70E-07 1.53E-06 6.054878472 DOWN DHH

MTRNR2L12 -1.076949245 -0.039514548 -5.274067652 3.71E-07 1.54E-06 5.901103311 DOWN MTRNR2L12

AL132988.1 -1.333512788 -1.06854562 -5.272406272 3.74E-07 1.55E-06 5.9673069 DOWN AL132988.1

AC000032.2 -1.428176656 -5.391126047 -5.271132296 3.77E-07 1.56E-06 6.042549557 DOWN AC000032.2

PMS2P10 -1.135818687 -3.228307626 -5.269892106 3.79E-07 1.57E-06 6.035465713 DOWN PMS2P10

SFN 2.089165879 2.535146251 5.269187263 3.80E-07 1.57E-06 5.567904604 UP SFN

TMC5 2.495200571 0.24425254 5.263605456 3.90E-07 1.61E-06 5.774782617 UP TMC5

SMARCE1P6 1.140199753 -4.887173487 5.262170879 3.93E-07 1.62E-06 6.002977469 UP SMARCE1P6

LINC00940 -1.521229223 -4.613195196 -5.260850991 3.95E-07 1.63E-06 5.997587339 DOWN LINC00940

ITGA2 1.265666987 2.408912295 5.260294335 3.96E-07 1.64E-06 5.542649246 UP ITGA2

CXCL1 2.410536899 0.469199445 5.260184554 3.97E-07 1.64E-06

5.741984072 UP CXCL1

SLC6A19 2.724123117-3.046979942 5.259973423 3.97E-07 1.64E-06 5.942765491 UP SLC6A19

AP006285.7 -1.64023484 -4.060515226 -5.258943429 3.99E-07 1.64E-06 5.988851884 DOWN AP006285.7

EFCAB12 -1.507313065 1.02797794 -5.258222029 4.00E-07 1.65E-06 5.745667045 DOWN EFCAB12

CTD_2337A12.1-1.313443383 -3.794070978 -5.258082678 4.01E-07 1.65E-06 5.984698087 DOWN CTD-2337A12.1

NOTUM -2.966721166 3.222856732 -5.257980276 4.01E-07 1.65E-06 5.543422163 DOWN NOTUM

RP13_452N2.1 -1.300708949 -3.622429744 -5.255845285 4.05E-07 1.67E-06 5.974028976 DOWN RP13-452N2.1

GLYATL1P4-2.062362903 -3.743527755 -5.255832525 4.05E-07 1.67E-06 5.973439187 DOWN GLYATL1P4

MT1JP -1.773429535 -4.911303925 -5.254854727 4.07E-07 1.67E-06 5.96958082 DOWN MT1JP

CADM2 1.985651179-3.765725575 5.253213991 4.10E-07 1.69E-06 5.946719014 UP CADM2

DOK5 -1.087754349 -0.386150321 -5.251142217 4.14E-07 1.70E-06 5.82240963 DOWN DOK5

ITLN2 -2.040400564 -2.072913249 -5.247766271 4.21E-07 1.73E-06 5.908583175 DOWN ITLN2

CSF2RA 1.028703362 1.00622912 5.246561233 4.23E-07 1.74E-06 5.646006818 UP CSF2RA

PPP2R2B 1.295631974 -1.373998894 5.246497581 4.23E-07 1.74E-06 5.836247046 UP PPP2R2B

CCL23 -1.100416655 -2.447513139 -5.242469516 4.31E-07 1.77E-06 5.896207668 DOWN CCL23

LINC01277 -1.086938171 -2.16916277 -5.242273987 4.32E-07 1.77E-06 5.885005879 DOWN LINC01277

WI2_89031B12.1 1.076454541 -5.075303578 5.241965174 4.32E-07 1.77E-06 5.91140719 UP WI2-89031B12.1

C1orf233 1.28462381 0.473644185 5.240146872 4.36E-07 1.79E-06 5.664515973 UP C1orf233

FAM27E3 1.102346687 -4.039626606 5.238919044 4.38E-07 1.80E-06 5.895169381 UP FAM27E3

RP11_492E3.2 2.108667201 -2.425790097 5.238845637 4.39E-07 1.80E-06 5.836733962 UP RP11-492E3.2

TFF1 2.392907663 -3.976716213 5.23832636 4.40E-07 1.80E-06 5.87815087 UP TFF1

SNPH 1.054452342 -0.337381074 5.238192512 4.40E-07 1.80E-06 5.729844315 UP SNPH

RP5_1092A11.5 -1.693960449 -4.204691915 -5.237968167 4.40E-07 1.80E-06 5.893973521 DOWN RP5-1092A11.5

C1orf111 -1.686365287 -3.836906679 -5.236209212 4.44E-07 1.82E-06 5.885524308 DOWN C1orf111

KCTD9P4 -1.375413869 -4.664235565 -5.235562329 4.45E-07 1.82E-06 5.883215504 DOWN KCTD9P4

NPPB 1.554203846 -4.83183574 5.23411952 4.48E-07 1.83E-06 5.875227119 UP NPPB

AC005083.1 1.481275632 -3.219578151 5.233812376 4.49E-07 1.84E-06 5.852619525 UP AC005083.1

DBNDD1 -1.17509955 4.324009712 -5.230300675 4.57E-07 1.87E-06 5.221581976 DOWN DBNDD1

AC010980.2 1.42809012 -4.737287598 5.230087449 4.57E-07 1.87E-06 5.857231948 UP AC010980.2

CTD_2523D13.2 1.138168472 -4.155946073 5.229462589 4.58E-07 1.87E-06 5.853222776 UP CTD-2523D13.2

ABCC4 1.44261821 2.524141048 5.229013916 4.59E-07 1.88E-06 5.38527378 UP ABCC4

NAALADL2 -1.378959535 0.929668767 -5.228331379 4.61E-07 1.88E-06 5.616642127 DOWN NAALADL2

AC007128.1 1.400322943 -5.303055758 5.227056977 4.64E-07 1.89E-06 5.843299735 UP AC007128.1

CHI3L2 1.505964709 -1.667971431 5.224262825 4.70E-07 1.92E-06

5.749672411UP CHI3L2

FAM124B -1.075647309 0.075962645 -5.224197026 4.70E-07 1.92E-06 5.66567509 DOWN FAM124B

WIF1 2.105028345 -4.586066084 5.220237393 4.79E-07 1.95E-06 5.809493969 UP WIF1

MTRNR2L10 -1.140390837 -3.473103862 -5.219627086 4.80E-07 1.95E-06 5.80977253 DOWN MTRNR2L10

MAPK15 1.715151441 -2.516332484 5.21844867 4.83E-07 1.96E-06 5.756552545 UP MAPK15

PLA2G5 -1.08638723 0.629482178 -5.218148393 4.84E-07 1.97E-06 5.592255512 DOWN PLA2G5

RP11_757A13.1 -1.640761319 -4.528430256 -5.218098392 4.84E-07 1.97E-06 5.804277886 DOWN RP11-757A13.1

HLA_DQB2 1.513181202 1.613525395 5.215070424 4.91E-07 1.99E-06 5.427833672 UP HLA-DQB2

DUXAP8 1.50243852 -0.742605074 5.213319077 4.95E-07 2.01E-06 5.641356468 UP DUXAP8

CRB2 1.272327505 -3.476131999 5.2115159244.99E-07 2.02E-06 5.761331704 UP CRB2

GLRB 2.150887984 -1.235472966 5.210596626 5.01E-07 2.03E-06 5.6551559 UP GLRB

RP11_61I13.3 -1.057002661 -1.081518154 -5.210065468 5.02E-07 2.04E-06 5.681784353 DOWN RP11-61I13.3

TAF4B 1.275684453 -1.162662767 5.209439874 5.04E-07 2.04E-06 5.655581669 UP TAF4B

CTD_2527I21.14 1.171463338 -3.458947185 5.207177672 5.09E-07 2.06E-06 5.742801079 UP CTD-2527I21.14

PKD1L3 -1.046606179 -2.715928009 -5.207148505 5.09E-07 2.06E-06 5.744023056 DOWN PKD1L3

ARTN 1.074085258 -2.036850433 5.206902495 5.10E-07 2.06E-06 5.695769216 UP ARTN

SLC52A1 1.10889289 -3.616814442 5.205951704 5.12E-07 2.07E-06 5.740862972 UP SLC52A1

GAP43 1.530724977 -3.658898252 5.204733226 5.15E-07 2.08E-06
5.731636529 UP GAP43
AQP7 -1.11899144 2.831960769 -5.203764656 5.17E-07 2.09E-06
5.27560303 DOWN AQP7
RP11_1103G16.1 1.785279133 -5.232948509 5.202969722 5.19E-07
2.10E-06 5.734276119UP RP11-1103G16.1
RP11_345M22.2 -1.322867332 -2.026901662 -5.202876276 5.19E-07
2.10E-06 5.702524959 DOWN RP11-345M22.2
RP5_965G21.4 1.105177357 -2.929348592 5.202215994 5.21E-07
2.10E-06 5.707562644 UP RP5-965G21.4
RP11_432J24.5 1.4695995 -4.658998268 5.201063015 5.24E-07 2.12E-06 5.726240462 UP RP11-432J24.5
MT1P3 -1.423984527 -5.360269288 -5.200912581 5.24E-07 2.12E-06
5.72537359 DOWN MT1P3
PLEKHG4B 1.920017841 -2.444762034 5.200745416 5.25E-07 2.12E-06 5.670743402 UP PLEKHG4B
UBD 1.666310516 4.805811645 5.199093233 5.29E-07 2.13E-06
5.040614075 UP UBD
MATN3 1.925056625 -1.065696458 5.198835455 5.29E-07 2.13E-06
5.593254447 UP MATN3
CTD_2008L17.1 1.39285621 -5.594089338 5.198357157 5.31E-07 2.14E-06 5.713502177 UP CTD-2008L17.1
AC018641.7 1.409944808 -5.327424649 5.196103614 5.36E-07 2.16E-06 5.703868425 UP AC018641.7
RP11_560A15.3 -1.099420483 -5.913077255 -5.196092826 5.36E-07
2.16E-06 5.701693809 DOWN RP11-560A15.3
MT1E -2.299000305 4.706742738 -5.195667861 5.37E-07 2.16E-06
5.069976072 DOWN MT1E
CACNA1F 1.13483445 -3.369477485 5.194561334 5.40E-07 2.17E-06
5.684178434 UP CACNA1F
RP1_81D8.3 -1.202410367 -5.003308734 -5.193883562 5.42E-07 2.18E-06 5.695081546 DOWN RP1-81D8.3
PLCH1 1.441916265 -1.731594854 5.189756963 5.52E-07 2.22E-06

5.598773723 UP PLCH1

CCL15 -1.508038503 3.372966559 -5.188552306 5.56E-07 2.23E-06

5.147152654 DOWN CCL15

MIR126-1.103224839 -4.001643529 -5.187599568 5.58E-07 2.24E-06

5.667306289 DOWN MIR126

HS3ST51.484298964 -5.37926787 5.185524294 5.63E-07 2.26E-06

5.656189138 UP HS3ST5

GCK -2.842940937 -1.658816123 -5.184185228 5.67E-07 2.28E-06

5.607330885 DOWN GCK

C1orf204 1.242712325 -2.622426293 5.183960758 5.68E-07 2.28E-

06 5.614058891 UP C1orf204

MACC11.754337253 -1.867978131 5.183520594 5.69E-07 2.28E-06

5.572975936 UP MACC1

AIM1 -1.049549462 3.196005118-5.183406163 5.69E-07 2.28E-06

5.13370607 DOWN AIM1

ANKRD36B1.098028902 -2.246096703 5.181418851 5.74E-07 2.31E-

06 5.590766756 UP ANKRD36B

ACVR1C -1.188929702 -0.443486089 -5.178069964 5.83E-07 2.34E-

06 5.497463823 DOWN ACVR1C

SSTR5 2.478397515 -3.929529716 5.17786931 5.84E-07 2.34E-06

5.603120452 UP SSTR5

ASS1P11 -1.350318324 -4.121326697 -5.177073448 5.86E-07 2.35E-

06 5.620147261 DOWN ASS1P11

KRT18P59 -1.057457487 -3.647210637 -5.175307743 5.91E-07 2.37E-

06 5.611382441DOWN KRT18P59

KCNMA1 -1.192981752 2.389859001 -5.173714802 5.95E-07 2.38E-

06 5.200886546 DOWN KCNMA1

RP11_475J5.5 -1.115383393 -3.356003966 -5.173093926 5.97E-07

2.39E-06 5.600004843 DOWN RP11-475J5.5

PRAMEF17 -1.121902534 -5.883023659 -5.172595405 5.99E-07 2.39E-

06 5.596419901 DOWN PRAMEF17

KIF26B 1.691662706 0.707427279 5.172342703 5.99E-07 2.39E-06

5.329142578 UP KIF26B

FUT7 1.29246991 -2.707119665 5.171269499 6.02E-07 2.41E-06 5.557914949 UP FUT7

TRIM67 1.347353604 -2.036021484 5.171224179 6.02E-07 2.41E-06 5.531672666 UP TRIM67

RP4_789D17.5 1.014732689 -3.815879786 5.167442675 6.13E-07 2.45E-06 5.571911285UP RP4-789D17.5

POU5F1B -1.152563207 -2.972057532 -5.166046508 6.17E-07 2.46E-06 5.564315276 DOWN POU5F1B

CLEC4M -2.154161617 -3.946927705 -5.165751302 6.18E-07 2.47E-06 5.568351943 DOWN CLEC4M

TMEM191B1.22607499 -3.721806262 5.164622733 6.21E-07 2.48E-06 5.555959203 UP TMEM191B

PRAMEF4 -2.112833803 -4.38346619-5.162596319 6.27E-07 2.50E-06 5.554628534 DOWN PRAMEF4

RUFY4 1.283584346 -2.25630893 5.161813104 6.30E-07 2.51E-06 5.499713681 UP RUFY4

FAM218A 1.193920904 -4.602218681 5.160356242 6.34E-07 2.52E-06 5.544308911UP FAM218A

RP11_142C4.6 -1.269484338 -1.822923058 -5.158923601 6.38E-07 2.54E-06 5.495679615 DOWN RP11-142C4.6

RP11_754N21.1 -1.302290419 -5.150780481 -5.158879921 6.38E-07 2.54E-06 5.537773069 DOWN RP11-754N21.1

PRSS35 1.295763973 -1.542720696 5.158723822 6.39E-07 2.54E-06 5.450558606 UP PRSS35

PPP1R14D 1.918209813 -3.07344204 5.15851135 6.39E-07 2.54E-06 5.501895435 UP PPP1R14D

PACRG_AS1 -1.335113318 -3.990240932 -5.158392021 6.40E-07 2.54E-06 5.536409237 DOWN PACRG-AS1

PTCHD2 1.386327226 -3.792461816 5.158178406 6.40E-07 2.55E-06 5.526785321 UP PTCHD2

ANKRD20A5P 1.261216853 -4.713177866 5.15793634 6.41E-07 2.55E-06 5.533494915 UP ANKRD20A5P

COL28A1 1.885951646 -3.119033664 5.15403916 6.53E-07 2.59E-06

5.484670392 UP COL28A1

MYO3A 2.023208136 -4.821098352 5.153674576 6.54E-07 2.59E-06 5.512454782 UP MYO3A

GAL3ST4 1.095272076 0.595722087 5.152474123 6.58E-07 2.61E-06 5.260586725 UP GAL3ST4

B3GNT3 2.445375974 1.596177677 5.151073798 6.62E-07 2.62E-06 5.130093846 UP B3GNT3

HNRNPA1P21 1.242458879 -2.509661242 5.150828546 6.63E-07 2.63E-06 5.46105352 UP HNRNPA1P21

C11orf70 1.163270998 -3.754024797 5.147187563 6.74E-07 2.67E-06 5.479488434 UP C11orf70

LINC01330 -1.297147656 -2.930478297 -5.14421881 6.83E-07 2.70E-06 5.465877246 DOWN LINC01330

MTMR7 1.207432037 0.426355197 5.14280803 6.88E-07 2.72E-06 5.229930807 UP MTMR7

GAS7 1.0728512 2.100542961 5.142594048 6.89E-07 2.72E-06 5.048336871 UP GAS7

AC090954.5 1.10635151 -5.688692551 5.14191766 6.91E-07 2.73E-06 5.460867428 UP AC090954.5

BMP7 2.30752361 -3.651138205 5.137564001 7.05E-07 2.78E-06 5.418416613 UP BMP7

TMEM30B -1.237551461 3.267032359 -5.134766603 7.14E-07 2.82E-06 4.911632258DOWN TMEM30B

HOXC10 2.376388227 -4.255770371 5.134134415 7.16E-07 2.82E-06 5.416035873 UP HOXC10

GPX7 1.200395971 1.720937584 5.130880136 7.27E-07 2.87E-06 5.039171623 UP GPX7

RP11_79E3.3 -1.550653929 -4.50876271 -5.129531033 7.32E-07 2.88E-06 5.407742181 DOWN RP11-79E3.3

SLC6A15 2.044852542 -5.051027266 5.127550954 7.38E-07 2.91E-06 5.396342442 UP SLC6A15

KIRREL3 1.270199652 -2.970710004 5.1275411787.38E-07 2.91E-06 5.371532503 UP KIRREL3

CD5L -2.585055372 1.171669507 -5.127189502 7.40E-07 2.91E-06
5.160425404 DOWN CD5L
GDF2 -2.339490337 -4.290448977 -5.126616862 7.42E-07 2.92E-06
5.393829142 DOWN GDF2
RP11_249C24.11 -1.452202191 -5.501666975 -5.126271159 7.43E-07
2.92E-06 5.390971703 DOWN RP11-249C24.11
KLK1 1.328083223 -4.693265422 5.125794122 7.44E-07 2.93E-06
5.390029241 UP KLK1
LY6H 1.450377881 -1.859651487 5.122448619 7.56E-07 2.97E-06
5.303035324 UP LY6H
APCDD1L 1.510367344 -4.717355983 5.122049924 7.58E-07 2.98E-
06 5.372984097 UP APCDD1L
RP11_982M15.6 1.117007664-5.154334074 5.121507747 7.59E-07 2.98E-
06 5.37115533 UP RP11-982M15.6
GJB3 1.847555072 -2.625704635 5.121491073 7.59E-07 2.98E-06
5.323670133 UP GJB3
ALOX12B 1.200989318 -5.319633831 5.121385247 7.60E-07 2.98E-
06 5.370218874 UP ALOX12B
PTPRO 1.017452346 -0.897899269 5.120284239 7.64E-07 3.00E-06
5.240888848 UP PTPRO
SLC34A3 1.223808865 -3.898044879 5.1198685287.65E-07 3.00E-06
5.359174884 UP SLC34A3
FGF21 -2.670018027 2.955978394 -5.119637955 7.66E-07 3.01E-06
4.939995624 DOWN FGF21
MEGF11 1.122796761 -3.774028122 5.1195049787.67E-07 3.01E-06
5.356677452 UP MEGF11
PRKG1_AS1 1.222393208 -5.159295981 5.1176441227.73E-07 3.03E-
06 5.353840889 UP PRKG1-AS1
CBS -1.640511975 1.556584159 -5.117050112 7.75E-07 3.04E-06
5.056886494 DOWN CBS
KLF5 1.567499491 1.799974697 5.1161766297.78E-07 3.05E-06
4.960818224 UP KLF5
RP11_475J5.4 -1.279165486 -2.550250522 -5.114554212 7.84E-07

3.07E-06 5.325781678 DOWN RP11-475J5.4

GLI1 1.194127747 -0.62246967 5.113914725 7.87E-07 3.08E-06 5.189689784 UP GLI1

MAP3K5 -1.163159032 3.064191437 -5.113340334 7.89E-07 3.09E-06 4.841555717 DOWN MAP3K5

NPR3 -1.438943954 1.963876666 -5.112388608 7.92E-07 3.10E-06 4.986667305 DOWN NPR3

PLCH2 -2.032928339 1.266480949 -5.111835166 7.94E-07 3.11E-06 5.071921955 DOWN PLCH2

ARMCX6 1.103856882 0.923297315 5.111696088 7.95E-07 3.11E-06 5.044573133 UP ARMCX6

CTC_558O2.1 -1.335771157 -3.48811223 -5.109021195 8.05E-07 3.15E-06 5.315000953 DOWN CTC-558O2.1

C3orf14 1.443719844 0.166061223 5.108111312 8.08E-07 3.16E-06 5.095221863 UP C3orf14

TMEM130 1.841039326 -2.533377467 5.107655473 8.10E-07 3.16E-06 5.259516807 UP TMEM130

SNTG1 -2.221803173 -2.84025191 -5.102713013 8.29E-07 3.23E-06 5.275903197 DOWN SNTG1

IGF1R 1.258158102 1.857118255 5.101577999 8.33E-07 3.25E-06 4.891028027 UP IGF1R

RP11_700H6.1 -1.340092499 -2.333643662 -5.100499804 8.37E-07 3.26E-06 5.256865284 DOWN RP11-700H6.1

DUSP4 1.156852137 1.581399592 5.099501523 8.41E-07 3.28E-06 4.916972037 UP DUSP4

GAPDHP23 -1.249019627 -4.929616643 -5.099088268 8.43E-07 3.29E-06 5.2723214 DOWN GAPDHP23

SYTL5 -1.490957629 -0.210237091 -5.09870679 8.44E-07 3.29E-06 5.130000067 DOWN SYTL5

MCEMP1 1.429170409 -4.028980462 5.098366337 8.45E-07 3.30E-06 5.263911343 UP MCEMP1

MIA2 -1.052233751 -0.115513241 -5.097569935 8.49E-07 3.31E-06 5.11084307 DOWN MIA2

RP11_384L8.1 -1.062190924 2.192551686 -5.096521452 8.53E-07 3.32E-06 4.875242619 DOWN RP11-384L8.1

PDE11A -1.651665805 1.423892206 -5.094808107 8.59E-07 3.35E-06 4.972775861 DOWN PDE11A

FGF14_AS2 -1.095167471 0.171254843 -5.093891851 8.63E-07 3.36E-06 5.073253578 DOWN FGF14-AS2

AC005550.3 -2.643720143 -3.327692644 -5.092473397 8.69E-07 3.38E-06 5.235927266 DOWN AC005550.3

TCN1 1.889272303 -4.369389216 5.092437043 8.69E-07 3.38E-06 5.237967997 UP TCN1

IQSEC3 -1.383592353 -1.448774459 -5.092161255 8.70E-07 3.38E-06 5.179403972 DOWN IQSEC3

LDOC1 1.426352786 1.38149961 5.09182997 8.71E-07 3.39E-06 4.900122821 UP LDOC1

RP11_563J2.3 1.304810087 -3.983938302 5.091204578 8.74E-07 3.40E-06 5.232657228 UP RP11-563J2.3

CTC_458A3.1 1.370960195 -4.593693802 5.088046625 8.87E-07 3.45E-06 5.222315414 UP CTC-458A3.1

GRIN2A 2.168210794 -2.486394774 5.084272917 9.02E-07 3.51E-06 5.147675614 UP GRIN2A

CBLN3 -1.072501249 0.602369086 -5.083349595 9.06E-07 3.52E-06 4.989061155DOWN CBLN3

RP11_33A14.1 -1.286400339 -5.290165002 -5.083148879 9.07E-07 3.52E-06 5.200793798 DOWN RP11-33A14.1

LGALS14 1.794435775 -5.64176887 5.0825113679.10E-07 3.53E-06 5.19664531 UP LGALS14

RP11_373D7.1 -1.020137499 -6.115546754 -5.081534919 9.14E-07 3.55E-06 5.190076319 DOWN RP11-373D7.1

FAM225B 1.0829438 -4.784254588 5.080336914 9.19E-07 3.56E-06 5.189043903 UP FAM225B

LINC01234 2.401096062 -3.067574269 5.078394699 9.27E-07 3.60E-06 5.135468904 UP LINC01234

BAMBI 1.199953402 4.506566236 5.076794047 9.34E-07 3.62E-06

4.500541221 UP BAMBI

GABRQ 1.370206337 -2.760250806 5.075517437 9.40E-07 3.64E-06 5.132793525 UP GABRQ

IFI44 -1.117473447 3.50204743 -5.075162042 9.41E-07 3.65E-06 4.611928713DOWN IFI44

CTD_3010D24.31.2605911 -5.481598784 5.07464672 9.43E-07 3.65E-06 5.162797049 UP CTD-3010D24.3

FANK1 1.183899511-2.864393265 5.072436277 9.53E-07 3.69E-06 5.126022696 UP FANK1

RP11_190C22.8 1.001860282 -3.99799394 5.071083919 9.59E-07 3.71E-06 5.145675513 UP RP11-190C22.8

HBB -1.327316038 2.958114253-5.069518651 9.66E-07 3.74E-06 4.660966192 DOWN HBB

FRMD1 1.8826447 -3.171848872 5.068640861 9.70E-07 3.75E-06 5.10506564 UP FRMD1

RP11_625I7.1 -1.046755138 -6.055471422 -5.067466088 9.75E-07 3.77E-06 5.128302269 DOWN RP11-625I7.1

KL -1.126754571 -0.889016801 -5.06717125 9.76E-07 3.77E-06 5.032110406DOWN KL

FGFBP2 -1.172370318 -1.807151912 -5.066503956 9.79E-07 3.78E-06 5.082851653 DOWN FGFBP2

RP5_1059L7.1 1.336432609 -2.438628414 5.066072417 9.81E-07 3.79E-06 5.081126802UP RP5-1059L7.1

GRAMD1C -1.086074897 2.453749993 -5.066010558 9.82E-07 3.79E-06 4.70891244 DOWN GRAMD1C

CTB_50L17.14 -1.301379335 -0.025039377 -5.064061983 9.90E-07 3.83E-06 4.959162562 DOWN CTB-50L17.14

SLC28A3 1.95289232 -3.49236394 5.062074832 1.00E-06 3.86E-06 5.085771503 UP SLC28A3

GALR3 -1.628236555 -3.070636545 -5.060120621 1.01E-06 3.89E-06 5.094207929 DOWN GALR3

MUC12 1.709044343 -2.474734971 5.058765927 1.01E-06 3.91E-06 5.041370839 UP MUC12

PCDHA1 1.770603703 -3.983448785 5.058035233 1.02E-06 3.92E-06 5.081764681 UP PCDHA1
COL8A2 1.4118436980.057716409 5.054950347 1.03E-06 3.97E-06 4.869594334 UP COL8A2
FBN2 1.28750365 -2.349054338 5.05403587 1.04E-06 3.99E-06 5.025151741 UP FBN2
ZNF285 1.223665989 -0.521409473 5.047984486 1.07E-06 4.10E-06 4.889206991 UP ZNF285
NMU 1.237900612 -4.978242046 5.0478211821.07E-06 4.10E-06 5.045435585 UP NMU
FBLL1 1.943344035 -1.612002609 5.047337609 1.07E-06 4.11E-06 4.9483676 UP FBLL1
RP11_71E19.1 -1.237355198 -1.885415856 -5.046087994 1.08E-06 4.13E-06 4.997448762 DOWN RP11-71E19.1
CD7 1.413627426 2.00080621 5.044235876 1.09E-06 4.16E-06 4.615495475 UP CD7
CLGN 1.928166757 1.782170224 5.043907556 1.09E-06 4.17E-06 4.63553906 UP CLGN
RP11_38M8.1 1.627889453 -2.092545726 5.041941458 1.10E-06 4.21E-06 4.954262687 UP RP11-38M8.1
MAP3K15 1.309976062 -3.093942519 5.038831978 1.11E-06 4.26E-06 4.982195778 UP MAP3K15
MMEL1 1.377800955 -2.828466144 5.037621599 1.12E-06 4.28E-06 4.967593921 UP MMEL1
EGFR_AS1 2.331966572 -2.00275124 5.036563007 1.12E-06 4.30E-06 4.912203271 UP EGFR-AS1
AC020571.3 1.399027163 -3.676562321 5.034365888 1.14E-06 4.34E-06 4.975985913 UP AC020571.3
TFF2 2.455321158-3.801165017 5.034241536 1.14E-06 4.35E-06 4.963266 UP TFF2
CTB_43E15.2 -1.238931656 -1.181944338 -5.03389065 1.14E-06 4.35E-06 4.904988047 DOWN CTB-43E15.2
RP11_736N17.8 -1.374930962 -2.94949305 -5.033591263 1.14E-06 4.36E-

06 4.976531034 DOWN RP11-736N17.8

LINC01296 1.510816363 -2.936231584 5.032455904 1.15E-06 4.38E-06 4.946503563 UP LINC01296

CTD_2531D15.41.234570359 -5.032145382 5.0309787 1.15E-06 4.40E-06 4.971437826 UP CTD-2531D15.4

MAL2 1.328819898 5.429166075 5.026040393 1.18E-06 4.50E-06 4.234434178 UP MAL2

AC022816.2-1.711019822 -1.289973251 -5.025749619 1.18E-06 4.51E-06 4.879981723 DOWN AC022816.2

RIPPLY2 1.471344152 -5.582514895 5.025593645 1.18E-06 4.51E-06 4.946590409 UP RIPPLY2

SNAP91 1.507057412 -5.18246586 5.025143802 1.18E-06 4.52E-06 4.945245585 UP SNAP91

C1orf105 -1.235318694 -1.741374041 -5.024332037 1.19E-06 4.53E-06 4.894558359 DOWN C1orf105

VAX2 1.485246608 -2.437256439 5.021458285 1.20E-06 4.59E-06 4.880662848 UP VAX2

DAAM2 -1.152331835 2.363351748 -5.019059992 1.22E-06 4.64E-06 4.512706812 DOWN DAAM2

RP11_255H23.2 1.563149673 -3.012554448 5.016234756 1.23E-06 4.69E-06 4.876785645 UP RP11-255H23.2

AGPAT9 -1.23746659 3.034836454 -5.015599971 1.24E-06 4.71E-06 4.411929108DOWN AGPAT9

RP11_178L8.9 -1.234594416 -3.646073028 -5.014587895 1.24E-06 4.72E-06 4.899732785 DOWN RP11-178L8.9

RP11_675F6.3 1.037856143 -5.80703119 5.0114540621.26E-06 4.79E-06 4.884461507 UP RP11-675F6.3

RAB38 1.231673292 -1.372469447 5.0113075481.26E-06 4.79E-06 4.787629731 UP RAB38

ZNF287 1.06193053 -1.167144487 5.010557305 1.27E-06 4.81E-06 4.772927748 UP ZNF287

CATSPER1 1.001466093 -2.799360814 5.009239958 1.27E-06 4.83E-06 4.848425681 UP CATSPER1

ZNF385C 1.083032637 -0.680934878 5.008593089 1.28E-06 4.85E-06 4.729591225 UP ZNF385C

LHB 1.121843397 -4.897746646 5.007295725 1.29E-06 4.87E-06 4.868022079 UP LHB

VSIG8 1.120592326 -5.059666643 5.005972599 1.29E-06 4.90E-06 4.862107523 UP VSIG8

GNAS_AS1 1.407600526 -4.163182853 5.002926894 1.31E-06 4.96E-06 4.845629602 UP GNAS-AS1

RP11_557H15.4 -1.204388313 -2.591220632 -5.00254877 1.31E-06 4.97E-06 4.833367223 DOWN RP11-557H15.4

ANKS4B -1.693425379 3.713793685 -5.000385913 1.33E-06 5.02E-06 4.271969806 DOWN ANKS4B

FAM163A 1.325277461 -3.483637916 4.999744406 1.33E-06 5.03E-06 4.821175251UP FAM163A

TCL6 1.70810677 -3.784306962 4.998584038 1.34E-06 5.06E-06 4.817715635 UP TCL6

CTD_2529O21.2-1.637667509 -3.475924023 -4.998163824 1.34E-06 5.06E-06 4.826589712 DOWN CTD-2529O21.2

GRPR -2.086682401 -2.773268031 -4.996773179 1.35E-06 5.09E-06 4.809373059 DOWN GRPR

RP11_809N8.5 -1.178422255 -2.686306862 -4.996407327 1.35E-06 5.10E-06 4.808958207 DOWN RP11-809N8.5

CASC20 1.677804068 -5.161403449 4.996163793 1.35E-06 5.10E-06 4.818254391 UP CASC20

RP11_747H7.3 1.566958822 -1.510093247 4.992534666 1.37E-06 5.19E-06 4.709485199 UP RP11-747H7.3

CD207 1.634431682 -2.21242858 4.992053223 1.38E-06 5.20E-06 4.741690152 UP CD207

PDE6B 1.078160627 -0.619466599 4.991997127 1.38E-06 5.20E-06 4.651722178 UP PDE6B

AC013444.1 1.060101778 -5.657745706 4.991325169 1.38E-06 5.21E-06 4.796995428 UP AC013444.1

HMGB2P1 1.086021808 -4.692801147 4.990946502 1.38E-06 5.22E-

06	4.796692764	UP	HMGB2P1

ZNF630	1.157182147	-1.260073315	4.989963595	1.39E-06	5.24E-06	4.687939808	UP	ZNF630

RP11_510N19.5	1.351518702	-2.331684371	4.989717196	1.39E-06	5.24E-06	4.740588703	UP	RP11-510N19.5

PAX9	1.273128602	-4.461108487	4.987008731	1.41E-06	5.31E-06	4.778687727	UP	PAX9

MFSD2B	1.130377603	-1.882715697	4.986569874	1.41E-06	5.31E-06	4.709058591	UP	MFSD2B

PLAGL1	1.112357292	0.767704823	4.985479476	1.42E-06	5.34E-06	4.501922503	UP	PLAGL1

GPER1	-1.136350024	3.470344621	-4.984158626	1.43E-06	5.37E-06	4.208052041	DOWN	GPER1

RP11_314M24.1	1.515302757	-5.550807622	4.984103465	1.43E-06	5.37E-06	4.765349824	UP	RP11-314M24.1

BFSP2	1.417161547	-3.667880985	4.982147347	1.44E-06	5.41E-06	4.747241791	UP	BFSP2

GLYATL1P3	-1.284208876	-5.380003668	-4.980322724	1.45E-06	5.46E-06	4.749468107	DOWN	GLYATL1P3

CRABP2	1.304807684	-0.709886615	4.980217267	1.45E-06	5.46E-06	4.604507649	UP	CRABP2

TRIM22	-1.032931042	3.928670125	-4.97773754	1.47E-06	5.52E-06	4.126159825	DOWN	TRIM22

SSBP2	1.000326354	1.665064604	4.977710544	1.47E-06	5.52E-06	4.370546286	UP	SSBP2

SHISA6	-1.444853616	-4.012584836	-4.976809048	1.48E-06	5.54E-06	4.735701401	DOWN	SHISA6

CASP5	1.124026469	-3.816025942	4.975952212	1.48E-06	5.56E-06	4.726062355	UP	CASP5

TMEM27	-1.630268815	0.872342676	-4.975920339	1.48E-06	5.56E-06	4.501855747	DOWN	TMEM27

HSD3B1	-1.9783361	-3.468265832	-4.973432473	1.50E-06	5.62E-06	4.717940518	DOWN	HSD3B1

RP11_209E8.1 -1.557092927 -2.883797517 -4.972069274 1.51E-06 5.65E-06 4.70610686 DOWN RP11-209E8.1
TMEM63C 1.562552968 -2.71786748 4.971572705 1.51E-06 5.66E-06 4.670848633 UP TMEM63C
CYP4F26P 1.188823516 -5.214796814 4.970316065 1.52E-06 5.69E-06 4.706411261 UP CYP4F26P
LINC00661 1.41098651 -5.212114845 4.970174137 1.52E-06 5.69E-06 4.70548062 UP LINC00661
AC069277.2 1.675425652 -4.867378541 4.969850619 1.52E-06 5.70E-06 4.703729951 UP AC069277.2
CHADL -1.24375971 1.558382962 -4.969677885 1.53E-06 5.71E-06 4.394180849 DOWN CHADL
TSSK5P 1.162591677 -2.798263845 4.969247785 1.53E-06 5.72E-06 4.671994302 UP TSSK5P
APOBEC3D 1.020125432 0.308848829 4.968135131 1.54E-06 5.74E-06 4.470300941 UP APOBEC3D
RP11_141M1.3 -1.00497179 -1.742360495 -4.967058635 1.54E-06 5.77E-06 4.641940415 DOWN RP11-141M1.3
PCYT1B 1.592714596 -3.503117899 4.96519808 1.56E-06 5.81E-06 4.667624109 UP PCYT1B
PI3 2.244610869 -1.361296566 4.961351994 1.58E-06 5.90E-06 4.555496787 UP PI3
AGR2 2.972331025 -1.745754733 4.960719216 1.59E-06 5.92E-06 4.558921214 UP AGR2
PGC 3.313459443 -1.145718209 4.960647605 1.59E-06 5.92E-06 4.519473256 UP PGC
FREM1 1.949764009 -2.282929813 4.958251735 1.61E-06 5.98E-06 4.591514634 UP FREM1
HIST1H4J -1.184488574 -2.795896539 -4.957353856 1.61E-06 6.00E-06 4.641455319 DOWN HIST1H4J
SPEF1 1.25066719 -3.197303168 4.952850529 1.65E-06 6.12E-06 4.611032981 UP SPEF1
GABRA3 2.101827589 -4.525401362 4.952559941 1.65E-06 6.13E-

06 4.625355782 UP GABRA3
HORMAD2_AS1 -2.849054284 -0.056256117 -4.951798023 1.65E-06
6.14E-06 4.492329201 DOWN HORMAD2-AS1
FGL1 -1.776012076 8.953981265 -4.950576807 1.66E-06 6.18E-06
3.910034277 DOWN FGL1
EYA4 2.04646865 -4.156266779 4.950140192 1.67E-06 6.19E-06
4.61045327 UP EYA4
ADAMTSL1 -1.144001124 0.815357731 -4.950110442 1.67E-06
6.19E-06 4.384369766 DOWN ADAMTSL1
GYLTL1B 1.656599352 1.1148436364.950034513 1.67E-06 6.19E-06
4.29945768 UP GYLTL1B
UGT1A9 -2.48374658 2.795763712 -4.944267853 1.71E-06 6.35E-06
4.176692621 DOWN UGT1A9
GNG5P2 -1.027474039 -4.783124553 -4.943635028 1.72E-06 6.36E-
06 4.592007479 DOWN GNG5P2
SCN3B 1.307523619 -3.486479143 4.943330198 1.72E-06 6.37E-06
4.576242857 UP SCN3B
SFRP5 3.062026356 -1.709457082 4.941272696 1.74E-06 6.43E-06
4.473213012 UP SFRP5
DYX1C1 1.103339723 -3.60560099 4.940404478 1.74E-06 6.45E-06
4.568769114UP DYX1C1
SSTR2 1.439384537 0.955789941 4.940036177 1.75E-06 6.46E-06
4.276338258 UP SSTR2
NOS2 -1.081003436 -0.0900928 -4.939345838 1.75E-06 6.48E-06
4.413688703 DOWN NOS2
SCX 1.024110646-0.732756731 4.937779529 1.76E-06 6.52E-06
4.424658865 UP SCX
AC007773.2 1.03896729 -1.25332774 4.937539987 1.76E-06 6.53E-06
4.459670744 UP AC007773.2
SLC26A9 1.779792259 -3.497592345 4.936408091 1.77E-06 6.56E-
06 4.540126646 UP SLC26A9
PRAC2 1.150742322 -5.766856461 4.935418014 1.78E-06 6.58E-06
4.553923871 UP PRAC2

MERTK-1.060318191	3.61906213	-4.93192971 1.81E-06	6.68E-06	3.960358362	DOWN MERTK
ANKRD22	1.684015581	-1.383235952	4.931336647	1.82E-06	6.70E-06	4.433406578	UP	ANKRD22
LINC00648	1.962597716	-4.80900452 4.930624823	1.82E-06	6.72E-06	4.532771521	UP	LINC00648
VIM_AS1	1.088425438	-1.718009647	4.930009164	1.83E-06	6.73E-06	4.455756993	UP	VIM-AS1
GAS6_AS2	-1.062212546	-1.680841749	-4.929531794	1.83E-06	6.74E-06	4.476807043	DOWN GAS6-AS2
CPN1	-1.685742424	4.553767879	-4.929013865	1.83E-06	6.76E-06	3.870204494	DOWN CPN1
SERPINA5	-1.404456526	8.134670119-4.928800185	1.84E-06	6.76E-06	3.799742948	DOWN SERPINA5
PRSS12 2.054127501	-1.521862774	4.928794829	1.84E-06	6.76E-06	4.42532001	UP	PRSS12
CCDC162P	1.935185207	-0.821235365	4.927883955	1.84E-06	6.79E-06	4.37519855	UP	CCDC162P
CPSF4L 1.229387128	-4.04455573 4.926694179	1.85E-06	6.82E-06	4.514596991	UP	CPSF4L
LHFPL4	2.098471982	-3.872850338	4.926314364	1.86E-06	6.83E-06	4.499721786	UP	LHFPL4
TXLNB 1.190328455	-1.495427591	4.925692258	1.86E-06	6.85E-06	4.422329224	UP	TXLNB
RP11_569G13.3 1.511593641-5.103410685	4.925602203	1.86E-06	6.85E-06	4.51241947	UP	RP11-569G13.3
CLDN4 2.293252033	2.190248214	4.924564882	1.87E-06	6.88E-06	4.064300798	UP	CLDN4
TG	-1.376828441	-1.248926423	-4.923948894	1.88E-06	6.90E-06	4.430694368	DOWN TG
RP11_55L3.1	-1.549851876	-5.721852891	-4.922360877	1.89E-06	6.94E-06	4.496396302	DOWN RP11-55L3.1
GXYLT2	1.532227592	-1.433279133	4.922003813	1.89E-06	6.95E-

06	4.397342974	UP	GXYLT2

CCR3	1.691751768	-4.117148998	4.919378044	1.92E-06	7.03E-06	4.480345566	UP	CCR3

FBXO2	-1.386300092	4.251891975	-4.919261578	1.92E-06	7.03E-06	3.846928943	DOWN	FBXO2

MICALCL	-1.017078365	-2.253180114	-4.918826486	1.92E-06	7.04E-06	4.457825771	DOWN	MICALCL

LAMC2	1.99099081	0.134886834	4.91753229	1.93E-06	7.08E-06	4.252610666	UP	LAMC2

NCCRP1	1.432845019	-3.379859218	4.917318526	1.93E-06	7.09E-06	4.459138336	UP	NCCRP1

LINC01143	1.041835877	-5.678648234	4.911277664	1.99E-06	7.27E-06	4.450098468	UP	LINC01143

HIST1H4E	1.15053396	-3.422950156	4.911132439	1.99E-06	7.27E-06	4.43766182	UP	HIST1H4E

FAM180A	-1.920454123	-1.034593415	-4.908793911	2.01E-06	7.34E-06	4.359028779	DOWN	FAM180A

THPO	-1.225582487	3.456477406	-4.908762216	2.01E-06	7.34E-06	3.882833389	DOWN	THPO

RP4_754E20__A.5	-1.940799367	-3.571269281	-4.907083881	2.03E-06	7.40E-06	4.431829252	DOWN	RP4-754E20--A.5

RP11_238F2.1	-2.764434669	-2.980761832	-4.90634589	2.03E-06	7.42E-06	4.416633221	DOWN	RP11-238F2.1

RP11_375H19.2	-1.184655715	-4.290153946	-4.906200417	2.03E-06	7.42E-06	4.43061019	DOWN	RP11-375H19.2

SEC14L6	1.813081364	-1.816462229	4.905629867	2.04E-06	7.44E-06	4.344801336	UP	SEC14L6

SPECC1	1.136220453	2.446487008	4.90416457	2.05E-06	7.49E-06	3.950689183	UP	SPECC1

BRSK2	1.723820503	-2.0652724	4.903923864	2.05E-06	7.49E-06	4.349886007	UP	BRSK2

PIK3C2G	-2.18259474	1.437405117	-4.903555216	2.06E-06	7.50E-06	4.144032085	DOWN	PIK3C2G

ZNF331 1.166060541 3.22930783 4.903363743 2.06E-06 7.51E-06 3.852970537 UP ZNF331

SLC6A17 1.349192869 -3.416085655 4.902467369 2.07E-06 7.54E-06 4.397607518 UP SLC6A17

CTD_2091N23.1 1.187308129 -5.497417452 4.899759647 2.09E-06 7.62E-06 4.400895632 UP CTD-2091N23.1

GRM3 1.381061793 -4.790134436 4.899357632 2.10E-06 7.64E-06 4.399732611 UP GRM3

RP11_278J6.4 -1.06086382 -2.537534194 -4.898173112 2.11E-06 7.68E-06 4.379244879 DOWN RP11-278J6.4

RP11_218E20.3 1.44229202 -4.528611626 4.897959394 2.11E-06 7.68E-06 4.393048375 UP RP11-218E20.3

CTD_2280E9.1 -1.801905531 -3.37537717 -4.895578003 2.13E-06 7.76E-06 4.381125874 DOWN CTD-2280E9.1

AC009264.1 1.163257866 -5.720125548 4.893849135 2.15E-06 7.82E-06 4.374940839 UP AC009264.1

LINC00173 1.192895499 -3.490847737 4.893287727 2.16E-06 7.84E-06 4.361745721 UP LINC00173

TMEM158 1.292012443 -0.88031165 4.893226037 2.16E-06 7.84E-06 4.23937287 UP TMEM158

TM4SF5 -1.653099131 6.488121445 -4.893115325 2.16E-06 7.84E-06 3.635523524 DOWN TM4SF5

OMD -1.728035143 -2.572666862 -4.892498665 2.16E-06 7.86E-06 4.355160798 DOWN OMD

AC008592.5 -1.205674838 -4.558327227 -4.89210679 2.17E-06 7.87E-06 4.369980924 DOWN AC008592.5

PDCD1 1.468842819 -0.471915128 4.891714648 2.17E-06 7.88E-06 4.198537841 UP PDCD1

RP11_1260E13.3 -1.205814004 -2.061000175 -4.887834584 2.21E-06 8.01E-06 4.317120003 DOWN RP11-1260E13.3

PALM2_AKAP2 -1.440858931 -2.407287034 -4.887074155 2.22E-06 8.04E-06 4.327855079 DOWN PALM2-AKAP2

USP12_AS2 -1.148807721 -3.925665097 -4.884189211 2.24E-06 8.14E-

06	4.335698892	DOWN	USP12-AS2
RP11_701H24.7	1.287902227	-4.093306856	4.883179397	2.26E-06	8.17E-06	4.327478247	UP	RP11-701H24.7
ENTPD2	1.258785456	1.14902629	4.882616622	2.26E-06	8.19E-06	4.009400827	UP	ENTPD2
BAI1	1.281655472	-1.778176276	4.880748737	2.28E-06	8.26E-06	4.24299169	UP	BAI1
ERVV_2	1.013095379	-5.951716806	4.88060016	2.28E-06	8.26E-06	4.317493947	UP	ERVV-2
VN1R1	1.149333001	-1.49320208	4.880303982	2.28E-06	8.27E-06	4.227075419	UP	VN1R1
ZNF660	1.000099582	-1.042589736	4.879424748	2.29E-06	8.30E-06	4.195067925	UP	ZNF660
LINC01139	2.21114628	-3.911466334	4.876441815	2.32E-06	8.41E-06	4.285765077	UP	LINC01139
RERGL	-1.356038328	-1.367365011	-4.874111982	2.35E-06	8.49E-06	4.223031475	DOWN	RERGL
SCNN1A	-1.234829133	1.88016653	-4.873877007	2.35E-06	8.50E-06	3.942996554	DOWN	SCNN1A
FST	-1.463154844	5.935840888	-4.873277384	2.36E-06	8.52E-06	3.555753688	DOWN	FST
TEDDM2P	-1.514664451	-5.465113563	-4.869884036	2.39E-06	8.65E-06	4.272166277	DOWN	TEDDM2P
RP1_90J4.1	-1.276117431	-4.849175214	-4.869772627	2.39E-06	8.65E-06	4.273874313	DOWN	RP1-90J4.1
FIGN	1.13370333	1.334670421	4.8691814	2.40E-06	8.67E-06	3.934082668	UP	FIGN
ITIH5	2.141795669	1.952061234	4.868609021	2.41E-06	8.69E-06	3.848428901	UP	ITIH5
PLGLB1	-1.304987302	0.358398705	-4.868461347	2.41E-06	8.69E-06	4.074384137	DOWN	PLGLB1
CTD_2240J17.4	-1.140954319	-3.818564878	-4.867485293	2.42E-06	8.73E-06	4.263947182	DOWN	CTD-2240J17.4

GCNT1 1.025836915 0.531415527 4.865618049 2.44E-06 8.80E-06 4.005446558 UP GCNT1

KAZALD1 1.1152935780.762321662 4.865062345 2.45E-06 8.82E-06 3.977463087 UP KAZALD1

TPRXL 1.498441935 -4.374844473 4.863886549 2.46E-06 8.86E-06 4.246064631 UP TPRXL

ZNF541 -1.519626061 -0.857438792 -4.86220439 2.48E-06 8.92E-06 4.142171397 DOWN ZNF541

EGFEM1P 1.621661052 -4.450851037 4.86157089 2.48E-06 8.94E-06 4.236035174 UP EGFEM1P

COLEC12 1.390523578 0.300493 4.8611472592.49E-06 8.96E-06 4.001521257 UP COLEC12

SPAG6 1.159281074 -4.676991533 4.860935275 2.49E-06 8.96E-06 4.235440711UP SPAG6

HYAL4 1.175885672 -5.317403364 4.857870968 2.53E-06 9.08E-06 4.221823184 UP HYAL4

ZSWIM5 1.4711959141.558276143 4.857534804 2.53E-06 9.09E-06 3.851490537 UP ZSWIM5

SMR3A -1.586708384 -5.929146247 -4.857349707 2.53E-06 9.10E-06 4.216192354 DOWN SMR3A

APBA2 1.326099651 0.022700823 4.857215565 2.53E-06 9.10E-06 4.010334635 UP APBA2

RP11_832A4.7 1.104652447 -4.326481904 4.856204022 2.55E-06 9.14E-06 4.21450539 UP RP11-832A4.7

LINC01135 -1.130116995 -4.014795426 -4.853331767 2.58E-06 9.25E-06 4.203866405 DOWN LINC01135

JPH1 1.791362561 -1.361620082 4.851395651 2.60E-06 9.33E-06 4.086845384 UP JPH1

COL24A1 1.722404963 -2.522833037 4.850828865 2.61E-06 9.35E-06 4.142756782 UP COL24A1

PNPLA5 -1.317719662 -5.171659307 -4.849981307 2.62E-06 9.38E-06 4.188510164 DOWN PNPLA5

GS1_44D20.1 1.008657619 -3.277678975 4.849001982 2.63E-06

9.42E-06 4.169910056 UP GS1-44D20.1
NTF3 -1.403176867 -1.766085474 -4.84420193 2.69E-06 9.61E-06
4.117411734 DOWN NTF3
RP11_790I12.2 -1.308593658 -4.799840446 -4.842861829 2.70E-06
9.66E-06 4.159039726 DOWN RP11-790I12.2
ZNF793_AS1 1.475985796 -2.019396811 4.8412691 2.72E-06 9.73E-
06 4.08339312 UP ZNF793-AS1
NAP1L4P1 1.487460525 -2.097331379 4.839536208 2.74E-06 9.79E-
06 4.079227469 UP NAP1L4P1
RP11_431K24.3 -1.381287273 -3.773720648 -4.837208995 2.77E-06
9.89E-06 4.134532284 DOWN RP11-431K24.3
HTRA3 1.423149567 1.745224712 4.837025702 2.77E-06 9.89E-06
3.742726012 UP HTRA3
CAMK1D -1.016258819 4.495184667 -4.836312395 2.78E-06 9.92E-
06 3.456497669 DOWN CAMK1D
GALNT7 1.126038893 0.980093294 4.835629279 2.79E-06 9.95E-
06 3.829393706 UP GALNT7
SLC2A12 -1.760022538 1.039820177 -4.835505669 2.79E-06 9.95E-
06 3.879346726 DOWN SLC2A12
RP11_383J24.1 1.419885896 -4.74178872 4.835098547 2.80E-06 9.97E-
06 4.124795673 UP RP11-383J24.1
RP11_395G23.3 1.178740781 -1.855515606 4.834813114 2.80E-06 9.98E-
06 4.052904945 UP RP11-395G23.3
CLUL1 1.207095618 -3.312276447 4.834706393 2.80E-06 9.98E-06
4.106689382 UP CLUL1
GFRA2 -1.075554052 0.504600116 -4.831438465 2.84E-06 1.01E-05
3.897918677 DOWN GFRA2
LRRC37A4P 1.084634941 -2.127469379 4.829689545 2.86E-06
1.02E-05 4.046244044 UP LRRC37A4P
CHRM2 -1.521792821 -4.919242592 -4.829333196 2.87E-06 1.02E-05
4.100974647 DOWN CHRM2
ESPNP -1.245130699 -3.16297019 -4.828827733 2.88E-06 1.02E-05
4.095127673 DOWN ESPNP

SEMA3C 1.633337985 -0.277740623 4.827136973 2.90E-06 1.03E-05 3.904638551 UP SEMA3C

MAGEA4 1.886781247 -5.383226901 4.82679533 2.90E-06 1.03E-05 4.088298609 UP MAGEA4

RP11_338N10.3 1.296407842 -4.733143699 4.826448048 2.91E-06 1.03E-05 4.088276215 UP RP11-338N10.3

IGSF11 1.36171659 -4.643924242 4.82502611 2.93E-06 1.04E-05 4.081979815 UP IGSF11

EPHB6 1.373809614 1.446367647 4.824528088 2.93E-06 1.04E-05 3.726559995 UP EPHB6

KAAG1 1.599572519 -2.028942302 4.823986382 2.94E-06 1.04E-05 4.008334641 UP KAAG1

IFI27 -1.74943168 6.31662503 -4.822706954 2.96E-06 1.05E-05 3.334135703 DOWN IFI27

RP11_141O11.2 -1.323375515 -2.286621974 -4.821948706 2.97E-06 1.05E-05 4.045477873 DOWN RP11-141O11.2

APLNR -1.11001003 3.789591887 -4.821623588 2.97E-06 1.05E-05 3.464895847 DOWN APLNR

SDSL -1.055351573 5.141541352 -4.819907708 2.99E-06 1.06E-05 3.346425698 DOWN SDSL

TMEM179 1.785415036 -4.171943235 4.818170933 3.02E-06 1.07E-05 4.047088505 UP TMEM179

LINC01136 1.245032952 -3.39505783 4.812793392 3.09E-06 1.09E-05 4.014967577 UP LINC01136

ANGPT4 -1.419353738 -3.764967684 -4.812458866 3.09E-06 1.10E-05 4.029353632 DOWN ANGPT4

GDNF_AS1 -1.918477151 -2.694895576 -4.811914889 3.10E-06 1.10E-05 4.013008529 DOWN GDNF-AS1

CTD_2377O17.1-1.492107583 -2.677102954 -4.810572856 3.12E-06 1.10E-05 4.008645794 DOWN CTD-2377O17.1

RP11_439M11.1 1.209086368 -5.160836336 4.808364662 3.15E-06 1.11E-05 4.011495056UP RP11-439M11.1

SLC5A5 1.284078361 -4.288200481 4.808322573 3.15E-06 1.11E-

05 4.009989836 UP SLC5A5

SPINK4 1.603134195 -4.919499237 4.807231788 3.17E-06 1.12E-05 4.006149742 UP SPINK4

GDA -2.125350649 3.975528772 -4.804986259 3.20E-06 1.13E-05 3.415317537 DOWN GDA

RP11_685B14.1 -1.293886537 -3.073166479 -4.803356288 3.22E-06 1.14E-05 3.985854351 DOWN RP11-685B14.1

ASTN1 -1.453082312 -2.227135512 -4.802272286 3.24E-06 1.14E-05 3.959995783 DOWN ASTN1

ANO7P1 -1.120558411 -0.470804483 -4.801924925 3.24E-06 1.14E-05 3.852355126 DOWN ANO7P1

ADCY1 -1.797907805 2.309989714 -4.80081181 3.26E-06 1.15E-05 3.58946844 DOWN ADCY1

TRPV6 2.146254546 -2.628591398 4.80007223 3.27E-06 1.15E-05 3.921836294 UP TRPV6

RP11_595B24.1 -1.522214779 -3.688632982 -4.799339714 3.28E-06 1.16E-05 3.973427228 DOWN RP11-595B24.1

SNCB 1.023098484 -5.400319711 4.799129995 3.28E-06 1.16E-05 3.972153653 UP SNCB

ALOX12P2 -1.33582598 0.141321905 -4.794927935 3.34E-06 1.18E-05 3.778977234 DOWN ALOX12P2

PLTP 1.181435631 4.252587208 4.792547175 3.38E-06 1.19E-05 3.276659526 UP PLTP

NBEA 1.109645134 -0.099098983 4.790381216 3.41E-06 1.20E-05 3.740177847 UP NBEA

LINC01116 1.333441849 -1.743208695 4.789773684 3.42E-06 1.20E-05 3.852832906 UP LINC01116

BHLHE41 1.336542576 0.692246358 4.788048075 3.45E-06 1.21E-05 3.652534049 UP BHLHE41

LPO 1.250072925 -4.946045972 4.787396965 3.46E-06 1.21E-05 3.922996087 UP LPO

RP11_758M4.4 1.806163795 -5.004033131 4.784591549 3.50E-06 1.23E-05 3.909948117UP RP11-758M4.4

RP11_669N7.2 1.747644549 -5.336251981 4.784081787 3.51E-06 1.23E-05 3.90771428 UP RP11-669N7.2

SLC22A12 -3.166461737 -1.551893795 -4.783493725 3.52E-06 1.23E-05 3.855521638 DOWN SLC22A12

ALDH3B2 1.708405646 -4.441810117 4.782890427 3.53E-06 1.24E-05 3.901380548 UP ALDH3B2

CAPN6 2.359225779 -2.190138835 4.7803323113.57E-06 1.25E-05 3.81743842 UP CAPN6

TPPP -1.257918642 2.35506225 -4.780168002 3.57E-06 1.25E-05 3.482394198 DOWN TPPP

ZNF682 1.184528839 0.573244785 4.77928916 3.58E-06 1.26E-05 3.629217227 UP ZNF682

XCL1 1.361500861 -1.660811252 4.77677123 3.62E-06 1.27E-05 3.792405381 UP XCL1

CTA_293F17.1 1.51924247 -4.02118236 4.775289477 3.65E-06 1.28E-05 3.866157892 UP CTA-293F17.1

AC006273.5 1.406575803 -1.648981245 4.774152486 3.67E-06 1.28E-05 3.780522812 UP AC006273.5

CLEC1B -1.983795938 -3.657844881 -4.773242207 3.68E-06 1.29E-05 3.862343004 DOWN CLEC1B

RP11_327J17.2 -1.461380114 -1.713179487 -4.772966447 3.68E-06 1.29E-05 3.812891155DOWN RP11-327J17.2

RP11_477H21.2 1.379949707 -3.741337609 4.772857279 3.69E-06 1.29E-05 3.852358882 UP RP11-477H21.2

IL17B 1.080090957 -4.52777324 4.7721162913.70E-06 1.29E-05 3.858607717 UP IL17B

ZNF829 1.056577567 0.20143569 4.77116091 3.71E-06 1.30E-05 3.632339747 UP ZNF829

ARHGEF37 -1.15035149 2.227437429 -4.769401243 3.74E-06 1.31E-05 3.449242982 DOWN ARHGEF37

TMSB15A 1.092733243 -3.306360396 4.7691111683.75E-06 1.31E-05 3.830671136UP TMSB15A

KCNMB2_AS1 1.916624025 -2.259532223 4.76857156 3.76E-06 1.31E-

05 3.777146035 UP KCNMB2-AS1

TTC9 -1.286119291 3.470179547 -4.768362691 3.76E-06 1.31E-05 3.284861627 DOWN TTC9

TRPC7_AS1 1.259578387 -5.339812947 4.767848529 3.77E-06 1.32E-05 3.840019824 UP TRPC7-AS1

RP11_156K13.2 -1.239734018 -3.79951631 -4.766976156 3.78E-06 1.32E-05 3.837457301 DOWN RP11-156K13.2

HAPLN1 1.618641238 -4.01093105 4.766527213 3.79E-06 1.32E-05 3.828237813 UP HAPLN1

HSD17B2 -1.307104982 6.015846448 -4.765475843 3.81E-06 1.33E-05 3.090213091 DOWN HSD17B2

AP1M2 2.511622244 0.179022711 4.763621366 3.84E-06 1.34E-05 3.578570186 UP AP1M2

FZD9 1.726963666 -2.686215849 4.75991275 3.90E-06 1.36E-05 3.76001377 UP FZD9

NLRP11 -1.883662401 -1.078438311 -4.758233849 3.93E-06 1.37E-05 3.719131694 DOWN NLRP11

KIF25_AS1 1.385278199 -4.864744091 4.758201324 3.93E-06 1.37E-05 3.79977711 UP KIF25-AS1

FAM83B 1.769525919 -4.631402806 4.757062372 3.95E-06 1.38E-05 3.793456973 UP FAM83B

RNF165 -1.287638508 -0.365486402 -4.756896106 3.96E-06 1.38E-05 3.656181287 DOWN RNF165

B3GNT7 1.314059831 0.41548067 4.756630899 3.96E-06 1.38E-05 3.547221486 UP B3GNT7

TUSC3 1.301194495 1.992498181 4.756224735 3.97E-06 1.38E-05 3.370692353 UP TUSC3

AC114498.1 -1.144500744 -2.426913358 -4.753298034 4.02E-06 1.40E-05 3.759964915 DOWN AC114498.1

FOSB -1.573342971 2.903720236 -4.752821315 4.03E-06 1.40E-05 3.302777759 DOWN FOSB

SMLR1 -1.293417863 4.599831993 -4.750689777 4.07E-06 1.41E-05 3.086684726 DOWN SMLR1

CDA -1.401108955 2.926425673 -4.74985277 4.08E-06 1.42E-05 3.282905038 DOWN CDA
CTD_2575K13.6 1.433569846 -4.237963363 4.746537055 4.14E-06 1.44E-05 3.748401542 UP CTD-2575K13.6
PANX2 -1.500145504 3.283145158 -4.744036298 4.19E-06 1.45E-05 3.209929673 DOWN PANX2
AS3MT -1.23609122 0.245709363 -4.742489521 4.21E-06 1.46E-05 3.5461268 DOWN AS3MT
ISM2 1.507877585 -5.091024426 4.738608244 4.29E-06 1.48E-05 3.717224576 UP ISM2
FAM46B 1.129156342 -1.947733307 4.737100203 4.32E-06 1.49E-05 3.644577063 UP FAM46B
RP11_227H15.5 1.192993273 -4.731001046 4.736898225 4.32E-06 1.49E-05 3.710747546 UP RP11-227H15.5
ASAH2 -1.178865749 0.392063278 -4.736647237 4.32E-06 1.50E-05 3.507867244 DOWN ASAH2
FAM27C 1.25872717 -4.317118924 4.736574968 4.33E-06 1.50E-05 3.708051506 UP FAM27C
NEB 1.521942554 2.226692293 4.735172233 4.35E-06 1.51E-05 3.250887353 UP NEB
CALB2 1.490685003 -3.982637226 4.734732063 4.36E-06 1.51E-05 3.695542515 UP CALB2
P2RY6 1.077543589 0.359821509 4.734147741 4.37E-06 1.51E-05 3.46065187 UP P2RY6
GNGT1 1.70087952 -5.096424945 4.73409586 4.37E-06 1.51E-05 3.697935708 UP GNGT1
TUBBP5 2.060296744 -2.812677409 4.732977732 4.39E-06 1.52E-05 3.645334123 UP TUBBP5
EMILIN3 1.162863378 -3.058600698 4.732796041 4.40E-06 1.52E-05 3.669955342 UP EMILIN3
FOXD3_AS1 1.2181479 -5.538446703 4.732261779 4.41E-06 1.52E-05 3.690283306 UP FOXD3-AS1
LINC01559 1.774321445 -4.599029699 4.731471147 4.42E-06 1.53E-05

3.68599301 UP LINC01559

ST18 1.421099925 -3.958551284 4.730061725 4.45E-06 1.54E-05

3.67625444 UP ST18

OGN -2.069927405 -0.832678544 -4.729427349 4.46E-06 1.54E-05

3.583916931 DOWN OGN

LGALS17A 1.491645578 -4.056699838 4.7291933 4.47E-06 1.54E-05

3.673232086 UP LGALS17A

CTD_2587H19.21.183552374 -5.279419218 4.727837283 4.50E-06

1.55E-05 3.672380422 UP CTD-2587H19.2

RP11_740C1.2 -1.399055931 -1.316037321 -4.723314668 4.59E-06

1.58E-05 3.580785998 DOWN RP11-740C1.2

CLEC4G -1.915207401 -1.82257443 -4.715250483 4.75E-06 1.63E-05

3.578504782 DOWN CLEC4G

GACAT2 1.158558443 -5.647139971 4.713721008 4.78E-06 1.64E-

05 3.61259029 UP GACAT2

SLC13A3 -2.382389093 3.650128683 -4.712249807 4.81E-06 1.65E-

05 3.064609163 DOWN SLC13A3

CXCL3 1.446493621 -1.868544203 4.7110125554.84E-06 1.66E-05

3.527609753 UP CXCL3

ZNF354C 1.007010714 -0.126484342 4.7110023924.84E-06 1.66E-05

3.408792167 UP ZNF354C

ADAM11 1.046061624 -1.450612004 4.709993656 4.86E-06 1.67E-

05 3.503445774 UP ADAM11

DLX2 1.402699675 -4.976014407 4.709300306 4.88E-06 1.67E-05

3.595047859 UP DLX2

LINC00864 -1.894483904 -3.258247485 -4.707383389 4.92E-06 1.68E-

05 3.583125045 DOWN LINC00864

ZNF415 1.084618214 -0.654438485 4.706975203 4.93E-06 1.69E-05

3.434063096 UP ZNF415

CTA_221G9.12 -1.116941945 0.108014136 -4.706044823 4.95E-06

1.69E-05 3.401864459 DOWN CTA-221G9.12

SPAG171.237368836 -4.290176771 4.704421864 4.98E-06 1.70E-05

3.573788668 UP SPAG17

PPM1H 1.150694422 2.568962967 4.703663949 5.00E-06 1.71E-05 3.077952796 UP PPM1H

SH2D6 1.038071151-2.196912056 4.70283298 5.02E-06 1.71E-05 3.515106971 UP SH2D6

HIST1H4K -1.093383115 -3.104862431 -4.701563079 5.04E-06 1.72E-05 3.559181257 DOWN HIST1H4K

HMGCLL1 -1.346262862 -3.944124703 -4.700457757 5.07E-06 1.73E-05 3.559335423 DOWN HMGCLL1

LINC01182 -1.517268459 -4.199578429 -4.699313449 5.09E-06 1.74E-05 3.554755737 DOWN LINC01182

ERICH4 1.23228217 -5.026042963 4.6966404115.15E-06 1.76E-05 3.542594715 UP ERICH4

XKR5 1.062717521 -5.330391189 4.696019297 5.17E-06 1.76E-05 3.539733549 UP XKR5

CSN2 -1.380454465 -5.717122791 -4.695169655 5.19E-06 1.77E-05 3.53410497 DOWN CSN2

SNRPN 1.012148922 4.929245382 4.693361225 5.23E-06 1.78E-05 2.812690206 UP SNRPN

SLC22A18AS -1.255541075 1.657727546 -4.690499143 5.29E-06 1.80E-05 3.187634282 DOWN SLC22A18AS

PBX4 1.061955023 -1.986999853 4.690126182 5.30E-06 1.80E-05 3.451231715 UP PBX4

AATK 1.093568888 0.501399725 4.689923612 5.31E-06 1.81E-05 3.26125373 UP AATK

HS3ST61.179865123 -5.284288578 4.689798231 5.31E-06 1.81E-05 3.513822182 UP HS3ST6

GOLT1A -1.100342111 5.993918484 -4.689199711 5.32E-06 1.81E-05 2.766439561 DOWN GOLT1A

FBXW11P1 -1.169066494 -2.672389112 -4.688055342 5.35E-06 1.82E-05 3.49471804 DOWN FBXW11P1

RP11_399H11.2 -1.242970492 -5.772347665 -4.687708285 5.36E-06 1.82E-05 3.503020357 DOWN RP11-399H11.2

CMB9_22P13.1 1.060530239 -0.803026551 4.6868888 5.38E-06 1.83E-

05 3.362007141 UP CMB9-22P13.1
FAM101A 1.838729398 -1.358879365 4.683998548 5.45E-06 1.85E-05 3.377787077 UP FAM101A
EDA 1.223768612 1.004002773 4.682526153 5.48E-06 1.86E-05 3.17716736 UP EDA
FOXF2 1.294345197 -2.06309154 4.681916513 5.50E-06 1.87E-05 3.418138912 UP FOXF2
RP11_713C5.1 -1.09882891 -5.310066385 -4.681607463 5.50E-06 1.87E-05 3.47995665 DOWN RP11-713C5.1
RASGEF1C 1.056116312 -4.616742613 4.680936322 5.52E-06 1.87E-05 3.477646616 UP RASGEF1C
CR2 1.637199043 -3.825011418 4.677386526 5.60E-06 1.90E-05 3.451946752 UP CR2
ENTPD8 -1.366139428 2.350308345 -4.677128526 5.61E-06 1.90E-05 3.050813752 DOWN ENTPD8
VSIG2 -1.515555098 -0.180398914 -4.672913756 5.72E-06 1.93E-05 3.294153985 DOWN VSIG2
PWRN1 -1.179067006 -5.237816364 -4.670823365 5.77E-06 1.95E-05 3.435386218 DOWN PWRN1
HOXA10 2.051068278 -1.333999339 4.670506686 5.78E-06 1.95E-05 3.316024576 UP HOXA10
CYP2G1P -1.224473412 -4.610077445 -4.670181749 5.78E-06 1.96E-05 3.433969873 DOWN CYP2G1P
RP11_164J13.1 -1.067517651 -0.484837229 -4.669028933 5.81E-06 1.96E-05 3.293830366 DOWN RP11-164J13.1
DIRAS2 1.878036084 -2.320783148 4.667270328 5.86E-06 1.98E-05 3.358206635 UP DIRAS2
PAGE1 2.037439566 -4.808979193 4.666566307 5.88E-06 1.98E-05 3.415526493 UP PAGE1
AC025627.7 -1.050884354 -2.628655775 -4.666240951 5.88E-06 1.99E-05 3.402972586 DOWN AC025627.7
EYA1 1.512892121 -4.397966189 4.665216795 5.91E-06 1.99E-05 3.410415975 UP EYA1

RAB25 2.320190351 -2.40874271 4.663956598 5.94E-06 2.01E-05 3.33876829 UP RAB25

PDIA2 1.753500146 -2.615639208 4.662540802 5.98E-06 2.02E-05 3.350918236 UP PDIA2

AIRE 1.094637045 -4.741959509 4.662181795 5.99E-06 2.02E-05 3.399996296 UP AIRE

CTD_2020K17.1 1.002141923 -0.642956323 4.661123639 6.02E-06 2.03E-05 3.242779172 UP CTD-2020K17.1

LMTK3 1.658035626 -1.014192331 4.660857467 6.02E-06 2.03E-05 3.261650948 UP LMTK3

ATP6V1B1 1.353619096 -2.023559508 4.660646331 6.03E-06 2.03E-05 3.325862751 UP ATP6V1B1

SAGE1 1.359575762 -5.664682731 4.658168015 6.09E-06 2.05E-05 3.381679515 UP SAGE1

GDF7 -1.146250237 2.249848894 -4.653172623 6.23E-06 2.10E-05 2.954347752 DOWN GDF7

NUDT16P1 -1.346203627 1.722595555 -4.652328582 6.25E-06 2.10E-05 3.024413601 DOWN NUDT16P1

ANGPTL7 -1.790470951 -4.958983227 -4.652046007 6.26E-06 2.10E-05 3.357635485 DOWN ANGPTL7

GNAT1 -1.177666733 -1.903132392 -4.651475502 6.27E-06 2.11E-05 3.312539272 DOWN GNAT1

HOXC13 1.435089477 -5.213205023 4.650606531 6.30E-06 2.12E-05 3.351270273 UP HOXC13

EFHC2 1.392770924 -4.170663642 4.64947402 6.33E-06 2.12E-05 3.344245025 UP EFHC2

CDC20B 1.713655738 -3.616103964 4.649040983 6.34E-06 2.13E-05 3.328629988 UP CDC20B

RBP2 1.721093281 -3.807246524 4.646780658 6.40E-06 2.15E-05 3.322969318 UP RBP2

HR 1.332623008 -0.983195948 4.646673026 6.41E-06 2.15E-05 3.203853439 UP HR

RP11_426C22.1 -1.342758748 -4.387226989 -4.640793859 6.57E-06

2.20E-05 3.312594822 DOWN RP11-426C22.1
DDC -1.792814657 4.073029518 -4.63899597 6.62E-06 2.21E-05 2.684282861 DOWN DDC
GALNT5 1.481783215 -3.647458995 4.633250059 6.79E-06 2.27E-05 3.268153215 UP GALNT5
XRCC6P1 -1.317361585 -1.903167538 -4.631317749 6.85E-06 2.28E-05 3.230682542 DOWN XRCC6P1
CSPG5 1.066553948 -0.688270943 4.6311880326.85E-06 2.28E-05 3.121580703 UP CSPG5
C14orf132 1.449833418 1.092406421 4.628948212 6.92E-06 2.31E-05 2.940621081 UP C14orf132
NLRP2 1.696577856 -1.82910202 4.626552763 6.99E-06 2.33E-05 3.169627043 UP NLRP2
RP11_429J17.7 1.10196712 -4.935386006 4.626046642 7.00E-06 2.33E-05 3.250973681 UP RP11-429J17.7
SIRPG 1.194562707 -0.558825083 4.625001985 7.04E-06 2.34E-05 3.083466309 UP SIRPG
RP11_268G13.1 1.065540644 -5.412726901 4.62497413 7.04E-06 2.34E-05 3.245808118UP RP11-268G13.1
MTATP8P1 -1.125577738 -2.57503448 -4.624430192 7.05E-06 2.35E-05 3.229002763 DOWN MTATP8P1
FGF11 1.011220943-4.597670306 4.621391226 7.15E-06 2.38E-05 3.231912646 UP FGF11
ZC3H12B 1.066550615 -0.561903536 4.620720367 7.17E-06 2.38E-05 3.068264839 UP ZC3H12B
RP11_92C4.6 -1.042911139 -3.960430099 -4.620658854 7.17E-06 2.38E-05 3.229565673 DOWN RP11-92C4.6
SYNGR1 1.330148289 2.901771877 4.620429212 7.18E-06 2.38E-05 2.689559581 UP SYNGR1
RPL39P40 -1.330323763 -1.460930411 -4.619022661 7.22E-06 2.40E-05 3.155675729 DOWN RPL39P40
ART3 1.231689492 -4.022591154 4.618534207 7.23E-06 2.40E-05 3.216278108 UP ART3

C8orf31 1.194151726 -2.431810938 4.618241529 7.24E-06 2.40E-05 3.172229439 UP C8orf31

SRRM3 1.779729648 -0.453126187 4.617904745 7.25E-06 2.41E-05 3.038085297 UP SRRM3

GPR27 1.668726203 -2.99895898 4.616699968 7.29E-06 2.42E-05 3.178076307 UP GPR27

NPFFR2 1.986491161 -3.982398167 4.616601855 7.30E-06 2.42E-05 3.200179395 UP NPFFR2

TMEM151A 1.676949233 -1.179595793 4.615747758 7.32E-06 2.43E-05 3.084814768 UP TMEM151A

XKR6 1.029737474 -3.001866319 4.61549152 7.33E-06 2.43E-05 3.184057272 UP XKR6

MNX1_AS2 1.212284046 -5.345584391 4.61131985 7.46E-06 2.47E-05 3.189735679 UP MNX1-AS2

RP11_396C23.2 1.123446669 -1.535673496 4.610545771 7.49E-06 2.48E-05 3.09490774 UP RP11-396C23.2

NUDT11 1.450611452 -3.73235093 4.609691219 7.52E-06 2.49E-05 3.173416312 UP NUDT11

LINC00958 1.603110621 -4.938173391 4.609015075 7.54E-06 2.49E-05 3.18009351 UP LINC00958

SSTR1 -1.818425857 1.598664688 -4.606076568 7.63E-06 2.52E-05 2.859018224 DOWN SSTR1

KLHL29 1.191684685 2.410453867 4.602439709 7.75E-06 2.56E-05 2.67630464 UP KLHL29

AC019129.1 -1.220926859 -5.076355203 -4.60224473 7.76E-06 2.56E-05 3.153429598 DOWN AC019129.1

RP11_215P8.4 -1.691901979 -3.446671913 -4.601198199 7.80E-06 2.57E-05 3.147047896 DOWN RP11-215P8.4

RP11_88H9.2 -1.630482226 -2.264565007 -4.600374171 7.82E-06 2.58E-05 3.120300283 DOWN RP11-88H9.2

AC131097.4 -1.621998948 -3.920005753 -4.599700726 7.85E-06 2.59E-05 3.143238193 DOWN AC131097.4

TWIST1 1.269907151 -2.274162812 4.598743769 7.88E-06 2.60E-

05 3.084728801 UP TWIST1

PLA2G7 1.003284828 1.981050251 4.597318655 7.93E-06 2.61E-05 2.709803537 UP PLA2G7

RP11_794M8.2 -1.293031436 -5.613568105 -4.595289257 8.00E-06 2.63E-05 3.122849057 DOWN RP11-794M8.2

SV2C -1.042194491 -2.897343805 -4.588161329 8.25E-06 2.71E-05 3.088235596 DOWN SV2C

NTS 2.891325707 -1.242758734 4.585782173 8.33E-06 2.74E-05 2.949356864 UP NTS

AC009014.3 2.496926128 -1.870918422 4.584624353 8.37E-06 2.75E-05 2.984029973 UP AC009014.3

ANO2 -1.159221326 -1.562053187 -4.584301301 8.38E-06 2.75E-05 3.017576957 DOWN ANO2

NKAIN4 1.183631617 -4.508759246 4.583052318 8.43E-06 2.77E-05 3.074411171 UP NKAIN4

B3GNT5 1.177208261 2.100479606 4.582602794 8.44E-06 2.77E-05 2.633888531 UP B3GNT5

KB_1742H10.3 1.233715002 -3.738830217 4.581265612 8.49E-06 2.79E-05 3.059659459 UP KB-1742H10.3

CAMKV 1.399691123 -5.220065332 4.57951055 8.56E-06 2.81E-05 3.059624934 UP CAMKV

KCNC1 1.290598809 -4.062866464 4.579148319 8.57E-06 2.81E-05 3.055139882 UP KCNC1

FAM163B -1.910995116 -2.395388862 -4.577789789 8.62E-06 2.83E-05 3.031500212 DOWN FAM163B

CLLU1OS 1.193075504 -5.619640814 4.577221179 8.64E-06 2.83E-05 3.049694272 UP CLLU1OS

PTPN20A 1.645188825 -3.032769984 4.576929656 8.65E-06 2.83E-05 3.016242395 UP PTPN20A

GRHL2 1.902247312 -2.926214216 4.575617363 8.70E-06 2.85E-05 3.002150473 UP GRHL2

CACNA1D 1.146331205 1.373667969 4.572329786 8.83E-06 2.89E-05 2.68018696 UP CACNA1D

PTGES2_AS1 1.081080998 -3.184533091 4.571517743 8.86E-06 2.90E-05 3.008606154 UP PTGES2-AS1

DNAAF1 -1.592977202 -1.600047379 -4.571176957 8.87E-06 2.90E-05 2.970668839 DOWN DNAAF1

RP11_416N2.4 1.142457863 -4.194312272 4.571121117 8.87E-06 2.90E-05 3.024489207 UP RP11-416N2.4

ACPP 1.386431285 -3.243401001 4.570923103 8.88E-06 2.90E-05 3.003286446 UP ACPP

CTD_2314B22.1 1.093463009 -5.579741914 4.567093172 9.03E-06 2.95E-05 3.008650509 UP CTD-2314B22.1

INHBA_AS1 -1.115602355 -2.731853506 -4.567006062 9.03E-06 2.95E-05 2.998328482 DOWN INHBA-AS1

RP5_907C10.4 -1.517590881 -5.01177609 -4.565768694 9.08E-06 2.97E-05 3.004338045 DOWN RP5-907C10.4

DCSTAMP -1.008654017 -2.917704224 -4.565133961 9.10E-06 2.97E-05 2.99475495 DOWN DCSTAMP

PHGR1 -1.236305913 -5.112993371 -4.563274407 9.17E-06 3.00E-05 2.994310584 DOWN PHGR1

RP3_406A7.7 -1.061621528 0.146068481 -4.558744916 9.35E-06 3.05E-05 2.789569809 DOWN RP3-406A7.7

TTC39A_AS1 1.313848672 -3.959388327 4.557859052 9.39E-06 3.06E-05 2.96702096 UP TTC39A-AS1

ITGB3 -1.102103332 -0.603079114 -4.55618793 9.46E-06 3.08E-05 2.83887139 DOWN ITGB3

RP11_320M16.1 -1.046547589 -3.143223007 -4.554524002 9.52E-06 3.10E-05 2.955046827 DOWN RP11-320M16.1

IL2RA 1.128959641 -0.604146025 4.552803375 9.59E-06 3.12E-05 2.792160288 UP IL2RA

HAP1 1.168502742 -2.758345131 4.552687587 9.60E-06 3.12E-05 2.916786462 UP HAP1

FAM83H_AS1 1.062765594 -0.325163618 4.55104075 9.67E-06 3.14E-05 2.763320065 UP FAM83H-AS1

RP11_351J23.1 1.248850642 -1.535417728 4.550510038 9.69E-06

3.15E-05 2.8477615 UP RP11-351J23.1

ZNF469 1.055981195 0.12020577 4.550009235 9.71E-06 3.16E-05 2.720452921 UP ZNF469

LONRF2 1.648624933 0.039461518 4.548559885 9.77E-06 3.17E-05 2.712705273 UP LONRF2

ZNF560 1.517673913 -5.322189075 4.54563433 9.89E-06 3.21E-05 2.9215522 UP ZNF560

LINC00272 -1.168006068 -5.602440667 -4.545234759 9.91E-06 3.22E-05 2.919306169 DOWN LINC00272

CD1D -1.004810695 1.581737554 -4.545156582 9.91E-06 3.22E-05 2.59032877 DOWN CD1D

ZNF878 1.094806433 -3.923224137 4.544474699 9.94E-06 3.22E-05 2.914050323 UP ZNF878

RP11_1C1.4 1.398376629 -5.503717219 4.542670385 1.00E-05 3.25E-05 2.909346131 UP RP11-1C1.4

FAT2 1.022011319 -3.083861825 4.542523872 1.00E-05 3.25E-05 2.88907561 UP FAT2

TANC2 1.112662042 1.855243222 4.542085204 1.00E-05 3.25E-05 2.499556168 UP TANC2

AGTR1 -1.17944681 4.439436773 -4.539818885 1.01E-05 3.28E-05 2.218501388 DOWN AGTR1

RP11_347C12.12 -1.026389618 -2.549140209 -4.539701553 1.01E-05 3.28E-05 2.882171924 DOWN RP11-347C12.12

HBM -1.024591467 -5.814571812 -4.538775837 1.02E-05 3.30E-05 2.892227672 DOWN HBM

STK32A 1.254732289 -3.291107827 4.538588953 1.02E-05 3.30E-05 2.87564645 UP STK32A

RP11_936I5.1 -1.283025288 -2.994035011 -4.538571849 1.02E-05 3.30E-05 2.887904005 DOWN RP11-936I5.1

PLXNB3 1.518768272 -0.19704469 4.538421528 1.02E-05 3.30E-05 2.694467793 UP PLXNB3

AC005523.3 -1.024032562 -3.539804643 -4.537755058 1.02E-05 3.31E-05 2.890238013 DOWN AC005523.3

HSPA7 1.218757302 0.3911840734.535833166 1.03E-05 3.33E-05 2.63394567 UP HSPA7
CPNE7 1.478301026 0.017971345 4.534678455 1.04E-05 3.35E-05 2.659621718 UP CPNE7
FAM153A 1.484504202 -3.289315398 4.534604791 1.04E-05 3.35E-05 2.855487544 UP FAM153A
RP11_624L4.1 1.411053202-2.659673826 4.534289074 1.04E-05 3.35E-05 2.83407957 UP RP11-624L4.1
SPNS2 -1.074872712 2.761929026 -4.533735092 1.04E-05 3.36E-05 2.393328375 DOWN SPNS2
HFM1 1.155917939 -3.885943731 4.532918099 1.04E-05 3.37E-05 2.866286795 UP HFM1
SORCS3 1.194753838 -5.644130449 4.532540956 1.05E-05 3.37E-05 2.868206796 UP SORCS3
PCDHB2 1.492624604 -1.427429543 4.531996844 1.05E-05 3.38E-05 2.762601136UP PCDHB2
TRPV5 1.00596017 -5.56349276 4.52906332 1.06E-05 3.42E-05 2.854542623 UP TRPV5
IGSF1 1.902709782 0.86482994 4.527189444 1.07E-05 3.45E-05 2.537448243 UP IGSF1
AC013275.2 1.691979356 -0.940601619 4.526402545 1.07E-05 3.46E-05 2.704525636 UP AC013275.2
TMPRSS3 1.949198474 0.79525959 4.525969794 1.08E-05 3.46E-05 2.541098859 UP TMPRSS3
CD36 -1.259894847 5.025958459 -4.525926909 1.08E-05 3.46E-05 2.123752124 DOWN CD36
CHI3L1 -2.330461821 6.421539698 -4.525489089 1.08E-05 3.47E-05 2.093579596 DOWN CHI3L1
FBXO39 -1.072170764 -2.224833367 -4.52494982 1.08E-05 3.48E-05 2.810338802 DOWN FBXO39
ARL14 1.948252727 -2.35140718 4.52278405 1.09E-05 3.51E-05 2.765823669 UP ARL14
IL32 -1.018090113 7.8655277 -4.521730211 1.10E-05 3.52E-05

2.075723994 DOWN IL32

RP11_473M20.5 1.151740343 -4.249745205 4.521470626 1.10E-05 3.53E-05 2.823512904 UP RP11-473M20.5

CLCNKA 1.490561178-1.896023277 4.5201135671.10E-05 3.54E-05 2.742339791 UP CLCNKA

ROBO1 1.15565484 4.98781784 4.5176117741.11E-05 3.58E-05 2.083610656 UP ROBO1

TCF21 -1.03083492-0.725100174 -4.516064566 1.12E-05 3.60E-05 2.683419373 DOWN TCF21

CTB_39G8.2 -1.070429327 -3.618841549 -4.514133681 1.13E-05 3.63E-05 2.795153093 DOWN CTB-39G8.2

CYP2S1 1.14700667 0.223732606 4.513121986 1.14E-05 3.64E-05 2.559387741 UP CYP2S1

CTCFL -1.281263473 -4.182758234 -4.512551931 1.14E-05 3.65E-05 2.789936091 DOWN CTCFL

PCDHGA8 1.161344713 -3.220167067 4.510809704 1.15E-05 3.68E-05 2.762498021 UP PCDHGA8

CDKN2A 1.213343002 2.89627752 4.510340584 1.15E-05 3.68E-05 2.237893337 UP CDKN2A

LBP -1.455304462 8.433349972 -4.510238587 1.15E-05 3.69E-05 2.037701525 DOWN LBP

S100A9 1.303853359 2.657708223 4.509377616 1.15E-05 3.70E-05 2.262616467 UP S100A9

KCNJ10 1.423810454 -1.51717722 4.506675175 1.17E-05 3.74E-05 2.666115586UP KCNJ10

KCNE1 -1.151530227 -3.294346904 -4.505044016 1.18E-05 3.76E-05 2.756461937 DOWN KCNE1

AC104534.2 1.083077069 -2.680359731 4.502849317 1.19E-05 3.80E-05 2.713869671 UP AC104534.2

CHRNB2 1.101555281 -3.454183571 4.502617318 1.19E-05 3.80E-05 2.736188396 UP CHRNB2

MCOLN2 1.214738635 -1.31702081 4.500851014 1.20E-05 3.83E-05 2.633510124 UP MCOLN2

ADORA1 1.715705626 -0.333061675 4.500327368 1.20E-05 3.83E-05 2.548868561 UP ADORA1
AC099552.4 1.059424167 -5.713503889 4.500061567 1.20E-05 3.84E-05 2.737119491 UP AC099552.4
SLC38A5 1.075057026 -0.161378171 4.499626322 1.20E-05 3.84E-05 2.539924511 UP SLC38A5
TGFB2_AS1 1.234554356 -3.687894749 4.498874222 1.21E-05 3.86E-05 2.725002611 UP TGFB2-AS1
CTD_2186M15.1 -1.018934344 -4.822708751 -4.498595528 1.21E-05 3.86E-05 2.733678474 DOWN CTD-2186M15.1
CPA2 1.661589699 -4.963431092 4.497817389 1.21E-05 3.87E-05 2.728560846 UP CPA2
KRT15 1.761220847 -3.794896709 4.496620191 1.22E-05 3.89E-05 2.71126642 UP KRT15
KRT16P1 -1.105491345 -5.938181932 -4.495348938 1.23E-05 3.91E-05 2.716206548 DOWN KRT16P1
DLEU7 1.032610674 -2.986050479 4.494168464 1.23E-05 3.93E-05 2.691105749 UP DLEU7
AC002511.3 1.046130895 -5.542010263 4.493232202 1.24E-05 3.94E-05 2.710195056 UP AC002511.3
UCA1 2.213593724 -2.699713618 4.490983613 1.25E-05 3.98E-05 2.64630391 UP UCA1
AMH 1.396409415 -2.264585013 4.48997379 1.25E-05 3.99E-05 2.640660504 UP AMH
ADAMTS3 1.34285671 -1.729144566 4.489266322 1.26E-05 4.00E-05 2.611582419 UP ADAMTS3
AC011242.6 1.187384749 -3.644078839 4.484446664 1.28E-05 4.08E-05 2.66667793 UP AC011242.6
TRPA1 1.377411129 -4.155955697 4.483558222 1.29E-05 4.10E-05 2.669168485 UP TRPA1
RP11_180D21.3 -1.572768403 -3.468686836 -4.483214887 1.29E-05 4.10E-05 2.6694846 DOWN RP11-180D21.3
NAA11 1.867784183 -5.094061755 4.482971034 1.29E-05 4.11E-05

2.66845092 UP NAA11

RP11_488L18.3 1.007891646 -3.854025717 4.482752266 1.29E-05 4.11E-05 2.66489873 UP RP11-488L18.3

RP11_159K7.2 -1.458575264 -5.467595174 -4.481735648 1.30E-05 4.13E-05 2.663488727 DOWN RP11-159K7.2

NOS1 -1.414461448 -4.275254103 -4.47863039 1.31E-05 4.18E-05 2.653605547 DOWN NOS1

NCRNA00250 -1.102320666 -4.439270362 -4.478077222 1.32E-05 4.19E-05 2.651595565 DOWN NCRNA00250

CTD_2231H16.1 1.147374597 -5.431777925 4.477602703 1.32E-05 4.20E-05 2.647689038 UP CTD-2231H16.1

FAM35BP -1.22454454 -1.914423633 -4.475104325 1.33E-05 4.24E-05 2.595376422 DOWN FAM35BP

CH507_42P11.8 -1.352849661 3.00093981 -4.474216114 1.34E-05 4.25E-05 2.127872003 DOWN CH507-42P11.8

CTD_2561J22.2 1.010592731 -3.791032304 4.471343329 1.36E-05 4.30E-05 2.618428023 UP CTD-2561J22.2

BX470102.3 1.035204552 -4.810374092 4.470118832 1.36E-05 4.32E-05 2.618799364 UP BX470102.3

RP5_875H18.4 -1.22486551 -2.490616389 -4.467689272 1.38E-05 4.37E-05 2.5906271 DOWN RP5-875H18.4

RP11_81A22.5 -1.118244294 -2.204896266 -4.463801127 1.40E-05 4.43E-05 2.563653707 DOWN RP11-81A22.5

PNMA3 1.805804655 -0.326044781 4.463074311 1.40E-05 4.44E-05 2.393805394 UP PNMA3

RP11_353N14.5 1.417107293 -2.712628435 4.462895494 1.40E-05 4.45E-05 2.549102633 UP RP11-353N14.5

HLA_DPB2 1.315910496 -1.426239967 4.461884928 1.41E-05 4.46E-05 2.482282935 UP HLA-DPB2

FRMD3 -1.142429109 1.330484068 -4.461145367 1.42E-05 4.48E-05 2.281569857 DOWN FRMD3

ANO4 1.27849637 -2.781285624 4.460391459 1.42E-05 4.49E-05 2.54421083 UP ANO4

CDX2 1.472986979 -4.624318324 4.460160395 1.42E-05 4.49E-05 2.577794046 UP CDX2
RNF17 1.541329788 -5.108260728 4.457743715 1.44E-05 4.54E-05 2.568254472 UP RNF17
C6orf183 1.592194017 -2.275727173 4.4571755 1.44E-05 4.54E-05 2.505101113 UP C6orf183
CTAG2 2.643432499 -3.930608749 4.456355306 1.44E-05 4.56E-05 2.538703385 UP CTAG2
SHISA3 -1.763195484 -1.289243238 -4.456245509 1.44E-05 4.56E-05 2.489864516 DOWN SHISA3
RP1_232L22__B.1 -1.077753923 -1.54365494 -4.453433602 1.46E-05 4.61E-05 2.486568043 DOWN RP1-232L22--B.1
CHST4 2.236797537 -2.581096204 4.452439172 1.47E-05 4.63E-05 2.487476045 UP CHST4
CABYR 1.556287508 0.237010734 4.448400107 1.49E-05 4.71E-05 2.289741437 UP CABYR
RP11_389O22.1 -1.181637242 -2.435095381 -4.445297348 1.51E-05 4.76E-05 2.499316436 DOWN RP11-389O22.1
SLC16A9 1.81935525 -0.505631445 4.443847466 1.52E-05 4.79E-05 2.334856406 UP SLC16A9
RP11_7K24.3 1.299431639 -4.062888573 4.443266647 1.53E-05 4.80E-05 2.50761287 UP RP11-7K24.3
ASPHD1 1.800649507 0.062617802 4.442577057 1.53E-05 4.81E-05 2.278537746 UP ASPHD1
RP11_384O8.1 1.165879488 -4.718373292 4.441356615 1.54E-05 4.83E-05 2.503788208 UP RP11-384O8.1
RP11_407N17.3 -1.137626951 -1.687226596 -4.440307882 1.54E-05 4.85E-05 2.443185236 DOWN RP11-407N17.3
ADAM23 1.516938688 -0.827268998 4.439152139 1.55E-05 4.87E-05 2.346153912 UP ADAM23
RP11_163F15.1 -1.286785225 -5.643171099 -4.439050654 1.55E-05 4.88E-05 2.492507998 DOWN RP11-163F15.1
SERPINA3 -1.336518771 3.391968514 -4.438341465 1.56E-05 4.89E-

05	1.928408396	DOWN	SERPINA3
RP5_875H18.10	-1.051274399	-0.803637308	-4.437632945	1.56E-05	4.90E-05	2.372891676	DOWN	RP5-875H18.10
BARX2	1.379421617	-3.947786797	4.436199625	1.57E-05	4.93E-05	2.47728556	UP	BARX2
S100P	2.55214418	1.648554832	4.435389399	1.58E-05	4.95E-05	2.07476469	UP	S100P
FAM131C	1.652194139	-3.062020714	4.434604018	1.58E-05	4.96E-05	2.443586336	UP	FAM131C
LYZL1	-1.083049953	-4.186711887	-4.432834295	1.59E-05	4.99E-05	2.471044472	DOWN	LYZL1
KRTDAP	1.184320814	-5.264832548	4.43195612	1.60E-05	5.01E-05	2.465920057	UP	KRTDAP
GRIA3	-1.932332406	-0.26184147	-4.430417453	1.61E-05	5.04E-05	2.318901964	DOWN	GRIA3
H19	2.007612428	5.135472972	4.429542924	1.62E-05	5.06E-05	1.732757781	UP	H19
HES5	-1.104936446	-3.299751344	-4.429225155	1.62E-05	5.06E-05	2.453441581	DOWN	HES5
TM4SF4	-1.107672097	8.356763043	-4.428311364	1.62E-05	5.08E-05	1.705696971	DOWN	TM4SF4
ADAMTS17	-1.487436829	1.656169567	-4.427542193	1.63E-05	5.10E-05	2.118168114	DOWN	ADAMTS17
ALX1	1.255349102	-5.697549759	4.426888182	1.63E-05	5.11E-05	2.444667658	UP	ALX1
SLC7A9	-1.438771651	3.14609427	-4.426191369	1.64E-05	5.12E-05	1.917112725	DOWN	SLC7A9
RP11_753B14.1	-1.350599103	-3.662864852	-4.425545178	1.64E-05	5.13E-05	2.440849573	DOWN	RP11-753B14.1
NAT8	-1.44861012	4.330711057	-4.425508009	1.64E-05	5.13E-05	1.771737183	DOWN	NAT8
BX322559.3	-1.351572329	-5.844235441	-4.423658034	1.66E-05	5.17E-05	2.430159809	DOWN	BX322559.3

NEFL 1.645311562-4.380405733 4.423302604 1.66E-05 5.18E-05 2.429099407 UP NEFL

DNAH6-1.412203142 -0.069904363 -4.421387276 1.67E-05 5.22E-05 2.259292405 DOWN DNAH6

CTC_378H22.2 1.525438726 -2.142337941 4.41746121 1.70E-05 5.30E-05 2.342011337UP CTC-378H22.2

CCT7P2 1.151095452 -5.602377248 4.416169762 1.71E-05 5.33E-05 2.402536244 UP CCT7P2

NRXN3 1.503744788 -0.115276625 4.4161155141.71E-05 5.33E-05 2.193905975 UP NRXN3

ARL9 1.002970883 -3.772501113 4.415812425 1.71E-05 5.33E-05 2.397114596UP ARL9

ATP10B1.781944583 -3.354785453 4.415584289 1.71E-05 5.34E-05 2.37512229 UP ATP10B

HEPACAM2 1.247931847 -4.853303371 4.415309885 1.72E-05 5.34E-05 2.400286923 UP HEPACAM2

RP11_731J8.2 1.056908503 -5.118619584 4.41110643 1.75E-05 5.43E-05 2.383709677 UP RP11-731J8.2

FAM19A1 -1.014114261 -4.561908296 -4.41006452 1.75E-05 5.45E-05 2.380854063 DOWN FAM19A1

HOXC_AS1 1.465756278 -5.00545055 4.406874389 1.78E-05 5.52E-05 2.366394352 UP HOXC-AS1

AL589743.1 1.152714134 -3.202207693 4.406606187 1.78E-05 5.53E-05 2.345712895 UP AL589743.1

SEMA3A 1.449120882 -1.050586545 4.406146821 1.78E-05 5.53E-05 2.232833619 UP SEMA3A

IFITM10 1.075473582 2.58431437 4.404941243 1.79E-05 5.56E-05 1.851993476 UP IFITM10

AC002511.2 1.143655061 -5.312240302 4.404632767 1.79E-05 5.57E-05 2.357606696 UP AC002511.2

LINC01436 1.894103647 -2.30168109 4.399745887 1.83E-05 5.68E-05 2.273051759 UP LINC01436

RP11_436K8.1 1.155787314 -4.969696137 4.399221596 1.83E-05

5.69E-05 2.33677025 UP RP11-436K8.1

RP11_21L23.3 -1.224221143 -1.789058448 -4.397966081 1.84E-05 5.72E-05 2.281522768 DOWN RP11-21L23.3

LINC01587 1.555983975 -4.093774992 4.39586203 1.86E-05 5.76E-05 2.31793306 UP LINC01587

RPL39L 1.292133895 1.325681597 4.395566722 1.86E-05 5.77E-05 1.968518147 UP RPL39L

HOXA11 1.53250136 -4.434800042 4.393489548 1.88E-05 5.82E-05 2.312106109 UP HOXA11

FXYD1 -2.031540698 1.994485919 -4.393454418 1.88E-05 5.82E-05 1.955518883 DOWN FXYD1

TRIM31 1.753451568 0.97931512 4.391714715 1.89E-05 5.86E-05 1.983750853 UP TRIM31

RP11_325P15.1 -1.276752881 -4.44514355 -4.388713797 1.92E-05 5.93E-05 2.296429231 DOWN RP11-325P15.1

C10orf126 -1.494769803 -3.836552715 -4.388530165 1.92E-05 5.93E-05 2.294940459 DOWN C10orf126

GLUL -1.349437195 9.988371999 -4.387971222 1.92E-05 5.94E-05 1.58228367 DOWN GLUL

CCDC144NL 1.03216217 -5.782989114 4.386117153 1.94E-05 5.98E-05 2.283352133 UP CCDC144NL

ABCA17P 1.031130552 -2.771474692 4.381049223 1.98E-05 6.10E-05 2.232811861 UP ABCA17P

GALNT12 1.260278315 -1.413603487 4.380874479 1.98E-05 6.10E-05 2.159957423 UP GALNT12

HSPA6 1.026440831 1.340827344 4.380694861 1.98E-05 6.11E-05 1.912213046 UP HSPA6

TNFAIP8L3 1.077050018 0.837551288 4.378538506 2.00E-05 6.16E-05 1.959292049 UP TNFAIP8L3

KIAA0319 1.057926926 -2.404170019 4.378230341 2.00E-05 6.17E-05 2.207888655 UP KIAA0319

SVOPL 1.107210435 -5.127120625 4.377898035 2.00E-05 6.17E-05 2.252608396 UP SVOPL

PDX1 2.461692956 -2.044771672 4.375413947 2.03E-05 6.24E-05 2.153430179 UP PDX1

CTD_2245F17.3 1.029976676 -3.035024074 4.374242484 2.04E-05 6.26E-05 2.214889358 UP CTD-2245F17.3

RP4_655J12.4 1.078158162 -3.492780421 4.374036462 2.04E-05 6.27E-05 2.22571216 UP RP4-655J12.4

RP11_44N21.1 1.416924031 -1.652672773 4.374003834 2.04E-05 6.27E-05 2.145658674 UP RP11-44N21.1

CTD_3064H18.4 1.050849472 -5.058948827 4.373506936 2.04E-05 6.28E-05 2.235529605 UP CTD-3064H18.4

FSIP1 1.044320507 -2.761993501 4.37328504 2.04E-05 6.28E-05 2.202777981 UP FSIP1

KEL 1.553509153 -1.949535637 4.37288854 2.05E-05 6.29E-05 2.156274776 UP KEL

ZNF295_AS1 -1.035240796 -3.154157829 -4.372765113 2.05E-05 6.30E-05 2.228963483 DOWN ZNF295-AS1

CLCNKB 1.123688004 -4.736663838 4.371367399 2.06E-05 6.33E-05 2.227161082 UP CLCNKB

NKX2_5 1.223429608 -5.513873422 4.371359838 2.06E-05 6.33E-05 2.225930352 UP NKX2-5

TTLL6 1.24361159 -2.918186218 4.368464977 2.08E-05 6.40E-05 2.185038651 UP TTLL6

IL33 -1.033624105 2.376741501 -4.36690593 2.10E-05 6.44E-05 1.773428397 DOWN IL33

LYVE1 -1.058430856 1.153811071 -4.365832593 2.11E-05 6.47E-05 1.918878313 DOWN LYVE1

AC098830.1 -1.18585966 -5.375770108 -4.364534696 2.12E-05 6.50E-05 2.199487304 DOWN AC098830.1

CALML3 -1.423969464 -3.731185422 -4.363174989 2.13E-05 6.54E-05 2.19499158 DOWN CALML3

TEKT5 -1.120299226 -2.505593586 -4.362814889 2.13E-05 6.54E-05 2.175453389 DOWN TEKT5

RP11_528A4.2 1.27805516 -3.813899032 4.362095474 2.14E-05 6.56E-

05 2.183369325 UP RP11-528A4.2

VSIG1 1.712663178 -1.786577833 4.3605611852.15E-05 6.60E-05 2.096004193 UP VSIG1

IGLON5 1.385100621 -1.739817452 4.3605177112.15E-05 6.60E-05 2.097416996 UP IGLON5

EFNA2 -1.231248744 0.172543274 -4.357521943 2.18E-05 6.68E-05 1.981662428 DOWN EFNA2

TENM2 -2.340867853 0.036517777 -4.352767976 2.22E-05 6.80E-05 1.994676867 DOWN TENM2

HOXC4 1.214745794 -2.557597801 4.35079838 2.24E-05 6.85E-05 2.102304698 UP HOXC4

MCF2L2 1.289307823 -1.963519855 4.349663976 2.25E-05 6.88E-05 2.070409889 UP MCF2L2

ZNF215 1.349613965 -2.270458054 4.345427972 2.29E-05 6.99E-05 2.067767073 UP ZNF215

LINC00535 -1.41501585 -1.592407497 -4.344904558 2.30E-05 7.01E-05 2.063545204 DOWN LINC00535

CTD_2527I21.5 -1.354323694 -3.131766987 -4.344369844 2.30E-05 7.02E-05 2.116808268DOWN CTD-2527I21.5

RP1_232P20.1 -1.977118183 -2.909275423 -4.343018219 2.32E-05 7.05E-05 2.105293973 DOWN RP1-232P20.1

RXRG -1.813028906 -2.001434811 -4.341072435 2.33E-05 7.11E-05 2.072031243 DOWN RXRG

SLC8A3 -1.038553195 -3.585312561 -4.340420349 2.34E-05 7.12E-05 2.105580065 DOWN SLC8A3

RAB27B 1.504662167 0.206714058 4.340377071 2.34E-05 7.12E-05 1.866157715 UP RAB27B

NGFRAP1 1.083367977 5.295121857 4.339254717 2.35E-05 7.15E-05 1.355148322 UP NGFRAP1

RAB9B 1.024965724 -2.599589734 4.338653973 2.36E-05 7.17E-05 2.060663461 UP RAB9B

LINC00943 1.092932578 -4.417195948 4.338358165 2.36E-05 7.18E-05 2.097367701 UP LINC00943

TTTY14 -2.198557262 -2.140328431 -4.338339583 2.36E-05 7.18E-05 2.06604055 DOWN TTTY14
L1TD1 1.049446104 -4.658217939 4.338041684 2.36E-05 7.19E-05 2.096723348 UP L1TD1
RP11_70C1.1 -1.630933064 -2.907692052 -4.336856564 2.38E-05 7.22E-05 2.082932536 DOWN RP11-70C1.1
WSCD2 -1.123703177 -4.099282954 -4.336787852 2.38E-05 7.22E-05 2.092712211 DOWN WSCD2
RP11_387H17.4 -1.04390873 -2.538854514 -4.334827953 2.40E-05 7.27E-05 2.066830977 DOWN RP11-387H17.4
SLN 1.153699831 -5.45902003 4.333066907 2.41E-05 7.32E-05 2.076247979 UP SLN
TNFRSF9 1.257779201 -0.569437799 4.331596131 2.43E-05 7.36E-05 1.903319643 UP TNFRSF9
PLD4 1.032534286 -0.265465601 4.331553865 2.43E-05 7.36E-05 1.880975738 UP PLD4
ATOH7 -1.002214404 -2.628192835 -4.331376299 2.43E-05 7.36E-05 2.056143795 DOWN ATOH7
LINC00668 1.678994215 -4.729380716 4.330892224 2.44E-05 7.38E-05 2.067294738 UP LINC00668
CTD_2342N23.3 -1.513759711 -1.373464346 -4.33034526 2.44E-05 7.39E-05 1.994853221 DOWN CTD-2342N23.3
PCDHA5 1.129670984 -4.964383132 4.329386868 2.45E-05 7.42E-05 2.062838158 UP PCDHA5
CXorf67 1.352688625 -5.540436838 4.327726513 2.47E-05 7.46E-05 2.054989623 UP CXorf67
RP3_522D1.1 1.081816713 -4.047829332 4.325450638 2.49E-05 7.53E-05 2.044769996 UP RP3-522D1.1
CTD_2297D10.2 1.700741103 -4.289469495 4.324645075 2.50E-05 7.55E-05 2.039992756 UP CTD-2297D10.2
RP11_431J24.2 -1.748999558 -2.998291842 -4.323875375 2.51E-05 7.57E-05 2.033486744 DOWN RP11-431J24.2
AL163953.2 1.364359195 -3.801970258 4.323396902 2.51E-05 7.59E-

05 2.030592835 UP AL163953.2

MPPED2 -1.16388771 -0.526257267 -4.323023068 2.52E-05 7.60E-05 1.902650451 DOWN MPPED2

ARF4P2 1.070589898 -4.909317268 4.320969789 2.54E-05 7.66E-05 2.030185237 UP ARF4P2

RP11_54C4.2 -1.005326804 -5.356132443 -4.32064293 2.54E-05 7.67E-05 2.028296932 DOWN RP11-54C4.2

CDH18 1.218162562 -5.586981421 4.31970409 2.55E-05 7.69E-05 2.023779865 UP CDH18

GGH -1.110558701 6.700344958 -4.318784044 2.56E-05 7.72E-05 1.253540827 DOWN GGH

RP11_96H17.3 1.024796029 -5.860973745 4.31398976 2.61E-05 7.87E-05 2.000854537 UP RP11-96H17.3

BMPER -1.375583176 -2.142993864 -4.312299383 2.63E-05 7.92E-05 1.964571043 DOWN BMPER

PROM2 1.601417231 -1.751478717 4.310215369 2.65E-05 7.98E-05 1.899308125 UP PROM2

RP11_923I11.6 1.233157726 0.58389225 4.309981582 2.65E-05 7.99E-05 1.71161778 UP RP11-923I11.6

ZNF365 1.070868664 -3.900857817 4.309846163 2.66E-05 7.99E-05 1.982669834 UP ZNF365

KIAA1211 1.490473475 -1.395939032 4.308402162 2.67E-05 8.03E-05 1.871807908 UP KIAA1211

HOXD11 1.134885614 -5.686936027 4.307858719 2.68E-05 8.05E-05 1.977532113UP HOXD11

IL1B 1.023883608 -0.631356553 4.306824262 2.69E-05 8.08E-05 1.815089548 UP IL1B

LINC00504 -1.258133164 -2.120176691 -4.306391675 2.69E-05 8.10E-05 1.940017982 DOWN LINC00504

TMEM54 1.037984507 2.665259505 4.30506392 2.71E-05 8.14E-05 1.447691852 UP TMEM54

AC012499.1 -1.116311418 -5.243686792 -4.304148213 2.72E-05 8.16E-05 1.964467588 DOWN AC012499.1

MLPH -1.056305073 4.248464203 -4.30261947 2.74E-05 8.21E-05 1.282267288 DOWN MLPH

PRPH 1.077590129 -4.973811932 4.300441034 2.76E-05 8.28E-05 1.95039072 UP PRPH

SCG2 1.371395297 -2.911148371 4.297064869 2.80E-05 8.39E-05 1.903782084 UP SCG2

C2orf70 1.336738078 -4.778375598 4.294583096 2.83E-05 8.47E-05 1.927251319 UP C2orf70

RP11_1430O6.1 -1.150118776 -4.588140546 -4.293602105 2.84E-05 8.50E-05 1.92491608 DOWN RP11-1430O6.1

PCDHA3 1.309645543 -4.131973128 4.291510247 2.86E-05 8.57E-05 1.912490065 UP PCDHA3

LINC01010 -1.309274552 -2.286937988 -4.291246181 2.87E-05 8.57E-05 1.888777222 DOWN LINC01010

CACNA1E 1.605446403 -3.181136772 4.290668017 2.87E-05 8.59E-05 1.882683802 UP CACNA1E

AC241377.2 1.240371207 -3.452303621 4.288783581 2.89E-05 8.66E-05 1.890305343 UP AC241377.2

BANK1 1.377485299 -2.167389216 4.288495088 2.90E-05 8.66E-05 1.840221308 UP BANK1

FGFR2 2.037421077 3.804130292 4.288152766 2.90E-05 8.67E-05 1.265742603 UP FGFR2

CDKN1C 1.103219006 1.870115114 4.285907298 2.93E-05 8.75E-05 1.472891091 UP CDKN1C

COL4A5 1.669679035 1.0399668 4.285834694 2.93E-05 8.75E-05 1.564887129 UP COL4A5

LINC01239 -1.206920884 -1.737177379 -4.28424254 2.95E-05 8.80E-05 1.833900342 DOWN LINC01239

TMPRSS9 -1.209665195 1.085020656 -4.282937013 2.96E-05 8.84E-05 1.602798077 DOWN TMPRSS9

LYG2 1.000597647 -3.94247664 4.281740232 2.98E-05 8.88E-05 1.874711626 UP LYG2

BCL2A1 1.029926178 -0.187694098 4.28044227 3.00E-05 8.92E-05

1.675157818 UP BCL2A1

SMCO2 1.182718168 -3.493092833 4.280247839 3.00E-05 8.92E-05 1.859134537 UP SMCO2

LEF1_AS1 1.062229069 -3.403407669 4.278778083 3.02E-05 8.98E-05 1.85259423 UP LEF1-AS1

BPIFA1 1.435951504 -5.474386973 4.278433692 3.02E-05 8.99E-05 1.863848317 UP BPIFA1

GAGE2A 1.283421104-5.750707736 4.277739127 3.03E-05 9.01E-05 1.860630307 UP GAGE2A

TM4SF19 1.295461157-3.219579853 4.277644605 3.03E-05 9.01E-05 1.839607791 UP TM4SF19

CD22 1.008955104 0.060855373 4.276789166 3.04E-05 9.04E-05 1.638392127 UP CD22

COCH 1.649247288 -0.287715608 4.276780736 3.04E-05 9.04E-05 1.659099034 UP COCH

GLULP3 -1.146485863 -5.148239533 -4.275010955 3.06E-05 9.10E-05 1.852022093 DOWN GLULP3

GJB6 1.525903091 -4.383943119 4.274526433 3.07E-05 9.11E-05 1.847672179 UP GJB6

ATP1A3 1.241496598 -2.380098874 4.271097394 3.11E-05 9.24E-05 1.785046542 UP ATP1A3

RP11_359K18.4 -1.000454512 -3.136552123 -4.267230963 3.16E-05 9.38E-05 1.818092584 DOWN RP11-359K18.4

PMEPA1 1.223238273 3.293506546 4.264130518 3.20E-05 9.49E-05 1.211447082UP PMEPA1

PTPN131.259972324 -0.038010875 4.262648958 3.22E-05 9.54E-05 1.589670026 UP PTPN13

KB_1615E4.3 1.198876538 -4.510033745 4.262543229 3.22E-05 9.55E-05 1.803441351 UP KB-1615E4.3

PRKAR2B 1.040433266 -0.286547368 4.261732856 3.23E-05 9.57E-05 1.610958519 UP PRKAR2B

FAM153B 1.493388393 -3.228432467 4.258715334 3.27E-05 9.69E-05 1.763561101UP FAM153B

MROH3P 1.249259814 -4.750248449 4.2568114543.30E-05 9.76E-05 1.781683043 UP MROH3P

AP006285.6 -1.673930205 -2.342036666 -4.256717626 3.30E-05 9.76E-05 1.757682894 DOWN AP006285.6

KCNK2 1.53930227 -4.603784944 4.256350892 3.30E-05 9.77E-05 1.778837395 UP KCNK2

DPP10_AS1 1.848767233 -4.946174035 4.253964999 3.34E-05 9.86E-05 1.769274229 UP DPP10-AS1

GRIA2 1.178725073 -5.249847782 4.25390777 3.34E-05 9.86E-05 1.77021453 UP GRIA2

SLC14A2 -1.592113942 -3.152651141 -4.253360974 3.34E-05 9.88E-05 1.763671274 DOWN SLC14A2

ADAMDEC1 1.452473363 -1.156532192 4.252565868 3.36E-05 9.91E-05 1.63945025 UP ADAMDEC1

BSPRY 1.441821424 0.146565915 4.250921252 3.38E-05 9.97E-05 1.523605095 UP BSPRY

RP13_580F15.2 -1.083005675 -2.765902435 -4.250019881 3.39E-05 0.00010005 1.745017854 DOWN RP13-580F15.2

HMX2 1.035983926 -5.764378112 4.24977283 3.39E-05 0.000100132 1.75309071 UP HMX2

ATP2B2-1.330155184 5.338820485 -4.24876831 3.41E-05 0.00010051 1.006291387 DOWN ATP2B2

RP11_38L15.8 -1.14531279 0.118749932-4.248182811 3.42E-05 0.000100725 1.558431536 DOWN RP11-38L15.8

RP11_326C3.13 -1.173031495 -1.984936003 -4.248024506 3.42E-05 0.000100777 1.707273307 DOWN RP11-326C3.13

MYOCD -1.193443987 -1.517307763 -4.244742911 3.46E-05 0.000102041 1.668352317 DOWN MYOCD

GRIP1 1.286993988 -3.101104658 4.244346162 3.47E-05 0.000102155 1.708737924 UP GRIP1

RP4_710M16.2 -1.291545488 1.096840612 -4.241499712 3.51E-05 0.000103266 1.442928402 DOWN RP4-710M16.2

SUCNR1 -1.066653462 1.062323733 -4.240105002 3.53E-05

0.000103827 1.437299133 DOWN SUCNR1

ART4 -1.303476917 3.202002403 -4.24002171 3.53E-05 0.000103849

1.174200936 DOWN ART4

RP11_109J4.1 1.386435926 -4.502781938 4.239976924 3.53E-05

0.000103855 1.716153406 UP RP11-109J4.1

TACR2 1.002577944 -2.439558005 4.239533064 3.54E-05

0.000104017 1.67016925 UP TACR2

FCGR1A 1.057556196 -0.265540882 4.239242993 3.54E-05

0.0001041141.521879491 UP FCGR1A

RP11_501C14.5 1.111207152-5.778454588 4.238352563 3.55E-05

0.000104452 1.709200024 UP RP11-501C14.5

LGI1 -1.312589483 -4.353324406 -4.235716448 3.59E-05

0.000105484 1.702028249 DOWN LGI1

AC005307.3 1.497857812 -4.84935204 4.234804696 3.61E-05

0.000105849 1.696731037 UP AC005307.3

CFAP61 1.062591565 -4.344858112 4.234754056 3.61E-05

0.000105857 1.696621036 UP CFAP61

TCAM1P 1.523516652 -4.149820059 4.234675181 3.61E-05

0.000105878 1.692211307UP TCAM1P

SERPINA7 -2.284309375 5.78346439 -4.231253676 3.66E-05

0.000107317 0.938527346 DOWN SERPINA7

SLC8A2 1.104985767 -3.953887339 4.230561622 3.67E-05

0.000107592 1.677321981 UP SLC8A2

RP4_595K12.2 1.242658725 -5.218250655 4.229087782 3.69E-05

0.000108223 1.675043413 UP RP4-595K12.2

CREB3L1 1.274245879 1.24130854 4.226123899 3.74E-05

0.000109409 1.316417236 UP CREB3L1

RASD1 -1.318073461 4.307812189 -4.225837794 3.74E-05

0.000109522 0.985950861 DOWN RASD1

ZDBF2 1.238644285 0.252421371 4.225693849 3.74E-05

0.000109572 1.419714282 UP ZDBF2

LINC00323 1.047483286 -4.782081096 4.223984232 3.77E-05

0.0001102791.656289956 UP LINC00323

VDR 1.014523252 1.063098867 4.221905202 3.80E-05
0.000111183 1.324249482 UP VDR
RAG1 -1.069852224 -1.048411591 -4.21987058 3.83E-05 0.000112047
1.540231654 DOWN RAG1
WNT10B 1.076865447 -2.712093984 4.219704672 3.83E-05
0.000112083 1.603939152 UP WNT10B
MYBPC3 1.007275863 -3.56586352 4.217822664 3.86E-05
0.000112868 1.623372726 UP MYBPC3
GPC5 1.829069093 -1.936710231 4.215679159 3.90E-05
0.000113794 1.540085925 UP GPC5
KRT17 1.48731036 -0.051267215 4.213445637 3.93E-05 0.000114728
1.397411089 UP KRT17
ASIP -1.069373282 -2.570207298 -4.212387075 3.95E-05
0.000115191 1.595434271 DOWN ASIP
LRRC52 -1.409980126 -5.067106427 -4.210147759 3.98E-05
0.000116194 1.603212216 DOWN LRRC52
TOX 1.357791148 0.073420581 4.208960038 4.00E-05 0.00011671
1.370728346 UP TOX
TCP10L -1.400086802 0.585814089 -4.207883544 4.02E-05
0.000117175 1.367301767 DOWN TCP10L
CPXM1 1.114185003 0.759102204 4.206626213 4.04E-05 0.000117728
1.296675755 UP CPXM1
FOXD1 1.252265945 -4.76018739 4.20419576 4.08E-05 0.000118758
1.580434094 UP FOXD1
TIFAB 1.079049049 -2.387646678 4.202196854 4.11E-05 0.00011966
1.523081753 UP TIFAB
RP5_875O13.7 -1.055967658 -3.993111693 -4.201828352 4.12E-05
0.000119824 1.572525499 DOWN RP5-875O13.7
TLX2 1.262475491 -4.804147715 4.201307982 4.13E-05
0.000120046 1.569442245 UP TLX2
AC003090.1 -1.634221788 -3.597448014 -4.199028439 4.17E-05
0.000121036 1.560144127 DOWN AC003090.1
MYOM2 -1.19429609 -0.042021337 -4.196548891 4.21E-05

0.000122221 1.376128914 DOWN MYOM2
RP4_604G5.3 -1.140726191 -4.438335971 -4.193640761 4.26E-05
0.0001236 1.541699353 DOWN RP4-604G5.3
GSTM5 -1.653267999 -1.398836033 -4.192282386 4.28E-05
0.000124233 1.465568723 DOWN GSTM5
AC004540.5 1.225367391 -2.370010975 4.190313545 4.32E-05
0.000125145 1.475725023 UP AC004540.5
CD19 1.292031079 -2.201032699 4.1895611574.33E-05 0.000125448
1.462983929 UP CD19
LUZP2 -1.709920778 -3.219887772 -4.188540883 4.35E-05
0.000125948 1.516654185 DOWN LUZP2
TRIM72 1.175875004 -3.787293987 4.188435936 4.35E-05
0.000125956 1.513879832 UP TRIM72
RP11_165A20.3 1.067736927 -3.763320846 4.187047363 4.37E-05
0.0001266 1.50919557 UP RP11-165A20.3
C9orf66 1.328973228 -2.566574542 4.1861711014.39E-05 0.000127031
1.464964033 UP C9orf66
SSTR5_AS1 2.478174483 -2.703164749 4.185559403 4.40E-05
0.000127313 1.443815102 UP SSTR5-AS1
DNAH7 1.085285659 -2.647440706 4.184207266 4.42E-05
0.000127939 1.467014374 UP DNAH7
CASC22 -1.340103236 -4.653065114 -4.181205238 4.48E-05
0.000129356 1.49423137 DOWN CASC22
RP5_1050E16.2 -1.267934876 -4.858287108 -4.179037022 4.52E-05
0.000130424 1.485778599 DOWN RP5-1050E16.2
OLFM4 1.819635424 -4.156186049 4.17336028 4.62E-05 0.000133171
1.456374693 UP OLFM4
HENMT1 1.008497819 0.455840606 4.173046221 4.63E-05
0.000133307 1.200914918 UP HENMT1
NPY1R -1.188279479 0.426095383 -4.171668941 4.65E-05
0.000133981 1.240408751 DOWN NPY1R
TMEM25 -1.016777654 2.274456392 -4.169606581 4.69E-05
0.000135028 1.019689428 DOWN TMEM25

RP4_781K5.6 -1.611118896 -2.554689624 -4.169477026 4.69E-05 0.000135082 1.431598372 DOWN RP4-781K5.6

AC007163.2 -1.577755563 -5.289823117 -4.168083375 4.72E-05 0.000135773 1.442569783 DOWN AC007163.2

RP5_884M6.1 1.024770607 -5.65476273 4.167151107 4.74E-05 0.000136248 1.438780024 UP RP5-884M6.1

NNMT -1.75588168 7.073346828 -4.166690427 4.75E-05 0.000136483 0.66622131 DOWN NNMT

RP11_138B4.1 1.130420294 -5.28178886 4.16425618 4.79E-05 0.000137655 1.42872788 UP RP11-138B4.1

PLGLB2 -1.391803511 0.428118655 -4.163617306 4.80E-05 0.000137958 1.213861484 DOWN PLGLB2

RHCG -1.879203343 -1.266917881 -4.163495074 4.81E-05 0.000138009 1.350039864 DOWN RHCG

RP11_197N18.7 -1.162932365 -5.391914999 -4.162075075 4.83E-05 0.000138745 1.42016247 DOWN RP11-197N18.7

RSPO2 -1.934704665 -3.197705746 -4.161928452 4.84E-05 0.00013881 1.414748626 DOWN RSPO2

KCNU1 -2.312999016 -3.484410356 -4.160029055 4.87E-05 0.000139785 1.409390116 DOWN KCNU1

MAATS1 -1.147488513 -0.282842198 -4.159294046 4.89E-05 0.000140161 1.254261204 DOWN MAATS1

MSC 1.37535345 1.650174877 4.15926805 4.89E-05 0.000140161 1.009967059 UP MSC

FBLN2 -1.212500731 3.362213294 -4.157180454 4.93E-05 0.000141201 0.82990242 DOWN FBLN2

NELL1 1.218126945 -5.660979259 4.155757503 4.96E-05 0.000141939 1.395595067 UP NELL1

SOBP 1.185587573 1.677208185 4.155685026 4.96E-05 0.000141963 0.993802463 UP SOBP

SEMA6A 1.090565509 2.950865569 4.1553831 4.97E-05 0.000142066 0.833502508 UP SEMA6A

DPYSL5 1.190612159 -5.268132535 4.152670643 5.02E-05

0.000143512 1.38497724 UP DPYSL5

RP11_1134I14.8 1.053332717 -3.452871807 4.151294656 5.05E-05

0.000144268 1.368021772 UP RP11-1134I14.8

TDGF1P3 -1.117115255 -5.469008198 -4.149167485 5.09E-05

0.000145412 1.371247974 DOWN TDGF1P3

LY86_AS1 -1.272550804 -4.79026499 -4.146787004 5.14E-05

0.000146678 1.364171589 DOWN LY86-AS1

R3HDML -1.095305517 -4.780083102 -4.145529882 5.16E-05

0.000147363 1.359629965 DOWN R3HDML

TNXB -1.098977244 2.093139385 -4.144313675 5.19E-05

0.000148045 0.950491793 DOWN TNXB

HIST1H1C -1.005961918 6.304407128 -4.144191176 5.19E-05

0.000148099 0.579664564 DOWN HIST1H1C

NR0B2 -1.295009862 4.985847034 -4.143181706 5.21E-05

0.000148626 0.616853299 DOWN NR0B2

RP11_10B2.1 -1.005466074 -2.872569594 -4.142271704 5.23E-05

0.000149131 1.3379757 DOWN RP11-10B2.1

CR1L 1.167923153 -4.393537103 4.138383316 5.31E-05

0.000151281 1.331015695 UP CR1L

SLCO4A1 1.20374505 -0.616411689 4.136922738 5.34E-05

0.000152128 1.158792472 UP SLCO4A1

PPP1R3G -1.606422597 2.962477897 -4.135978906 5.36E-05

0.000152664 0.815537582 DOWN PPP1R3G

MLK4 1.08682766 2.151910831 4.134393277 5.40E-05 0.000153577

0.856397584 UP MLK4

ANKRD33B1.148397679 -0.274537052 4.132883015 5.43E-05

0.0001544111.117396125UP ANKRD33B

RP11_499F3.2 1.110379078-5.392876251 4.13023955 5.49E-05

0.000155989 1.300500559 UP RP11-499F3.2

TDGF1 -1.915345072 0.657079688 -4.130193191 5.49E-05

0.000155999 1.074880744 DOWN TDGF1

CHRNA7 1.069951258 -4.919977934 4.128387927 5.53E-05

0.000157031 1.294443368 UP CHRNA7

MAMDC2 -1.153446919 -1.691667763 -4.128023723 5.54E-05
0.000157202 1.234876838 DOWN MAMDC2
SLC22A9 -1.44715332 4.351103596 -4.127641415 5.55E-05 0.000157423
0.606826084 DOWN SLC22A9
PRRX1 1.029823201 0.746718678 4.127432867 5.55E-05
0.000157535 0.999450921 UP PRRX1
RP11_10A14.4 -1.207939092 -0.087191261 -4.127110691 5.56E-05
0.000157718 1.116693545 DOWN RP11-10A14.4
FHL5 -1.160315728 -2.18428648 -4.126562888 5.57E-05 0.000158006
1.255729356 DOWN FHL5
SEZ6L2 1.802873484 2.343851826 4.12624667 5.58E-05 0.000158186
0.795084843 UP SEZ6L2
TRPM3 -1.347042816 -1.644017043 -4.122671642 5.66E-05
0.000160203 1.213546878 DOWN TRPM3
DUOXA1 1.246375545 -3.872916642 4.11861156 5.75E-05
0.000162647 1.251492549 UP DUOXA1
RP11_973F15.2 1.099758205 -4.462943603 4.117740647 5.77E-05
0.00016316 1.254077361 UP RP11-973F15.2
CHRM3 -1.207239498 0.463156469 -4.116520176 5.80E-05
0.000163914 1.029002516 DOWN CHRM3
PDZRN4 -1.312247083 -3.687781726 -4.113766551 5.86E-05
0.000165617 1.239549155 DOWN PDZRN4
C1orf186 1.773686875 -0.713871641 4.112780495 5.88E-05
0.000166175 1.068245867 UP C1orf186
GABRB1 1.016554932 -5.554818133 4.110695159 5.93E-05
0.000167469 1.227011857 UP GABRB1
L1CAM 1.258441365 -2.510724578 4.110313002 5.94E-05 0.000167704
1.178865585 UP L1CAM
ERVMER61_1 1.244023494 -5.624974025 4.108828213 5.98E-05
0.000168614 1.219607864 UP ERVMER61-1
NALCN 1.582890971 -1.409485627 4.108470418 5.98E-05
0.000168814 1.107282142 UP NALCN
GUCY2D 1.273866132 -2.131892352 4.107986564 6.00E-05

0.000169091 1.151416172 UP GUCY2D

PCAT18-1.151327371 -4.700975147 -4.107966957 6.00E-05

0.000169091 1.218927927 DOWN PCAT18

CTD_3035D6.2 -1.053228486 -2.345589324 -4.10779932 6.00E-05

0.000169184 1.19220114 DOWN CTD-3035D6.2

SLCO6A1 1.313024992 -5.189102392 4.1071189886.02E-05 0.0001696

1.214129844 UP SLCO6A1

RP11_326C3.2 -1.387905862 0.115925543-4.106765394 6.03E-05

0.000169818 1.026161011DOWN RP11-326C3.2

MEOX2_AS1 -1.46586527-4.506548426 -4.10595374 6.04E-05

0.000170345 1.211306528DOWN MEOX2-AS1

GAGE1 1.314286077 -5.667365047 4.104874237 6.07E-05

0.000171015 1.204654936 UP GAGE1

KCNJ16 2.072619276 -2.690007357 4.101972799 6.14E-05

0.00017291 1.140363107 UP KCNJ16

AC147651.5 1.618444884 -4.748473143 4.101610724 6.15E-05

0.000173138 1.193004598 UP AC147651.5

NKAPL -1.078721254 -2.710838988 -4.101366294 6.16E-05

0.000173285 1.180869769 DOWN NKAPL

COL16A1 1.10030842 2.4810114044.1008621 6.17E-05 0.00017359

0.686902132 UP COL16A1

EMR3 1.093873851 -4.040236607 4.098105554 6.24E-05

0.000175391 1.178241227 UP EMR3

POSTN 1.448018669 2.628257041 4.09535049 6.30E-05 0.00017723

0.645483607 UP POSTN

TRIM54 1.635473305 -2.482678116 4.094058213 6.34E-05

0.000178054 1.108852519 UP TRIM54

BRDT 1.268460876 -5.439300313 4.093678357 6.35E-05 0.00017828

1.163590482 UP BRDT

CTC_367J11.1 -1.305987156 -2.734603454 -4.093313302 6.35E-05

0.000178474 1.151255559 DOWN CTC-367J11.1

RP11_279F6.1 -1.589150054 1.733062898 -4.093048463 6.36E-05

0.00017864 0.81784301 DOWN RP11-279F6.1

GIPC2 -1.138423962 2.732807364 -4.092467576 6.38E-05 0.00017903 0.668060136 DOWN GIPC2

RP5_907C10.3 -1.524151103 -1.338416463 -4.090656463 6.42E-05 0.000180105 1.07716383 DOWN RP5-907C10.3

LL22NC03_N14H11.1 1.095383762 -3.909064977 4.090281513 6.43E-05 0.000180351 1.147600012 UP LL22NC03-N14H11.1

CXCL8 1.671714952 2.003065478 4.081506149 6.66E-05 0.000186272 0.669980828 UP CXCL8

TMEM156 1.339916877 0.583733457 4.077991839 6.75E-05 0.000188673 0.824857888 UP TMEM156

LEFTY1 1.42420137 -1.797383497 4.076305497 6.80E-05 0.000189864 1.014130924 UP LEFTY1

RP11_276H19.2 1.519189005 -4.657432255 4.076143017 6.80E-05 0.000189964 1.098413144 UP RP11-276H19.2

RP11_93B14.4 -1.053400276 -5.232947072 -4.075426959 6.82E-05 0.000190478 1.09672295 DOWN RP11-93B14.4

SMR3B -1.086757547 -6.093818251 -4.071803274 6.92E-05 0.000192992 1.078635196 DOWN SMR3B

TNNC1 1.35635737 -0.896614768 4.069803145 6.97E-05 0.000194472 0.92669205 UP TNNC1

MGC39584 1.92673728 -4.289600834 4.06890017 7.00E-05 0.000195119 1.066220947 UP MGC39584

LAMA1 1.704225686 -1.897374021 4.068729199 7.00E-05 0.000195228 0.986706103 UP LAMA1

XKR4 1.049034951 -5.270694855 4.068397792 7.01E-05 0.00019546 1.070395462 UP XKR4

RP11_368I23.2 1.129224124 -5.262527666 4.067442101 7.04E-05 0.000196127 1.066762708 UP RP11-368I23.2

COL2A1 2.375102337 -2.647991852 4.067272548 7.04E-05 0.000196235 0.998118513UP COL2A1

PRG2 -1.062302478 -3.282330812 -4.066251972 7.07E-05 0.000196979 1.060420409 DOWN PRG2

ACTL8 1.972046881 -3.65100763 4.065534708 7.09E-05 0.00019749

1.037411398UP ACTL8

GALNT3 1.15937881 -0.67845034 4.064670187 7.11E-05 0.000198116 0.895162411UP GALNT3

LA16c_312E8.4 1.358719655 -2.989528749 4.063765952 7.14E-05 0.000198729 1.021491149UP LA16c-312E8.4

RP5_1031D4.3 1.01807116 -3.537983484 4.061923537 7.19E-05 0.000200081 1.036669428 UP RP5-1031D4.3

STAC2 1.173322891 -4.710126467 4.059190375 7.27E-05 0.000202124 1.036646626 UP STAC2

CILP2 1.272772003 -1.457238166 4.056120253 7.36E-05 0.000204481 0.919810444 UP CILP2

SPDEF 1.637149796 -2.836321737 4.055156582 7.39E-05 0.000205161 0.978656326 UP SPDEF

RP1_127D3.4 -1.212687217 -4.921803857 -4.054813224 7.40E-05 0.00020539 1.02112973 DOWN RP1-127D3.4

GABRA2 1.968872387 -4.426950594 4.050876223 7.51E-05 0.00020837 1.00064943 UP GABRA2

TEKT2 1.027392123 -4.011121292 4.050526455 7.52E-05 0.000208632 1.0019323 UP TEKT2

LINC00704 1.188204208 -3.992896312 4.05022781 7.53E-05 0.000208853 0.999519653 UP LINC00704

SOHLH2 1.62270635 -5.09537571 4.047068224 7.62E-05 0.000211208 0.990865089 UP SOHLH2

RP11_1070N10.7 1.080083023 -5.466052157 4.04639343 7.64E-05 0.0002116730.98857006 UP RP11-1070N10.7

SLC16A12 -1.595160332 0.112223858-4.044147706 7.71E-05 0.000213445 0.796745408 DOWN SLC16A12

CTNNA2 -2.520543554 -1.733161118 -4.042841876 7.75E-05 0.000214391 0.927991244 DOWN CTNNA2

FAM184A -1.000127612 1.890732505 -4.042711268 7.76E-05 0.000214476 0.592376669 DOWN FAM184A

SYN3 1.185394425 -1.704581707 4.041216428 7.80E-05 0.00021566 0.880745274 UP SYN3

LINC00885 -1.645998737 -2.947361273 -4.039999054 7.84E-05 0.000216516 0.957143123 DOWN LINC00885

PVRL4 1.331616965 -2.042671503 4.039562274 7.85E-05 0.000216861 0.893550909 UP PVRL4

FAM230C 1.328259775 -5.500061231 4.039404229 7.86E-05 0.00021697 0.962436376 UP FAM230C

HLA_DQA21.399557669 2.184199681 4.0390114757.87E-05 0.000217254 0.489925208 UP HLA-DQA2

RP11_796E10.1 1.399409903 -4.376838336 4.038956602 7.87E-05 0.000217276 0.960221208 UP RP11-796E10.1

LINC00944 1.420803285 -2.403931445 4.038468843 7.89E-05 0.000217641 0.90340837 UP LINC00944

DLGAP1 1.107926377 -2.680674043 4.037912635 7.90E-05 0.00021809 0.920664362 UP DLGAP1

KRT17P8 -2.049939052 0.396133919 -4.036064509 7.96E-05 0.000219597 0.748834351 DOWN KRT17P8

NRXN2 -1.308343208 1.424581586 -4.03596419 7.96E-05 0.000219657 0.631175899DOWN NRXN2

UBE2U 1.463797046 -4.88723073 4.034422879 8.01E-05 0.000220884 0.944761153UP UBE2U

NPSR1_AS1 1.836426648 -3.201162046 4.032801628 8.06E-05 0.000222161 0.904720683 UP NPSR1-AS1

TUBA3C 1.752636983 -4.877605439 4.032542632 8.07E-05 0.00022236 0.937032336 UP TUBA3C

SLITRK1 1.106005382 -5.759409973 4.032495204 8.07E-05 0.000222376 0.936478552 UP SLITRK1

NEU4 -2.090904383 3.044431627 -4.027072081 8.24E-05 0.000226877 0.412158255 DOWN NEU4

RP11_219G17.4 1.211078223-4.166291843 4.025174507 8.31E-05 0.000228407 0.909005408 UP RP11-219G17.4

FOXE1 1.29657504 -5.04700915 4.022413475 8.40E-05 0.000230698 0.900905782 UP FOXE1

FRRS1 -1.030575443 2.931046436 -4.022323475 8.40E-05

0.000230733 0.376143851 DOWN FRRS1

FAM83A -1.462058776 -2.226085996 -4.02231419 8.40E-05

0.000230733 0.872017332 DOWN FAM83A

VNN2 1.38362431 2.04855776 4.021838479 8.41E-05 0.000231109

0.443662926 UP VNN2

RP11_566K19.6 1.006282951 -4.115520102 4.021090898 8.44E-05

0.000231696 0.894701952 UP RP11-566K19.6

ZNF135 1.062276064 -0.268573425 4.021070439 8.44E-05

0.000231696 0.69996914 UP ZNF135

CBLN4 -1.921324779 -2.329487004 -4.018889227 8.51E-05

0.000233542 0.862794075 DOWN CBLN4

KCNE5 1.372295795 -1.826104541 4.014039139 8.67E-05 0.00023759

0.78579429 UP KCNE5

RP11_707A18.1 -1.337789908 -5.258046785 -4.011829884 8.75E-05

0.000239425 0.861903498 DOWN RP11-707A18.1

PPP1R1B 1.764724782 -2.712404634 4.011538488 8.76E-05

0.000239642 0.810163767 UP PPP1R1B

C9 -2.837430765 4.721454178 -4.011461929 8.76E-05 0.000239686

0.194121705 DOWN C9

LINC00880 -1.04087474 -3.268554319 -4.00970025 8.82E-05 0.000241277

0.85217262 DOWN LINC00880

TIMP3 -1.175544837 0.77781324 -4.004370479 9.01E-05 0.000246027

0.580176911 DOWN TIMP3

WTAPP1 1.258094312 -4.404350505 4.00297888 9.06E-05

0.000247221 0.829077153 UP WTAPP1

HAS2_AS1 1.063671553 -4.67453692 3.999353998 9.18E-05

0.000250558 0.81702794 UP HAS2-AS1

PLN -1.067078845 -1.150087676 -3.997765019 9.24E-05

0.000251966 0.715939642 DOWN PLN

TIGIT 1.021235312 0.278188832 3.996859613 9.27E-05

0.000252825 0.560195873 UP TIGIT

CD3D 1.003811473 1.20630399 3.993729072 9.39E-05 0.000255829

0.449144246 UP CD3D

XXyac_YM21GA2.4 1.485816845 -3.498765415 3.991446586 9.47E-05 0.000257959 0.769291311 UP XXyac-YM21GA2.4

LINC00890 -2.368823654 -1.683096938 -3.991295258 9.48E-05 0.000258051 0.736583321 DOWN LINC00890

RP11_91K9.1 -1.017862928 0.982499583 -3.988545551 9.58E-05 0.000260581 0.497395905 DOWN RP11-91K9.1

GPR1_AS 1.390999719 -5.33640108 3.987451703 9.62E-05 0.00026157 0.772489442 UP GPR1-AS

PIGR -1.871159801 4.58047023 -3.986388848 9.66E-05 0.00026256 0.079224937 DOWN PIGR

WNT5B -1.117541354 1.174693079 -3.984922316 9.71E-05 0.000263937 0.466902529 DOWN WNT5B

LINC00858 1.123050741 -5.561709729 3.982594821 9.80E-05 0.000266086 0.754593799 UP LINC00858

SLC9C2 -1.137760387 -3.91848044 -3.979649684 9.91E-05 0.000268894 0.745867318 DOWN SLC9C2

KCNQ5 1.00179577 -3.689833035 3.976683183 0.000100284 0.000271814 0.727260534 UP KCNQ5

RAP2CP1 -1.062488085 -4.809374955 -3.974848482 0.000100998 0.000273627 0.728632543 DOWN RAP2CP1

RGAG1 -1.400194406 -2.65188983 -3.974479624 0.000101142 0.000273986 0.711814178 DOWN RGAG1

NPIPA3 1.017473517 -3.73949591 3.97314296 0.000101667 0.000275214 0.715128836 UP NPIPA3

RP11_21L23.4 -1.295871015 -2.445158069 -3.97295704 0.00010174 0.000275324 0.699879133 DOWN RP11-21L23.4

CCL28 1.255960109 1.147264221 3.970346593 0.000102772 0.000277865 0.367494415 UP CCL28

SYT7 -1.375000743 4.583414094 -3.969889466 0.000102953 0.000278293 -0.00078813 DOWN SYT7

SNHG24 1.147209168 -5.452198526 3.969638154 0.000103053 0.000278469 0.70780206 UP SNHG24

HOXB7 1.000654759 -0.382388978 3.969362533 0.000103163

0.000278734	0.521126106UP	HOXB7

GPR1	1.197685068	-3.981075789	3.968751027	0.000103407

0.000279205	0.701255283	UP	GPR1

BDKRB1	1.110931942-1.709884519	3.968506966	0.000103505

0.000279436	0.617307729	UP	BDKRB1

RNU6_1189P	-1.317663523	-3.989436247	-3.968262904	0.000103603

0.000279668	0.704574362	DOWN	RNU6-1189P

RP11_159F24.2	-1.460811979	-3.740783447	-3.965955855	0.00010453

0.000282013	0.695441489	DOWN	RP11-159F24.2

MTNR1B	1.426254577	-5.469425974	3.965916034	0.000104546

0.000282024	0.693893864	UP	MTNR1B

ZNF229 1.210676305	-0.349935773	3.965858279	0.000104569

0.000282055	0.503699971	UP	ZNF229

OXCT1	1.008790108	1.06925975	3.964184775	0.000105247

0.000283756	0.358746534	UP	OXCT1

AFF2	1.520082451	-3.350761108	3.963581435	0.000105493

0.000284353	0.663934035	UP	AFF2

RP11_424G14.1	-1.398636062	-3.504258483	-3.962249849	0.000106036

0.000285786	0.680907604	DOWN	RP11-424G14.1

RP11_742B18.1	1.458592644	-4.117208273	3.962052685	0.000106117

0.000285971	0.67655106	UP	RP11-742B18.1

UMODL1	1.353909277	-4.029615647	3.959546521	0.000107148

0.000288522	0.667090062	UP	UMODL1

AQP10	1.654027428	-4.149042061	3.950088028	0.000111125 0.00029846

0.63185528	UP	AQP10

UTS2	1.284779698	-3.694364936	3.948679041	0.00011173

0.000300016	0.622443385	UP	UTS2

SULT1E1	-1.828089377	0.403877694	-3.944610388	0.000113493

0.000304408	0.410158821	DOWN	SULT1E1

MT1G	-2.422194424	3.586077198	-3.943887815	0.000113808

0.000305186	0.060365894	DOWN	MT1G

CTC_458G6.2	1.039923599	-5.773031016	3.942654123	0.00011435

0.0003065	0.609285759	UP	CTC-458G6.2

ICOS 1.104213242 -1.760275861 3.938828303 0.000116043 0.000310901 0.512359067 UP ICOS

ANKRD36BP2 1.233697777 -3.421658769 3.936053866 0.000117287 0.000314161 0.570960852 UP ANKRD36BP2

RP11_706C16.7 -1.575141955 0.554436142 -3.934795288 0.000117855 0.000315577 0.353860736 DOWN RP11-706C16.7

CHMP1B2P 1.306599321 -4.996814022 3.934479164 0.000117998 0.00031589 0.581331428 UP CHMP1B2P

SULT1C2P1 1.131428441 -4.216828528 3.933720841 0.000118342 0.000316669 0.577277481 UP SULT1C2P1

CT83 1.140574638 -5.801799741 3.9309471190.0001196080.000319914 0.566950047 UP CT83

U91319.1 -1.749577004 -1.472070511 -3.927322128 0.000121283 0.000324066 0.48903452 DOWN U91319.1

SMIM24 1.986103821 0.136148268 3.925608889 0.000122082 0.000325874 0.297555237 UP SMIM24

MAP9 1.070813186 -0.902131877 3.92506241 0.000122338 0.000326379 0.40241427 UP MAP9

ZDHHC11B -1.112351012 0.752523595 -3.9232511 0.00012319 0.000328538 0.286633725 DOWN ZDHHC11B

WI2_1896O14.1 -1.001434616 -1.027950046 -3.92315038 0.000123237 0.000328628 0.435399592 DOWN WI2-1896O14.1

LEF1 1.09514193 1.728361461 3.922807739 0.000123399 0.000328986 0.125027384 UP LEF1

LINC00925 1.175776649 -2.295341945 3.922726769 0.000123437 0.000329051 0.48229387 UP LINC00925

ERC2 1.230729466 -4.159807687 3.921036588 0.000124239 0.000331078 0.530800228 UP ERC2

C2orf40 -1.094646177 -2.744596688 -3.91756723 0.0001259 0.000335056 0.50910534 DOWN C2orf40

BMS1P8 2.013855928 -1.509476234 3.915154025 0.000127067 0.000337761 0.394876729 UP BMS1P8

BPIFB1 1.516963203 -3.843447377 3.910797082 0.000129202

0.000342725 0.486454716 UP BPIFB1

GAL 1.346335994 -4.272469942 3.910313748 0.000129441

0.000343265 0.492502135 UP GAL

RP11_268G12.1 1.371629162 -4.28673668 3.907868098 0.000130656

0.000346325 0.483770308 UP RP11-268G12.1

ARHGAP28 1.069167607 -0.630546841 3.904181847 0.000132508

0.000351001 0.306064343 UP ARHGAP28

PCDH9_AS2 -1.092618265 -5.785890916 -3.903852199 0.000132675

0.000351326 0.468948568 DOWN PCDH9-AS2

GPR88 -1.943532223 0.987752261 -3.903308207 0.00013295

0.000351978 0.209273641 DOWN GPR88

CNGB3 1.012493723 -4.745889782 3.901042907 0.000134104

0.000354759 0.461977103 UP CNGB3

SLC6A2 -2.44932046 -2.660589639 -3.900187804 0.000134542

0.000355799 0.43859253 DOWN SLC6A2

ACTBP8 1.324144604 -4.244031706 3.899681029 0.000134803

0.000356448 0.454447768 UP ACTBP8

CYP24A1 1.187983264 -5.179124861 3.894067854 0.000137718

0.000363433 0.436387382 UP CYP24A1

TFCP2L1 1.172939983 -1.083659429 3.892223775 0.000138688

0.000365793 0.297024234 UP TFCP2L1

CDH9 1.508561543 -5.229755375 3.891968227 0.000138823

0.000366109 0.428251053 UP CDH9

CNFN 1.069569492 -1.47629201 3.891826796 0.000138898

0.000366266 0.325348288 UP CNFN

PPEF1 1.00686791 -3.259927713 3.883470766 0.000143385

0.000377473 0.381628654 UP PPEF1

DCDC2 1.744265978 3.285581577 3.879382017 0.00014563 0.00038292

-0.221550796 UP DCDC2

LINC00239 1.267156632 -3.189803865 3.87624715 0.000147374

0.000387293 0.348429927 UP LINC00239

ASCL1 -1.860526488 -2.881065623 -3.873955045 0.000148662

0.000390505 0.353899132 DOWN ASCL1

RP11_476K15.1 1.601931196-3.507218647 3.869964158 0.00015093 0.000396071 0.330668864 UP RP11-476K15.1

AF277315.13 1.247103662 -5.647354541 3.860387669 0.000156506 0.00040958 0.315371195UP AF277315.13

PAEP 2.128768818 -3.616472612 3.852282041 0.000161378 0.0004211770.262249084 UP PAEP

RFX4 1.044967062 -5.135996213 3.852025615 0.000161534 0.000421447 0.287297665 UP RFX4

CTD_2054N24.21.034239503 -3.492684087 3.851399542 0.000161917 0.000422272 0.273356834 UP CTD-2054N24.2

TMC4 1.392349547 1.677393905 3.850151859 0.000162682 0.000424071 -0.133172294 UP TMC4

LINC01091 1.050232163 -1.999795374 3.847050139 0.000164598 0.00042874 0.19940619 UP LINC01091

SCGB2A1 1.30875618 -4.018482205 3.846842739 0.000164727 0.000428889 0.264174909 UP SCGB2A1

RP11_1070N10.5 1.011805458-5.521462438 3.846291303 0.00016507 0.000429643 0.266120567 UP RP11-1070N10.5

GPC3 1.759075795 6.883407262 3.8411241180.00016832 0.000437481 -0.527698985 UP GPC3

BCL2L14 1.110171256-1.252128042 3.8362165110.000171462 0.000444997 0.110843032UP BCL2L14

HCAR3 1.155142033 -3.87593781 3.835261398 0.00017208 0.000446429 0.222684328 UP HCAR3

ATP1A2 -1.276268288 -0.139132091 -3.833652267 0.000173126 0.000448753 0.050305132 DOWN ATP1A2

CD70 1.060634277 -2.814726611 3.833133508 0.000173464 0.000449582 0.186922657 UP CD70

ANKRD29 -1.060757981 2.124360757 -3.831101935 0.000174796 0.000452689 -0.202361388 DOWN ANKRD29

RP1_117P20.3 -1.150260365 -5.143477654 -3.826240797 0.000178021 0.000460295 0.196522259 DOWN RP1-117P20.3

LRRN2 1.036095674 0.638222172 3.825848621 0.000178284

0.000460875 -0.091412638 UP LRRN2

UNC5A 1.154580697 -1.871955264 3.823361398 0.000179958

0.000464801 0.106738893 UP UNC5A

CTD_2224J9.8 1.007096861 -3.977735586 3.820869252 0.000181651

0.000468718 0.174447855 UP CTD-2224J9.8

GTSF1 1.766264666 -2.168745878 3.819931544 0.000182292

0.000470219 0.099481895 UP GTSF1

MAPK8IP2 1.170549363 1.915449196 3.816236701 0.000184838

0.000476426 -0.282243468 UP MAPK8IP2

C11orf97 -1.214857008 -3.298103448 -3.815266665 0.000185512

0.000478066 0.155397254 DOWN C11orf97

DNAJC12 -1.107040742 3.029554825 -3.814572905 0.000185995

0.000479203 -0.38879421 DOWN DNAJC12

NETO1 -1.045527596 -4.776528905 -3.814248994 0.000186221

0.000479682 0.155391622 DOWN NETO1

RNASE2 1.066042292 -3.070844859 3.81238753 0.000187526

0.000482835 0.123814449 UP RNASE2

SULT1C4 1.163329832 -0.259667563 3.809508079 0.000189561

0.000487812 -0.065293982 UP SULT1C4

SLCO1B3 -2.490648961 1.464413453 -3.809006632 0.000189917

0.000488544 -0.16735997 DOWN SLCO1B3

AC016710.1 1.371332889 -5.376068926 3.808992727 0.000189927

0.000488544 0.134829774 UP AC016710.1

ADAMTS161.661105282-1.373453471 3.808407167 0.000190344

0.000489459 0.013994531 UP ADAMTS16

CNTN3 -1.952042276 -2.107770834 -3.807424924 0.000191046

0.000491053 0.096139222 DOWN CNTN3

UGT3A1 -2.483563982 3.042368092 -3.805920066 0.000192126

0.000493669 -0.363991862 DOWN UGT3A1

AC010969.1-1.236043471 -4.082850658 -3.803406101 0.000193942

0.000497856 0.117374807DOWN AC010969.1

AC005537.2 1.157415663 -5.11694653 3.802313404 0.000194737

0.000499628 0.112291042UP AC005537.2

PKHD1L1 1.338492137 -3.221081699 3.798272801 0.000197703
0.000506421 0.073146783 UP PKHD1L1
CTD_3128G10.7-1.338171382 5.589127946 -3.796623668 0.000198925
0.000509335 -0.679547037 DOWN CTD-3128G10.7
ANKRD26P1 1.231983507 -5.57803036 3.795247478 0.000199951
0.0005116880.086415301 UP ANKRD26P1
ERICH1_AS1 -1.398671677 -4.544325971 -3.795211823 0.000199978
0.0005117010.088701932 DOWN ERICH1-AS1
EHF 1.415048733 1.163207988 3.795150468 0.000200024
0.000511764-0.264732728 UP EHF
DPT -1.692599497 0.1301185 -3.794627909 0.000200415
0.000512709 -0.102388182 DOWN DPT
RP11_714M23.2 -1.820908374 -1.400254215 -3.789216967 0.000204505
0.000522225 -0.004291774 DOWN RP11-714M23.2
RP11_676J12.7 -1.485878968 -2.810826072 -3.788099252 0.00020536
0.000524102 0.05176157 DOWN RP11-676J12.7
DOK6 1.235166883 -0.901486221 3.784148175 0.000208409
0.000531456 -0.100442611 UP DOK6
C19orf81 1.288690193 -4.23334026 3.7831171460.000209212
0.000533048 0.04310202 UP C19orf81
TRIM50 1.92483871 -0.99011137 3.779819421 0.0002117990.000539295
-0.119922361 UP TRIM50
MCCD11.798261152-3.568114961 3.778944658 0.00021249 0.000540882
0.007473448 UP MCCD1
KCNH7 1.105447422 -4.3761221 3.7779234 0.0002133 0.000542828
0.026767984 UP KCNH7
LINC00942 1.792180837 -3.564301678 3.776864171 0.000214143
0.000544799 0.002083226 UP LINC00942
RP11_758N13.1 -1.489250723 -3.107296597 -3.776538119 0.000214403
0.000545345 0.017039407 DOWN RP11-758N13.1
CAPN9 1.171572519 -2.619962371 3.77613411 0.000214726 0.00054605
-0.021769121 UP CAPN9
CT45A10 1.015156654 -5.98056994 3.775914288 0.000214902

0.000546438 0.017858923 UP CT45A10

MMP13 1.110555988-5.401891986 3.77466725 0.000215901 0.000548864 0.015332651 UP MMP13

PPY2 1.001800994 -5.803312721 3.773378498 0.000216939 0.000551444 0.009674655 UP PPY2

LPAR3 1.390808482 -4.127937067 3.771737752 0.000218267 0.000554669 0.001798066 UP LPAR3

DUSP26 1.045660491 -3.022570033 3.771016344 0.000218854 0.000556074 -0.02259654 UP DUSP26

LINC01314 -1.280536418 2.966793722 -3.770184545 0.000219532 0.000557619 -0.53215821 DOWN LINC01314

SLC25A21 1.037845494 -3.053971523 3.766653501 0.000222432 0.000564447 -0.036008285 UP SLC25A21

AC034243.1 -1.186537037 -2.83217924 -3.765722166 0.000223203 0.000566237 -0.024797761 DOWN AC034243.1

ITGB6 1.323483339 -1.719732332 3.765715428 0.000223209 0.000566237 -0.106474228 UP ITGB6

HNF1B 1.067263745 3.714669093 3.764700266 0.000224052 0.000568015 -0.682261437 UP HNF1B

MMP17 1.264405631 -1.179909093 3.760782602 0.000227335 0.000575849 -0.161149142 UP MMP17

LINC01124 -1.223810524 1.480399066 -3.759419316 0.000228488 0.000578586 -0.373264669 DOWN LINC01124

CTD_2171N6.1 1.16121213 -3.665603972 3.758386487 0.000229365 0.000580561 -0.049673842 UP CTD-2171N6.1

GSDMC 1.11492991 -2.688105504 3.755994891 0.000231408 0.0005853 -0.087916906 UP GSDMC

BEND6 1.106612911-3.214299474 3.755213285 0.00023208 0.000586874 -0.071669025 UP BEND6

FIRRE 1.140545608 -3.365069071 3.754124502 0.000233018 0.000589185 -0.071813041 UP FIRRE

CASC9 2.096359436 -2.662087873 3.75406943 0.000233066 0.000589217 -0.116911813 UP CASC9

ZNF385D -1.624684207 -0.784826958 -3.753514053 0.000233546 0.000590332 -0.173825388 DOWN ZNF385D

SULF1 1.136537813 2.6751169893.750973497 0.000235755 0.000595349 -0.607405615 UP SULF1

MGC50722 -1.909474628 -2.02774719 -3.750189774 0.00023644 0.000596954 -0.106457719 DOWN MGC50722

RP11_427J23.1 -1.111437195 -2.83953022 -3.749278522 0.00023724 0.000598845 -0.081524344 DOWN RP11-427J23.1

AKR1C7P -1.011874669 -2.590753743 -3.747674708 0.000238652 0.000602157 -0.094133476 DOWN AKR1C7P

TRPV4 1.172274045 1.4812611063.746731757 0.000239487 0.000604008 -0.470177903 UP TRPV4

RP11_354K1.2 -1.087331025 -4.733712387 -3.746265175 0.000239901 0.000604988 -0.081211017 DOWN RP11-354K1.2

TACR1 -1.192583243 -2.783219867 -3.744872103 0.00024114 0.000607986 -0.098195812 DOWN TACR1

DNER 1.858373207 -3.517411958 3.744427023 0.000241538 0.000608786 -0.11320758 UP DNER

LINC00491 1.162597807 -5.351111349 3.736626485 0.000248603 0.00062555 -0.116290328 UP LINC00491

AREG 1.192020002 -1.031994728 3.7341729110.000250866 0.000630838 -0.262589882 UP AREG

FABP1 -1.878672343 8.350031165-3.733908168 0.0002511110.000631329 -0.900974862 DOWN FABP1

LINC00689 1.356238521 -4.458591227 3.7296631140.000255076 0.000640756 -0.140765072 UP LINC00689

RP11_874J12.4 1.159422435 -5.148760357 3.729241357 0.000255473 0.000641552 -0.141279896 UP RP11-874J12.4

HOXC11 1.005059093 -5.535776644 3.719558918 0.000264751 0.000663458 -0.175310255 UP HOXC11

KCND2 1.223328977 -3.402146788 3.718593496 0.000265693 0.000665668 -0.194269464 UP KCND2

RP11_347H15.5 1.24386847 -5.650531388 3.71547957 0.000268754

0.000672856 -0.189908959 UP RP11-347H15.5

LOXL4 1.363264217 3.261446524 3.713176369 0.00027104

0.000678224 -0.809664967 UP LOXL4

MT1M -1.751681313 0.727268672 -3.712478398 0.000271736

0.000679736 -0.441300302 DOWN MT1M

PZP-1.348945808 -0.141573167 -3.712456449 0.000271758

0.000679736 -0.371649779 DOWN PZP

RP11_946L20.2 1.620426417 -4.69866616 3.706770864 0.000277493

0.00069285 -0.219462085 UP RP11-946L20.2

SLC16A4 -1.01696443 0.775194013 -3.706166182 0.00027811

0.000694245 -0.483825663 DOWN SLC16A4

GAST 1.460766097 -5.273431665 3.704993221 0.00027931

0.000696949 -0.225215528 UP GAST

FAM87A 1.070167789 -4.247357103 3.704927554 0.000279377

0.000696972 -0.225605961 UP FAM87A

AC019181.2 -1.00773688 -2.603011801 -3.703115869 0.000281241

0.000701328 -0.247036951 DOWN AC019181.2

SHISA9 1.391584824 -4.14314586 3.701840891 0.000282559

0.000704028 -0.238838507 UP SHISA9

AGAP1_IT1 1.051482628 -1.811041881 3.698913732 0.000285608

0.000710958 -0.328232647 UP AGAP1-IT1

RP11_462L8.1 1.225744698 -0.819261497 3.693750412 0.000291062

0.00072363 -0.41969586 UP RP11-462L8.1

RP11_750B16.1 -1.090668625 0.946777584 -3.692516545 0.000292379

0.000726421 -0.548070306 DOWN RP11-750B16.1

PTPRS 1.268678508 2.331572811 3.687931924 0.000297325

0.000737629 -0.783021916 UP PTPRS

PCDHA4 1.027993878 -4.314023818 3.685607052 0.000299864

0.000743427 -0.291041 UP PCDHA4

IGHG4 1.74321999 1.900626506 3.685243317 0.000300263

0.000744261 -0.743147204 UP IGHG4

TDRD15 -1.331287082 -3.37105045 -3.683344354 0.000302353

0.000749134 -0.300076274 DOWN TDRD15

RHEBP2 -1.237874707 -1.346772658 -3.682573923 0.000303206 0.000750856 -0.38357693 DOWN RHEBP2

RP5_978I12.1 -1.329709739 -5.324706484 -3.680213339 0.000305831 0.000756184 -0.309580377 DOWN RP5-978I12.1

TMPRSS4 1.140091249 -3.24198634 3.676070785 0.00031049 0.000766436 -0.343687466 UP TMPRSS4

RP11_481F24.3 -1.744858846 -4.43747999 -3.669621977 0.000317877 0.000783699 -0.344013565 DOWN RP11-481F24.3

CRNDE 1.093779402 0.574594519 3.668342699 0.000319362 0.000787198 -0.635430777 UP CRNDE

DUOX2 2.060052913 -0.38642795 3.666729952 0.000321243 0.000791427 -0.561380324 UP DUOX2

ZDHHC19 -1.251594783 -0.853047763 -3.666579557 0.000321419 0.000791779 -0.473545022 DOWN ZDHHC19

AC004012.1 -1.056652683 -2.637564718 -3.665198602 0.000323039 0.00079536 -0.374941025 DOWN AC004012.1

RP11_278H7.5 -1.255508786 -4.496597393 -3.664652699 0.000323682 0.000796459 -0.360370694 DOWN RP11-278H7.5

CTD_2591A6.2 1.286519845 -4.870996805 3.664430946 0.000323943 0.000797012 -0.362541031 UP CTD-2591A6.2

SUN3 1.005713545 -5.074159603 3.662603549 0.000326105 0.000801236 -0.368409973 UP SUN3

AFAP1_AS1 1.832106591 -1.792500412 3.65853668 0.000330964 0.000812279 -0.479111175 UP AFAP1-AS1

HHLA2 1.109748785 -4.753906234 3.658256374 0.000331301 0.000813023 -0.383097715 UP HHLA2

HOGA1 -1.30921423 3.419698944 -3.653954396 0.00033652 0.000824731 -0.9942576 DOWN HOGA1

SLC5A1 1.715085375 -2.640681152 3.653444206 0.000337144 0.000826176 -0.453370771 UP SLC5A1

RP13_392I16.1 -1.214381868 -1.578344409 -3.652794515 0.000337941 0.000827958 -0.470067189 DOWN RP13-392I16.1

CXCL6 2.074456758 -0.52064231 3.650275011 0.000341045 0.000835308

-0.603972518 UP CXCL6

RP11_101E14.3 -1.578237013 -3.964240555 -3.650099875 0.000341262 0.000835668 -0.410194066 DOWN RP11-101E14.3

RP11_672A2.1 -1.170226256 -3.846288998 -3.647075828 0.000345027 0.000843849 -0.420386214 DOWN RP11-672A2.1

ZFP92 1.073043528 -0.665669989 3.646770037 0.00034541 0.000844699 -0.59060064 UP ZFP92

AC018804.7 1.072832523 -2.878605616 3.64286644 0.000350332 0.000856124 -0.467036835 UP AC018804.7

C11orf53 1.382672656 -4.970099093 3.64227903 0.000351078 0.000857685 -0.437658585 UP C11orf53

ZCCHC12 1.12403536 -3.967515632 3.641980048 0.000351459 0.000858527 -0.442219223 UP ZCCHC12

ABCC8 1.294540131 -3.117685052 3.641501846 0.000352068 0.00085984 -0.467235894 UP ABCC8

RP11_43F13.3 1.510169299 -2.548380466 3.638227213 0.000356268 0.000868766 -0.503002455 UP RP11-43F13.3

IGFALS -1.595031204 2.56637564 -3.637483083 0.000357229 0.000871021 -0.919451568 DOWN IGFALS

ANXA8L1 1.308033629 -3.98576447 3.633996876 0.000361763 0.000881538 -0.470226802 UP ANXA8L1

CEACAMP10 -1.079842802 -5.139652894 -3.63177041 0.000364688 0.00088803 -0.472369448 DOWN CEACAMP10

GABRB3 2.00429575 -1.863398162 3.631287864 0.000365324 0.000889309 -0.569968929 UP GABRB3

CLIC6 1.509054137 -1.461561765 3.624409887 0.000374514 0.000910476 -0.610577431 UP CLIC6

PRSS3 1.960012168 -0.577539234 3.623879903 0.000375232 0.000912034 -0.688852011 UP PRSS3

B4GALNT3 1.167736575 -0.179103845 3.623395465 0.000375888 0.000913259 -0.714497225 UP B4GALNT3

GMPR -1.006033339 1.629534399 -3.616502927 0.000385349 0.000935021 -0.884975029 DOWN GMPR

UGT1A6 -1.388891044 3.574458815 -3.611975432 0.000391684 0.000949226 -1.153204925 DOWN UGT1A6

RTL1 1.212196993 -5.308162144 3.610625143 0.000393593 0.000953755 -0.544205293 UP RTL1

LINC01101 1.031233988 -4.681226148 3.610044148 0.000394417 0.000955461 -0.545218264 UP LINC01101

FAM198A -1.23343341 -0.115887043 -3.607280975 0.000398357 0.000964225 -0.731922857 DOWN FAM198A

LMOD2 -1.011483324 -4.82321692 -3.607031696 0.000398715 0.000964993 -0.554465187 DOWN LMOD2

PROK1 -1.247415173 -4.021212934 -3.60547013 0.00040096 0.00097023 -0.559775282 DOWN PROK1

TC2N 1.199618391 2.041753099 3.603381525 0.000403982 0.000976455 -1.031236115 UP TC2N

MYOM1 -1.137067819 4.329255806 -3.599572676 0.000409547 0.000989308 -1.289735455 DOWN MYOM1

14-Sep 1.130867147 -5.547520959 3.595773042 0.000415171 0.001001781 -0.594328146 UP 14-Sep

MYO18B 1.603056175 -2.199378682 3.594281388 0.000417399 0.00100675 -0.670604013 UP MYO18B

WNK4 1.336027341 0.474689014 3.590853086 0.000422562 0.001018791 -0.892808561 UP WNK4

MIR646HG 1.164900632 -3.215848247 3.590675668 0.00042283 0.001019236 -0.632091715 UP MIR646HG

RP11_497G19.1 1.187433518 -4.087184555 3.584441323 0.000432379 0.001040153 -0.63399527 UP RP11-497G19.1

NPNT 1.111763866 2.792233858 3.584229109 0.000432707 0.001040839 -1.193393798 UP NPNT

LINC01474 -1.048227749 -1.805727419 -3.584052296 0.000432981 0.001041289 -0.687744586 DOWN LINC01474

PAGE2B 1.469194871 -4.700965283 3.582878656 0.000434804 0.001045236 -0.636867775 UP PAGE2B

RP5_968D22.1 -1.210613696 -2.333511604 -3.582196312 0.000435867

0.001047385	-0.664719914	DOWN RP5-968D22.1
ICAM5 1.107278896	-3.202651056	3.581660325	0.000436703
0.00104929 -0.661319167	UP ICAM5
RNF1861.131827067	-3.685541105	3.578474239	0.000441708
0.001059946	-0.659590517	UP RNF186
MAMDC4	-1.098224991	3.532009981	-3.57562849 0.000446223
0.001070014	-1.280844811	DOWN MAMDC4
RNF1831.092896219	-3.689811151	3.571827056	0.000452323
0.00108312 -0.681195756	UP RNF183
ENTPD3	1.246177472	-3.753648238	3.570633216	0.000454254
0.00108731 -0.685408682	UP ENTPD3
ACSL4 1.300483871	6.584702226	3.570323884	0.000454756
0.001088185	-1.469384277	UP ACSL4
GRIN2B	-1.48617603 -2.35216446 -3.569171199	0.000456631
0.001092561	-0.706769986	DOWN GRIN2B
GUCY2EP	-1.194946827	-3.499787864	-3.567637315	0.000459136
0.001098116-0.687473893	DOWN GUCY2EP
UST	-1.096102258	0.295261281	-3.565367598	0.000462868
0.001106155-0.912456931	DOWN UST
RP11_728F11.4 1.033393294	-2.996400645	3.565134101	0.000463253
0.001106966-0.721455814	UP RP11-728F11.4
SMKR1 1.086210495	-3.230070701	3.561732451	0.000468903
0.001119013-0.726052069	UP SMKR1
BPI -1.00214093 -2.393064475	-3.561010102	0.000470111 0.001121337-
0.733349146	DOWN BPI
RP11_436I9.6	-1.080788844	-2.200877237	-3.55896499 0.000473547
0.001128746-0.748865773	DOWN RP11-436I9.6
GDNF	-1.659901736	-1.133340896	-3.555356676	0.000479668
0.001141514-0.819750985	DOWN GDNF
EVX1	1.316950394	-4.061566954	3.553022664	0.000483666
0.001150457-0.739398739	UP EVX1
BAALC -1.021493655	0.863324305	-3.551067362	0.00048704
0.001157677-1.017160389	DOWN BAALC

CTD_2015G9.2 1.255600493 -3.767431874 3.546576108 0.000494874 0.001175012-0.764888549 UP CTD-2015G9.2

LIPH 1.325262769 -1.021253239 3.544605638 0.000498348 0.001182439-0.906450772 UP LIPH

FAM183A 1.322799066 -4.275898695 3.543315477 0.000500635 0.001187639-0.769140493 UP FAM183A

C6orf222 1.137347897 -4.624973115 3.53937146 0.000507688 0.001202453 -0.779939769 UP C6orf222

U3 -1.001518327 -5.207765603 -3.535343165 0.000514988 0.001218657 -0.793041184 DOWN U3

TMEM163 1.450688886 -0.960367693 3.534328368 0.000516842 0.001222803 -0.947040061 UP TMEM163

PCAT141.140383025 -4.664171326 3.533323171 0.000518685 0.001226921 -0.799763002 UP PCAT14

ANKRD18DP -1.03792008-4.237171354 -3.533261133 0.000518799 0.001227069 -0.798635611 DOWN ANKRD18DP

SCD5 1.094179254 0.723049442 3.5292117220.000526289 0.001243657 -1.116955354 UP SCD5

AF124730.4 -1.066126119 -3.112291071 -3.528795831 0.000527063 0.001245261 -0.819231631 DOWN AF124730.4

ABO -1.020850245 -1.723477329 -3.52743637 0.000529604 0.001251016 -0.880217154 DOWN ABO

LRRN1 1.421128056-3.379792828 3.522917764 0.000538131 0.001269403 -0.85524849 UP LRRN1

RP11_408E5.4 1.1362208 -5.712235616 3.516280533 0.00055089 0.001296432 -0.85736034 UP RP11-408E5.4

PERM1 1.007255188 -1.722898079 3.5119114250.000559443 0.001315396 -0.95879312 UP PERM1

FERMT1 1.391949305 0.310268267 3.508792863 0.000565625 0.001329276 -1.146732333 UP FERMT1

AC141928.1 1.26034496 -2.550811858 3.508348006 0.000566512 0.001331099 -0.931115725 UP AC141928.1

MAGEA8 1.31456574 -4.812091747 3.505079775 0.000573068

0.001345315 -0.892456757 UP MAGEA8
PRAMEF8 -1.06190486 -5.024285079 -3.504804065 0.000573624
0.001346225 -0.892484006 DOWN PRAMEF8
LINC01146 -1.462602844 2.125723125 -3.502232261 0.000578838
0.001357795 -1.317108018 DOWN LINC01146
GSTM1 -2.596884939 1.337880559 -3.4971895 0.000589191
0.001379454 -1.206267698 DOWN GSTM1
INSM1 1.065960497 -5.398804401 3.497173187 0.000589225
0.001379454 -0.918650874 UP INSM1
AIM2 1.008671345 -2.140401378 3.496742847 0.000590117
0.001381406 -0.98364935 UP AIM2
RP11_357H14.17 1.000723818 -5.099756054 3.490663774
0.000602847 0.001408449 -0.939027073 UP RP11-357H14.17
MAGEA6 2.086418817 -3.80730133 3.488087646 0.000608319
0.001420124 -0.967605049 UP MAGEA6
KCNK17 -1.157657992 -1.846257821 -3.483541982 0.000618089
0.00144026 -1.014990915 DOWN KCNK17
DKK4 -1.819482941 -2.49251882 -3.482238571 0.000620917
0.001446568 -0.987877386 DOWN DKK4
CCR6 1.002118053 -4.218410912 3.479955424 0.0006259 0.00145676 -
0.974950967 UP CCR6
VIPR2 1.050691348 -3.982018033 3.479380926 0.00062716
0.001459124 -0.979132865 UP VIPR2
CRPP1 -1.0224937 -4.995476925 -3.479113391 0.000627748
0.001460207 -0.975855379 DOWN CRPP1
BPIFB2 2.008928593 -1.489529794 3.478783818 0.000628472
0.001461608 -1.102574124 UP BPIFB2
UGT1A10 1.639653676 -3.90856603 3.478103024 0.000629971
0.001464524 -0.990098869 UP UGT1A10
RP11_235G24.1 1.004039536 -4.811732059 3.478047409 0.000630094
0.001464667 -0.979678999 UP RP11-235G24.1
RP11_54O7.18 1.022264884 -3.546783707 3.477386424 0.000631553
0.001467773 -0.992763174 UP RP11-54O7.18

TBX1 1.057083862 -2.757202466 3.471977416 0.000643611 0.001494634 -1.036684068 UP TBX1
NOL4 -1.305513045 -4.045943898 -3.470626791 0.000646655 0.001500684 -1.003036289 DOWN NOL4
CAMK2B -1.51997576 0.070846541 -3.46991462 0.000648266 0.001504276 -1.197908637 DOWN CAMK2B
RP1_167O22.1 -1.186718173 -4.825784784 -3.469389603 0.000649455 0.001506744 -1.007126083 DOWN RP1-167O22.1
MYO1A 1.218159072 -0.086205305 3.468992192 0.000650357 0.001508544 -1.236764377 UP MYO1A
RP11_150O12.3 1.457139611 -2.574268659 3.467367885 0.000654056 0.001516499 -1.068791113 UP RP11-150O12.3
NOVA1 1.051431286 -0.170464666 3.467346972 0.000654104 0.001516499 -1.229488114 UP NOVA1
NPHS1 1.107170944 -4.524331712 3.467157607 0.000654537 0.001517208 -1.015425389 UP NPHS1
GSPT2 1.104391949 1.645110958 3.464068154 0.000661631 0.001533207 -1.442043982 UP GSPT2
RASEF 1.434723015 1.37403247 3.461947126 0.000666544 0.001543693 -1.418193263 UP RASEF
RP11_242J7.1 1.691257171 -1.521171144 3.458728473 0.000674063 0.001558694 -1.156833438 UP RP11-242J7.1
MT1DP -1.194848179 -1.658936388 -3.455623125 0.000681394 0.001574427 -1.115902674 DOWN MT1DP
LINC00540 1.124793268 -4.581140194 3.452084508 0.000689838 0.001592247 -1.063925841 UP LINC00540
KLB -1.040144659 5.230128681 -3.449331585 0.000696475 0.001605087 -1.84794029 DOWN KLB
PLD5 1.213613547 -3.423709897 3.446639731 0.000703023 0.001618462 -1.09810788 UP PLD5
RP11_63P12.7 -1.051706137 -3.785178919 -3.445630668 0.000705492 0.00162399 -1.084074313 DOWN RP11-63P12.7
DSC3 1.23972758 -3.99799027 3.445312591 0.000706272 0.001625598

-1.09016281 UP DSC3
PEG3 1.551719085 0.924855338 3.442117471 0.000714153
0.001642977 -1.436763239 UP PEG3
UTY -2.125506863 -0.184894206 -3.440908738 0.000717155
0.001648616 -1.258262996 DOWN UTY
TMEM178B 1.220845635 -0.607646358 3.439419541 0.000720871
0.00165668 -1.283217534 UP TMEM178B
FGF2 -1.043973591 0.93839546 -3.429671113 0.000745643 0.00170934
-1.421592264 DOWN FGF2
DPP10 1.541628281 -4.325715845 3.428349633 0.000749062
0.001716519 -1.143109787 UP DPP10
EREG 1.413703869 -3.314061132 3.427182115 0.000752094
0.001722808 -1.166953929 UP EREG
ARHGEF4 1.268353492 -1.751422473 3.422854739 0.000763435
0.001746278 -1.250278236 UP ARHGEF4
GABRG3 1.18003394 -4.092247779 3.419623348 0.000772008
0.001764706 -1.170869569 UP GABRG3
PCP4L1 -1.156386313 -0.51143818 -3.41352836 0.000788424 0.001799135
-1.336526707 DOWN PCP4L1
RP11_403A3.3 -1.493574728 -3.627740557 -3.411416315 0.000794188
0.001811424 -1.194499505 DOWN RP11-403A3.3
ELOVL2_AS1 -1.006864939 -1.702496142 -3.41130513 0.000794492
0.001811946 -1.258146297 DOWN ELOVL2-AS1
HERC2P3 1.165296797 -0.92764921 3.407511398 0.000804949
0.001834569 -1.357290124 UP HERC2P3
CTC_480C2.1 1.099054589 -5.226635014 3.407163717 0.000805914
0.001836068 -1.207883277 UP CTC-480C2.1
THBS4 -1.279090375 2.601829499 -3.402722014 0.000818334
0.001863299 -1.712235033 DOWN THBS4
MMP23A -1.166711394 -4.143038189 -3.394983852 0.000840401
0.001909726 -1.245015927 DOWN MMP23A
AC099684.1 -1.053253313 -1.780792437 -3.388378052 0.000859677
0.001950379 -1.32551484 DOWN AC099684.1

GP2 1.930054217 -3.01115728 3.387266256 0.000862962 0.001956862 -1.316858111 UP GP2

PALM3 -1.08047143 3.291820811-3.3820511 0.000878527 0.001988428 -1.883038443 DOWN PALM3

RP11_492D6.3 1.110398233-3.915632496 3.376319566 0.000895935 0.002024191 -1.3099327 UP RP11-492D6.3

HOXD10 1.236140126 -3.613664259 3.372830633 0.000906689 0.002045396 -1.32850098 UP HOXD10

KRT23 2.081959319 1.497393727 3.37271164 0.000907058 0.002046036 -1.732646016 UP KRT23

TINAG 1.819472592 -3.173840563 3.371074517 0.000912148 0.002056934 -1.35976205 UP TINAG

LINC01194 1.36005743 -4.816623356 3.367808828 0.000922381 0.002078245 -1.332483253 UP LINC01194

CDH3 1.004709109 -2.351039819 3.362329603 0.00093979 0.002114682-1.403432347 UP CDH3

CYP2C19 -1.145764518 -2.169986342 -3.359152308 0.000950026 0.002136508 -1.394509693 DOWN CYP2C19

LAD1 1.274145514 4.895625835 3.358321882 0.000952718 0.002141757 -2.127563729 UP LAD1

SLC1A7 1.437292562 0.735473845 3.354973328 0.000963646 0.002165308 -1.689595523 UP SLC1A7

OR2I1P 1.192124474 4.269687545 3.354632344 0.000964766 0.002167416 -2.100079302 UP OR2I1P

SHISA2 1.019400165 -2.46283933 3.352410528 0.00097209 0.002181856 -1.428878698 UP SHISA2

ELN -1.029664588 3.759019033 -3.351849015 0.000973949 0.002185582 -2.041173924 DOWN ELN

SPEG 1.064514561 -0.645267471 3.351697129 0.000974452 0.002186301 -1.556453591 UP SPEG

C1QTNF3 -1.186924077 2.064914417 -3.346099522 0.000993177 0.002225806 -1.821132028 DOWN C1QTNF3

SLC6A11 2.053957019 -0.557516113 3.346034896 0.000993395

0.002226087 -1.600351799 UP SLC6A11

RP11_57A1.1 1.117598091-3.976001253 3.34554741 0.000995042 0.00222915 -1.405967371 UP RP11-57A1.1

FAM201A 1.511034964-2.013556081 3.345336868 0.000995754 0.002230536 -1.484106404 UP FAM201A

B3GALT2 1.079950026 -1.90160588 3.344808684 0.000997542 0.002234124 -1.485070943 UP B3GALT2

STRA8 1.011808122-4.33770929 3.342581393 0.0010051160.002249824 -1.411798722UP STRA8

PRDM16 1.105786171 -0.446631354 3.341976275 0.001007183 0.002253607 -1.605737618 UP PRDM16

CDHR2 1.64871423 1.960685694 3.3417986110.001007791 0.002254756 -1.882712698 UP CDHR2

ANKRD33 1.327426132 -4.192988408 3.332637213 0.001039593 0.002319398 -1.445434279 UP ANKRD33

LINC00189 1.127803358 -3.110660628 3.328302989 0.001054962 0.002351275 -1.48131865 UP LINC00189

RBBP8NL 1.270238928 -4.753237768 3.32673705 0.001060567 0.002362886 -1.460922123 UP RBBP8NL

VCAN 1.1193468513.656266826 3.325321499 0.001065657 0.002373564 -2.136930366 UP VCAN

FGF7 -1.270515983 -1.45953729 -3.322315184 0.001076544 0.002396027 -1.551125202 DOWN FGF7

CYP3A7 -1.424271719 4.656396029 -3.320572118 0.001082903 0.002408836 -2.217959813 DOWN CYP3A7

ALDH3A1 -2.361866665 2.301958444 -3.320138632 0.00108449 0.002412142 -1.897012521 DOWN ALDH3A1

RP11_180N14.1 -1.274627509 -3.553409458 -3.318273358 0.001091344 0.002426257 -1.487649195 DOWN RP11-180N14.1

PAGE2 1.497902693 -5.009131771 3.318092817 0.001092009 0.002427511-1.488345708 UP PAGE2

NTM 1.262444145 -0.838637133 3.314765659 0.001104342 0.002451735 -1.658800297 UP NTM

GGT1 1.090978299 4.541093935 3.314288329 0.0011061210.00245523 -2.248302729 UP GGT1

PWAR5 1.04961204 -3.189349158 3.310994601 0.0011184760.002478513 -1.530742597 UP PWAR5

CMA1 -1.111433063 -3.92783076-3.305848351 0.0011380370.00251673 -1.524681441 DOWN CMA1

RP11_25K19.1 1.180666499 -1.700453404 3.305485564 0.001139428 0.002519573 -1.621479427 UP RP11-25K19.1

AC096559.1-1.010491567 -4.150409696 -3.305392778 0.001139784 0.002519894 -1.525691133 DOWN AC096559.1

FCRLA 1.13582689 -2.219894021 3.305196639 0.0011405370.002521092 -1.591669639 UP FCRLA

AC003973.3 1.075380291 -2.354249115 3.30206719 0.001152612 0.002545431 -1.592256489 UP AC003973.3

RND2 1.255226686 0.468643715 3.299642923 0.001162048 0.002563902 -1.831007579 UP RND2

RP11_1018N14.5 -1.000616822 -2.815767039 -3.298996419 0.0011645760.002569007 -1.557402081 DOWN RP11-1018N14.5

TMEM52B 1.059999056 -3.473855008 3.297296837 0.001171248 0.002582771 -1.56511544 UP TMEM52B

PCDHA6 1.045159391 -4.624895153 3.294213635 0.001183442 0.002607257 -1.561375169 UP PCDHA6

ZFY -1.927469806 -0.565665744 -3.29264331 0.0011896980.00261935 -1.699066772 DOWN ZFY

CHGA 1.407636577 -3.539411491 3.286639519 0.001213901 0.002668708 -1.602473179 UP CHGA

SYPL2 -1.132529523 1.78563599 -3.285843914 0.001217143 0.002675342 -1.975131116 DOWN SYPL2

RP1_40E16.9 1.069121949 -5.27011628 3.284129334 0.001224156 0.002689769 -1.593035188 UP RP1-40E16.9

CFTR 1.856600604 -1.423567835 3.281463459 0.001235135 0.002711899-1.725219183 UP CFTR

FAM83F 1.337929026 -2.046389842 3.2761175340.001257428

0.002757302 -1.694074026 UP FAM83F
MKX -1.268063541 -2.15835876 -3.272039553 0.001274684
0.002790538 -1.664913785 DOWN MKX
FAM19A4 1.313379169 -4.579498585 3.271513266 0.001276927
0.002794681 -1.632070429 UP FAM19A4
EIF1AY -2.29599742 0.574306226 -3.265018127 0.001304914
0.002853086 -1.876061195 DOWN EIF1AY
DLX3 1.058271882 -4.656089755 3.264119301 0.001308832
0.002860842 -1.653908458 UP DLX3
FIGF -1.10552278 -0.179908363 -3.2631514 0.001313063 0.002869565
-1.837670183 DOWN FIGF
BPIFB4 1.128197703 -4.214364269 3.259739297 0.00132808 0.00289841
-1.669246899 UP BPIFB4
C5orf46 1.036067955 -2.13145737 3.254211211 0.001352748 0.002947138
-1.75059869 UP C5orf46
TXLNGY -2.211619613 0.211570411 -3.253725199 0.001354937
0.002951369 -1.879033636 DOWN TXLNGY
RSPO4 1.008371048 -3.652186528 3.253504491 0.001355932
0.002952461 -1.694913422 UP RSPO4
DLX1 1.126582657 -3.874142466 3.249849196 0.001372513
0.002986119 -1.703379384 UP DLX1
SMPX -1.440851149 -2.416802375 -3.249717177 0.001373116
0.002986886 -1.721606716 DOWN SMPX
FUT3 1.129487552 -1.830785084 3.244688045 0.001396246
0.003032788 -1.800553212 UP FUT3
ADCYAP1 -1.050444518 -3.267897441 -3.244609519 0.00139661
0.003033303 -1.716388145 DOWN ADCYAP1
RP11_332J15.2 1.093082731 -5.306471276 3.24175766 0.001409891
0.003060201 -1.722877183 UP RP11-332J15.2
HAMP -1.901135968 2.422524644 -3.241224534 0.001412386
0.003064505 -2.171082414 DOWN HAMP
C6orf141 1.337339759 -1.306562558 3.240427865 0.001416123
0.003072335 -1.852156459 UP C6orf141

RP11_466P24.6 -1.101406222 -3.657203662 -3.235896546 0.001437554 0.003114076-1.740155243 DOWN RP11-466P24.6

RP11_44F21.5 1.136651736 -4.89349914 3.23478211 0.00144287 0.003123605 -1.743467489 UP RP11-44F21.5

RP11_91H12.1 -1.071423285 -4.823827325 -3.234363566 0.001444872 0.003126764 -1.743774407 DOWN RP11-91H12.1

SSUH2 1.150923109 0.26676753 3.212395834 0.001553613 0.003346944 -2.077799088 UP SSUH2

MAGEA3 1.959295369 -3.382817175 3.2118772350.00155627 0.003352063 -1.844786049 UP MAGEA3

SDK2 1.202002411-0.970771932 3.208506348 0.001573641 0.003387038 -1.974354414 UP SDK2

RP11_203I2.1 -1.061857174 -2.215065558 -3.20720616 0.001580389 0.003401256 -1.861066754 DOWN RP11-203I2.1

RP11_1036E20.7 -1.391321554 -3.51978563 -3.207151535 0.001580673 0.003401562 -1.828171424 DOWN RP11-1036E20.7

DUOXA2 1.771908912 -2.318523522 3.203793667 0.001598228 0.003436557 -1.90878698 UP DUOXA2

ANKRD18A 1.311899125-2.693533743 3.2004872 0.001615691 0.003470985 -1.892556229 UP ANKRD18A

NLGN1 1.090927249 -3.995861233 3.198193109 0.001627911 0.003494413 -1.857635957 UP NLGN1

CTSLP1 -1.049698044 -4.160411253 -3.195816375 0.001640662 0.003519571 -1.859990962 DOWN CTSLP1

ZFP57 1.064265535 -4.13123675 3.192076001 0.001660916 0.003558233 -1.874566525 UP ZFP57

MAGEA12 1.735305015 -4.012915555 3.188936526 0.001678094 0.003591498 -1.891496758 UP MAGEA12

RP11_500B12.1 -1.075111165 -4.665527208 -3.18065619 0.001724197 0.003678646 -1.905474579 DOWN RP11-500B12.1

C1QL1 1.306280884 -0.197190218 3.180369143 0.001725816 0.003681772 -2.126837435 UP C1QL1

CKB 1.033278356 4.433062668 3.179719878 0.001729484

0.003688834 -2.655714807 UP CKB

SCN7A -1.178659249 -2.33054929 -3.175331509 0.001754461

0.003736882 -1.950480215 DOWN SCN7A

C5orf58 1.043772122 -3.039552354 3.173277279 0.001766268

0.003760355 -1.954723436 UP C5orf58

KDM5D -2.183075982 0.890808215 -3.165505877 0.001811603

0.003848652 -2.211786857 DOWN KDM5D

SLC22A31 1.860611608 -1.340451333 3.165455312 0.001811902

0.003848944 -2.086335484 UP SLC22A31

PNMA6B -1.192028972 -4.813485153 -3.162936873 0.001826826

0.003878579 -1.958688213 DOWN PNMA6B

NKD1 -1.481979431 1.871582262 -3.162280224 0.001830736

0.003885845 -2.354851149 DOWN NKD1

RP4_568C11.4 -1.491982526 -0.360776932 -3.160218064 0.001843064

0.003909239 -2.126723807 DOWN RP4-568C11.4

RP11_392E22.9 1.002393763 -4.233830798 3.16003079 0.001844188

0.003911275 -1.969340178 UP RP11-392E22.9

MGAM 1.217391776 -2.067235375 3.158582291 0.001852899

0.003928356 -2.046229204 UP MGAM

LRRTM4 -1.397531728 -3.665655788 -3.155803406 0.001869717

0.003960853 -1.981022932 DOWN LRRTM4

TDRD9 1.04799543 -2.837321427 3.149206971 0.001910208

0.004037596 -2.034236054 UP TDRD9

MAGEA11 1.109324237 -5.365087558 3.148214151 0.001916372

0.004049644 -2.004094422 UP MAGEA11

SLC45A2 -1.128137368 -1.547091901 -3.146237245 0.001928701

0.004073537 -2.083864531 DOWN SLC45A2

RP11_407A16.3 1.083619999 -5.21377687 3.14510598 0.001935789

0.004088146 -2.01289315 UP RP11-407A16.3

VCX3A 1.078691934 -5.267596166 3.143964372 0.001942966

0.004101493 -2.016393332 UP VCX3A

CNTNAP3B -1.083780406 -0.604549916 -3.143782429 0.001944112

0.00410355 -2.163622884 DOWN CNTNAP3B

RP11_256L6.3 -1.111022379 -2.857472615 -3.143078144 0.001948554 0.004112564-2.028376404 DOWN RP11-256L6.3

LINC01021 -1.253723099 -0.899083674 -3.142757077 0.001950583 0.004116482-2.13884765 DOWN LINC01021

CTD_2118P12.1 1.049455453 -5.038994867 3.130793609 0.00202757 0.004263904 -2.054808018 UP CTD-2118P12.1

CTD_2147F2.1 1.095929375 -5.119458581 3.129830639 0.002033887 0.004276814 -2.057868268 UP CTD-2147F2.1

FAM9B -1.2601073 -2.279644054 -3.128415953 0.002043201 0.004293757 -2.091728134 DOWN FAM9B

SAMD12 1.028754618 -0.154904639 3.1261311060.002058328 0.004321747 -2.289160296 UP SAMD12

PRKAA2 1.03953235 2.448714015 3.125385036 0.002063289 0.004330641 -2.600662354 UP PRKAA2

TLX1 -1.141058002 0.004206904 -3.12323225 0.002077668 0.004358146 -2.275124748 DOWN TLX1

CCBE1 -1.125866997 -1.59274325 -3.11572407 0.002128544 0.004453928 -2.170491336 DOWN CCBE1

FAM3B 1.697314023 0.818680944 3.1139641290.002140635 0.00447727 -2.441515794 UP FAM3B

KRT6B 1.212879217 -4.24280068 3.103727875 0.002212228 0.004614097 -2.136470279 UP KRT6B

GFPT2 1.038257043 -0.154115502 3.102701388 0.002219528 0.004628112-2.358656858 UP GFPT2

TMSB4Y -1.263627693 -2.435770852 -3.102390331 0.002221744 0.004631522 -2.161518507 DOWN TMSB4Y

BARX1 1.173026763 -3.972472606 3.097034061 0.002260233 0.004705606 -2.158731791 UP BARX1

MTRNR2L1-1.969364225 -0.658552564 -3.09361832 0.002285099 0.00475241 -2.291928055 DOWN MTRNR2L1

KRT87P-1.187361465 -3.079363675 -3.090392459 0.002308814 0.004794227 -2.178946953 DOWN KRT87P

CA12 1.359550645 1.877909924 3.090204306 0.002310204

0.004796697 -2.634515147 UP CA12
KCNS1 1.032756435 -4.111574611 3.089963209 0.002311987
0.004799982 -2.176731798 UP KCNS1
SPINK1 1.913903717 4.623945064 3.088090594 0.002325875
0.004825046 -2.930102369 UP SPINK1
LINC01320 -1.463516967 -1.427788034 -3.086790011 0.002335566
0.00484305 -2.261509165 DOWN LINC01320
FGF14 -1.046073613 0.063703829 -3.083733463 0.002358489
0.004888463 -2.397950072 DOWN FGF14
TENM4 1.002306012 -2.267030832 3.081820458 0.00237294
0.004916713 -2.257150497 UP TENM4
GLB1L3 1.058338814 -4.233980813 3.079585483 0.002389928
0.004947781 -2.206128972 UP GLB1L3
LINC01152 1.172630668 -2.833662936 3.078660265 0.002396993
0.004960534 -2.244192595 UP LINC01152
CHRND 1.084667906 -4.639371184 3.073345806 0.002437951
0.005035552 -2.22290063 UP CHRND
PRG4 -1.371214935 5.180040614 -3.071126343 0.002455246
0.005068806 -3.008539492 DOWN PRG4
AC068138.1 -1.043569368 -5.636112103 -3.070762705 0.002458091
0.005073803 -2.232478139 DOWN AC068138.1
TUBB4A 1.36959918 -0.206608423 3.061381572 0.002532531
0.005216202 -2.480158264 UP TUBB4A
ASNSP1 1.476762307 -3.820441021 3.05682092 0.002569466
0.005283631 -2.281692483 UP ASNSP1
RPS4Y1 -2.508635466 3.347290517 -3.054241044 0.002590578
0.005323882 -2.825214055 DOWN RPS4Y1
SLC30A3 1.094272432 -2.127755807 3.050335676 0.002622843
0.005385058 -2.359137033 UP SLC30A3
ATRNL1 1.257875518 -1.204897067 3.047256302 0.002648544
0.005433628 -2.433043926 UP ATRNL1
VCX 1.12044414 -4.753075885 3.046916004 0.002651399
0.005439017 -2.299263246 UP VCX

NAP1L6 1.01910072 -4.912460319 3.04156081 0.002696693 0.005524351 -2.314491849 UP NAP1L6

C19orf80 -1.204079105 5.027622606 -3.038907761 0.002719395 0.005567044 -3.099313216 DOWN C19orf80

KRT6C 1.206730375 -4.805762259 3.034671935 0.002756007 0.005635241 -2.334626724 UP KRT6C

TEX41 1.152619998 -1.051867589 3.034047968 0.002761438 0.005644899 -2.481521025 UP TEX41

CLDN10 1.63060247 -1.985345093 3.029951672 0.00279734 0.005711942-2.433791924 UP CLDN10

COMP 1.433876676 -1.327678433 3.022729298 0.002861689 0.005835864 -2.495961387 UP COMP

NEBL 1.083788534 0.919681609 3.022059485 0.002867725 0.005845682 -2.705202746 UP NEBL

LINC01426 1.023884031 -0.091646425 3.016245969 0.002920611 0.005944872 -2.616620531 UP LINC01426

CCNO 1.254214641 -1.115116694 3.012269244 0.002957302 0.006015973 -2.540062949 UP CCNO

AC068535.3 -1.502020553 -2.10159363 -3.008572795 0.002991788 0.006080954 -2.446869173 DOWN AC068535.3

XKR9 -1.098335575 -0.622423569 -3.00586322 0.0030173 0.006128643 -2.56241058 DOWN XKR9

OSR2 1.135455105 -1.466775775 2.983622806 0.003234397 0.006529676 -2.59273177 UP OSR2

KRTCAP3 1.069487932 0.74127477 2.981077137 0.003260141 0.006577207 -2.805029342 UP KRTCAP3

TKTL1 1.166708763 -3.274304346 2.977216207 0.003299545 0.006651939 -2.519636668 UP TKTL1

RP11_168L7.1 -1.191392735 -2.533678736 -2.975585703 0.003316317 0.006684298 -2.522308091 DOWN RP11-168L7.1

CST2 1.11104668 -3.113799692 2.97527202 0.003319552 0.006689167 -2.52905062 UP CST2

NOVA1_AS1 1.144432362 -4.668575563 2.974632024 0.003326162

0.006700743	-2.505262883	UP	NOVA1-AS1

AC011294.3	1.109376198	-2.24083831	2.974198639	0.003330646	0.006708131	-2.570929606	UP	AC011294.3

RP11_191L9.4	1.005313929	-4.869681212	2.971184364	0.003361981	0.006768392	-2.514616123	UP	RP11-191L9.4

MZB1	1.092071964	0.014480029	2.95374117	0.00354869	0.007110164	-2.806278047	UP	MZB1

DDX3Y	-2.124614144	1.377186703	-2.943097638	0.003667241	0.007324988	-2.910383273	DOWN	DDX3Y

IGHGP	1.327342664	0.108690682	2.933742095	0.003774432	0.007519624	-2.875988004	UP	IGHGP

RFX6	1.064171749	-4.635932043	2.930628996	0.003810732	0.007581826	-2.628248766	UP	RFX6

FBXW10	-1.203732905	-2.22398627	-2.930602463	0.003811043	0.007581826	-2.661754121	DOWN	FBXW10

TENM3	1.138722564	-3.209209327	2.927978328	0.003841892	0.007633673	-2.658606148	UP	TENM3

IGKV2_29	1.126465716	-4.533674218	2.91991835	0.003938079	0.007805339	-2.658327054	UP	IGKV2-29

FAM150A	1.022281676	-4.785713775	2.91948474	0.003943315	0.007813129	-2.658966835	UP	FAM150A

GUCY2C	1.278860324	-1.031020533	2.917541565	0.003966861	0.007855877	-2.81457732	UP	GUCY2C

TREML3P	1.139107535	-4.202418194	2.913902083	0.004011308	0.007936013	-2.676659957	UP	TREML3P

RP11_510M2.5	1.154060031	-4.621239374	2.913070552	0.004021527	0.007951625	-2.677146629	UP	RP11-510M2.5

AC079466.1	1.858084784	-1.55404483	2.910094957	0.00405829	0.008015039	-2.804233819	UP	AC079466.1

IGHG1	1.464793882	6.226122711	2.904377422	0.004129793	0.00814751	-3.51275055	UP	IGHG1

GGT6	1.281498027	-2.844804403	2.902060505	0.004159095	0.008198556	-2.745968628	UP	GGT6

NQO1 1.254330707 4.690158858 2.898739058 0.004201433 0.008276557 -3.485595356 UP NQO1

CSAG1 1.480374652 -3.11322537 2.889992829 0.004314813 0.008477564 -2.77335921 UP CSAG1

MUC5B 1.92511188 -0.72043922 2.877754907 0.004478153 0.008770389 -2.961336941 UP MUC5B

AL162759.1 1.050159616 -3.829877636 2.876361113 0.00449711 0.008803191 -2.783512563 UP AL162759.1

MAGEB2 1.415922503 -4.696117213 2.871153926 0.004568583 0.008929215 -2.792861563 UP MAGEB2

CR848007.2 -1.035423858 -4.541344423 -2.870764944 0.004573964 0.008939001 -2.791769352 DOWN CR848007.2

ERICH5 1.358747966 3.094774009 2.86710552 0.004624866 0.009029627 -3.423111309 UP ERICH5

WFDC2 1.385972314 -0.96373954 2.861619291 0.004702149 0.00917068 -2.976704453 UP WFDC2

RP11_468N14.13 1.036999745 -4.187181596 2.856709807 0.004772304 0.009292453 -2.833002633 UP RP11-468N14.13

HOXD9 1.009772886 -0.830730043 2.846258319 0.004924844 0.009559122 -3.02338485 UP HOXD9

FAM19A5 1.35810945 -0.256305755 2.844556437 0.0049501 0.009605025 -3.086235711 UP FAM19A5

SCGB1D2 1.093373943 -4.722341729 2.84305762 0.00497244 0.009645243 -2.868295062 UP SCGB1D2

ARHGAP36 -1.01027247 -5.713100132 -2.842898055 0.004974824 0.009649084 -2.871323233 DOWN ARHGAP36

HAS2 1.025503285 -1.723772044 2.837882648 0.005050282 0.009778789 -2.976246397 UP HAS2

APOL5 -1.050099105 -2.934079877 -2.837412282 0.005057412 0.009790217 -2.891863622 DOWN APOL5

CTD_2325P2.4 -1.016633797 -2.080341538 -2.833308937 0.005120003 0.009898562 -2.93747872 DOWN CTD-2325P2.4

MUM1L1 1.285455626 -2.683926768 2.82998219 0.005171265

0.009988786 -2.94956574 UP MUM1L1

SULT4A1 -1.58817796 -2.403091337 -2.819485365 0.005336091

0.010285565 -2.956933807 DOWN SULT4A1

PRSS51 -1.012195663 -3.049135576 -2.812644617 0.005446067

0.010473071 -2.956160942 DOWN PRSS51

ASPG -1.73703432 2.178603456 -2.802670972 0.005610108

0.010757393 -3.408060066 DOWN ASPG

PLA2G2A -2.041721081 4.611740271 -2.794593808 0.005746237

0.010991982 -3.725086707 DOWN PLA2G2A

BRINP3 1.057461288 -4.907732808 2.778800372 0.006021122

0.011469192 -3.040121546 UP BRINP3

RPS4XP22 -1.100674485 -3.129777175 -2.775253669 0.006084472

0.011575113 -3.054415679 DOWN RPS4XP22

CNTN1 1.268391042 -3.339271786 2.767225582 0.006230108

0.011831466 -3.092049191 UP CNTN1

RP11_397G17.1 1.419249761 -0.166458016 2.767022573 0.006233831

0.011835718 -3.30248382 UP RP11-397G17.1

AC018890.6 1.122520575 -3.386637768 2.745009514 0.006649764

0.012543766 -3.146423264 UP AC018890.6

ZFPM2_AS1 1.128953504 -1.102011573 2.743782176 0.006673678

0.012582922 -3.275867523 UP ZFPM2-AS1

IGHV1_46 1.224348594 -1.924954634 2.736808704 0.00681104

0.012820686 -3.235984106 UP IGHV1-46

XXyac_YM21GA2.7 1.000470677 -4.219461275 2.735728329

0.006832548 0.012855215 -3.154731632 UP XXyac-YM21GA2.7

AL109763.2 1.079553752 -5.027032605 2.73278595 0.006891439

0.012957747 -3.161176146 UP AL109763.2

NRG1 -1.299312953 -0.171271088 -2.728543033 0.006977169

0.013108637 -3.354769622 DOWN NRG1

VAT1L 1.09939363 -0.954320637 2.727980547 0.006988606

0.013127032 -3.329746396 UP VAT1L

SLITRK3 -1.303164319 -3.847587093 -2.725600862 0.007037182

0.013205829 -3.179338254 DOWN SLITRK3

CYS1 1.130681804 -1.040317609 2.724357435 0.007062686 0.013250569 -3.331617776 UP CYS1

SPOCK1 1.018012473 -0.334853011 2.709535871 0.00737319 0.013765091 -3.431519478 UP SPOCK1

OR11Q1P 1.09209709 -4.436671609 2.70706216 0.007426199 0.013856483 -3.228435326 UP OR11Q1P

STMN2 1.420044903 -1.73434755 2.702600943 0.00752267 0.014018988 -3.340536366 UP STMN2

CYP4F60P -1.10855481 -3.840446166 -2.699850396 0.007582712 0.014116899 -3.245929054 DOWN CYP4F60P

RSPO3 -1.071098639 -2.257237314 -2.692077517 0.007754735 0.014393139 -3.2995582 DOWN RSPO3

CTSG -1.095283779 -2.296280964 -2.69009246 0.007799228 0.014471229 -3.302683045 DOWN CTSG

DHRS2 -1.506698952 3.078338253 -2.689042538 0.007822854 0.014510563 -3.843502541 DOWN DHRS2

IGLV3_1 1.238541715 -1.750954074 2.678870256 0.008055127 0.014892894 -3.398398244 UP IGLV3-1

TERT 1.156853776 -1.264658637 2.676926189 0.00810022 0.01496749 -3.436573746 UP TERT

XPNPEP2 -1.533190907 2.200848094 -2.674615657 0.008154109 0.015058438 -3.755132989 DOWN XPNPEP2

DCN -1.084628367 4.689508921 -2.674507633 0.008156637 0.015061943 -4.077544226 DOWN DCN

IGHV3_43 1.021883581 -3.649636846 2.668092991 0.008307989 0.015318956 -3.336980261 UP IGHV3-43

LRP2 1.335016215 -1.694989615 2.666823231 0.008338248 0.015372379 -3.434608308 UP LRP2

HIST1H2AG -1.010735082 0.258472258 -2.65530525 0.008617295 0.015840431 -3.590802964 DOWN HIST1H2AG

COL10A1 1.181598645 -2.143009389 2.64855035 0.008784835 0.016119909 -3.44843488 UP COL10A1

EEF1A2 -1.732478913 3.351992504 -2.647605473 0.008808503

0.01614971 -3.974837258 DOWN EEF1A2

COX6A2 -1.280980242 -1.860879098 -2.642702113 0.008932251 0.016361541 -3.44666364 DOWN COX6A2

PLA2G2D 1.05198605 -1.288799534 2.634784459 0.009135379 0.016695259 -3.541714371 UP PLA2G2D

MRAP2 1.10230385 -0.217334711 2.633776089 0.009161545 0.016734128 -3.638607125 UP MRAP2

IGHV1_18 1.278021974 -1.115954853 2.629142419 0.009282653 0.016932062 -3.572461393 UP IGHV1-18

IGKC 1.237544318 5.2113249392.623136062 0.00944178 0.017189545 -4.243597966 UP IGKC

HOTTIP -1.218594922 -2.217272022 -2.62045962 0.009513474 0.017306896 -3.482663664 DOWN HOTTIP

LGI3 -1.033359955 -2.976696327 -2.613330869 0.009706826 0.017622451 -3.47376396 DOWN LGI3

IGHV1_69 1.069855443 -3.592867894 2.605728271 0.009916915 0.01796976 -3.496468575 UP IGHV1-69

IGHV4_39 1.199557709 -0.762772243 2.600083136 0.010075545 0.018225443 -3.674426138 UP IGHV4-39

SLC22A8 1.064192102 -4.743264924 2.596538569 0.010176308 0.018389631 -3.508010594 UP SLC22A8

SLC22A11 -1.542154806 0.721485207 -2.593297552 0.010269233 0.018540745 -3.784077201 DOWN SLC22A11

DLK1 1.962985894 -1.655658131 2.588525736 0.010407434 0.01876335 -3.650119651 UP DLK1

DIO2 -1.144718401 -1.142721402 -2.587435783 0.010439235 0.018817846 -3.637582114 DOWN DIO2

MUC15 1.171821998 -4.416468673 2.582391501 0.010587544 0.019065072 -3.544087765 UP MUC15

AQP6 -1.330522421 -2.345271178 -2.581718935 0.01060746 0.019096623 -3.572854825 DOWN AQP6

PDPN 1.049983498 -1.470341722 2.56427875 0.01113578 0.01997109 -3.701851578 UP PDPN

CEACAM6 1.215024668 -3.629311612 2.546809461 0.011688483 0.020880897 -3.643189838 UP CEACAM6

LINC01207 1.187806943 -4.00264614 2.5411855280.0118715660.02118265 -3.648994378 UP LINC01207

IGHV4_34 1.03622876 -1.693264589 2.54041379 0.0118968880.021221501 -3.744946 UP IGHV4-34

HPGD -1.408197034 3.86976203 -2.533972193 0.0121101420.021571331 -4.343969181 DOWN HPGD

IGHG3 1.165524128 3.213087963 2.532293022 0.01216629 0.0216678 -4.309062025 UP IGHG3

ISX 1.48610621 -1.058885779 2.51745016 0.012672809 0.022473165 -3.858524557 UP ISX

LINC01549 -1.045746182 -2.396997931 -2.513993652 0.012793432 0.022668811-3.737187727 DOWN LINC01549

CDH16 1.162711239-1.834258832 2.513989967 0.012793562 0.022668811-3.80210067 UP CDH16

LINC00221 1.427973557 -3.740142972 2.5118346920.012869293 0.022792868 -3.728011858 UP LINC00221

MGAT4C -1.061330973 -2.376465524 -2.511156062 0.012893221 0.022826795 -3.744828555 DOWN MGAT4C

CDH19 -1.063119237 -1.876061032 -2.504816488 0.013118676 0.023191616 -3.788343091 DOWN CDH19

IGHG2 1.140438147 3.153798225 2.500977496 0.013256905 0.02340657 -4.37825072 UP IGHG2

GLT1D1 -1.157080797 2.76164082 -2.494050025 0.013509627 0.023808838 -4.298851328 DOWN GLT1D1

CAPN13 1.17058543 -3.239498234 2.486006025 0.013808459 0.024285382 -3.801024009 UP CAPN13

VSTM2L -1.03826011 0.251190299-2.480340895 0.014022434 0.02462549 -4.020631496 DOWN VSTM2L

EGF 1.06633747 -3.398860904 2.47072992 0.014392201 0.025211625-3.82975422 UP EGF

RAB3B 1.009218089 1.877780316 2.4688988 0.014463625

0.025327759 -4.286416377 UP RAB3B

TMPRSS15 1.117426801 -4.675544432 2.467692792 0.014510839

0.025399278 -3.821144016 UP TMPRSS15

IGHV1_24 1.131117098 -2.526277543 2.466420447 0.014560798

0.025473675 -3.87615639 UP IGHV1-24

NLGN4Y -1.311427829 -1.438097469 -2.461305687 0.014763176

0.025793768 -3.918754049 DOWN NLGN4Y

ADCY8 -1.197179802 -3.682420796 -2.459405773 0.014838986

0.025912969 -3.840752549 DOWN ADCY8

FAM133A 1.297907348 -2.879029408 2.459351547 0.014841154

0.025913753 -3.879437637 UP FAM133A

MINOS1P3 -1.00939429 -4.21561171 -2.458382366 0.014879964 0.02597166

-3.841805359 DOWN MINOS1P3

SEZ6 1.208600092 -2.52712372 2.44681318 0.015350247 0.026685775

-3.924471987 UP SEZ6

OTOG 1.056307814 -4.106853562 2.417298587 0.016610263

0.028675304 -3.941888546 UP OTOG

GPR37 -1.015342933 2.281420575 -2.415549096 0.016687747

0.028790366 -4.419165465 DOWN GPR37

PADI1 -1.20629008 -3.010076167 -2.410351194 0.01691985 0.029159249

-3.962785168 DOWN PADI1

MAPK4 -1.087769868 -0.65542409 -2.409803864 0.016944456 0.02919324

-4.105742949 DOWN MAPK4

HRASLS2 -1.044784189 -1.790674421 -2.409216271 0.016970907

0.029234599 -4.020631457 DOWN HRASLS2

PDZK1IP1 -1.288358263 2.901327998 -2.400445245 0.017370104

0.029862046 -4.526626665 DOWN PDZK1IP1

SYT3 1.004305816 -2.774779722 2.399600896 0.017408968

0.029924557 -4.018611551 UP SYT3

IGLV3_25 1.077628507 -0.966837273 2.39589065 0.017580655

0.030193633 -4.146709371 UP IGLV3-25

TRHDE 1.147531204 -2.168981678 2.374739673 0.018588263

0.031755392 -4.107926668 UP TRHDE

ST8SIA6_AS1 -1.341390773 -0.99617454 -2.374387482 0.018605464 0.031781746 -4.154139514 DOWN ST8SIA6-AS1

RP11_169F17.1 1.085564294 -4.027136795 2.374198949 0.018614677 0.031791433 -4.042343139 UP RP11-169F17.1

AQP4 -1.015293272 -1.503399124 -2.368459756 0.018897081 0.03222312 -4.133834596 DOWN AQP4

DPEP1 1.012629726 -1.460000858 2.357852219 0.019428992 0.033045314 -4.192977949 UP DPEP1

PI15 1.017480575 -1.320490912 2.345022036 0.020089945 0.034053241 -4.232772466 UP PI15

CST1 1.27649611 -2.997895768 2.3211965060.021369959 0.036033934 -4.194528777 UP CST1

XIST 1.64438601 -1.819562501 2.306921251 0.022170726 0.037252863 -4.292740246 UP XIST

TBX4 1.094893558 -2.845444687 2.306377387 0.022201748 0.037291883 -4.228937872 UP TBX4

CCL25 -1.129580492 -1.456875481 -2.285800021 0.023403759 0.039096717 -4.32317697 DOWN CCL25

BCL2L10 -1.214577078 0.349410635 -2.270871206 0.024311098 0.040423285 -4.509558475 DOWN BCL2L10

LINC01291 1.050838842 -1.815501186 2.223661724 0.027385661 0.044966555 -4.464552401 UP LINC01291

IGHV1_69_2 1.023967895 -2.287792419 2.212994365 0.02812549 0.046039104 -4.458148901 UP IGHV1-69-2

IGLV1_44 1.004568462 -0.647854351 2.202357939 0.028880404 0.047152203 -4.605004355 UP IGLV1-44

IGKV3_11 1.052074003 0.890181006 2.199648333 0.029075504 0.047425413 -4.771684509 UP IGKV3-11

SIK1 -1.060904424 -0.560060697 -2.19482761 0.02942544 0.047937351 -4.592673939 DOWN SIK1

SLC44A5 1.025892104 -0.777911647 2.189069404 0.02984821 0.048556634 -4.61999146 UP SLC44A5

USP9Y -1.245299188 -0.85765001 -2.188834521 0.029865566

0.048581565 -4.576871868 DOWN USP9Y
CLRN3 -1.173085241 1.032456441 -2.184739281 0.030169588
0.049019431 -4.770696884 DOWN CLRN3

Supplementary table 4.

Genes	logFC	AveExpr	t	P.Value	adj.P.Val	B	change	symbol
TMEM220_AS1	-2.610716517	1.552081725	-13.62473679	1.07E-29	9.63E-28	56.84718017	DOWN	TMEM220-AS1
LINC01485	-5.177320491	3.693786571	-12.90047952	1.50E-27	1.00E-25	51.94867687	DOWN	LINC01485
F11_AS1	-2.65114592	1.844298326	-12.84881068	2.13E-27	1.39E-25	51.61459201	DOWN	F11-AS1
NAV2_AS4	-2.891996048	-4.446634994	-12.71370542	5.36E-27	3.27E-25	50.56000815	DOWN	NAV2-AS4
B4GALT1_AS1	-1.916170882	-0.061209722	-12.52477327	1.94E-26	1.11E-24	49.41616899	DOWN	B4GALT1-AS1
MIR100HG	-3.160404513	-2.197376231	-12.49591287	2.36E-26	1.34E-24	49.14663809	DOWN	MIR100HG
SPATA3_AS1	2.529979381	-4.071152171	12.30132518	8.88E-26	4.70E-24	47.83602843	UP	SPATA3-AS1
SNHG1	1.273694319	4.051829248	12.2626809	1.15E-25	6.02E-24	47.51825507	UP	SNHG1
DDX11_AS1	1.859397094	-1.905771087	12.24504312	1.30E-25	6.68E-24	47.51197676	UP	DDX11-AS1
AC016682.1	-2.631201432	-4.980181801	-12.20113595	1.75E-25	8.87E-24	47.15286631	DOWN	AC016682.1
PRKAG2_AS1	-2.305049206	1.450046793	-11.96106685	8.95E-25	4.20E-23	45.64975488	DOWN	PRKAG2-AS1
LINC01093	-4.710648414	-0.782134014	-11.7308983	4.25E-24	1.83E-22	44.09112617	DOWN	LINC01093
LINC01354	-2.986262197	-2.74313591	-11.49231803	2.13E-23	8.44E-22	42.47624686	DOWN	LINC01354
LHFPL3_AS2	4.085957775	-3.172211825	11.34549436	5.73E-23	2.14E-21	41.53723919	UP	LHFPL3-AS2
LAMA5_AS1	-4.636406282	0.337019435	-11.30643823	7.45E-23	2.76E-21	41.29333507	DOWN	LAMA5-AS1
LINC01352	-2.527777483	-2.891785171	-11.23840448	1.18E-22	4.27E-			

21 40.79950248 DOWN LINC01352

TPRG1_AS1 -3.391914978 0.613461914 -11.07160642 3.61E-22 1.23E-20 39.74250355 DOWN TPRG1-AS1

LINC00205 1.371233422 0.396670377 11.061408413.86E-22 1.31E-20 39.66514494 UP LINC00205

LDLRAD4_AS1 -3.038730756 -4.376193098 -10.72867652 3.57E-21 1.08E-19 37.45728126 DOWN LDLRAD4-AS1

LINC01537 -1.812687574 -2.425869132 -10.54450705 1.21E-20 3.35E-19 36.26861444 DOWN LINC01537

PIK3CD_AS2 2.761885699 -2.337880567 10.3352736 4.85E-20 1.25E-18 34.92539982 UP PIK3CD-AS2

LINC01121 2.730481751 -3.640841675 10.16904268 1.45E-19 3.54E-18 33.84563108 UP LINC01121

PRR7_AS1 1.945640282 -3.4645782 10.047044 3.23E-19 7.52E-18 33.06001843 UP PRR7-AS1

LINC00844 -5.839876554 0.314128351 -9.911705386 7.83E-19 1.76E-17 32.19285742 DOWN LINC00844

LINC00402 -2.891566977 -3.41735828 -9.897406419 8.60E-19 1.92E-17 32.09888797 DOWN LINC00402

GNG12_AS1 -1.812069747 -1.633496947 -9.893279659 8.83E-19 1.96E-17 32.0789983 DOWN GNG12-AS1

USP30_AS1 -2.006642126 0.968935141 -9.835211394 1.29E-18 2.79E-17 31.66811152DOWN USP30-AS1

FAM225A 2.133641486 -3.084468135 9.831703421 1.32E-18 2.85E-17 31.68514522 UP FAM225A

SNHG3 1.557049918 3.164504378 9.828355272 1.35E-18 2.91E-17 31.42981032 UP SNHG3

LINC00526 -1.520427664 2.169458606 -9.574562391 7.00E-18 1.36E-16 29.92394273 DOWN LINC00526

BDNF_AS -1.272211202 0.680764912 -9.56328169 7.53E-18 1.45E-16 29.9359492 DOWN BDNF-AS

LINC01127 -2.501952916 1.347425243 -9.556522468 7.86E-18 1.51E-16 29.87501934 DOWN LINC01127

AC026202.3 -1.54162478 -1.382254167 -9.512425127 1.05E-17 1.97E-16 29.65814293 DOWN AC026202.3
MIR99AHG -2.046157693 0.943494854 -9.503835246 1.10E-17 2.07E-16 29.55414554 DOWN MIR99AHG
C2orf27A 1.871711239 -0.730737451 9.447725254 1.59E-17 2.92E-16 29.22978879 UP C2orf27A
LINC01018 -5.848807973 2.241505116 -9.422605464 1.86E-17 3.40E-16 29.03196115 DOWN LINC01018
RAB11B_AS1 -1.513810517 2.995540375 -9.400973508 2.14E-17 3.86E-16 28.74394912 DOWN RAB11B-AS1
SIAH2_AS1 -2.001509711 -2.733216233 -9.364048856 2.71E-17 4.81E-16 28.72566553 DOWN SIAH2-AS1
HULC -3.390888654 5.913021819 -9.215916849 7.00E-17 1.16E-15 27.40807136 DOWN HULC
F10_AS1 -1.831833412 -3.12566329 -9.164748777 9.71E-17 1.59E-15 27.4808119 DOWN F10-AS1
TAF1A_AS1 -1.286353091 0.689034496 -9.067975465 1.80E-16 2.84E-15 26.81376631 DOWN TAF1A-AS1
LINC00665 2.541820459 1.966410836 9.062392938 1.86E-16 2.94E-15 26.65379845 UP LINC00665
ALDH1L1_AS2 -2.870565672 -0.49997766 -9.055549701 1.94E-16 3.06E-15 26.78607586 DOWN ALDH1L1-AS2
MIR210HG 1.762389479 0.627580569 8.884257434 5.73E-16 8.52E-15 25.64310011 UP MIR210HG
FBXL19_AS1 1.45726398 -0.309977374 8.819368778 8.62E-16 1.26E-14 25.29177366 UP FBXL19-AS1
SMIM2_AS1 -2.083680778 0.901775275 -8.774552618 1.14E-15 1.63E-14 24.9913032 DOWN SMIM2-AS1
LINC01224 3.297391375 -3.464814356 8.774438983 1.14E-15 1.63E-14 25.06531701 UP LINC01224
LINC00494 3.368155811 -3.548318347 8.761496679 1.24E-15 1.76E-14 24.98627149 UP LINC00494
DHRS4_AS1 -1.117467854 3.939351269 -8.711361639 1.70E-15

2.38E-14 24.31707175 DOWN DHRS4-AS1

SNHG4 1.877516086 -1.30807511 8.655472315 2.40E-15 3.30E-14 24.31664114UP SNHG4

HNF4A_AS1 -3.523725885 0.720650007 -8.646432037 2.54E-15 3.48E-14 24.22759477 DOWN HNF4A-AS1

AC005592.2 1.85516781 -4.135329159 8.630129411 2.82E-15 3.83E-14 24.19277531 UP AC005592.2

TMEM51_AS1 2.522759758 -2.513704671 8.541324775 4.89E-15 6.46E-14 23.63911969UP TMEM51-AS1

LINC00261 -1.888621764 6.53965794 -8.507365445 6.03E-15 7.85E-14 22.95809046 DOWN LINC00261

LINC01554 -5.474272069 1.74920678 -8.432205333 9.60E-15 1.22E-13 22.90339343 DOWN LINC01554

SNHG12 1.01275245 2.267574815 8.406194681 1.13E-14 1.42E-13 22.58406944 UP SNHG12

CPB2_AS1 -1.460373924 -0.574509466 -8.401783894 1.16E-14 1.45E-13 22.77384282 DOWN CPB2-AS1

LINC01588 -1.507096508 0.953960226 -8.390312702 1.24E-14 1.55E-13 22.63519261 DOWN LINC01588

DGUOK_AS1 1.225955484 -2.052041617 8.38677209 1.27E-14 1.58E-13 22.70693093 UP DGUOK-AS1

UPK1A_AS1 2.574948444 -4.106900393 8.373006675 1.38E-14 1.72E-13 22.63780711UP UPK1A-AS1

HAO2_IT1 -2.266446896 -4.756392297 -8.340675666 1.69E-14 2.07E-13 22.44804139 DOWN HAO2-IT1

LINC01344 -2.548829406 -2.405066742 -8.300720481 2.15E-14 2.61E-13 22.20319861 DOWN LINC01344

AC144652.1 -1.501157317 0.51574211 -8.225438069 3.41E-14 3.99E-13 21.66756933 DOWN AC144652.1

TAT_AS1 -2.069426835 -1.6719156 -8.208665328 3.78E-14 4.38E-13 21.6463601 DOWN TAT-AS1

TRAM2_AS1 -1.009346563 2.610303508 -8.193902243 4.14E-14 4.77E-13 21.3013144 DOWN TRAM2-AS1

BOLA3_AS1 1.534489595 -1.920095015 8.136975872 5.85E-14
6.61E-13 21.20948481 UP BOLA3-AS1

BOK_AS1 -2.552210922 -2.912093348 -8.13263862 6.01E-14 6.77E-13
21.20611334DOWN BOK-AS1

LINC00992 2.972911553-3.443129814 8.050158637 9.90E-14 1.09E-12
20.70789337 UP LINC00992

LINC00324 -1.049941312 1.389268397 -8.041502828 1.04E-13 1.14E-
12 20.50672973 DOWN LINC00324

AC159540.1 1.644251249 -3.828820939 8.02164534 1.18E-13 1.28E-12
20.55184957 UP AC159540.1

LINC00574 -1.694685973 -0.449631728 -7.918901015 2.18E-13 2.28E-
12 19.89569299 DOWN LINC00574

DNMBP_AS1 -2.012435889 -0.815242161 -7.862466034 3.06E-13
3.14E-12 19.58173786 DOWN DNMBP-AS1

MYLK_AS11.209751008 -1.270246902 7.85871457 3.13E-13 3.20E-12
19.55308033 UP MYLK-AS1

LINC01252 -2.04835695-1.673223817 -7.804595385 4.32E-13 4.36E-12
19.26804432 DOWN LINC01252

AC008592.4-2.552595334 -2.873793538 -7.79258136 4.64E-13 4.66E-12
19.21227162 DOWN AC008592.4

AC099552.2-1.609087514 -5.82545587-7.770726225 5.29E-13 5.28E-12
19.08971681 DOWN AC099552.2

TMC3_AS1 -1.546835616 -1.025263165 -7.761228308 5.60E-13 5.57E-
12 18.99576211DOWN TMC3-AS1

CATIP_AS1-1.683633944 -1.775412013 -7.7373202 6.45E-13 6.34E-12
18.87913851 DOWN CATIP-AS1

CLRN1_AS1 -2.122325092 -4.612192115 -7.707173432 7.71E-13
7.49E-12 18.72372819 DOWN CLRN1-AS1

CPS1_IT1 -2.441569273 -3.597378594 -7.703891111 7.86E-13 7.63E-
12 18.70372104 DOWN CPS1-IT1

AC016735.12.597683159 -3.391950255 7.686280563 8.73E-13 8.42E-
12 18.58681011UP AC016735.1

LINC00886 -1.825977461 1.262722253 -7.670870057 9.56E-13 9.16E-

12 18.35348019 DOWN LINC00886

MIR181A2HG 1.706986188 -3.459524301 7.623587724 1.26E-12 1.19E-11 18.23438299 UP MIR181A2HG

MGC32805 -2.948037645 0.11001085 -7.612008049 1.35E-12 1.27E-11 18.0986743 DOWN MGC32805

RAP2C_AS1 -1.12849431 -0.829842011 -7.592193336 1.52E-12 1.42E-11 18.00916524 DOWN RAP2C-AS1

FAM83A_AS1 -4.629776982 -1.155552782 -7.571402419 1.72E-12 1.60E-11 17.91037969 DOWN FAM83A-AS1

FOXD2_AS1 1.530999952 0.910593075 7.48955648 2.77E-12 2.50E-11 17.28587738 UP FOXD2-AS1

CASC15 2.064710221 -0.908388223 7.489008459 2.78E-12 2.51E-11 17.39483864 UP CASC15

IL12A_AS1 1.492371887 -5.295979829 7.464341776 3.21E-12 2.86E-11 17.33460473 UP IL12A-AS1

HS1BP3_IT1 -2.125525912 0.705536854 -7.419297485 4.18E-12 3.66E-11 16.95082088 DOWN HS1BP3-IT1

LINC00628 1.572463565 -3.979506957 7.370486196 5.54E-12 4.75E-11 16.80115847UP LINC00628

GUSBP11 1.200266886 -0.348022236 7.357734227 5.97E-12 5.09E-11 16.62629788 UP GUSBP11

OSTN_AS1 -2.693122088 -2.912443745 -7.355435074 6.05E-12 5.15E-11 16.71274688 DOWN OSTN-AS1

AC006538.1 1.499196156 -2.445865927 7.342891846 6.50E-12 5.52E-11 16.62295852 UP AC006538.1

MIR600HG 1.213315471 0.659122172 7.322006143 7.34E-12 6.18E-11 16.35603156 UP MIR600HG

NRSN2_AS1 1.03669248 -0.789389718 7.320820662 7.39E-12 6.22E-11 16.44366909 UP NRSN2-AS1

VAC14_AS1 2.232235751 -1.228721077 7.2974464118.46E-12 7.07E-11 16.32124386 UP VAC14-AS1

LINC00654 1.648349217 -0.982758798 7.284746638 9.10E-12 7.57E-11 16.24432621 UP LINC00654

LINC00941 2.189011879-2.984982453 7.282218465 9.23E-12 7.68E-11 16.2856228 UP LINC00941

LINC00632 2.200884789 -4.867808598 7.243057269 1.16E-11 9.48E-11 16.08855254 UP LINC00632

MNX1_AS1 2.919955791 -4.484934532 7.219535497 1.32E-11 1.08E-10 15.95102531 UP MNX1-AS1

AC008592.3 -1.232577896 -1.351010711 -7.213049937 1.37E-11 1.11E-10 15.88225946 DOWN AC008592.3

LINC01044 -1.431363417 -5.838084415 -7.188590669 1.58E-11 1.28E-10 15.78511614DOWN LINC01044

LINC00570 -2.364715606 -3.155960175 -7.188065227 1.58E-11 1.28E-10 15.77964891 DOWN LINC00570

RNF144A_AS1 2.166820691 -2.939421977 7.170720757 1.75E-11 1.41E-10 15.66243837 UP RNF144A-AS1

AL133493.2 2.238075547 -3.496851537 7.154739816 1.92E-11 1.53E-10 15.58319941 UP AL133493.2

LINC01106 1.655241641 -2.54189175 7.08129699 2.91E-11 2.27E-10 15.16453838 UP LINC01106

LINC01353 1.499385971 -3.862035457 7.05224119 3.43E-11 2.65E-10 15.02843819 UP LINC01353

C15orf56 1.503893566 -5.235985184 7.0208911224.10E-11 3.12E-10 14.86053685 UP C15orf56

FAM99A -4.090875761 0.703007164 -7.012996511 4.28E-11 3.25E-10 14.70299115DOWN FAM99A

DBH_AS1 -1.51885094 3.030890965 -7.009309715 4.37E-11 3.32E-10 14.41431395 DOWN DBH-AS1

HM13_AS1 -1.293985715 -2.753336476 -7.006307609 4.45E-11 3.37E-10 14.77526247 DOWN HM13-AS1

MIR325HG -2.625583823 -3.337563667 -6.99753757 4.67E-11 3.54E-10 14.72926076 DOWN MIR325HG

KANSL1_AS1 -1.256414802 0.789101057 -6.993357088 4.79E-11 3.61E-10 14.54643924 DOWN KANSL1-AS1

LINC00511 2.43986247 0.064860431 6.983278352 5.06E-11 3.81E-10

14.49434966 UP LINC00511

MLIP_AS1 -2.135797936 -3.811180614 -6.970128197 5.45E-11 4.09E-10 14.58286479 DOWN MLIP-AS1

JMJD1C_AS1 1.221787032 -2.49306639 6.957755313 5.85E-11 4.36E-10 14.48942279 UP JMJD1C-AS1

LINC00664 1.759509697 -3.726106034 6.947145618 6.21E-11 4.61E-10 14.44947428 UP LINC00664

MGC27382 -1.751207694 -1.723457395 -6.914707067 7.44E-11 5.47E-10 14.25299372 DOWN MGC27382

AC092071.1 -2.348094258 -4.411383401 -6.862162787 9.98E-11 7.20E-10 13.99616317 DOWN AC092071.1

DLX6_AS1 2.713751693 -4.093260048 6.84798914 1.08E-10 7.75E-10 13.90687572 UP DLX6-AS1

FAM182B 1.50607162 -2.987553128 6.823538387 1.24E-10 8.80E-10 13.76825811 UP FAM182B

JAKMIP2_AS1 -2.220836869 -4.47592811 -6.818396924 1.27E-10 9.04E-10 13.75988381 DOWN JAKMIP2-AS1

LINC01558 -1.883694881 1.069243583 -6.801945155 1.40E-10 9.83E-10 13.48918785 DOWN LINC01558

ITIH4_AS1 -1.499615223 -2.064518915 -6.79717844 1.43E-10 1.01E-09 13.62539509 DOWN ITIH4-AS1

RNF217_AS1 -1.543929783 -2.701556512 -6.784846183 1.53E-10 1.08E-09 13.5719147 DOWN RNF217-AS1

LINC00342 1.018772743 0.985879428 6.778885847 1.59E-10 1.11E-09 13.32286124 UP LINC00342

U91324.1 -1.959398066 -0.737450991 -6.761513255 1.75E-10 1.21E-09 13.38517653 DOWN U91324.1

NCOA7_AS1 -1.636524526 -5.149750228 -6.760296771 1.76E-10 1.22E-09 13.44717138 DOWN NCOA7-AS1

AC104809.2 -3.275739296 -2.875426573 -6.715456034 2.25E-10 1.54E-09 13.19508551 DOWN AC104809.2

GAS5 1.011346989 6.497189534 6.703454126 2.41E-10 1.64E-09 12.51204927 UP GAS5

LINC00676 -1.346865296 -5.830559821 -6.700373324 2.45E-10 1.66E-09 13.12508692 DOWN LINC00676

TUSC8 -2.542026754 0.422284852 -6.669807007 2.89E-10 1.95E-09 12.83348174 DOWN TUSC8

LINC00399 -1.640793639 -4.595839726 -6.646634889 3.29E-10 2.20E-09 12.8413837 DOWN LINC00399

ST3GAL6_AS1 -1.257815282 -1.812848103 -6.640249935 3.40E-10 2.27E-09 12.77659505 DOWN ST3GAL6-AS1

RNU6ATAC35P -1.499135831 -2.805301306 -6.633004885 3.54E-10 2.35E-09 12.76232179 DOWN RNU6ATAC35P

ADORA2A_AS1 -1.799232991 3.689835426 -6.622836743 3.74E-10 2.48E-09 12.24075957 DOWN ADORA2A-AS1

LINC01138 1.010306982 1.392254563 6.601930504 4.20E-10 2.76E-09 12.33300405 UP LINC01138

AP001626.1 2.3608573 -1.733736234 6.590755799 4.46E-10 2.92E-09 12.47630825 UP AP001626.1

THRB_AS1 -1.135106272 -1.104504644 -6.589406248 4.49E-10 2.94E-09 12.4756612 DOWN THRB-AS1

RUNDC3A_AS1 1.350016379 -4.43428046 6.582385888 4.67E-10 3.04E-09 12.50017061 UP RUNDC3A-AS1

KCNQ1OT1 1.189527386 0.71245796 6.549820735 5.57E-10 3.59E-09 12.1163609 UP KCNQ1OT1

SLC25A30_AS1 -1.263462703 -2.028913321 -6.535516277 6.02E-10 3.87E-09 12.23001914 DOWN SLC25A30-AS1

ZNF528_AS1 1.240236167 -0.082598023 6.488489652 7.77E-10 4.91E-09 11.85532902UP ZNF528-AS1

HAGLR2.490667856 0.379435107 6.463951527 8.87E-10 5.54E-09 11.67346967UP HAGLR

COLCA1 1.762313484 0.274823804 6.450503682 9.53E-10 5.93E-09 11.62131654UP COLCA1

AC018647.3 -1.327870146 -2.311913072 -6.443353096 9.91E-10 6.15E-09 11.75582915DOWN AC018647.3

CASC8 1.814147825 -4.499873177 6.434844178 1.04E-09 6.41E-09

11.72562083UP CASC8

LINC01348 -2.579109255 2.172602749 -6.408794212 1.19E-09 7.30E-09 11.30669389DOWN LINC01348

FAM99B -2.877711786 -1.404402837 -6.394480886 1.29E-09 7.83E-09 11.4773236 DOWN FAM99B

AP000997.2 -1.682881291 -4.829462695 -6.379809276 1.39E-09 8.43E-09 11.4421185 DOWN AP000997.2

LINC01191 1.501618047 -4.609480595 6.375265327 1.43E-09 8.62E-09 11.4177895 UP LINC01191

HCG15 1.417885291 -2.980939298 6.373684388 1.44E-09 8.68E-09 11.38890141UP HCG15

SPATA41 -1.652888813 0.364139594 -6.345686985 1.67E-09 9.95E-09 11.11747347DOWN SPATA41

LINC01535 1.903468369 -2.949704163 6.344999525 1.68E-09 9.98E-09 11.23278533UP LINC01535

BCRP3 1.525840567 -1.594176515 6.310763466 2.01E-09 1.18E-08 11.01628757UP BCRP3

LINC01094 1.404990458 -1.981571726 6.303896185 2.09E-09 1.22E-08 10.9995592 UP LINC01094

LINC01151 -2.531250291 -1.252916658 -6.287048891 2.28E-09 1.33E-08 10.91575279 DOWN LINC01151

AC006369.2 -1.488230254 -3.838605607 -6.27637001 2.42E-09 1.40E-08 10.90964462 DOWN AC006369.2

SLCO4A1_AS1 2.014899574 -4.617998752 6.273041774 2.46E-09 1.42E-08 10.88992217 UP SLCO4A1-AS1

GAS6_AS1 -1.618355968 1.097895285 -6.268300301 2.52E-09 1.46E-08 10.65971845 DOWN GAS6-AS1

MAFA_AS1 1.981014116-4.536331744 6.234479528 3.01E-09 1.72E-08 10.69255662 UP MAFA-AS1

OGFR_AS1 -1.130141851 -2.062776038 -6.19229011 3.76E-09 2.12E-08 10.4545884 DOWN OGFR-AS1

A1BG_AS1 -1.204443735 0.290075701 -6.175730726 4.10E-09 2.30E-08 10.24116061DOWN A1BG-AS1

CTD_2201I18.1 -1.341159807 -1.412793887 -6.13670274 5.03E-09 2.79E-08 10.1469974 DOWN CTD-2201I18.1

LOXL1_AS1 1.7534797 -1.750120364 6.133821303 5.11E-09 2.83E-08 10.11698115 UP LOXL1-AS1

PGM5_AS1 -1.496818628 -4.92862378 -6.12908401 5.24E-09 2.89E-08 10.16087339 DOWN PGM5-AS1

LINC01123 1.665572435 -3.430066023 6.104379982 5.96E-09 3.25E-08 10.0203984 UP LINC01123

LINC00884 -1.036042054 1.280966416 -6.103016033 6.00E-09 3.27E-08 9.785365642 DOWN LINC00884

TFAP2A_AS1 1.956582804 -3.067952275 6.097576328 6.17E-09 3.37E-08 9.973198909 UP TFAP2A-AS1

EP300_AS1 -1.547149516 -1.950512515 -6.091704236 6.37E-09 3.46E-08 9.943682097 DOWN EP300-AS1

ZNF350_AS1 1.14040566 -4.108762826 6.078757278 6.81E-09 3.68E-08 9.90586877 UP ZNF350-AS1

UBXN10_AS1 -1.77820033 -2.965374472 -6.053250746 7.77E-09 4.16E-08 9.773609812 DOWN UBXN10-AS1

MIR137HG 1.347609508 -5.774763462 6.044182032 8.14E-09 4.34E-08 9.733082358 UP MIR137HG

PTPRG_AS1 1.445948307 -2.917067736 5.984499052 1.11E-08 5.79E-08 9.411279531 UP PTPRG-AS1

LINC01428 -1.695355349 -3.313269338 -5.97475676 1.17E-08 6.07E-08 9.386457469 DOWN LINC01428

LINC01564 -1.554565042 -0.069479874 -5.924511704 1.51E-08 7.73E-08 9.009041876 DOWN LINC01564

SATB1_AS1 1.654323475 -3.106415342 5.845194518 2.26E-08 1.13E-07 8.726430635 UP SATB1-AS1

LINC01096 1.249218392 -5.842319458 5.843614763 2.27E-08 1.14E-07 8.741779427 UP LINC01096

ZNF790_AS1 1.240524823 -1.665843238 5.812912722 2.66E-08 1.32E-07 8.52321524 UP ZNF790-AS1

SOCS2_AS1 -1.275919811 0.086631768 -5.797631585 2.87E-08

1.42E-07	8.370867985	DOWN SOCS2-AS1

ARHGEF7_AS2 -1.438884442	-5.024627706	-5.781504406	3.11E-08
1.53E-07	8.441038561	DOWN ARHGEF7-AS2

RGMB_AS1-1.386918926	-1.040802931	-5.775455765	3.21E-08	1.57E-07	8.335578168	DOWN RGMB-AS1

MAGI2_AS3	-1.741402693	2.220271376	-5.766816499	3.35E-08
1.63E-07	8.029340177	DOWN MAGI2-AS3

AC092198.1 1.300729586	-5.141310264	5.720682684	4.22E-08	2.03E-07	8.146769228	UP AC092198.1

ZIM2_AS1 1.597751035	-4.2210595 5.704668093	4.57E-08	2.19E-07
8.066675426	UP ZIM2-AS1

UPP2_IT1	-1.74663423 -5.153704424	-5.698116287	4.73E-08	2.25E-07
8.037951576	DOWN UPP2-IT1

LINC00671 -1.46244092 0.742549461	-5.68030591 5.16E-08	2.45E-07
7.751211839DOWN LINC00671

LINC00265 1.162328026	-1.173329901	5.664637658	5.58E-08	2.63E-07	7.779386138	UP LINC00265

SNHG14	1.501488141	3.402689961	5.659121217	5.74E-08	2.70E-07	7.308641001	UP SNHG14

DGCR5 -2.033243615	1.967776743	-5.635252016	6.46E-08	3.01E-07
7.427143048	DOWN DGCR5

ZFY_AS1	-1.851333761	-4.142809954	-5.620802141	6.94E-08	3.22E-07	7.669619889	DOWN ZFY-AS1

STXBP5_AS1	1.073738856	-1.969173234	5.57165987 8.84E-08	4.03E-07	7.379539187	UP STXBP5-AS1

PLS3_AS1	-1.101386846	-1.638637218	-5.561905493	9.27E-08	4.22E-07	7.340629516	DOWN PLS3-AS1

FOXP4_AS1	1.452222313	-2.332979468	5.558557443	9.42E-08
4.28E-07	7.325983742	UP FOXP4-AS1

LINC00840 -1.37729873 -4.158288408	-5.557634509	9.47E-08	4.29E-07
7.370823553	DOWN LINC00840

LINC01572 1.284909162	-3.312471092	5.554477685	9.61E-08	4.36E-07	7.3396609	UP LINC01572

LINC00501 1.430657284 -5.06160959 5.551700947 9.74E-08 4.41E-07 7.341574033 UP LINC00501
AC007952.5 1.330844143 -5.149798988 5.536625843 1.05E-07 4.73E-07 7.270630643 UP AC007952.5
LINC01063 1.127775118 -4.14382108 5.535252198 1.06E-07 4.76E-07 7.262909954 UP LINC01063
AL161645.1 -1.242197452 -5.47956641 -5.532086474 1.07E-07 4.83E-07 7.248663219 DOWN AL161645.1
LINC00639 -1.110782944 -2.285636053 -5.527874456 1.10E-07 4.92E-07 7.20811263 DOWN LINC00639
RRS1_AS1 -1.232279302 -2.998812333 -5.523836763 1.12E-07 5.00E-07 7.20595214 DOWN RRS1-AS1
LINC01506 -1.756898723 -3.390849092 -5.506310757 1.22E-07 5.42E-07 7.126534892 DOWN LINC01506
LINC00659 -2.409928066 -1.329906683 -5.457008806 1.55E-07 6.80E-07 6.843025249 DOWN LINC00659
PRSS30P 1.28451149 -3.046055204 5.448117592 1.61E-07 7.07E-07 6.833989348 UP PRSS30P
TNRC6C_AS1 1.058287633 0.651758237 5.438947529 1.69E-07 7.37E-07 6.568870793 UP TNRC6C-AS1
LINC00622 1.078346359 -2.151455859 5.423517698 1.82E-07 7.91E-07 6.692874105 UP LINC00622
BBOX1_AS1 2.062063815 -3.949599976 5.406497409 1.97E-07 8.55E-07 6.651405969 UP BBOX1-AS1
AC006037.2 -1.411407211 -4.962684399 -5.403327446 2.00E-07 8.67E-07 6.649523929 DOWN AC006037.2
HCG14 -1.330575505 -3.843502677 -5.38544708 2.18E-07 9.40E-07 6.567165315 DOWN HCG14
MIR663AHG 1.842713815 -5.206755218 5.373260685 2.31E-07 9.93E-07 6.509268441 UP MIR663AHG
USP2_AS1 -1.490350771 -0.453921682 -5.351953827 2.56E-07 1.09E-06 6.294205368 DOWN USP2-AS1
AC012668.2 -1.504684335 -3.250243768 -5.346067739 2.64E-07 1.12E-

06	6.382743193	DOWN	AC012668.2
ZNF571_AS1	1.141161412	-2.809051692	5.334180394	2.79E-07	1.18E-06	6.302772868	UP	ZNF571-AS1
LINC01482	-1.401721662	-2.425190121	-5.324122146	2.93E-07	1.23E-06	6.267542527	DOWN	LINC01482
LINC00398	-1.398750957	-4.274831056	-5.305089417	3.21E-07	1.34E-06	6.199007233	DOWN	LINC00398
LINC00940	-1.521229223	-4.613195196	-5.260850991	3.95E-07	1.63E-06	5.997587339	DOWN	LINC00940
LINC01277	-1.086938171	-2.16916277	-5.242273987	4.32E-07	1.77E-06	5.885005879	DOWN	LINC01277
FAM27E3	1.102346687	-4.039626606	5.238919044	4.38E-07	1.80E-06	5.895169381	UP	FAM27E3
AC005083.1	1.481275632	-3.219578151	5.233812376	4.49E-07	1.84E-06	5.852619525	UP	AC005083.1
AC007128.1	1.400322943	-5.303055758	5.227056977	4.64E-07	1.89E-06	5.843299735	UP	AC007128.1
DUXAP8	1.50243852	-0.742605074	5.213319077	4.95E-07	2.01E-06	5.641356468	UP	DUXAP8
FAM218A	1.193920904	-4.602218681	5.160356242	6.34E-07	2.52E-06	5.544308911	UP	FAM218A
PACRG_AS1	-1.335113318	-3.990240932	-5.158392021	6.40E-07	2.54E-06	5.536409237	DOWN	PACRG-AS1
PRKG1_AS1	1.222393208	-5.159295981	5.117644122	7.73E-07	3.03E-06	5.353840889	UP	PRKG1-AS1
FGF14_AS2	-1.095167471	0.171254843	-5.093891851	8.63E-07	3.36E-06	5.073253578	DOWN	FGF14-AS2
FAM225B	1.0829438	-4.784254588	5.080336914	9.19E-07	3.56E-06	5.189043903	UP	FAM225B
LINC01234	2.401096062	-3.067574269	5.078394699	9.27E-07	3.60E-06	5.135468904	UP	LINC01234
EGFR_AS1	2.331966572	-2.00275124	5.036563007	1.12E-06	4.30E-06	4.912203271	UP	EGFR-AS1

GNAS_AS1 1.407600526 -4.163182853 5.002926894 1.31E-06 4.96E-
06 4.845629602 UP GNAS-AS1
TCL6 1.70810677 -3.784306962 4.998584038 1.34E-06 5.06E-06
4.817715635 UP TCL6
CASC20 1.677804068 -5.161403449 4.996163793 1.35E-06 5.10E-
06 4.818254391 UP CASC20
CYP4F26P 1.188823516 -5.214796814 4.970316065 1.52E-06 5.69E-
06 4.706411261UP CYP4F26P
LINC00661 1.41098651 -5.212114845 4.970174137 1.52E-06 5.69E-06
4.70548062 UP LINC00661
HORMAD2_AS1 -2.849054284 -0.056256117 -4.951798023 1.65E-06
6.14E-06 4.492329201 DOWN HORMAD2-AS1
LINC00648 1.962597716 -4.80900452 4.930624823 1.82E-06 6.72E-06
4.532771521 UP LINC00648
VIM_AS1 1.088425438 -1.718009647 4.930009164 1.83E-06 6.73E-
06 4.455756993 UP VIM-AS1
LINC01143 1.041835877 -5.678648234 4.911277664 1.99E-06 7.27E-06
4.450098468 UP LINC01143
AC009264.1 1.163257866 -5.720125548 4.893849135 2.15E-06 7.82E-
06 4.374940839 UP AC009264.1
LINC00173 1.192895499 -3.490847737 4.893287727 2.16E-06 7.84E-
06 4.361745721 UP LINC00173
USP12_AS2 -1.148807721 -3.925665097 -4.884189211 2.24E-06 8.14E-
06 4.335698892 DOWN USP12-AS2
LINC01139 2.21114628 -3.911466334 4.876441815 2.32E-06 8.41E-06
4.285765077 UP LINC01139
LINC01135 -1.130116995 -4.014795426 -4.853331767 2.58E-06 9.25E-
06 4.203866405 DOWN LINC01135
ZNF793_AS1 1.475985796 -2.019396811 4.8412691 2.72E-06 9.73E-
06 4.08339312 UP ZNF793-AS1
LINC01136 1.245032952 -3.39505783 4.812793392 3.09E-06 1.09E-05
4.014967577 UP LINC01136
GDNF_AS1 -1.918477151 -2.694895576 -4.811914889 3.10E-06 1.10E-

05 4.013008529 DOWN GDNF-AS1
LINC01116 1.333441849 -1.743208695 4.789773684 3.42E-06 1.20E-05 3.852832906 UP LINC01116
KCNMB2_AS1 1.916624025 -2.259532223 4.76857156 3.76E-06 1.31E-05 3.777146035 UP KCNMB2-AS1
TRPC7_AS1 1.259578387 -5.339812947 4.767848529 3.77E-06 1.32E-05 3.840019824 UP TRPC7-AS1
KIF25_AS1 1.385278199 -4.864744091 4.758201324 3.93E-06 1.37E-05 3.79977711 UP KIF25-AS1
AC114498.1 -1.144500744 -2.426913358 -4.753298034 4.02E-06 1.40E-05 3.759964915 DOWN AC114498.1
FAM27C 1.25872717 -4.317118924 4.736574968 4.33E-06 1.50E-05 3.708051506 UP FAM27C
FOXD3_AS1 1.2181479 -5.538446703 4.732261779 4.41E-06 1.52E-05 3.690283306 UP FOXD3-AS1
LINC01559 1.774321445 -4.599029699 4.731471147 4.42E-06 1.53E-05 3.68599301 UP LINC01559
GACAT2 1.158558443 -5.647139971 4.713721008 4.78E-06 1.64E-05 3.61259029 UP GACAT2
LINC01182 -1.517268459 -4.199578429 -4.699313449 5.09E-06 1.74E-05 3.554755737 DOWN LINC01182
SLC22A18AS -1.255541075 1.657727546 -4.690499143 5.29E-06 1.80E-05 3.187634282 DOWN SLC22A18AS
PWRN1 -1.179067006 -5.237816364 -4.670823365 5.77E-06 1.95E-05 3.435386218 DOWN PWRN1
C8orf31 1.194151726 -2.431810938 4.618241529 7.24E-06 2.40E-05 3.172229439 UP C8orf31
MNX1_AS2 1.212284046 -5.345584391 4.61131985 7.46E-06 2.47E-05 3.189735679 UP MNX1-AS2
LINC00958 1.603110621 -4.938173391 4.609015075 7.54E-06 2.49E-05 3.18009351 UP LINC00958
AC131097.4 -1.621998948 -3.920005753 -4.599700726 7.85E-06 2.59E-05 3.143238193 DOWN AC131097.4

PTGES2_AS1 1.081080998 -3.184533091 4.571517743 8.86E-06
2.90E-05 3.008606154 UP PTGES2-AS1
INHBA_AS1 -1.115602355 -2.731853506 -4.567006062 9.03E-06
2.95E-05 2.998328482 DOWN INHBA-AS1
TTC39A_AS1 1.313848672 -3.959388327 4.557859052 9.39E-06
3.06E-05 2.96702096 UP TTC39A-AS1
FAM83H_AS1 1.062765594 -0.325163618 4.55104075 9.67E-06 3.14E-05 2.763320065 UP FAM83H-AS1
LINC00272 -1.168006068 -5.602440667 -4.545234759 9.91E-06 3.22E-05 2.919306169 DOWN LINC00272
AC104534.2 1.083077069 -2.680359731 4.502849317 1.19E-05 3.80E-05 2.713869671 UP AC104534.2
AC099552.4 1.059424167 -5.713503889 4.500061567 1.20E-05 3.84E-05 2.737119491 UP AC099552.4
TGFB2_AS1 1.234554356 -3.687894749 4.498874222 1.21E-05
3.86E-05 2.725002611 UP TGFB2-AS1
UCA1 2.213593724 -2.699713618 4.490983613 1.25E-05 3.98E-05
2.64630391 UP UCA1
NCRNA00250 -1.102320666 -4.439270362 -4.478077222 1.32E-05
4.19E-05 2.651595565 DOWN NCRNA00250
H19 2.007612428 5.135472972 4.429542924 1.62E-05 5.06E-05
1.732757781 UP H19
HOXC_AS1 1.465756278 -5.00545055 4.406874389 1.78E-05 5.52E-05
2.366394352 UP HOXC-AS1
AL589743.1 1.152714134 -3.202207693 4.406606187 1.78E-05 5.53E-05 2.345712895 UP AL589743.1
AC002511.2 1.143655061 -5.312240302 4.404632767 1.79E-05 5.57E-05 2.357606696 UP AC002511.2
LINC01436 1.894103647 -2.30168109 4.399745887 1.83E-05 5.68E-05
2.273051759 UP LINC01436
LINC01587 1.555983975 -4.093774992 4.39586203 1.86E-05 5.76E-05
2.31793306 UP LINC01587
ZNF295_AS1 -1.035240796 -3.154157829 -4.372765113 2.05E-05

6.30E-05 2.228963483 DOWN ZNF295-AS1

LINC00535 -1.41501585 -1.592407497 -4.344904558 2.30E-05 7.01E-05 2.063545204 DOWN LINC00535

LINC00943 1.092932578 -4.417195948 4.338358165 2.36E-05 7.18E-05 2.097367701 UP LINC00943

TTTY14 -2.198557262 -2.140328431 -4.338339583 2.36E-05 7.18E-05 2.06604055 DOWN TTTY14

LINC00668 1.678994215 -4.729380716 4.330892224 2.44E-05 7.38E-05 2.067294738 UP LINC00668

CTD_2297D10.2 1.700741103 -4.289469495 4.324645075 2.50E-05 7.55E-05 2.039992756 UP CTD-2297D10.2

LINC00504 -1.258133164 -2.120176691 -4.306391675 2.69E-05 8.10E-05 1.940017982 DOWN LINC00504

LINC01010 -1.309274552 -2.286937988 -4.291246181 2.87E-05 8.57E-05 1.888777222 DOWN LINC01010

LINC01239 -1.206920884 -1.737177379 -4.28424254 2.95E-05 8.80E-05 1.833900342 DOWN LINC01239

LEF1_AS1 1.062229069 -3.403407669 4.278778083 3.02E-05 8.98E-05 1.85259423 UP LEF1-AS1

DPP10_AS1 1.848767233 -4.946174035 4.253964999 3.34E-05 9.86E-05 1.769274229 UP DPP10-AS1

LINC00323 1.047483286 -4.782081096 4.223984232 3.77E-05 0.000110279 1.656289956 UP LINC00323

SSTR5_AS1 2.478174483 -2.703164749 4.185559403 4.40E-05 0.000127313 1.443815102 UP SSTR5-AS1

CASC22 -1.340103236 -4.653065114 -4.181205238 4.48E-05 0.000129356 1.49423137 DOWN CASC22

LY86_AS1 -1.272550804 -4.79026499 -4.146787004 5.14E-05 0.000146678 1.364171589 DOWN LY86-AS1

PCAT18 -1.151327371 -4.700975147 -4.107966957 6.00E-05 0.000169091 1.218927927 DOWN PCAT18

LINC00885 -1.645998737 -2.947361273 -4.039999054 7.84E-05 0.000216516 0.957143123 DOWN LINC00885

FAM230C 1.328259775 -5.500061231 4.039404229 7.86E-05
0.00021697 0.962436376 UP FAM230C
LINC00944 1.420803285 -2.403931445 4.038468843 7.89E-05
0.000217641 0.90340837 UP LINC00944
NPSR1_AS1 1.836426648 -3.201162046 4.032801628 8.06E-05
0.000222161 0.904720683 UP NPSR1-AS1
LINC00880 -1.04087474 -3.268554319 -4.00970025 8.82E-05 0.000241277
0.85217262 DOWN LINC00880
HAS2_AS1 1.063671553 -4.67453692 3.999353998 9.18E-05
0.000250558 0.81702794 UP HAS2-AS1
GPR1_AS 1.390999719 -5.33640108 3.987451703 9.62E-05 0.00026157
0.772489442 UP GPR1-AS
LINC00858 1.123050741 -5.561709729 3.982594821 9.80E-05
0.000266086 0.754593799 UP LINC00858
U91319.1 -1.749577004 -1.472070511 -3.927322128 0.000121283
0.000324066 0.48903452 DOWN U91319.1
PCDH9_AS2 -1.092618265 -5.785890916 -3.903852199 0.000132675
0.000351326 0.468948568 DOWN PCDH9-AS2
LINC00239 1.267156632 -3.189803865 3.87624715 0.000147374
0.000387293 0.348429927 UP LINC00239
LINC01091 1.050232163 -1.999795374 3.847050139 0.000164598
0.00042874 0.19940619 UP LINC01091
AC016710.1 1.371332889 -5.376068926 3.808992727 0.000189927
0.000488544 0.134829774 UP AC016710.1
AC010969.1 -1.236043471 -4.082850658 -3.803406101 0.000193942
0.000497856 0.117374807DOWN AC010969.1
LINC00942 1.792180837 -3.564301678 3.776864171 0.000214143
0.000544799 0.002083226 UP LINC00942
AC034243.1 -1.186537037 -2.83217924 -3.765722166 0.000223203
0.000566237 -0.024797761 DOWN AC034243.1
LINC01124 -1.223810524 1.480399066 -3.759419316 0.000228488
0.000578586 -0.373264669 DOWN LINC01124
FIRRE 1.140545608 -3.365069071 3.754124502 0.000233018

0.000589185 -0.071813041 UP FIRRE

CASC9 2.096359436 -2.662087873 3.75406943 0.000233066

0.000589217 -0.116911813 UP CASC9

LINC00491 1.162597807 -5.351111349 3.736626485 0.000248603

0.00062555 -0.116290328 UP LINC00491

LINC00689 1.356238521 -4.458591227 3.7296631140.000255076

0.000640756 -0.140765072 UP LINC00689

FAM87A 1.070167789 -4.247357103 3.704927554 0.000279377

0.000696972 -0.225605961 UP FAM87A

AGAP1_IT1 1.051482628 -1.811041881 3.698913732 0.000285608

0.000710958 -0.328232647 UP AGAP1-IT1

CRNDE 1.093779402 0.574594519 3.668342699 0.000319362

0.000787198 -0.635430777 UP CRNDE

AC004012.1 -1.056652683 -2.637564718 -3.665198602 0.000323039

0.00079536 -0.374941025 DOWN AC004012.1

AFAP1_AS1 1.832106591 -1.792500412 3.65853668 0.000330964

0.000812279 -0.479111175 UP AFAP1-AS1

LINC01101 1.031233988 -4.681226148 3.610044148 0.000394417

0.000955461 -0.545218264 UP LINC01101

MIR646HG 1.164900632 -3.215848247 3.590675668 0.00042283

0.001019236 -0.632091715 UP MIR646HG

LINC01474 -1.048227749 -1.805727419 -3.584052296 0.000432981

0.001041289 -0.687744586 DOWN LINC01474

PCAT14 1.140383025 -4.664171326 3.533323171 0.000518685

0.001226921 -0.799763002 UP PCAT14

LINC01146 -1.462602844 2.125723125 -3.502232261 0.000578838

0.001357795 -1.317108018 DOWN LINC01146

LINC00540 1.124793268 -4.581140194 3.452084508 0.000689838

0.001592247 -1.063925841 UP LINC00540

ELOVL2_AS1 -1.006864939 -1.702496142 -3.41130513 0.000794492

0.001811946-1.258146297 DOWN ELOVL2-AS1

AC099684.1 -1.053253313 -1.780792437 -3.388378052 0.000859677

0.001950379 -1.32551484 DOWN AC099684.1

LINC01194 1.36005743 -4.816623356 3.367808828 0.000922381
0.002078245 -1.332483253 UP LINC01194

FAM201A 1.511034964-2.013556081 3.345336868 0.000995754
0.002230536 -1.484106404 UP FAM201A

LINC00189 1.127803358 -3.110660628 3.328302989 0.001054962
0.002351275 -1.48131865 UP LINC00189

PWAR5 1.04961204 -3.189349158 3.310994601 0.0011184760.002478513
-1.530742597 UP PWAR5

AC096559.1-1.010491567 -4.150409696 -3.305392778 0.001139784
0.002519894 -1.525691133 DOWN AC096559.1

LINC01320 -1.463516967 -1.427788034 -3.086790011 0.002335566
0.00484305 -2.261509165 DOWN LINC01320

LINC01152 1.172630668 -2.833662936 3.078660265 0.002396993
0.004960534 -2.244192595 UP LINC01152

TEX41 1.152619998 -1.051867589 3.034047968 0.002761438
0.005644899 -2.481521025 UP TEX41

LINC01426 1.023884031 -0.091646425 3.016245969 0.002920611
0.005944872 -2.616620531 UP LINC01426

AC079466.1 1.858084784 -1.55404483 2.910094957 0.00405829
0.008015039 -2.804233819 UP AC079466.1

ZFPM2_AS1 1.128953504 -1.102011573 2.743782176 0.006673678
0.012582922 -3.275867523 UP ZFPM2-AS1

HOTTIP -1.218594922 -2.217272022 -2.62045962 0.009513474
0.017306896 -3.482663664 DOWN HOTTIP

LINC01549 -1.045746182 -2.396997931 -2.513993652 0.012793432
0.022668811-3.737187727 DOWN LINC01549

LINC00221 1.427973557 -3.740142972 2.5118346920.012869293
0.022792868 -3.728011858 UP LINC00221

ST8SIA6_AS1 -1.341390773 -0.99617454-2.374387482 0.018605464
0.031781746 -4.154139514 DOWN ST8SIA6-AS1

XIST 1.64438601 -1.819562501 2.306921251 0.022170726
0.037252863 -4.292740246 UP XIST

LINC01291 1.050838842 -1.815501186 2.223661724 0.027385661

0.044966555	-4.464552401	UP	LINC01291

Supplementary table 5.

Genes	bate	Hazard_Ratio	lower.95	upper.95	z_pvalue	Wald_pvalue	Likelihood_pvalue
TMEM220_AS1	-0.20618273	0.813684378	0.727464288	0.910123393	0.000308703	0.000308703	0.000861746
F11_AS1	-0.135108732	0.873620916	0.795346633	0.959598587	0.004786597	0.004786597	0.007036263
NAV2_AS4	-0.206935958	0.813071719	0.718480157	0.920116742	0.001040675	0.001040675	0.000669092
MIR100HG	-0.148802361	0.861739412	0.783124617	0.948246036	0.002297913	0.002297913	0.002571421
SPATA3_AS1	0.160573594	1.174184183	1.048154992	1.315367008	0.005574366	0.005574366	0.006403728
SNHG1	0.345635753	1.412887882	1.17358917	1.700980392	0.000261654	0.000261654	0.000294473
DDX11_AS1	0.357973853	1.430428219	1.227284638	1.667196692	4.64E-06	4.64E-06	3.31E-06
LHFPL3_AS2	0.104122523	1.109736414	1.045248537	1.17820295	0.000652536	0.000652536	0.001076259
LINC01352	-0.203420749	0.815934865	0.724528715	0.918872765	0.000791738	0.000791738	0.000879986
LINC00205	0.252804002	1.287630879	1.108033283	1.49633888	0.00097215	0.00097215	0.000946857
LINC01537	-0.16399018	0.848750357	0.736250724	0.978440013	0.023797018	0.023797018	0.02382415
PIK3CD_AS2	0.175593815	1.191953807	1.092971567	1.299900125	7.19E-05	7.19E-05	9.49E-05
PRR7_AS1	0.235609899	1.265680471	1.126889876	1.421564865	7.01E-05	7.01E-05	0.000103987
LINC00402	-0.196058796	0.821963905	0.741011025	0.911760606	0.000210346	0.000210346	0.000124913
FAM225A	0.177913003	1.19472138	1.071868168	1.331655532	0.001311007	0.001311007	0.001457135

SNHG3 0.371751735 1.450272885 1.253530813 1.677893691 5.80E-07 5.80E-07 1.26E-06

MIR99AHG -0.11300191 0.893148954 0.803003856 0.993413727 0.037370232 0.037370232 0.04120259

C2orf27A 0.254968862 1.290421439 1.153914847 1.443076581 7.84E-06 7.84E-06 7.19E-06

RAB11B_AS1 -0.171252658 0.842608655 0.712852537 0.995983472 0.044735427 0.044735427 0.045481495

MIR210HG 0.2645408111.302832592 1.173697303 1.446175907 6.79E-07 6.79E-07 7.83E-07

FBXL19_AS1 0.14363713 1.1544651121.01715063 1.310316934 0.026204694 0.026204694 0.02594631

LINC01224 0.13603757 1.145724938 1.070850298 1.225834867 7.98E-05 7.98E-05 8.38E-05

SNHG4 0.209679162 1.233282313 1.096734374 1.386831031 0.000461316 0.000461316 0.000403174

AC005592.20.278433857 1.321059223 1.179357175 1.479787046 1.51E-06 1.51E-06 4.02E-06

SNHG12 0.348007036 1.416242214 1.177141578 1.703908897 0.000225525 0.000225525 0.000288036

DGUOK_AS1 0.234706219 1.264537217 1.083199336 1.476232787 0.002959405 0.002959405 0.003154499

TAT_AS1 -0.107758229 0.897844643 0.810966758 0.994029652 0.037959655 0.037959655 0.03643447

BOLA3_AS1 0.133893378 1.143270915 1.00436131 1.301392608 0.042784552 0.042784552 0.045742057

LINC00324 -0.292893311 0.746101737 0.625147632 0.890458147 0.0011720890.0011720890.001376397

MYLK_AS10.299619574 1.349345384 1.158135789 1.57212391 0.000121531 0.000121531 0.000137954

LINC01252 -0.114486958 0.89182357 0.80623606 0.986496783 0.026143725 0.026143725 0.027597757

CPS1_IT1 -0.14940249 0.861222412 0.784404748 0.945562918

0.001723071 0.001723071 0.001319144
MIR181A2HG 0.135868444 1.1455311821.021365935 1.284790928
0.020279981 0.020279981 0.022623764
FOXD2_AS1 0.279401452 1.322338093 1.165506573 1.500272991
1.44E-05 1.44E-05 6.08E-06
CASC15 0.155604017 1.168363459 1.069918433 1.275866581
0.000530603 0.000530603 0.00060034
LINC00628 0.204999079 1.227523935 1.078724617 1.396848637
0.0018749110.0018749110.001935128
GUSBP11 0.177927065 1.19473818 1.036013208 1.377781006
0.014428173 0.014428173 0.013725015
AC006538.1 0.155136443 1.16781729 1.0405511151.310648947
0.008409463 0.008409463 0.00960488
NRSN2_AS1 0.262939442 1.300747946 1.1182626371.513012385
0.000651437 0.000651437 0.000727507
LINC00654 0.1611934921.174912283 1.059982787 1.302303103
0.002147204 0.002147204 0.002136154
LINC00941 0.103130618 1.108636208 1.015817253 1.209936372
0.020791808 0.020791808 0.024584076
LINC00632 0.183838678 1.201821927 1.1109337721.30014586 4.61E-06
4.61E-06 3.82E-05
RNF144A_AS1 0.155519814 1.168265084 1.079328468 1.264530072
0.0001183170.0001183170.000286373
AL133493.2 0.1179713461.12521187 1.047808671 1.208332958
0.0011775580.0011775580.001923414
LINC01106 0.139192571 1.149345409 1.028212622 1.284748739
0.01430185 0.01430185 0.014682169
LINC01353 0.2112963621.23527839 1.08334594 1.40851841 0.001602271
0.001602271 0.001836676
JMJD1C_AS1 0.203926692 1.226208259 1.056018076 1.423826665
0.007474901 0.007474901 0.007679315
LINC00664 0.139658958 1.149881574 1.052547097 1.256217075
0.00196915 0.00196915 0.002844147

FAM182B	0.192220225	1.211937386	1.088854886	1.348932945	0.000434976	0.000434976	0.000539392
GAS5	0.207889053	1.231076578	1.055138693	1.436351023	0.008239568	0.008239568	0.008321667
LINC01138	0.379032706	1.460870814	1.240043593	1.721022994	5.82E-06	5.82E-06	2.91E-06
RUNDC3A_AS1	0.167789986	1.182688204	1.046735642	1.336298614	0.007079407	0.007079407	0.009098224
SLC25A30_AS1	-0.241619572	0.785354892	0.673900307	0.915242655	0.001973989	0.001973989	0.002101065
CASC8	0.147175487	1.158557257	1.052349456	1.275484023	0.002699066	0.002699066	0.00452482
LINC01191	0.13333637	1.142634282	1.008048838	1.295188341	0.037038309	0.037038309	0.042390315
HCG15	0.174709755	1.190900513	1.062831503	1.334401576	0.00261493	0.00261493	0.002394101
LINC01094	0.20994045	1.233604596	1.102518956	1.38027586	0.000249594	0.000249594	0.000241701
AC006369.2	-0.224331608	0.799050119	0.698781596	0.913706223	0.001041323	0.001041323	0.001014396
ZNF350_AS1	0.165955678	1.180520777	1.014722359	1.373409477	0.031615488	0.031615488	0.034013563
MIR137HG	0.269438631	1.309229284	1.162650063	1.474288243	8.68E-06	8.68E-06	8.28E-05
PTPRG_AS1	0.133339906	1.142638322	1.024598248	1.274277345	0.016540619	0.016540619	0.016537226
LINC01096	0.198021898	1.218989087	1.089854668	1.363424352	0.000528235	0.000528235	0.001601508
SOCS2_AS1	-0.156731691	0.854933415	0.765324985	0.955033688	0.005530593	0.005530593	0.006155733
AC092198.1	0.129422536	1.13817094	1.011868407	1.280238695	0.031039776	0.031039776	0.036678994
ZIM2_AS1	0.131904205	1.140999012	1.036117512	1.256497193			

0.007336678 0.007336678 0.009366811
LINC00671 -0.12481236 0.88266251 0.791853544 0.983885357 0.02424319
0.02424319 0.02697632
FOXP4_AS1 0.154107791 1.16661663 1.057095026 1.287485353
0.002184964 0.002184964 0.002407433
LINC01572 0.145101578 1.156157004 1.027243294 1.301248717
0.016146996 0.016146996 0.016500123
LINC00501 0.241023173 1.272550524 1.143784592 1.415812774
9.50E-06 9.50E-06 3.06E-05
AC007952.5 0.131149911 1.140138687 1.021169962 1.272967551 0.0196718
0.0196718 0.025121094
LINC01063 0.309337388 1.362521991 1.191715941 1.557809301
6.00E-06 6.00E-06 9.01E-06
PRSS30P 0.12979737 1.138597646 1.015892209 1.276124167
0.025682888 0.025682888 0.028898484
LINC00622 0.187106664 1.205755889 1.07153922 1.356783995
0.00188652 0.00188652 0.002290899
BBOX1_AS1 0.106007149 1.111829825 1.030217467 1.199907397
0.00642409 0.00642409 0.009234435
AC006037.2 -0.180626337 0.834747215 0.735877143 0.94690115
0.00498153 0.00498153 0.003483277
MIR663AHG 0.144537208 1.155504689 1.078788963 1.237675886
3.73E-05 3.73E-05 0.000147319
USP2_AS1 0.132392548 1.141556347 1.0369096 1.256764229
0.006958792 0.006958792 0.006652586
FAM27E3 0.259317819 1.296045647 1.136436786 1.478071055
0.000110003 0.000110003 0.000166183
AC005083.1 0.100574788 1.105806339 1.02236236 1.196060915
0.011989977 0.011989977 0.015279986
AC007128.1 0.197700286 1.218597108 1.11503426 1.331778732 1.28E-
05 1.28E-05 3.86E-05
DUXAP8 0.155382638 1.168104837 1.073694539 1.270816662
0.000301966 0.000301966 0.000202912

PACRG_AS1	-0.139065382	0.870171133	0.764821386	0.990032202	0.034676703	0.034676703	0.030424332
FGF14_AS2	0.137412897	1.147301769	1.013653743	1.298570994	0.029662043	0.029662043	0.026989098
FAM225B	0.149735218	1.161526651	1.007702992	1.338831155	0.038844385	0.038844385	0.044910991
TCL6	0.112313816	1.118863923	1.037796647	1.206263752	0.003425449	0.003425449	0.004382105
CASC20	0.103975677	1.109573466	1.034815293	1.189732395	0.003482542	0.003482542	0.004979232
LINC00661	0.14373945	1.154583243	1.062861788	1.254219956	0.000665239	0.000665239	0.001694194
AC009264.1	0.192648069	1.212456018	1.086530515	1.352975895	0.000574733	0.000574733	0.001618631
LINC01136	0.197160603	1.21793963	1.094333673	1.355506989	0.000305055	0.000305055	0.000343098
LINC01116	0.170651402	1.186077213	1.083342301	1.298554624	0.00022275	0.00022275	0.000382068
KIF25_AS1	0.182552974	1.200277732	1.105258071	1.303466287	1.44E-05	1.44E-05	0.00011376
FAM27C	0.19170531	1.211313502	1.088173837	1.348387869	0.000456862	0.000456862	0.000843406
LINC01559	0.101033644	1.106313862	1.038459864	1.178601508	0.001756621	0.001756621	0.003665384
C8orf31	0.109843124	1.116102967	1.007305492	1.236651485	0.035811455	0.035811455	0.03765972
LINC00958	0.148949765	1.160614685	1.084376854	1.242212466	1.73E-05	1.73E-05	0.000101052
TTC39A_AS1	0.119019138	1.126391475	1.018479175	1.245737552	0.020540903	0.020540903	0.021698547
TGFB2_AS1	0.158178904	1.17137574	1.05862643	1.296133447	0.002189206	0.002189206	0.002882681
HOXC_AS1	0.113126141	1.119773173	1.021888471	1.22703406	0.015354898		

0.015354898 0.02074862

AL589743.1 0.1172105071.12435609 1.020037557 1.239343206

0.018309026 0.018309026 0.018553532

AC002511.2 0.218373362 1.244051462 1.1180327131.38427438 6.14E-05

6.14E-05 0.000268036

LINC01436 0.10296152 1.108448756 1.043404293 1.177548005

0.00084672 0.00084672 0.001220667

LINC01587 0.105975723 1.1117948851.032003772 1.197755183

0.005286606 0.005286606 0.006495036

LINC00943 0.140500355 1.150849488 1.020857676 1.297393923

0.02158852 0.02158852 0.024055832

LINC00668 0.124263798 1.132314534 1.06154858 1.207797954

0.000160677 0.000160677 0.000320391

HAS2_AS1 0.151972202 1.164127876 1.044483201 1.297477748

0.006023122 0.006023122 0.009334646

GPR1_AS 0.1107209211.1170831091.037002891 1.203347342

0.003530433 0.003530433 0.00727255

LINC00858 0.13685758 1.146664829 1.03552165 1.269737075

0.008513526 0.008513526 0.012263175

LINC00239 0.100457583 1.105676741 1.008533773 1.212176617

0.032268033 0.032268033 0.034751546

LINC01091 0.130074654 1.138913405 1.029569473 1.259870051

0.0115426310.0115426310.012478282

LINC00942 0.100650374 1.105889927 1.050016904 1.164736039

0.000141757 0.000141757 0.000392256

FIRRE 0.174822874 1.191035235 1.0847611861.307720953

0.000246259 0.000246259 0.000254878

LINC00491 0.102154562 1.107554645 1.023831455 1.198124247

0.010858085 0.010858085 0.014637683

MIR646HG 0.129082954 1.137784504 1.047293794 1.236094003

0.002267001 0.002267001 0.002326867

PCAT140.124274367 1.132326501 1.027796207 1.247487873

0.0119113390.0119113390.018063365

LINC00540 0.12097066 1.128591799 1.0454531161.218342009
0.001945021 0.001945021 0.00349221
ZFPM2_AS1 0.132391295 1.141554917 1.071492575 1.216198469
4.19E-05 4.19E-05 4.31E-05
